# Statistical approaches and software for clustering islet cell functional heterogeneity

**DOI:** 10.1080/19382014.2016.1150664

**Published:** 2016-02-24

**Authors:** Quin F. Wills, Tobias Boothe, Ali Asadi, Ziliang Ao, Garth L. Warnock, Timothy J. Kieffer, James D. Johnson

**Affiliations:** a Wellcome Trust Center for Human Genetics, University of Oxford, Oxford, United Kingdom; b Weatherall Institute of Molecular Medicine, University of Oxford, Oxford, United Kingdom; c Department of Cellular and Physiological Sciences, Life Sciences Center, University of British Columbia, Vancouver, Canada; d Department of Surgery, University of British Columbia, Vancouver, Canada

**Keywords:** calcium signals, insulin, oscillations

## Abstract

Worldwide efforts are underway to replace or repair lost or dysfunctional pancreatic β-cells to cure diabetes. However, it is unclear what the final product of these efforts should be, as β-cells are thought to be heterogeneous. To enable the analysis of β-cell heterogeneity in an unbiased and quantitative way, we developed model-free and model-based statistical clustering approaches, and created new software called TraceCluster. Using an example data set, we illustrate the utility of these approaches by clustering dynamic intracellular Ca^2+^ responses to high glucose in ∼300 simultaneously imaged single islet cells. Using feature extraction from the Ca^2+^ traces on this reference data set, we identified 2 distinct populations of cells with β-like responses to glucose. To the best of our knowledge, this report represents the first unbiased cluster-based analysis of human β-cell functional heterogeneity of simultaneous recordings. We hope that the approaches and tools described here will be helpful for those studying heterogeneity in primary islet cells, as well as excitable cells derived from embryonic stem cells or induced pluripotent cells.

## Introduction

Diabetes is the result of pancreatic islet β-cell dysfunction and/or death.^1,2^ Clinical studies demonstrate the therapeutic potential of islet cell replacement for diabetes,^3,4^ and therefore many academic and industrial laboratories have sought to produce functional β-cells from sources such as embryonic stem cells. Up to this point, single-cell analysis suggests that only a small percentage of partially functional cells have been produced.^5^ Among the many technical challenges is the fact that even the most sophisticated published protocols generate highly heterogeneous cultures of cells.^5^ This necessitates high-throughput analysis at the single-cell level. To do this, current functional studies typically compare ‘representative example’ Ca^2+^ signal traces between stem cell derived cells (or groups of cells) to primary human islet cell preparations of variable quality.^5,6^ The reason for assaying Ca^2+^ flux is that the main function of a pancreatic β-cell is glucose-stimulated insulin exocytosis, a process that requires voltage-dependent Ca^2+^ signals caused by the closure of ATP-sensitive K^+^ channels.^7^ The analyses of mRNA, protein, and metabolites can further assist with the comparison of stem cell derived cultures to human islets,^5,6,8^ but these can be misleading as preparations are typically of mixed cell types. New breakthroughs in single-cell transcriptomics, epigenomics and proteomics promise to enable snapshots of cell-to-cell heterogeneity.^9^ However, the continuous analysis of single living cells, including real-time measurements of signal transduction events, is still required to assess true phenotypic heterogeneity.

The development of surrogate β-cells is also hampered by limited understanding of the function and functional heterogeneity in fully responsive human islets from healthy donors. In other words, it is not clear exactly what the features are of the target cell type, or cell types, that need to be generated. This question is not trivial, and attempts to answer it reveal critical gaps in the understanding of human islet cell physiology. Indeed, heterogeneity in cell state and cell fate remains a poorly understood aspect of β-cell physiology, despite several studies.^10-18^ Importantly, it has been proposed that functional heterogeneity itself may be critical for the ideal performance of the intact islet.^19-21^ Studies in this realm typical focus on rodent β-cells,^11,24,25^ as the majority of human islet preparations do not match the quality that can be achieved under the controlled conditions of rodent islet isolation, where we and others have documented low levels of cell death in culture.^9^ Moreover, the majority of human islet donors are older, with β-cells that have endured decades of diets of variable quality.

Here, we present TraceCluster (https://jimjohnsonsci.shinyapps.io/TraceCluster), a software app designed to enable the unbiased, high-throughput analysis of functional heterogeneity between islets cells using model-free and model-based clustering. We demonstrate the utility of these approaches and the new software on a rare set of ∼300 high quality simultaneous recordings of human islet cells from a young donor and find two functional groupings of β-cell like responses and one functional grouping of non-β-cell responses.

## Methods

### Primary culture of human islet cells

**Figure 1.**
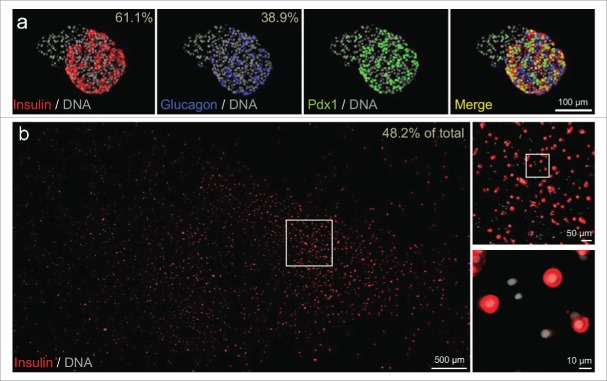
Cell composition of intact islets and dispersed islet cells of test sample. (A) Representative immunofluorescence staining of insulin, glucagon, and Pdx1 in intact islets from the donor. DNA counterstaining employed DAPI. The percentage of β-cells and α-cells (out of the major endocrine population i.e., β-cells or α-cells) is shown. Intact islets from this preparation contained attached non-endocrine cells; insulin positive cells were 37.3% of all cells and glucagon-positive cells were 23.7% of all cells. (B) Cultures of dispersed islet cells (including all endocrine and non-endocrine cells labeled with DAPI) contained 48.2% cells that stained robustly for insulin.

Human islets were isolated at the I.K. Barber Islet Transplantation Facility (Vancouver, Canada) from a healthy 11.8-year old male who died suddenly of head trauma (isolation code HR196). The warm ischemic time was 0 min and the cold ischemic time was 135 min. The pancreas weight was 61 g. The digestion time was 10 min. The final isolation yielded 378,006 IE (islet equivalents) with >90% purity. Some islets were embedded in agar, fixed, sectioned and examined with antibodies to insulin, glucagon or Pdx1 (Fig. 1a) as described elsewhere.^5,22^ Once isolated, the islets were transported to the laboratory within a few hours. Islets were gently dispersed as described.^23,24^ The resulting cells were plated on a coverslip (Fig. 1b) and incubated in RPMI media (containing 5.5 mM glucose and 100 IU/ml penicillin, 100µg/ml streptomycin, 10% fetal calf serum; pH 7.4 with NaOH) and maintained at 37°C, 5% CO_2_ and saturated humidity for 2 d.

### Ca^2+^ Imaging

Reagents were from Sigma (St. Louis, MO), unless otherwise indicated. Ca^2+^ imaging was performed in Ringer's solution containing (in mM): 5.5 KCl, 2 CaCl_2_, 1 MgCl_2_, 20 HEPES, 141 NaCl and 3 glucose according to standard protocols.^10,24,25^ Briefly, islet cell cultures were incubated with 1 µM Fura-2-AM (Molecular Probes, Eugene, OR) in RPMI for 30 minutes and rinsed in Ringer's solution for 30 minutes. Coverslips, in a 37°C chamber (∼300 µL), were continuously perfused with pre-heated solutions, to maximize control over the contents of the bath (especially ambient insulin levels). Cells were excited at 340 nm and 380 nm using a 300 W Sutter DG4 and imaged with a Roper CoolSnapHQ2 CCD camera, mounted on a Zeiss 200 m microscope (Intelligent Imaging Innovations). In total, 480 time points were recorded simultaneously for each of 291 islet cells over 80 min. All individual traces are provided in the Supplementary File, which is further explained in the Supplementary Information. Over the course of the experiment, cells were exposed to the following conditions: 20 min in 3 mM (low) glucose, 30 min in 20 mM (high) glucose, 15 min in 3 mM glucose, 10 min in 30 mM KCl, and 5 min in 3 mM glucose. The ratio of emission intensity >510 nm from excitations at 340 nm and 380 nm, was recorded for all cells.

### Normalization, quality control and analysis of live cell imaging data

The minor signal drift (as can be seen in the individual traces in the Supplementary File) was corrected per cell by estimating median increase in fluorescence signal per time step under baseline conditions (first 20 minutes). Correction for signal drift did not significantly alter the cell feature estimates described in this work. Fifteen cells (∼5%) were filtered from further analysis due to poor overall signal response, defined as a median KCl response less than 5% over baseline (median signal within the first 20 min). All data normalization, QC and analyses were performed within the R environment, details of which are provided in the Supplementary Information.

## Results

### Ca^2+^ imaging

The islets used to demonstrate the utility of our cluster analyses were isolated without complication and dispersed into single cells within 24 h (see Online Methods for details). Prior to dispersion, islets were collected for characterization of cellular composition using immunofluorescence staining for insulin, glucagon and PDX1, a critical regulator of islet-cell health and function^10,26,27^ (Fig. 1a). In these islets, β-cells accounted for 61.1% of the major endocrine population (i.e. β-cells and α-cells). Beta-cells were robustly positive for the PDX1 transcription factor (Fig. 1a). These observations compare favourably with the β-cell percentage reported by others^28,29^ and with our typical experience^5^ (see also Supplementary Fig. 5). After islet dispersion, coverslips demonstrated a large proportion of β-cells when compared to all cells (endocrine and non-endocrine, Fig. 1b). Collectively, these observations indicate that abundant and healthy dispersed β-cells were prepared from the donor.

**Figure 2.**
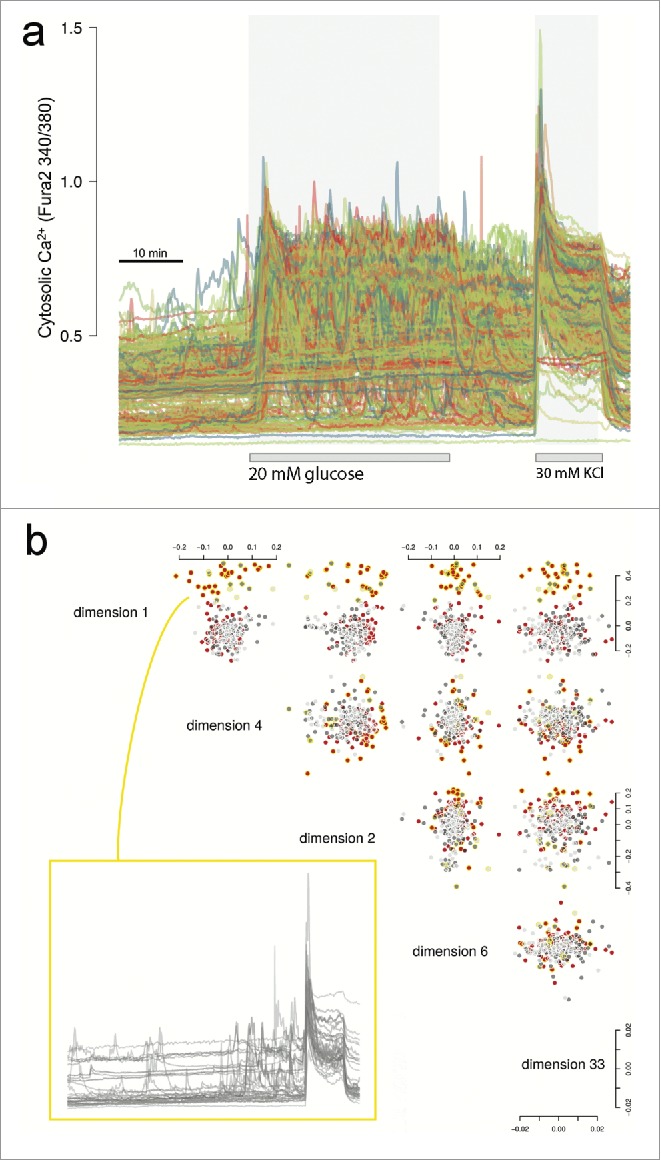
Ca^2+^ traces and exploratory data analysis. (A) Raw Ca^2+^ traces from Fura-2 stained islet cells showing the timed glucose perturbations and KCl depolarisation. (B) Cells were imaged in three microscopy fields (Supplementary Fig. 1). Shown are the five top ranked MDS dimensions of the raw Ca^2+^ traces based on large mean silhouette width between the fields (details provided in Supplementary Information). Cells are colored red, dark gray or light gray to reflect the three different microscopy fields. We noticed that the cluster of cells circled in yellow enriched for cells of one field due to inhomogeneous dispersion, but more importantly that these cells showed a distinct Ca^2+^ trace pattern irrespective of field. The traces are shown for these cells which are mostly/only responding during KCl depolarisation.

Ca^2+^ signals were simultaneously recorded from 291 cells, in 3 imaging fields, while sequentially exposed to either low glucose (3 mM) or high glucose (20 mM) before depolarization with 30 mM KCl (Fig. 2a, Supplementary Fig. 1). The recordings were technically sound and free of significant drift (correction for drift did not alter the main conclusions in this work). Importantly, because all cells were imaged in a single batch run, these data are free of experimental variability. Based on extensive experience working with human islets,^12,23,24,26,30-33^ it is estimated that these were among the highest quality islet cells we have studied. Therefore, we chose this data set as a test case for detailed analysis and development of a functional clustering strategy. Selected cells from this data set have also been compared to embryonic stem cell-derived cells in a recent publication.^5^

### Exploratory analysis

Our goal was to analyze functional responses to defined stimuli of human islet cells without using *a priori* knowledge of ‘cell type’. Multi-dimensional scaling (MDS) was initially used to explore low dimension projections of the intracellular Ca^2+^ traces acquired using Fura-2 imaging. The distance matrix for the projections was calculated as (1-ρ_ij_)/2, with ρ_ij_ being the Spearman correlation between cell *i* and *j*. As shown in Fig. 2b, we noticed that the first few dimensions suggest 2 clusters of cells, largely representing glucose responders (at low or high glucose) and non-responders (Fura-2 response only with or close to KCl perturbation). Non-responders were found to be in different proportions between the three imaging fields, suggesting nonhomogeneous dispersion of the cells (−log_10_P = 4.98 for the null hypothesis that the proportions across the fields are the same). However, no consistent spatial effect could be detected within the imaging fields (Supplementary Fig. 2).

### Ca^2+^ trace feature extraction and clustering

Next we sought to perform less naïve analyses of glucose responses in our human islet data set. To do this, nine Ca^2+^ response features were extracted from the Ca^2+^ imaging traces for further study. ‘High glucose response’ was defined as the median high glucose (20 mM) signal above baseline (median signal during the first 20 min), normalized as a ratio of median KCl signal above baseline. ‘High glucose oscillation’ was defined as the magnitude of oscillations during high glucose exposure, expressed as a ratio of the median absolute deviation (MAD) to median KCl response above baseline. ‘Low glucose oscillation’ was defined as the magnitude of oscillations during the first 2 low glucose exposures (3 mM before and after high glucose respectively), estimated in a similar manner to the high glucose oscillations. ‘Response speed’ was defined as the inverse of time to first oscillation peak with high glucose exposure, a measure of time responsiveness to perturbation. Number of ‘counted peaks’ during high glucose exposure were estimated as local maxima from each trace, with the constraint that a local maximum be considered a true peak if at least one third of the signal difference between median KCl and baseline glucose signal. Peak periodicity was used also to estimate the mean ‘oscillation frequency’. ‘Return to baseline’ was defined as the local minimum during the second low glucose exposure, normalized as a ratio of median KCl signal above baseline. ‘KCl response’ was defined as the maximum KCl response above baseline, normalized as a ratio of median KCl signal above baseline.

**Figure 3.**
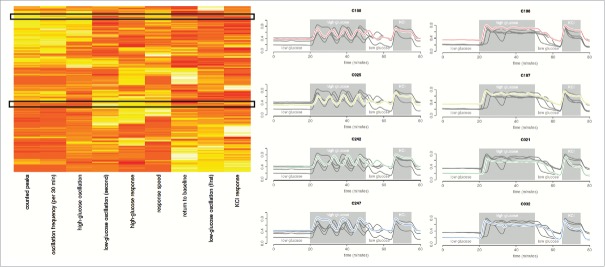
Hierarchical clustering of Ca^2+^ trace features and cells. The heatmap shown is reproduced from the provided software app (Supplementary Software), where users are able to select similar behaving cells and visualize their traces. In the heatmap, columns are clustered Ca^2+^ trace features while rows are clustered cells. Example traces from the software are also provided, where the first column of traces are cells from the top black box in the heatmap and the second column of traces are from the bottom black box. These are representative cells for the 2 β-like subtypes observed: oscillators and non-oscillators. The correlations between the features are provided in Supplementary Figure 3.

**Figure 4.**
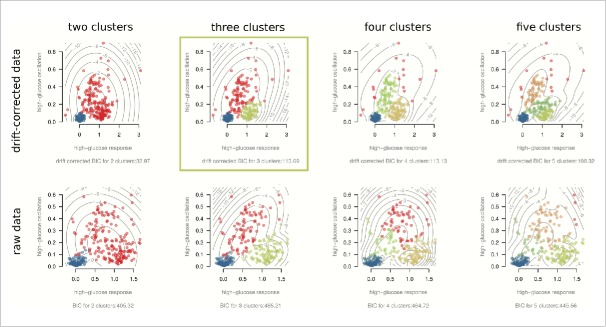
Model-based clustering of high glucose response and oscillations. All scatter-plots are of the same two features, though colored according to suggested clusters for drift-corrected (top row) and raw (bottom row) versions of the data. Gaussian mixture model BICs suggest optimal clustering of 3 major cell sub-types: non-responders (blue), oscillating responders (red) and non-oscillating responders (green). Drift-corrected BIC for four clusters was similar to that for three clusters, it did not suggest the same clustering pattern as the raw data. Three clusters therefore robustly describe the cell sub-type number. The drift-corrected 3-cluster plot highlighted is provided in the Trace Cluster software app (Supplementary Software), where users can upload their own data for analysis.

Hierarchical clustering of the features, as shown in Fig. 3a (and detailed in the Supplementary Information) pointed to 3 ‘feature’ clusters. The first feature cluster was comprised of cells that responded with clear oscillations when exposed to high glucose. The number of high glucose peaks and estimated oscillation frequency were, as expected, found to largely correlate (Supplementary Fig. 3). The lower the number of estimated peaks, the lower this correlation, suggesting a possible relationship between level and variability in oscillation frequency. Future studies will require longer high glucose exposures to study this relationship with sufficient statistical power. Overall, the defining relationship found in this cluster was the correlation between oscillation frequency and oscillation magnitude during high glucose exposure (Spearman ρ −log_10_P = 15.05). The second observed feature cluster (Fig. 3a) consists of the correlation between high glucose response, response speed (Spearman ρ −log_10_P = 11.44) and magnitude of low glucose oscillations after high glucose exposure (Spearman ρ −log_10_P = 7.61). This suggests a sub-type of predominantly rapid responders, usually with low/no oscillation during high glucose but some oscillation during subsequent low glucose instead of complete return to baseline. The final observed feature cluster (Fig. 3a) largely contains cells of little/no high glucose response. The same ‘non-responders’ were observed with the exploratory data analysis. Interestingly, these cells demonstrated greater relative low glucose oscillation magnitudes (during the initial 20 min) than high glucose oscillation magnitudes. This, despite their absolute magnitudes typically being much smaller than observed oscillation magnitudes for the oscillators in the first feature cluster. With 3 predominant clusters suggested by the data, we note that 2 features would suffice to capture the gross behavior in this sample. Parametric (Gaussian) mixture models of high glucose response and oscillation are presented in Fig. 4, with the Baysian Information Criterion (BIC) suggesting the same 3 dominant cell behaviors. Although tempting to further sub-divide the cells based on responsiveness to high glucose, as shown in Fig. 4, the agreement between the drift-corrected and raw data breaks down beyond three clusters.

Considering the different clustering strategies and normalized versions of the data, the most robust conclusion is that there is a non-responding (perhaps α-like cells) and responding (β-like cells) population, with a further sub-division of the responders into oscillators and non-oscillators. We anticipate that similar high quality samples from the community will enable the meaningful study of further functional sub-divisions. Moreover, we expect that post-hoc immunostaining and/or single-cell transcriptomics will enable characterization of functional islet cell sub-types. After filtering out cells that did not respond to KCl, the proportion of cells responding to high glucose (71%) was reasonably similar to the expected β-cell frequency within the endocrine cell population of this preparation (Fig. 1b). Interestingly, the non-oscillating cells of this population tended to possess a faster glucose response time. The 2 suggested cell states are reminiscent of the 2 β-cell sub-states, immature and mature, we have previously described in adult human and mouse β-cells based on mouse *Ins1* promoter activity and gene expression analysis.^6-8^ We speculate that the non-oscillators provide sensitivity in response while the oscillators syncronise the pulsatile insulin secretion. Worth noting is that the 15 cells (∼5%) filtered out for lack of any Fura-2 response, including to KCl, may represent non-excitable endothelial cells, exocrine cells, or unhealthy cells. However, the Fura-2 imaging technique tends to under-represent unhealthy cells, as they typically do not take up and cleave the Fura2-AM dye efficiently because AM esterase activity is energy-dependent.

## Discussion

The goals of the present study were to develop new statistical approaches for the analysis of functional heterogeneity from live-cell imaging data and to develop a software app, called TraceCluster, (Supplementary Software) to allow direct comparative analysis of similar data sets with the presented data. Details for the installation and running of the software app are provided in the Supplementary Information. The thorough characterization of Ca^2+^ response heterogeneity from simultaneously imaged cells controls for any technical variation in this unique high-quality reference resource of Ca^2+^ responses from a young healthy donor. We expect that these data may serve as a target for efforts to generate functionally young pancreatic β-cells and for studies of the systems biology of these sub-types using emerging single-cell ‘omic’ technologies. It is important to note that the goal of our study was not to characterize β-cell heterogeneity, but instead to merely provide the tools to do so. Robust characterization of β-cell heterogeneity will require highly powered studies with perhaps hundreds of donors.

Complete characterization of human islet cell functionality is essential for efforts aimed at β-cell replacement in type 1 diabetes or even type 2 diabetes,^5,6,34^ as it is not exactly clear how the desired output of cells from pluripotent stem cell differentiation protocols should behave. While there remains some controversy around how close the field is to producing true β-cells from human stem cells,^5,6,34^ it is clear that the field is now at the stage of optimizing the insulin secreting cells such that they exhibit relevant functional characteristics of *bona fide* human β-cells. Several key questions remain. Should we attempt to generate functionally homogeneous β-cell replacements, and if so, what functional characteristics are ideal? On the other hand, perhaps the goal should be to generate a range of functional islet cell types that more accurately mimics primary human islets. There is also the question of age. The peak age of type 1 diabetes diagnosis is between 10–14 years of age, so perhaps the ideal β-cell replacement cells would respond to glucose in a manner similar to the ‘teen-aged’ cells studied here. Little is known about age-dependent changes in human β-cell function, after the initial post-natal maturation.^35^ Evidence from rodent studies suggest some important functional differences between young mice, such as those from the most commonly studied ages (8–16 weeks of age), and mice older than 1 y.^36^ Previous studies have noted important differences in gene expression and proliferative capacity between young and old mice that mimic known differences in humans.^37-41^ It is notable that the oscillatory glucose responses observed in a large percentage of β-cells in the current study are not typical of published examples of β-cell responses from older human donors, which tend to exhibit brief, disorganized fluctuations^23,32,33,42-45^ or no fluctuations at all,^18^ much like dysfunctional mouse β-cells.^45^ Moreover, many of the Ca^2+^ responses in the current case exhibited the transient decrease in Ca^2+^ that initiates the response to glucose, known as ‘phase 0’,^46^ which is not typically observed in other studies of human β-cells.^32^ Collectively, our data demonstrate that β-cells from a healthy young donor can exhibit Ca^2+^ responses to glucose that resemble those observed commonly in mouse β-cells.^25,27,42,43,46,47^ Thus, it seems possible that some of the functional differences assumed previously to be species-specific, may actually represent the effects of age on human islets, or their quality. While there may be important morphologic, genomic and functional differences between islets of different species,^43,48-50^ the effects of age and quality should be considered in all studies. These speculations will require robust replication with multiple human and murine islet donors at multiple ages, which our new tools will enable.

Indeed, an obvious limitation of the experimental work described here is that it was conducted on a single donor, preventing any broad conclusions about the heterogeneity between donors. However, our main goal was to develop a statistical framework for analyzing heterogeneity of individual responses in populations of cells. These approaches and the software tools are modular and can be adapted to a variety of biological problems.

In summary, our statistical clustering to Ca^2+^ responses from ∼300 human islet cells imaged side-by-side was capable of identifying 2 functional β-cell states within this test sample. The approaches applied to this high-quality data set can be used to examine other β-cell states and *in vitro* generated replacement β-cells, or any other cell type.

## Supplementary Material

Supplementary_data.zip
