# Supplementary material for: Statistical approaches and software for clustering islet cell functional heterogeneity
Source: Islets. 2016 Feb 24;8(2):48–56. doi: 10.1080/19382014.2016.1150664 (PMC4878268; doi:10.1080/19382014.2016.1150664)
Supplement: Supplementary_data.zip [file kisl-08-02-1150664-s001.zip › Supplementary data.pdf]

C001 (3 actual peaks, at a rate of 4.34 peaks per 30 min)

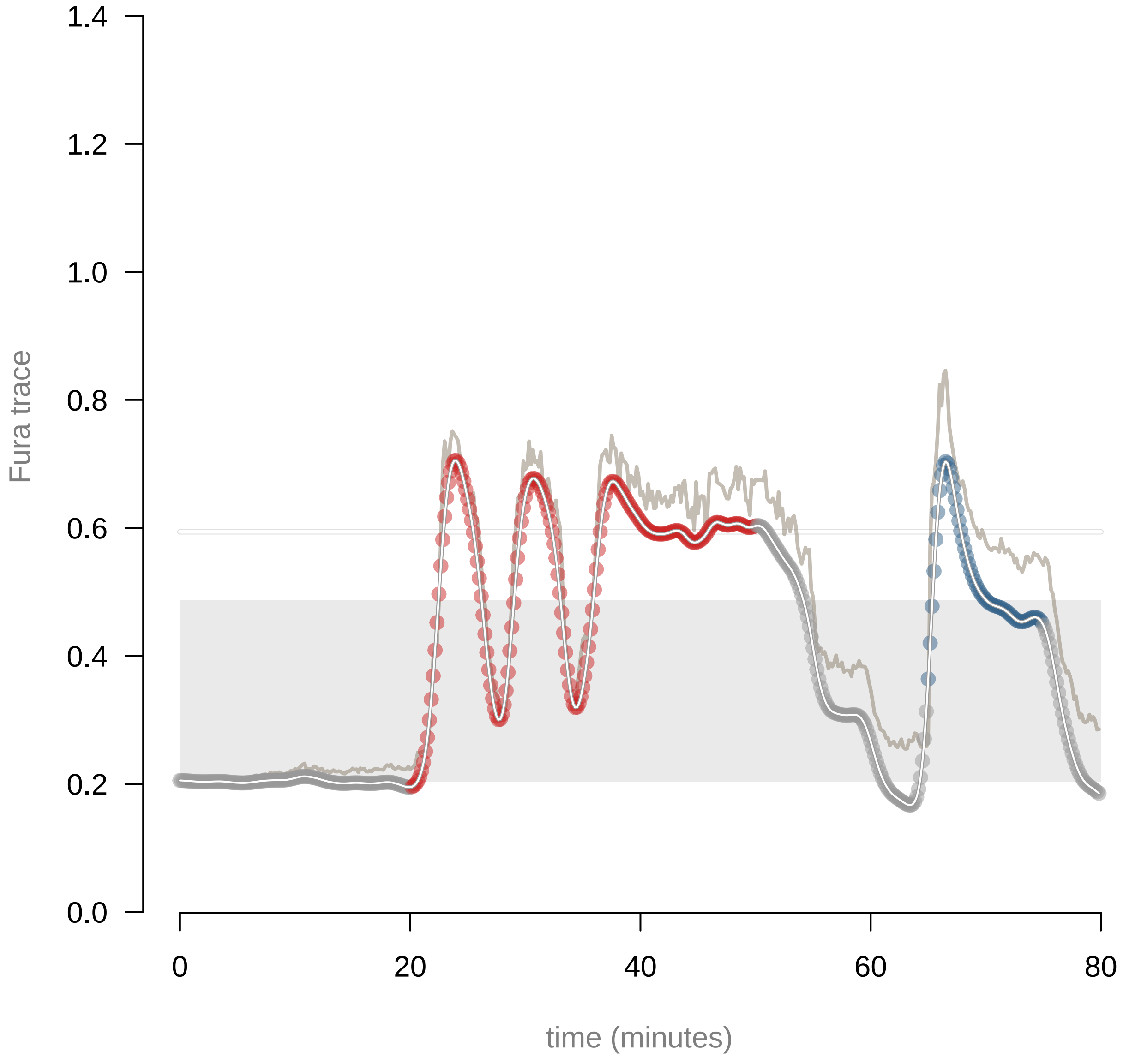

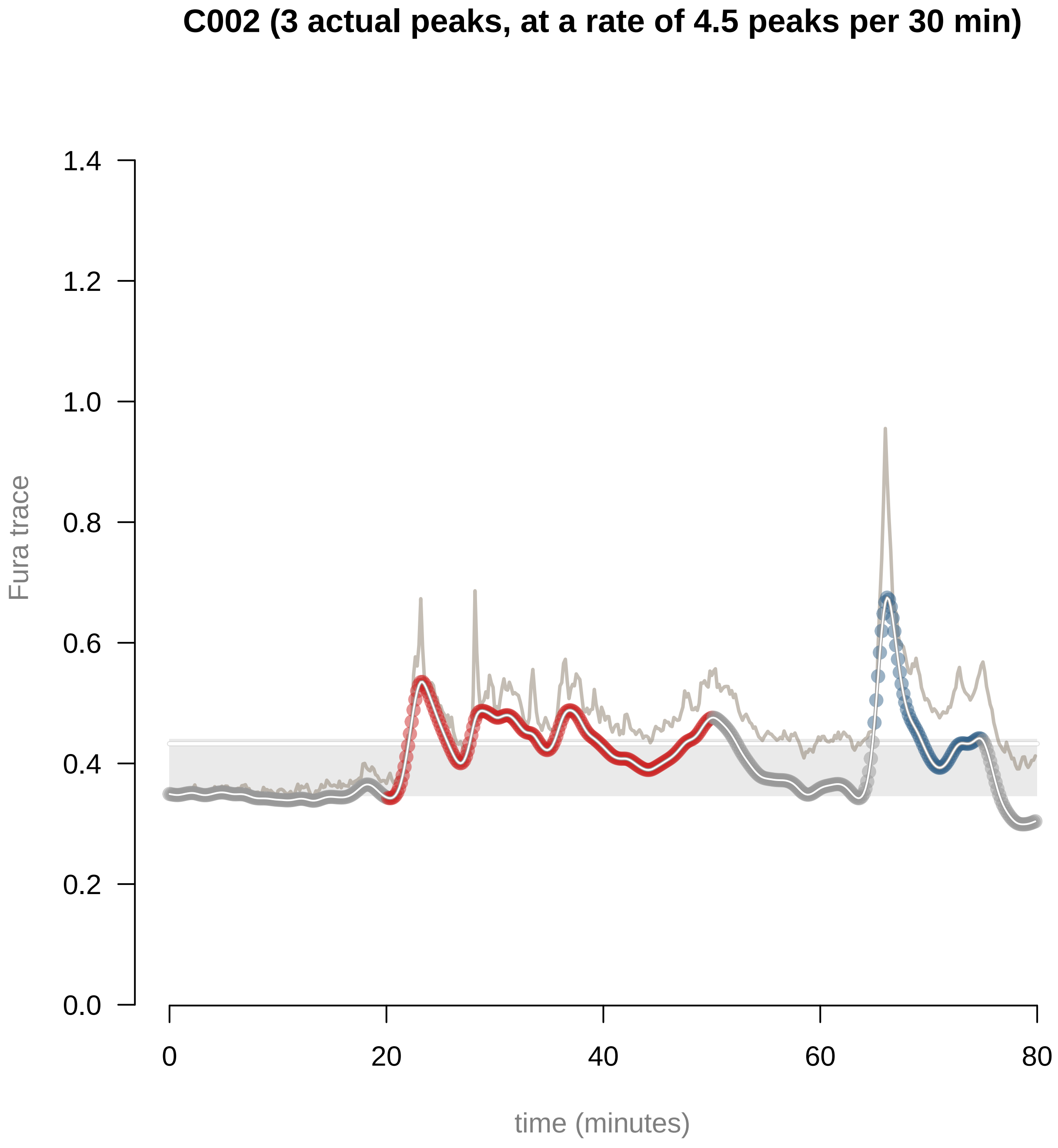

C002 (3 actual peaks, at a rate of 4.5 peaks per 30 min)
Fura trace
1.4
1.2
1.0
0.8
0.6
0.4
0.2
0.0
0
20
40
60
80
time (minutes)

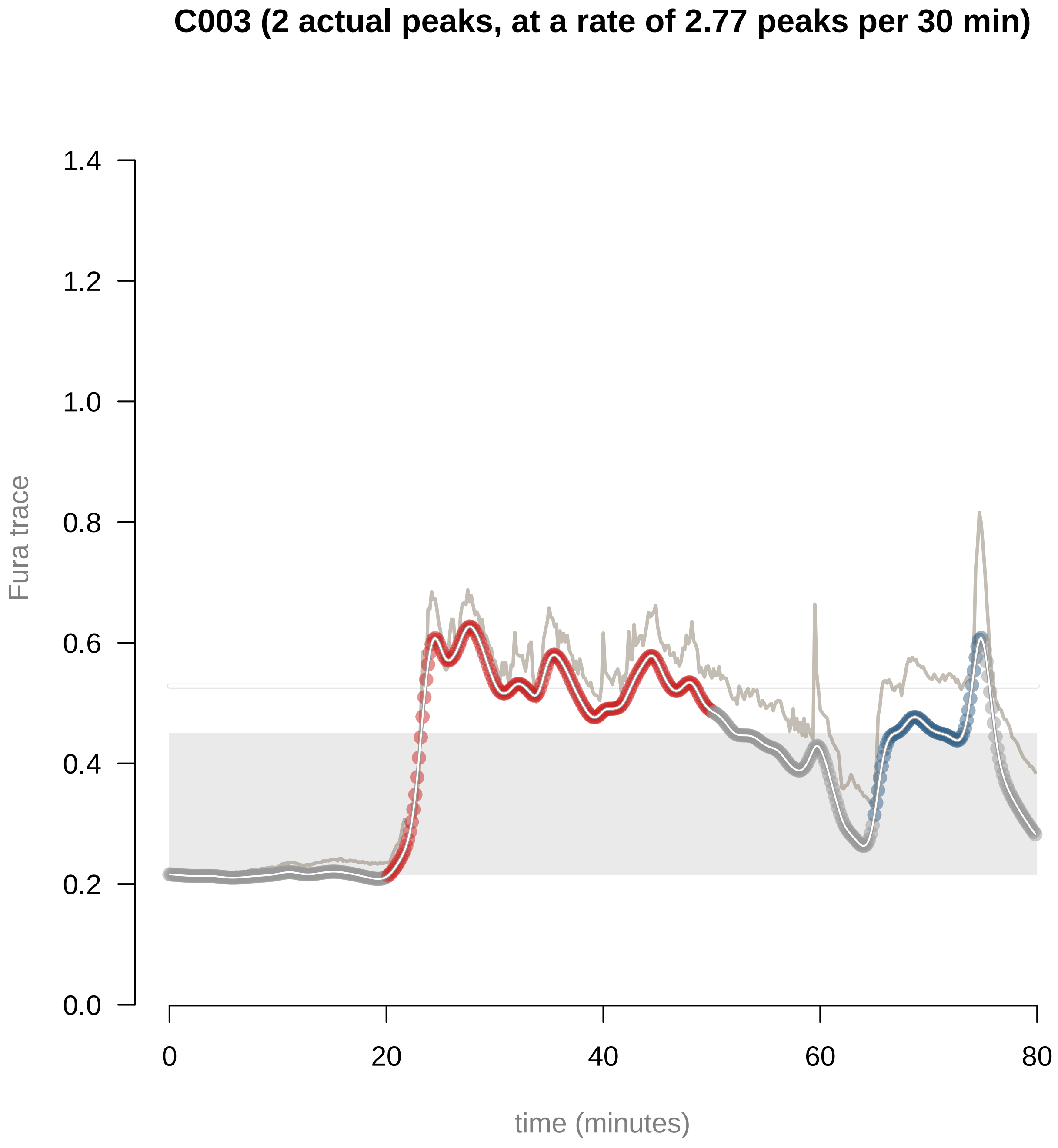

C003 (2 actual peaks, at a rate of 2.77 peaks per 30 min)
Fura trace
1.4
1.2
1.0
0.8
0.6
0.4
0.2
0.0
0
20
40
60
80
time (minutes)

C004 (1 actual peaks, at a rate of 1 peaks per 30 min)

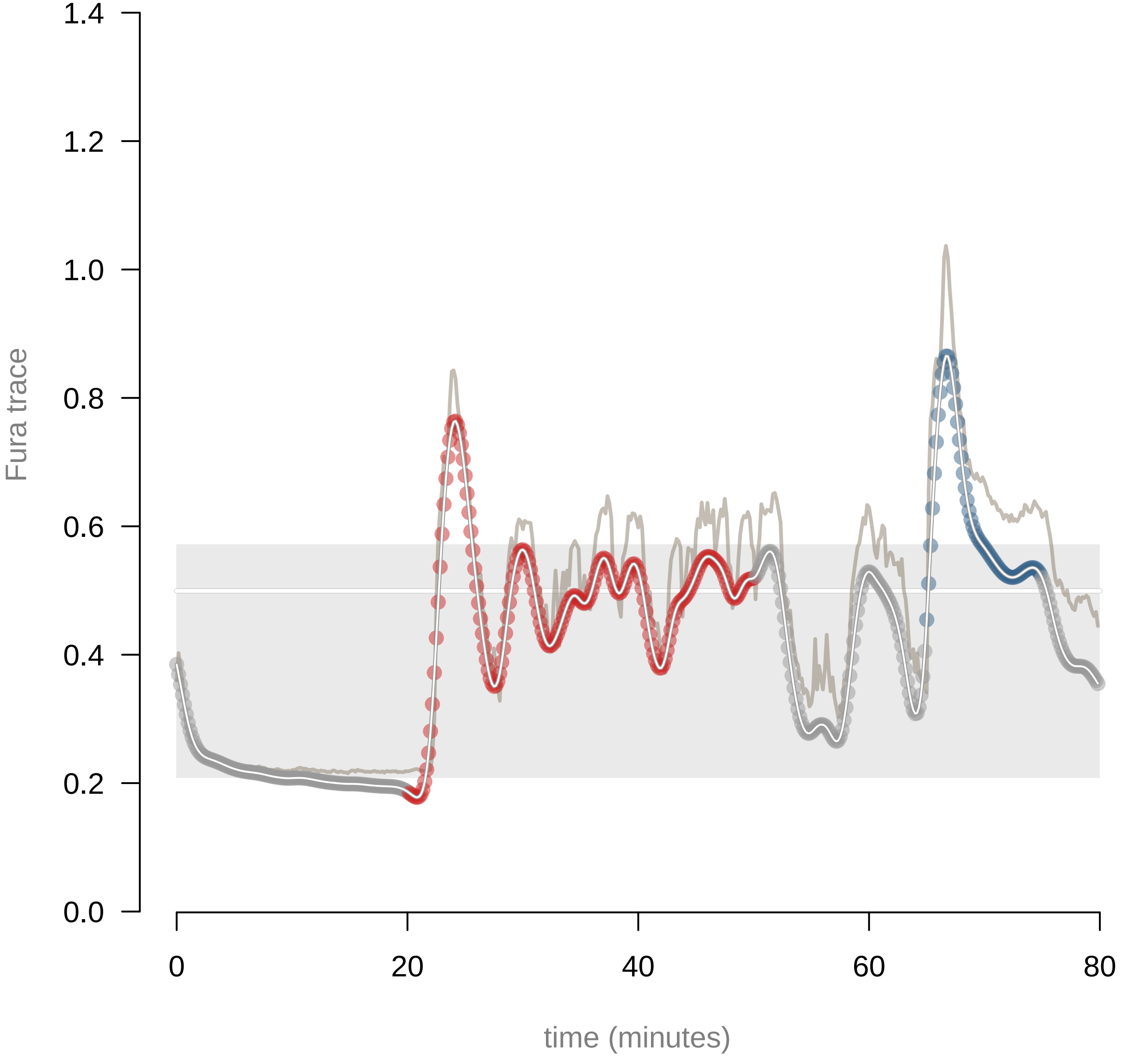

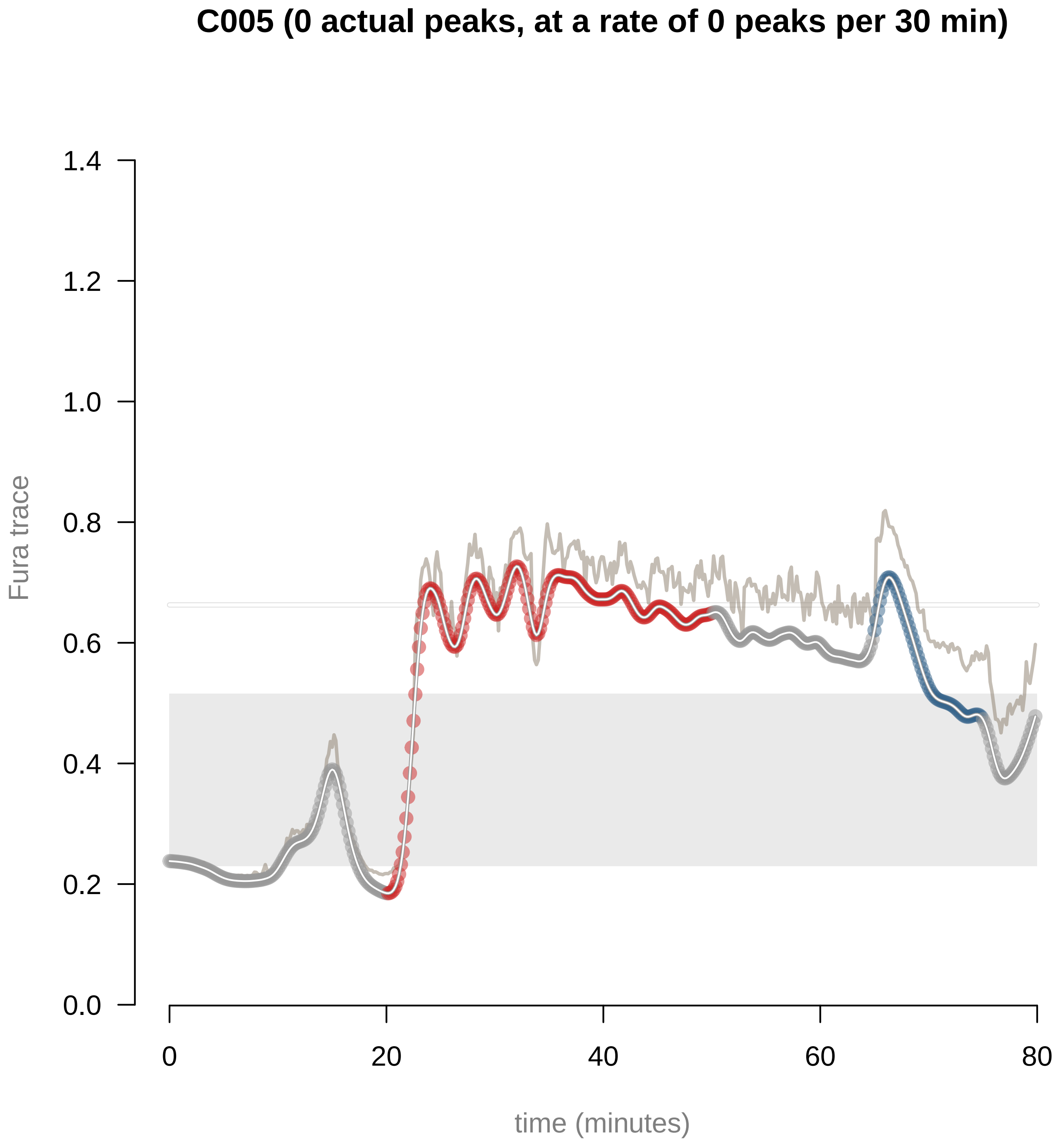
C005 (0 actual peaks, at a rate of 0 peaks per 30 min)
Fura trace
time (minutes)
0.0
0.2
0.4
0.6
0.8
1.0
1.2
1.4
0
20
40
60
80

C006 (1 actual peaks, at a rate of 1 peaks per 30 min)

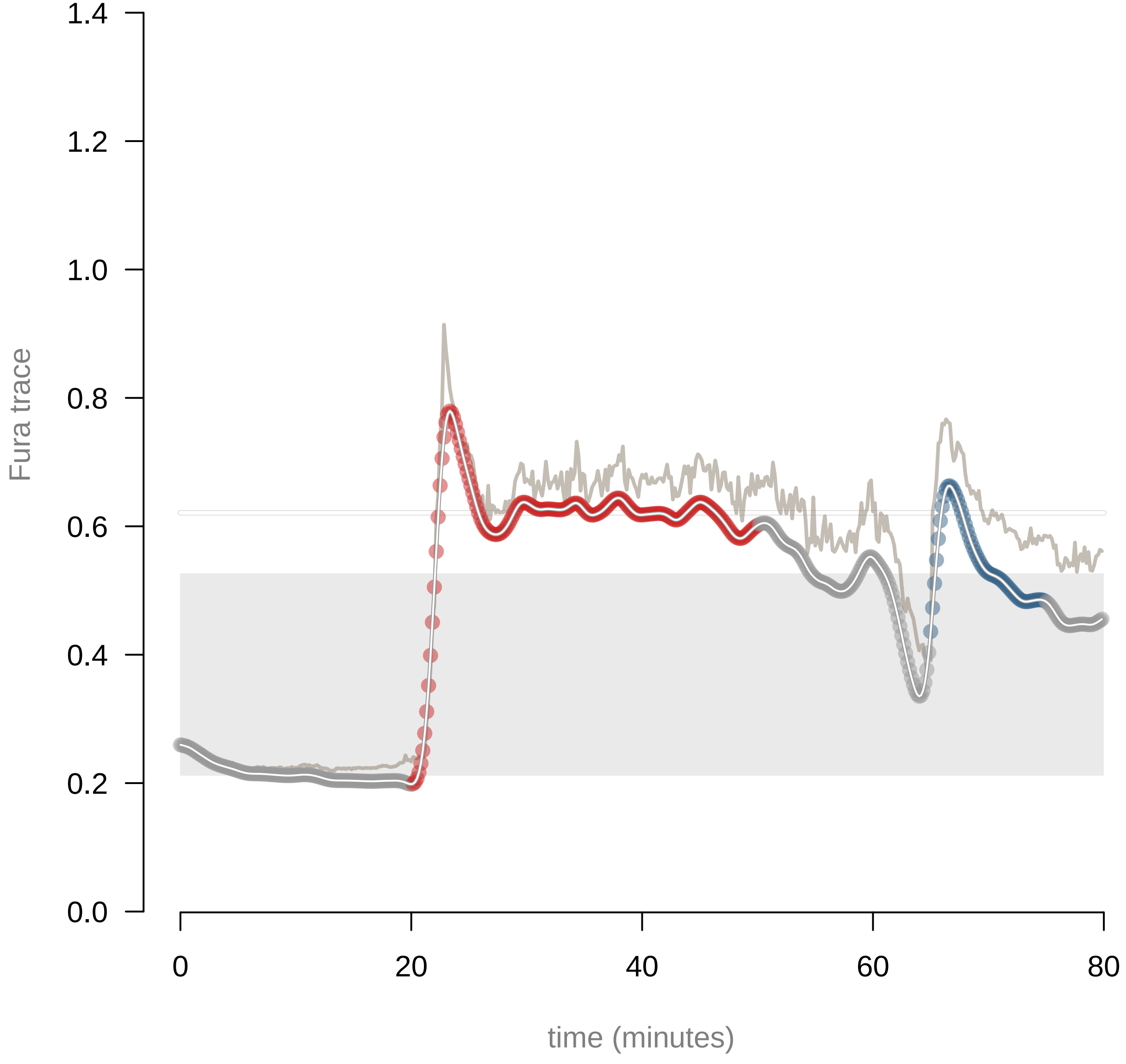

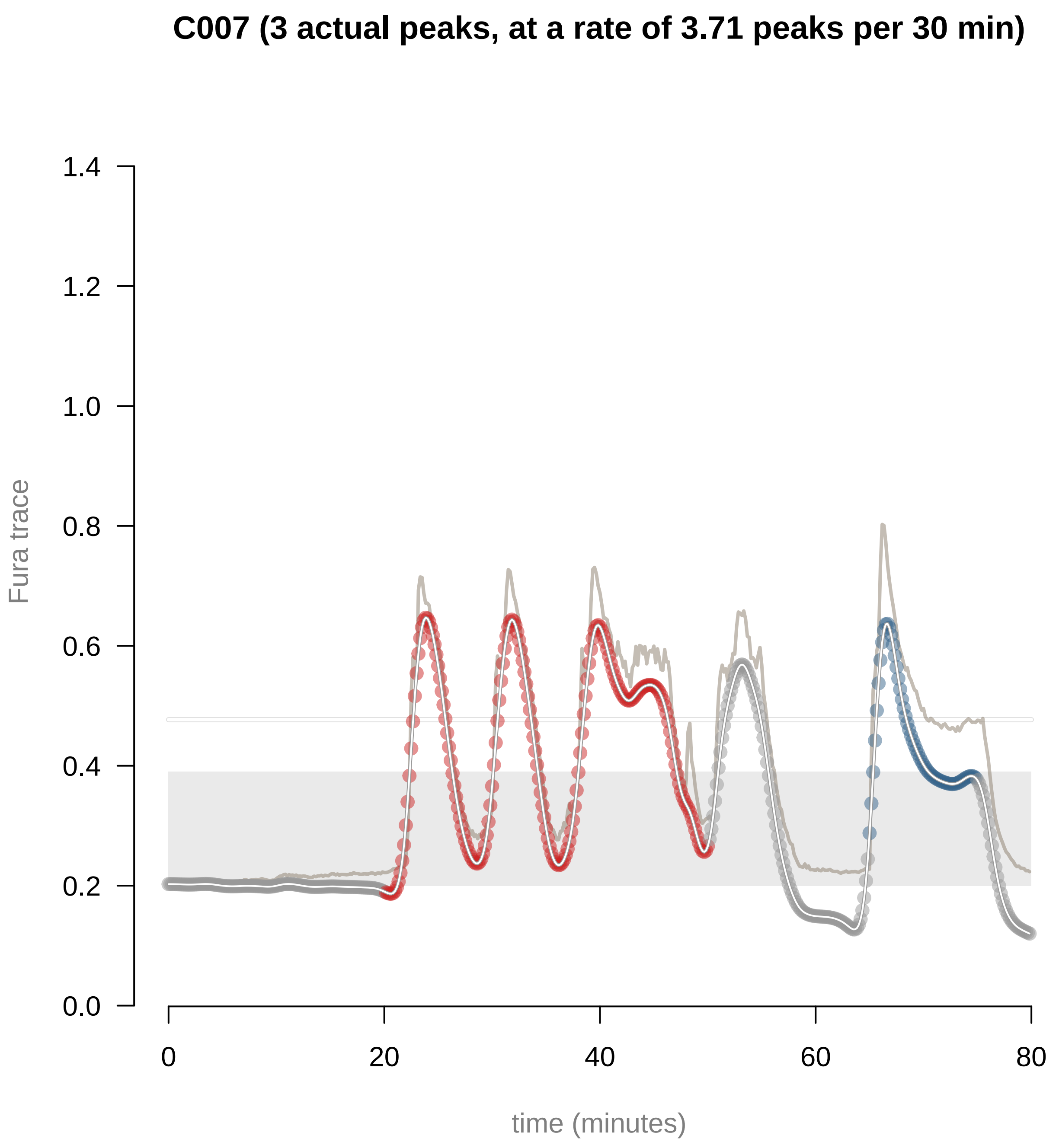
C007 (3 actual peaks, at a rate of 3.71 peaks per 30 min)
Fura trace
1.4
1.2
1.0
0.8
0.6
0.4
0.2
0.0
0
20
40
60
80
time (minutes)

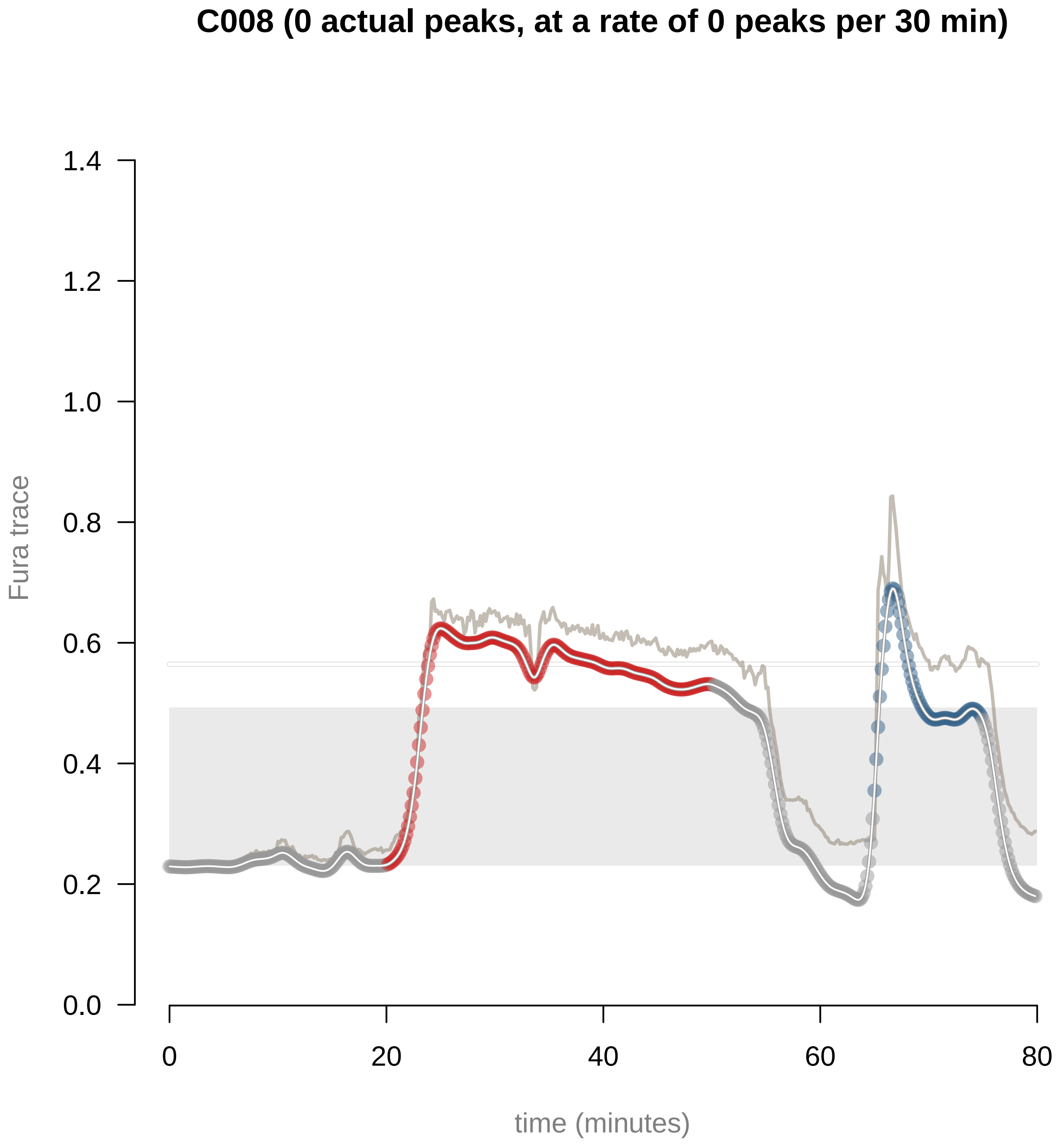

C008 (0 actual peaks, at a rate of 0 peaks per 30 min)
Fura trace
0.0
0.2
0.4
0.6
0.8
1.0
1.2
1.4
0
20
40
60
80
time (minutes)

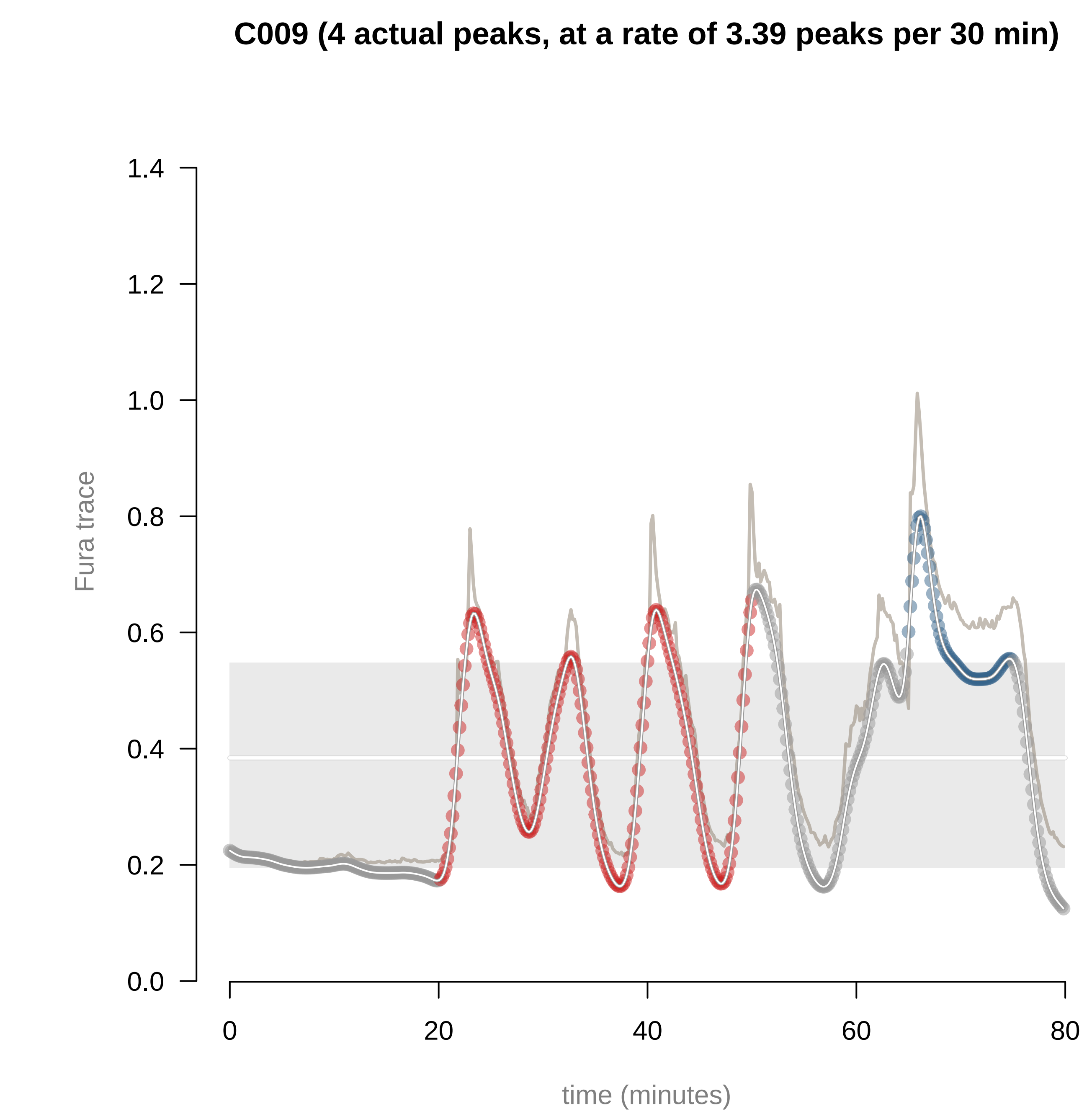

C009 (4 actual peaks, at a rate of 3.39 peaks per 30 min)
Fura trace
1.4
1.2
1.0
0.8
0.6
0.4
0.2
0.0
0
20
40
60
80
time (minutes)

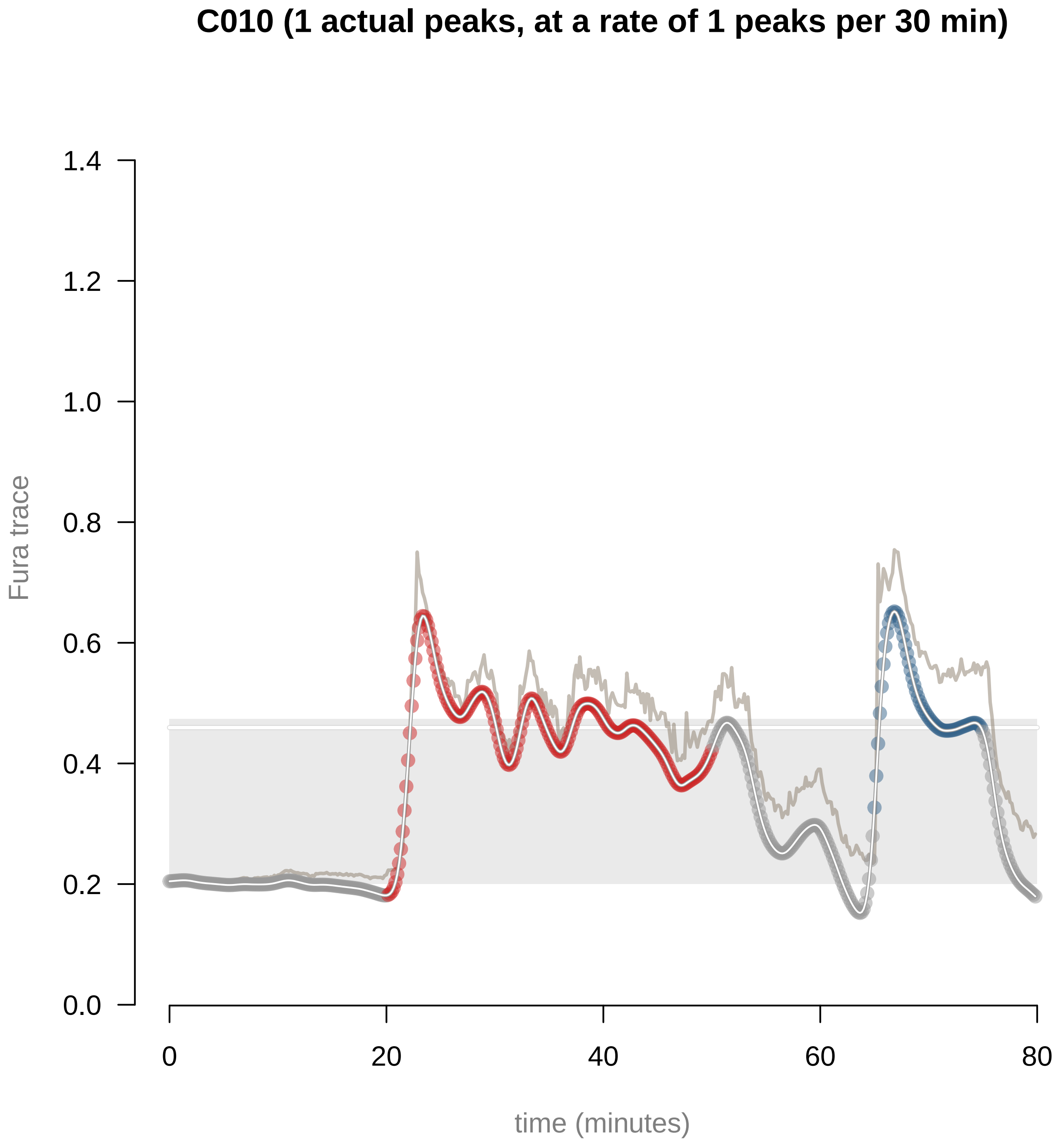
C010 (1 actual peaks, at a rate of 1 peaks per 30 min)
Fura trace
time (minutes)
0.0
0.2
0.4
0.6
0.8
1.0
1.2
1.4
0
20
40
60
80

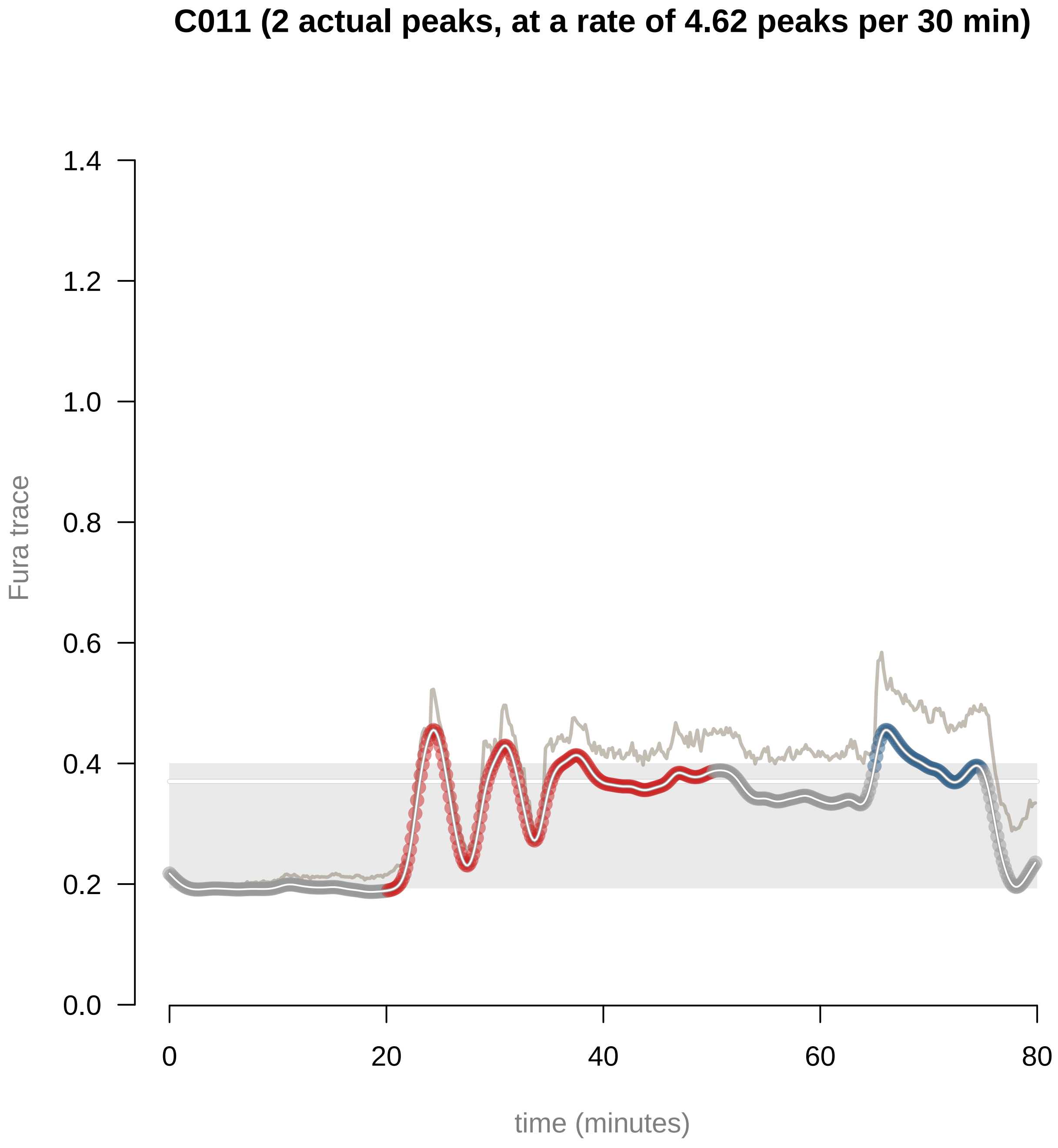

C011 (2 actual peaks, at a rate of 4.62 peaks per 30 min)
Fura trace
time (minutes)
0.0
0.2
0.4
0.6
0.8
1.0
1.2
1.4
0
20
40
60
80

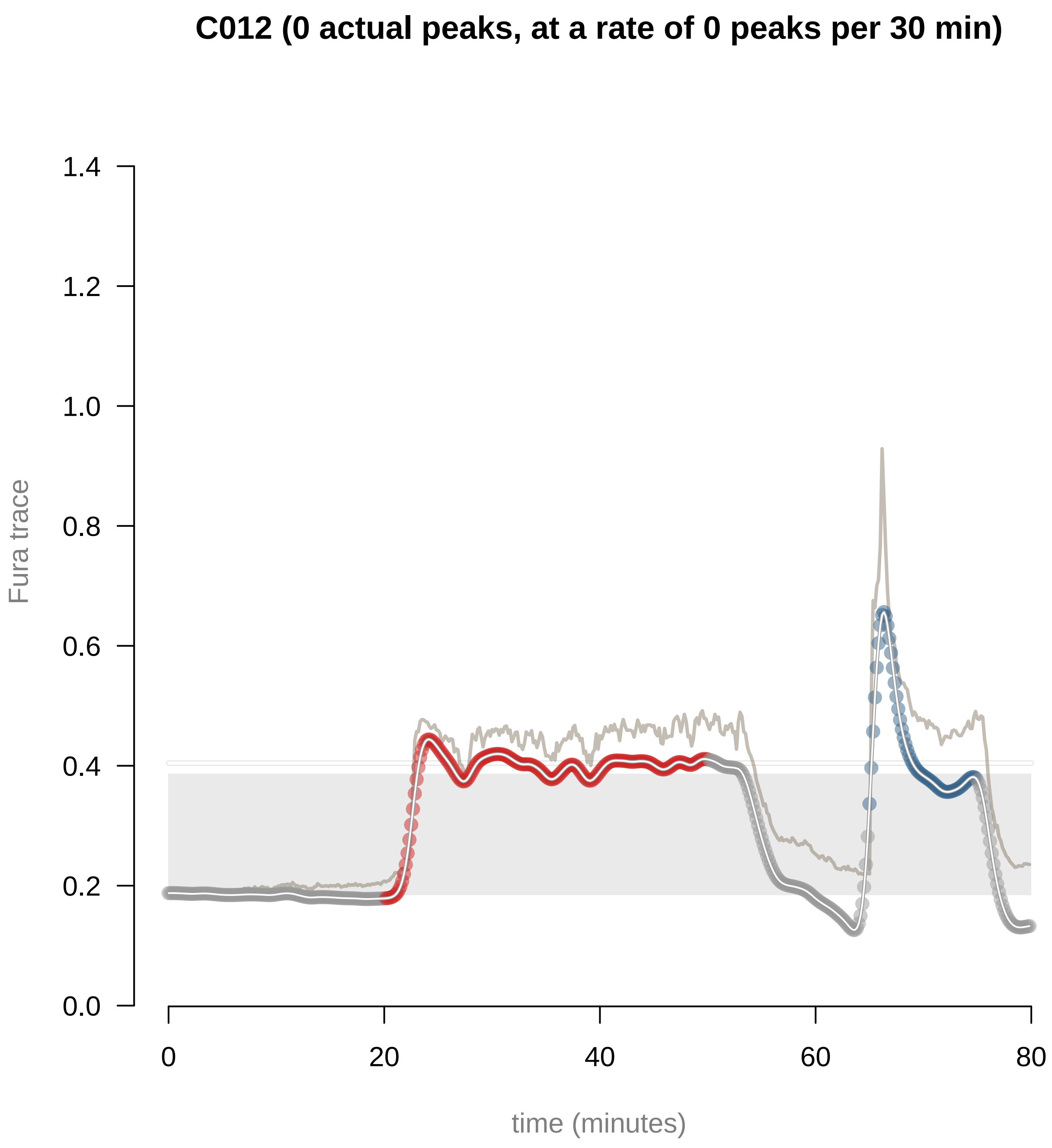

C012 (0 actual peaks, at a rate of 0 peaks per 30 min)
Fura trace
0.0
0.2
0.4
0.6
0.8
1.0
1.2
1.4
0
20
40
60
80
time (minutes)

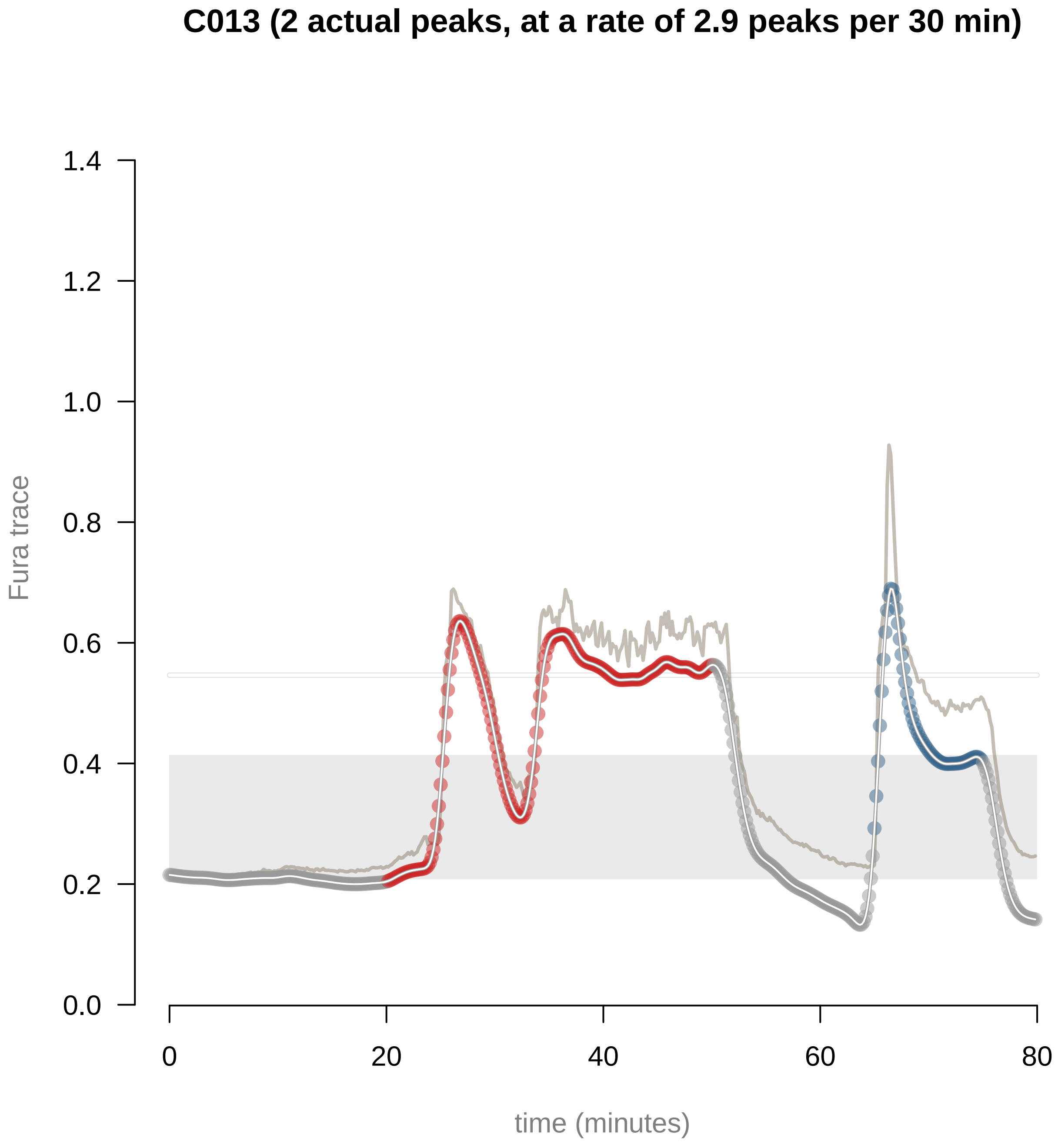

C013 (2 actual peaks, at a rate of 2.9 peaks per 30 min)
Fura trace
0.0
0.2
0.4
0.6
0.8
1.0
1.2
1.4
0
20
40
60
80
time (minutes)

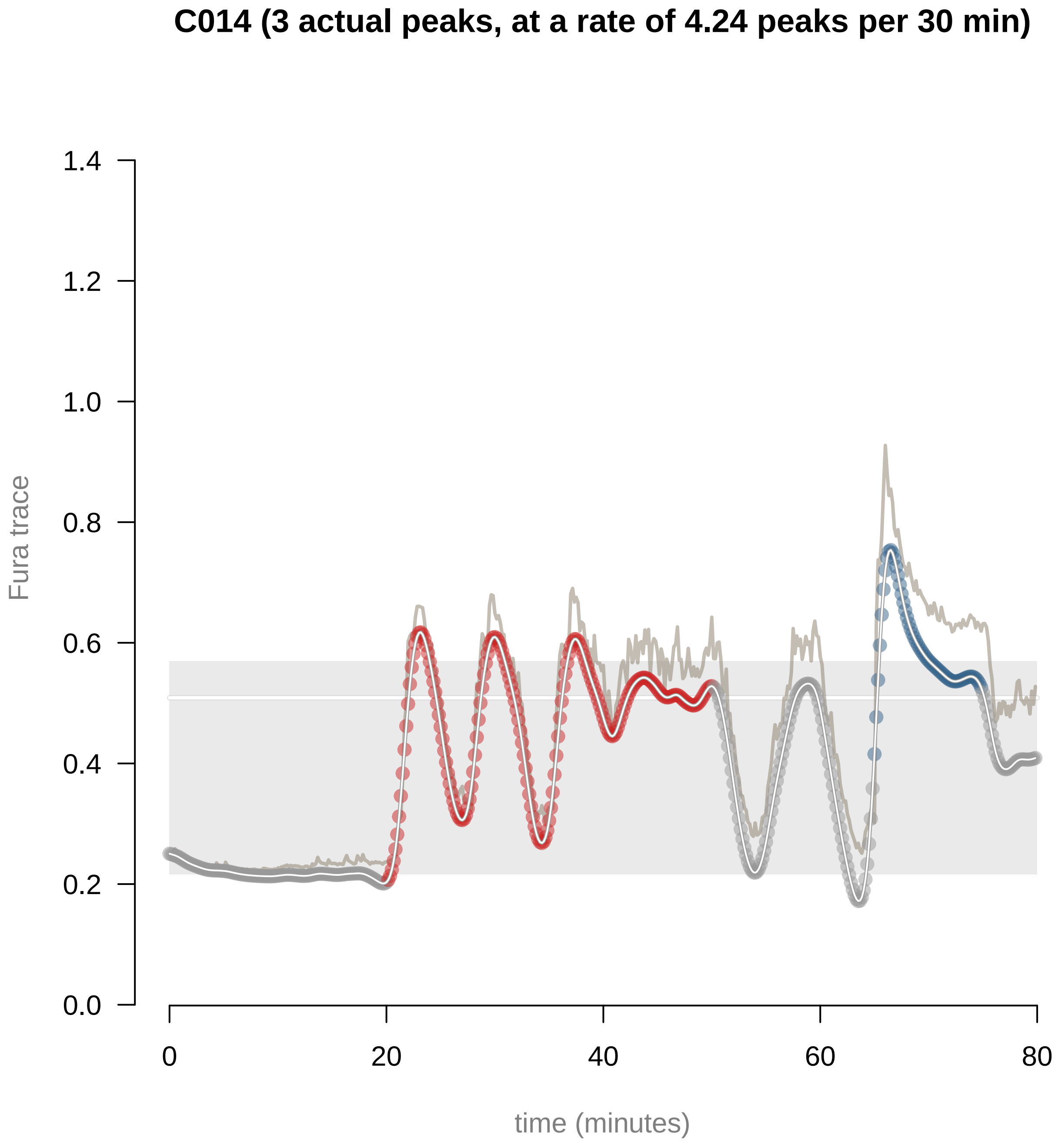
C014 (3 actual peaks, at a rate of 4.24 peaks per 30 min)
Fura trace
1.4
1.2
1.0
0.8
0.6
0.4
0.2
0.0
0
20
40
60
80
time (minutes)

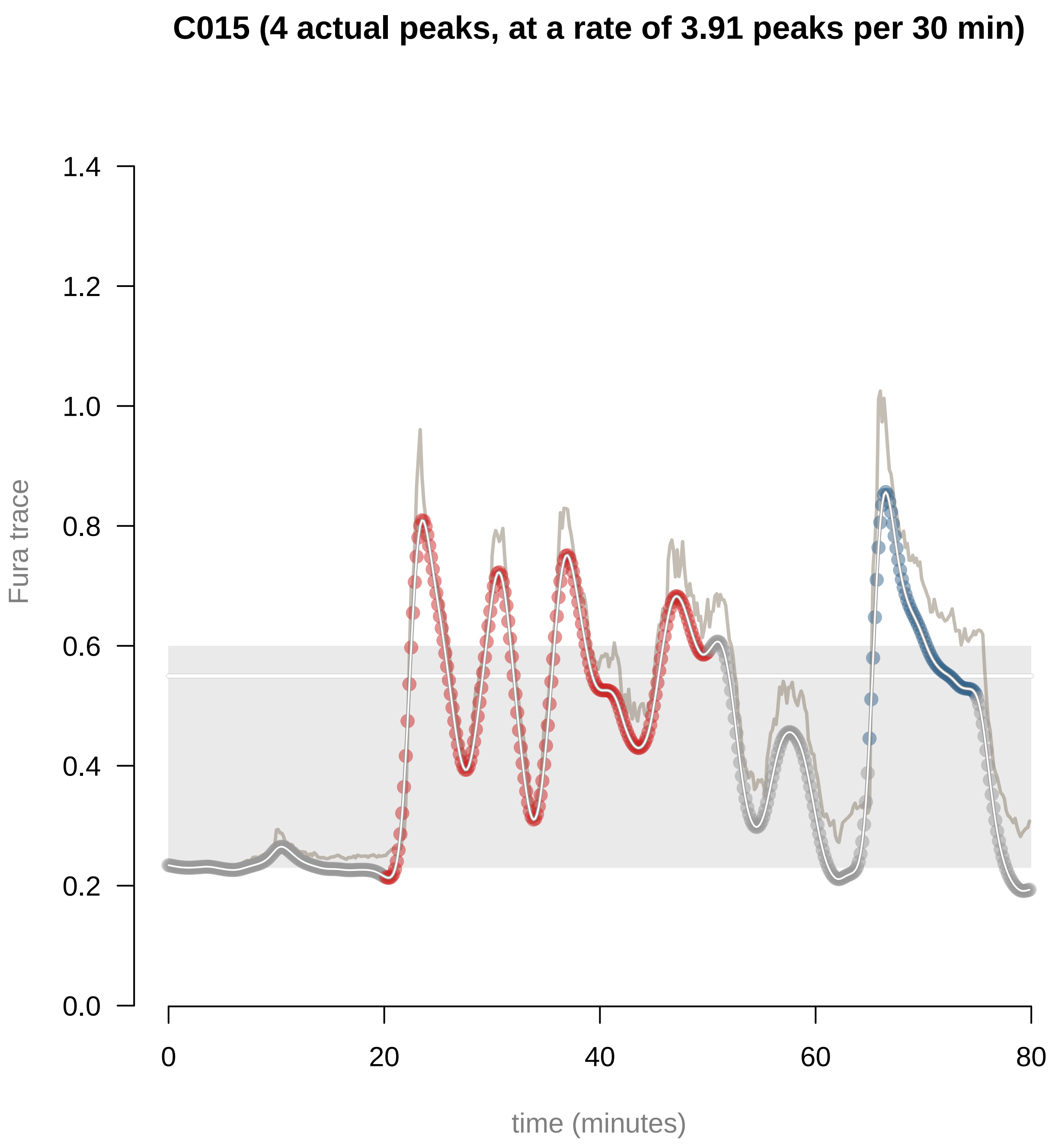
C015 (4 actual peaks, at a rate of 3.91 peaks per 30 min)
Fura trace
1.4
1.2
1.0
0.8
0.6
0.4
0.2
0.0
0
20
40
60
80
time (minutes)

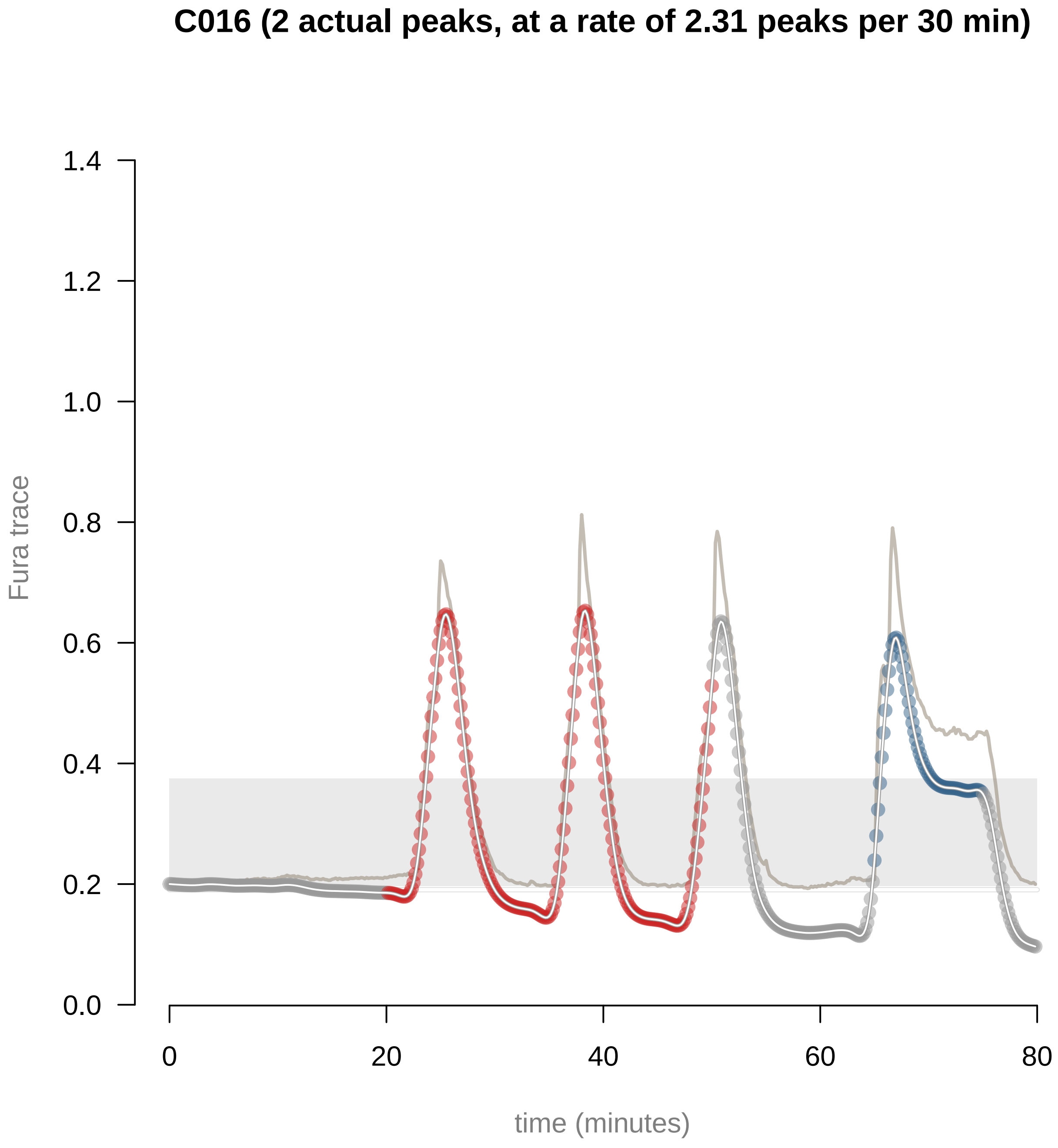
C016 (2 actual peaks, at a rate of 2.31 peaks per 30 min)
1.4
1.2
1.0
0.8
0.6
0.4
0.2
0.0
Fura trace
0
20
40
60
80
time (minutes)

C017 (2 actual peaks, at a rate of 1.57 peaks per 30 min)

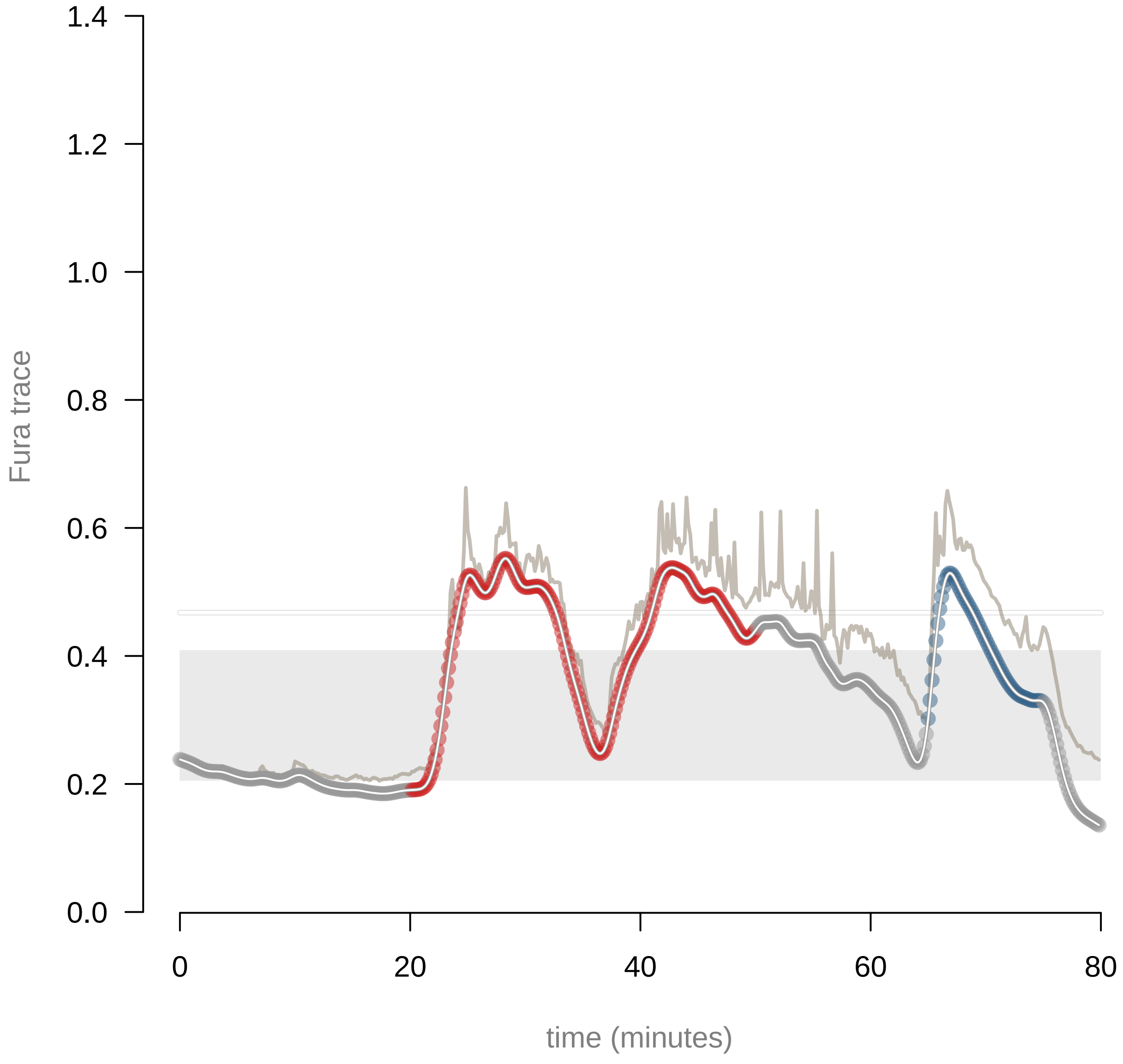

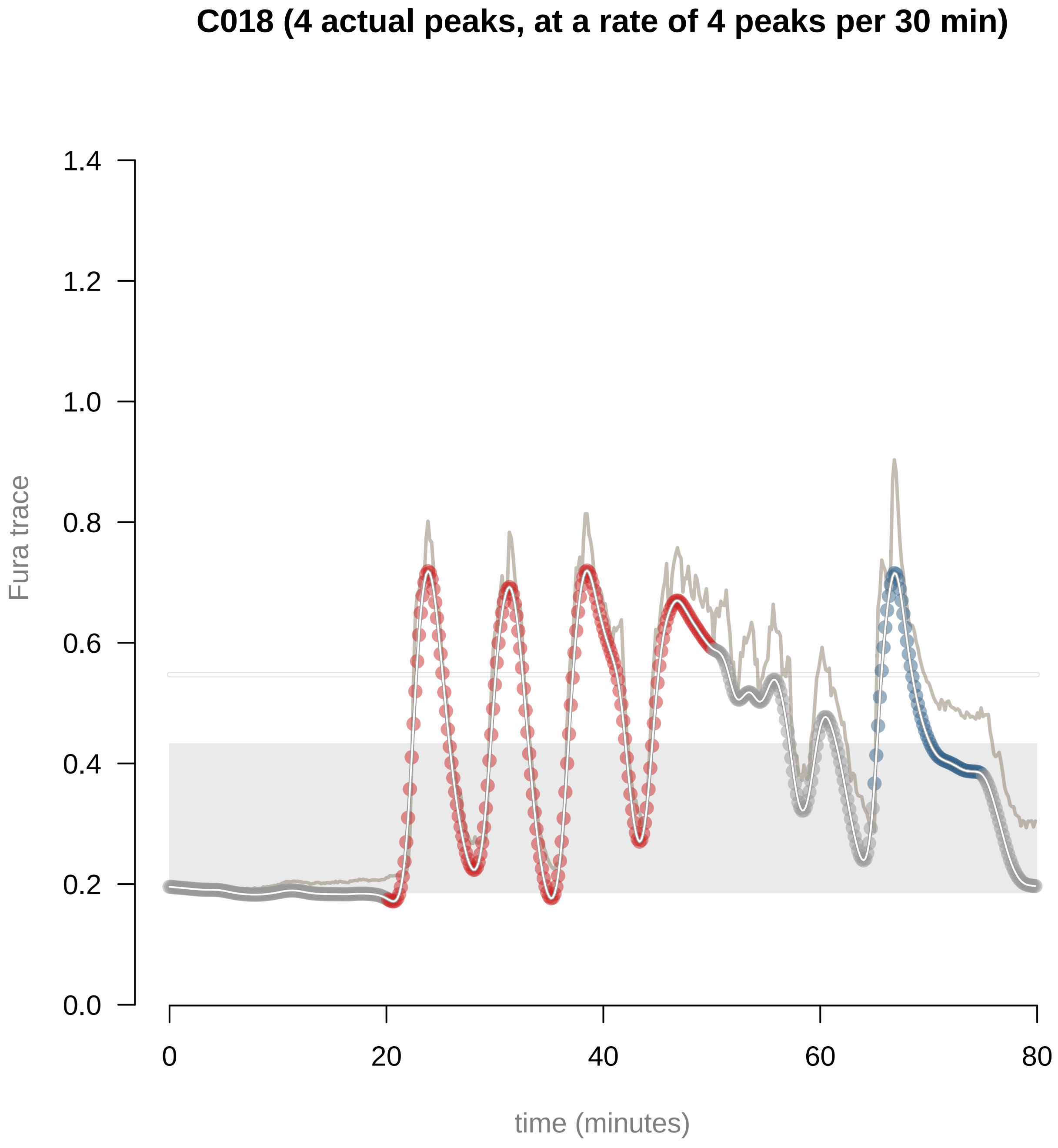

C018 (4 actual peaks, at a rate of 4 peaks per 30 min)
1.4
1.2
1.0
0.8
0.6
0.4
0.2
0.0
Fura trace
0
20
40
60
80
time (minutes)

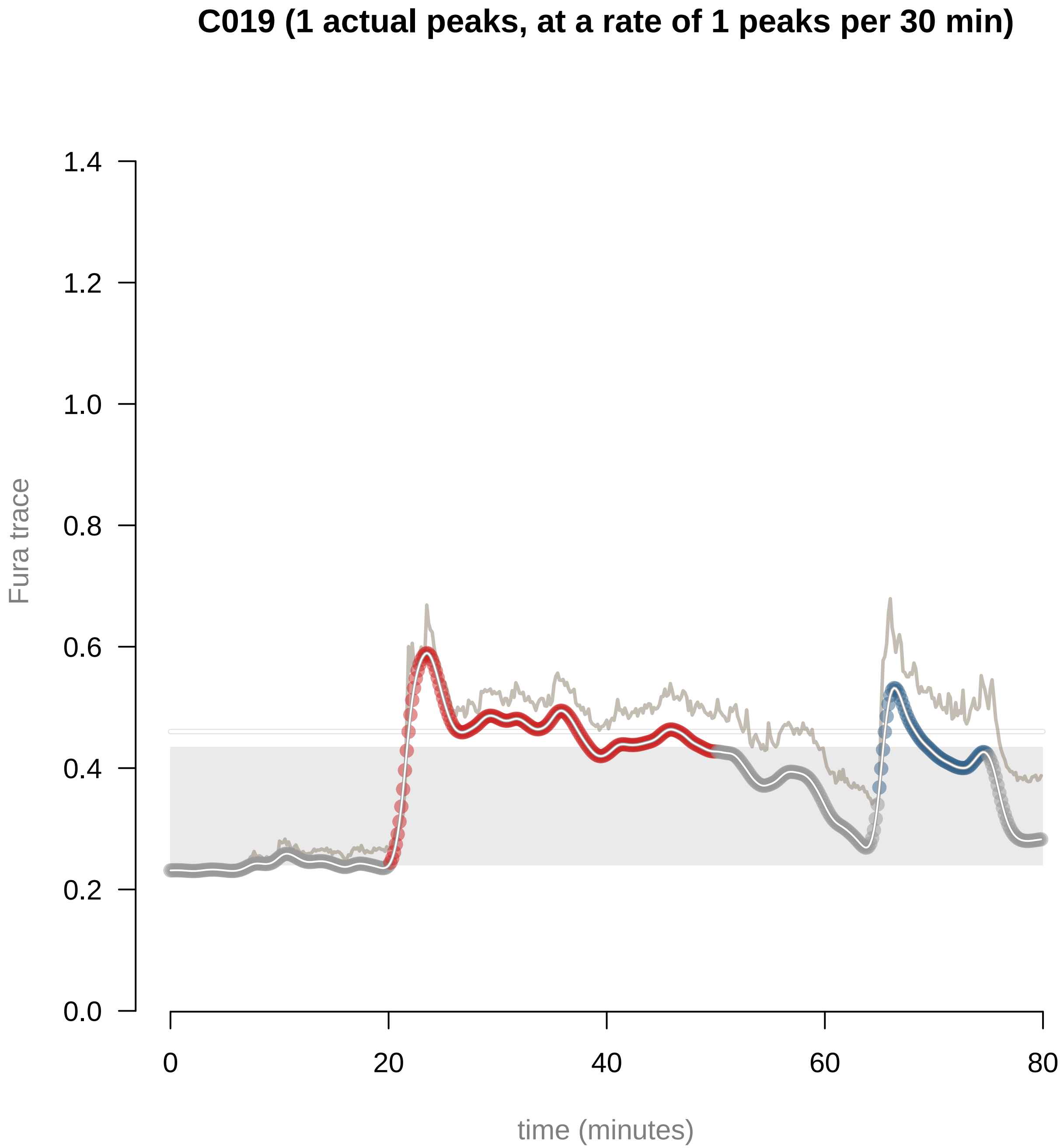

C019 (1 actual peaks, at a rate of 1 peaks per 30 min)
Fura trace
time (minutes)
0.0
0.2
0.4
0.6
0.8
1.0
1.2
1.4
0
20
40
60
80

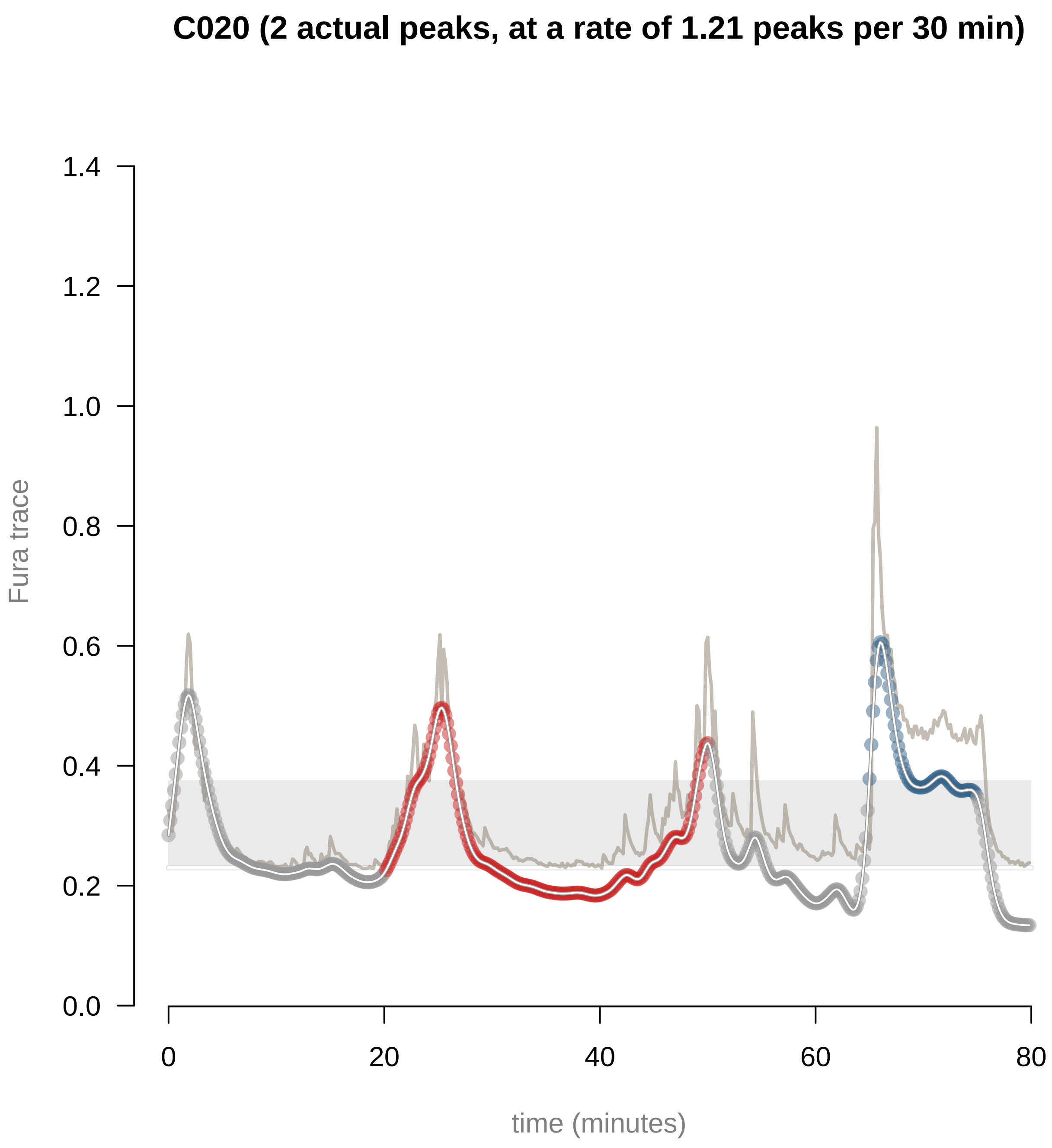
C020 (2 actual peaks, at a rate of 1.21 peaks per 30 min)
Fura trace
1.4
1.2
1.0
0.8
0.6
0.4
0.2
0.0
0
20
40
60
80
time (minutes)

C021 (1 actual peaks, at a rate of 1 peaks per 30 min)

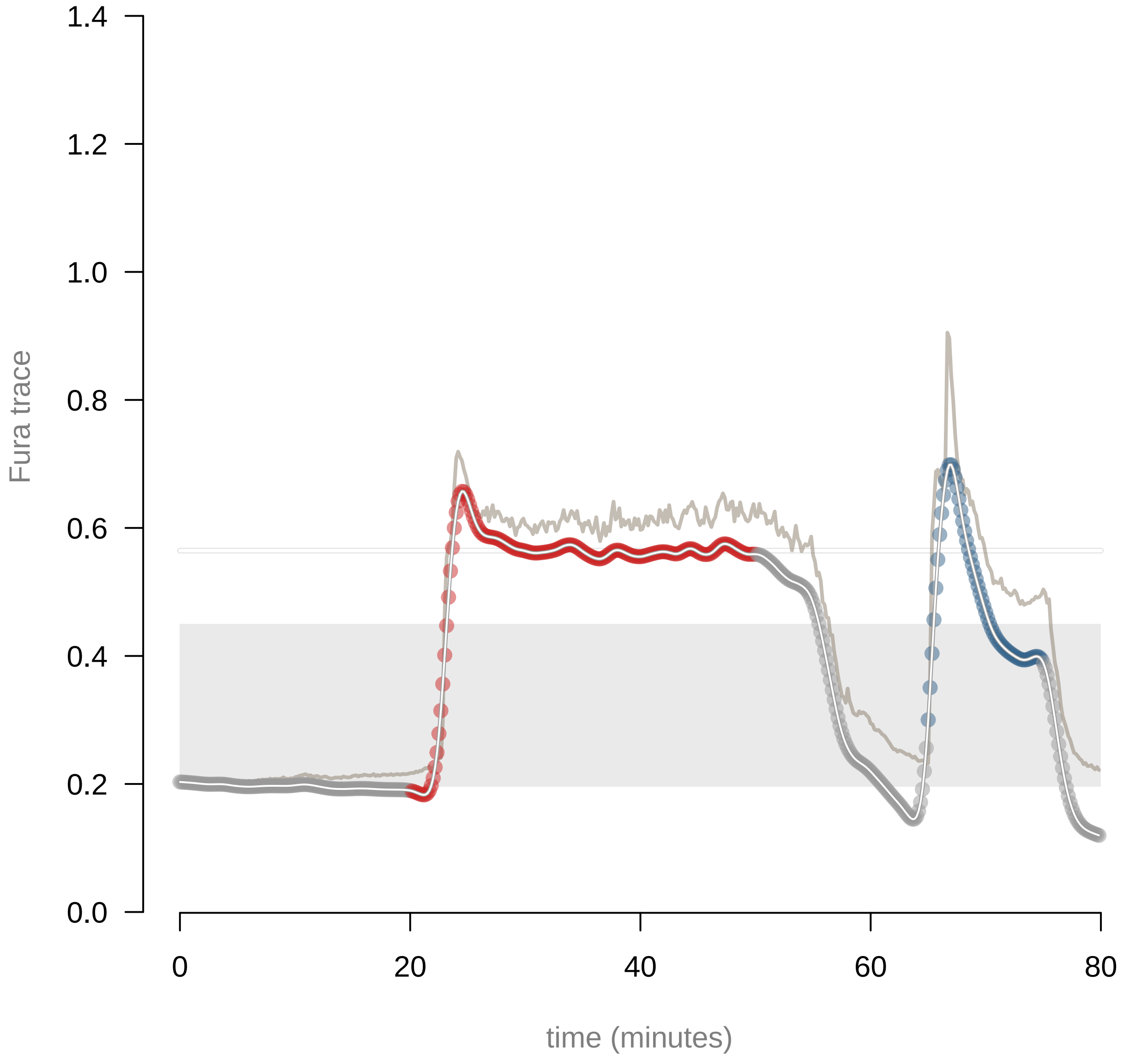

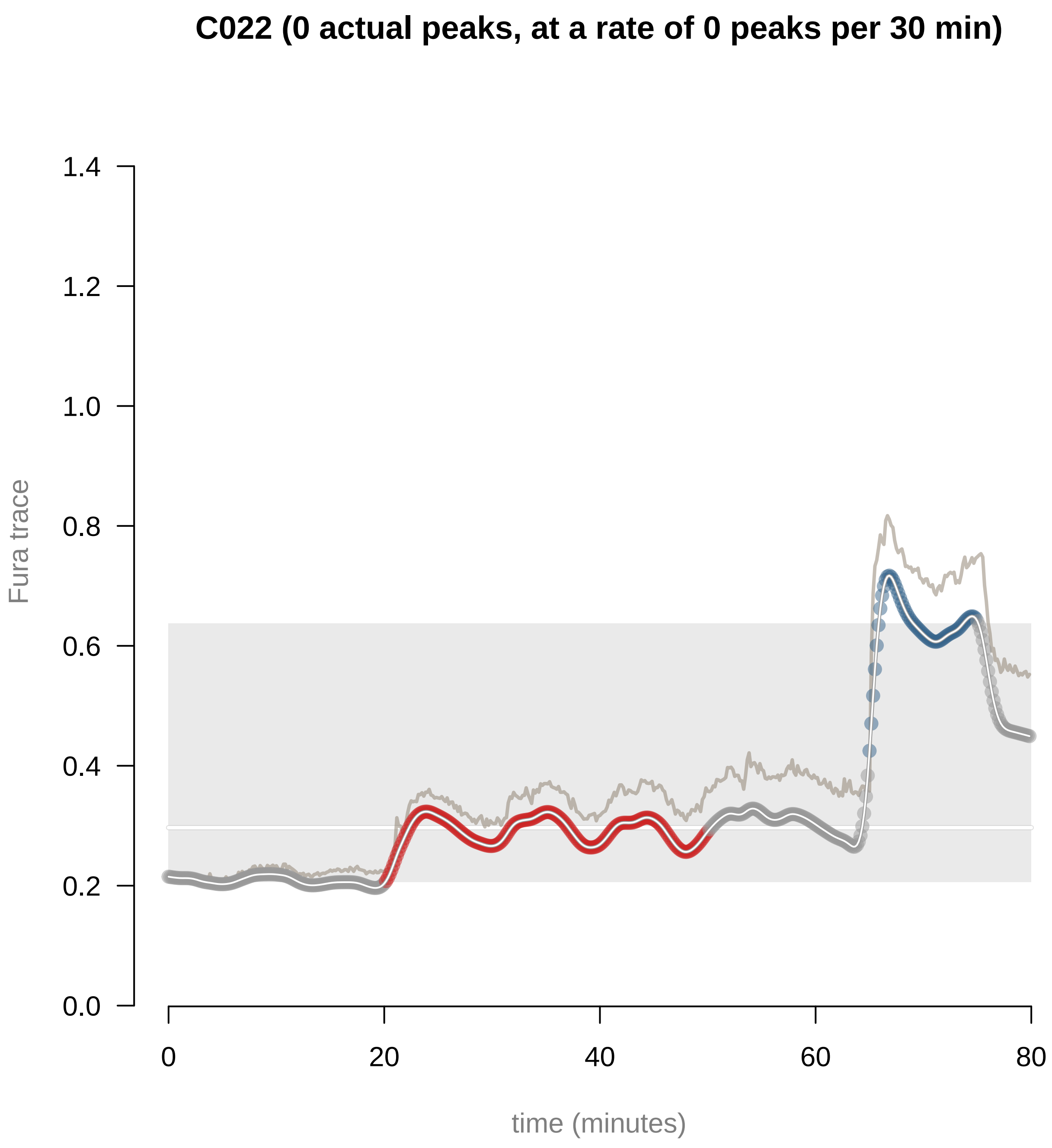
C022 (0 actual peaks, at a rate of 0 peaks per 30 min)
Fura trace
1.4
1.2
1.0
0.8
0.6
0.4
0.2
0.0
0
20
40
60
80
time (minutes)

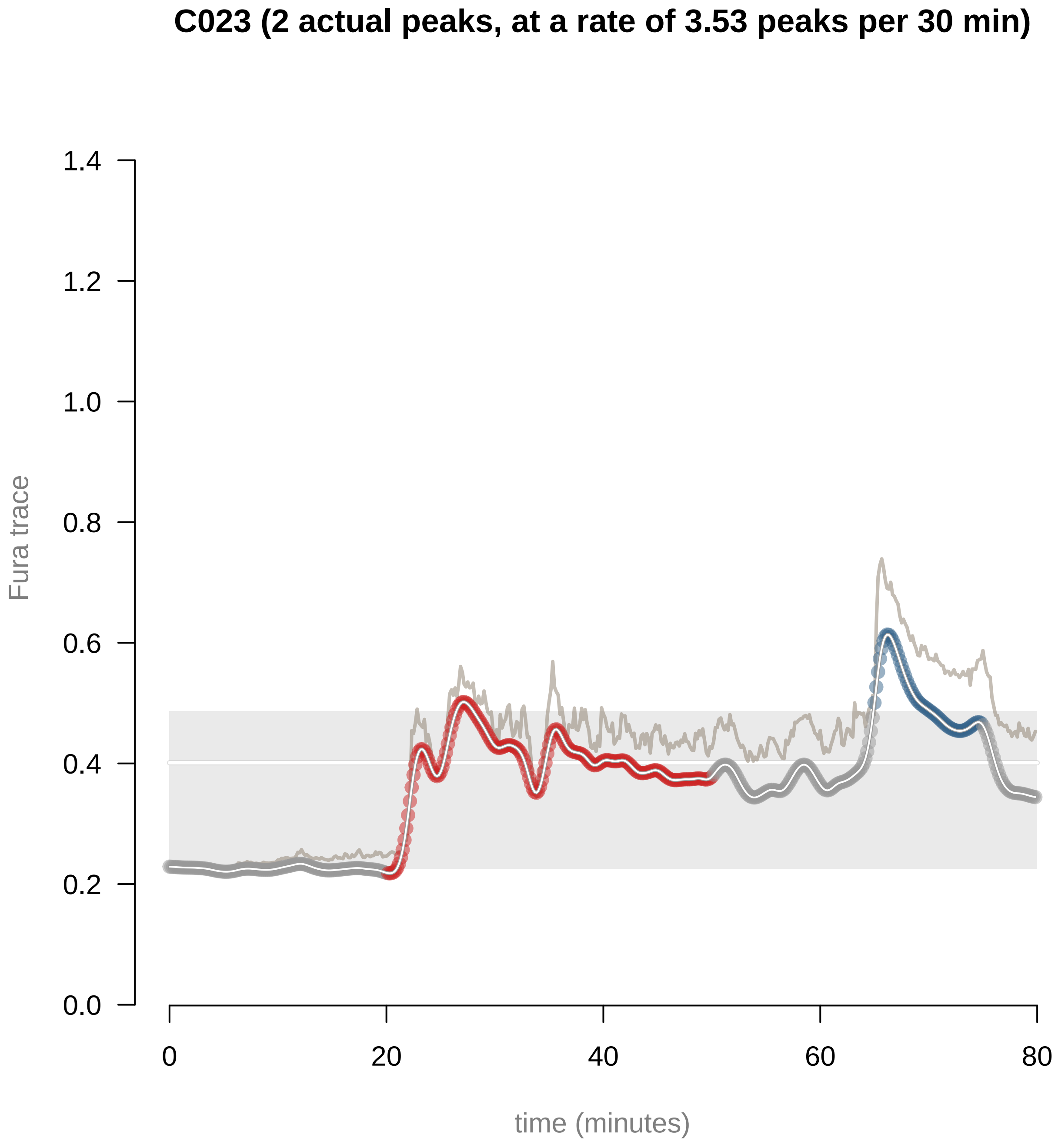

C023 (2 actual peaks, at a rate of 3.53 peaks per 30 min)
Fura trace
1.4
1.2
1.0
0.8
0.6
0.4
0.2
0.0
0
20
40
60
80
time (minutes)

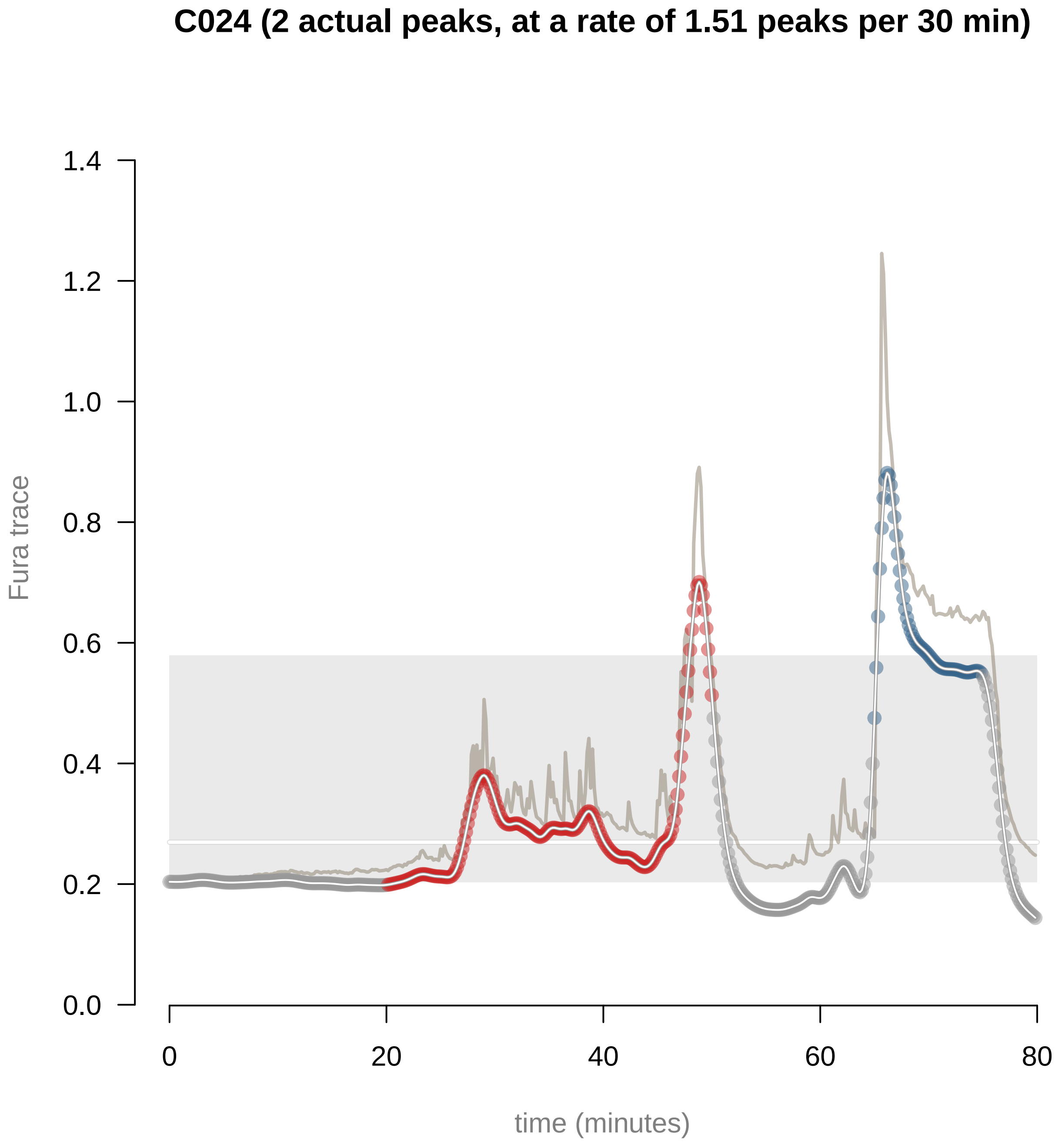

C024 (2 actual peaks, at a rate of 1.51 peaks per 30 min)
Fura trace
1.4
1.2
1.0
0.8
0.6
0.4
0.2
0.0
0
20
40
60
80
time (minutes)

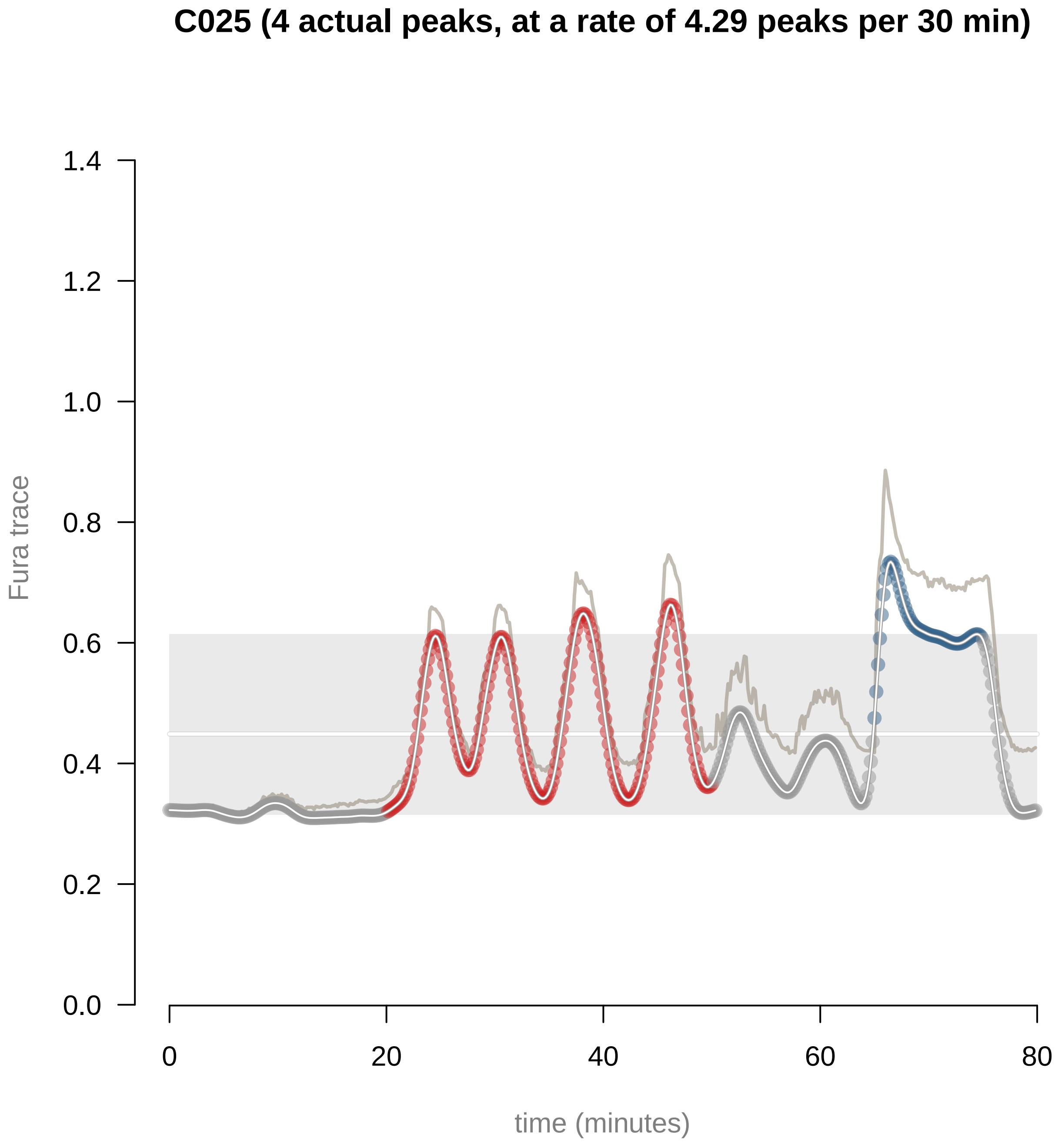
C025 (4 actual peaks, at a rate of 4.29 peaks per 30 min)
Fura trace
1.4
1.2
1.0
0.8
0.6
0.4
0.2
0.0
0
20
40
60
80
time (minutes)

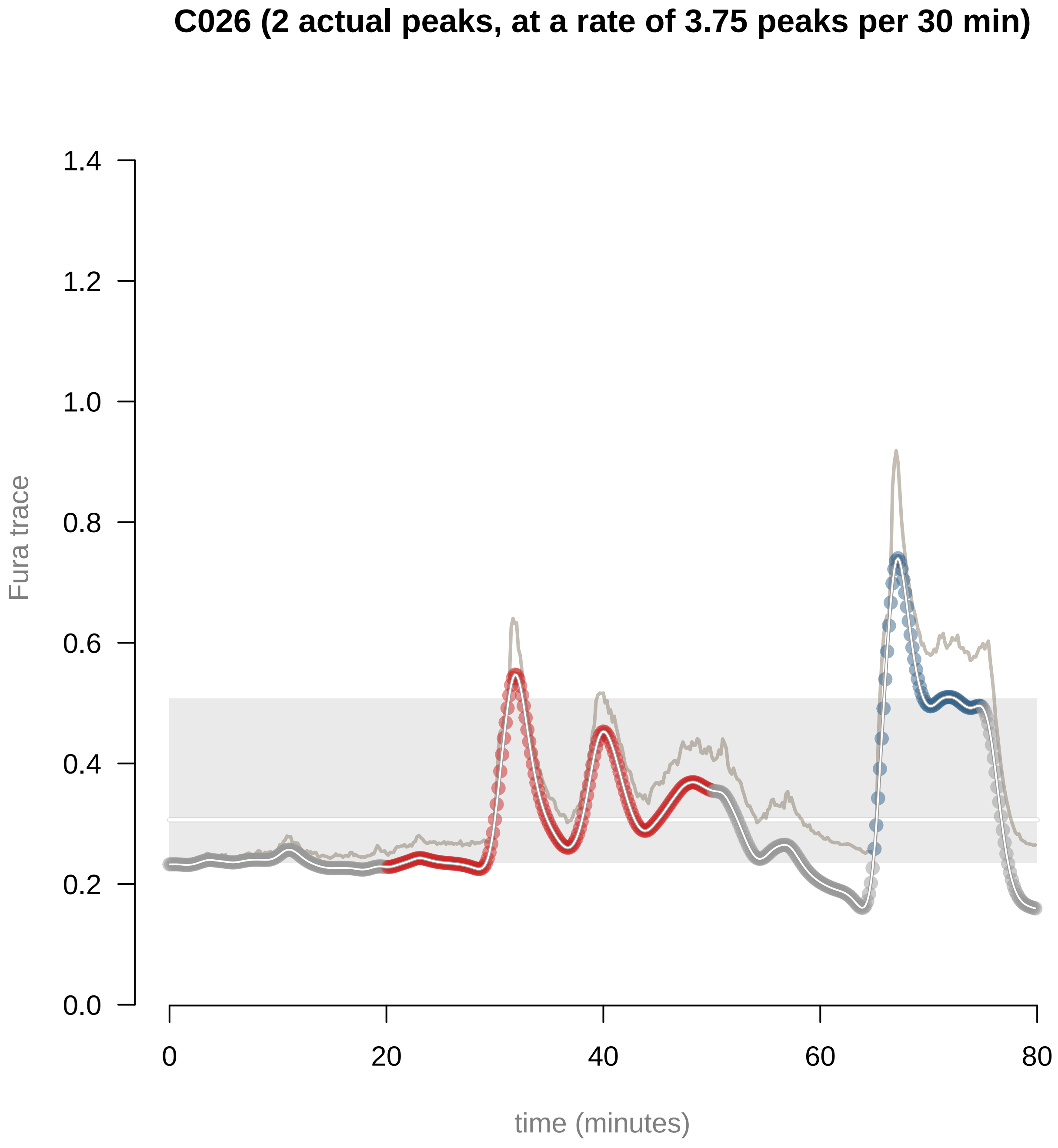
C026 (2 actual peaks, at a rate of 3.75 peaks per 30 min)
Fura trace
1.4
1.2
1.0
0.8
0.6
0.4
0.2
0.0
0
20
40
60
80
time (minutes)

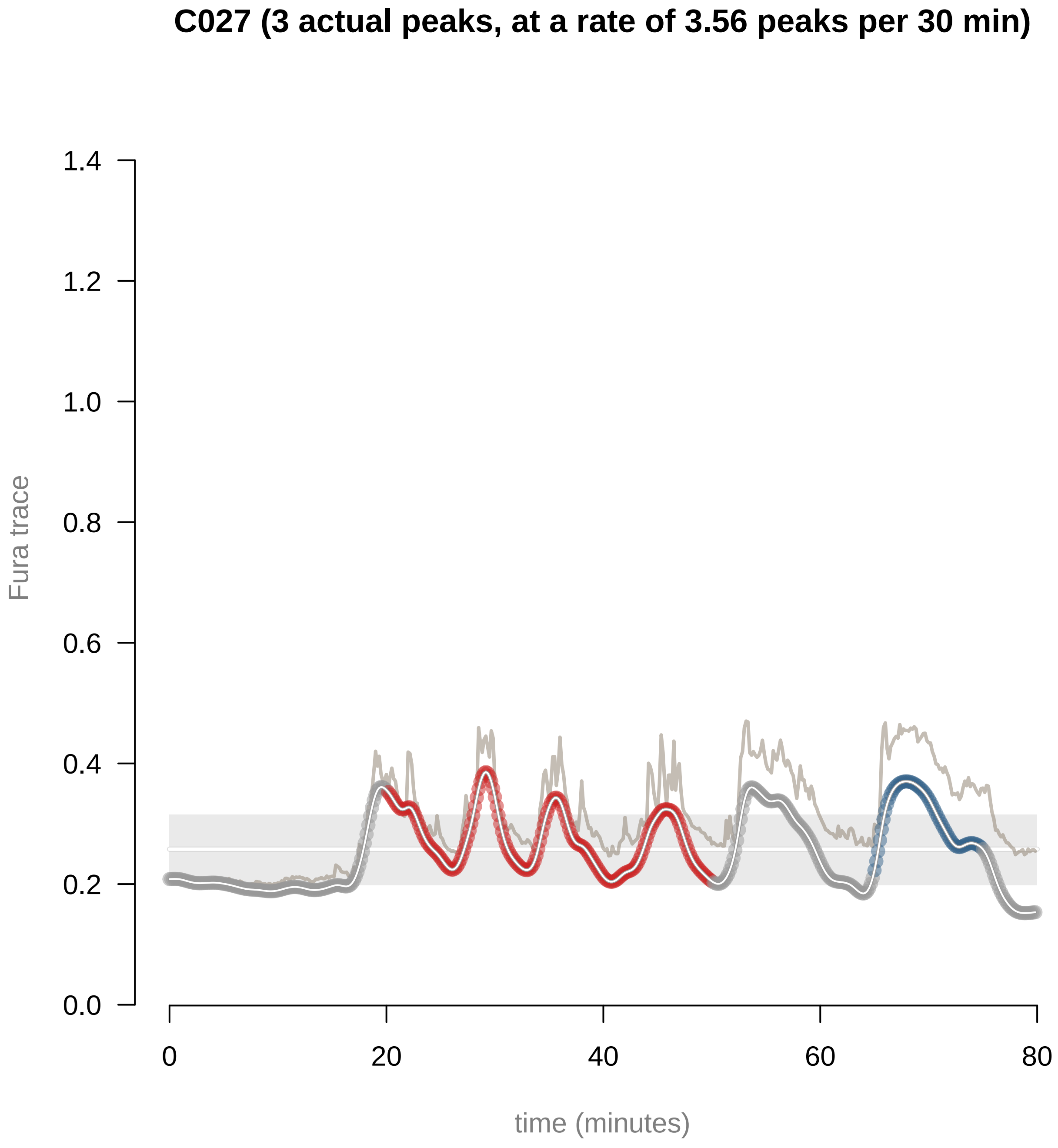
C027 (3 actual peaks, at a rate of 3.56 peaks per 30 min)
Fura trace
time (minutes)
0.0
0.2
0.4
0.6
0.8
1.0
1.2
1.4
0
20
40
60
80

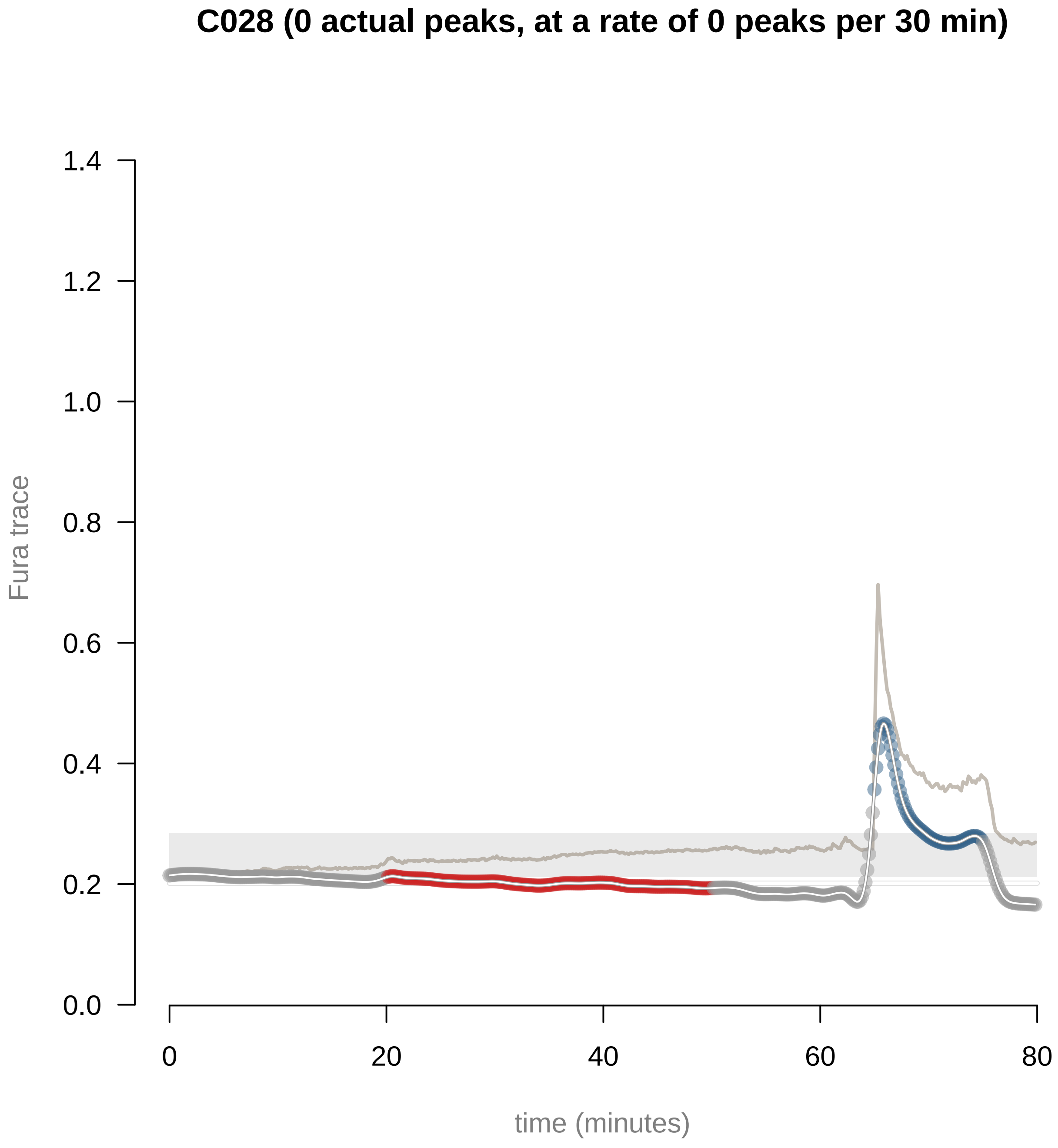

C028 (0 actual peaks, at a rate of 0 peaks per 30 min)
Fura trace
1.4
1.2
1.0
0.8
0.6
0.4
0.2
0.0
0
20
40
60
80
time (minutes)

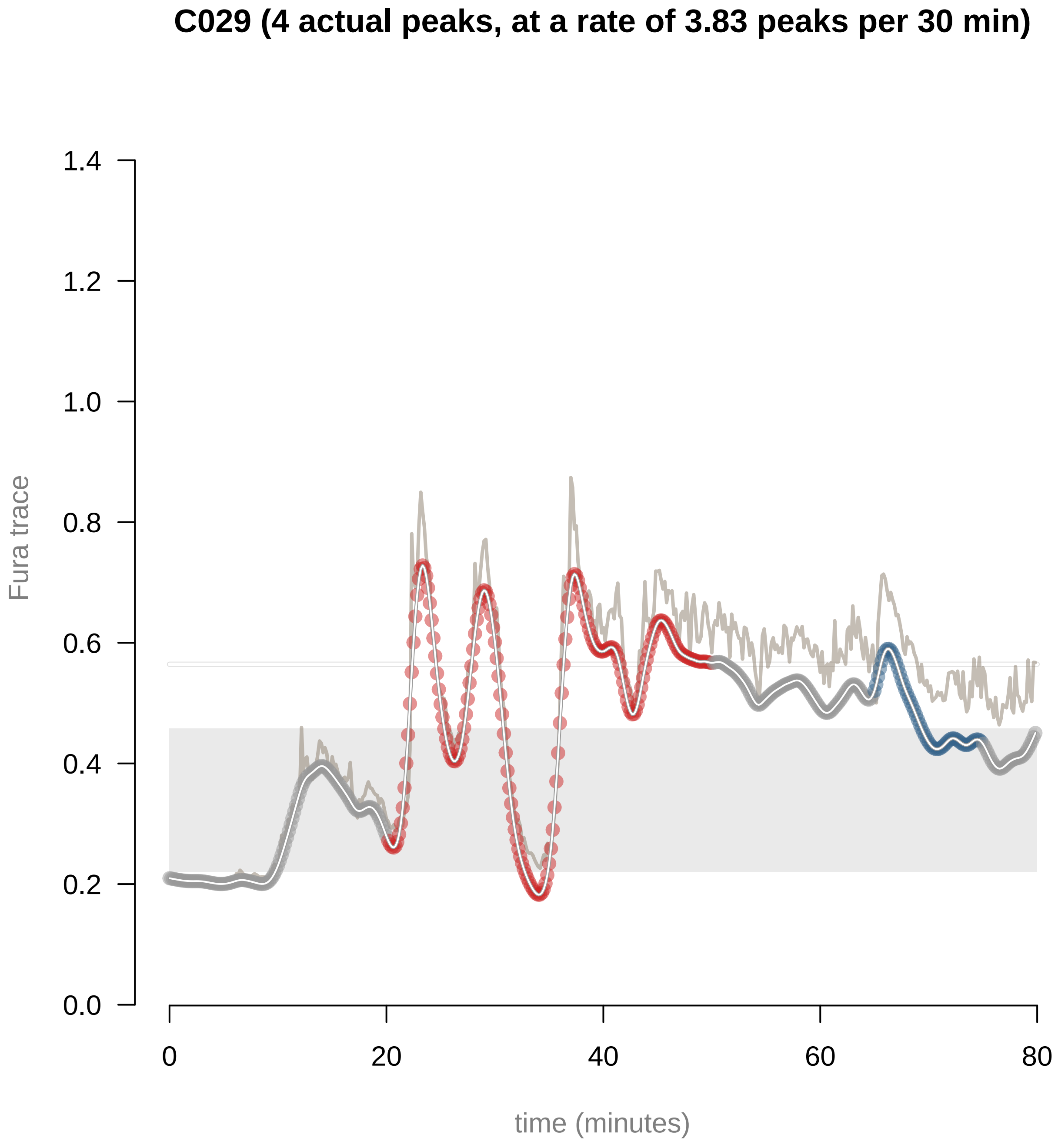
C029 (4 actual peaks, at a rate of 3.83 peaks per 30 min)
Fura trace
1.4
1.2
1.0
0.8
0.6
0.4
0.2
0.0
0
20
40
60
80
time (minutes)

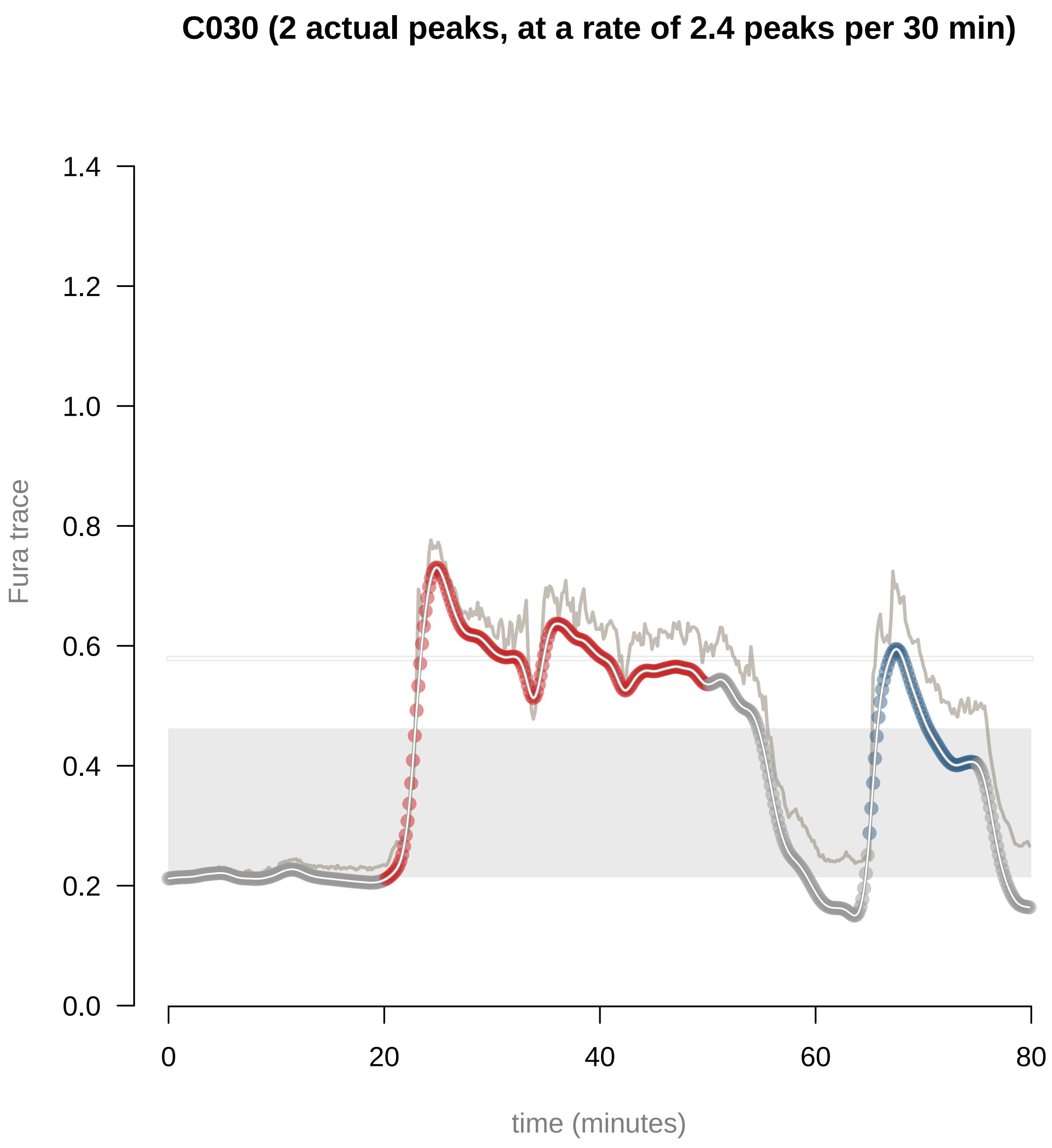

C030 (2 actual peaks, at a rate of 2.4 peaks per 30 min)
Fura trace
1.4
1.2
1.0
0.8
0.6
0.4
0.2
0.0
0
20
40
60
80
time (minutes)

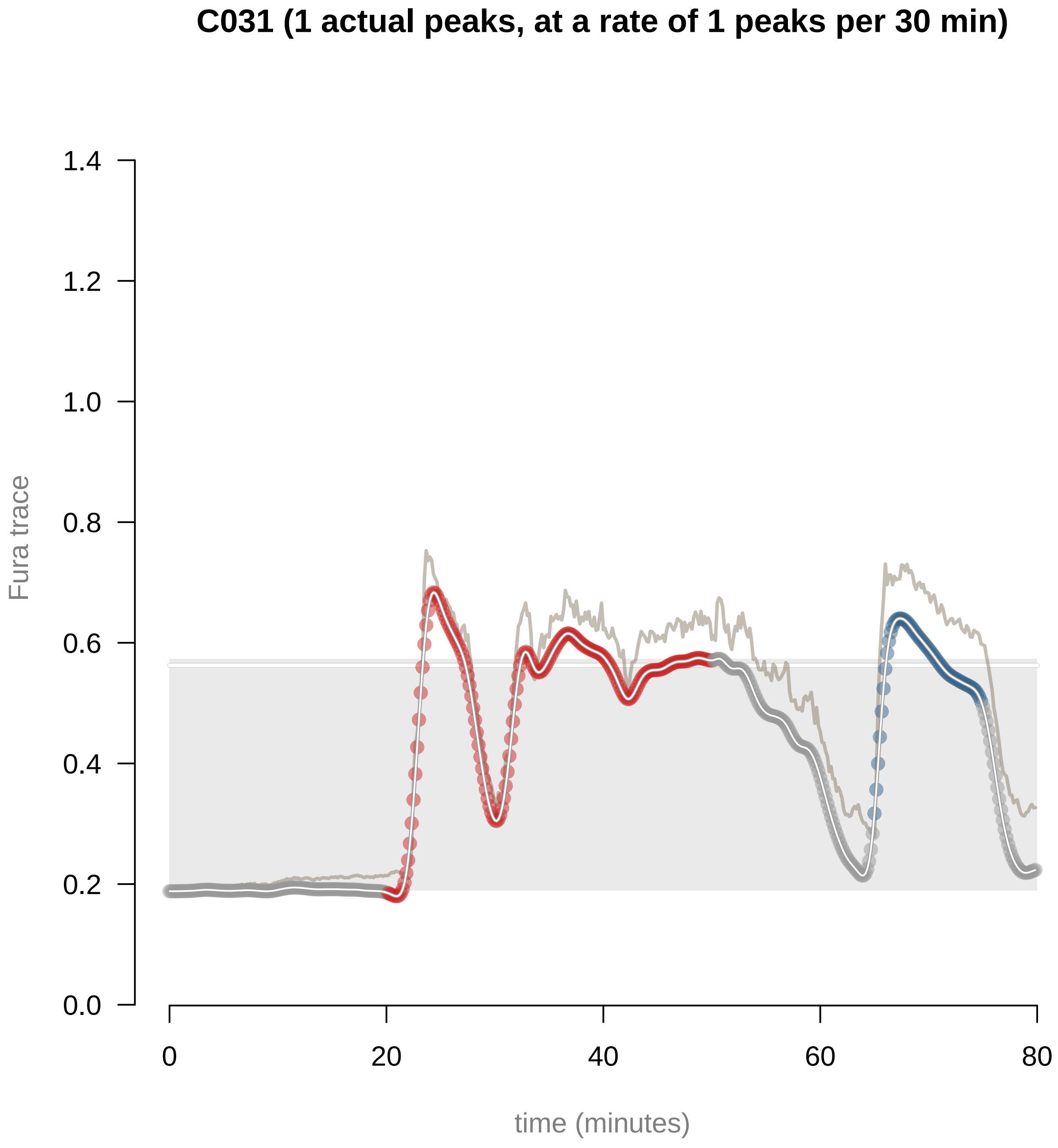
C031 (1 actual peaks, at a rate of 1 peaks per 30 min)
Fura trace
1.4
1.2
1.0
0.8
0.6
0.4
0.2
0.0
0
20
40
60
80
time (minutes)

C032 (1 actual peaks, at a rate of 1 peaks per 30 min)

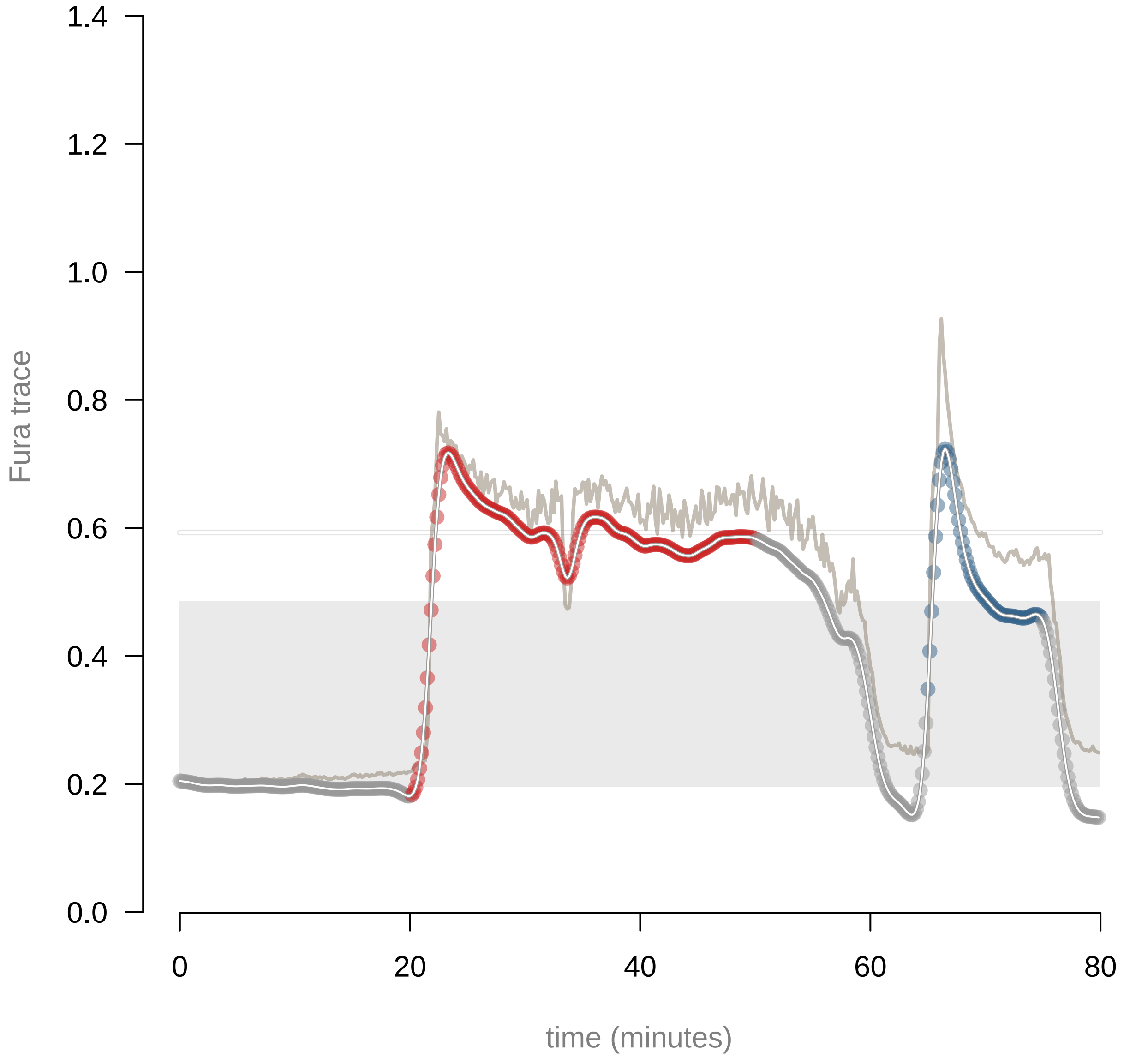

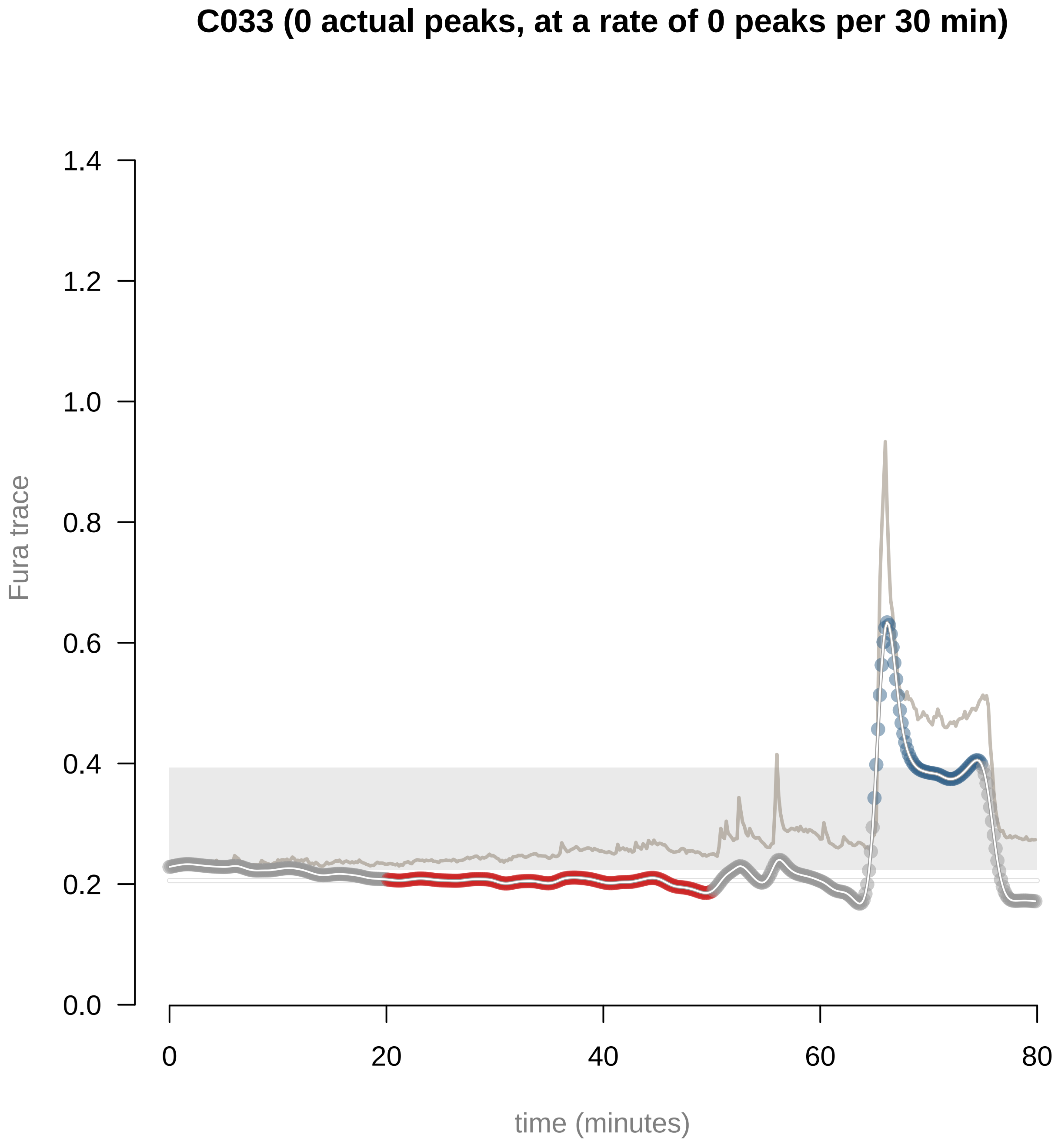

C033 (0 actual peaks, at a rate of 0 peaks per 30 min)
Fura trace
0.0
0.2
0.4
0.6
0.8
1.0
1.2
1.4
0
20
40
60
80
time (minutes)

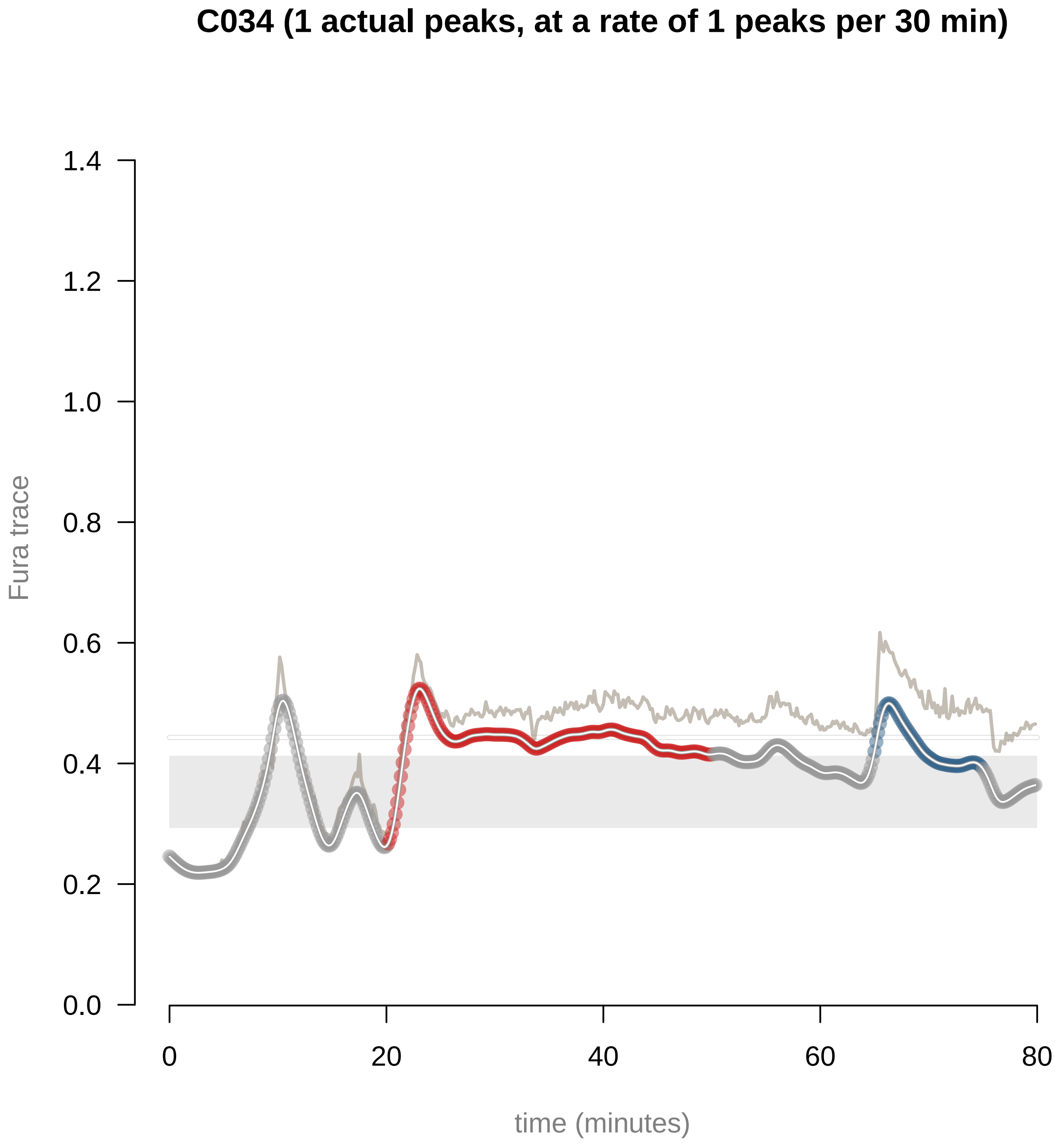
C034 (1 actual peaks, at a rate of 1 peaks per 30 min)
Fura trace
1.4
1.2
1.0
0.8
0.6
0.4
0.2
0.0
0
20
40
60
80
time (minutes)

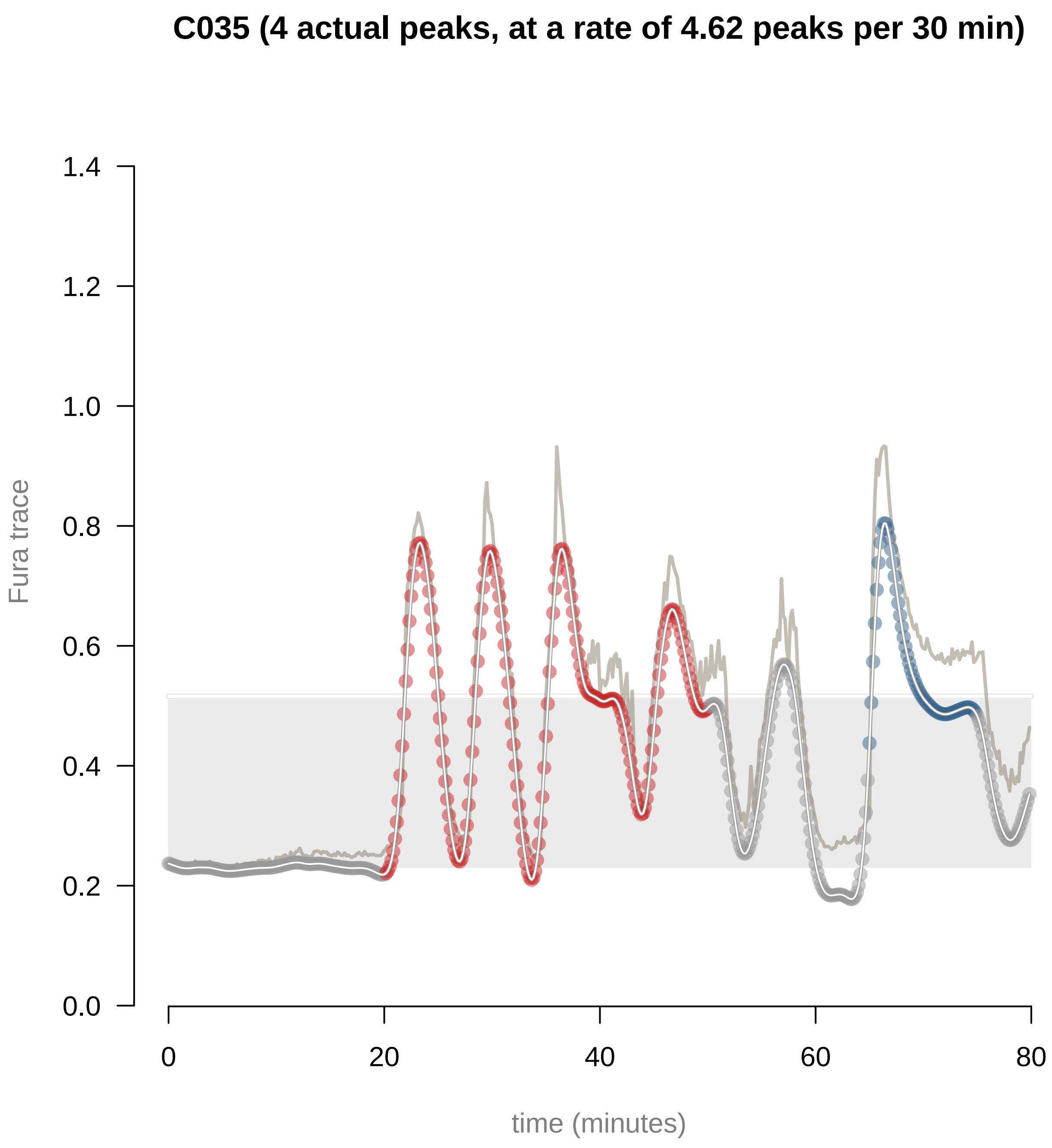

C035 (4 actual peaks, at a rate of 4.62 peaks per 30 min)
Fura trace
1.4
1.2
1.0
0.8
0.6
0.4
0.2
0.0
0
20
40
60
80
time (minutes)

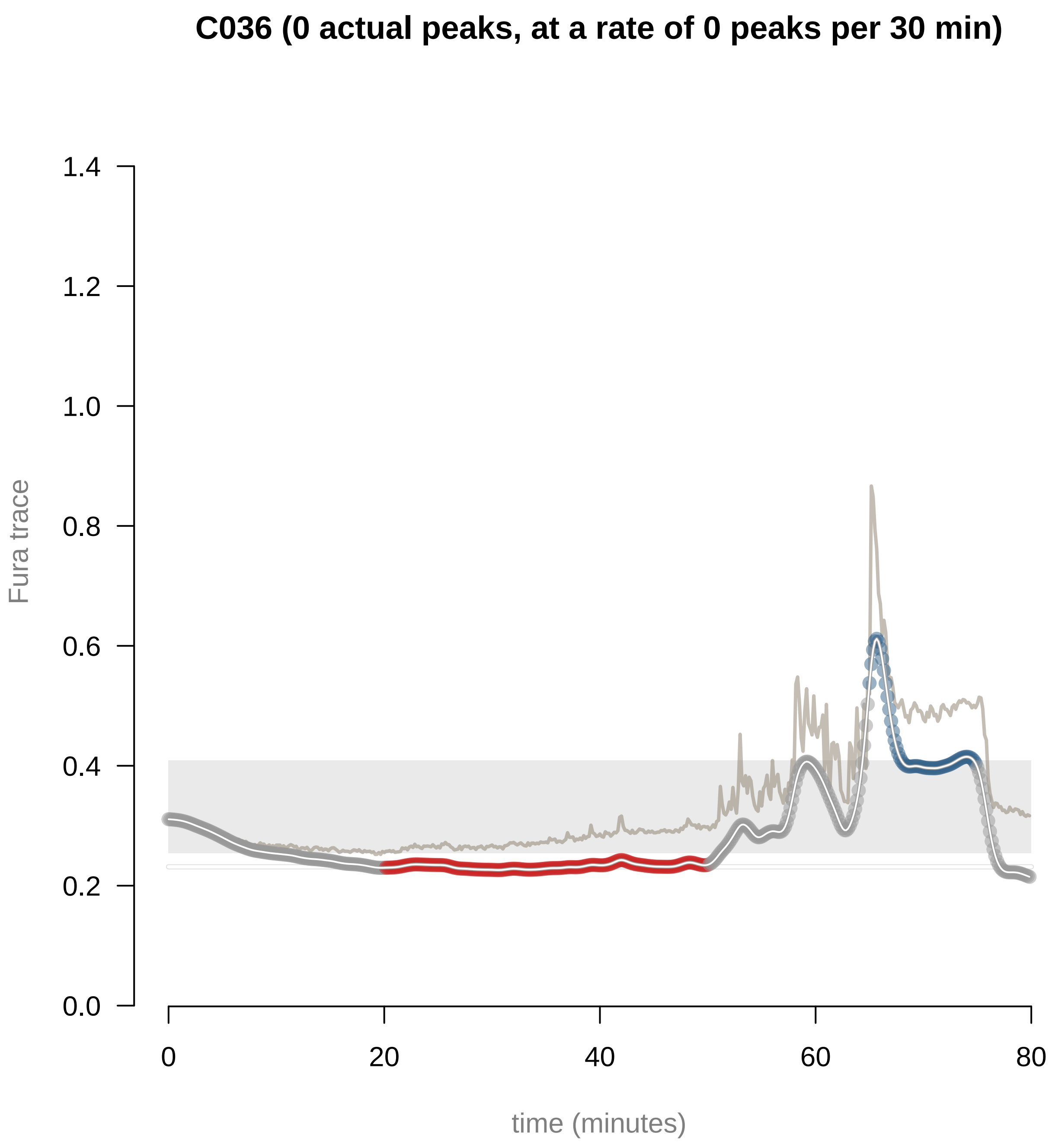
C036 (0 actual peaks, at a rate of 0 peaks per 30 min)
Fura trace
time (minutes)
0.0
0.2
0.4
0.6
0.8
1.0
1.2
1.4
0
20
40
60
80

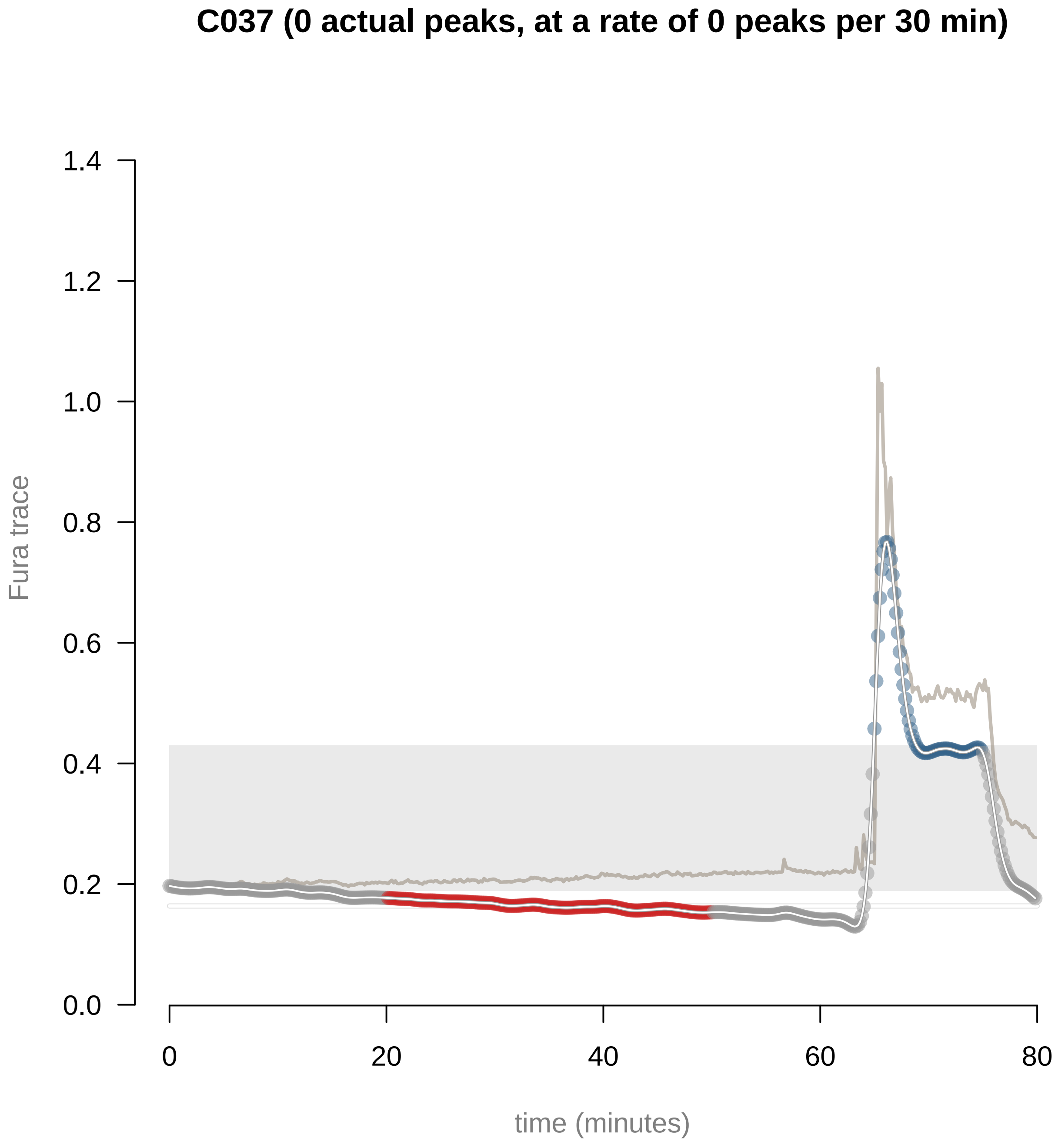

C037 (0 actual peaks, at a rate of 0 peaks per 30 min)
Fura trace
1.4
1.2
1.0
0.8
0.6
0.4
0.2
0.0
0
20
40
60
80
time (minutes)

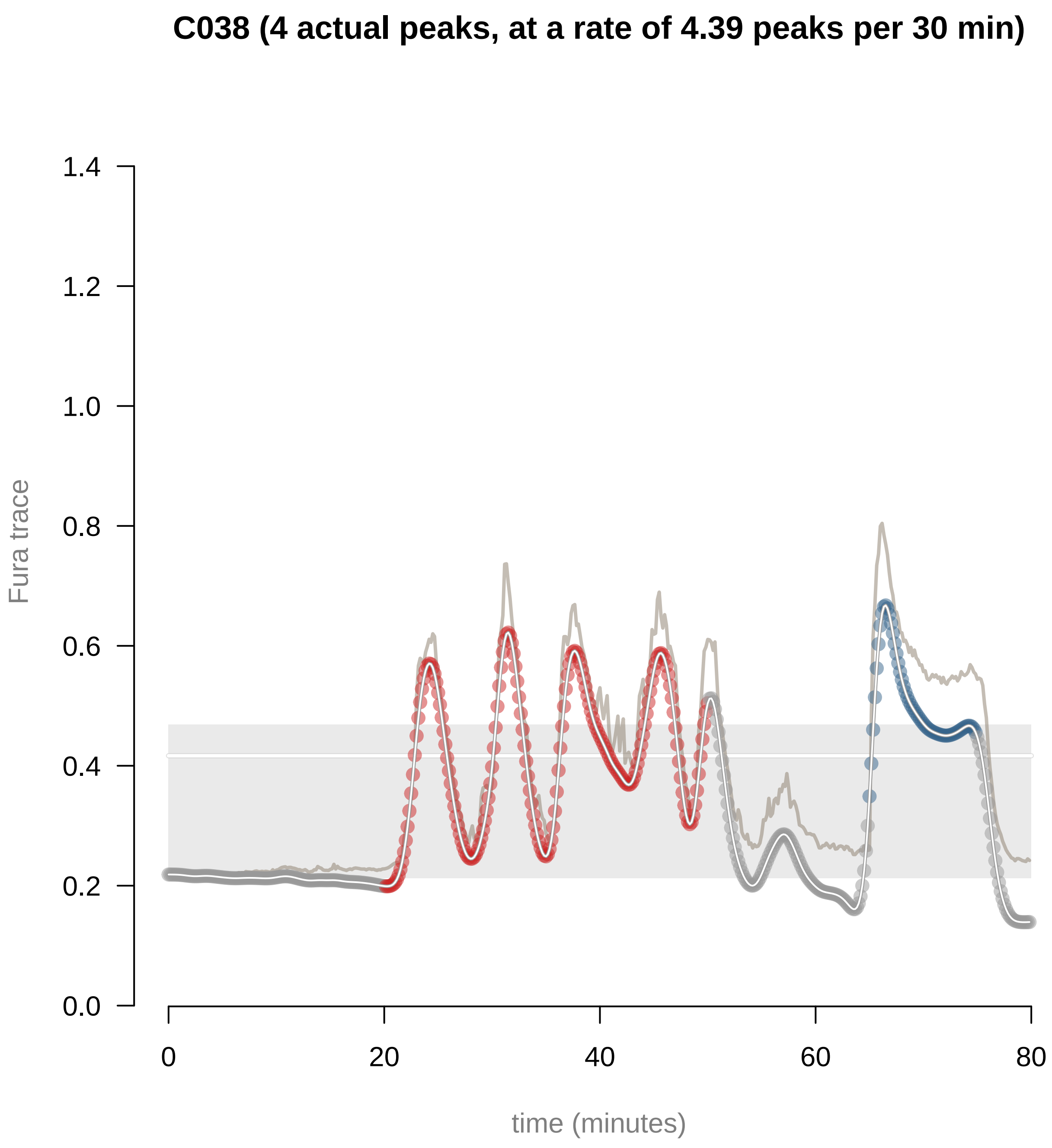
C038 (4 actual peaks, at a rate of 4.39 peaks per 30 min)
Fura trace
0.0
0.2
0.4
0.6
0.8
1.0
1.2
1.4
0
20
40
60
80
time (minutes)

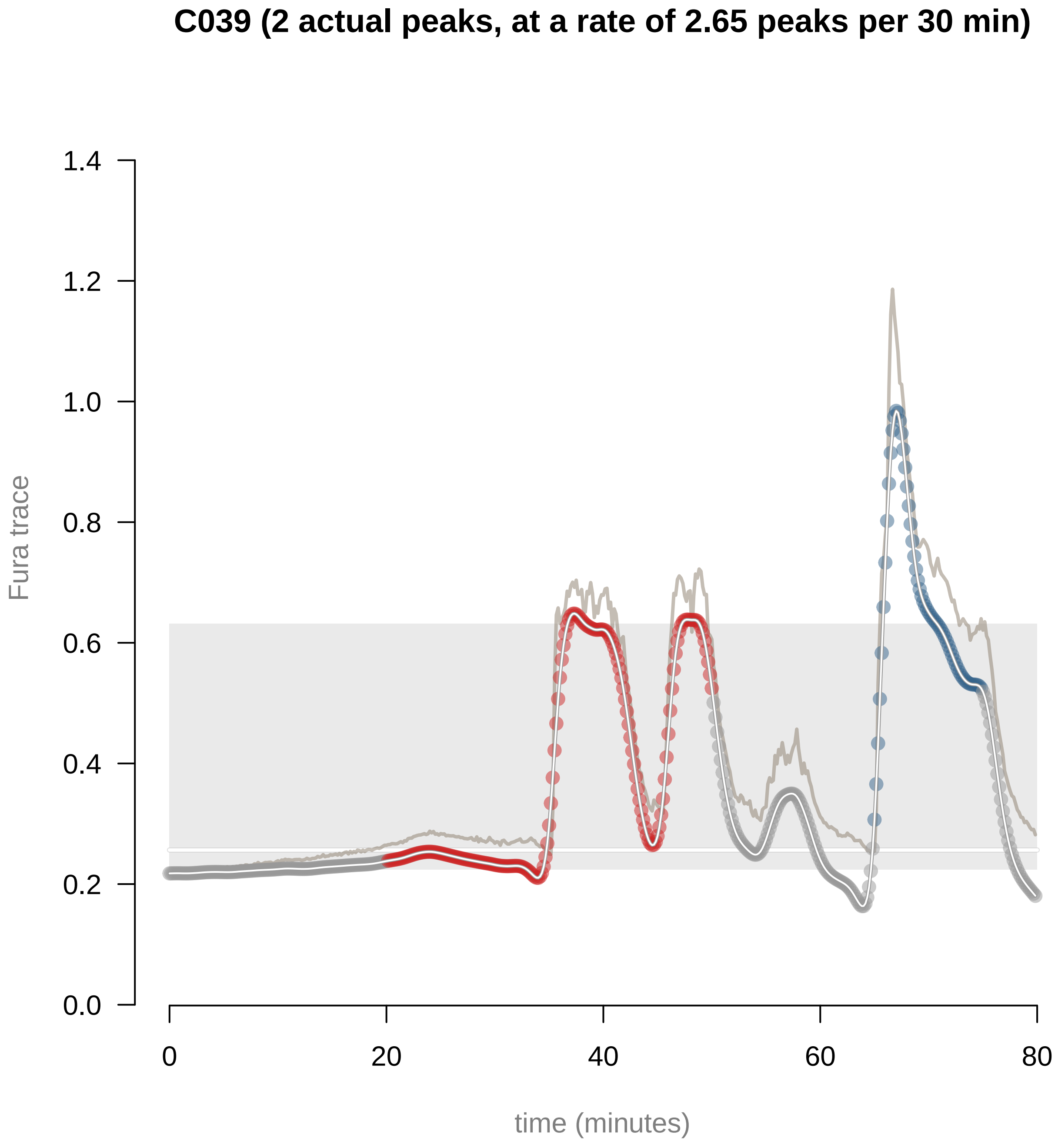
C039 (2 actual peaks, at a rate of 2.65 peaks per 30 min)
Fura trace
1.4
1.2
1.0
0.8
0.6
0.4
0.2
0.0
0
20
40
60
80
time (minutes)

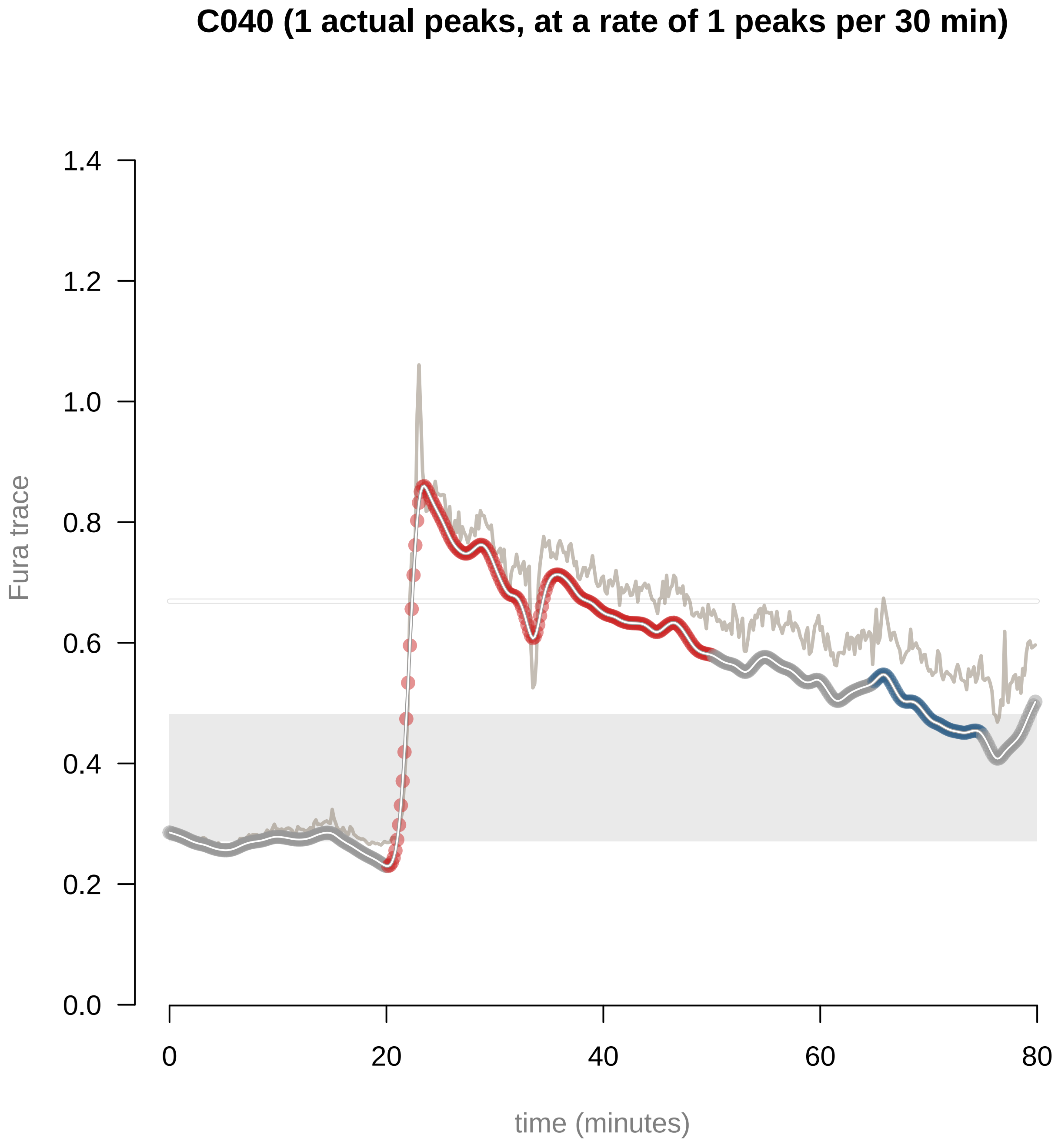

C040 (1 actual peaks, at a rate of 1 peaks per 30 min)
Fura trace
time (minutes)
0.0
0.2
0.4
0.6
0.8
1.0
1.2
1.4
0
20
40
60
80

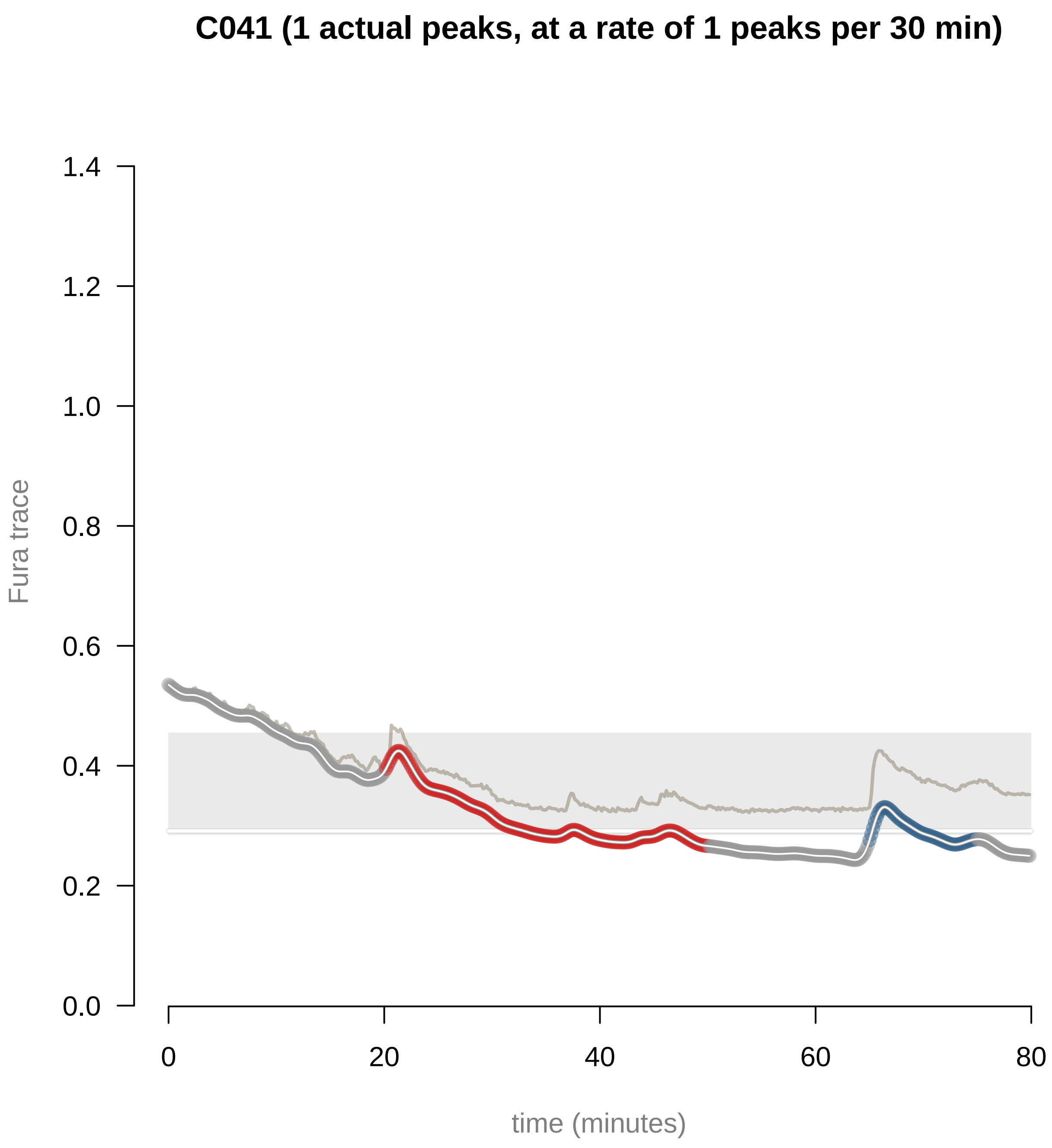
C041 (1 actual peaks, at a rate of 1 peaks per 30 min)
Fura trace
time (minutes)
0.0
0.2
0.4
0.6
0.8
1.0
1.2
1.4
0
20
40
60
80

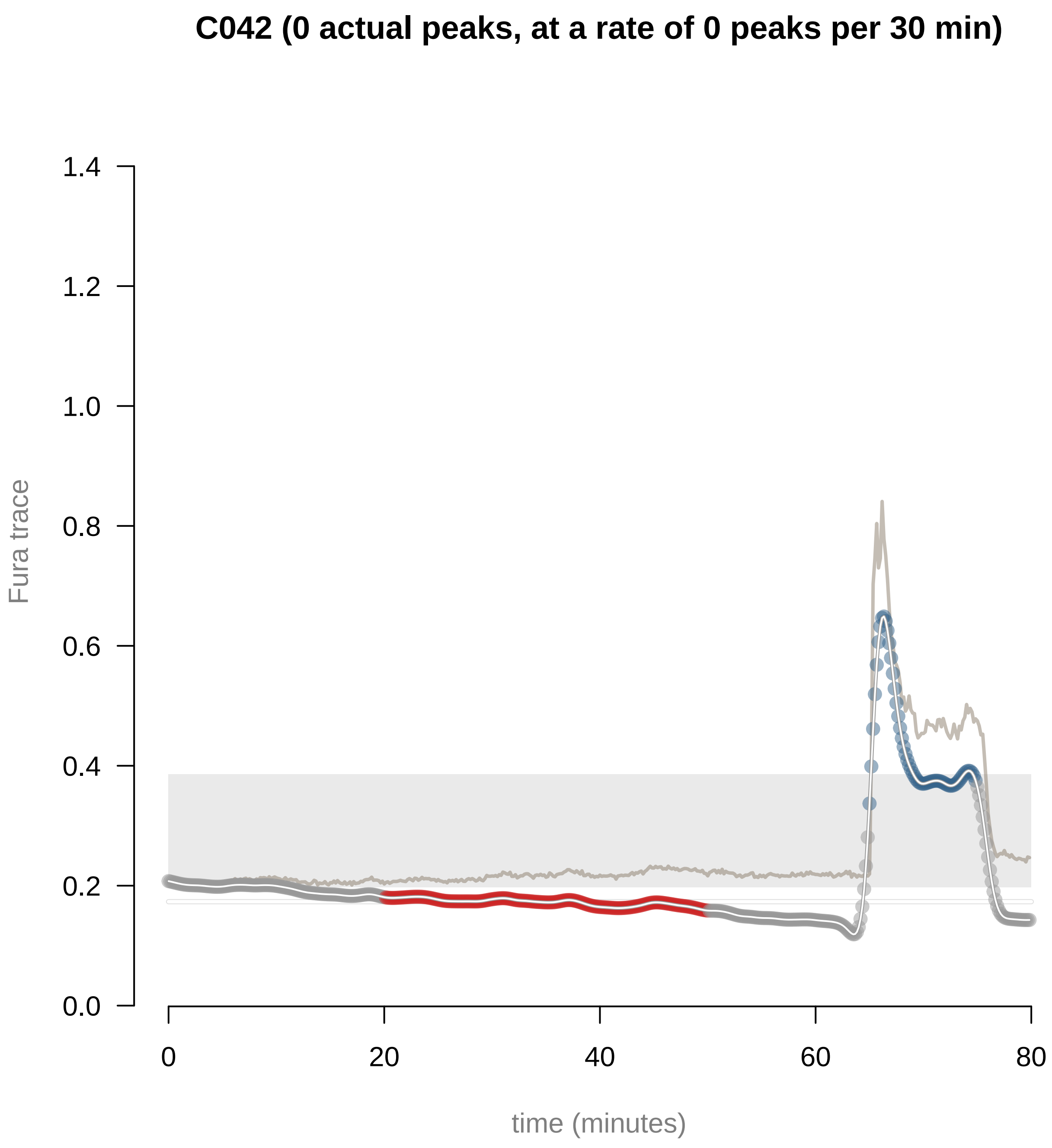
C042 (0 actual peaks, at a rate of 0 peaks per 30 min)
Fura trace
time (minutes)
0.0
0.2
0.4
0.6
0.8
1.0
1.2
1.4
0
20
40
60
80

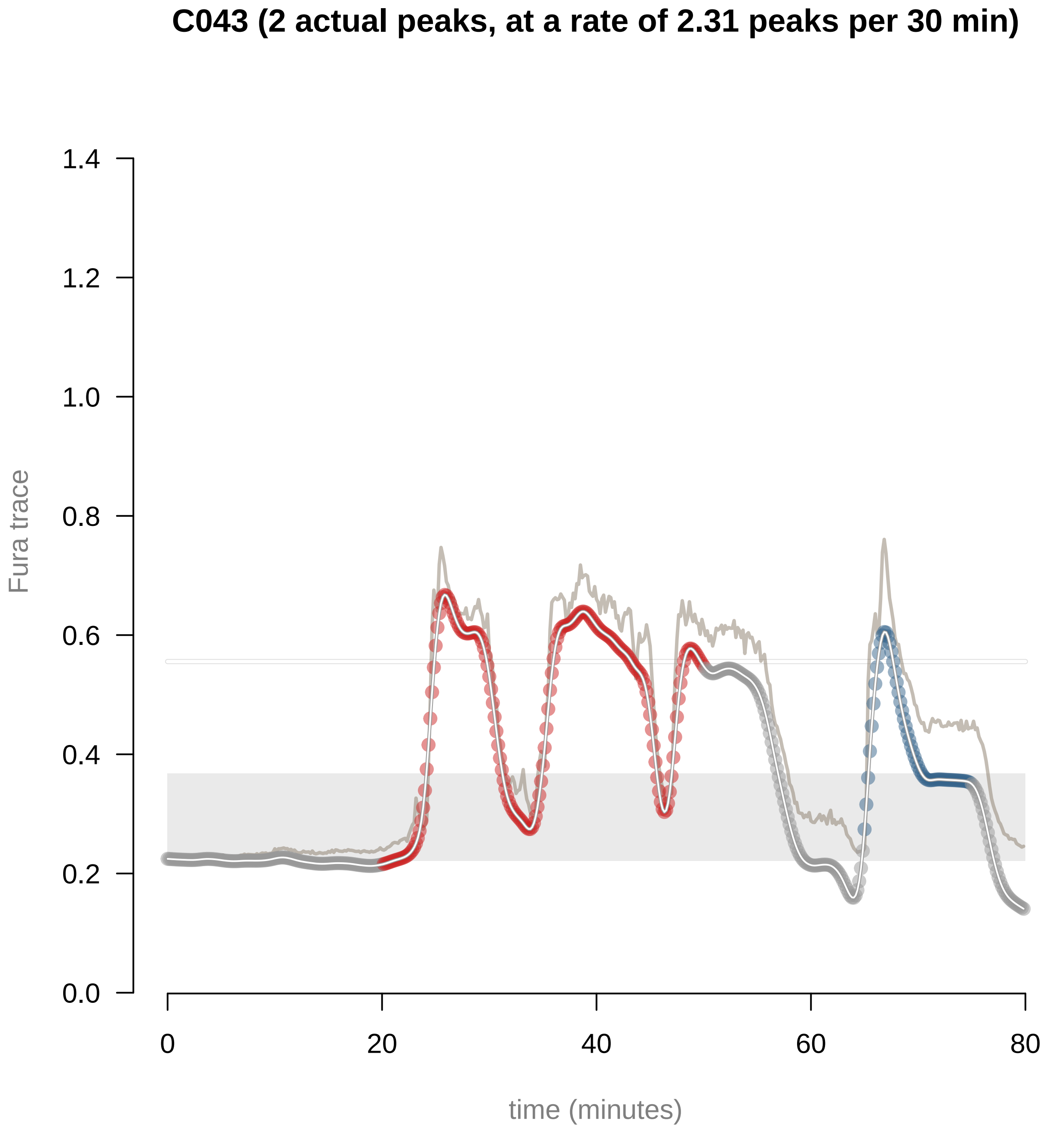

C043 (2 actual peaks, at a rate of 2.31 peaks per 30 min)
Fura trace
0.0
0.2
0.4
0.6
0.8
1.0
1.2
1.4
0
20
40
60
80
time (minutes)

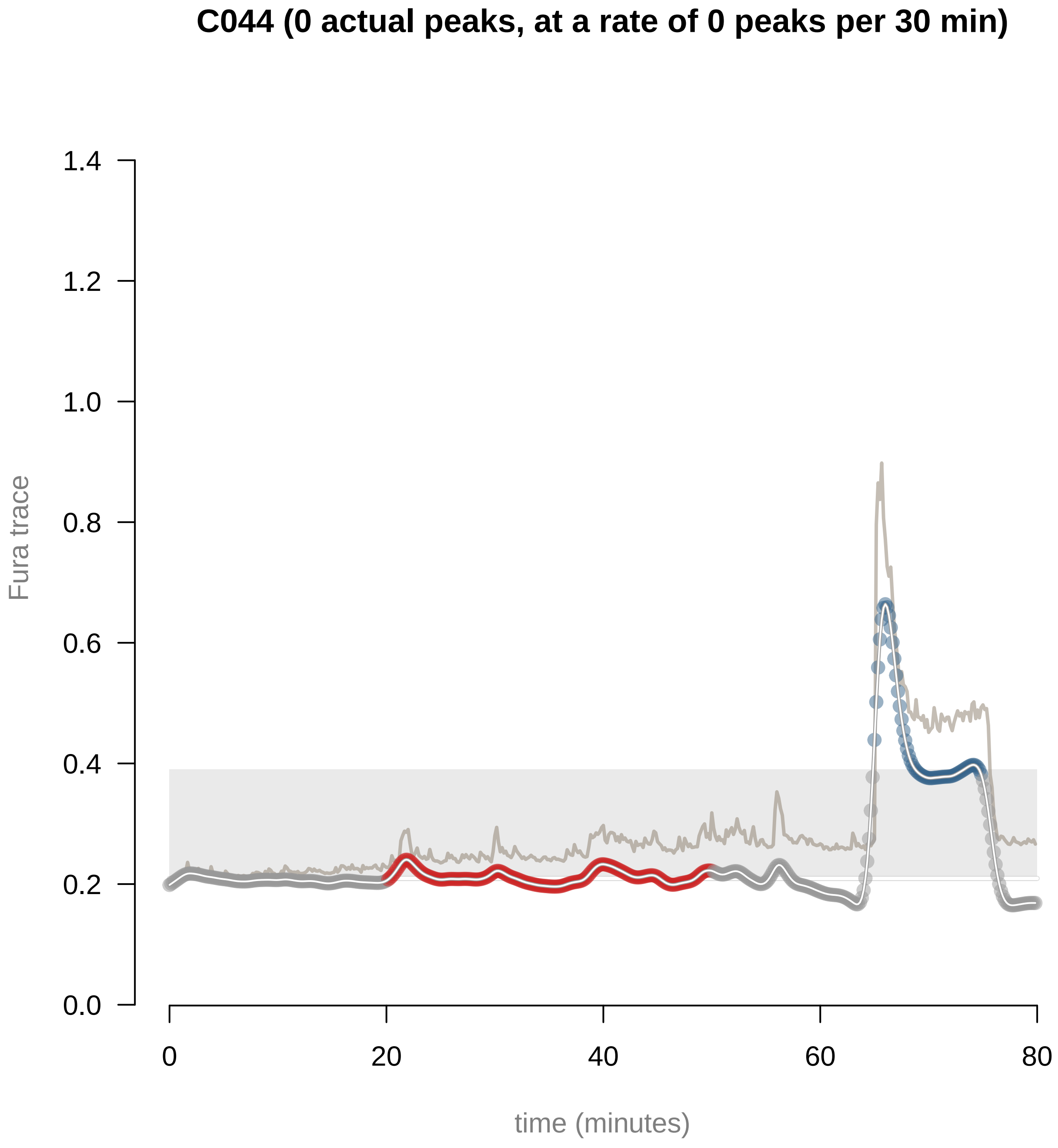

C044 (0 actual peaks, at a rate of 0 peaks per 30 min)
Fura trace
time (minutes)
0.0
0.2
0.4
0.6
0.8
1.0
1.2
1.4
0
20
40
60
80

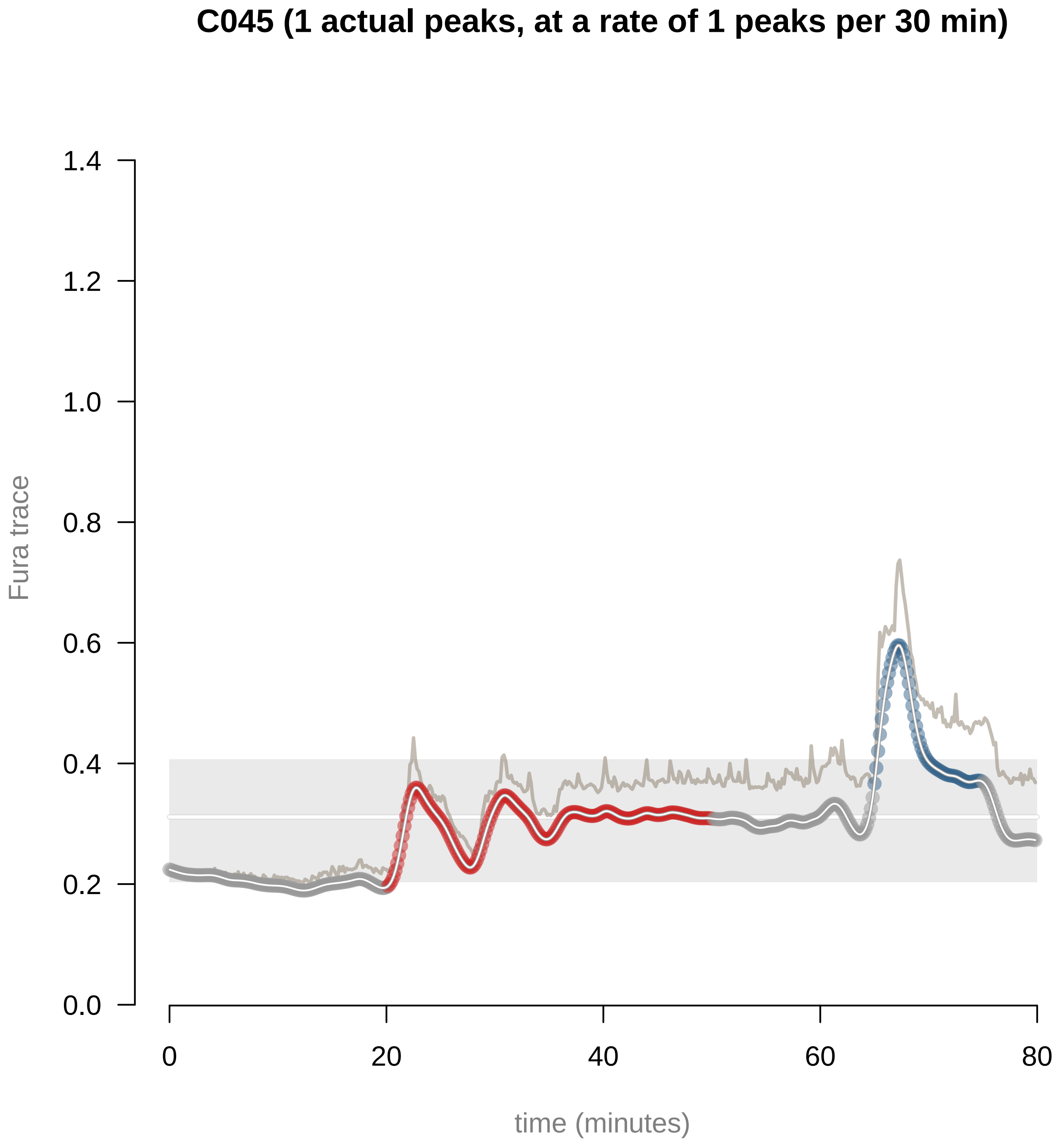
C045 (1 actual peaks, at a rate of 1 peaks per 30 min)
Fura trace
1.4
1.2
1.0
0.8
0.6
0.4
0.2
0.0
0
20
40
60
80
time (minutes)

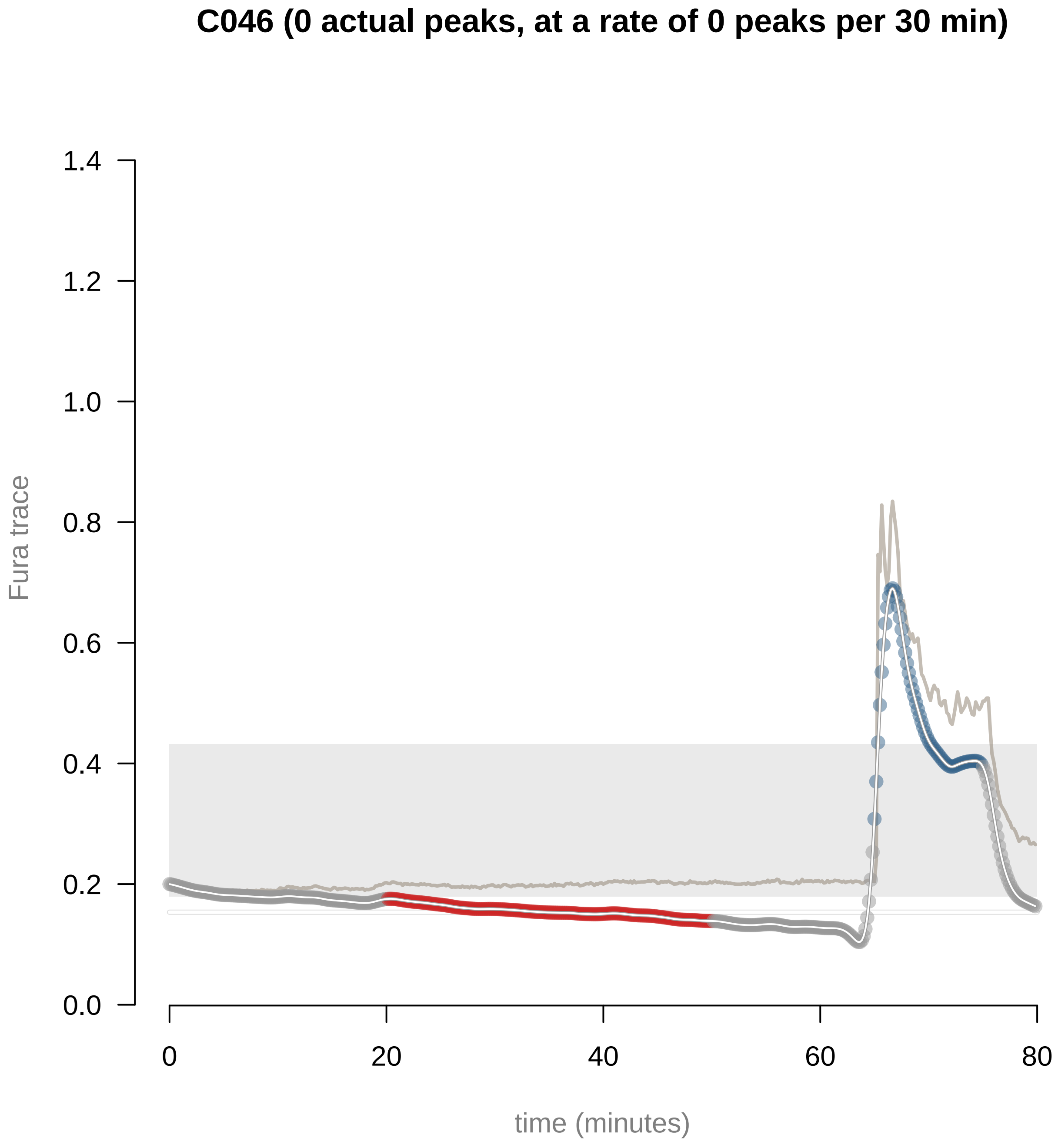
C046 (0 actual peaks, at a rate of 0 peaks per 30 min)
Fura trace
0.0
0.2
0.4
0.6
0.8
1.0
1.2
1.4
0
20
40
60
80
time (minutes)

C047 (2 actual peaks, at a rate of 6.67 peaks per 30 min)

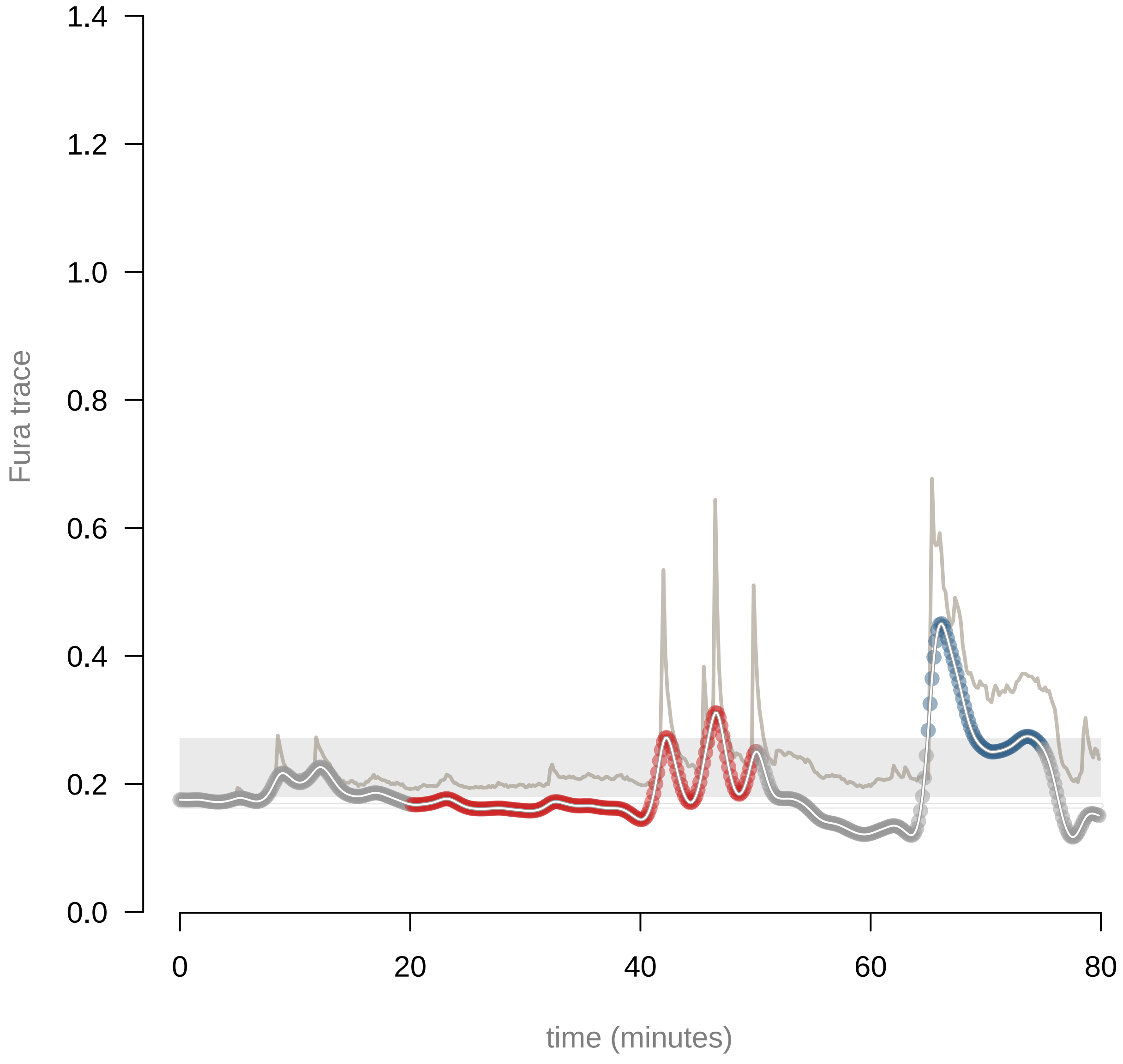

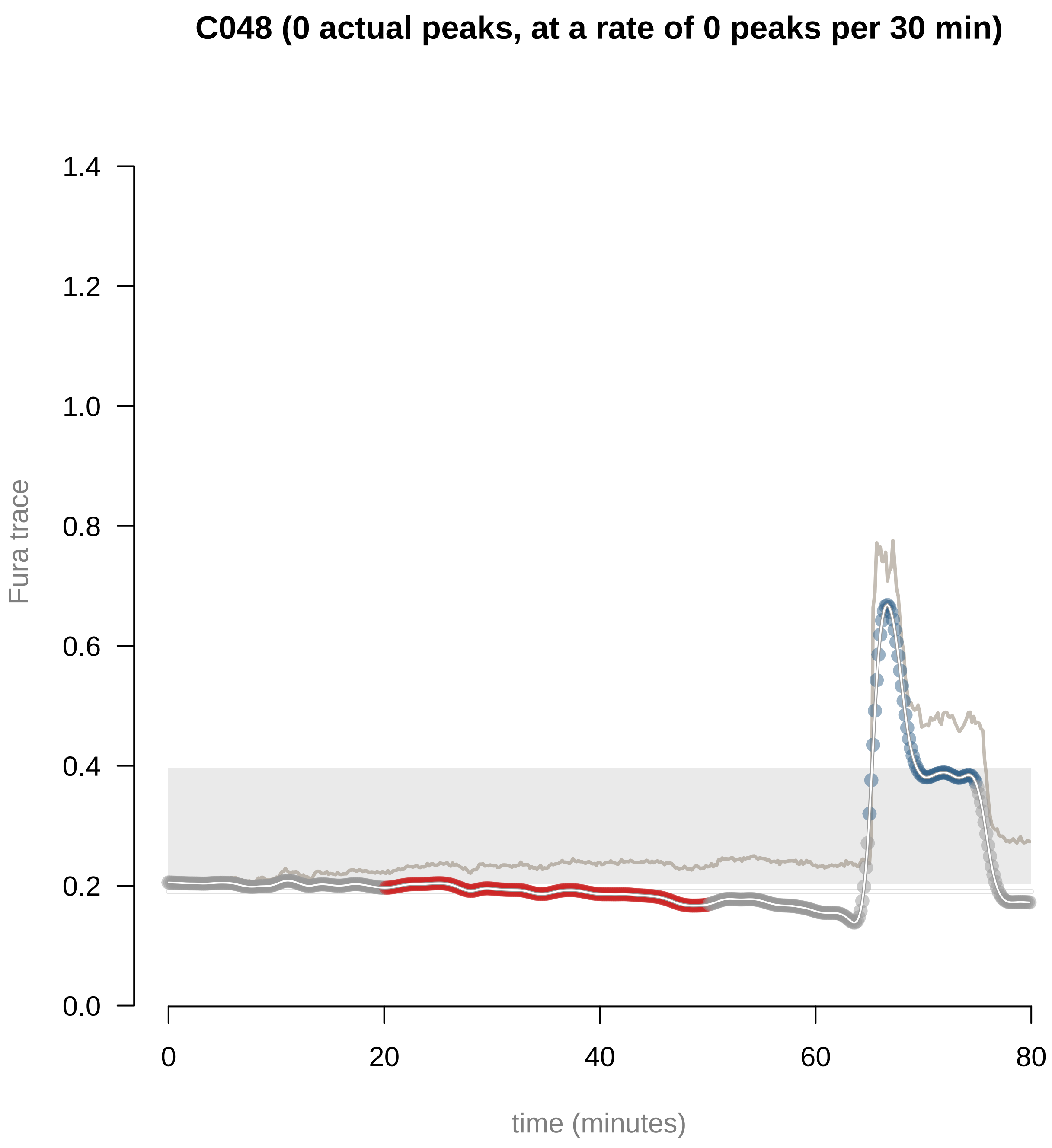

C048 (0 actual peaks, at a rate of 0 peaks per 30 min)
Fura trace
time (minutes)
0.0
0.2
0.4
0.6
0.8
1.0
1.2
1.4
0
20
40
60
80

C049 (2 actual peaks, at a rate of 5.81 peaks per 30 min)

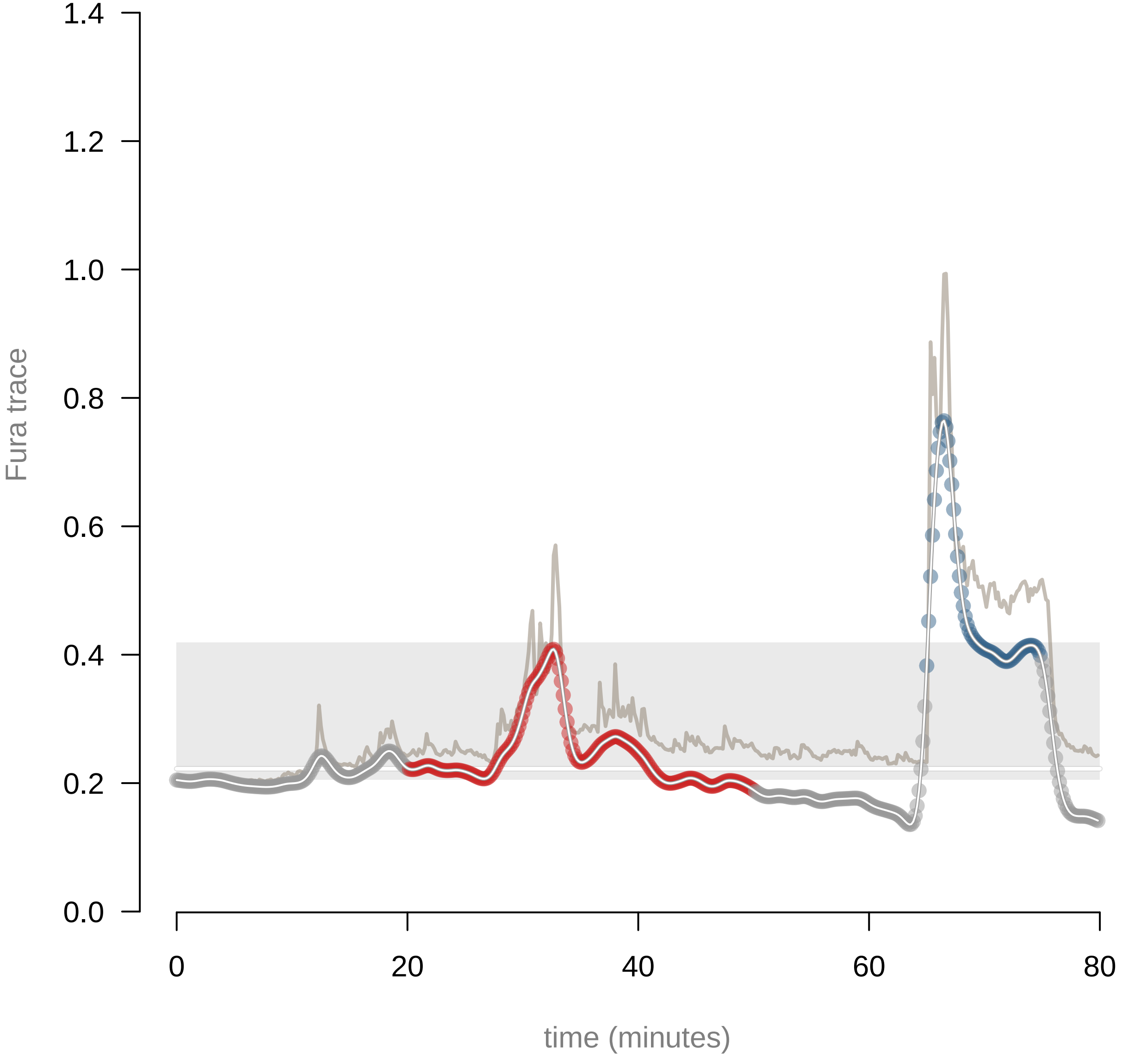

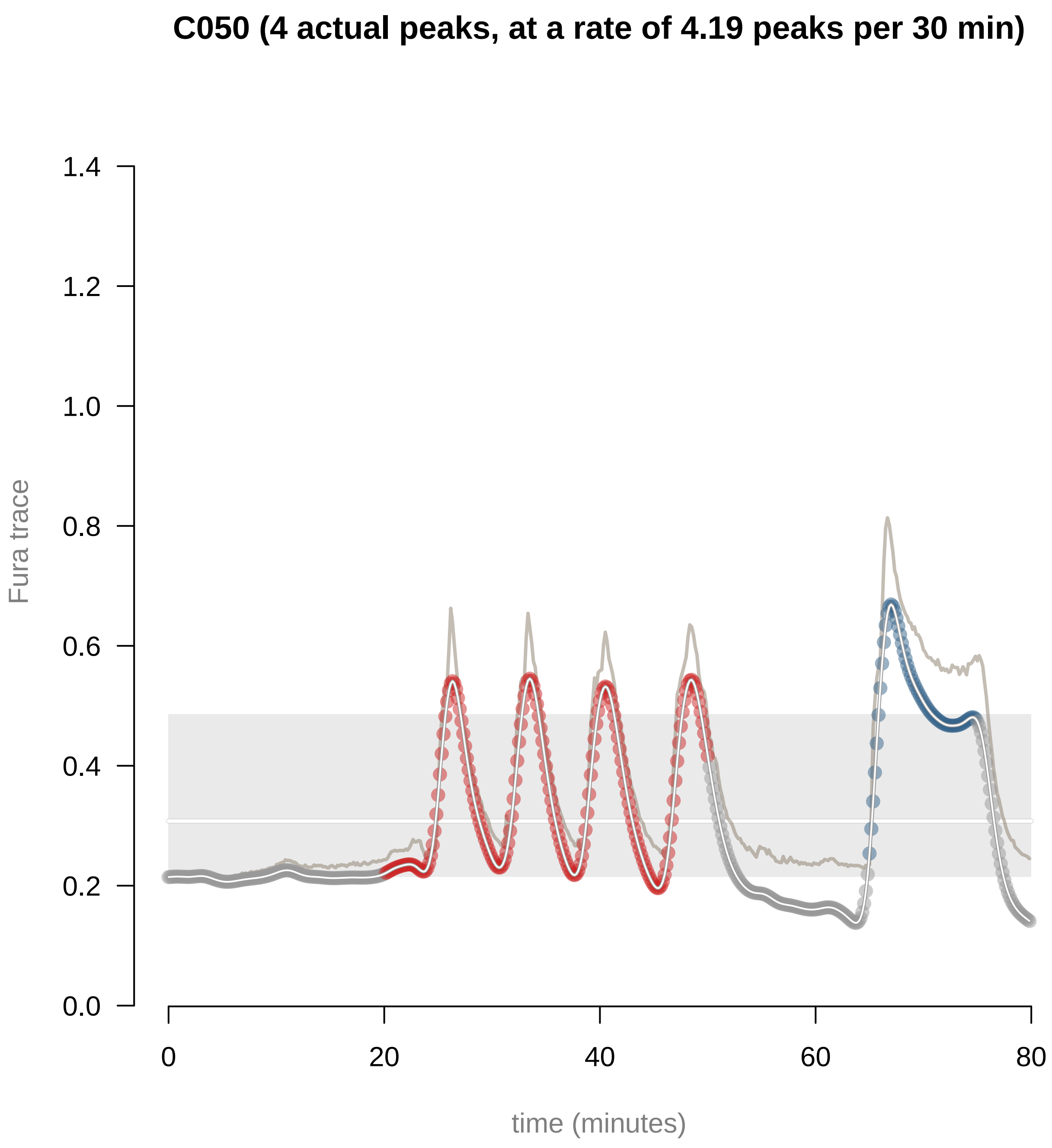
C050 (4 actual peaks, at a rate of 4.19 peaks per 30 min)
Fura trace
1.4
1.2
1.0
0.8
0.6
0.4
0.2
0.0
0
20
40
60
80
time (minutes)

C051 (3 actual peaks, at a rate of 4.29 peaks per 30 min)

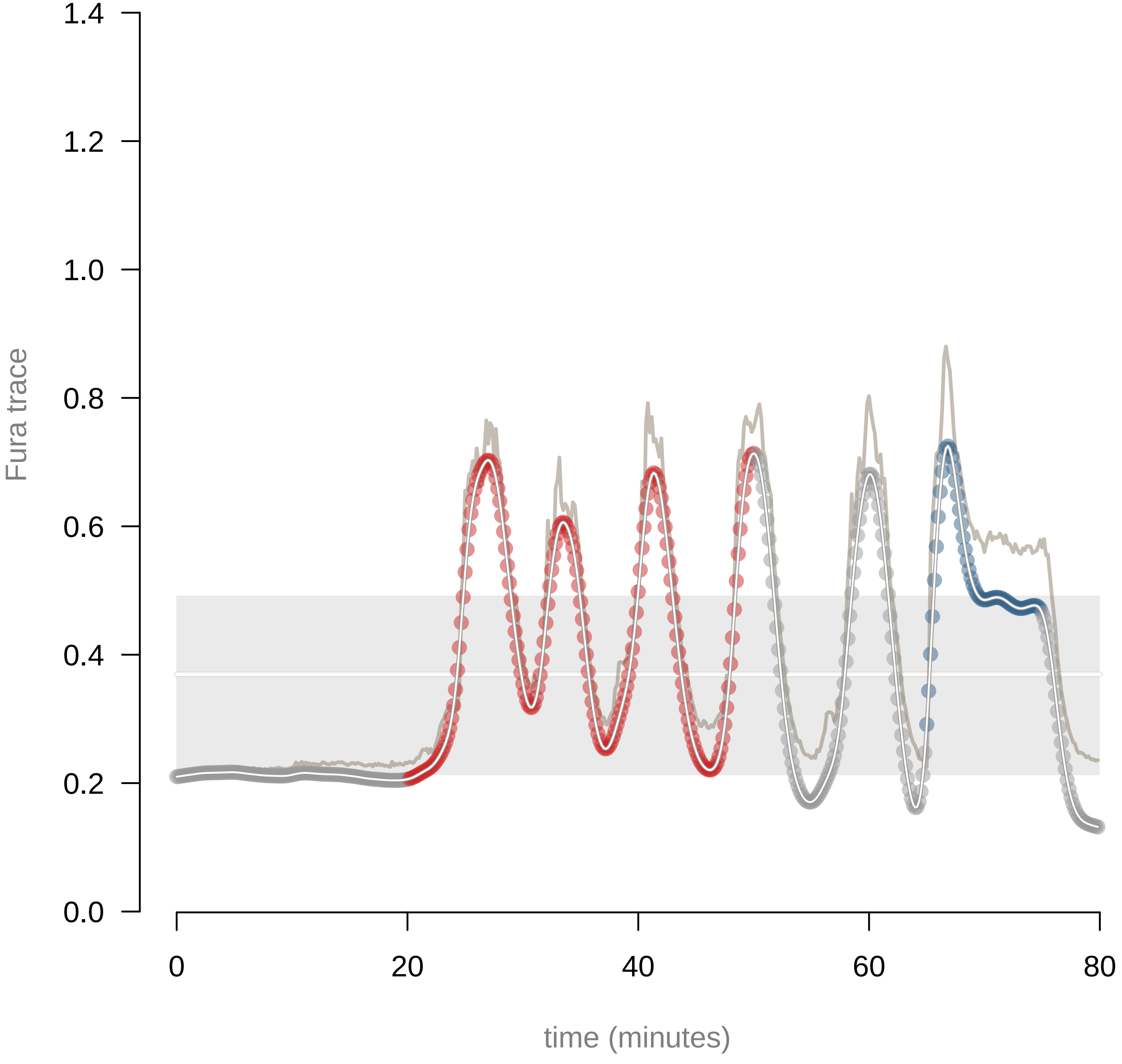

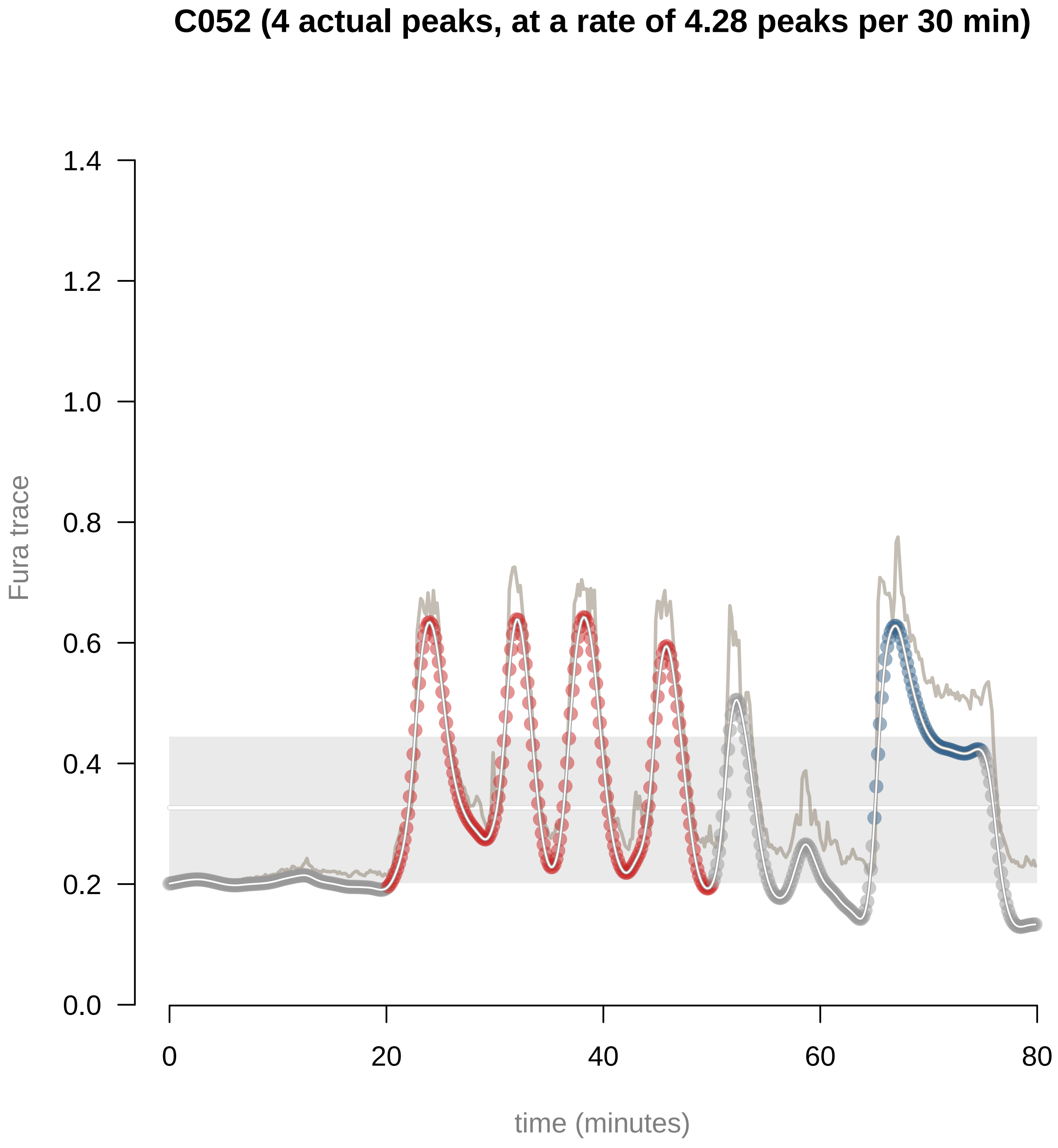
C052 (4 actual peaks, at a rate of 4.28 peaks per 30 min)
Fura trace
1.4
1.2
1.0
0.8
0.6
0.4
0.2
0.0
0
20
40
60
80
time (minutes)

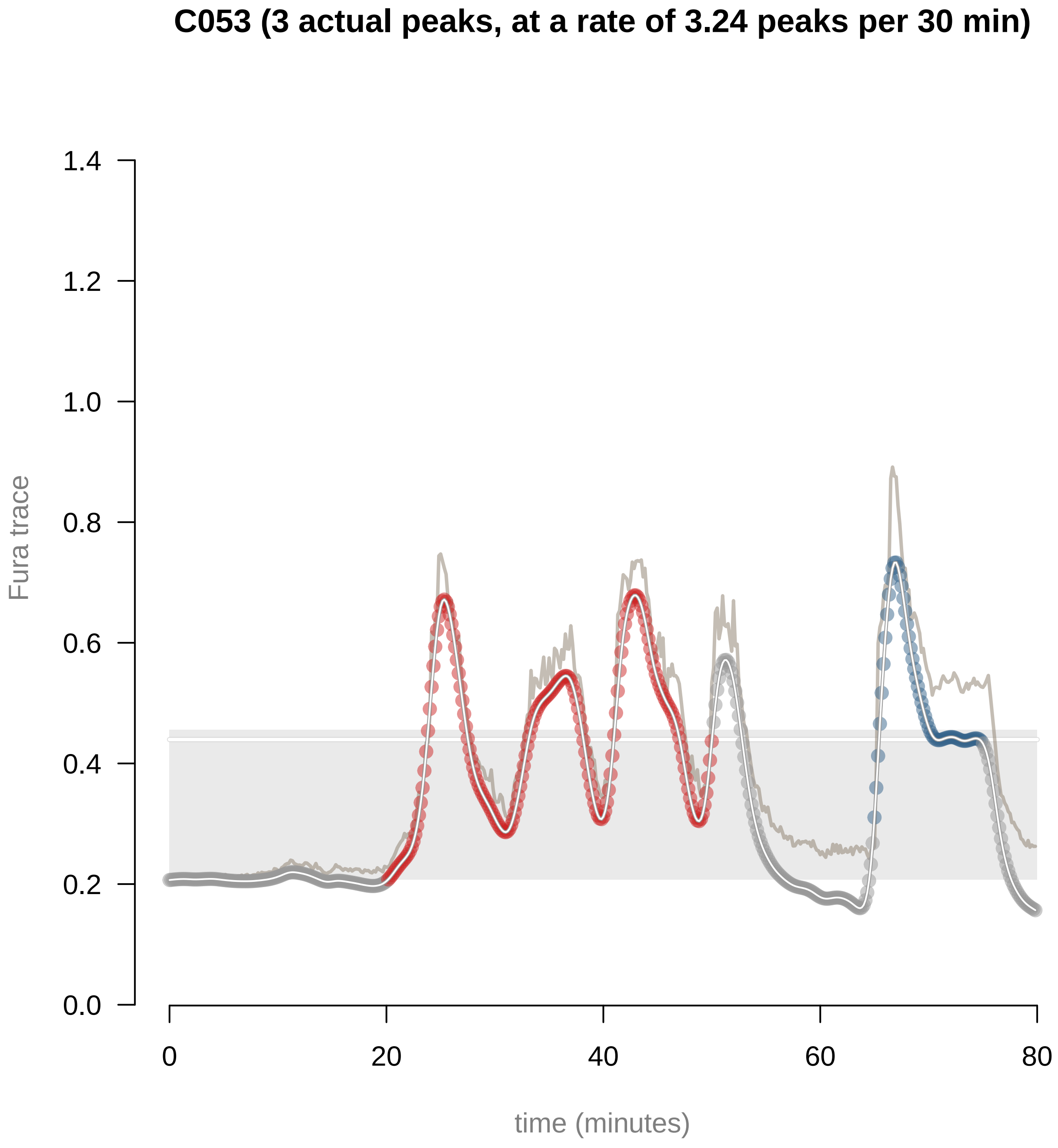

C053 (3 actual peaks, at a rate of 3.24 peaks per 30 min)
Fura trace
time (minutes)
0.0
0.2
0.4
0.6
0.8
1.0
1.2
1.4
0
20
40
60
80

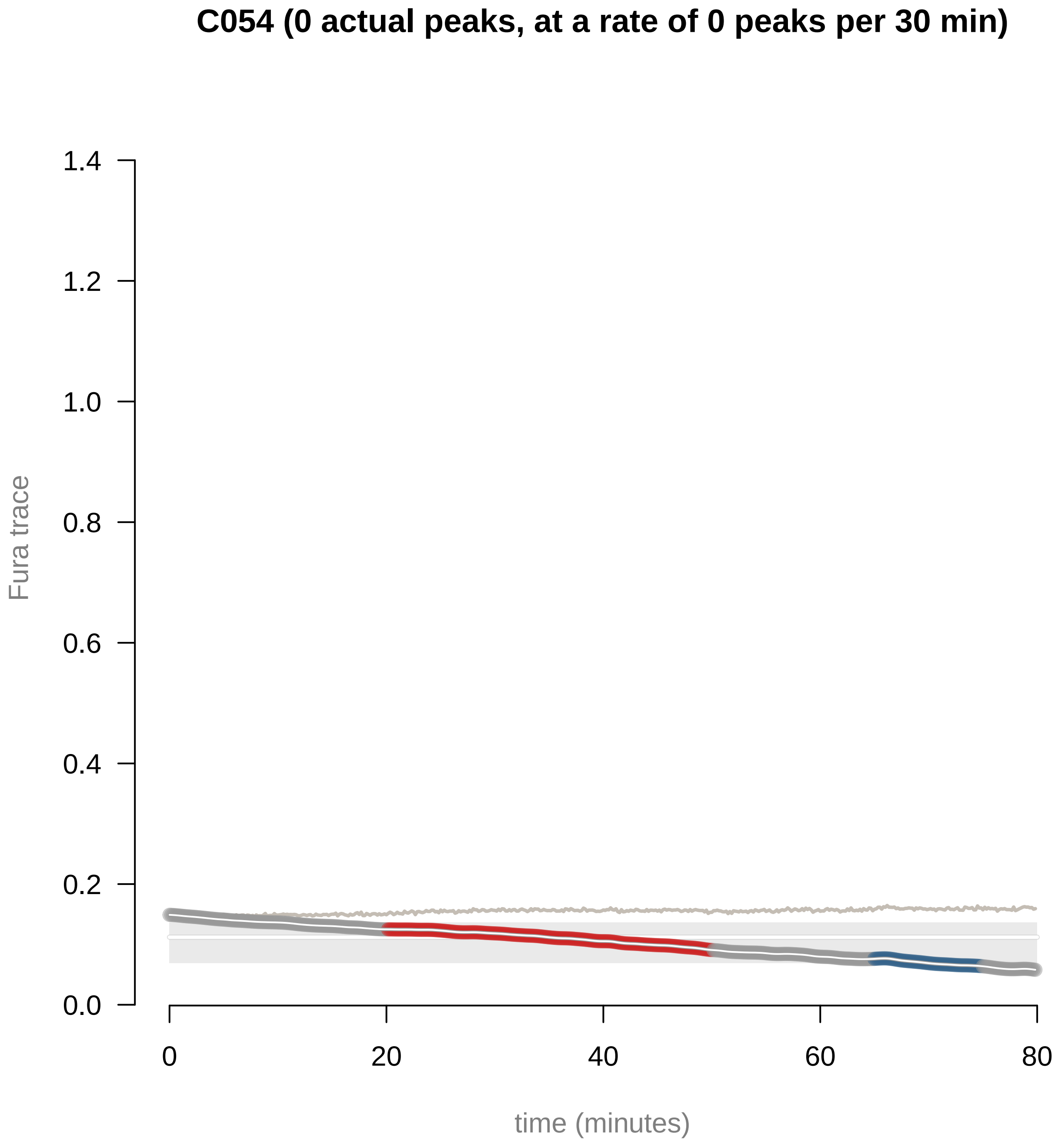
C054 (0 actual peaks, at a rate of 0 peaks per 30 min)
Fura trace
1.4
1.2
1.0
0.8
0.6
0.4
0.2
0.0
0
20
40
60
80
time (minutes)

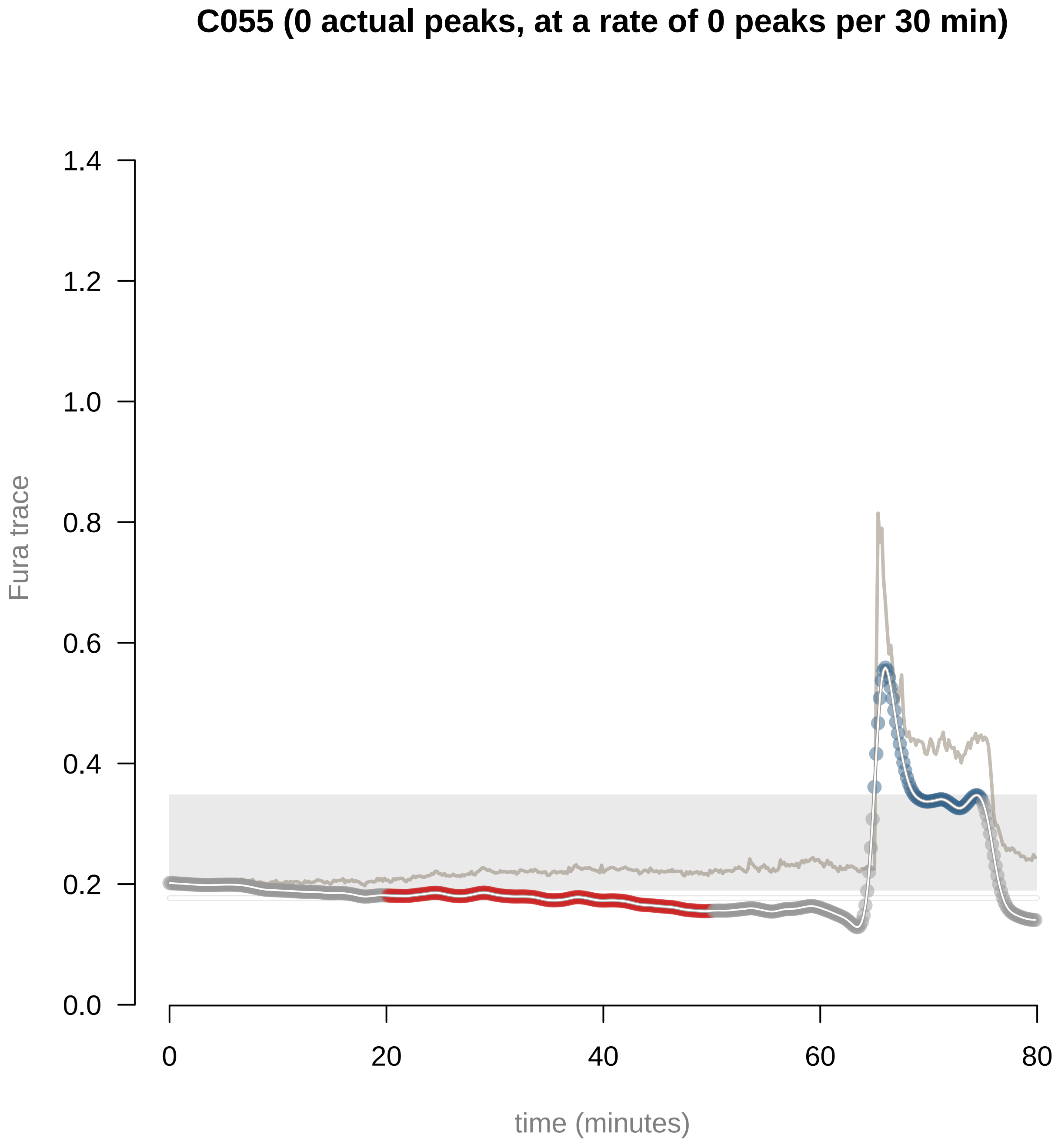

C055 (0 actual peaks, at a rate of 0 peaks per 30 min)
Fura trace
0.0
0.2
0.4
0.6
0.8
1.0
1.2
1.4
0
20
40
60
80
time (minutes)

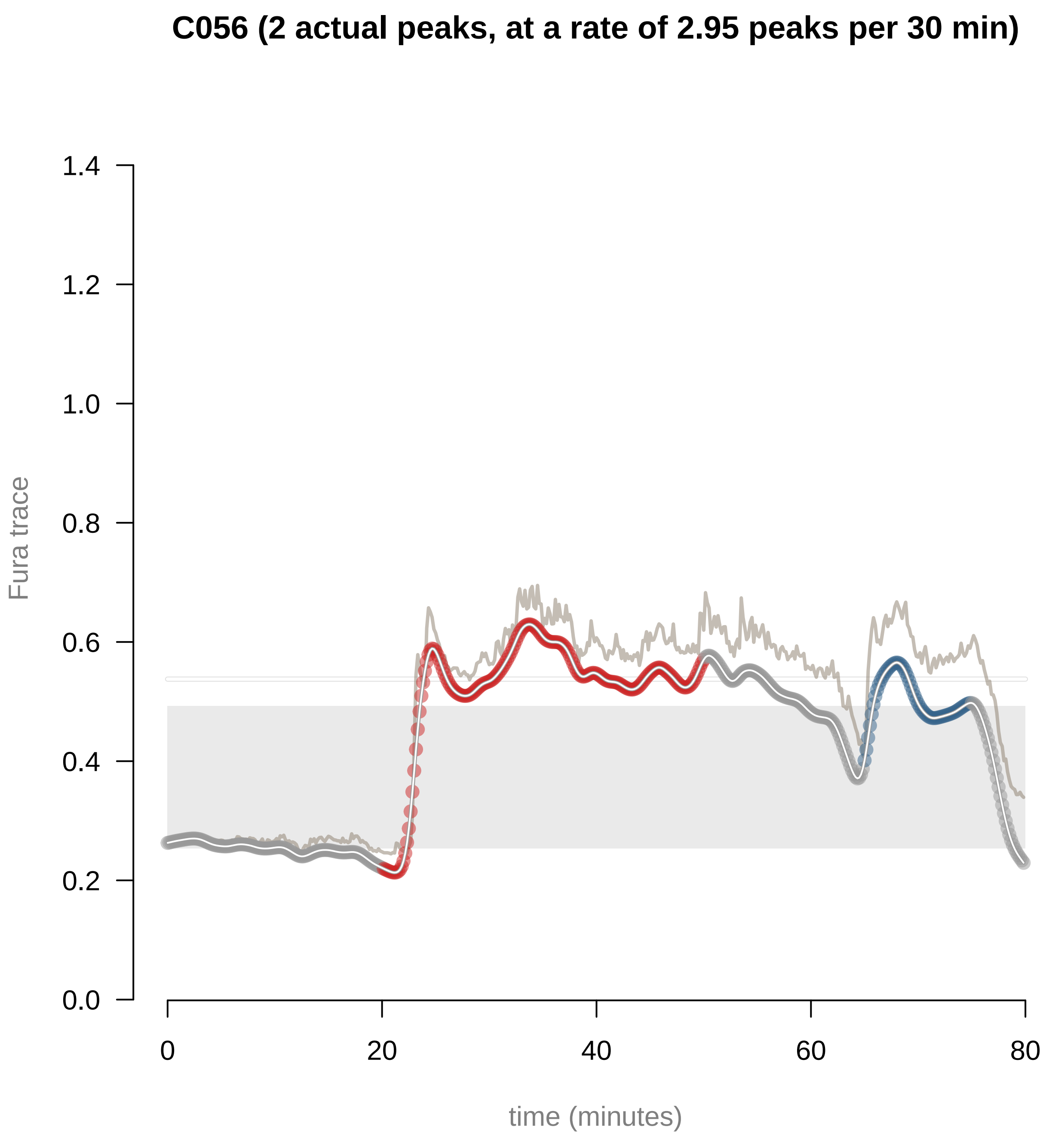

C056 (2 actual peaks, at a rate of 2.95 peaks per 30 min)
Fura trace
1.4
1.2
1.0
0.8
0.6
0.4
0.2
0.0
0
20
40
60
80
time (minutes)

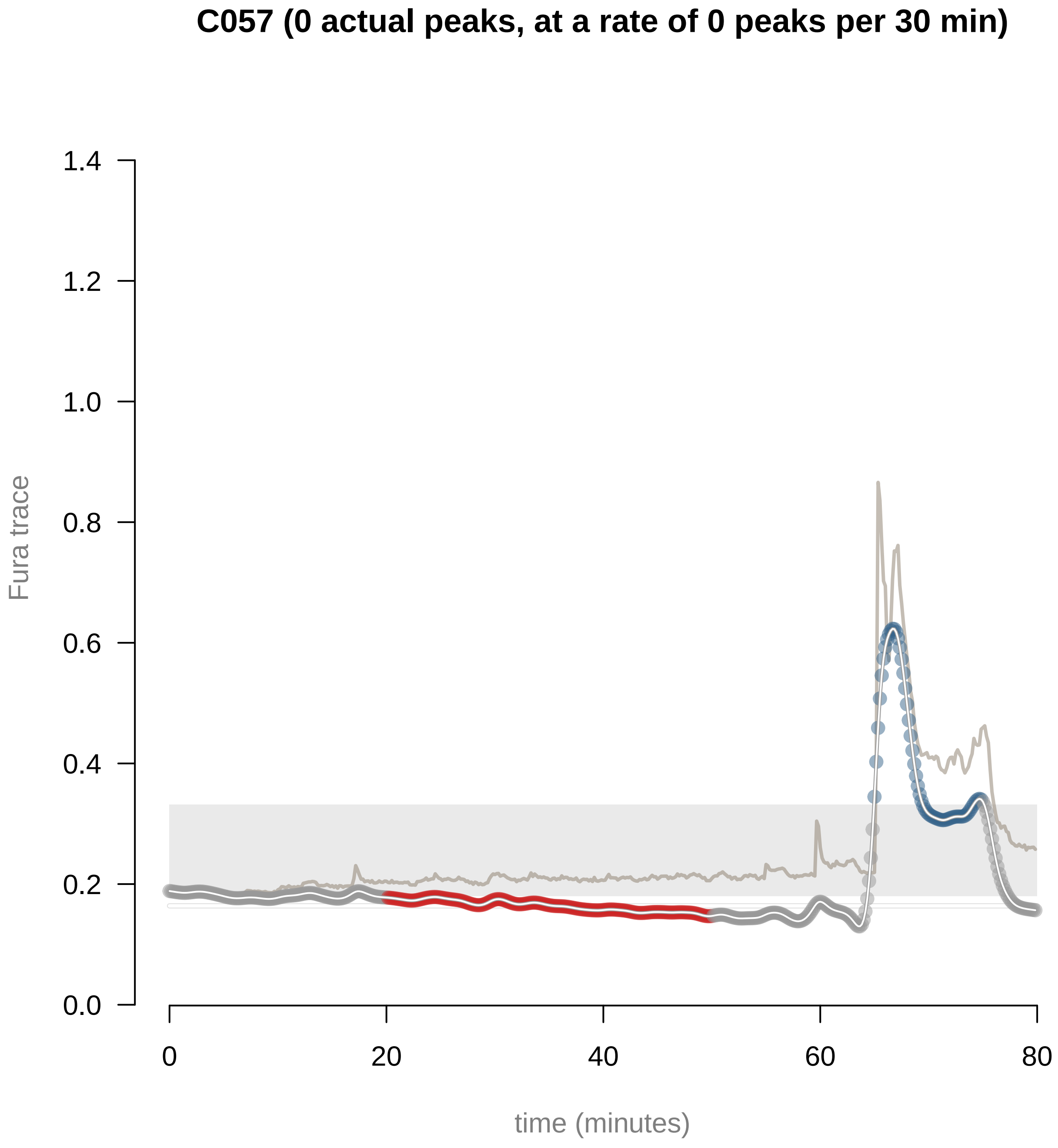
C057 (0 actual peaks, at a rate of 0 peaks per 30 min)
Fura trace
time (minutes)
0.0
0.2
0.4
0.6
0.8
1.0
1.2
1.4
0
20
40
60
80

C058 (3 actual peaks, at a rate of 3.03 peaks per 30 min)

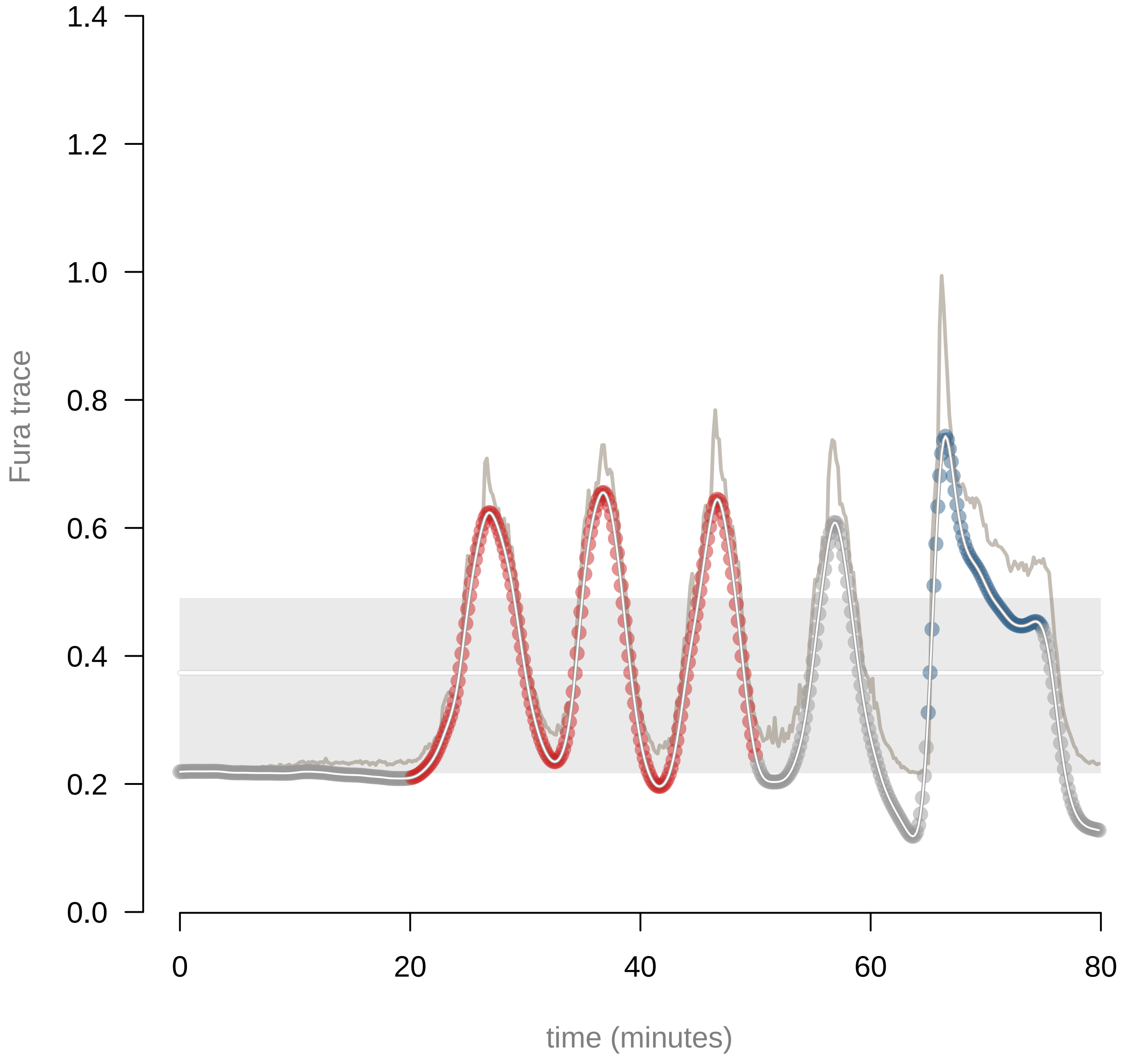

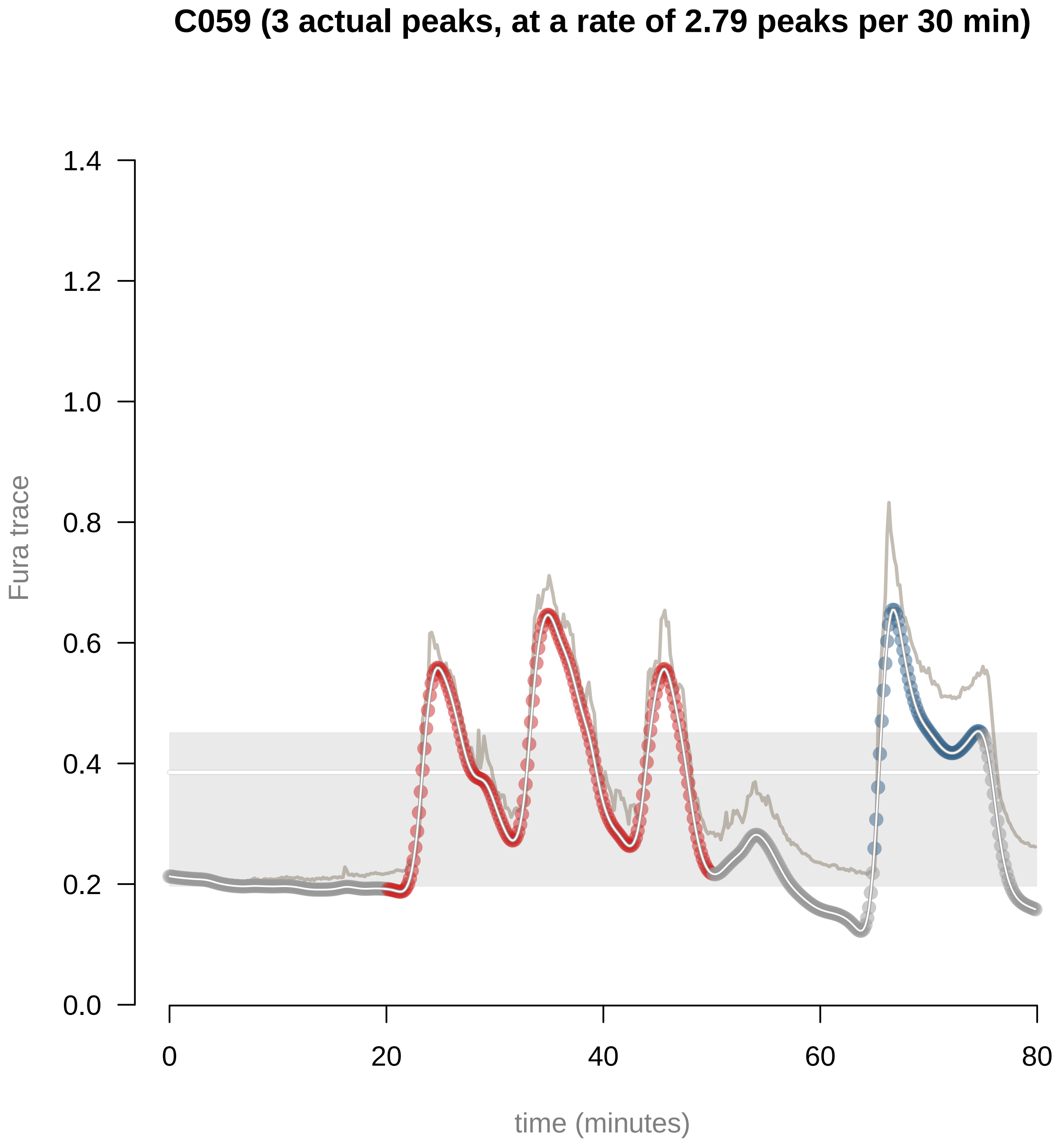
C059 (3 actual peaks, at a rate of 2.79 peaks per 30 min)
Fura trace
0.0
0.2
0.4
0.6
0.8
1.0
1.2
1.4
0
20
40
60
80
time (minutes)

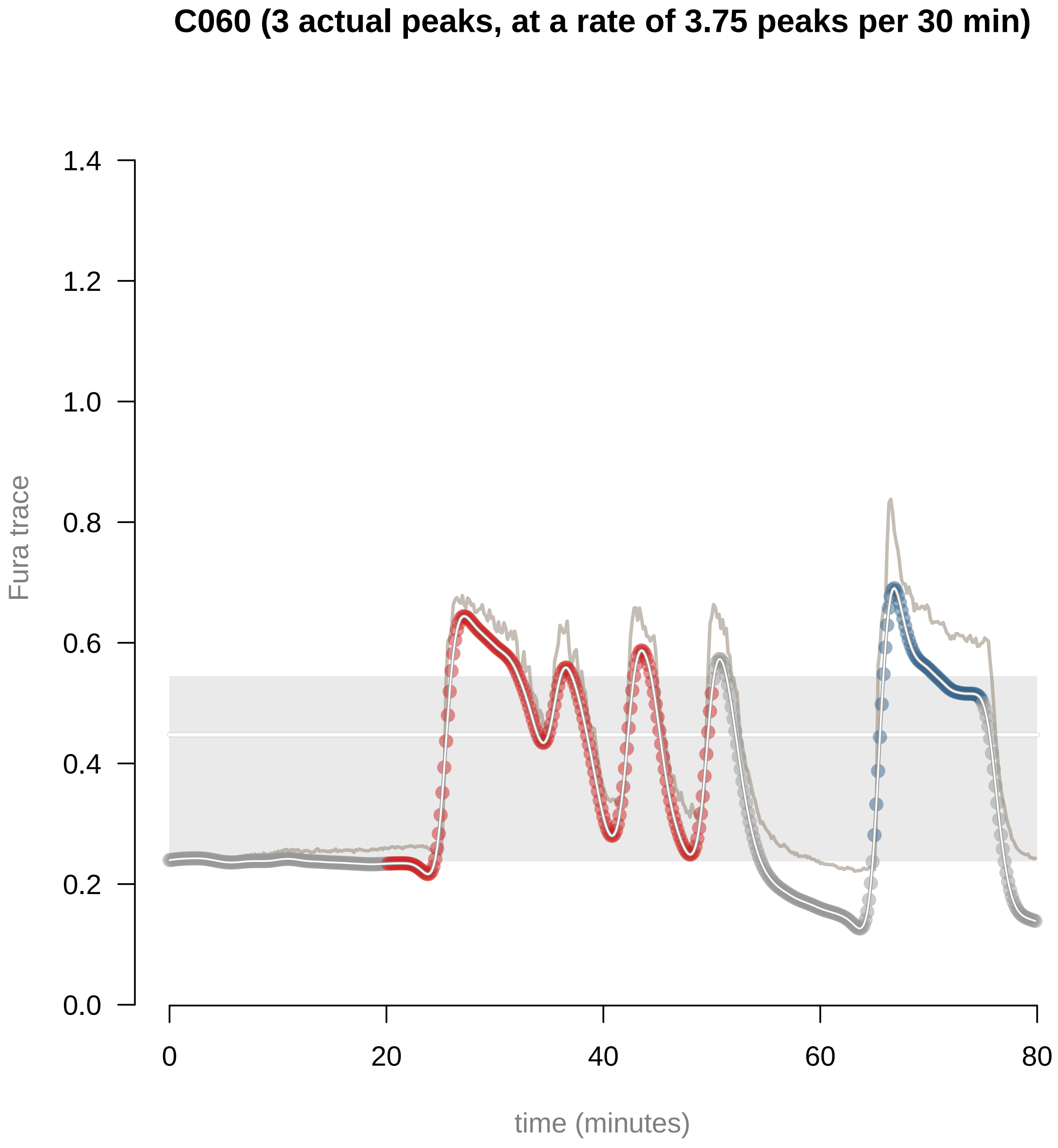
C060 (3 actual peaks, at a rate of 3.75 peaks per 30 min)
Fura trace
time (minutes)
0.0
0.2
0.4
0.6
0.8
1.0
1.2
1.4
0
20
40
60
80

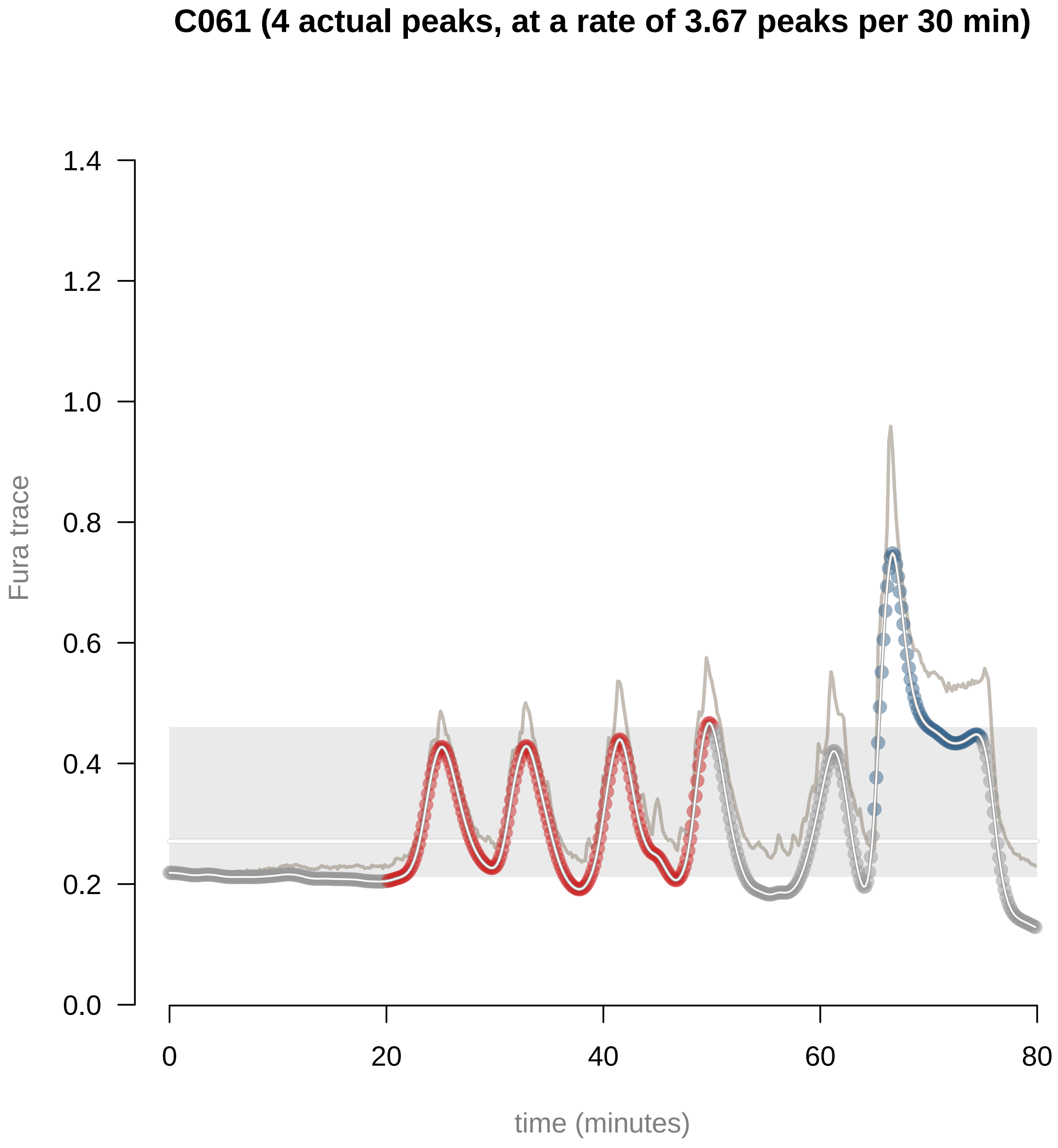

C061 (4 actual peaks, at a rate of 3.67 peaks per 30 min)
Fura trace
1.4
1.2
1.0
0.8
0.6
0.4
0.2
0.0
0
20
40
60
80
time (minutes)

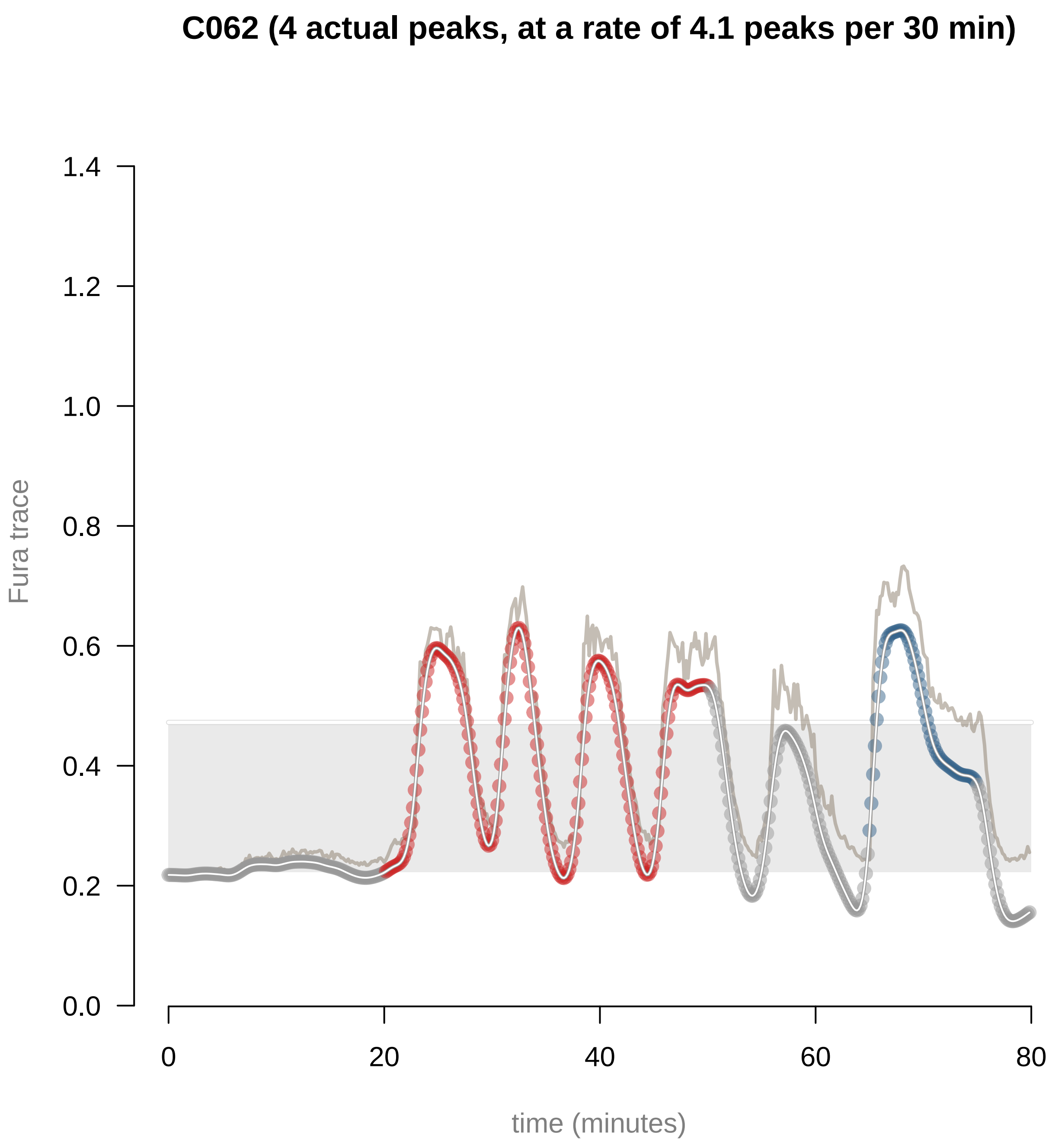
C062 (4 actual peaks, at a rate of 4.1 peaks per 30 min)
Fura trace
1.4
1.2
1.0
0.8
0.6
0.4
0.2
0.0
0
20
40
60
80
time (minutes)

C063 (3 actual peaks, at a rate of 2.55 peaks per 30 min)

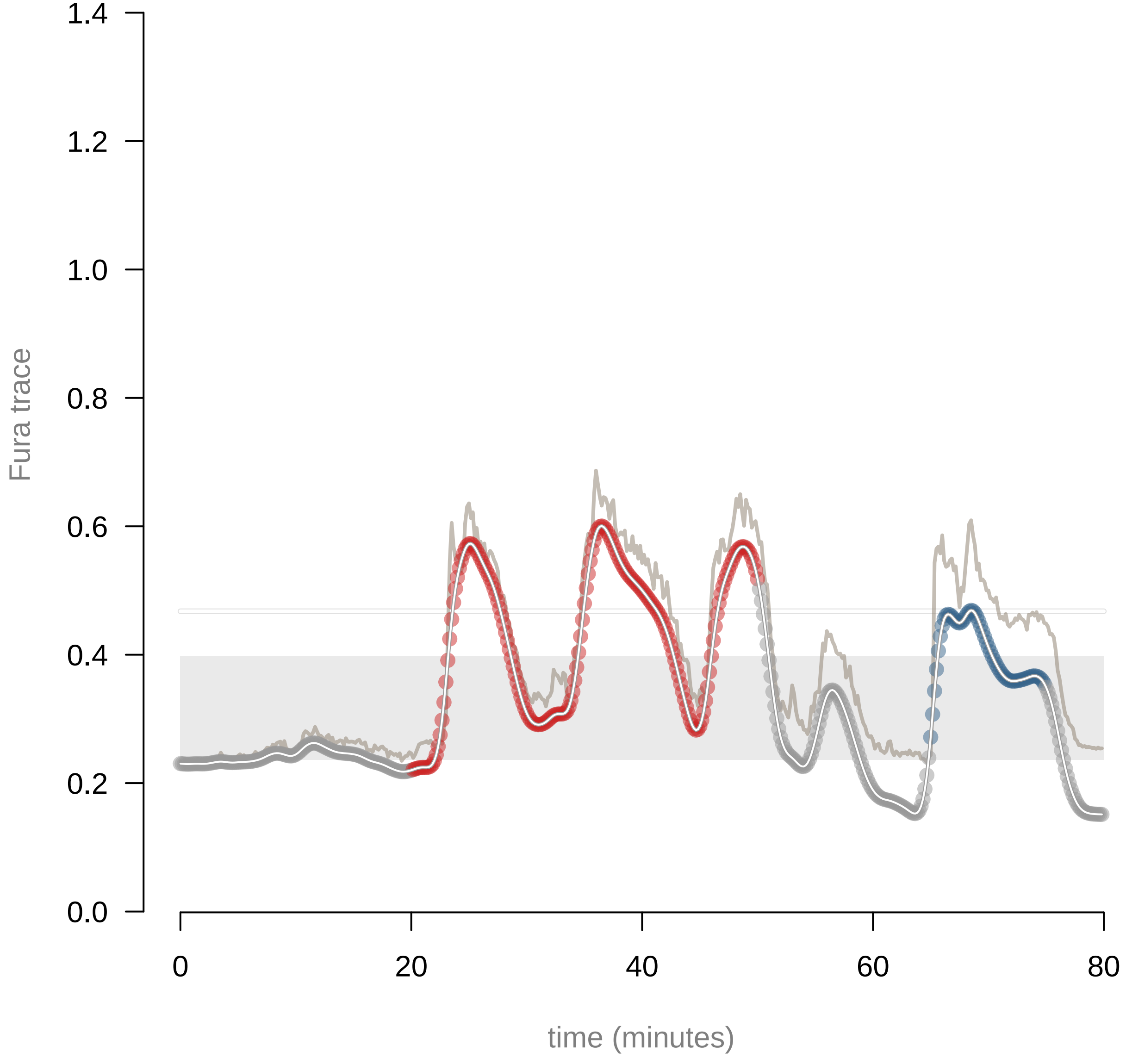

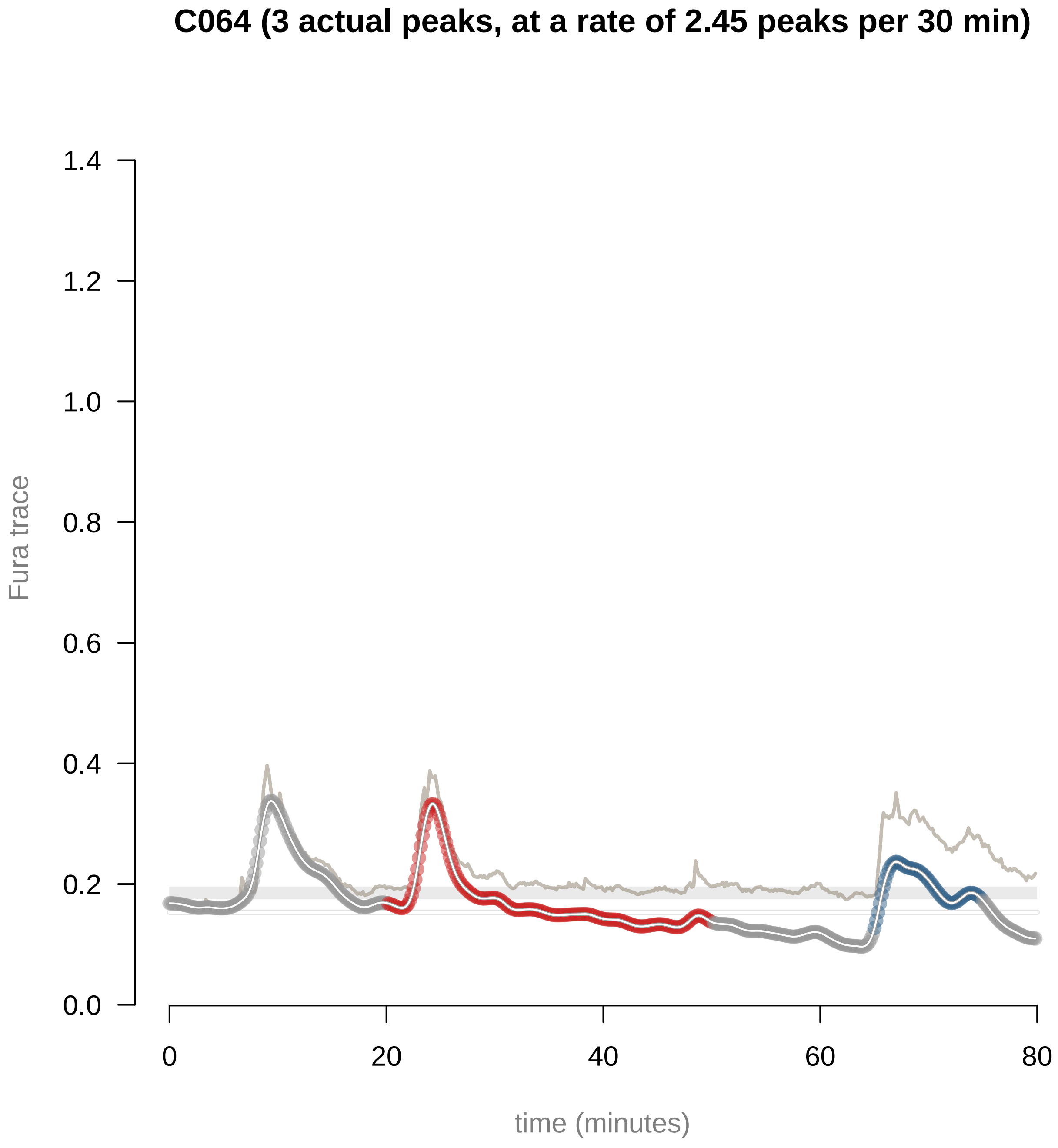

C064 (3 actual peaks, at a rate of 2.45 peaks per 30 min)
Fura trace
1.4
1.2
1.0
0.8
0.6
0.4
0.2
0.0
0
20
40
60
80
time (minutes)

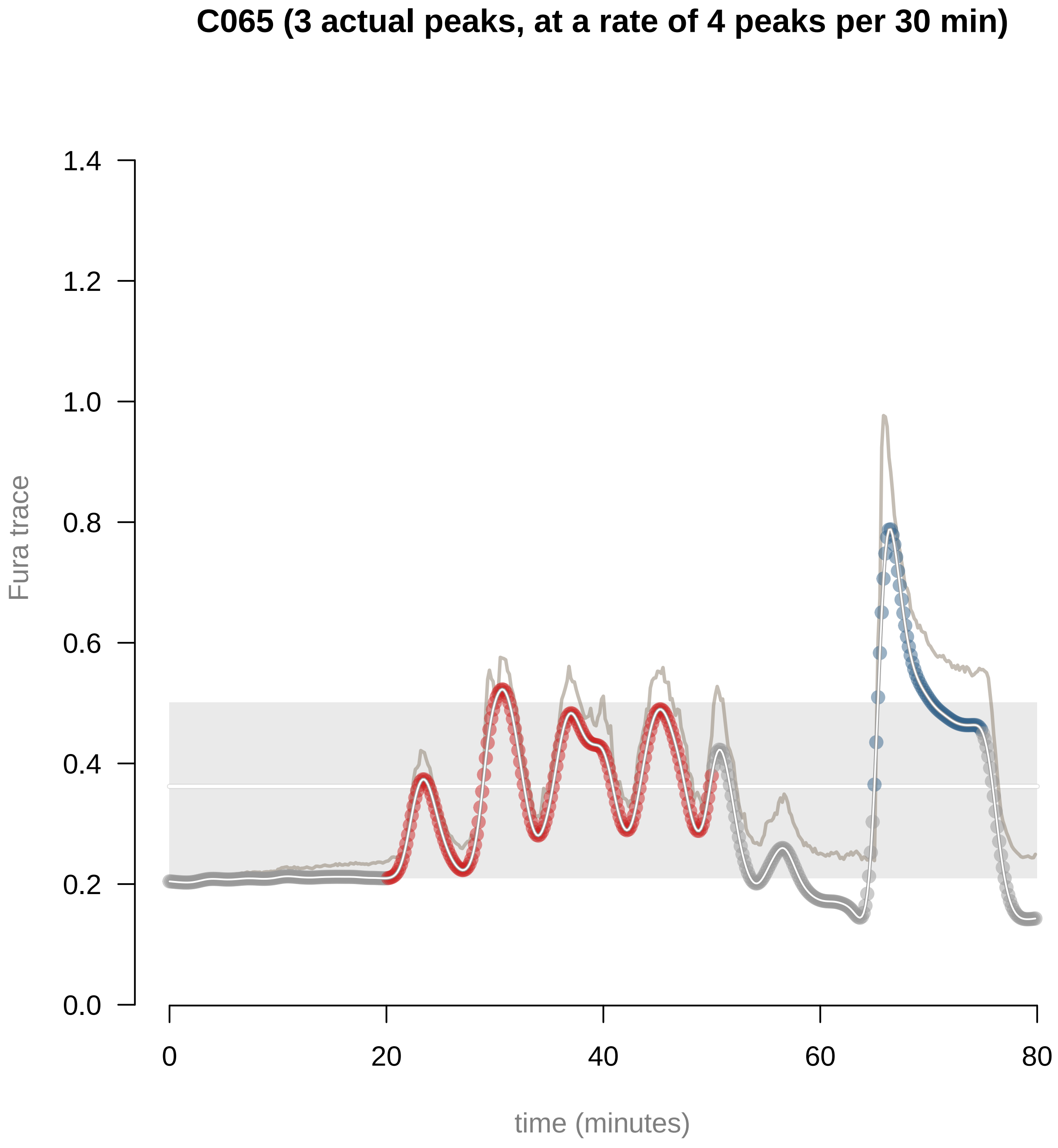

C065 (3 actual peaks, at a rate of 4 peaks per 30 min)
Fura trace
1.4
1.2
1.0
0.8
0.6
0.4
0.2
0.0
0
20
40
60
80
time (minutes)

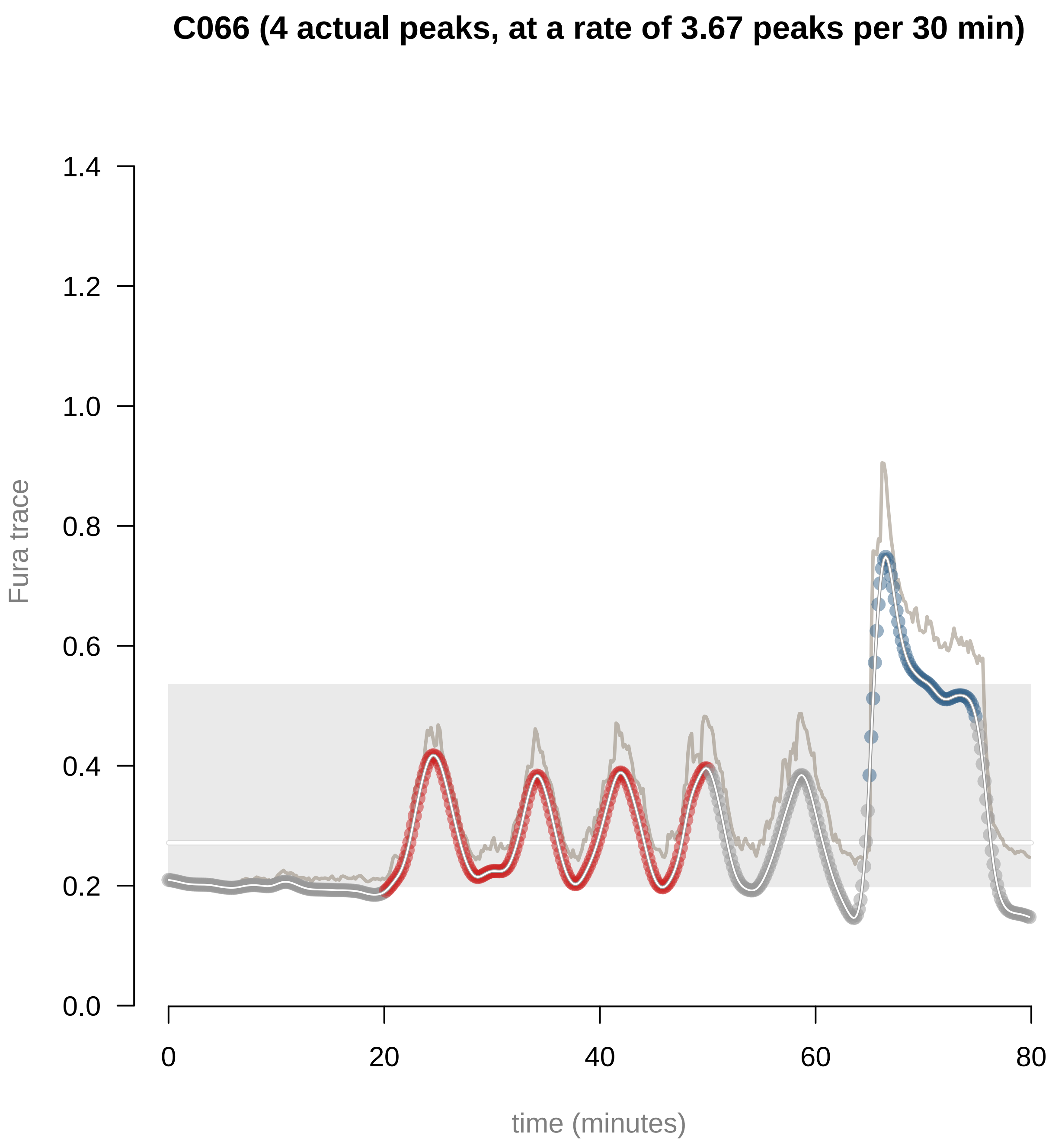

C066 (4 actual peaks, at a rate of 3.67 peaks per 30 min)
Fura trace
time (minutes)
0.0
0.2
0.4
0.6
0.8
1.0
1.2
1.4
0
20
40
60
80

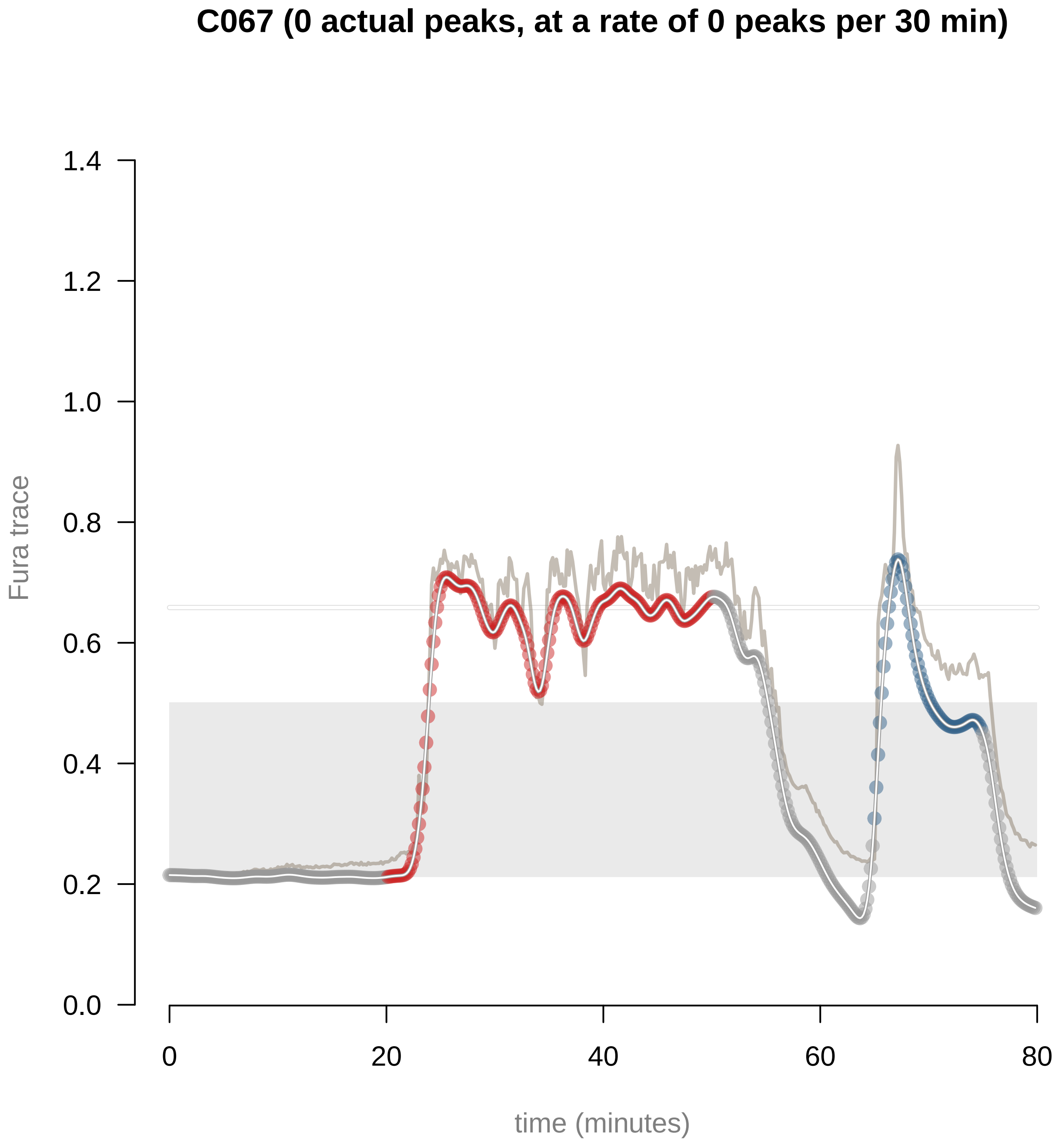

C067 (0 actual peaks, at a rate of 0 peaks per 30 min)
Fura trace
1.4
1.2
1.0
0.8
0.6
0.4
0.2
0.0
0
20
40
60
80
time (minutes)

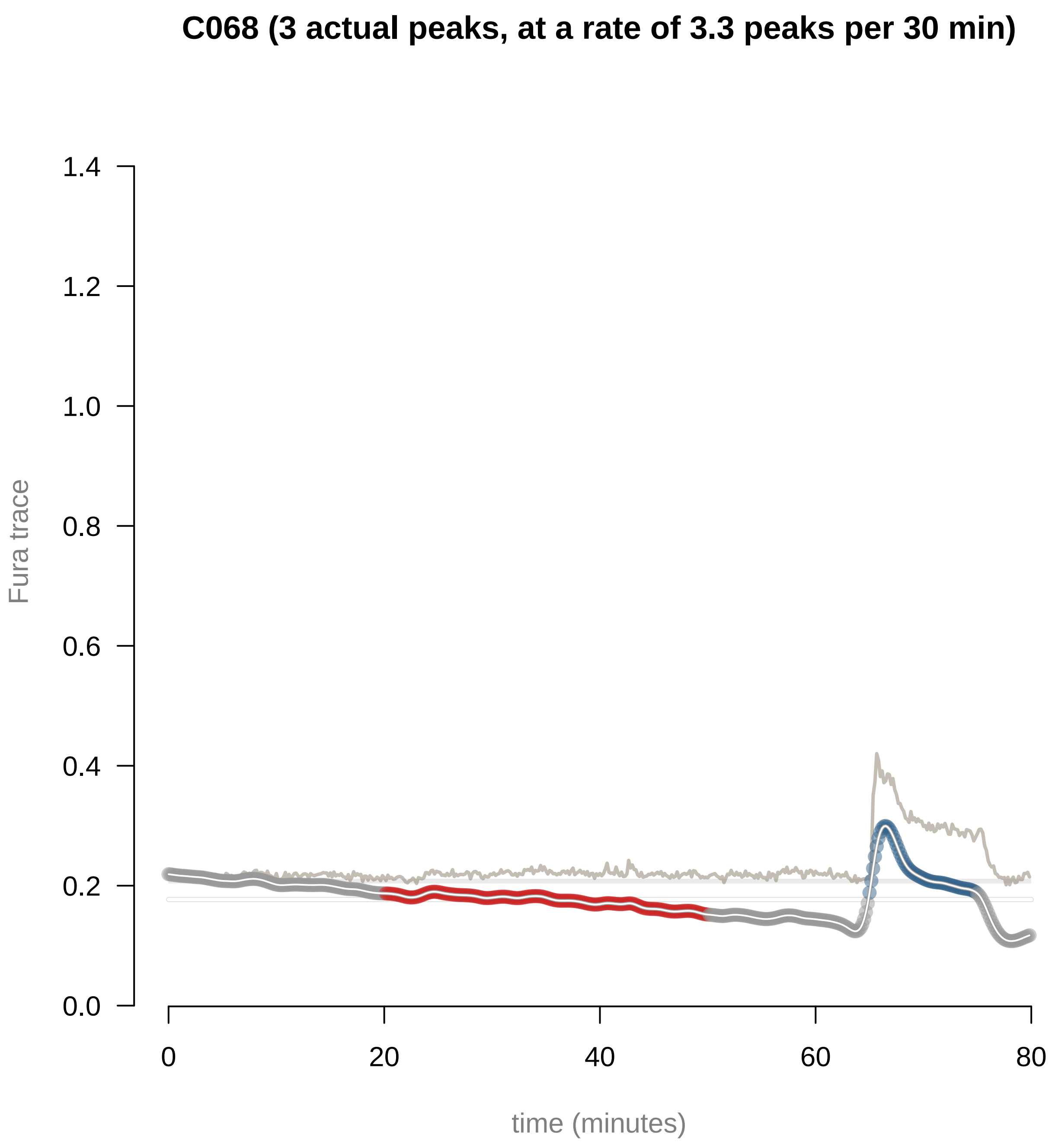
C068 (3 actual peaks, at a rate of 3.3 peaks per 30 min)
Fura trace
0.0
0.2
0.4
0.6
0.8
1.0
1.2
1.4
0
20
40
60
80
time (minutes)

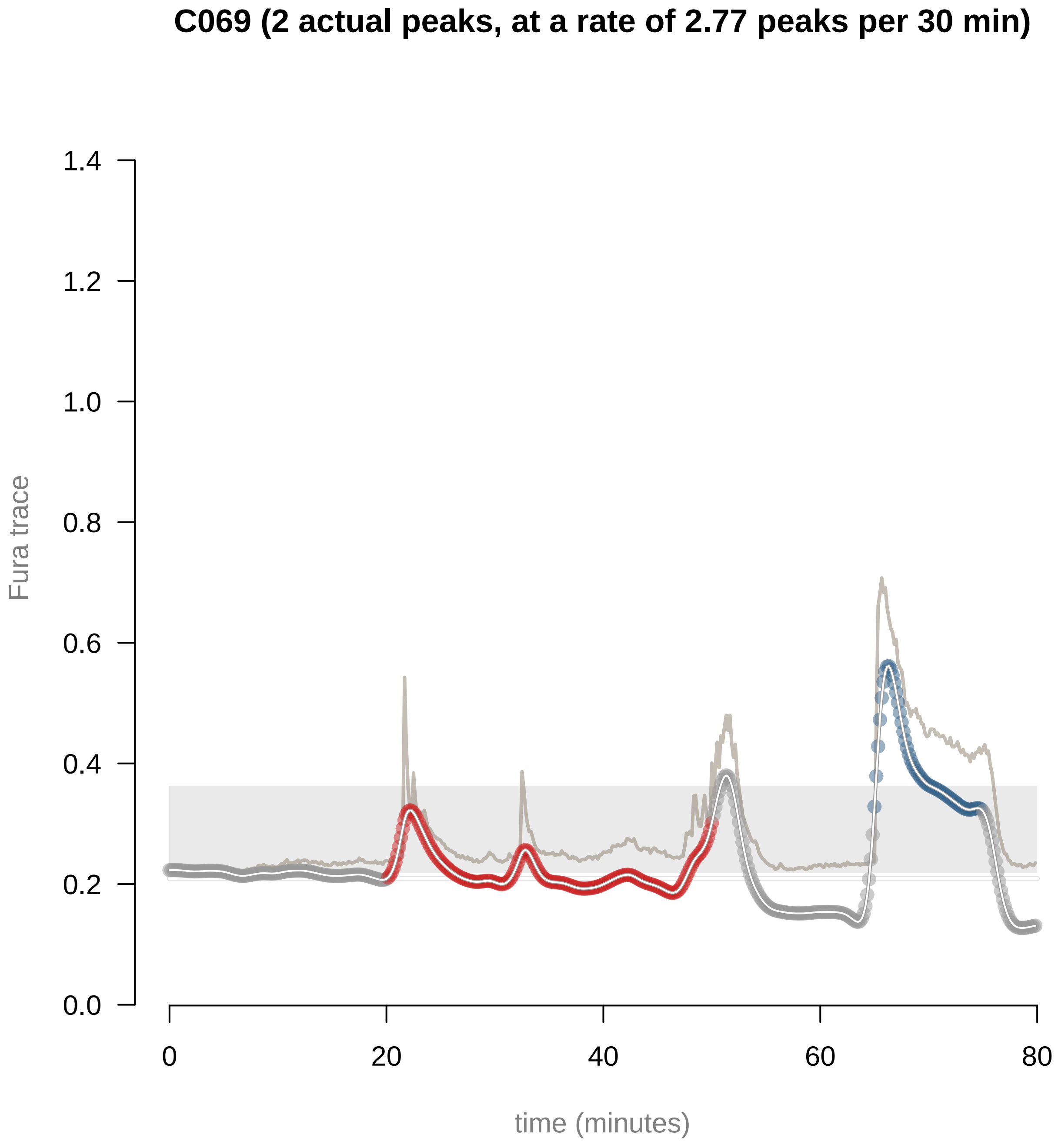
C069 (2 actual peaks, at a rate of 2.77 peaks per 30 min)
Fura trace
1.4
1.2
1.0
0.8
0.6
0.4
0.2
0.0
0
20
40
60
80
time (minutes)

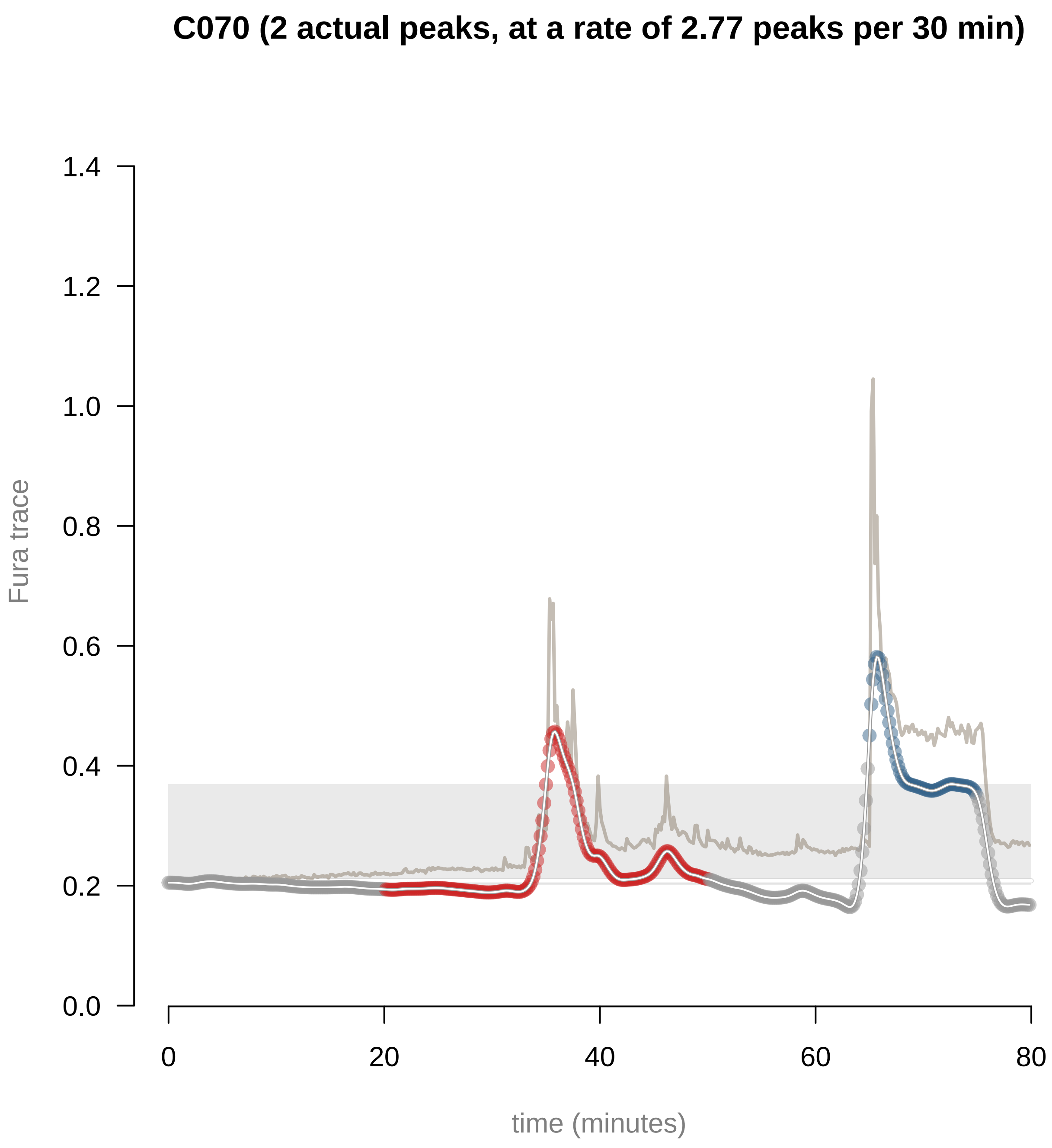

C070 (2 actual peaks, at a rate of 2.77 peaks per 30 min)
Fura trace
0.0
0.2
0.4
0.6
0.8
1.0
1.2
1.4
0
20
40
60
80
time (minutes)

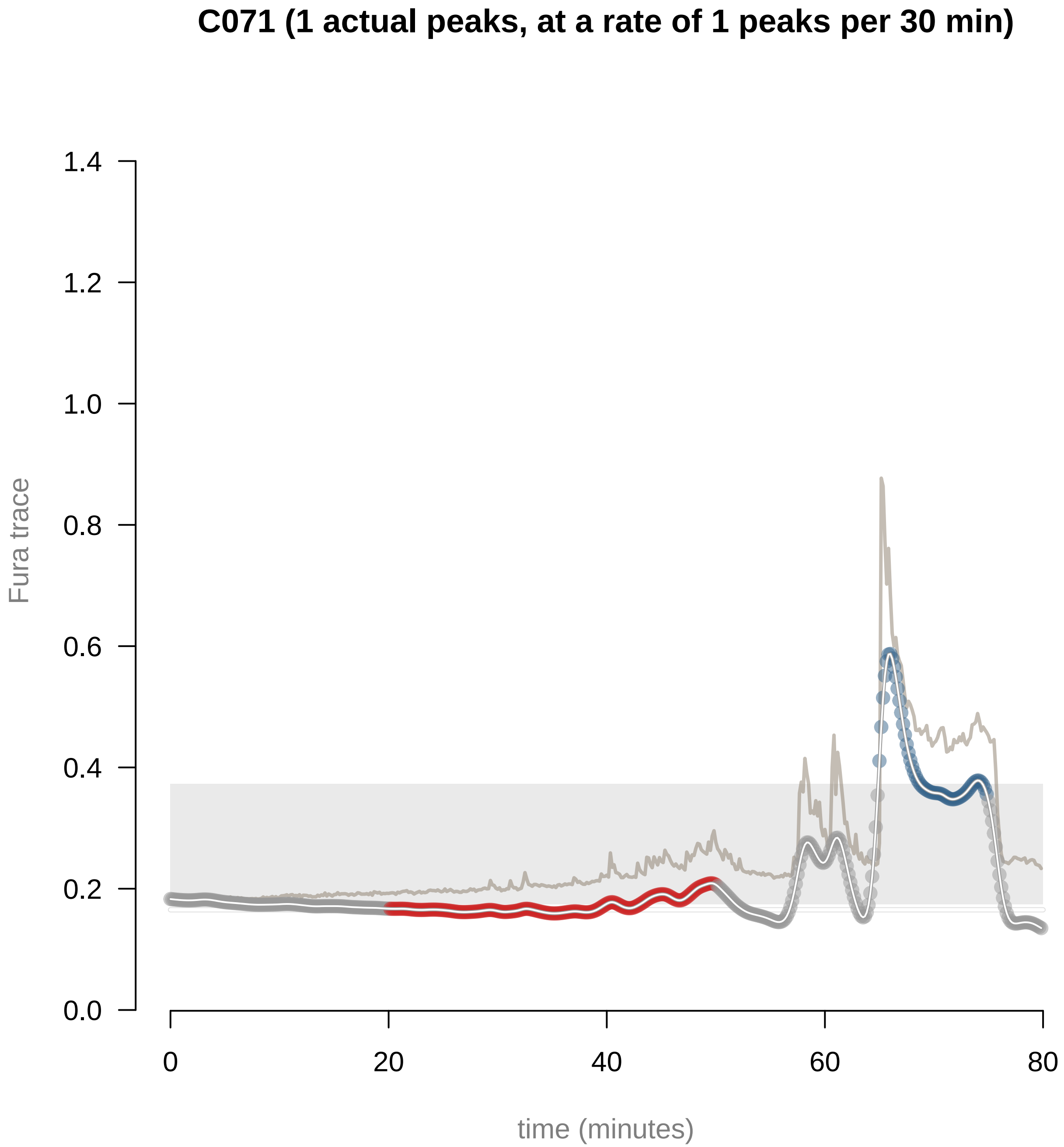
C071 (1 actual peaks, at a rate of 1 peaks per 30 min)
Fura trace
0.0
0.2
0.4
0.6
0.8
1.0
1.2
1.4
0
20
40
60
80
time (minutes)

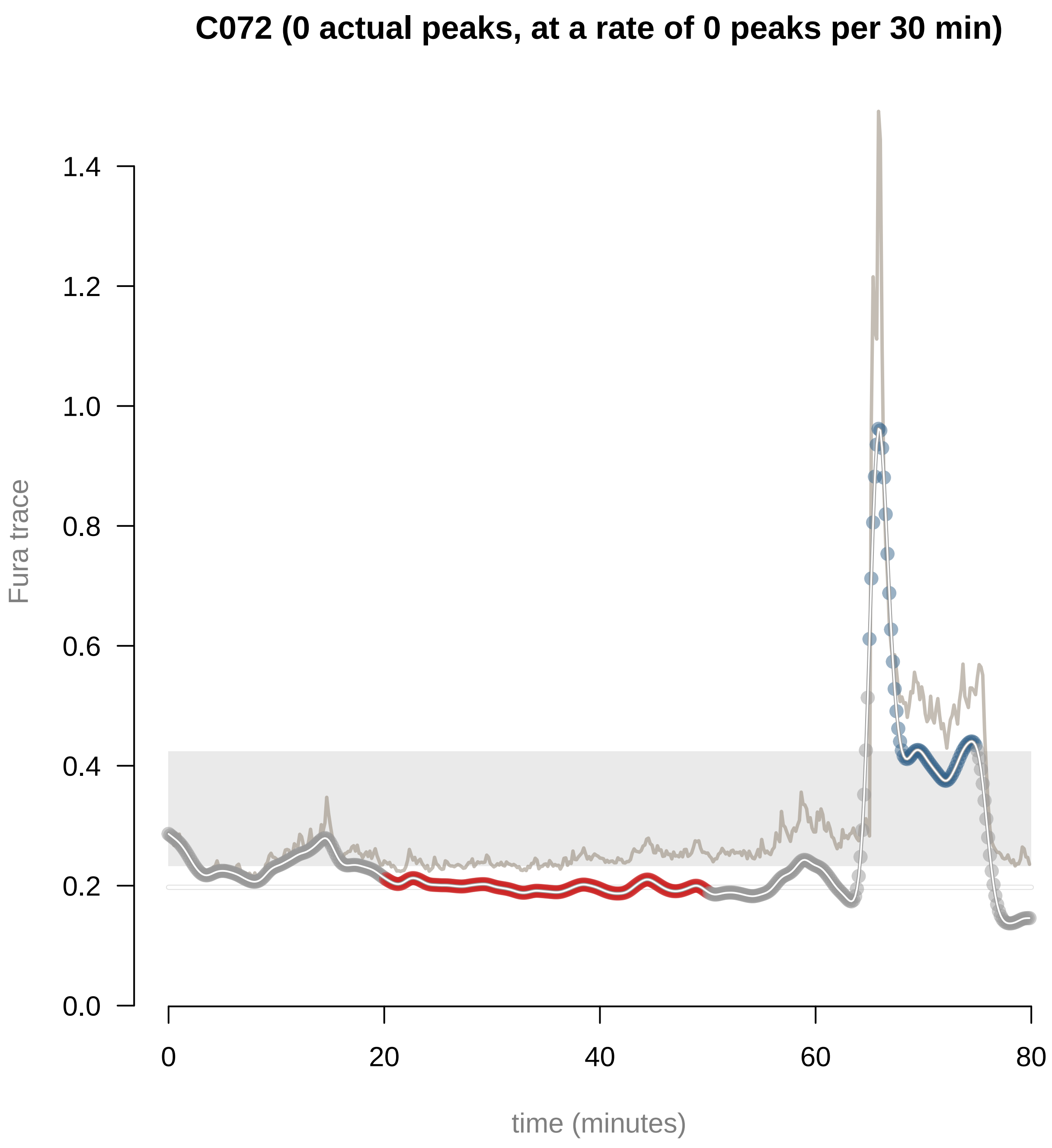
C072 (0 actual peaks, at a rate of 0 peaks per 30 min)
Fura trace
time (minutes)
0.0
0.2
0.4
0.6
0.8
1.0
1.2
1.4
0
20
40
60
80

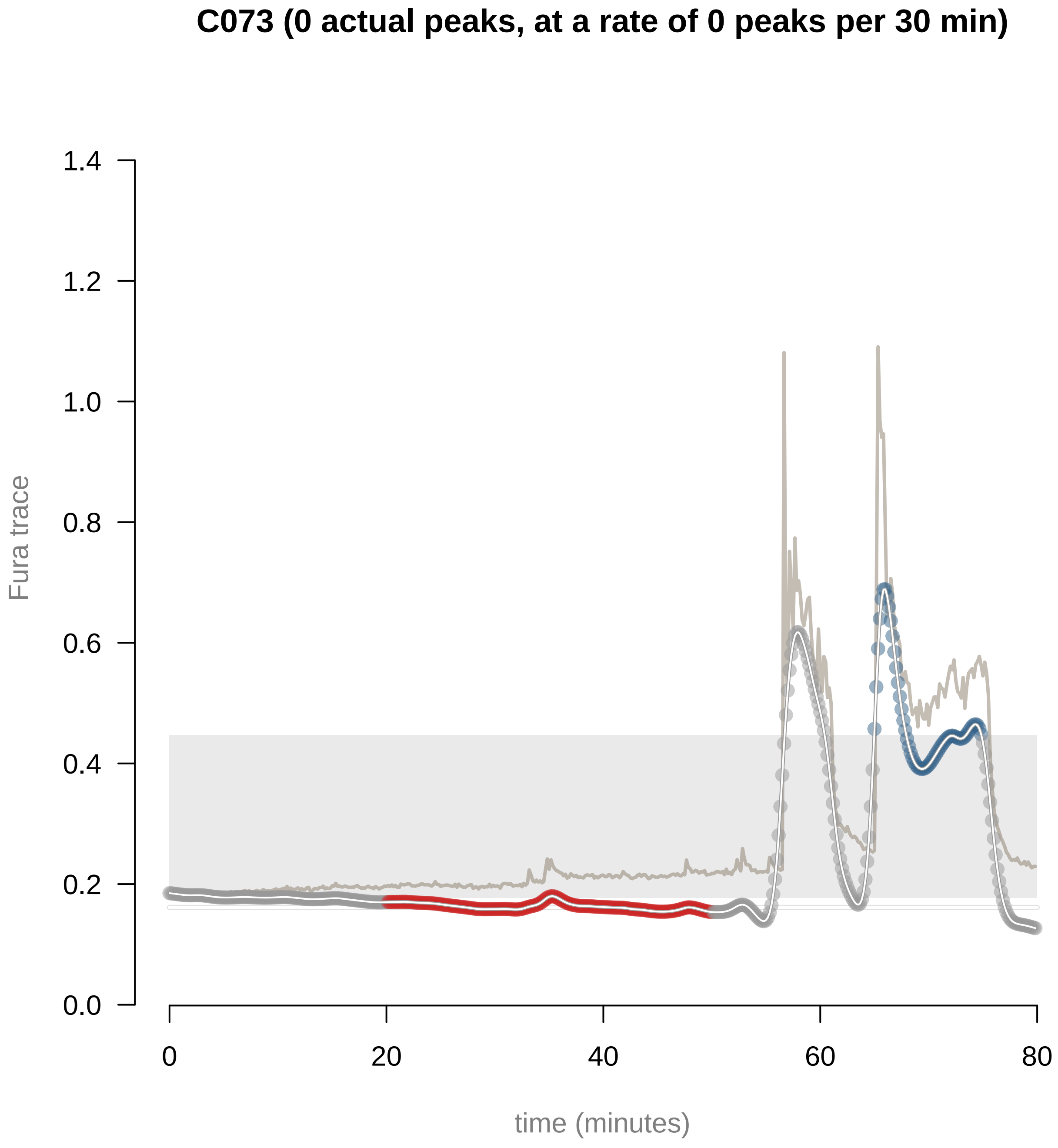
C073 (0 actual peaks, at a rate of 0 peaks per 30 min)
Fura trace
time (minutes)
0.0
0.2
0.4
0.6
0.8
1.0
1.2
1.4
0
20
40
60
80

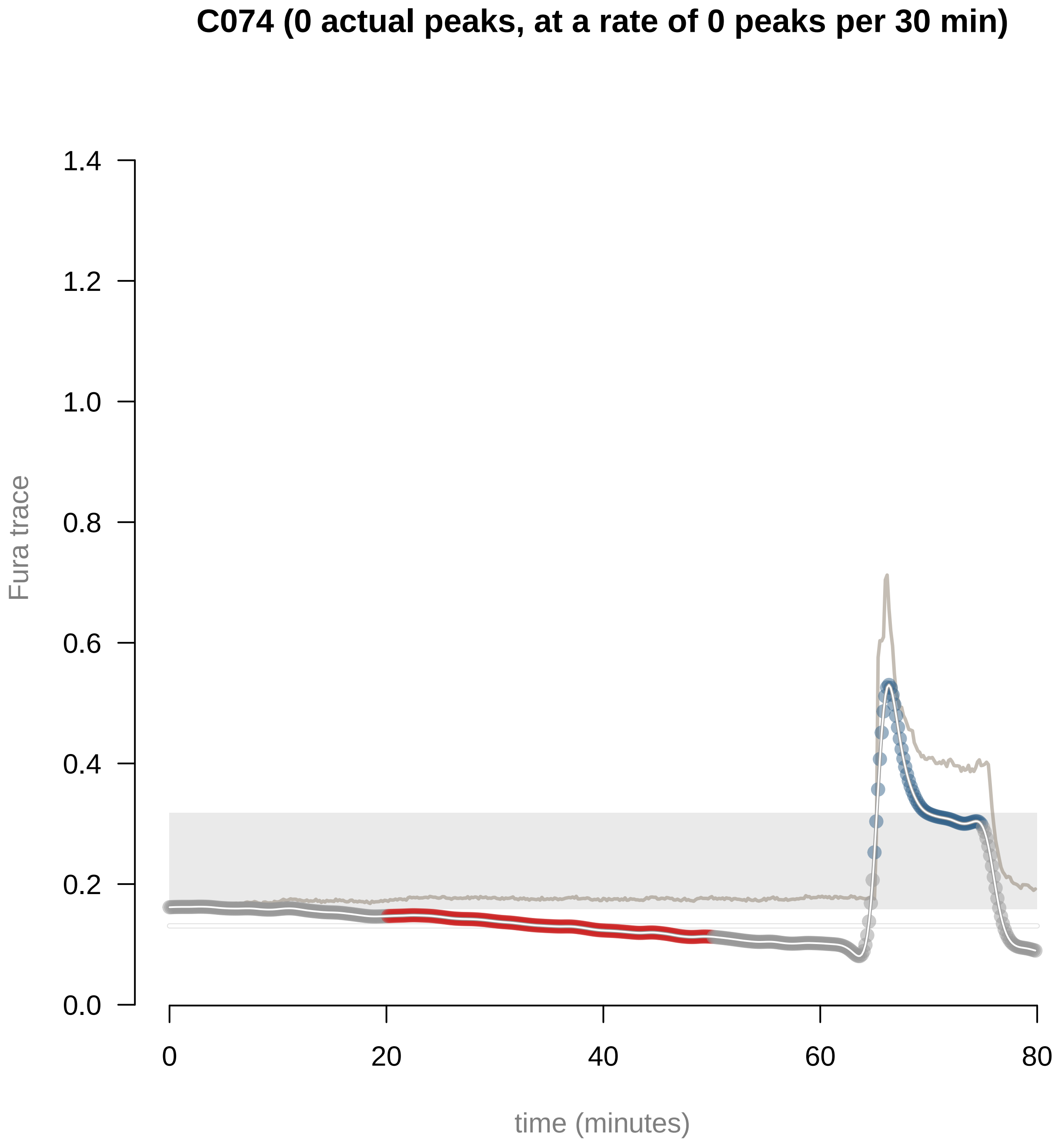
C074 (0 actual peaks, at a rate of 0 peaks per 30 min)
Fura trace
1.4
1.2
1.0
0.8
0.6
0.4
0.2
0.0
0
20
40
60
80
time (minutes)

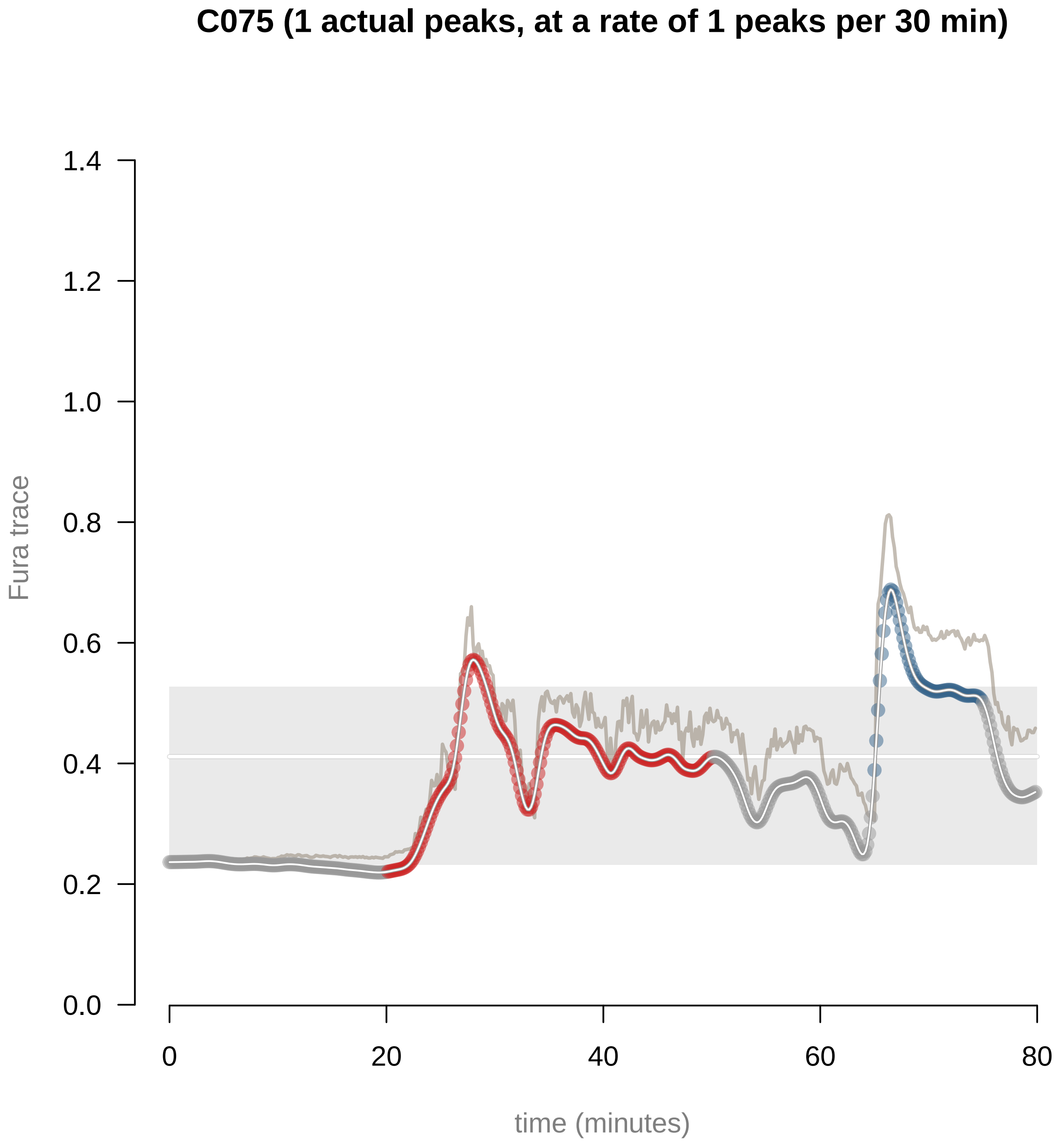

C075 (1 actual peaks, at a rate of 1 peaks per 30 min)
Fura trace
1.4
1.2
1.0
0.8
0.6
0.4
0.2
0.0
0
20
40
60
80
time (minutes)

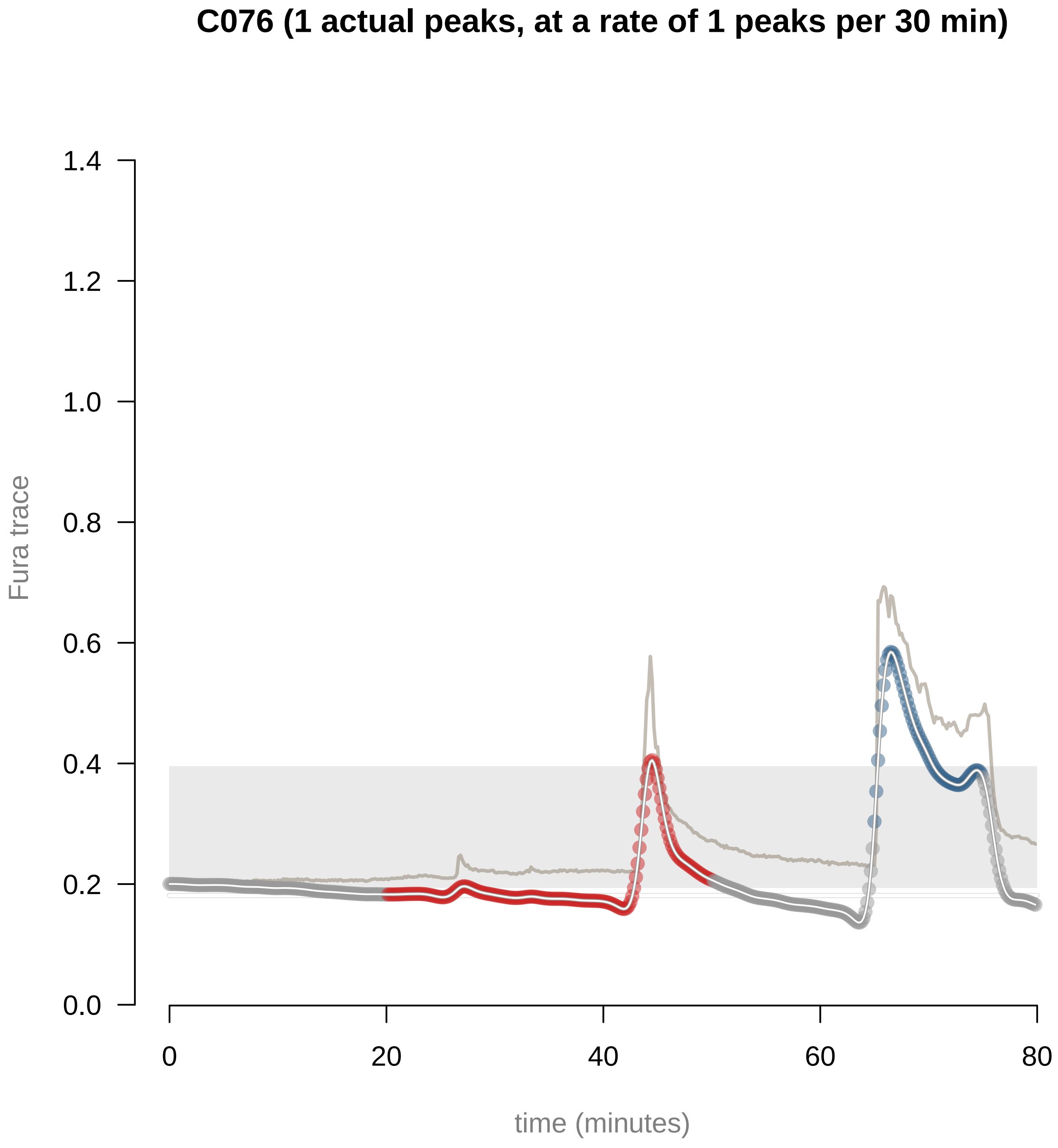
C076 (1 actual peaks, at a rate of 1 peaks per 30 min)
Fura trace
time (minutes)
0.0
0.2
0.4
0.6
0.8
1.0
1.2
1.4
0
20
40
60
80

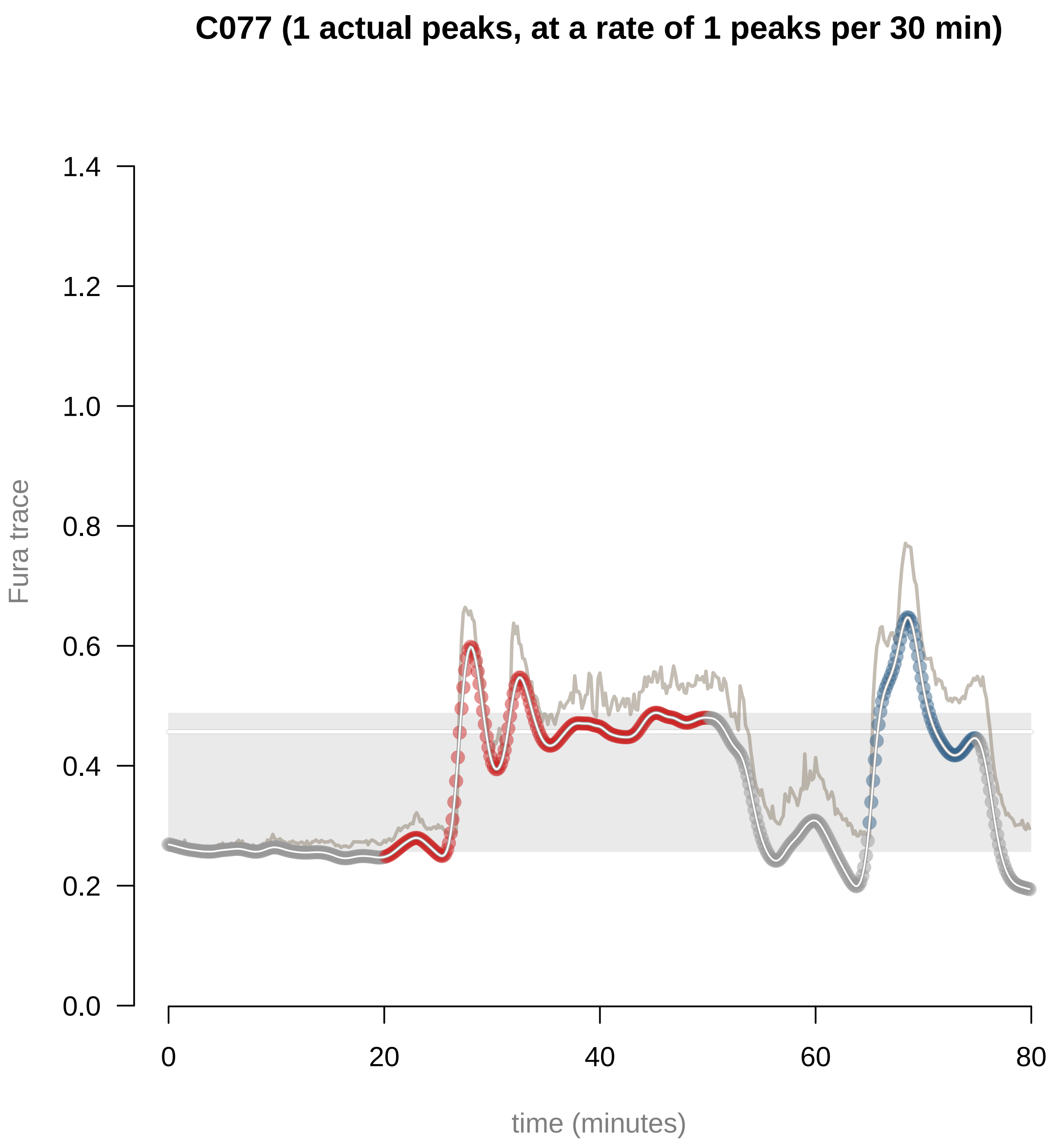
C077 (1 actual peaks, at a rate of 1 peaks per 30 min)
Fura trace
1.4
1.2
1.0
0.8
0.6
0.4
0.2
0.0
0
20
40
60
80
time (minutes)

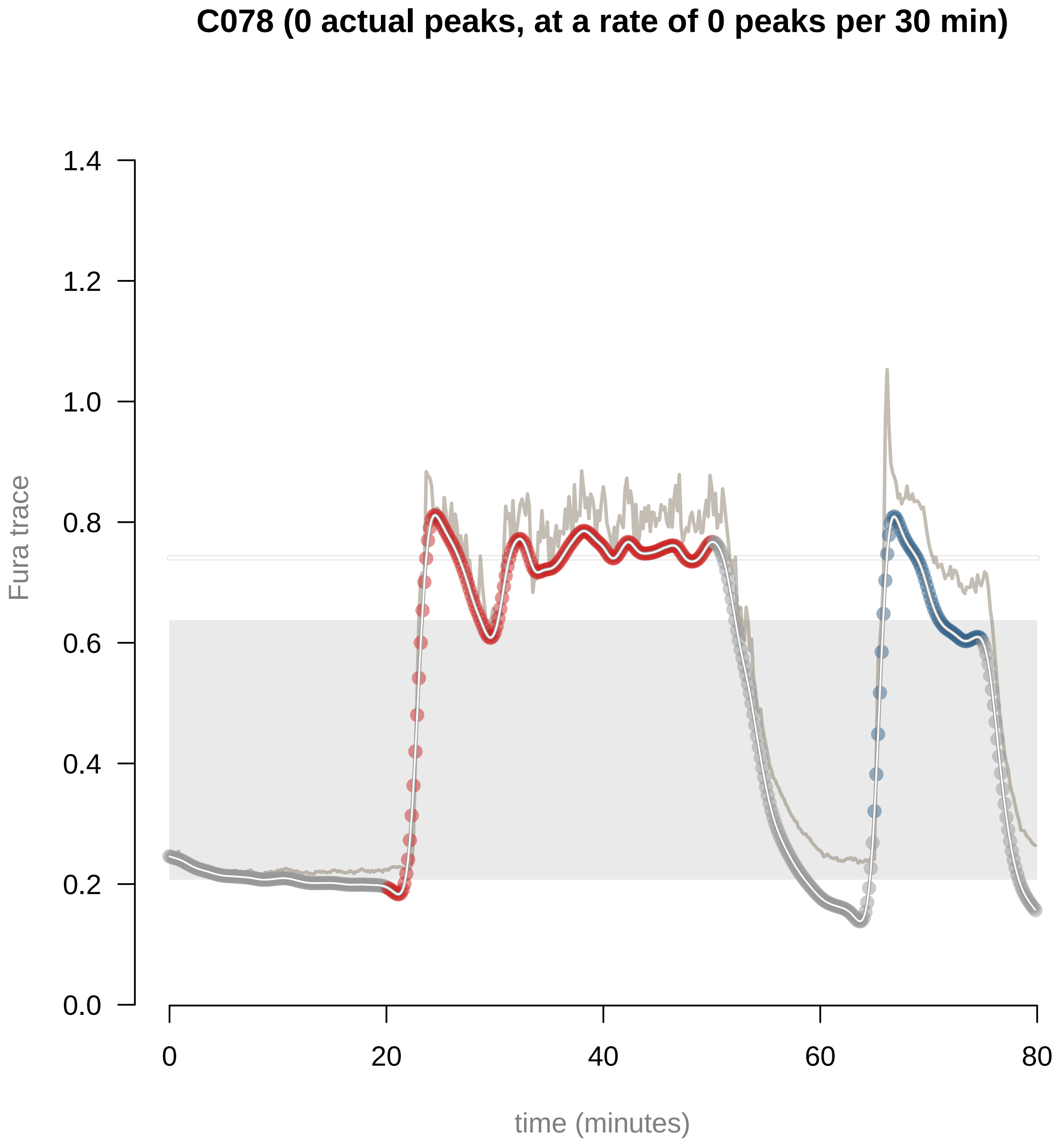

C078 (0 actual peaks, at a rate of 0 peaks per 30 min)
Fura trace
0.0
0.2
0.4
0.6
0.8
1.0
1.2
1.4
0
20
40
60
80
time (minutes)

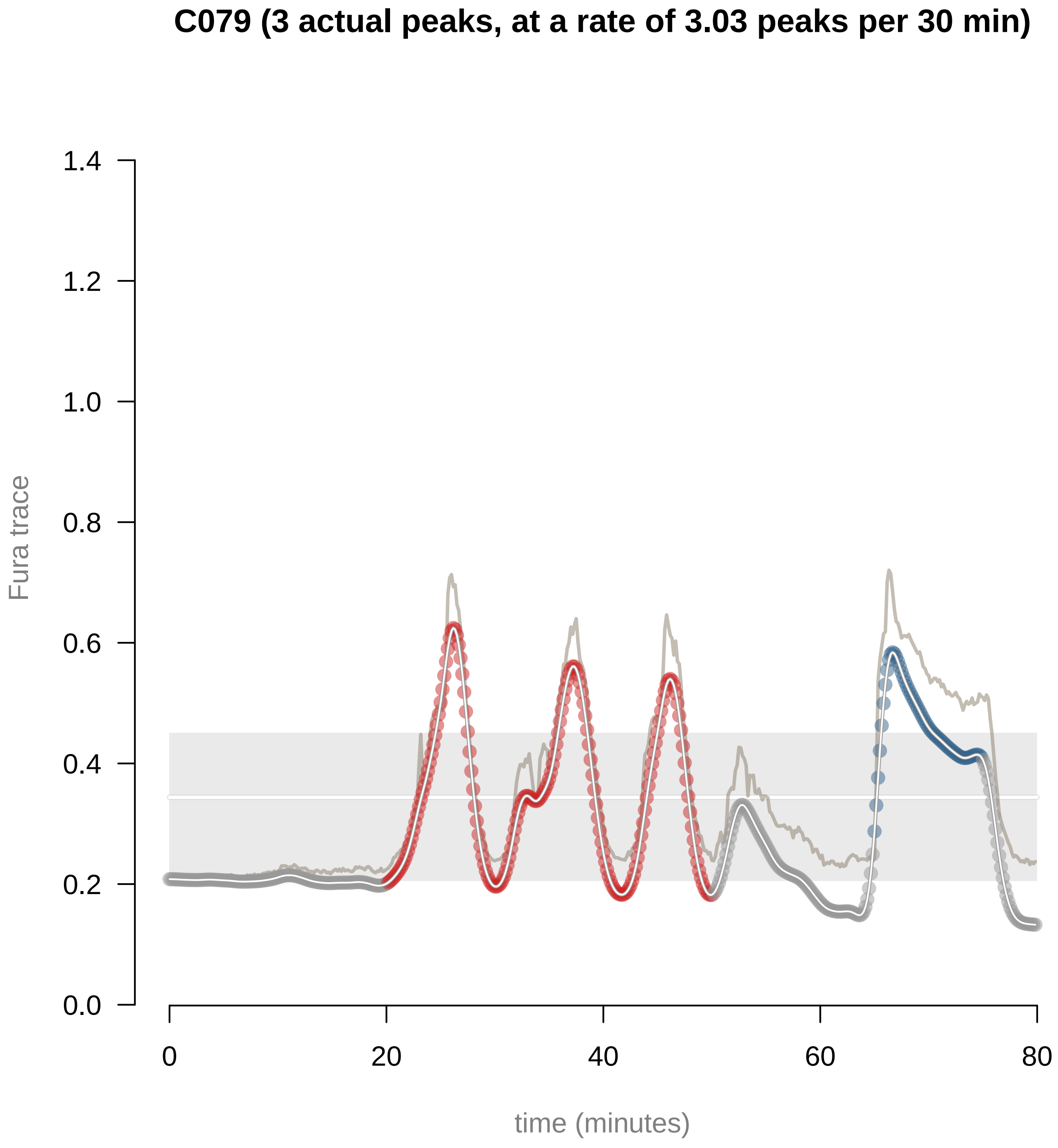
C079 (3 actual peaks, at a rate of 3.03 peaks per 30 min)
Fura trace
1.4
1.2
1.0
0.8
0.6
0.4
0.2
0.0
0
20
40
60
80
time (minutes)

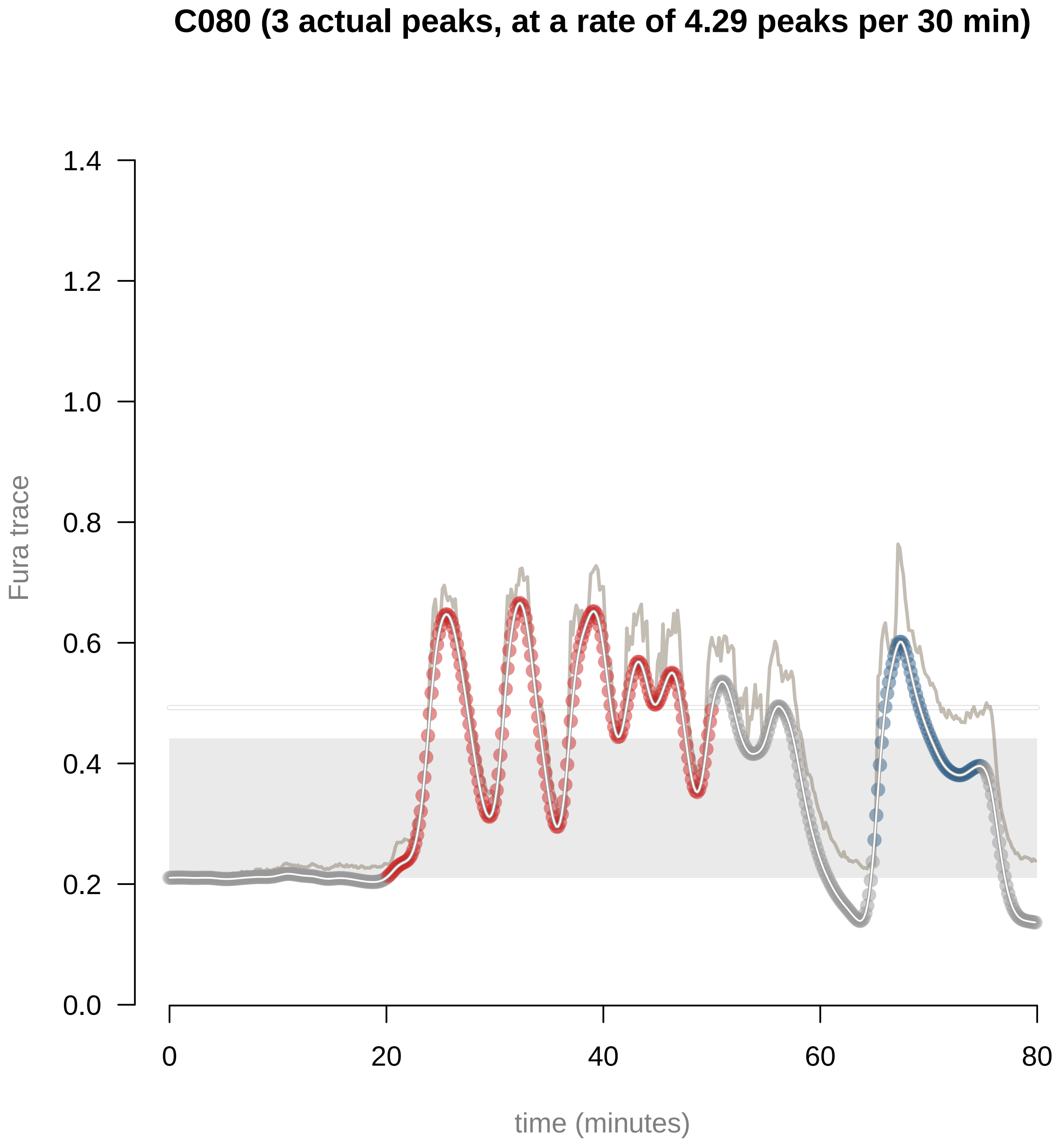

C080 (3 actual peaks, at a rate of 4.29 peaks per 30 min)
Fura trace
time (minutes)
0.0
0.2
0.4
0.6
0.8
1.0
1.2
1.4
0
20
40
60
80

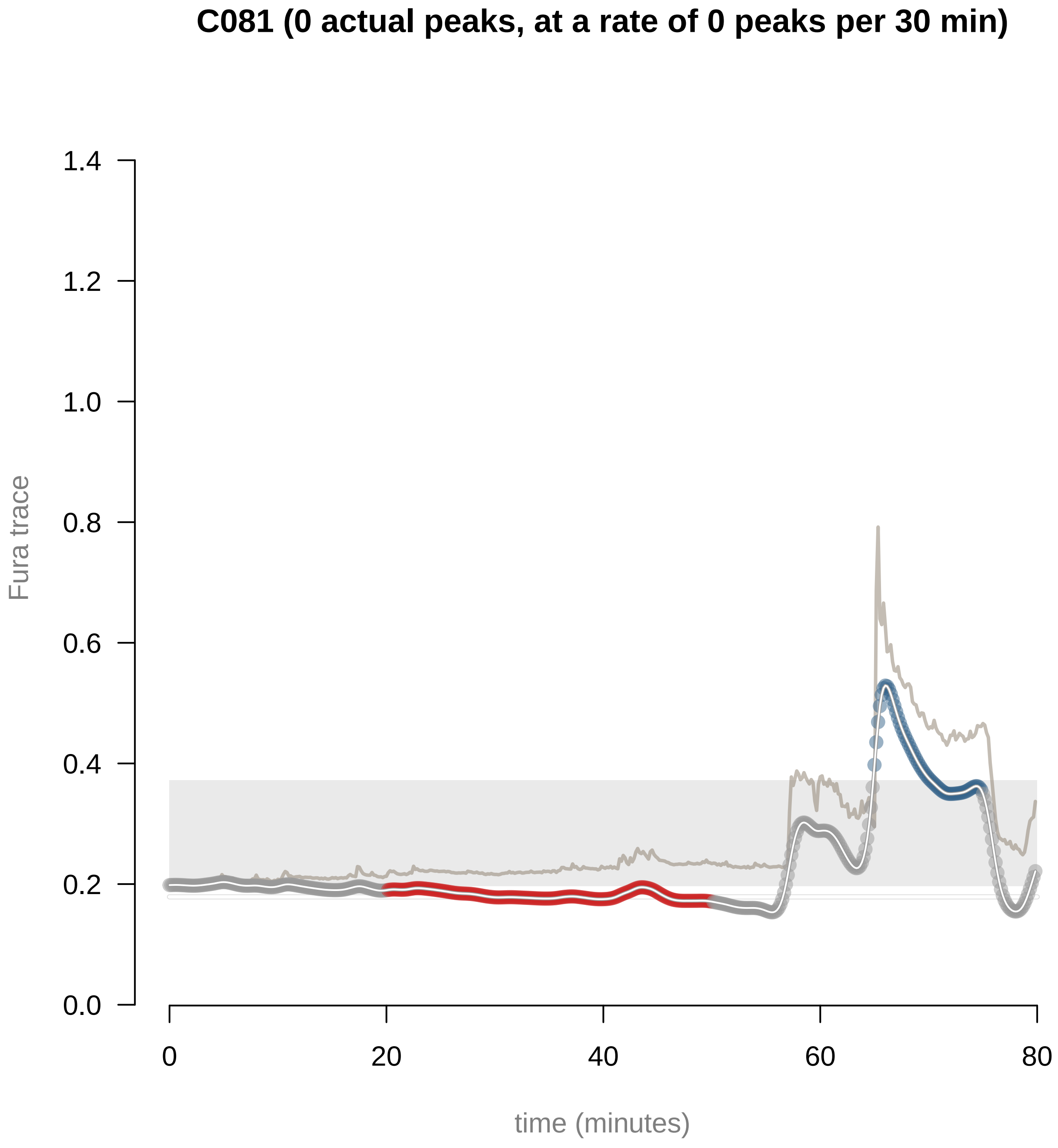
C081 (0 actual peaks, at a rate of 0 peaks per 30 min)
Fura trace
0.0
0.2
0.4
0.6
0.8
1.0
1.2
1.4
0
20
40
60
80
time (minutes)

C082 (3 actual peaks, at a rate of 2.63 peaks per 30 min)

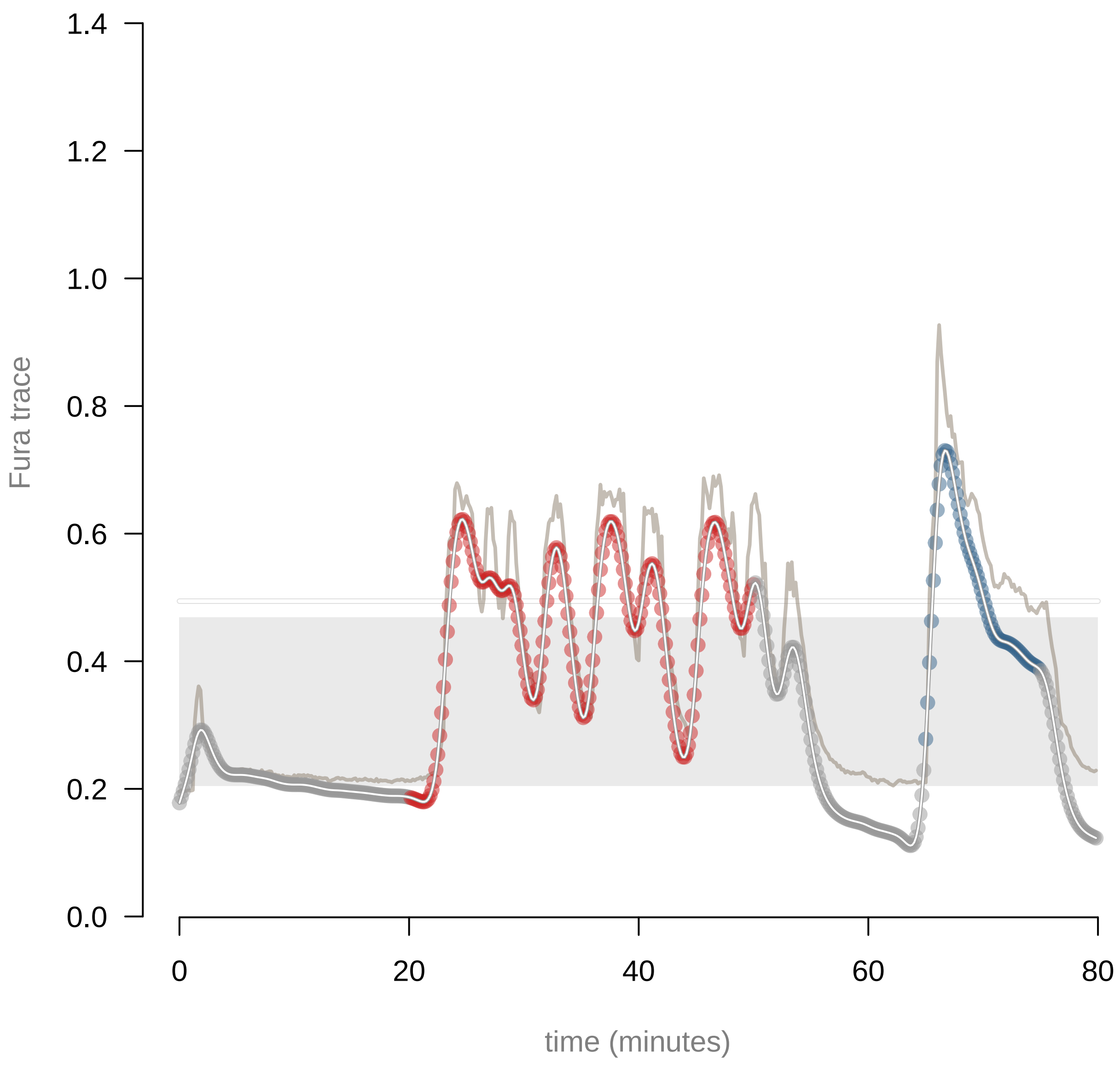

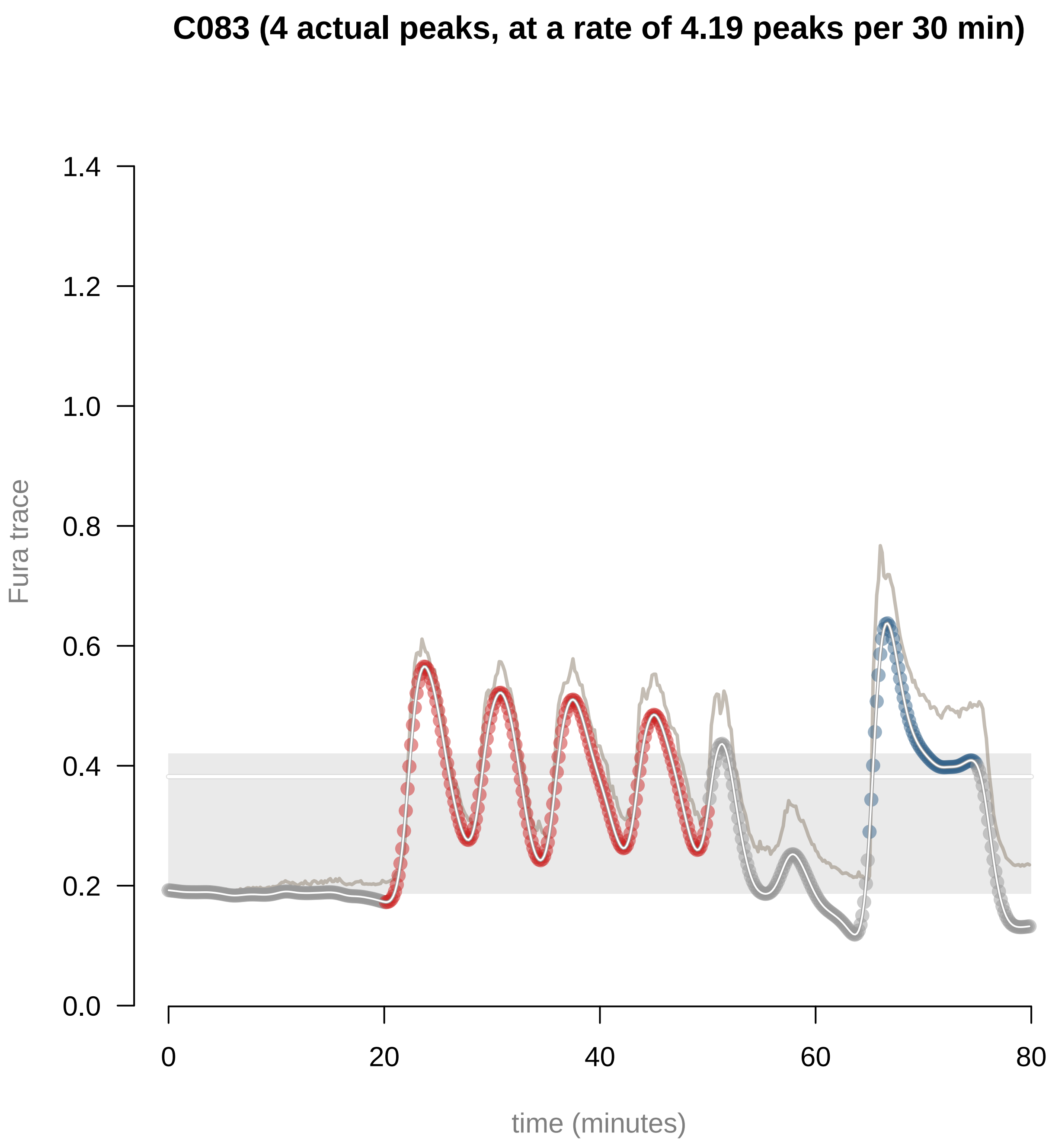
C083 (4 actual peaks, at a rate of 4.19 peaks per 30 min)
Fura trace
time (minutes)
0.0
0.2
0.4
0.6
0.8
1.0
1.2
1.4
0
20
40
60
80

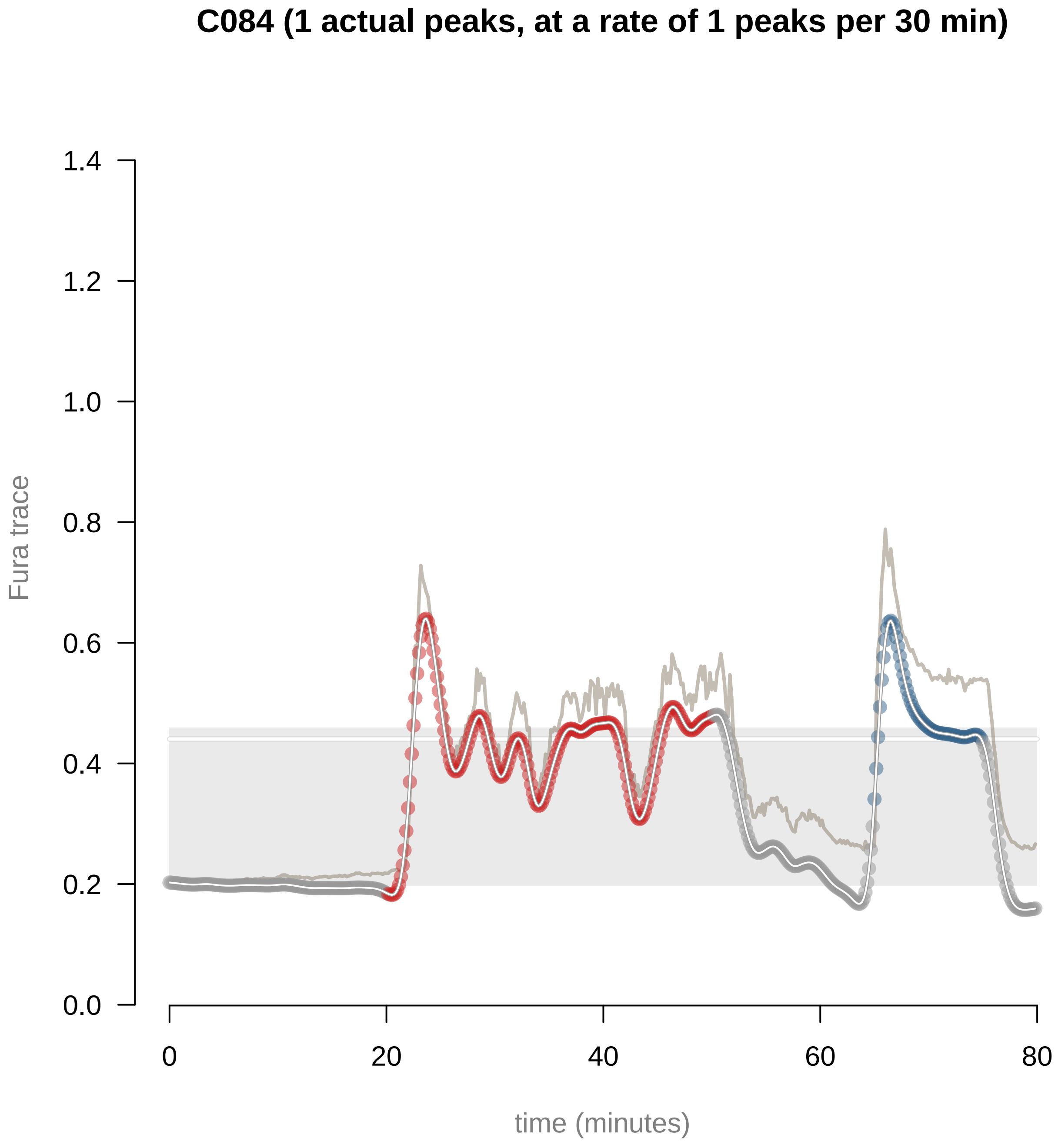

C084 (1 actual peaks, at a rate of 1 peaks per 30 min)
Fura trace
1.4
1.2
1.0
0.8
0.6
0.4
0.2
0.0
0
20
40
60
80
time (minutes)

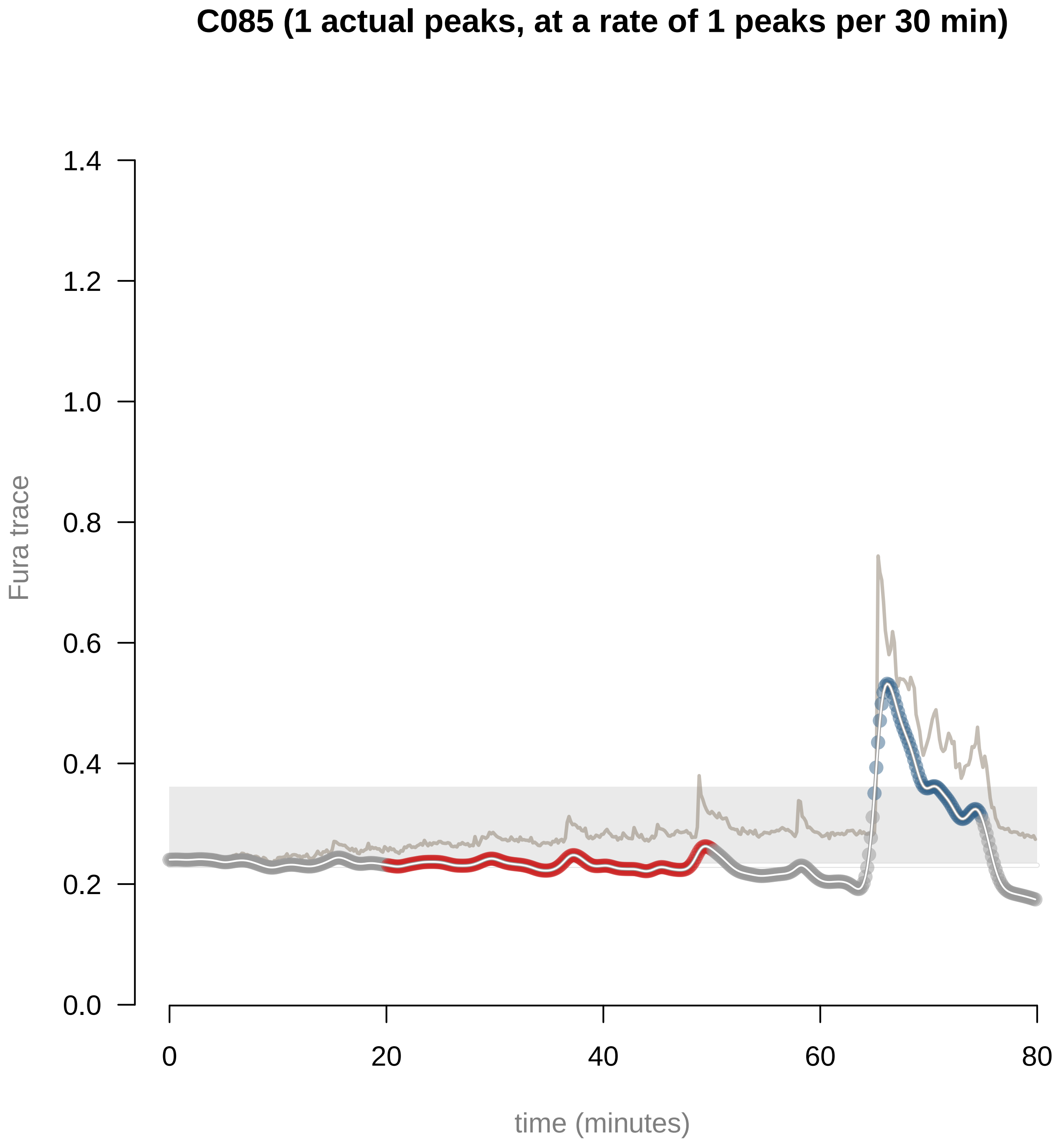
C085 (1 actual peaks, at a rate of 1 peaks per 30 min)
Fura trace
time (minutes)
0.0
0.2
0.4
0.6
0.8
1.0
1.2
1.4
0
20
40
60
80

C086 (3 actual peaks, at a rate of 3.08 peaks per 30 min)

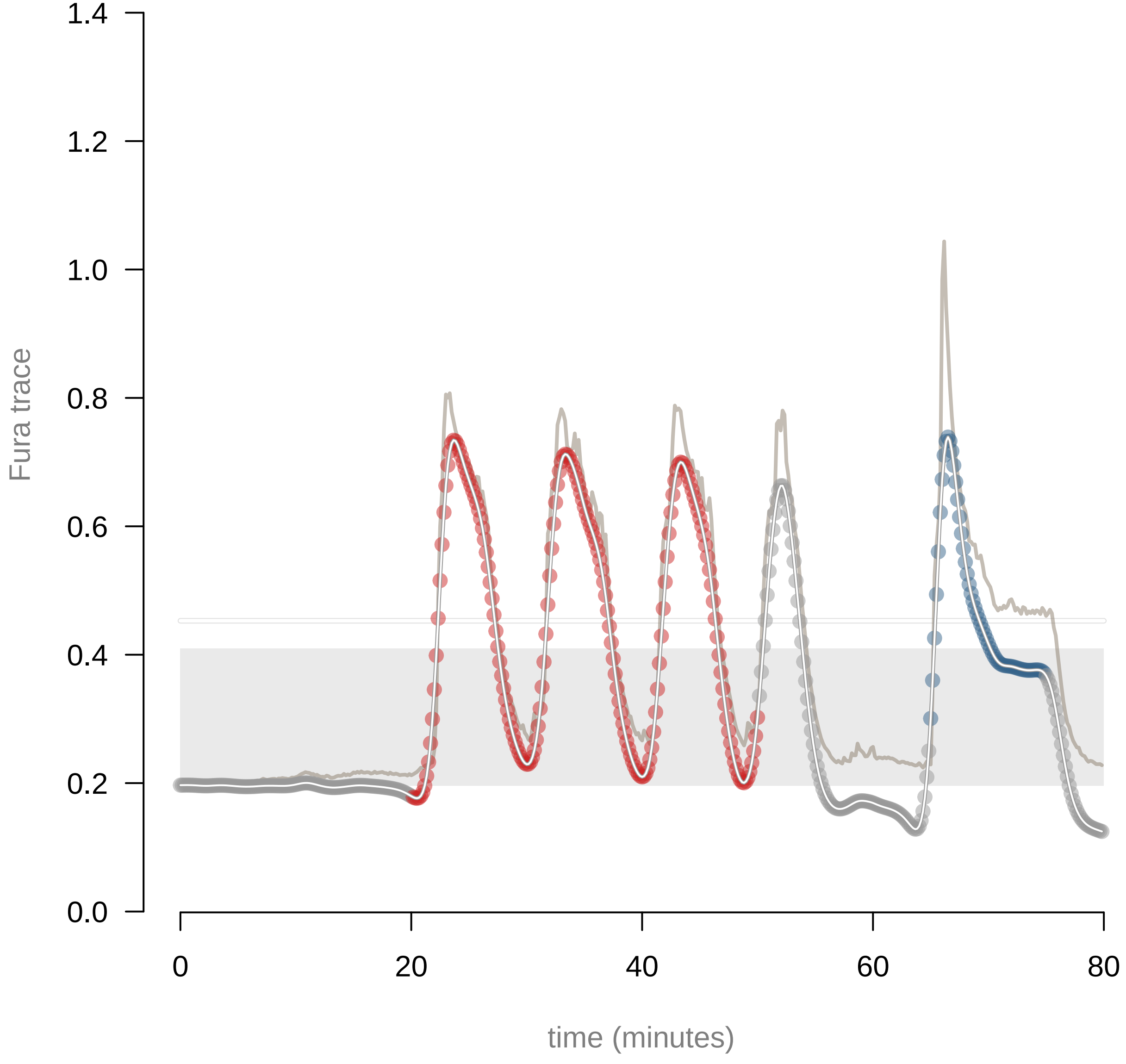

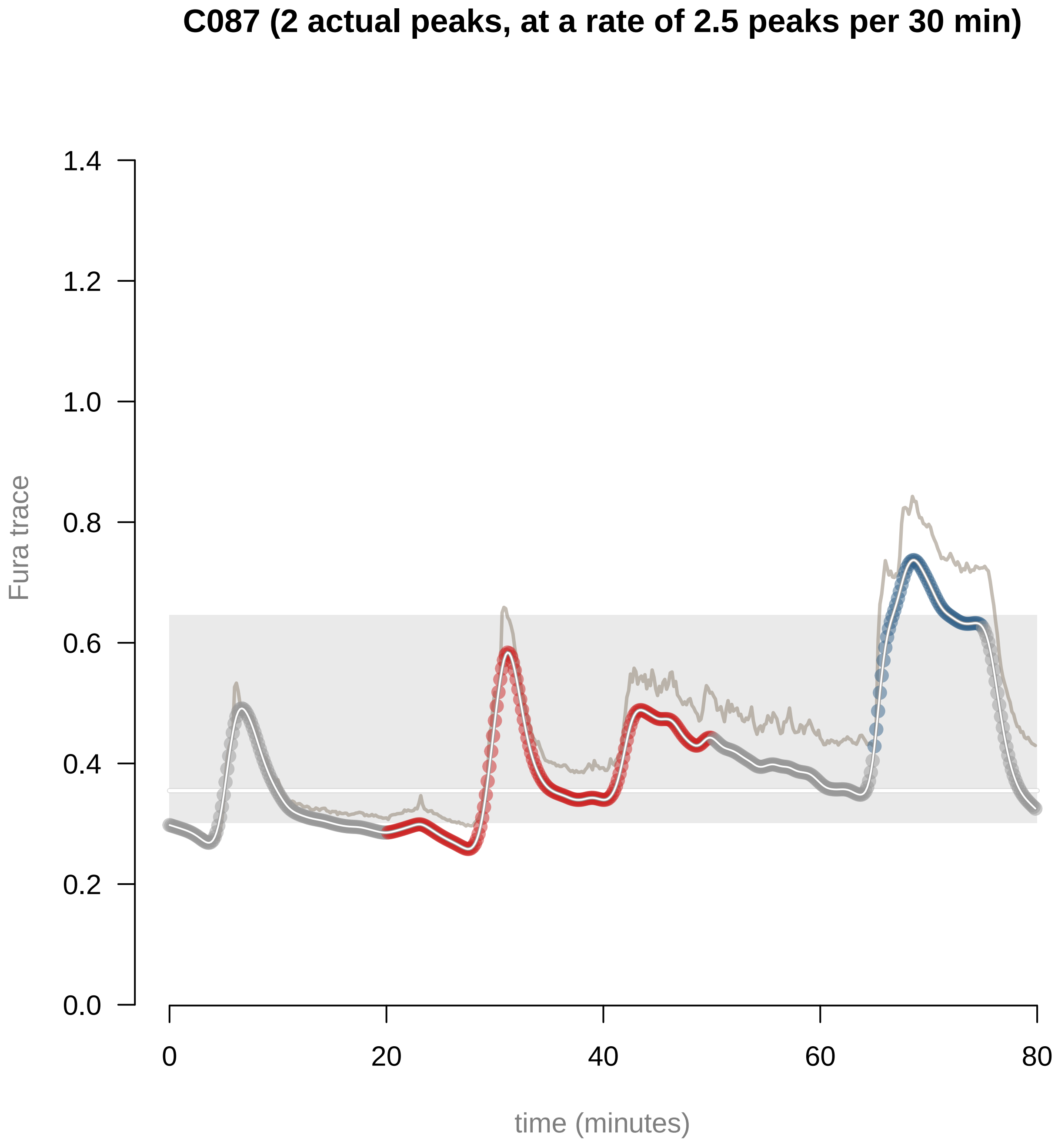
C087 (2 actual peaks, at a rate of 2.5 peaks per 30 min)
Fura trace
1.4
1.2
1.0
0.8
0.6
0.4
0.2
0.0
0
20
40
60
80
time (minutes)

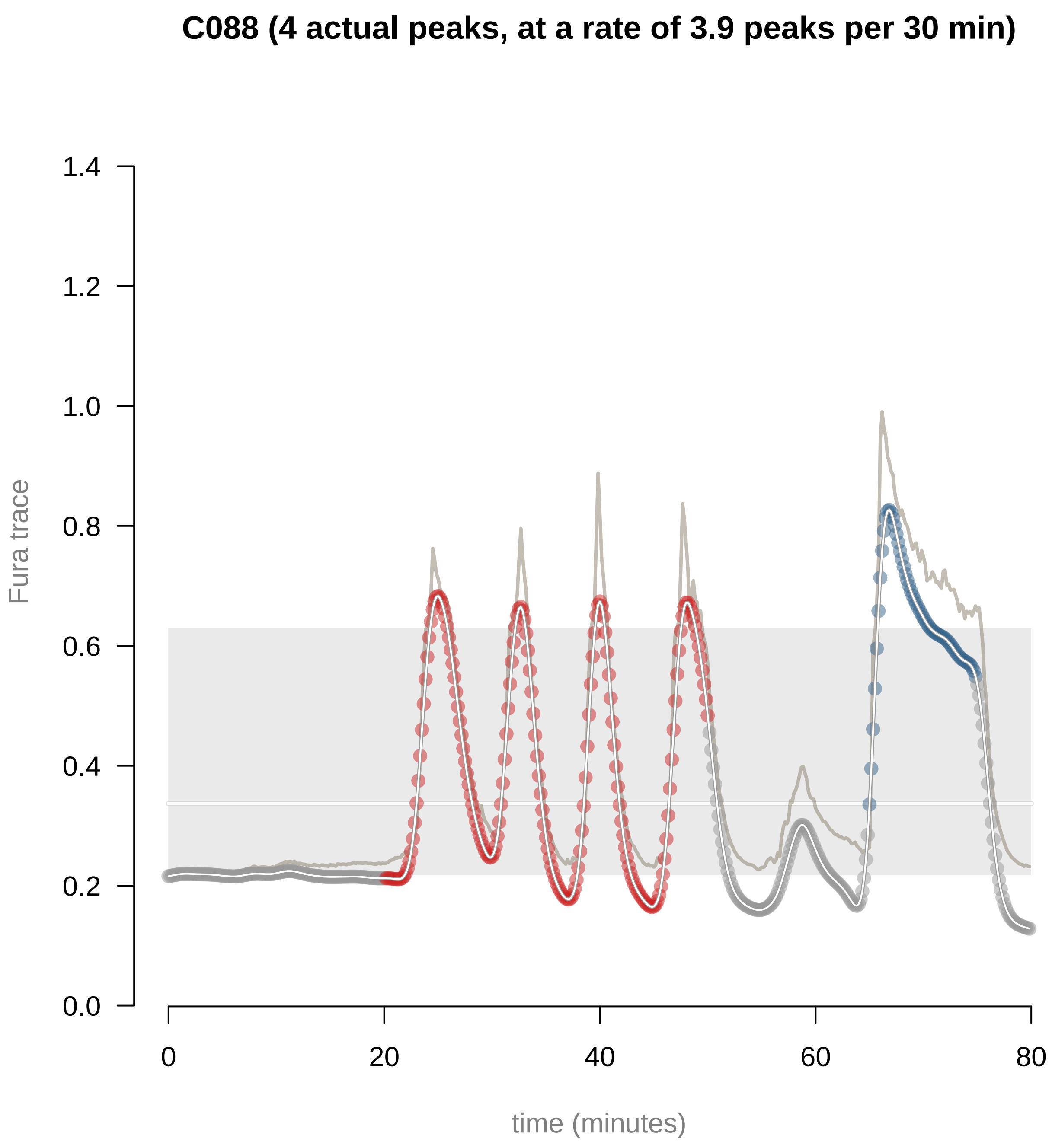
C088 (4 actual peaks, at a rate of 3.9 peaks per 30 min)
Fura trace
time (minutes)
0.0
0.2
0.4
0.6
0.8
1.0
1.2
1.4
0
20
40
60
80

C089 (0 actual peaks, at a rate of 0 peaks per 30 min)

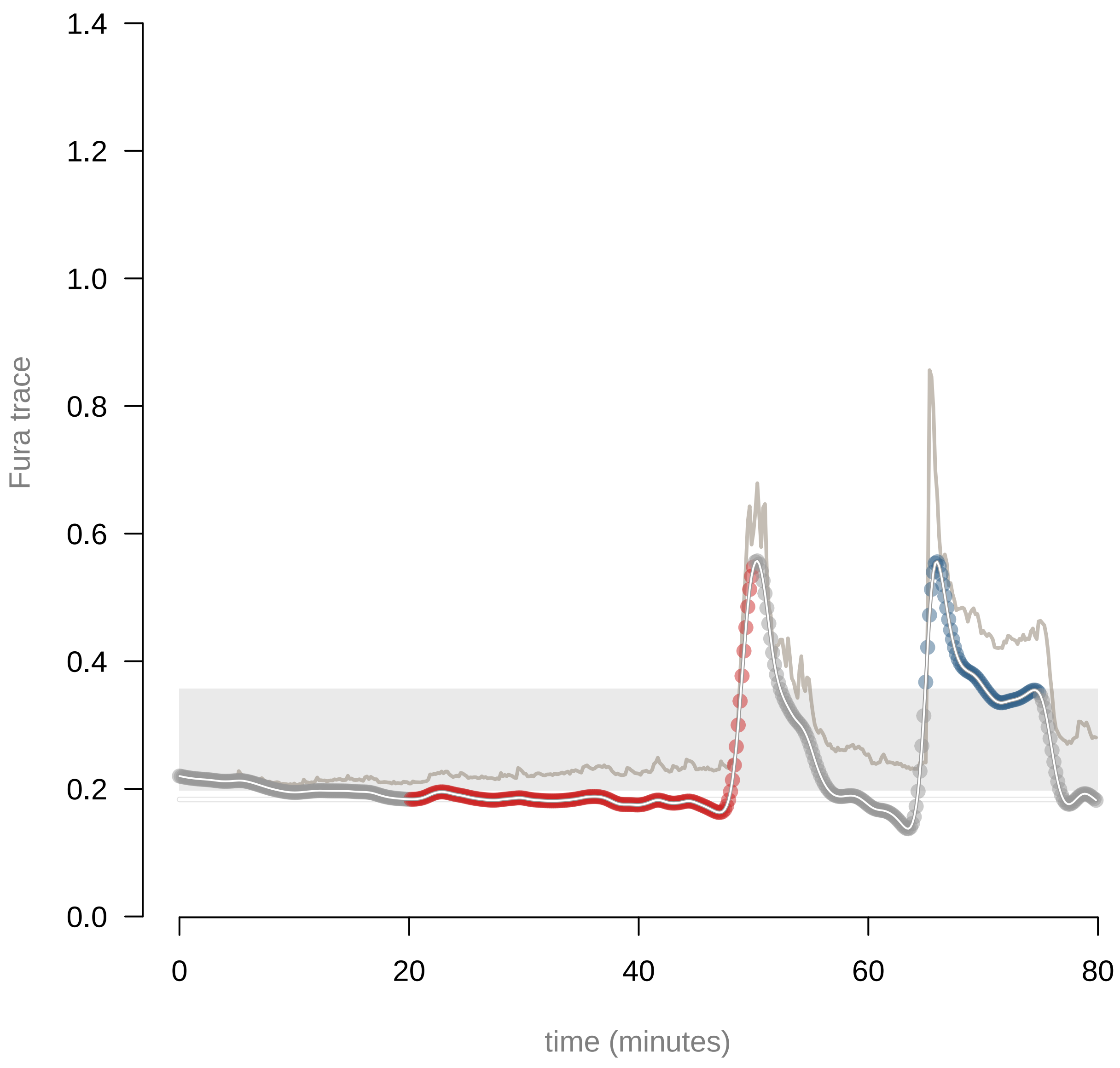

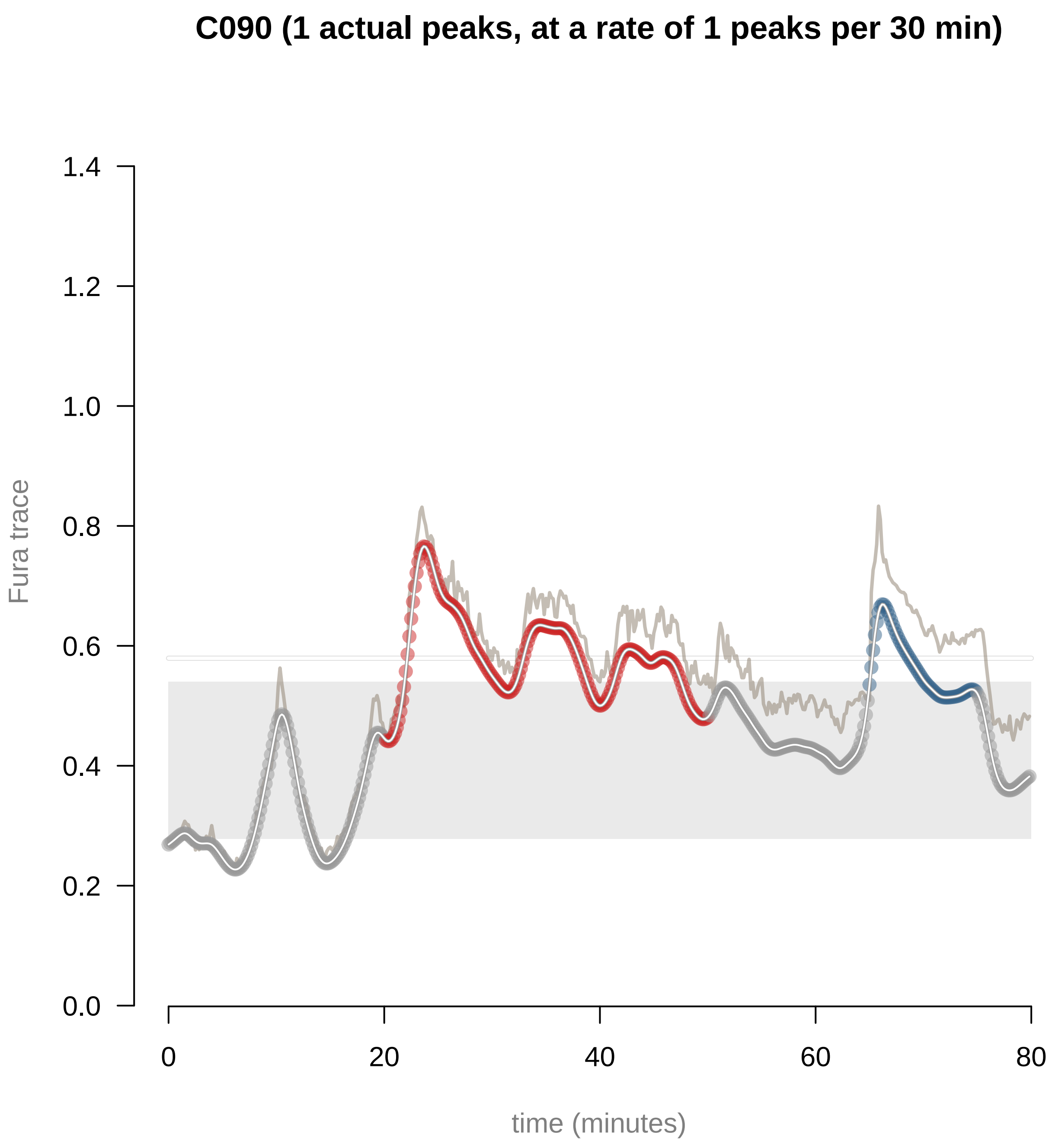

C090 (1 actual peaks, at a rate of 1 peaks per 30 min)
Fura trace
1.4
1.2
1.0
0.8
0.6
0.4
0.2
0.0
0
20
40
60
80
time (minutes)

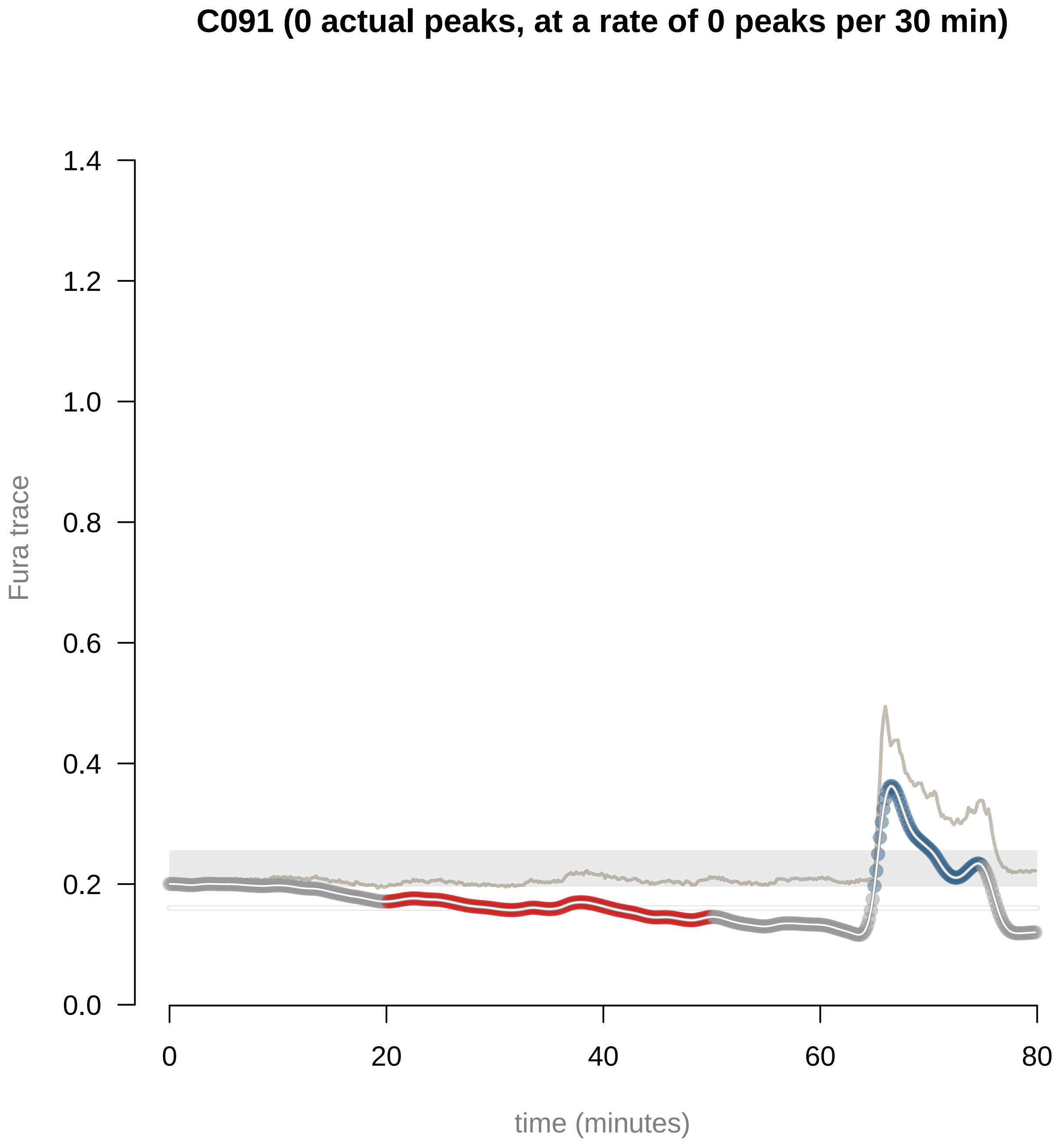
C091 (0 actual peaks, at a rate of 0 peaks per 30 min)
1.4
1.2
1.0
0.8
0.6
0.4
0.2
0.0
Fura trace
0
20
40
60
80
time (minutes)

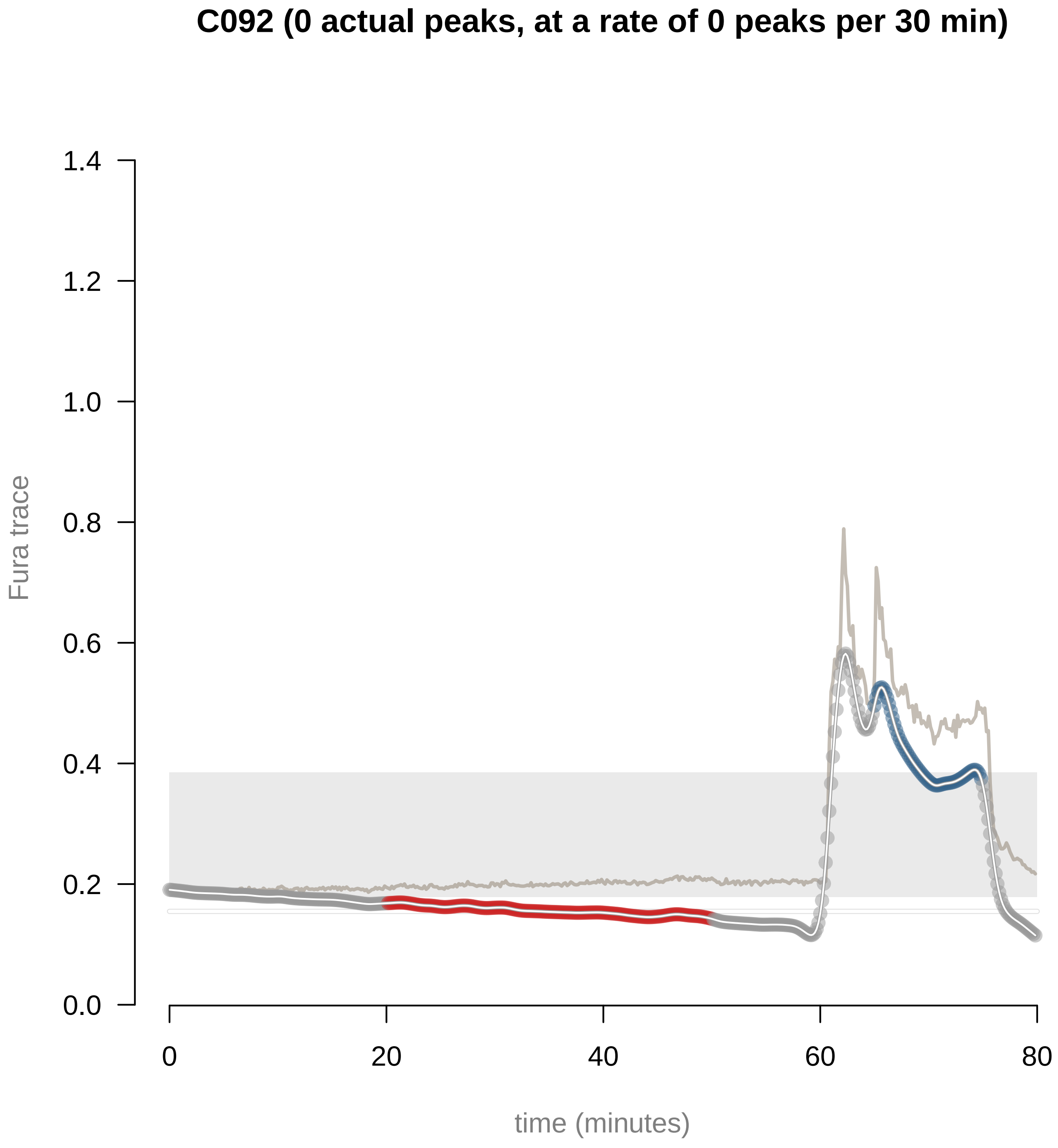

C092 (0 actual peaks, at a rate of 0 peaks per 30 min)
1.4
1.2
1.0
0.8
0.6
0.4
0.2
0.0
Fura trace
0
20
40
60
80
time (minutes)

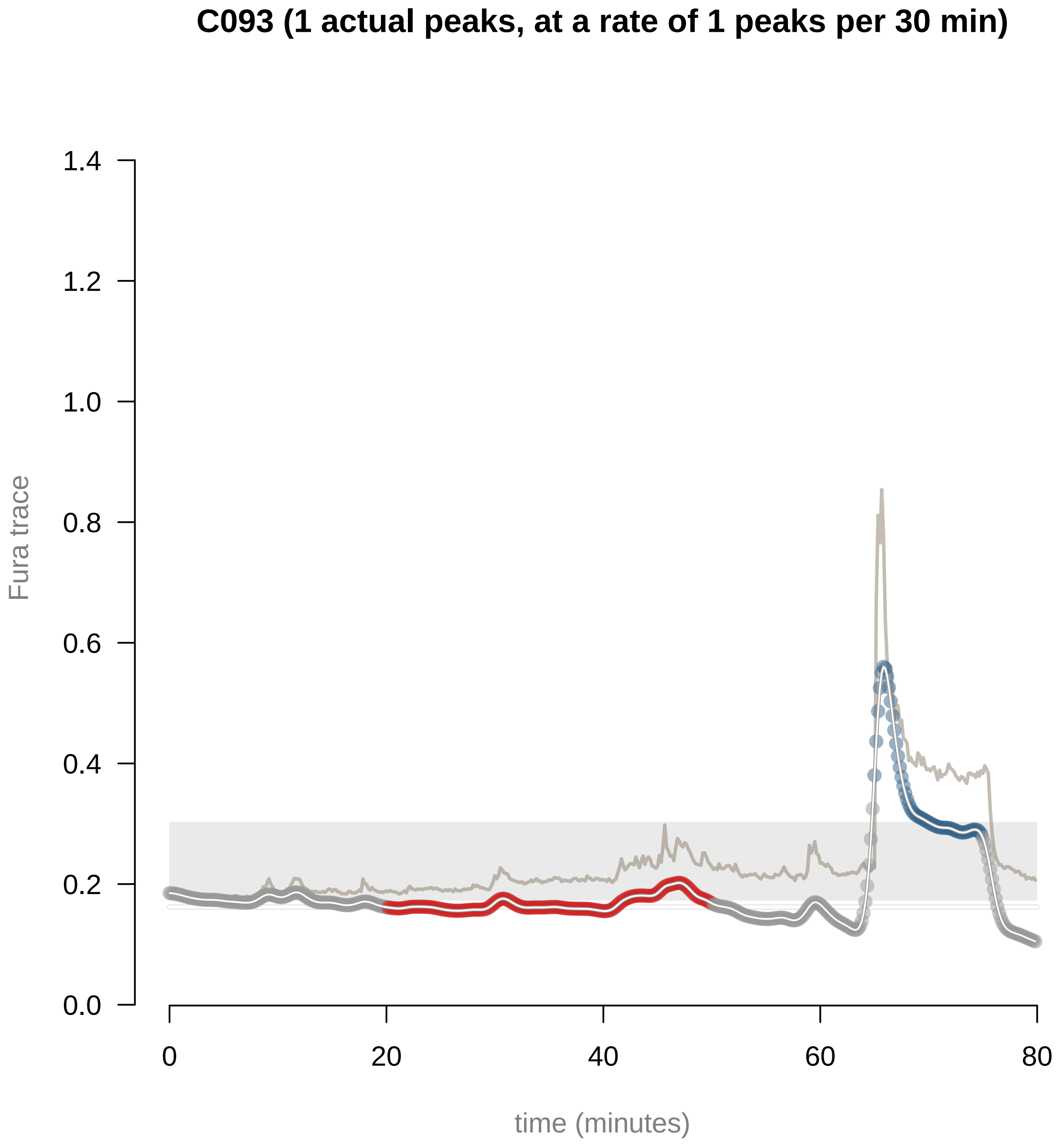

C093 (1 actual peaks, at a rate of 1 peaks per 30 min)
Fura trace
1.4
1.2
1.0
0.8
0.6
0.4
0.2
0.0
0
20
40
60
80
time (minutes)

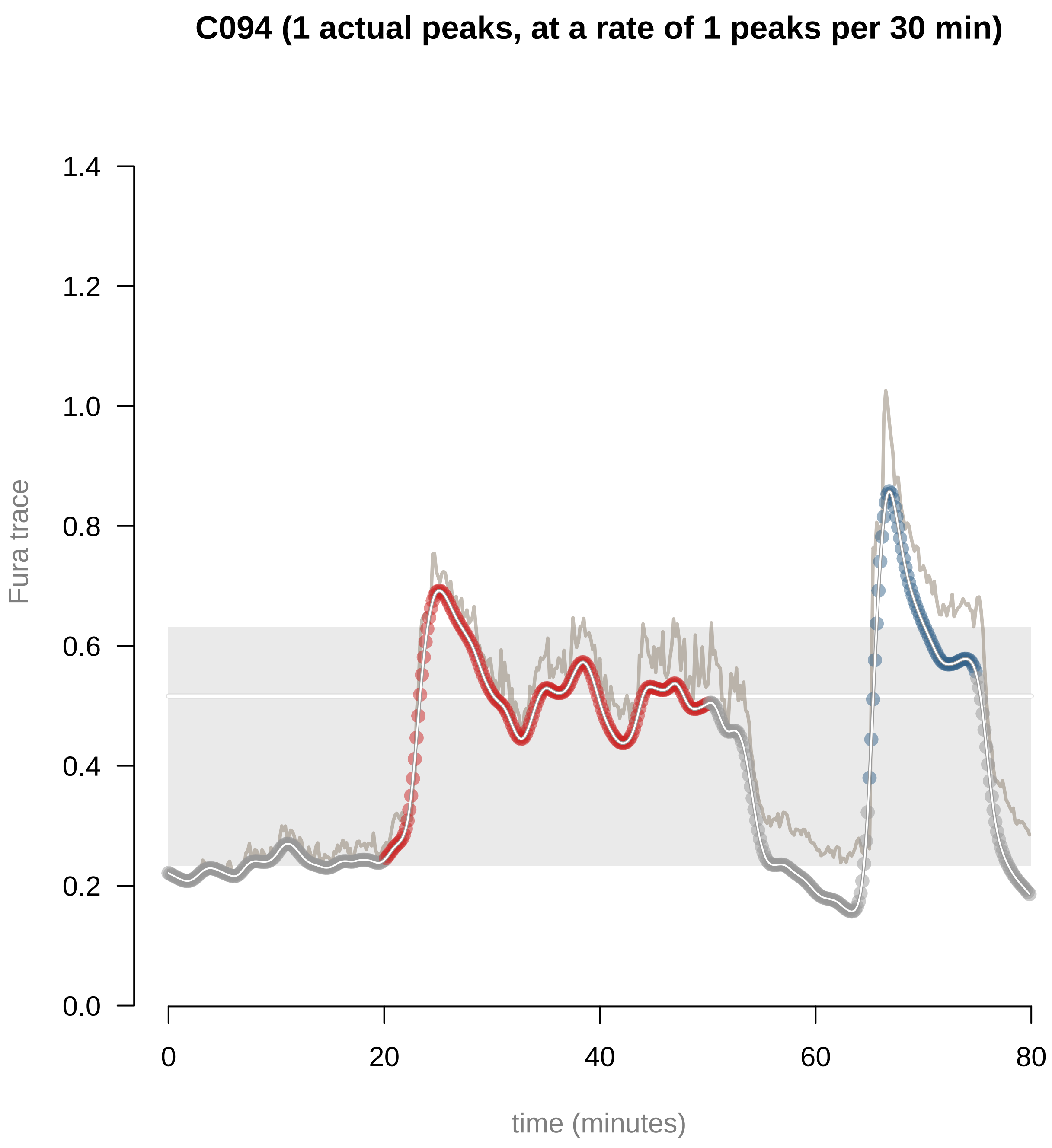
C094 (1 actual peaks, at a rate of 1 peaks per 30 min)
Fura trace
0.0
0.2
0.4
0.6
0.8
1.0
1.2
1.4
0
20
40
60
80
time (minutes)

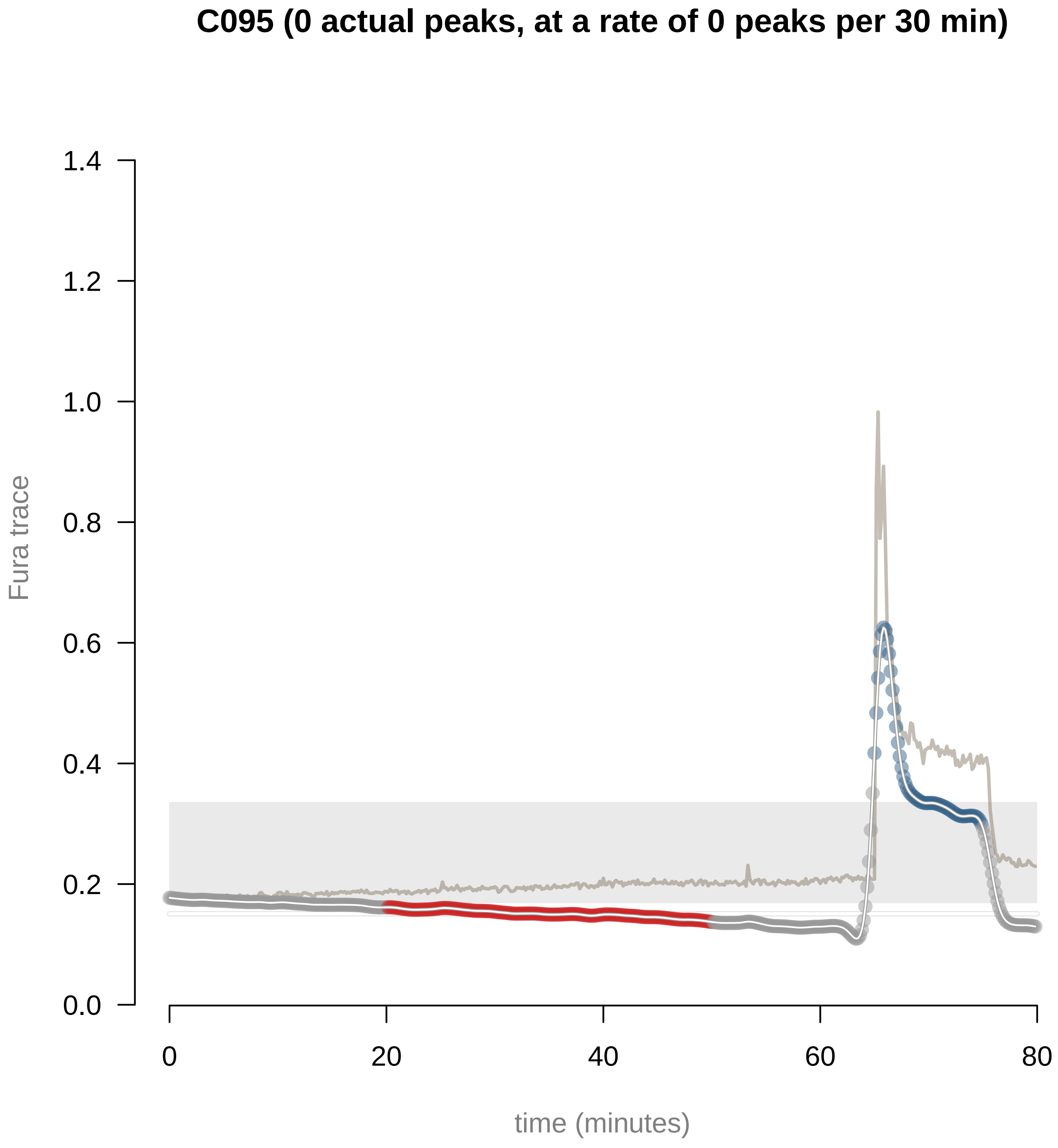
C095 (0 actual peaks, at a rate of 0 peaks per 30 min)
Fura trace
0.0
0.2
0.4
0.6
0.8
1.0
1.2
1.4
0
20
40
60
80
time (minutes)

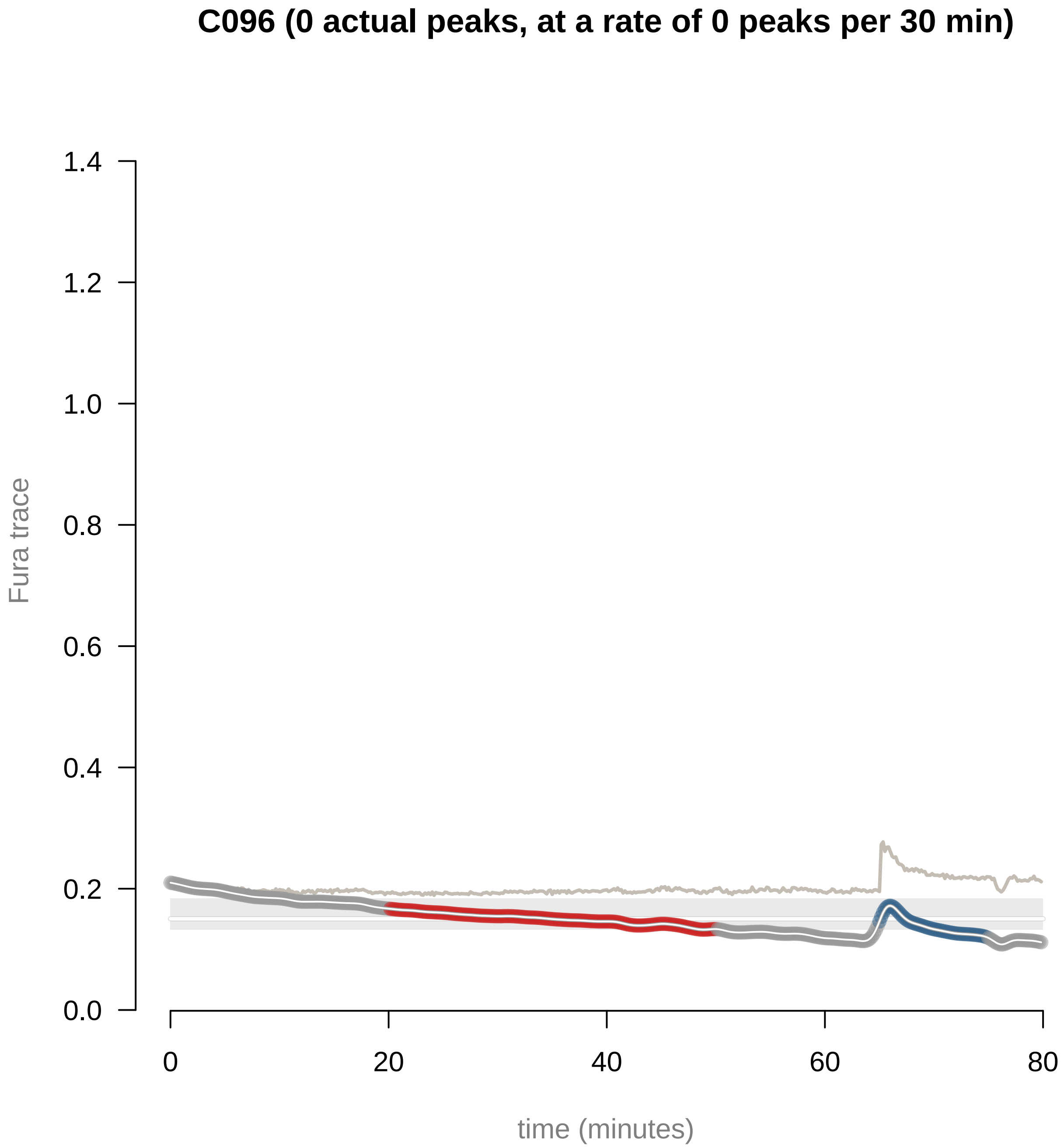
C096 (0 actual peaks, at a rate of 0 peaks per 30 min)
Fura trace
time (minutes)
0.0
0.2
0.4
0.6
0.8
1.0
1.2
1.4
0
20
40
60
80

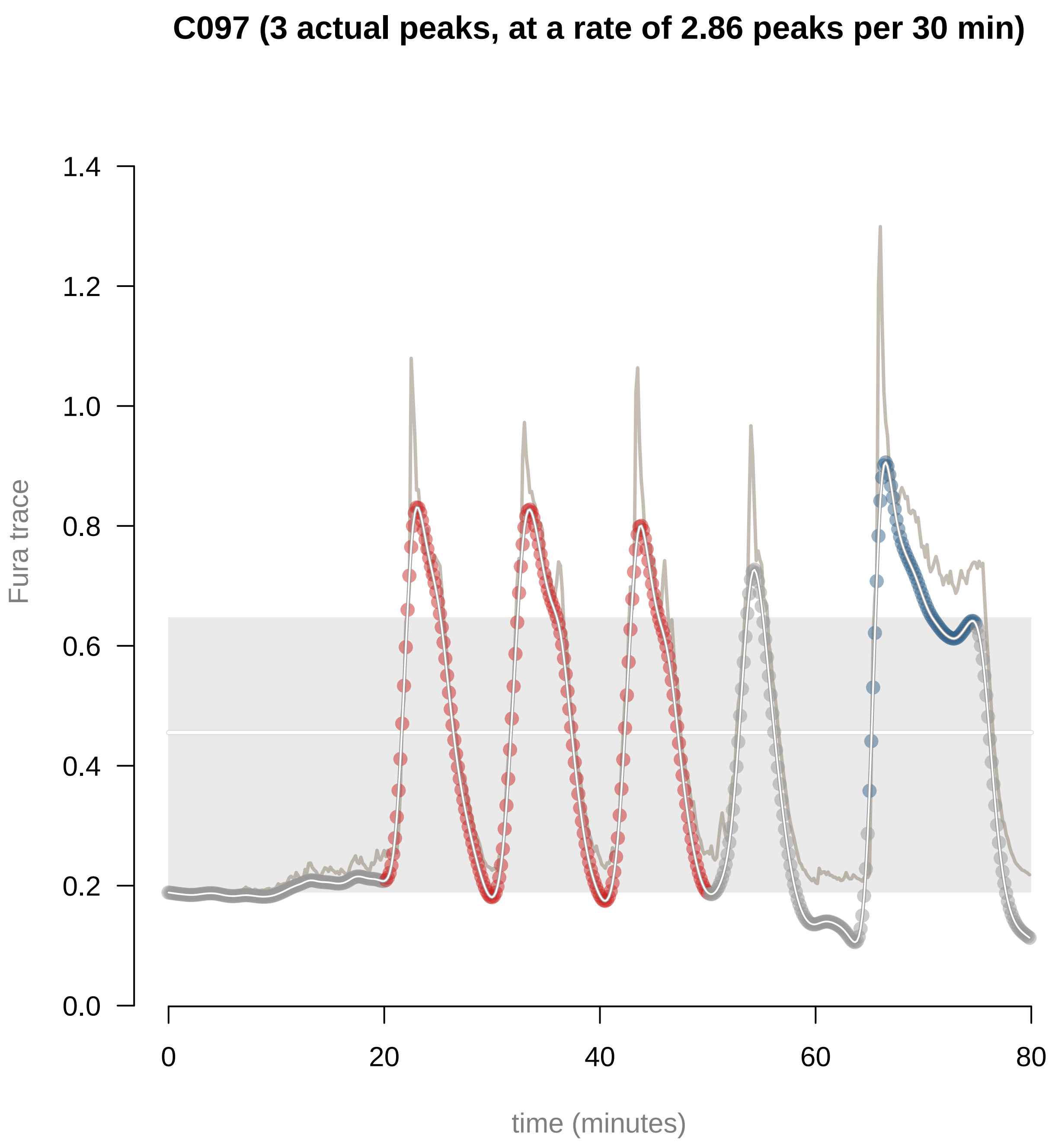
C097 (3 actual peaks, at a rate of 2.86 peaks per 30 min)
Fura trace
1.4
1.2
1.0
0.8
0.6
0.4
0.2
0.0
0
20
40
60
80
time (minutes)

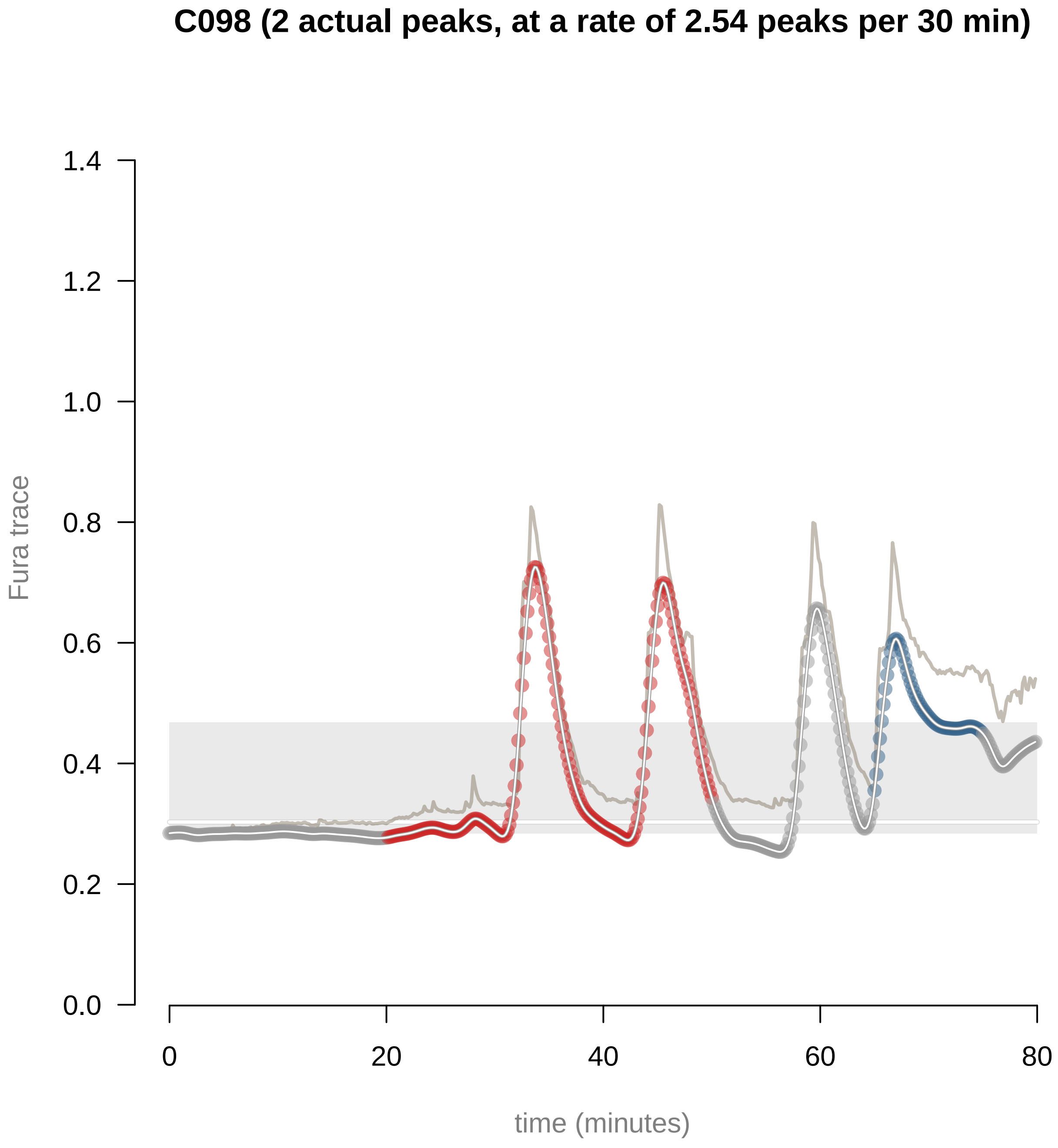
C098 (2 actual peaks, at a rate of 2.54 peaks per 30 min)
Fura trace
1.4
1.2
1.0
0.8
0.6
0.4
0.2
0.0
0
20
40
60
80
time (minutes)

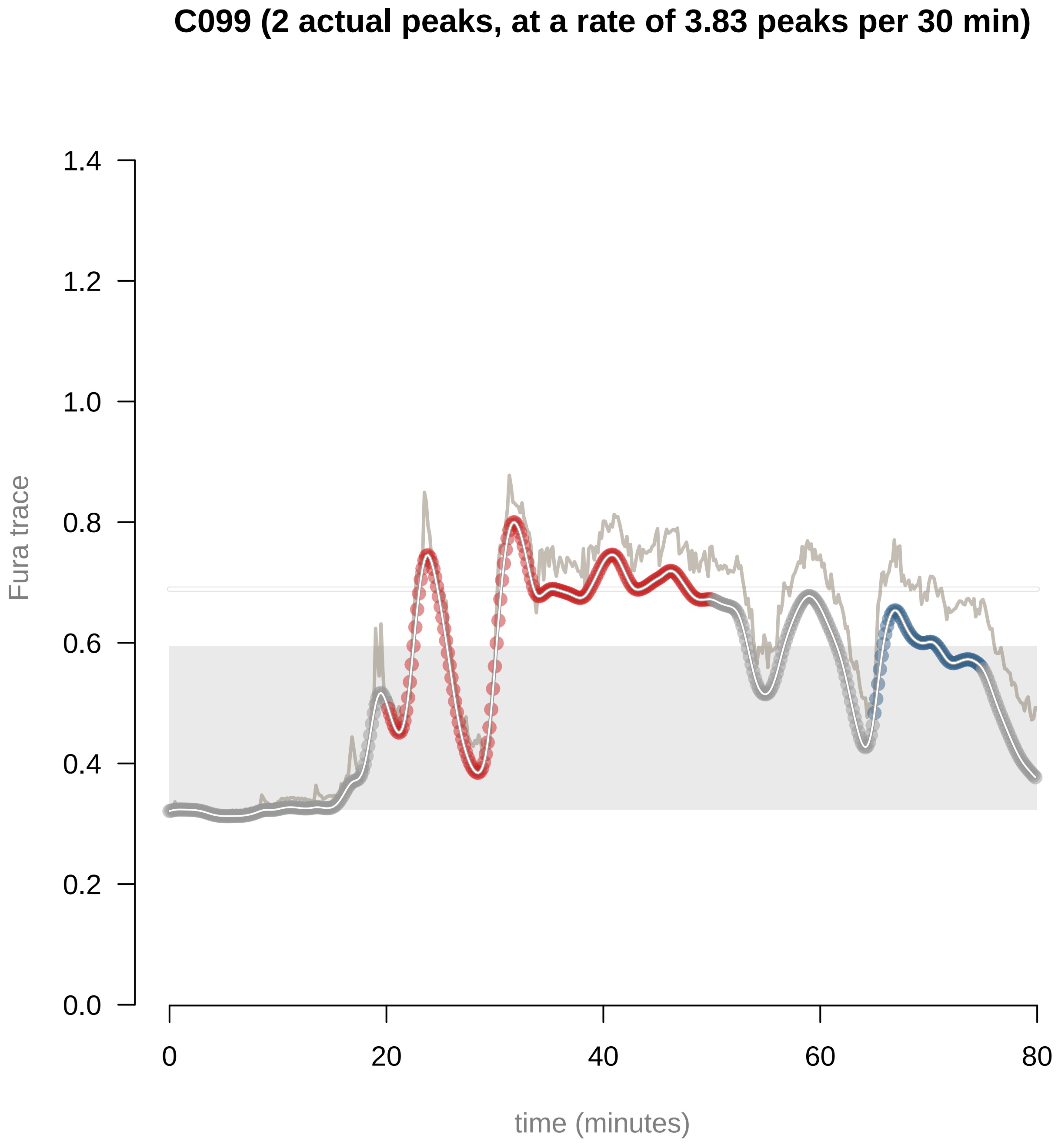

C099 (2 actual peaks, at a rate of 3.83 peaks per 30 min)
Fura trace
1.4
1.2
1.0
0.8
0.6
0.4
0.2
0.0
0
20
40
60
80
time (minutes)

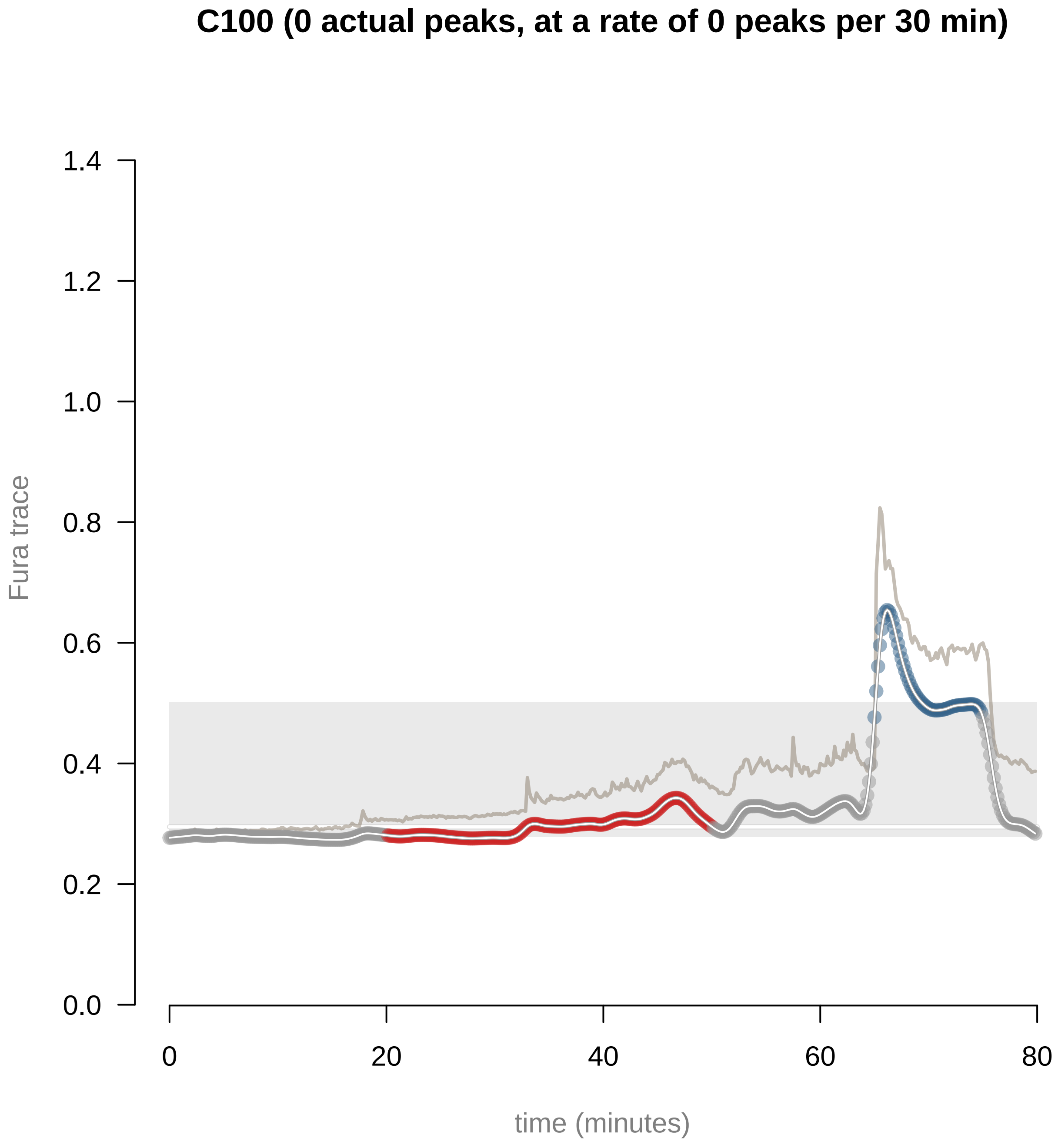

C100 (0 actual peaks, at a rate of 0 peaks per 30 min)
Fura trace
time (minutes)
0.0
0.2
0.4
0.6
0.8
1.0
1.2
1.4
0
20
40
60
80

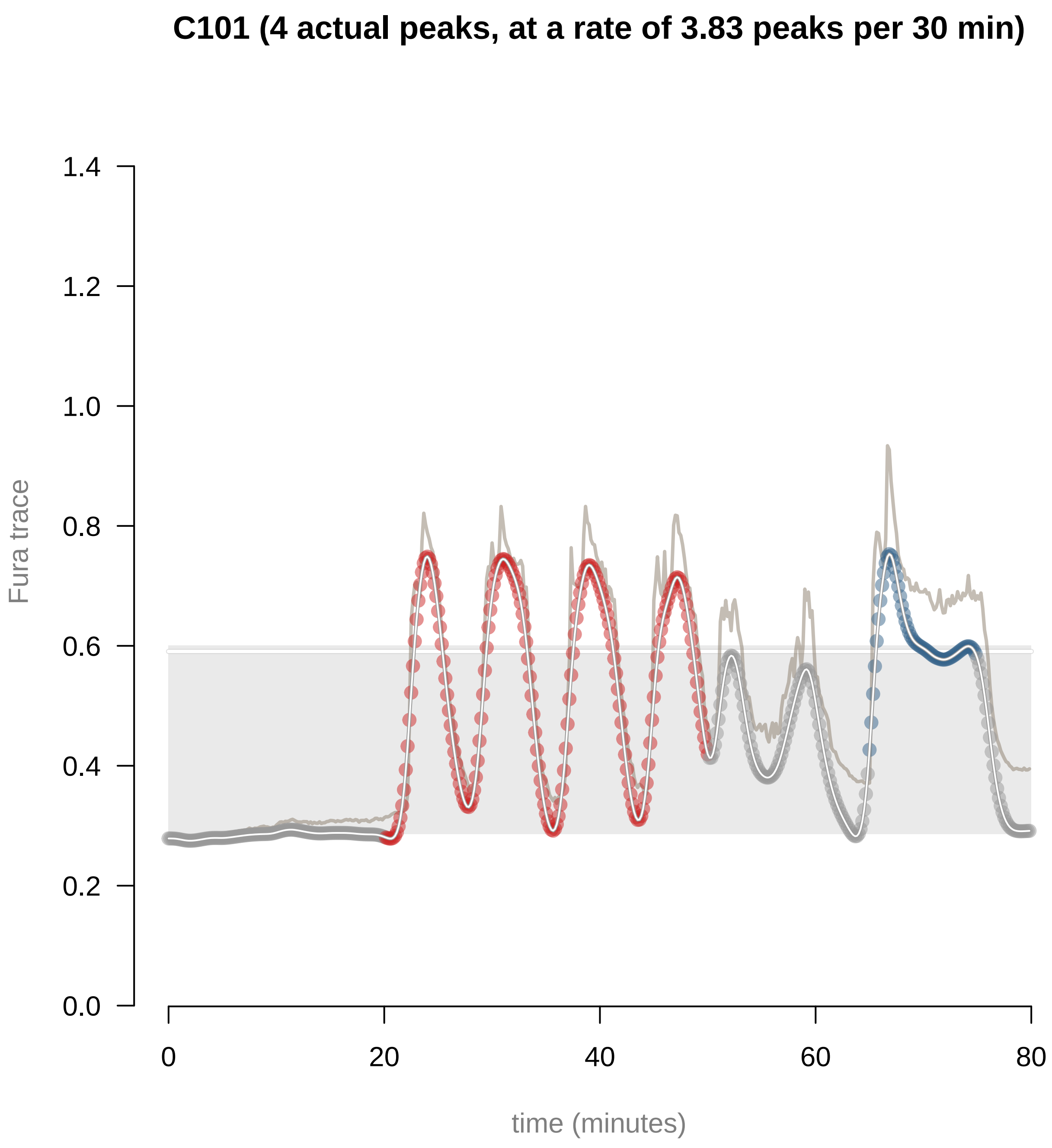

C101 (4 actual peaks, at a rate of 3.83 peaks per 30 min)
1.4
1.2
1.0
0.8
0.6
0.4
0.2
0.0
Fura trace
0
20
40
60
80
time (minutes)

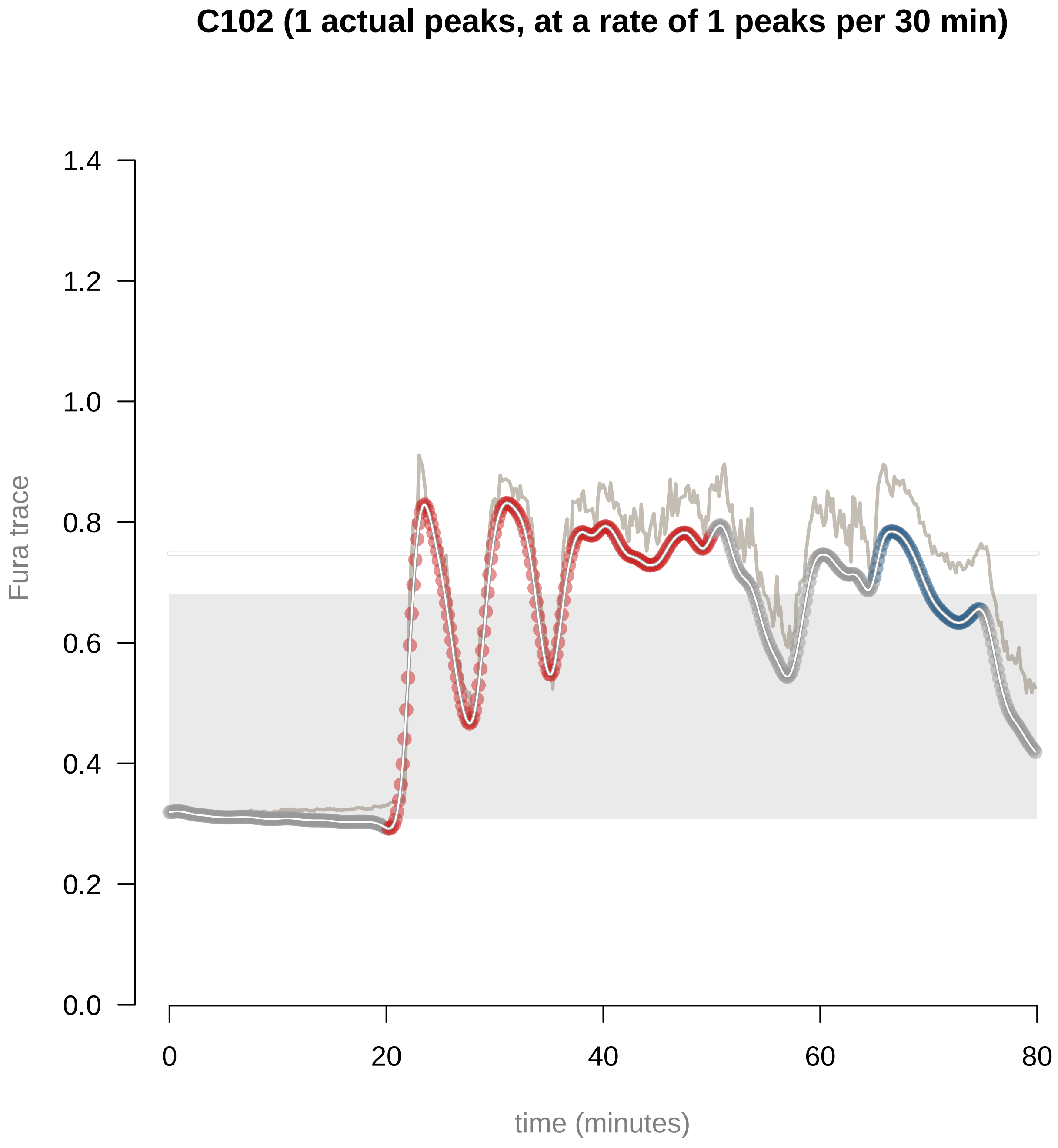
C102 (1 actual peaks, at a rate of 1 peaks per 30 min)
Fura trace
time (minutes)
0.0
0.2
0.4
0.6
0.8
1.0
1.2
1.4
0
20
40
60
80

C103 (0 actual peaks, at a rate of 0 peaks per 30 min)

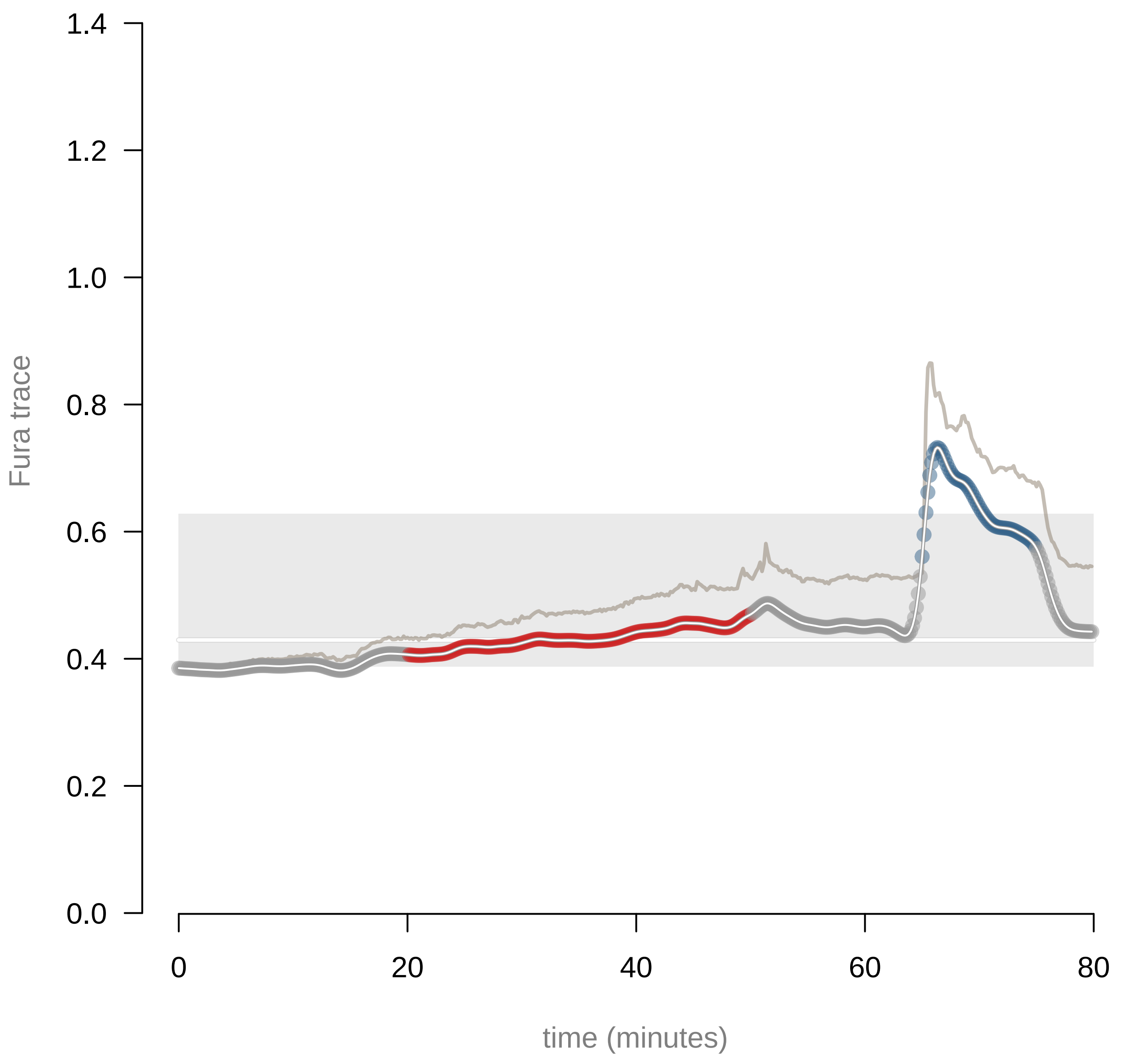

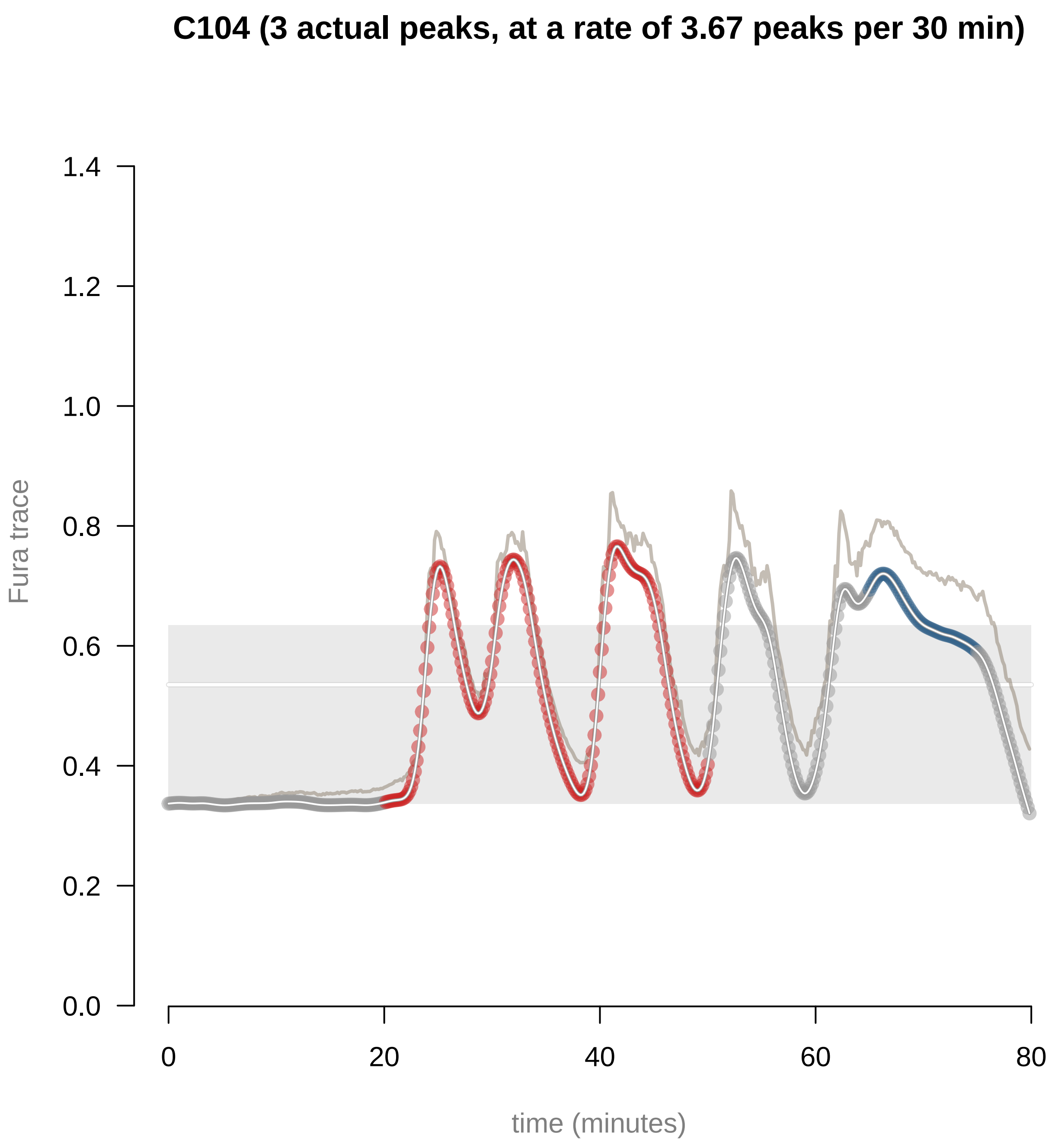

C104 (3 actual peaks, at a rate of 3.67 peaks per 30 min)
Fura trace
1.4
1.2
1.0
0.8
0.6
0.4
0.2
0.0
0
20
40
60
80
time (minutes)

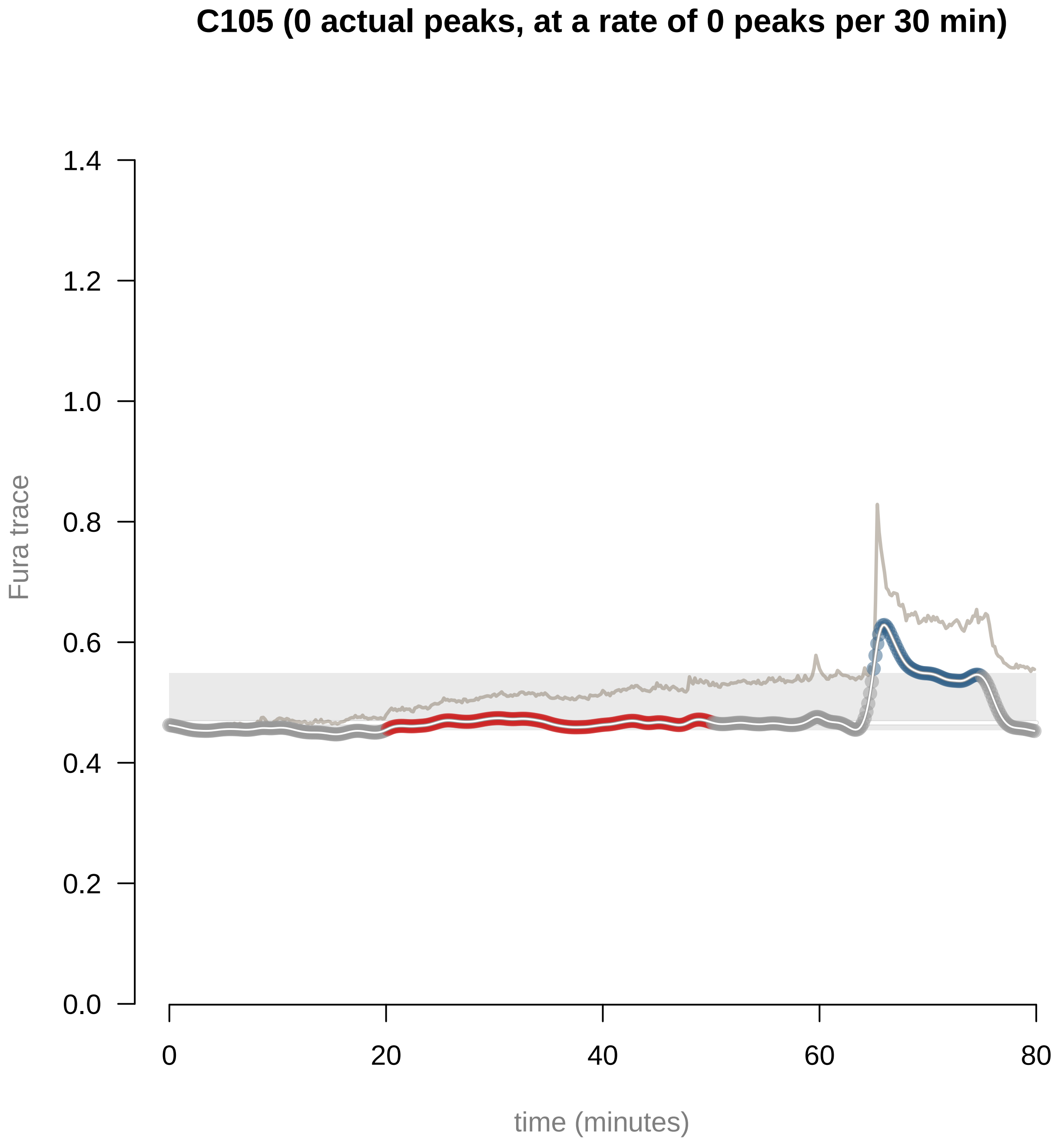

C105 (0 actual peaks, at a rate of 0 peaks per 30 min)
Fura trace
0.0
0.2
0.4
0.6
0.8
1.0
1.2
1.4
0
20
40
60
80
time (minutes)

C106 (2 actual peaks, at a rate of 2.5 peaks per 30 min)

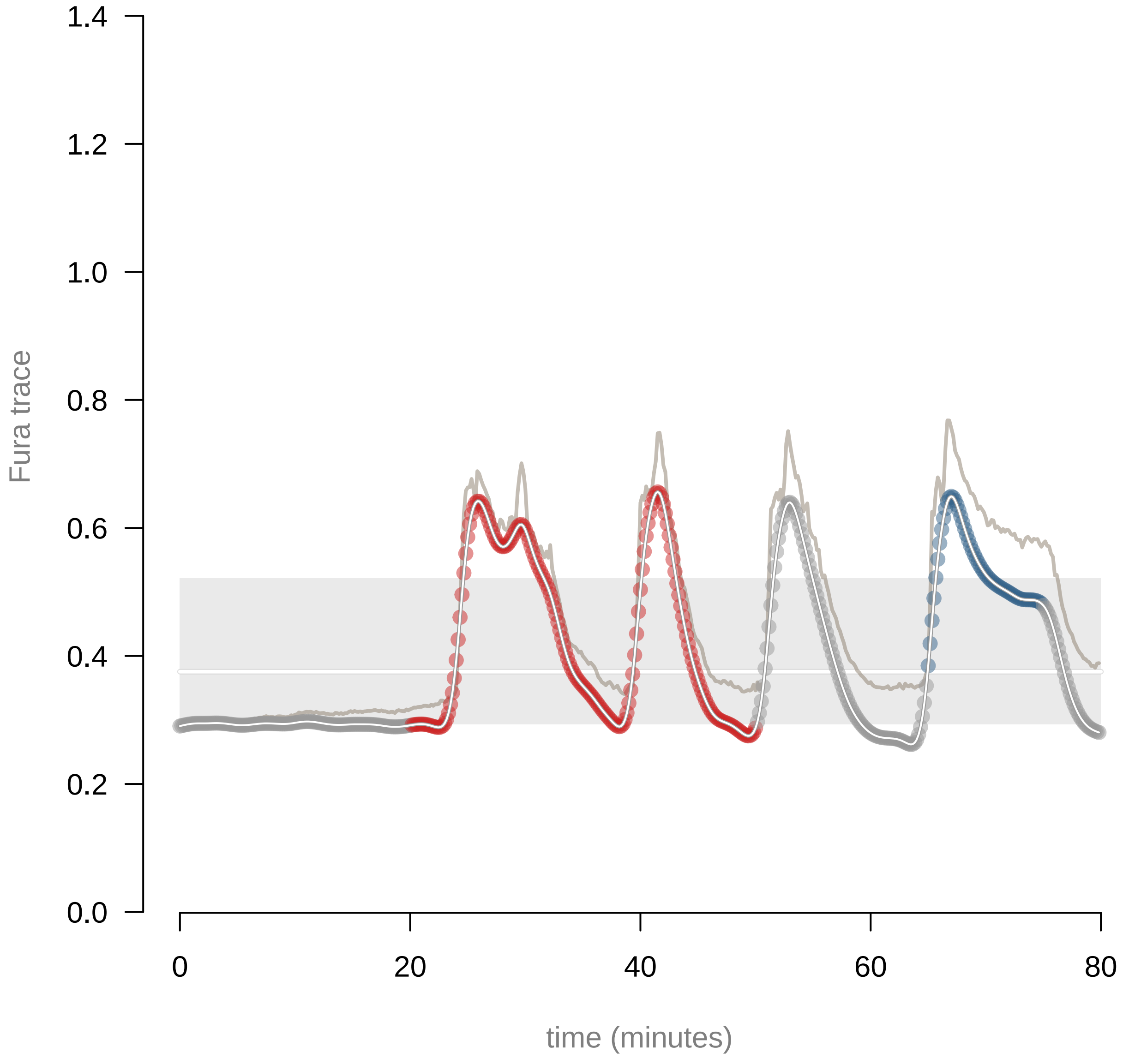

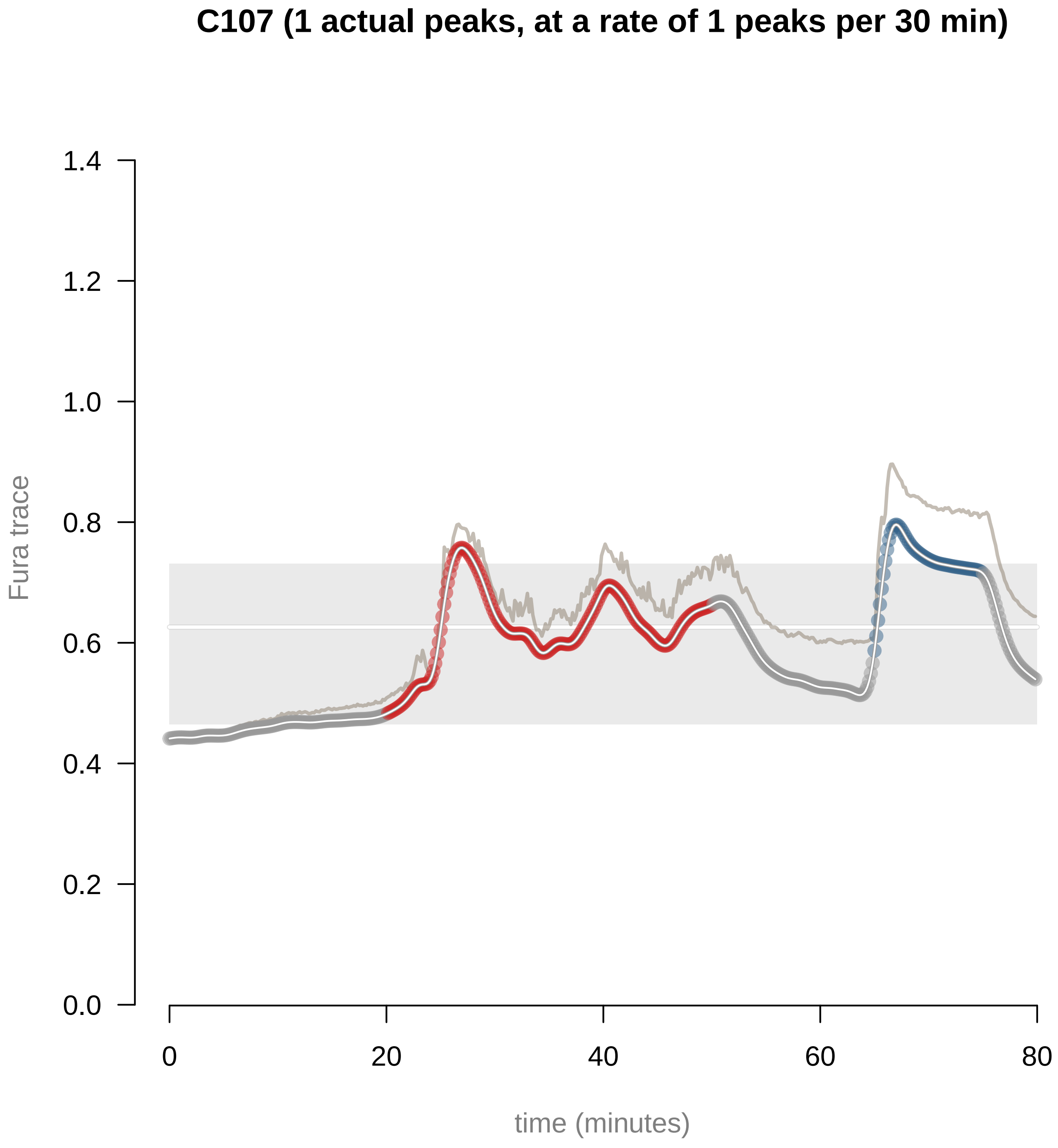
C107 (1 actual peaks, at a rate of 1 peaks per 30 min)
Fura trace
time (minutes)
0.0
0.2
0.4
0.6
0.8
1.0
1.2
1.4
0
20
40
60
80

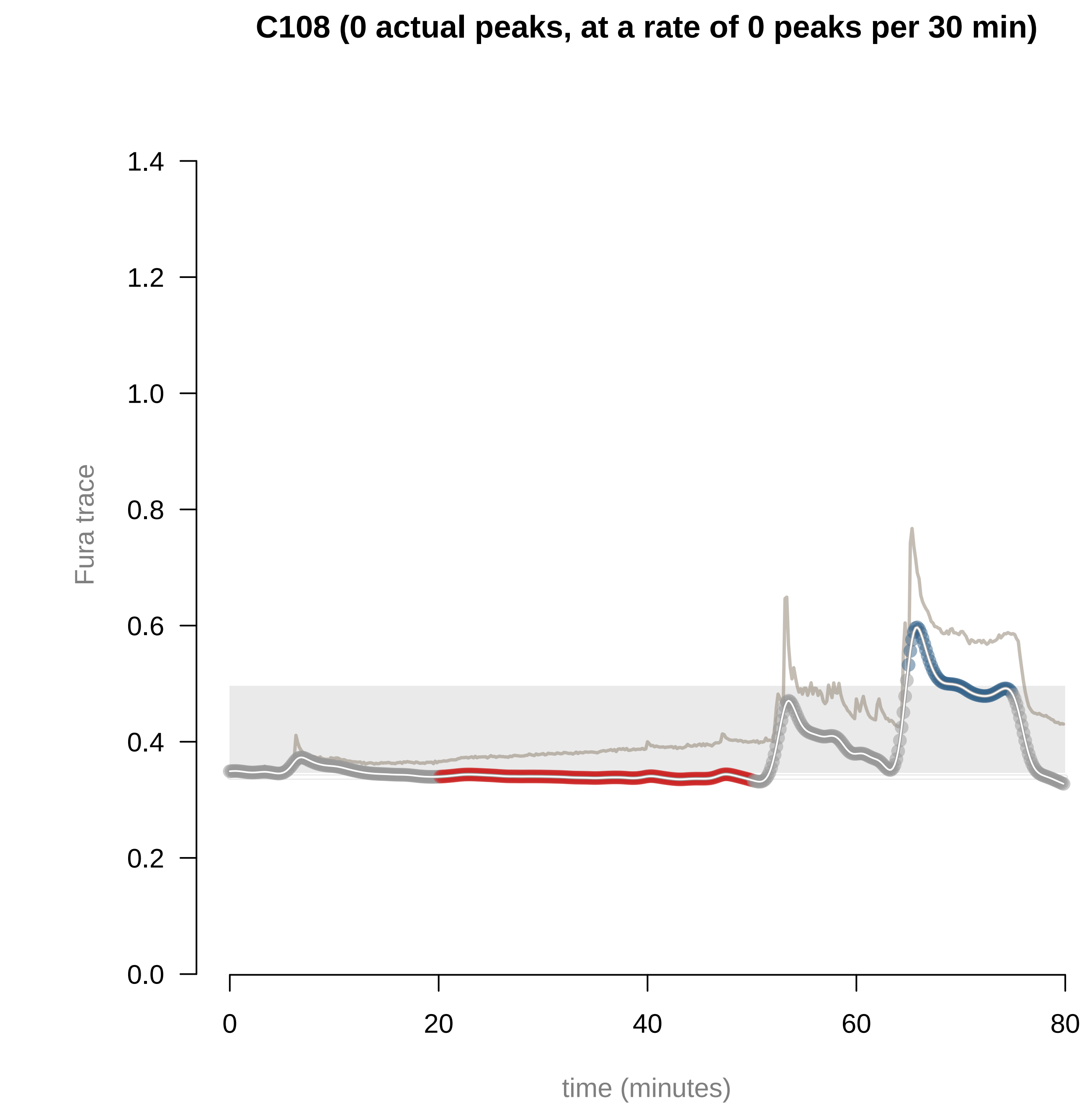

C108 (0 actual peaks, at a rate of 0 peaks per 30 min)
Fura trace
time (minutes)
0.0
0.2
0.4
0.6
0.8
1.0
1.2
1.4
0
20
40
60
80

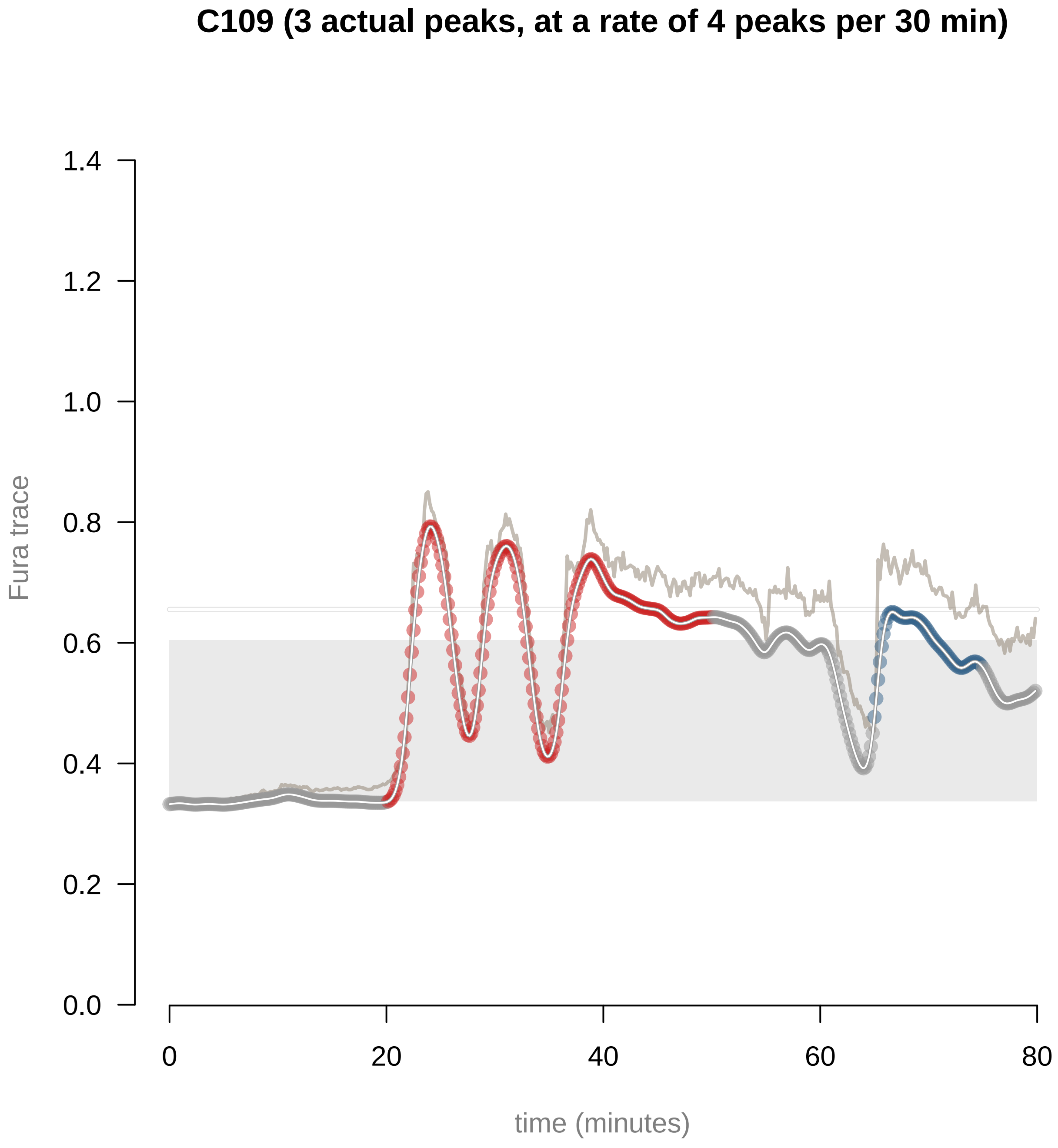
C109 (3 actual peaks, at a rate of 4 peaks per 30 min)
1.4
1.2
1.0
0.8
0.6
0.4
0.2
0.0
Fura trace
0
20
40
60
80
time (minutes)

C110 (2 actual peaks, at a rate of 4.19 peaks per 30 min)

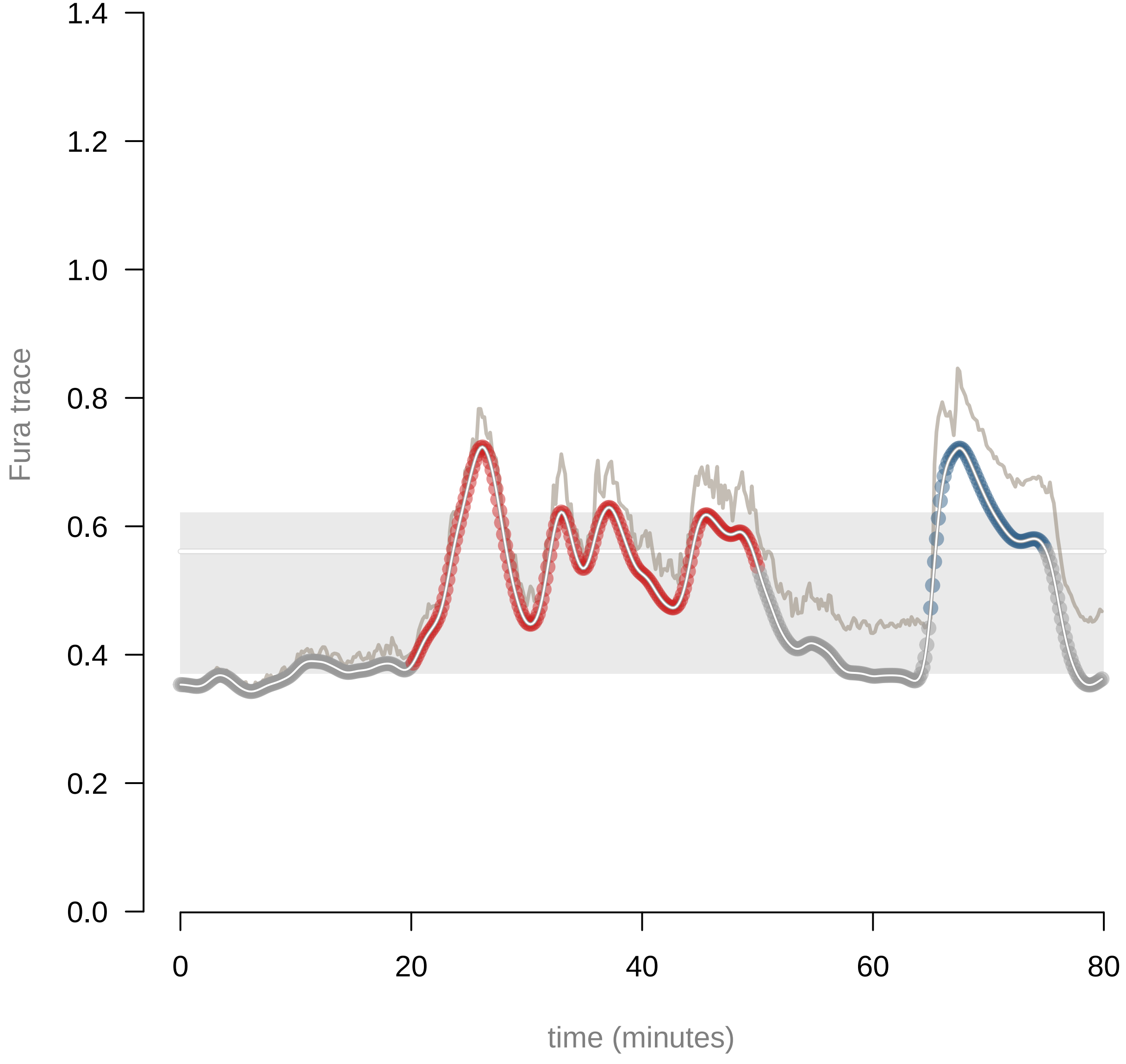

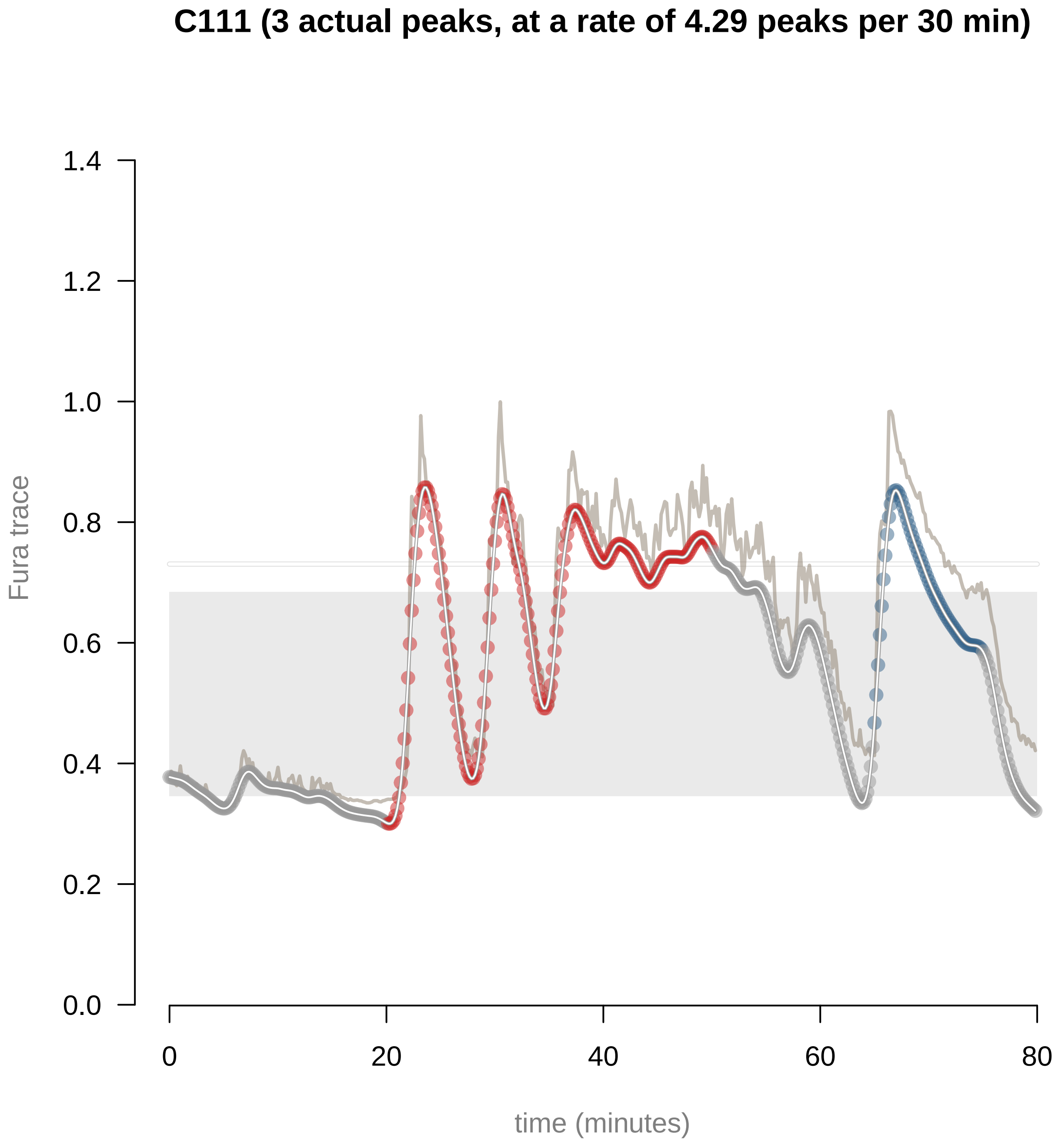
C111 (3 actual peaks, at a rate of 4.29 peaks per 30 min)
Fura trace
1.4
1.2
1.0
0.8
0.6
0.4
0.2
0.0
0
20
40
60
80
time (minutes)

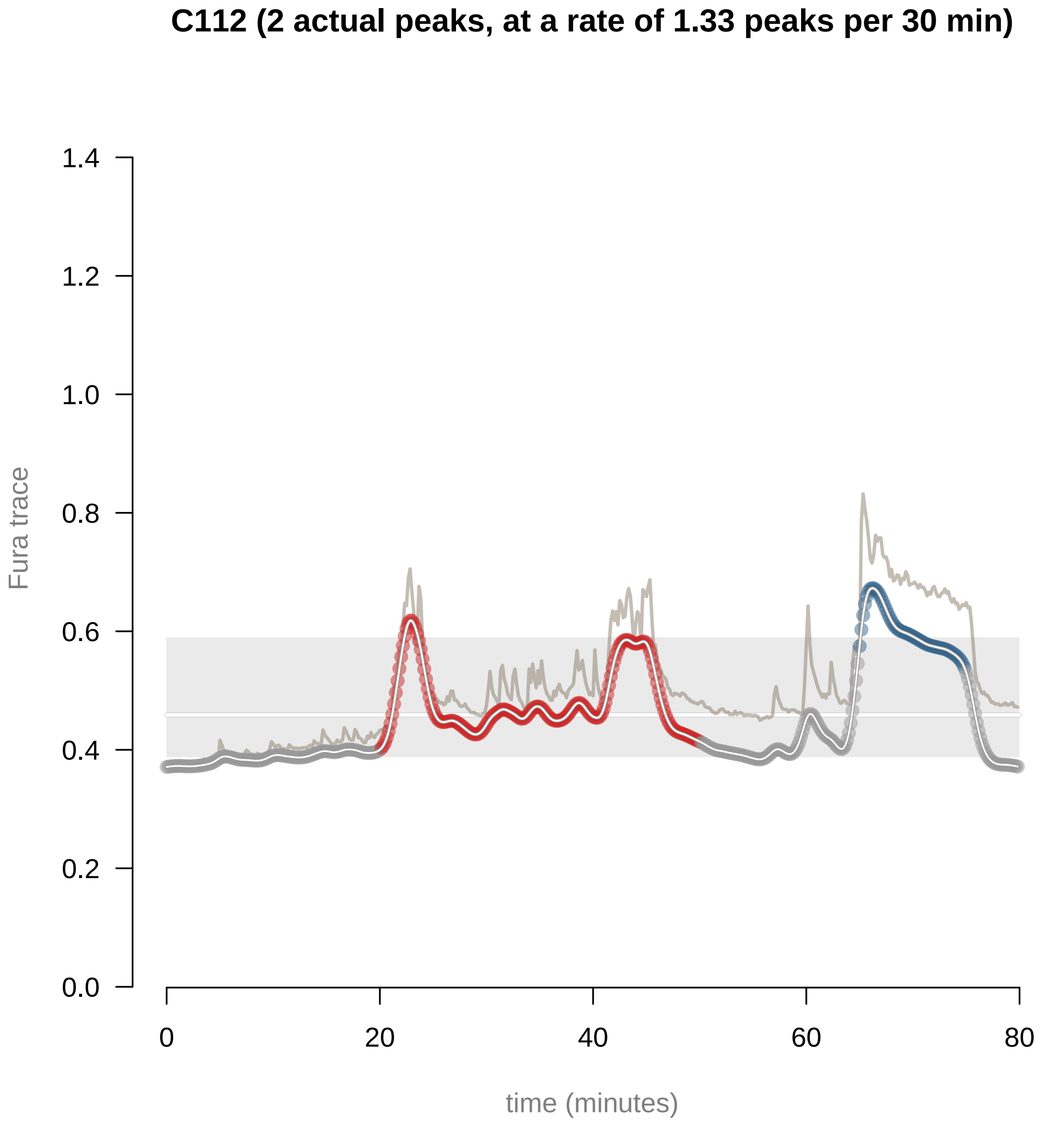
C112 (2 actual peaks, at a rate of 1.33 peaks per 30 min)
Fura trace
1.4
1.2
1.0
0.8
0.6
0.4
0.2
0.0
0
20
40
60
80
time (minutes)

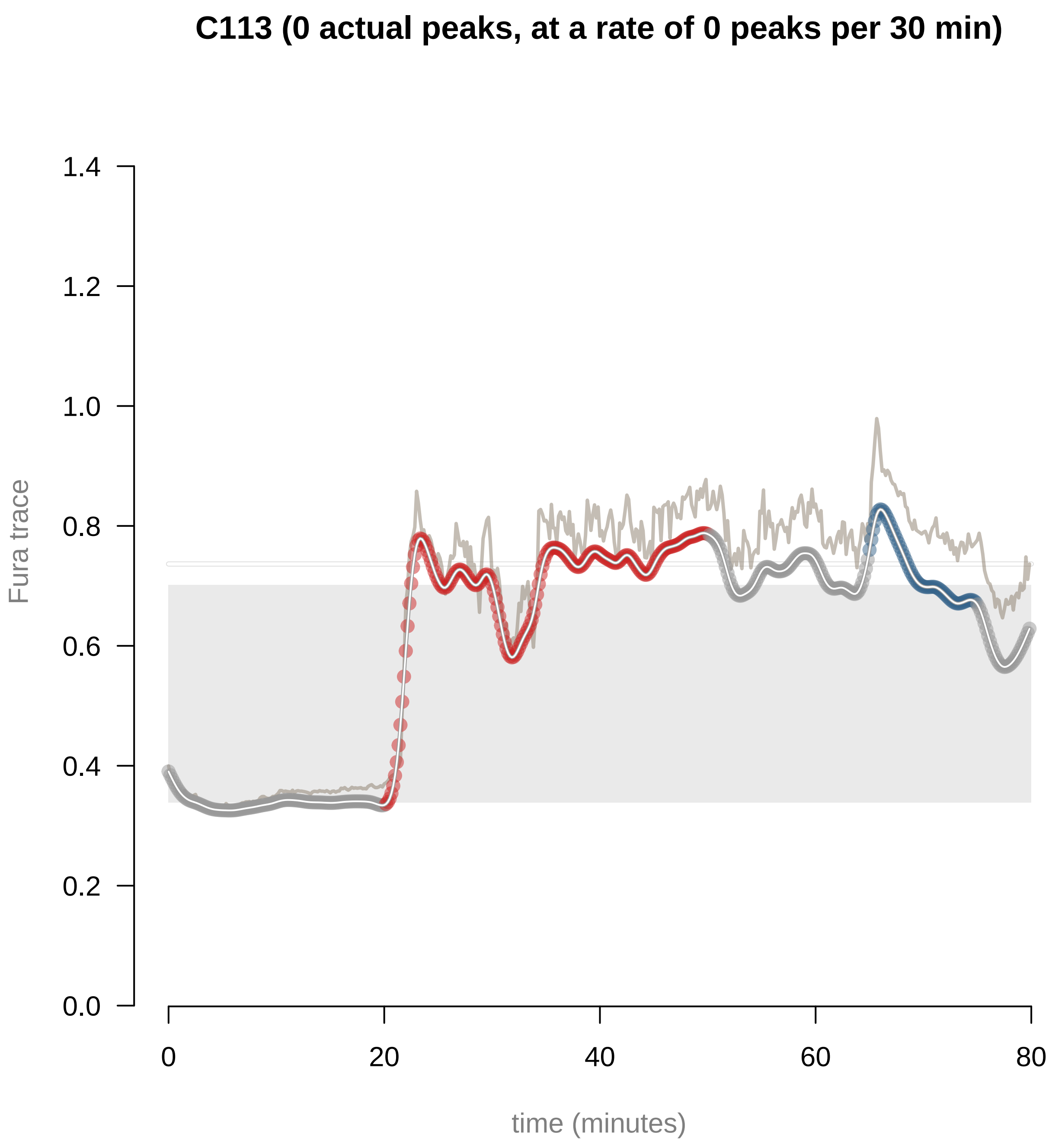
C113 (0 actual peaks, at a rate of 0 peaks per 30 min)
Fura trace
1.4
1.2
1.0
0.8
0.6
0.4
0.2
0.0
0
20
40
60
80
time (minutes)

C114 (3 actual peaks, at a rate of 2.79 peaks per 30 min)

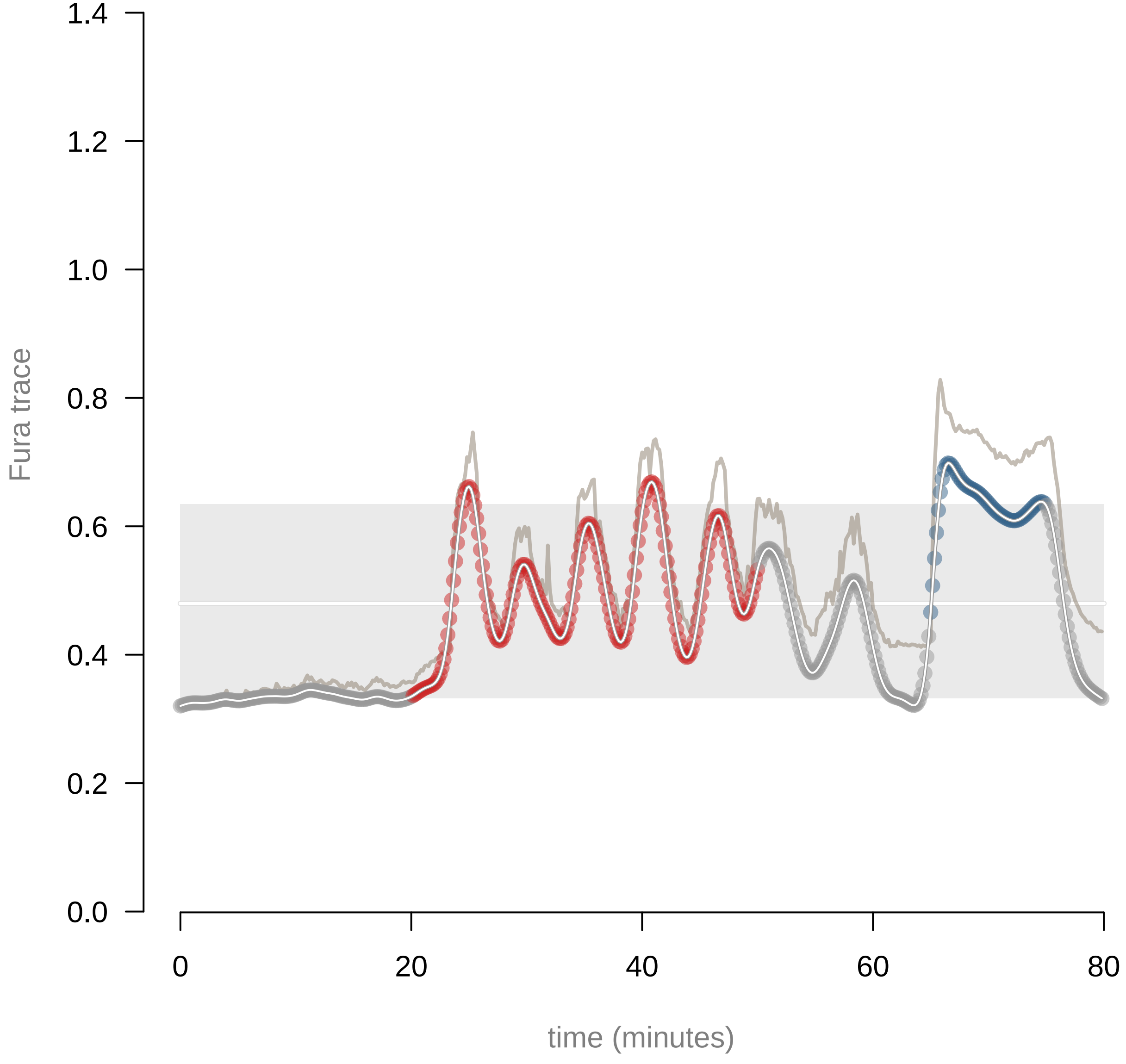

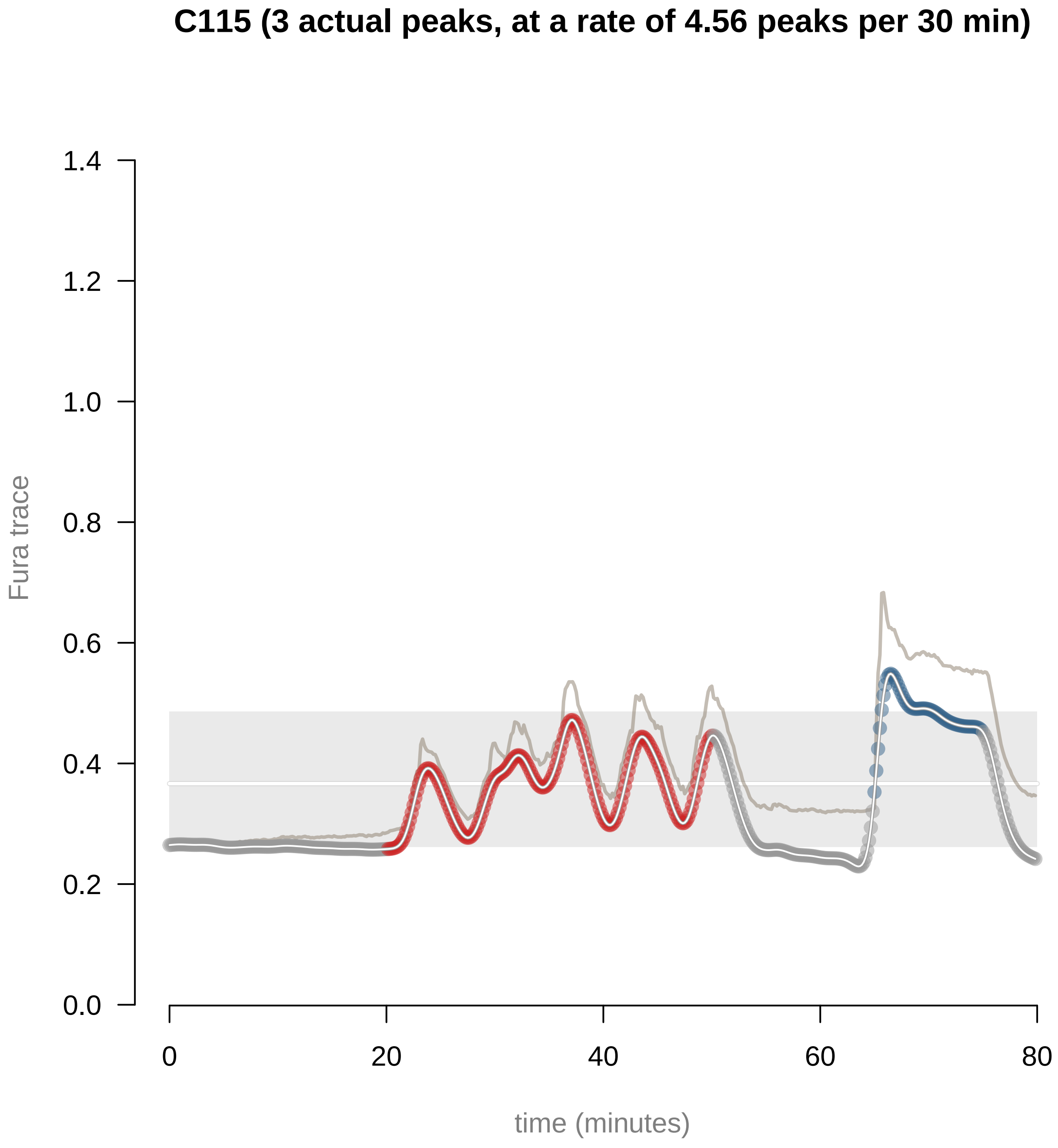

C115 (3 actual peaks, at a rate of 4.56 peaks per 30 min)
Fura trace
1.4
1.2
1.0
0.8
0.6
0.4
0.2
0.0
0
20
40
60
80
time (minutes)

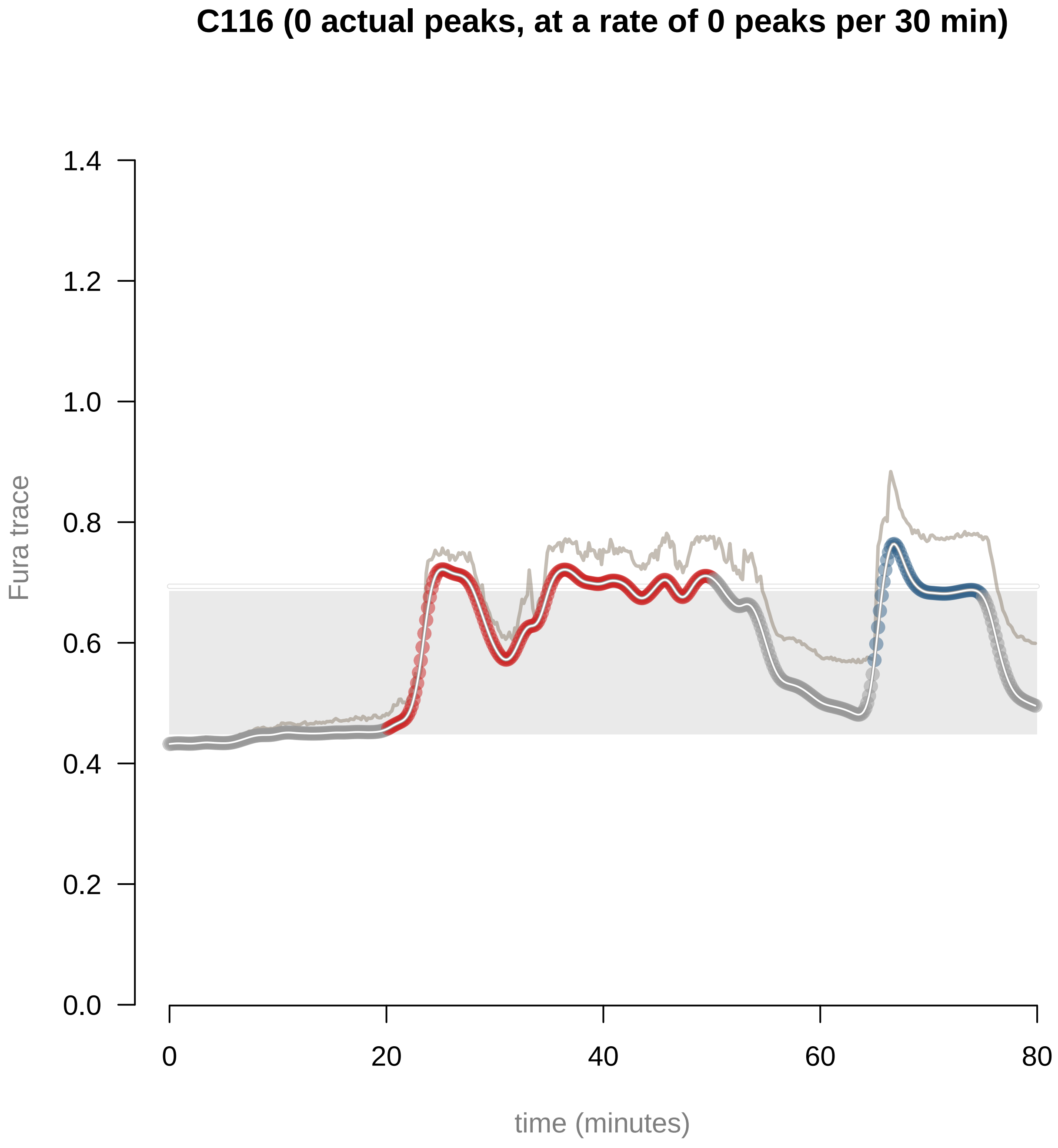

C116 (0 actual peaks, at a rate of 0 peaks per 30 min)
Fura trace
1.4
1.2
1.0
0.8
0.6
0.4
0.2
0.0
0
20
40
60
80
time (minutes)

C117 (0 actual peaks, at a rate of 0 peaks per 30 min)

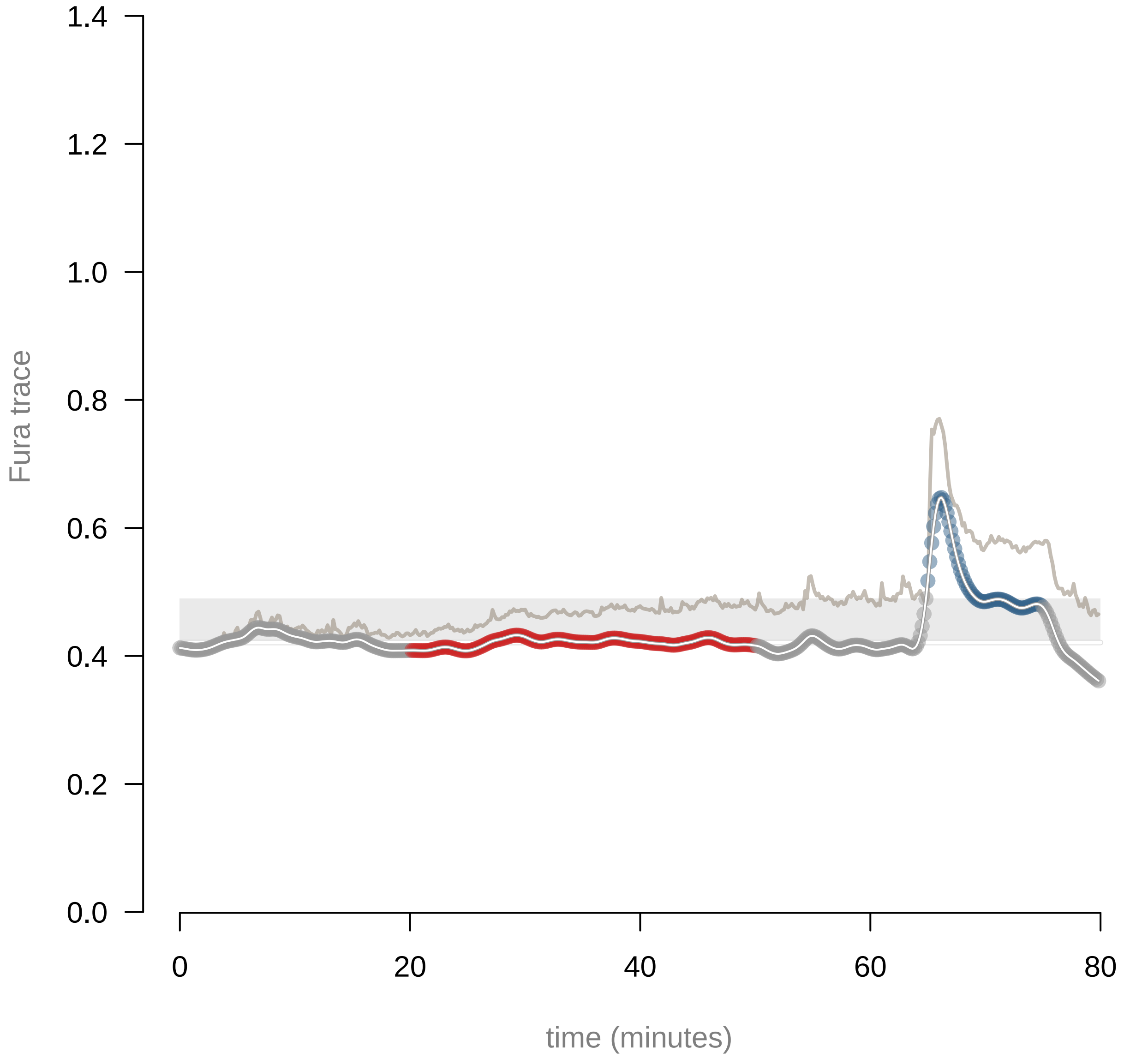

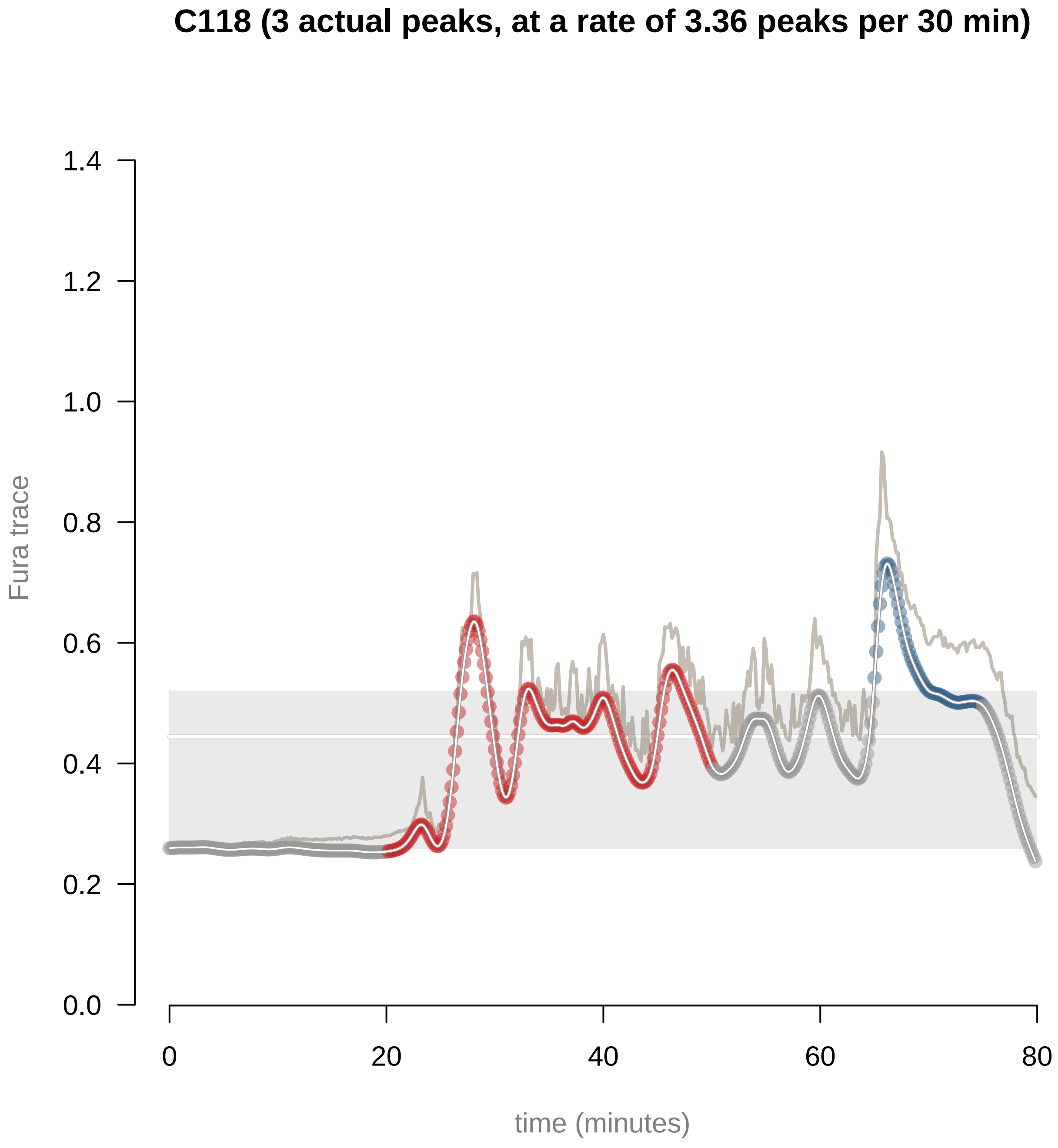

C118 (3 actual peaks, at a rate of 3.36 peaks per 30 min)
Fura trace
1.4
1.2
1.0
0.8
0.6
0.4
0.2
0.0
0
20
40
60
80
time (minutes)

C119 (1 actual peaks, at a rate of 1 peaks per 30 min)

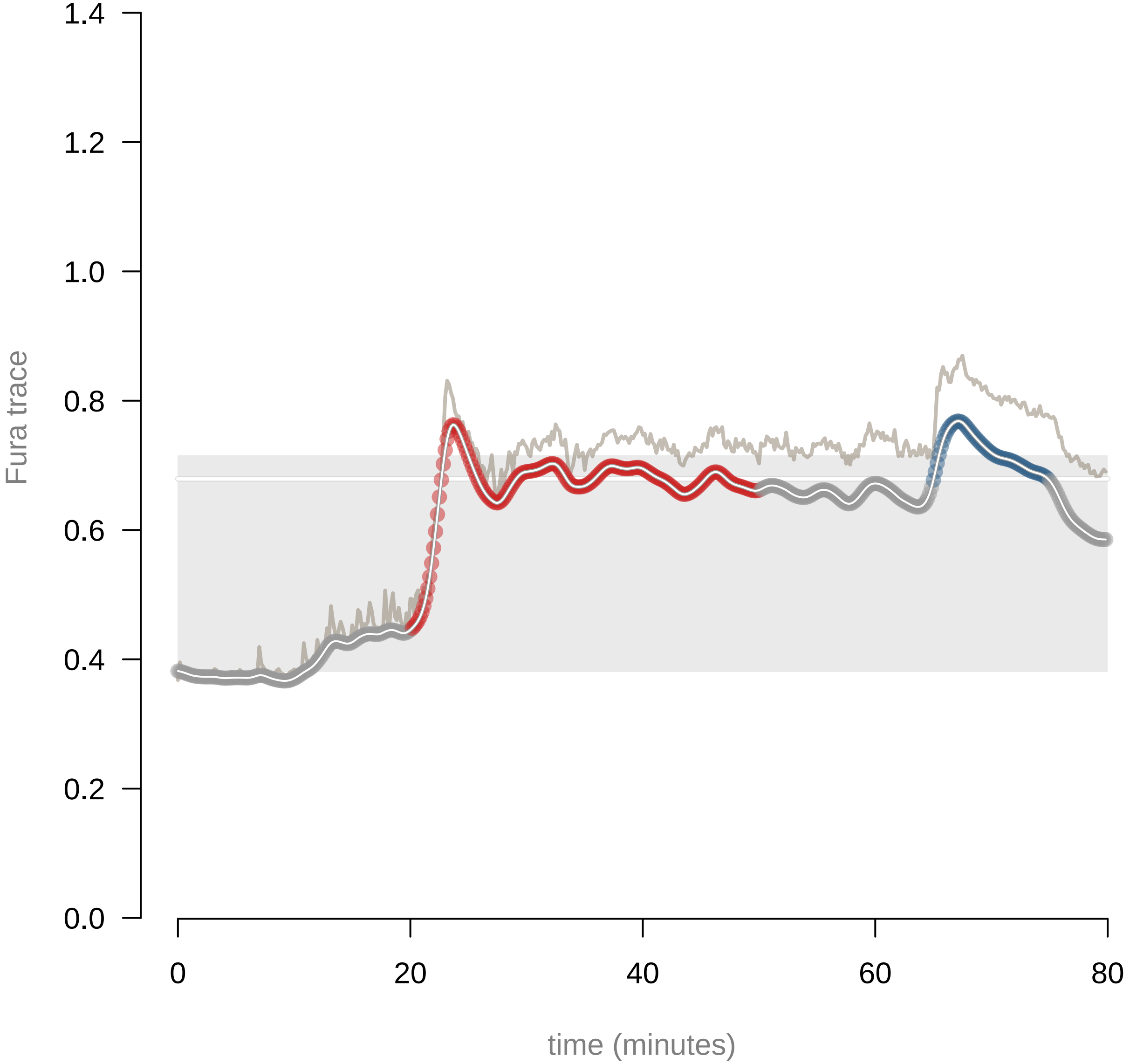

C120 (0 actual peaks, at a rate of 0 peaks per 30 min)

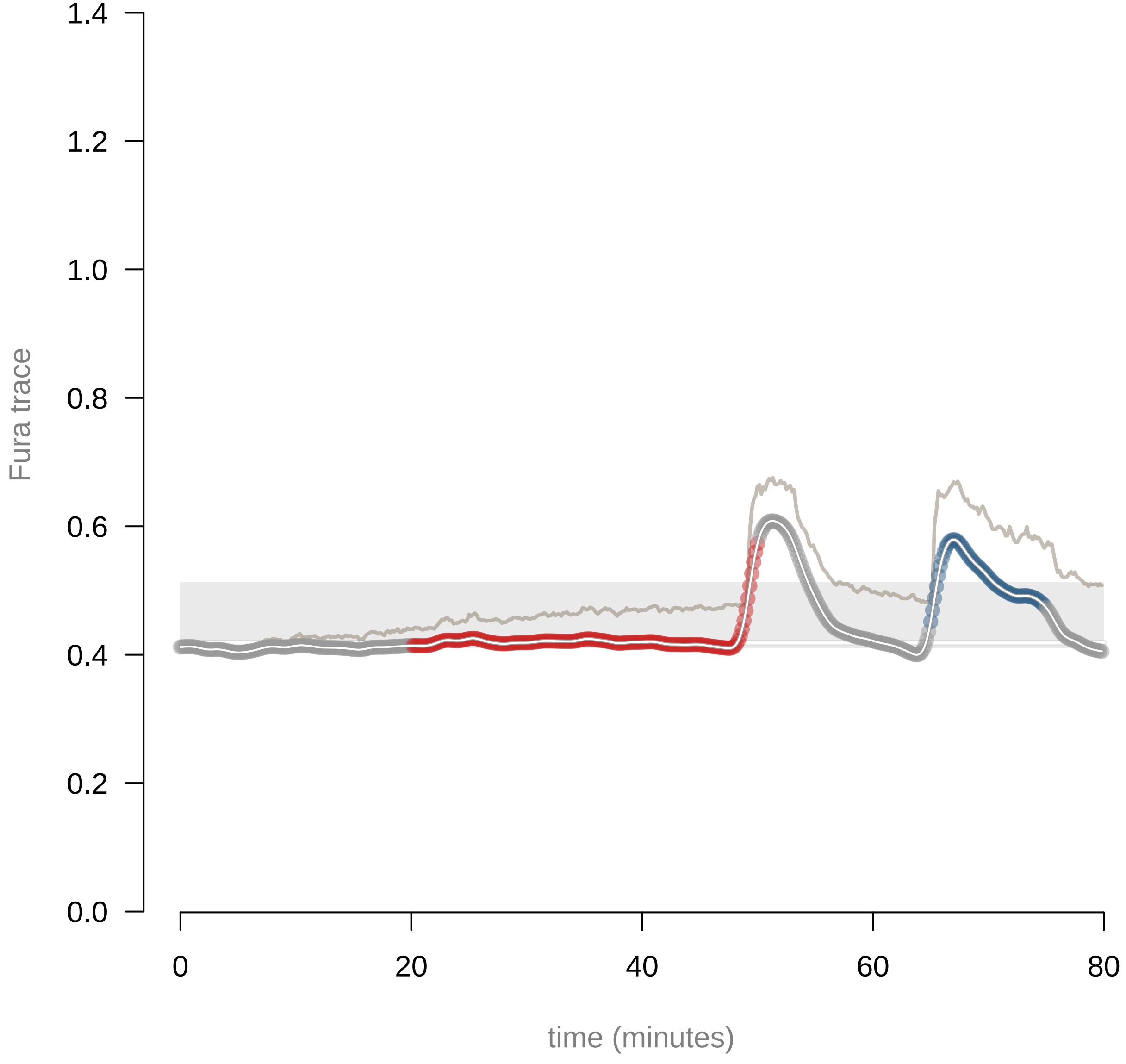

C121 (3 actual peaks, at a rate of 2.88 peaks per 30 min)

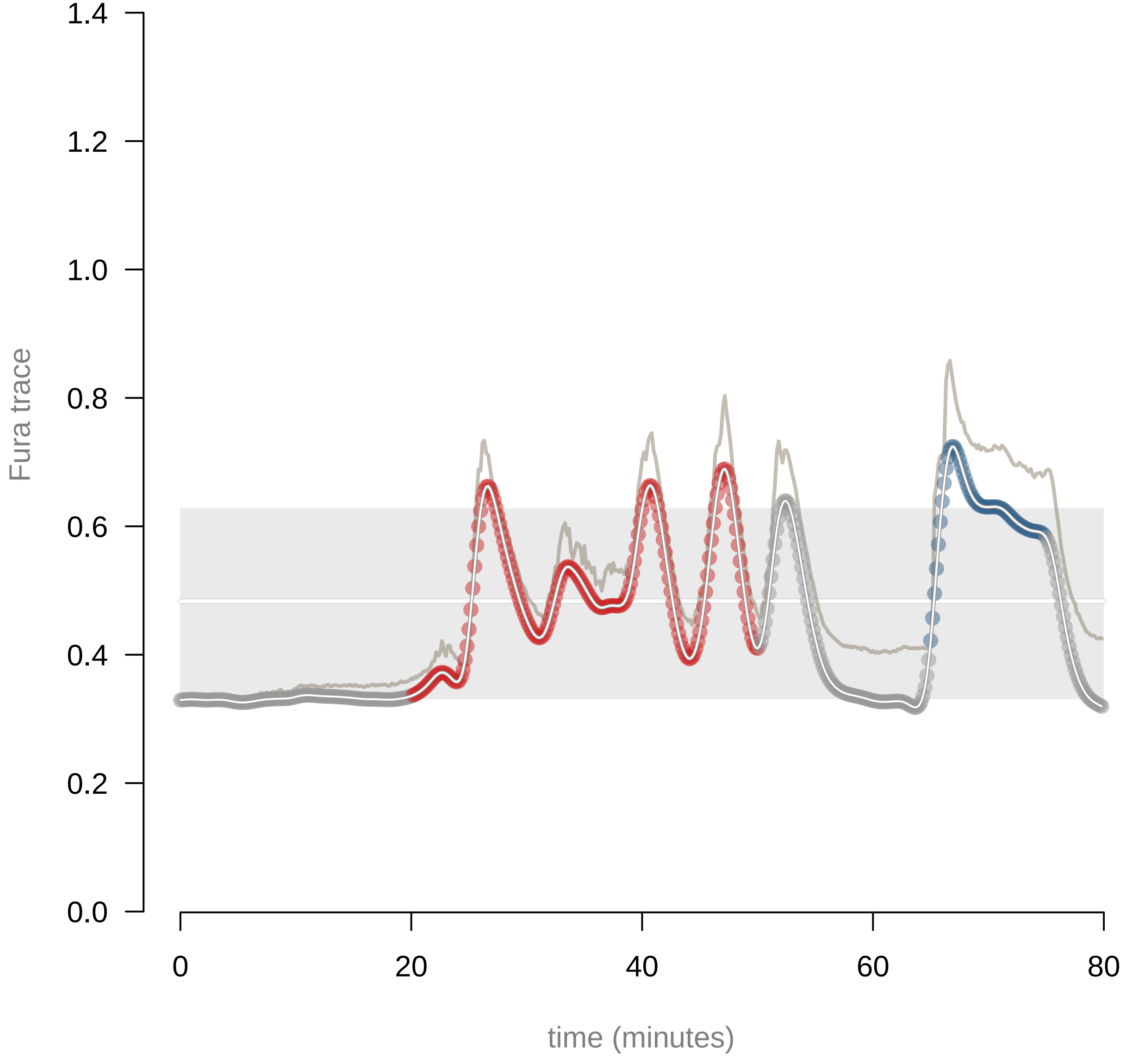

C122 (0 actual peaks, at a rate of 0 peaks per 30 min)

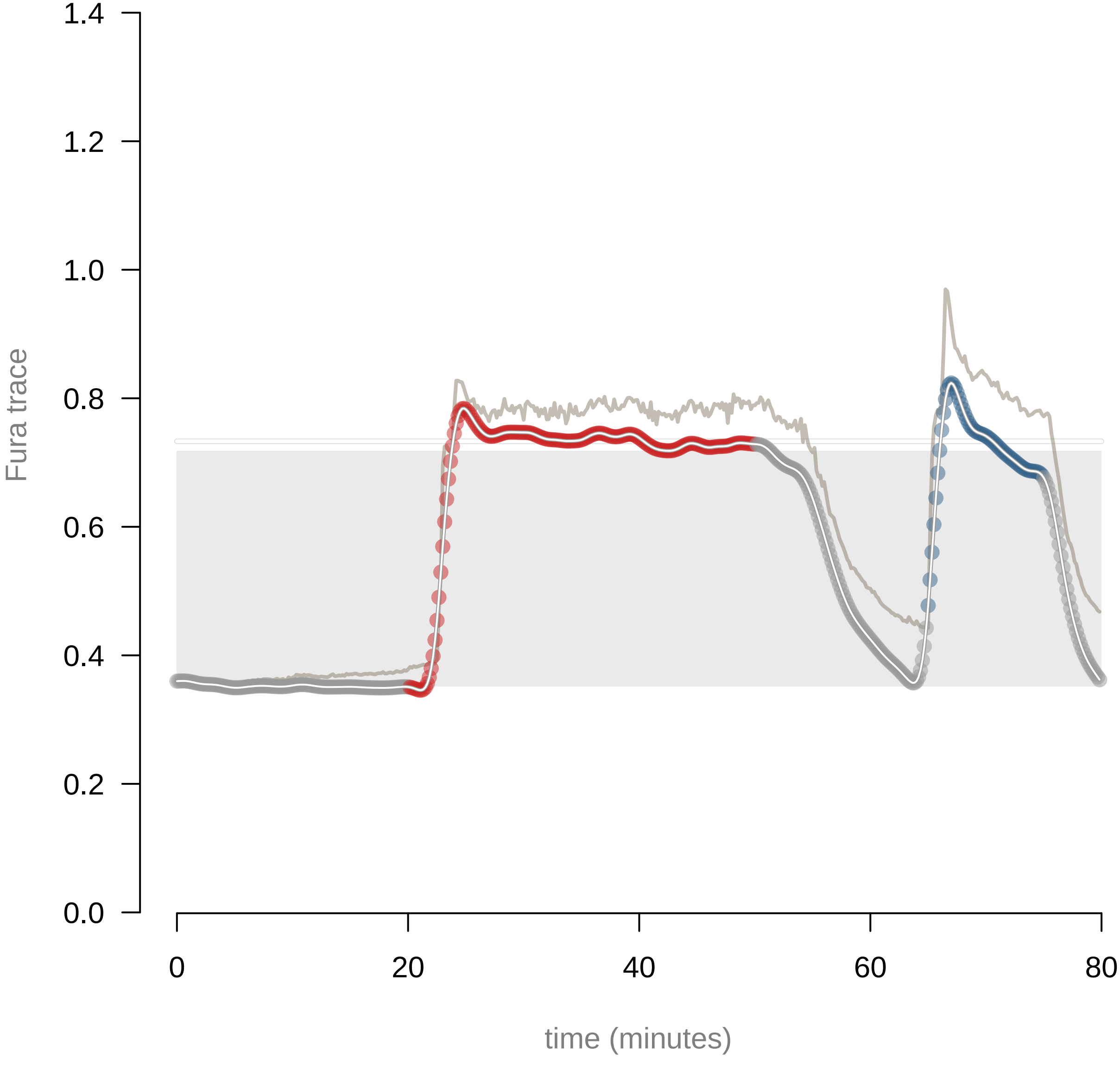

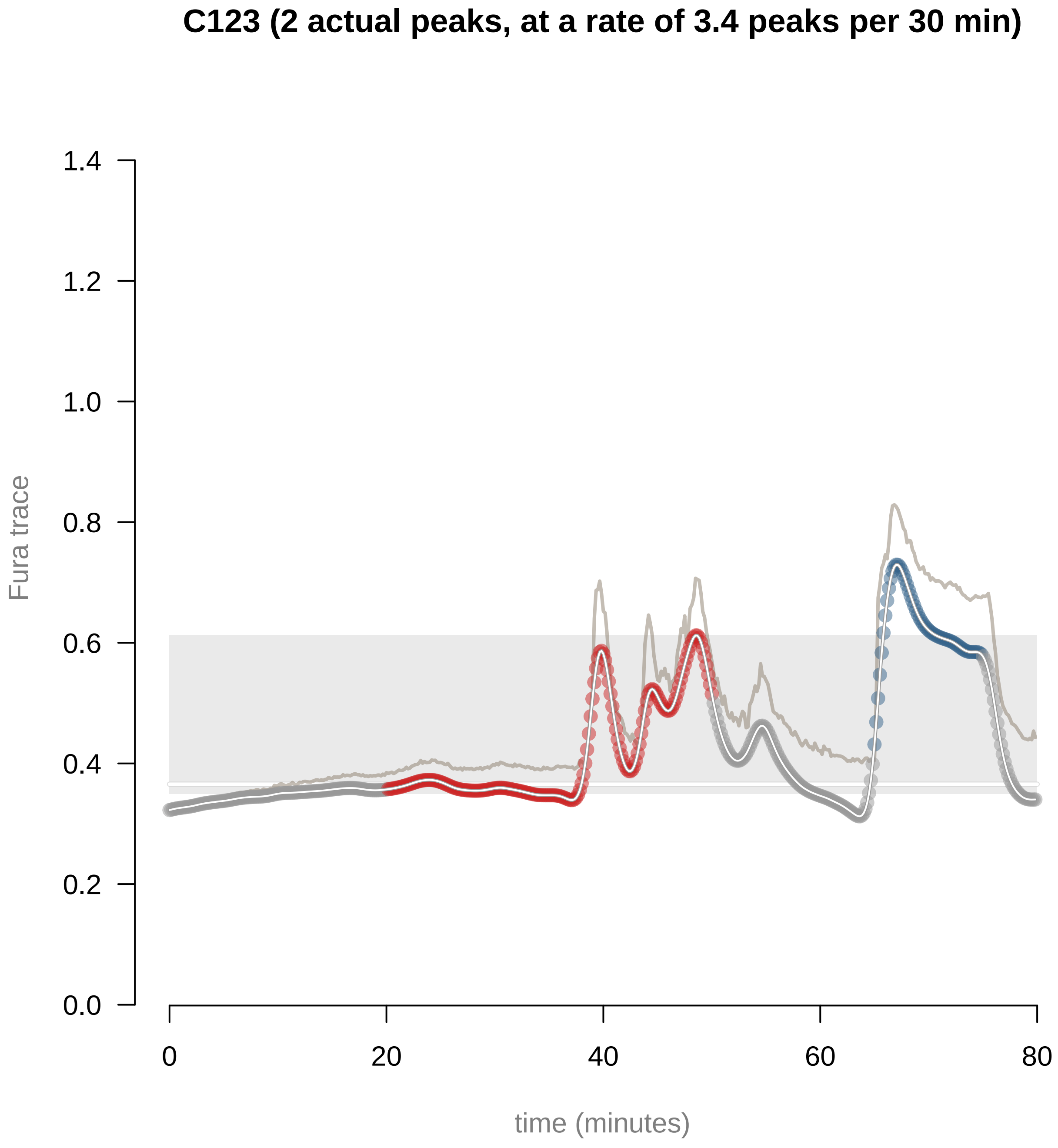

C123 (2 actual peaks, at a rate of 3.4 peaks per 30 min)
Fura trace
1.4
1.2
1.0
0.8
0.6
0.4
0.2
0.0
0
20
40
60
80
time (minutes)

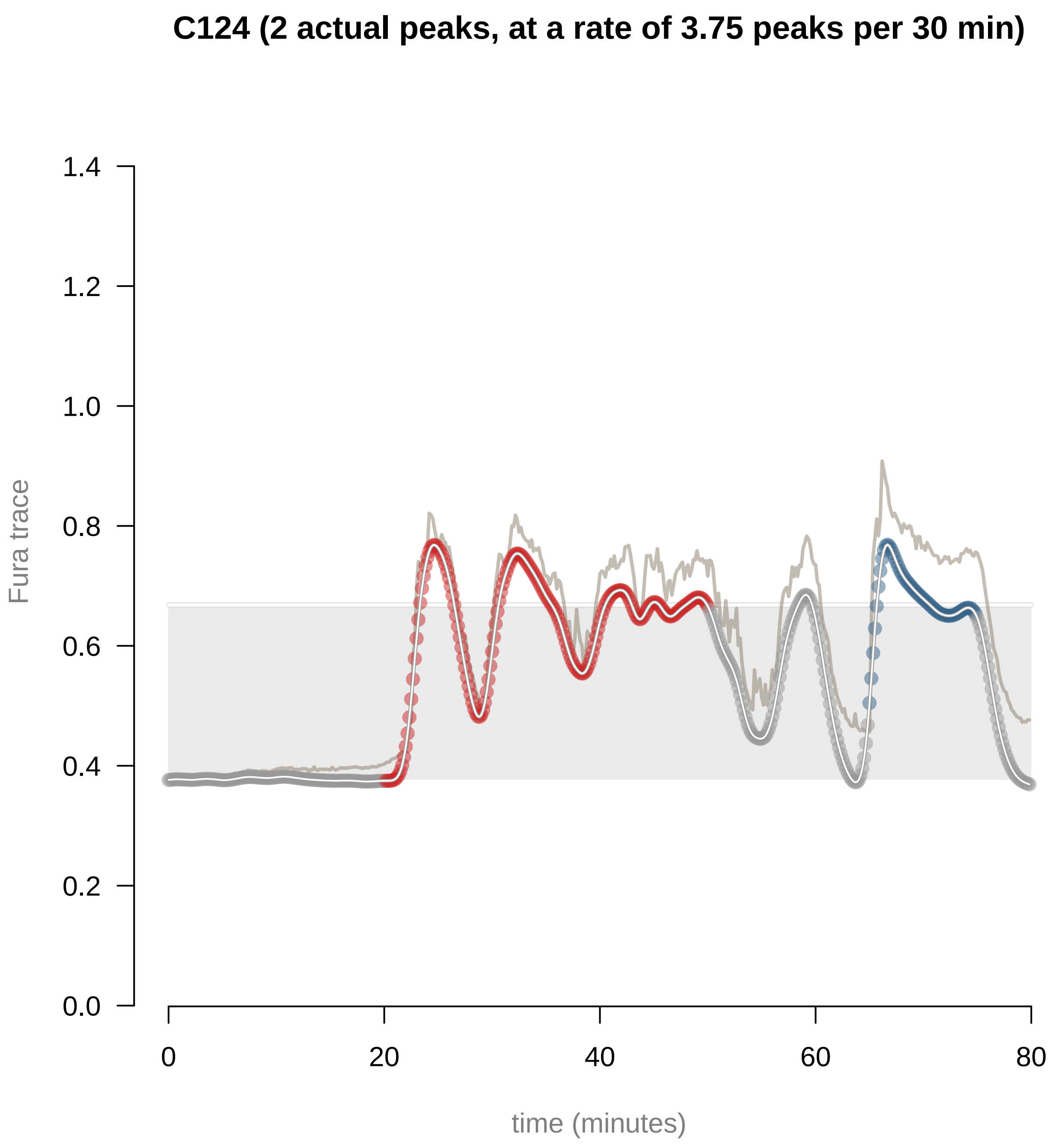
C124 (2 actual peaks, at a rate of 3.75 peaks per 30 min)
Fura trace
1.4
1.2
1.0
0.8
0.6
0.4
0.2
0.0
0
20
40
60
80
time (minutes)

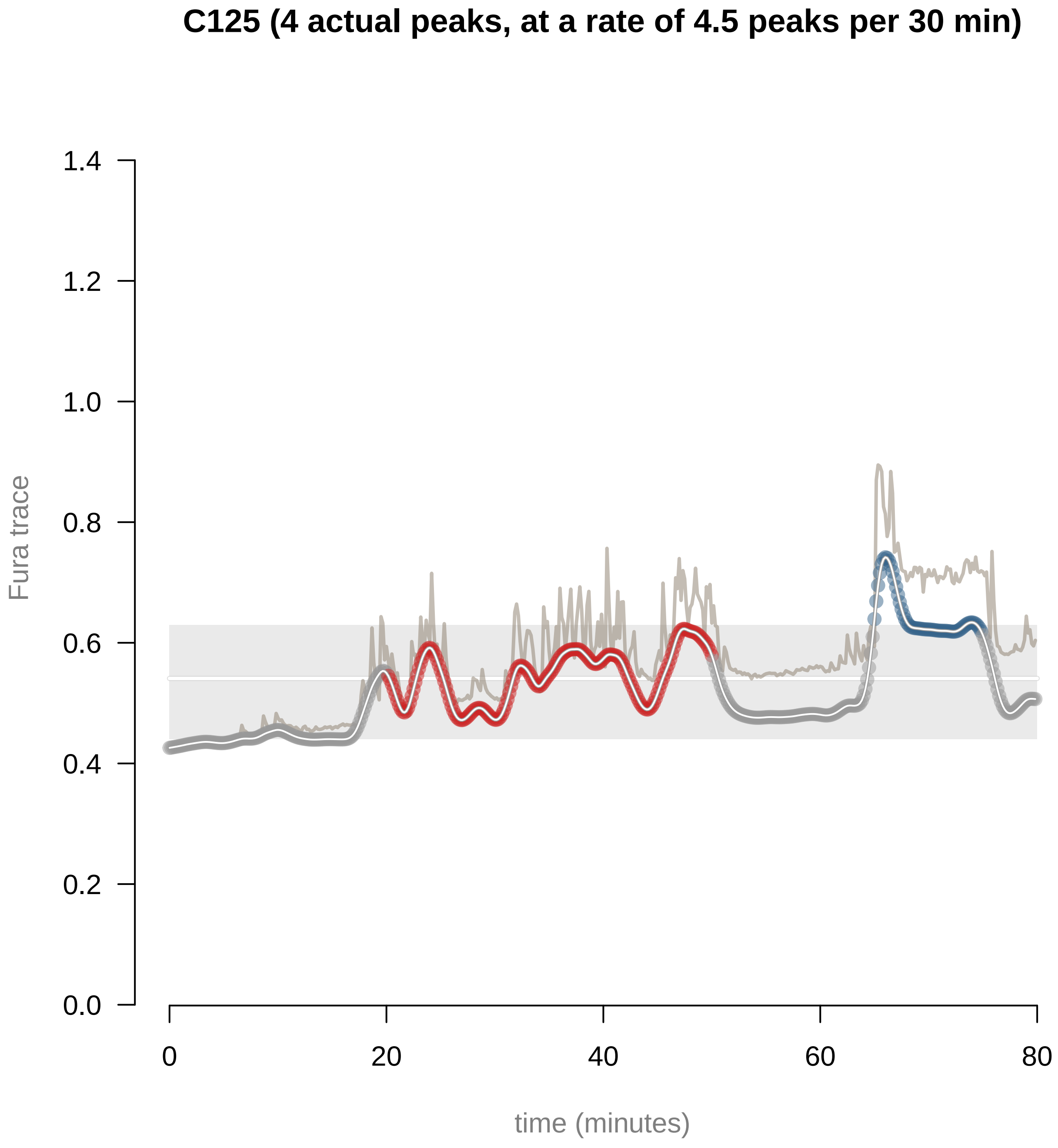

C125 (4 actual peaks, at a rate of 4.5 peaks per 30 min)
Fura trace
1.4
1.2
1.0
0.8
0.6
0.4
0.2
0.0
0
20
40
60
80
time (minutes)

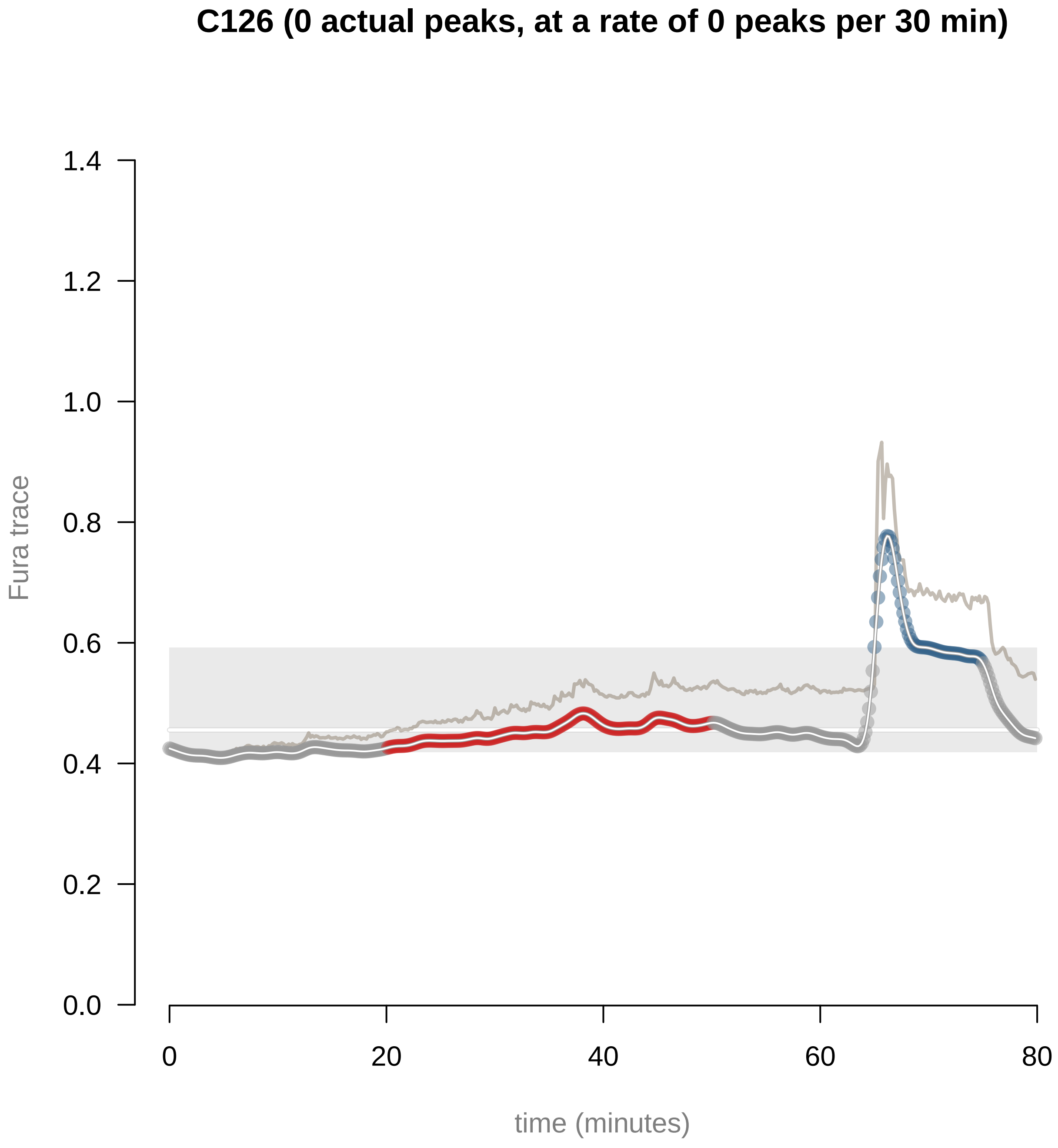

C126 (0 actual peaks, at a rate of 0 peaks per 30 min)
Fura trace
time (minutes)
0.0
0.2
0.4
0.6
0.8
1.0
1.2
1.4
0
20
40
60
80

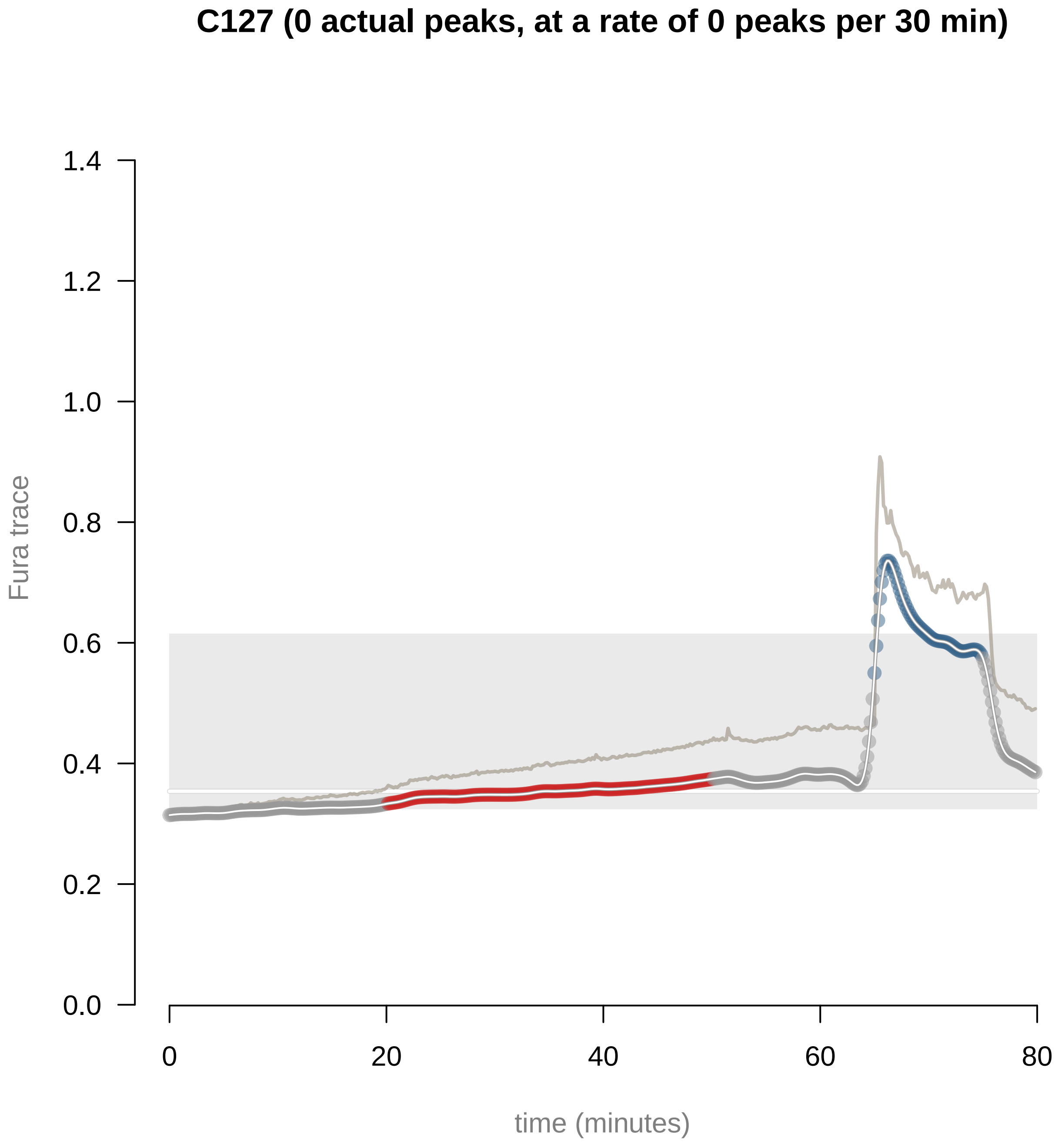

C127 (0 actual peaks, at a rate of 0 peaks per 30 min)
Fura trace
time (minutes)
0.0
0.2
0.4
0.6
0.8
1.0
1.2
1.4
0
20
40
60
80

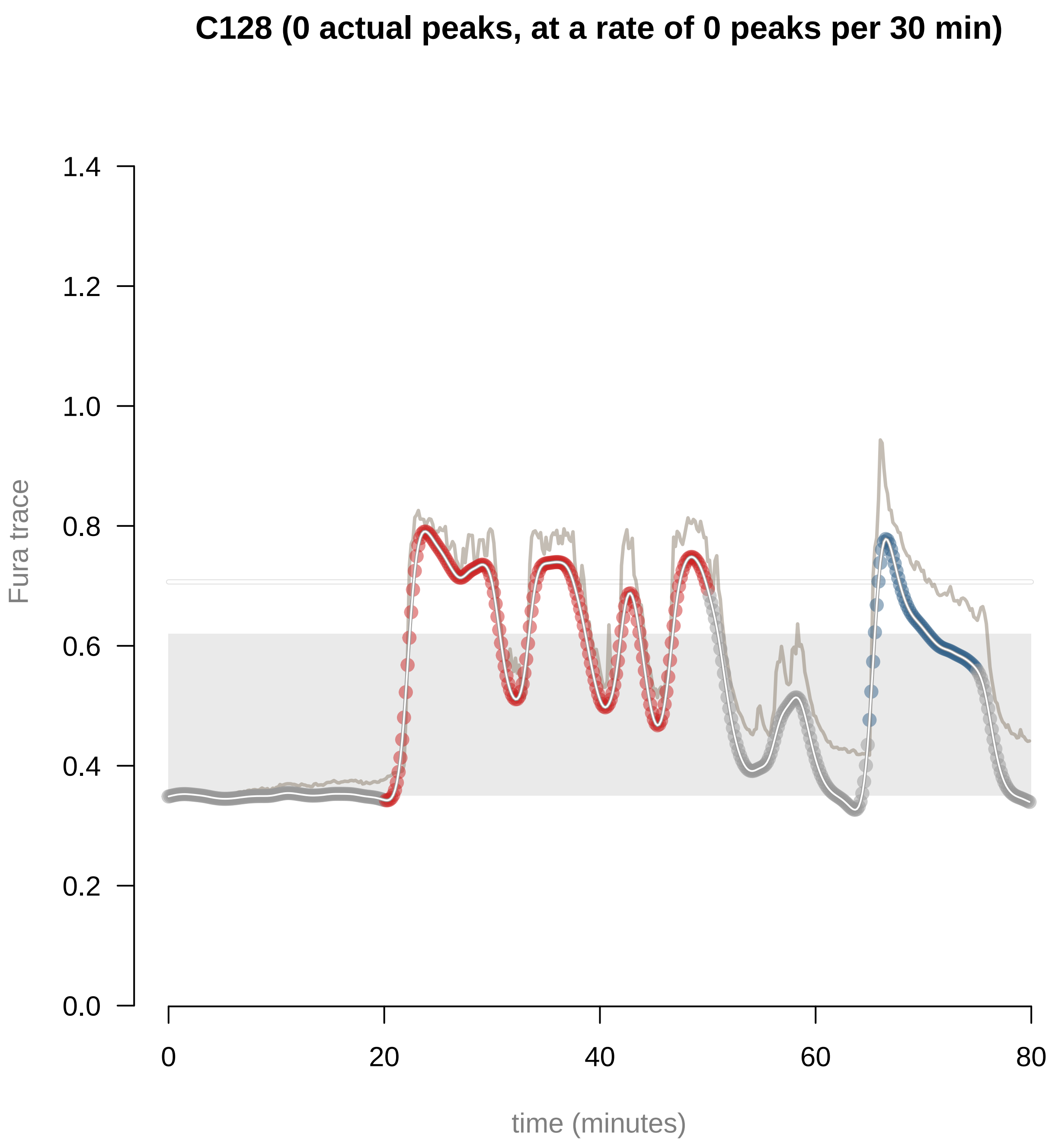

C128 (0 actual peaks, at a rate of 0 peaks per 30 min)
Fura trace
time (minutes)
0.0
0.2
0.4
0.6
0.8
1.0
1.2
1.4
0
20
40
60
80

C129 (0 actual peaks, at a rate of 0 peaks per 30 min)

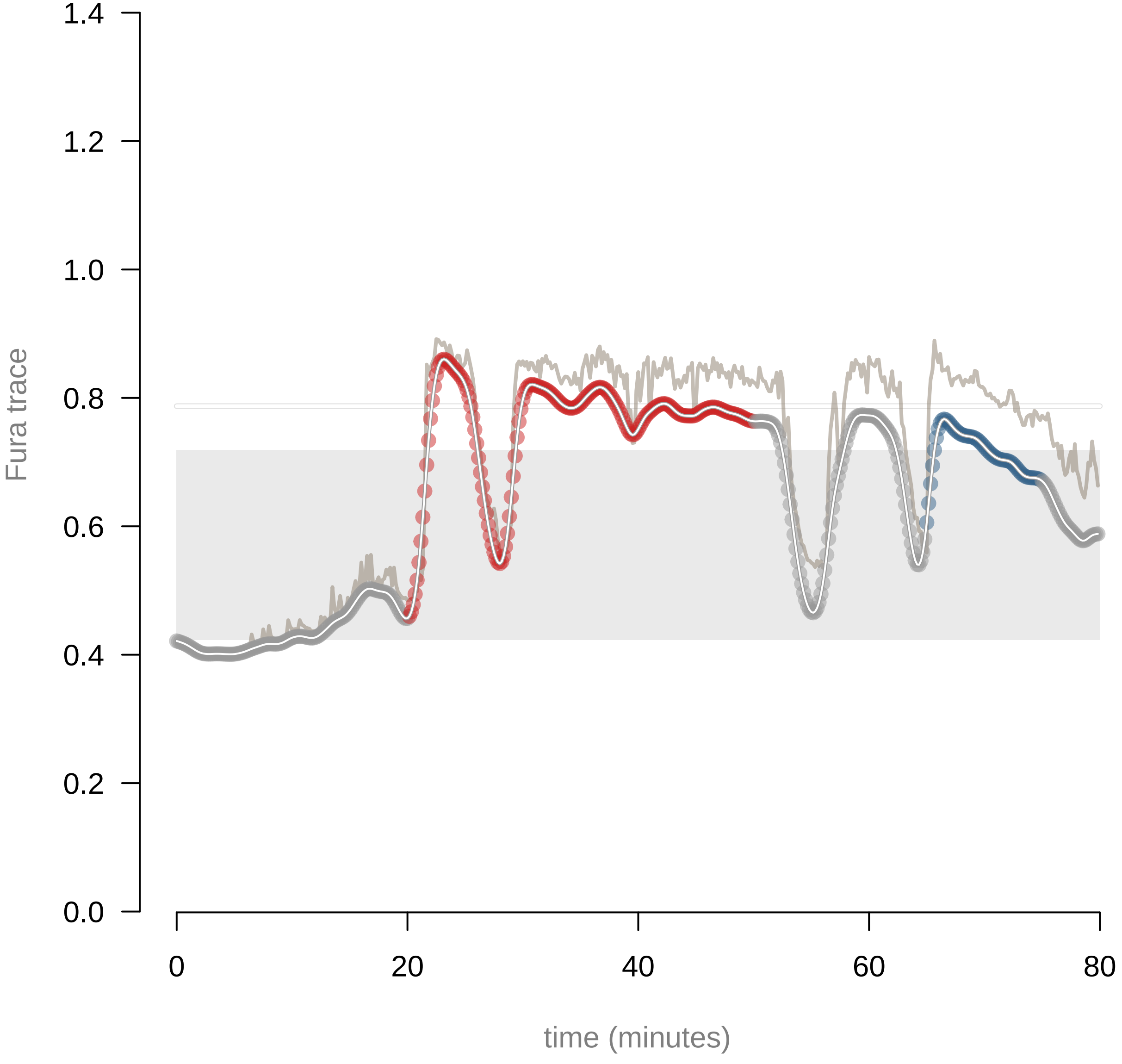

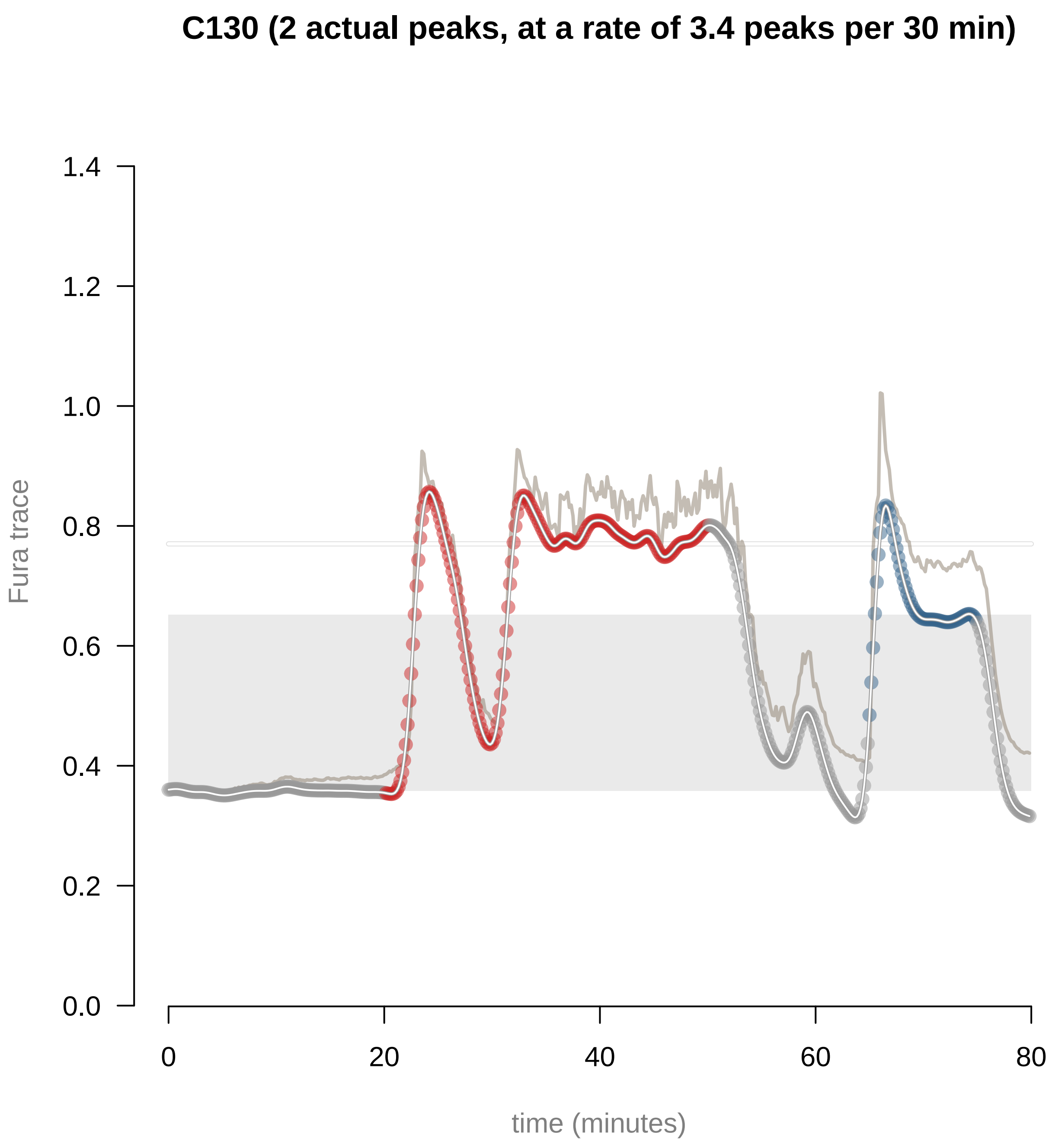
C130 (2 actual peaks, at a rate of 3.4 peaks per 30 min)
Fura trace
time (minutes)
0.0
0.2
0.4
0.6
0.8
1.0
1.2
1.4
0
20
40
60
80

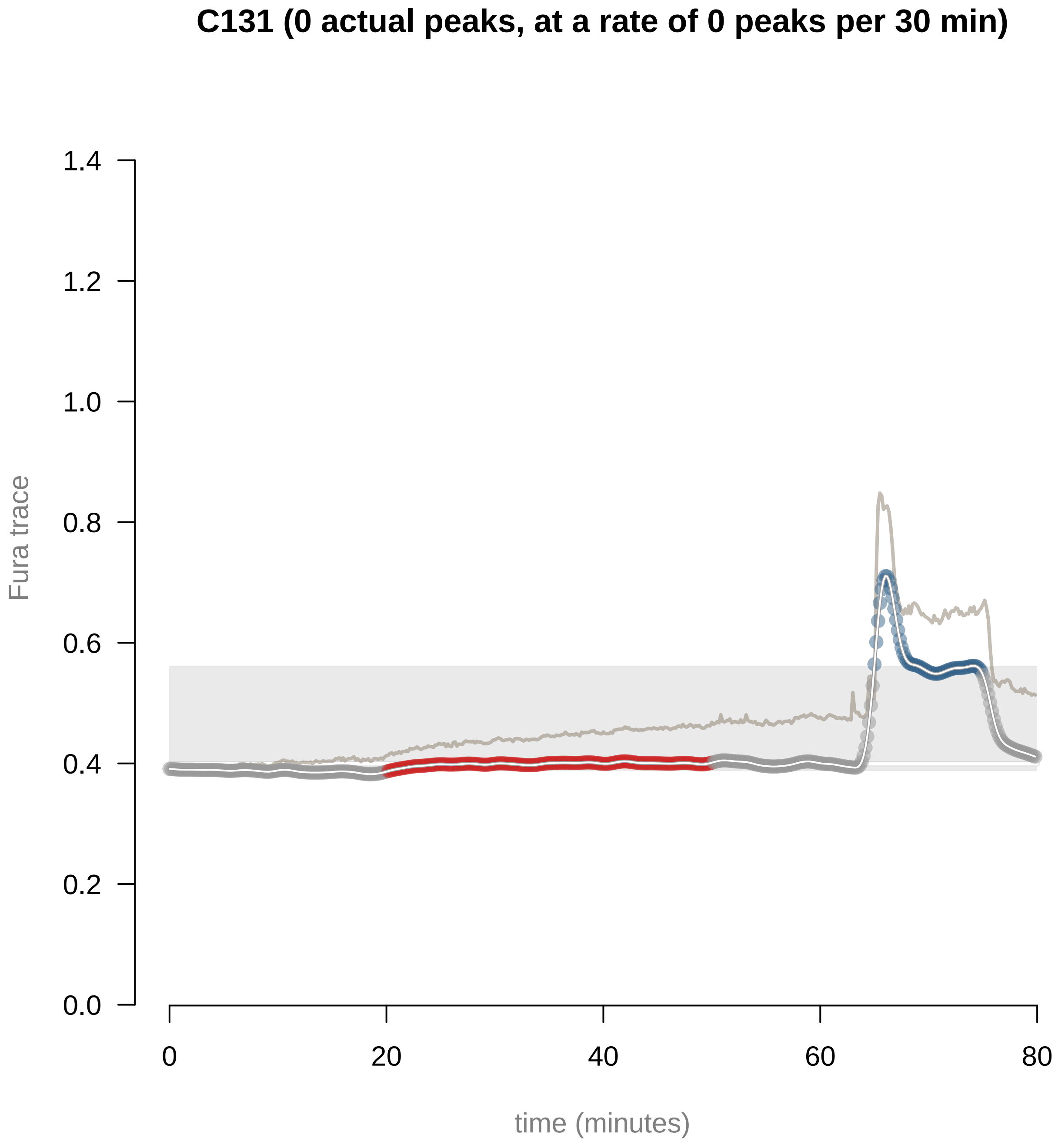
C131 (0 actual peaks, at a rate of 0 peaks per 30 min)
Fura trace
time (minutes)
0.0
0.2
0.4
0.6
0.8
1.0
1.2
1.4
0
20
40
60
80

C132 (3 actual peaks, at a rate of 2.88 peaks per 30 min)

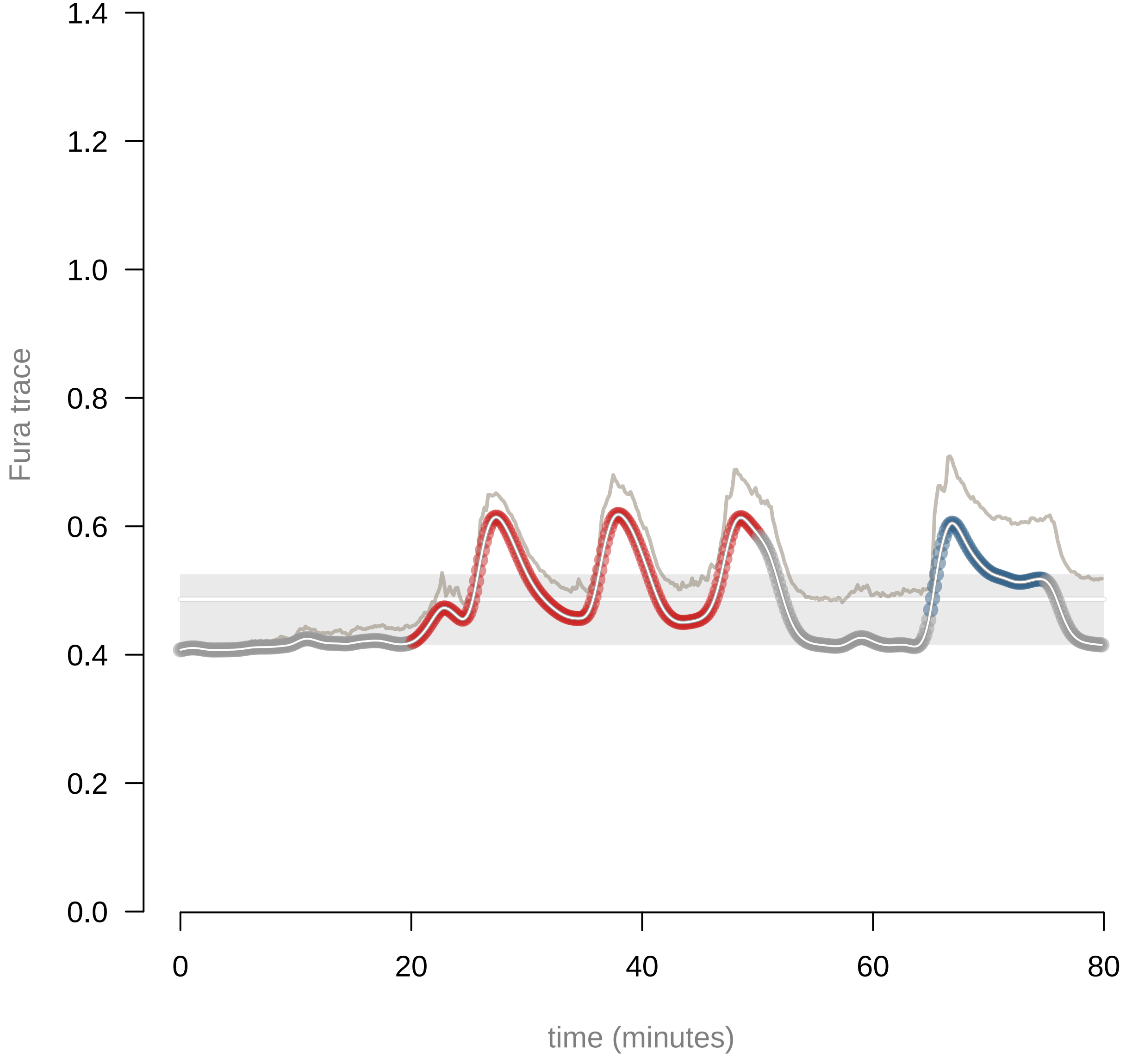

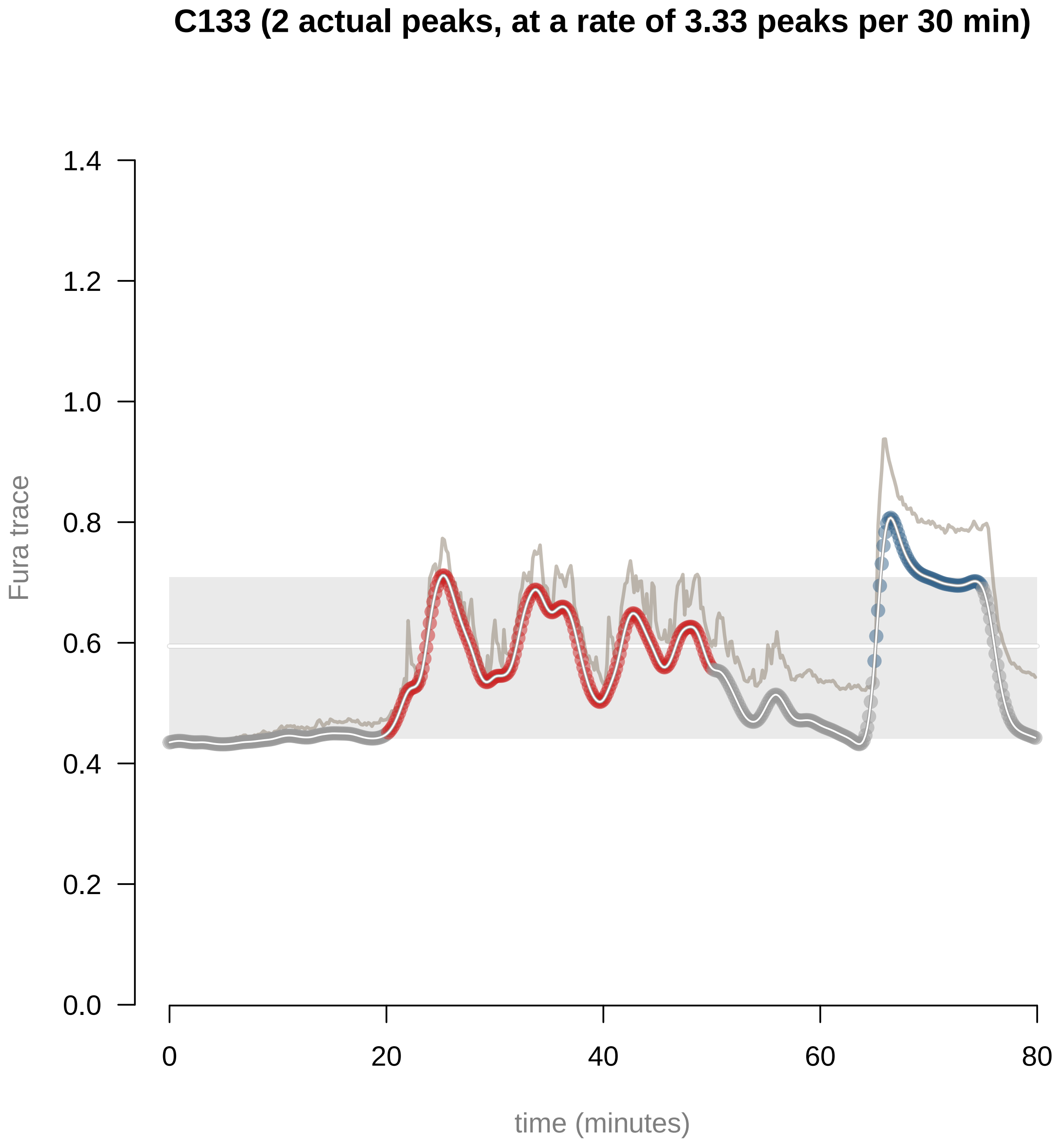

C133 (2 actual peaks, at a rate of 3.33 peaks per 30 min)
1.4
1.2
1.0
0.8
0.6
0.4
0.2
0.0
Fura trace
0
20
40
60
80
time (minutes)

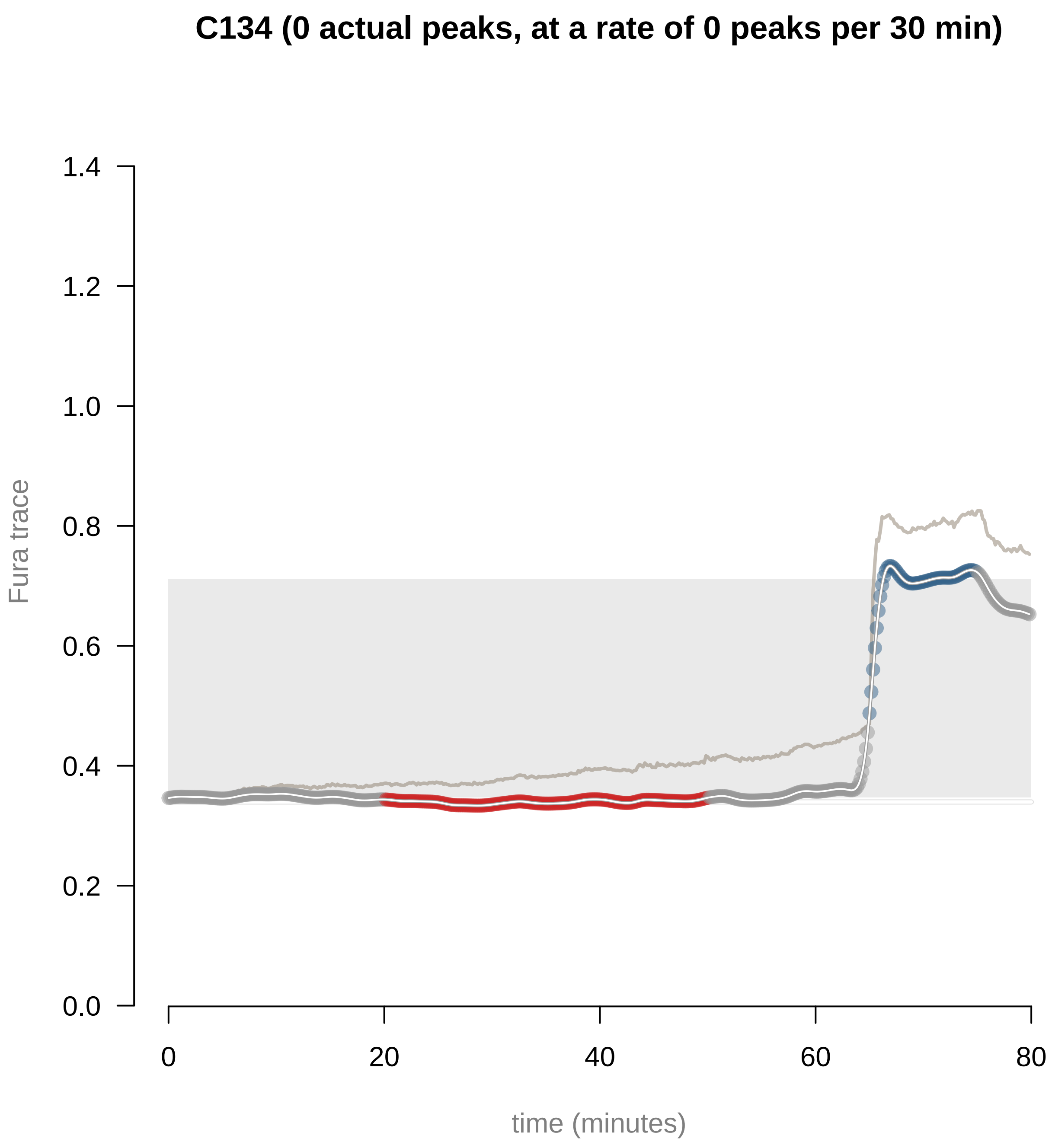
C134 (0 actual peaks, at a rate of 0 peaks per 30 min)
Fura trace
1.4
1.2
1.0
0.8
0.6
0.4
0.2
0.0
0
20
40
60
80
time (minutes)

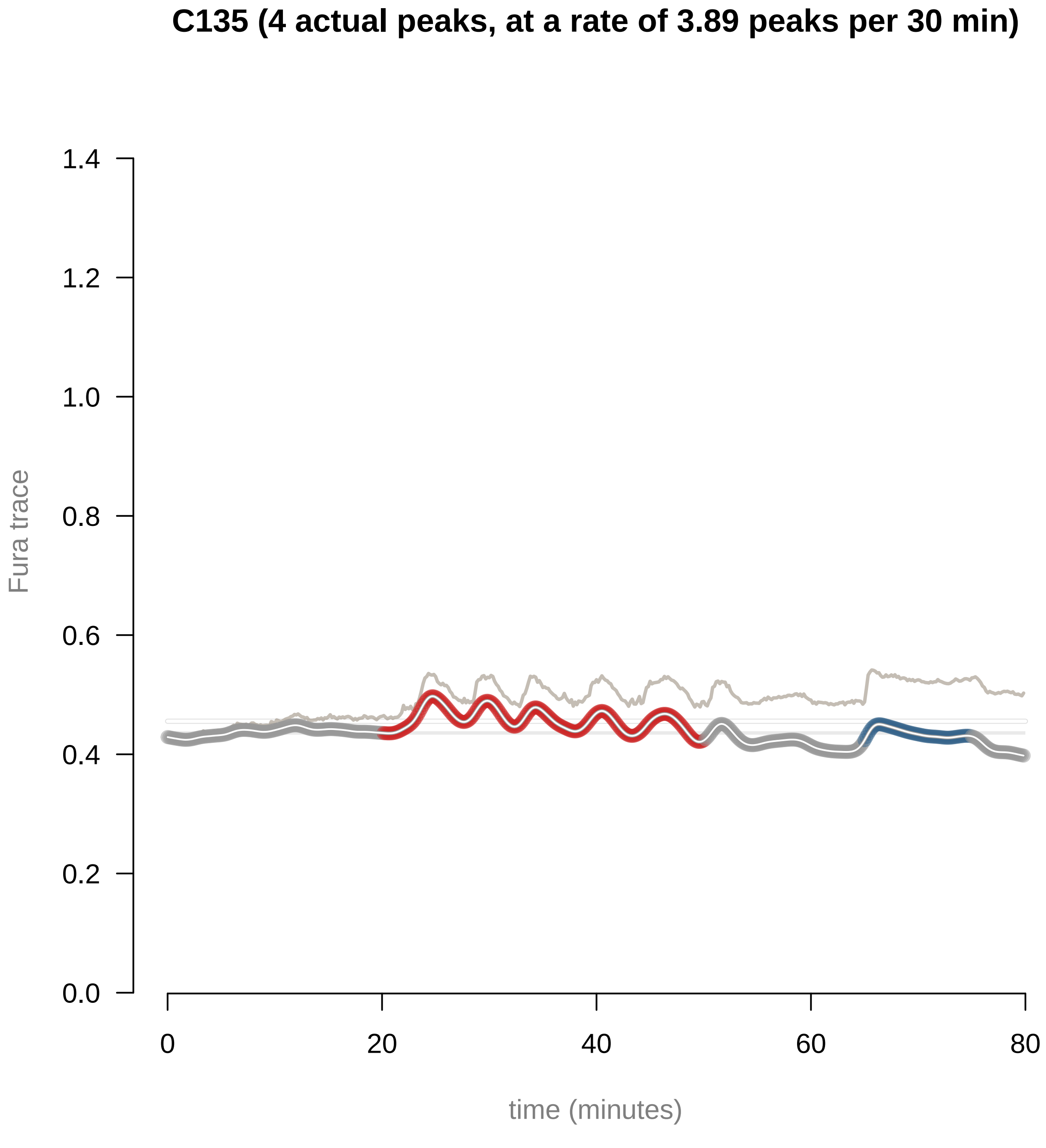

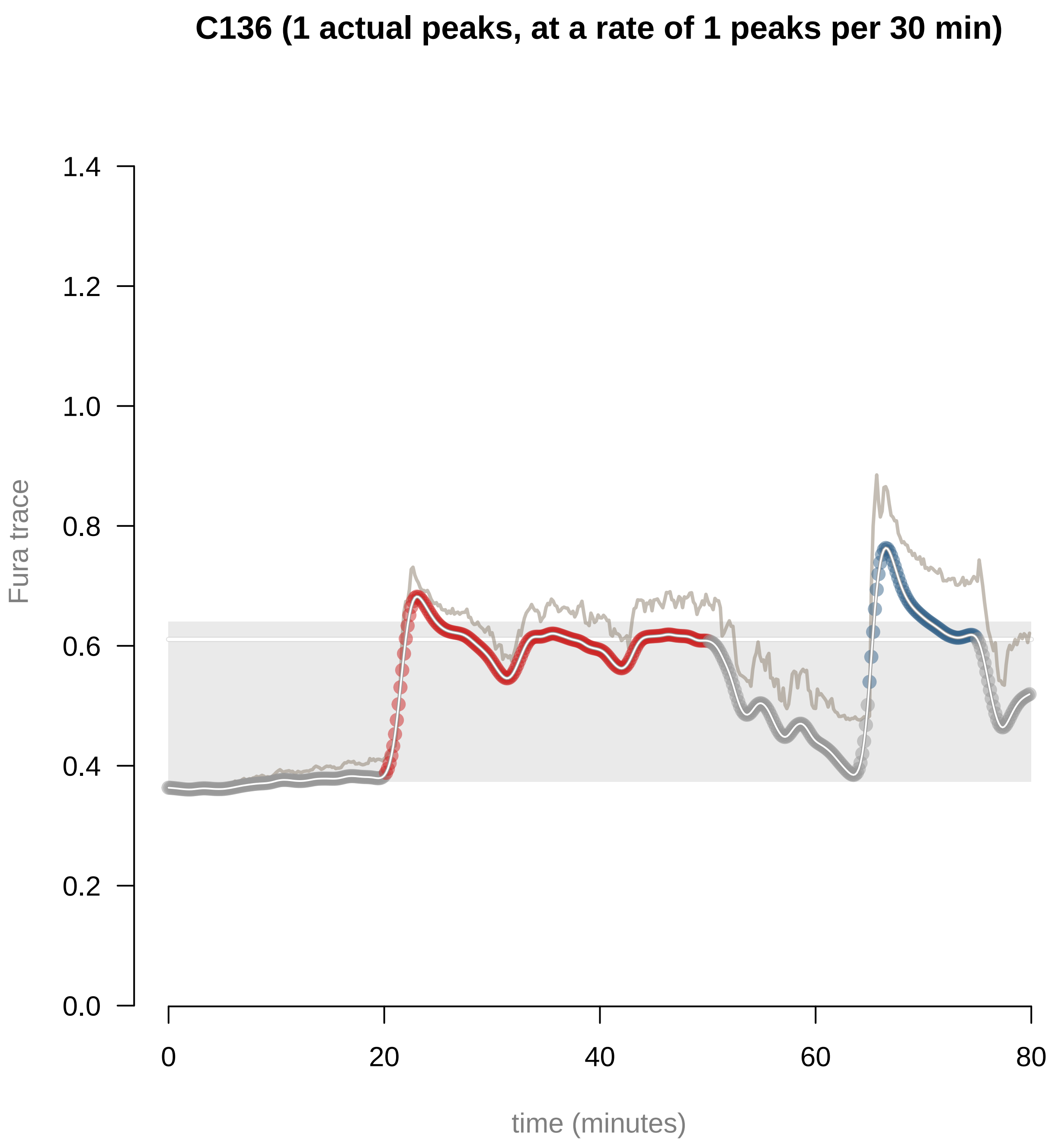

C136 (1 actual peaks, at a rate of 1 peaks per 30 min)
1.4
1.2
1.0
0.8
0.6
0.4
0.2
0.0
Fura trace
0
20
40
60
80
time (minutes)

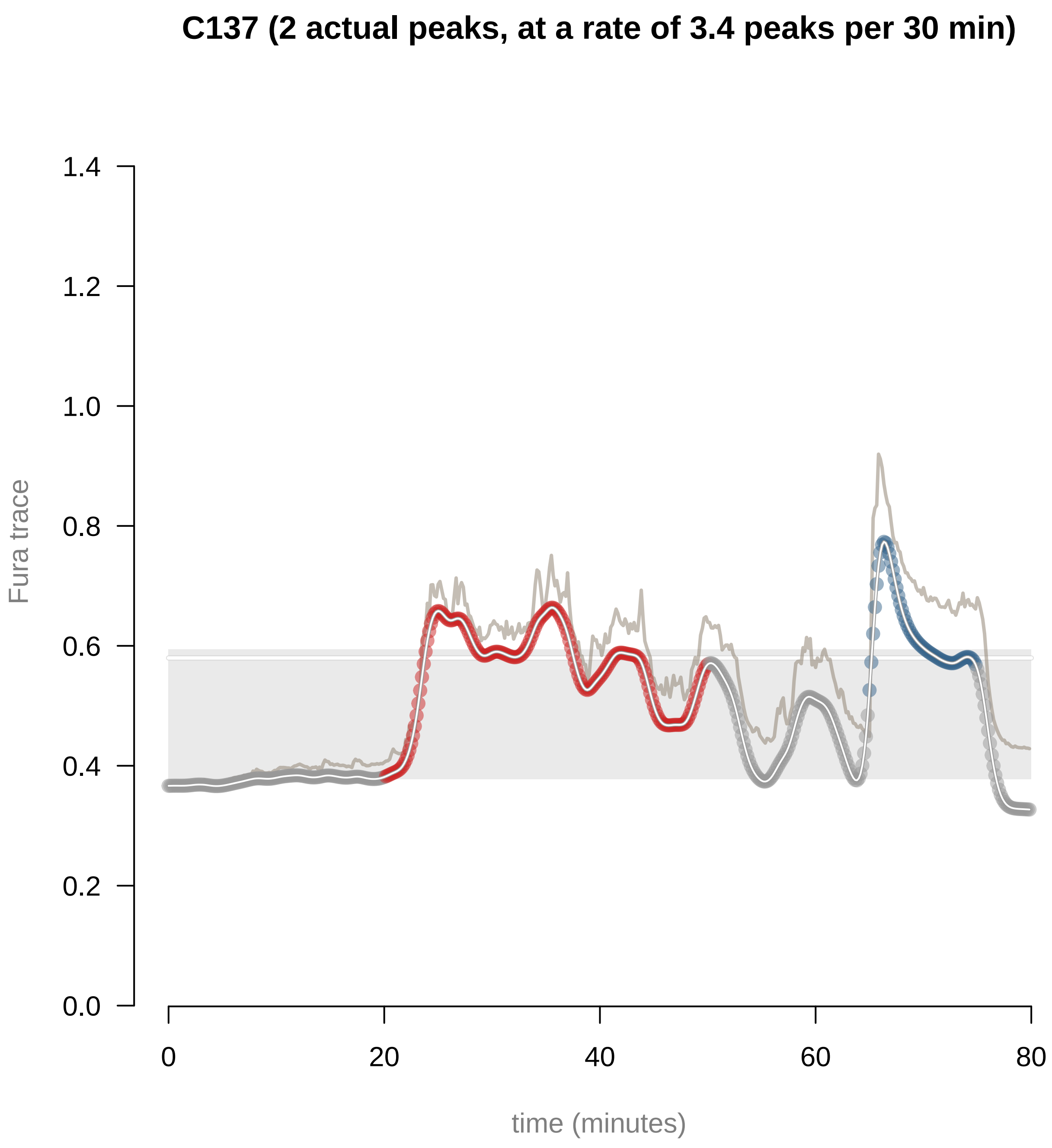
C137 (2 actual peaks, at a rate of 3.4 peaks per 30 min)
Fura trace
time (minutes)
0.0
0.2
0.4
0.6
0.8
1.0
1.2
1.4
0
20
40
60
80

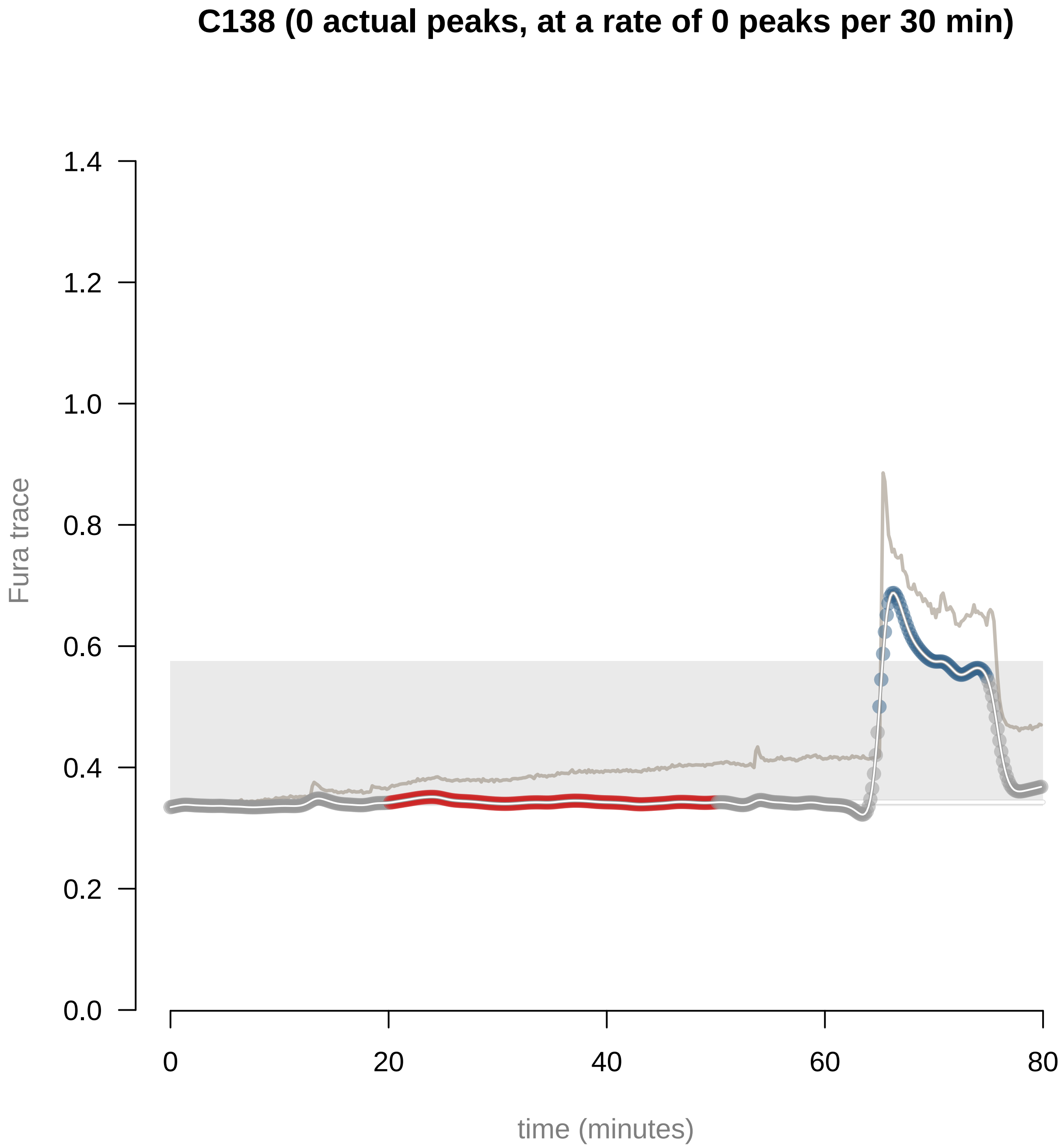
C138 (0 actual peaks, at a rate of 0 peaks per 30 min)
Fura trace
time (minutes)
0.0
0.2
0.4
0.6
0.8
1.0
1.2
1.4
0
20
40
60
80

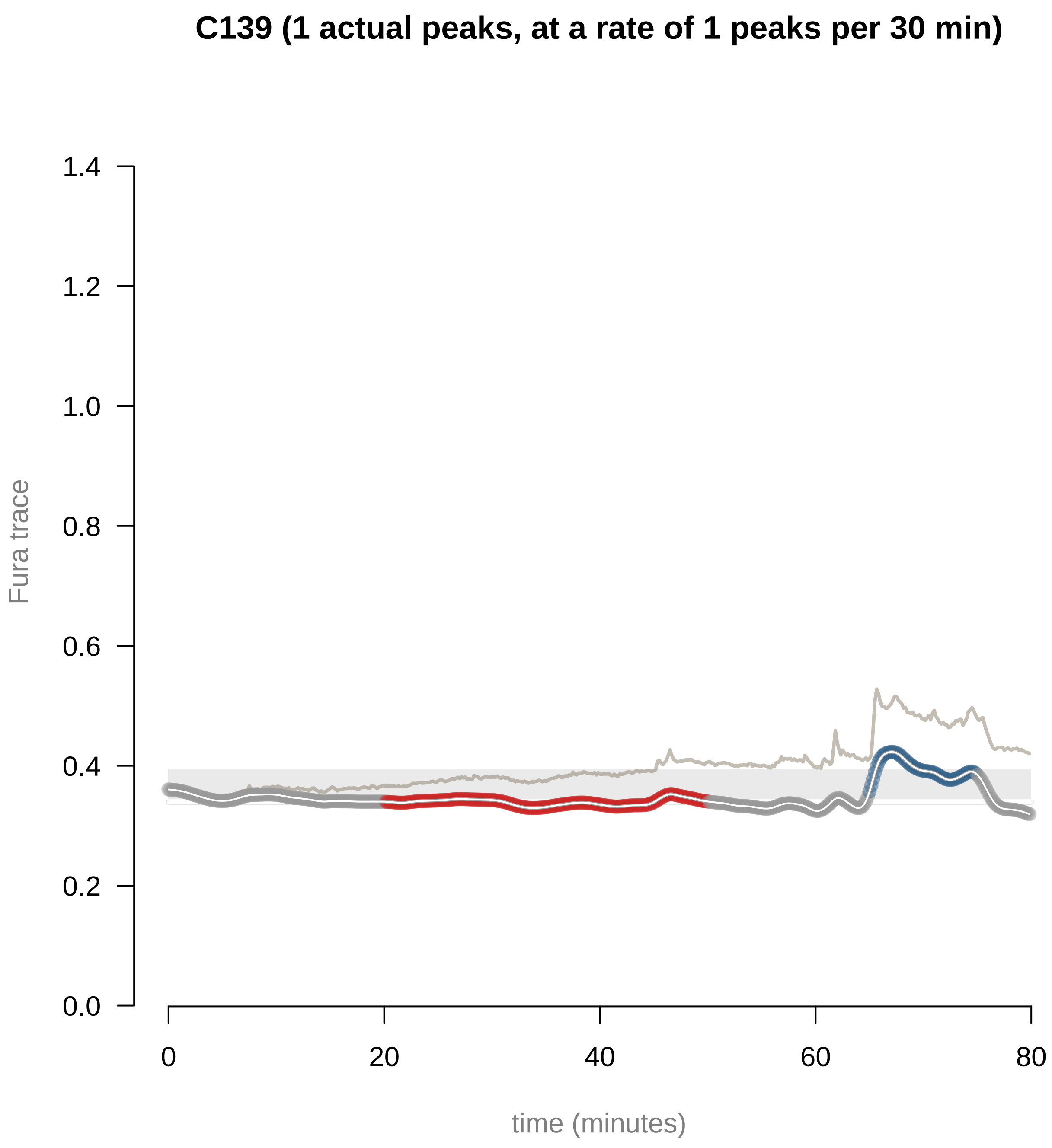
C139 (1 actual peaks, at a rate of 1 peaks per 30 min)
Fura trace
0.0
0.2
0.4
0.6
0.8
1.0
1.2
1.4
0
20
40
60
80
time (minutes)

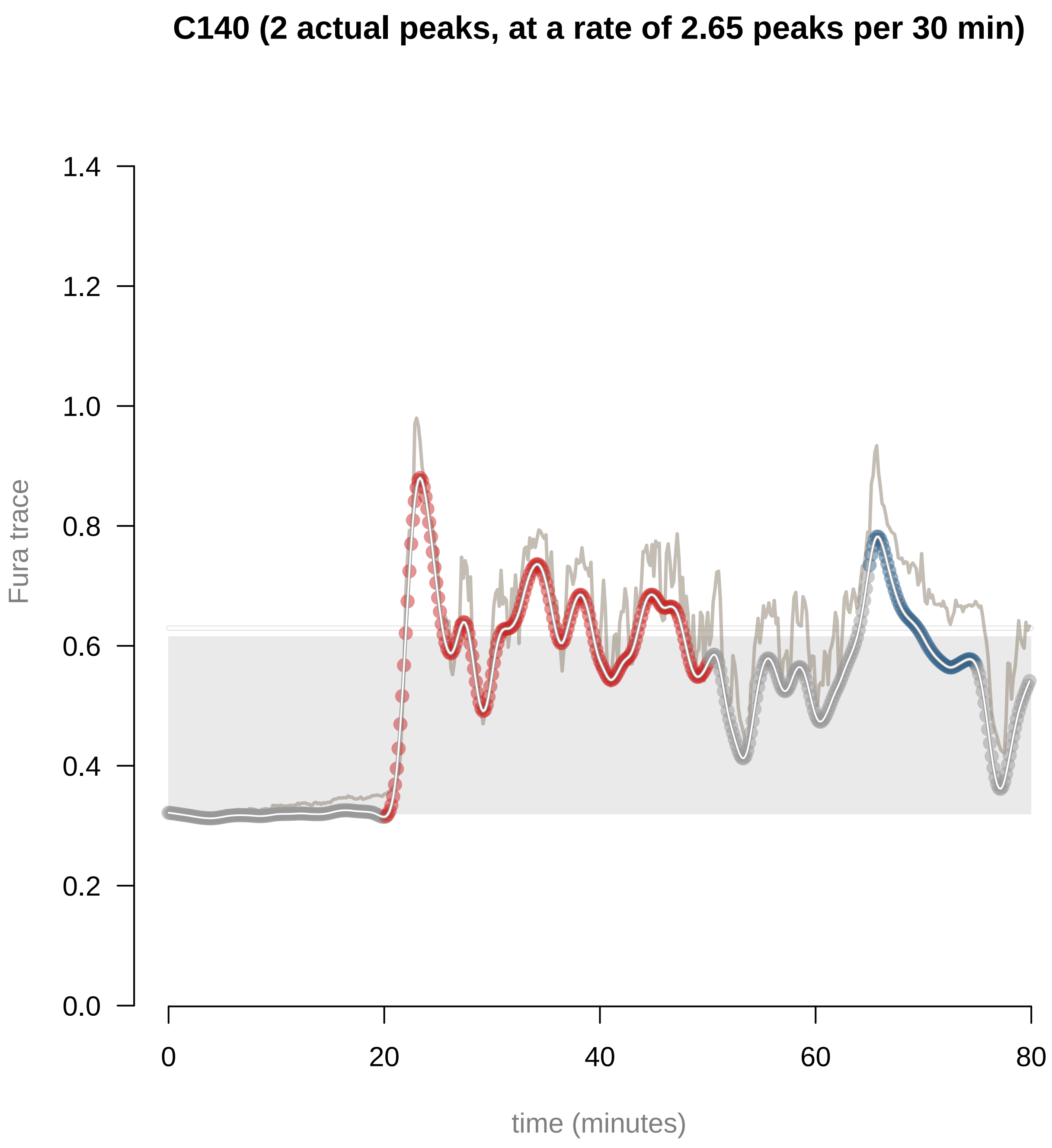
C140 (2 actual peaks, at a rate of 2.65 peaks per 30 min)
Fura trace
1.4
1.2
1.0
0.8
0.6
0.4
0.2
0.0
0
20
40
60
80
time (minutes)

C141 (3 actual peaks, at a rate of 2.61 peaks per 30 min)

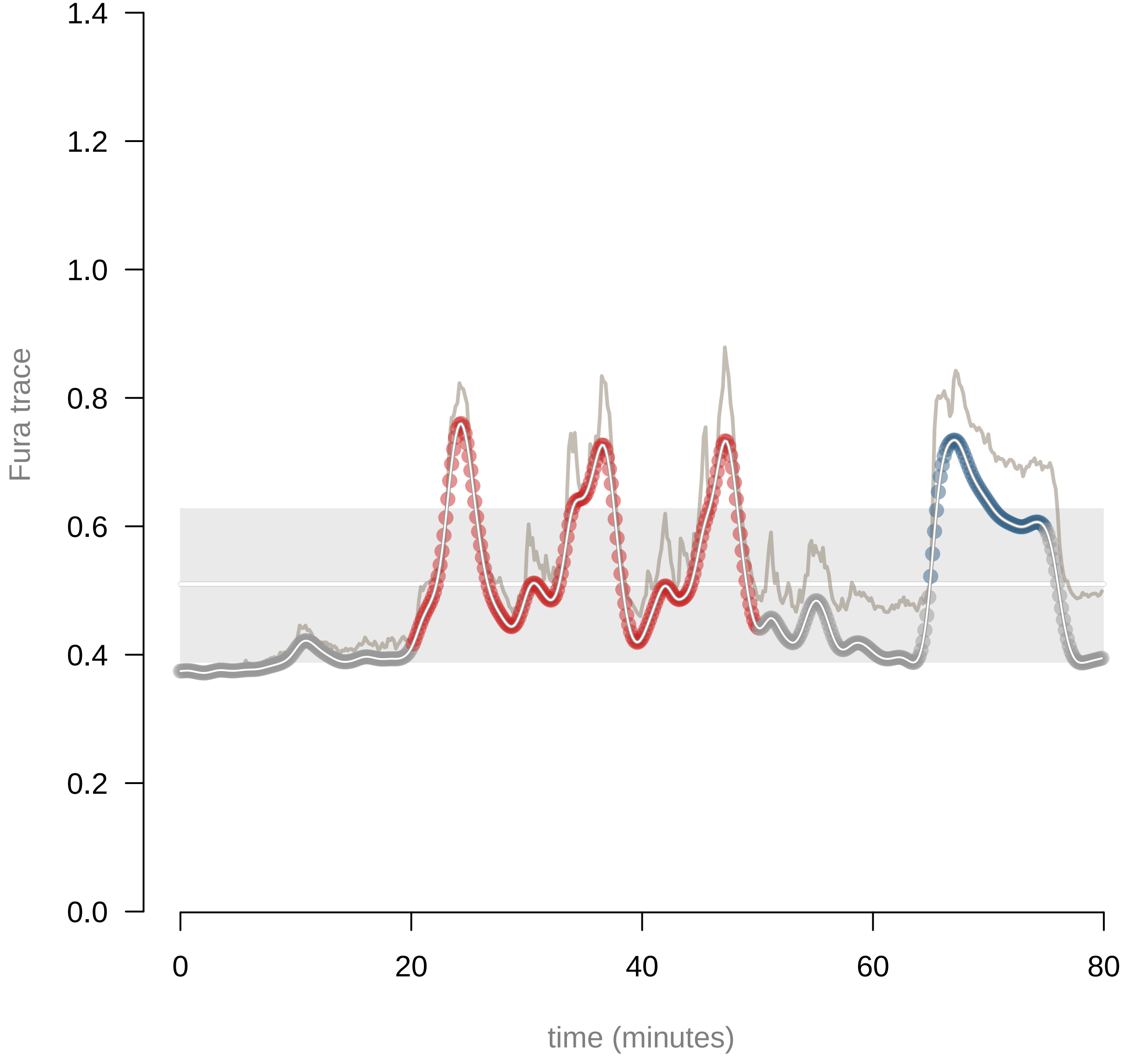

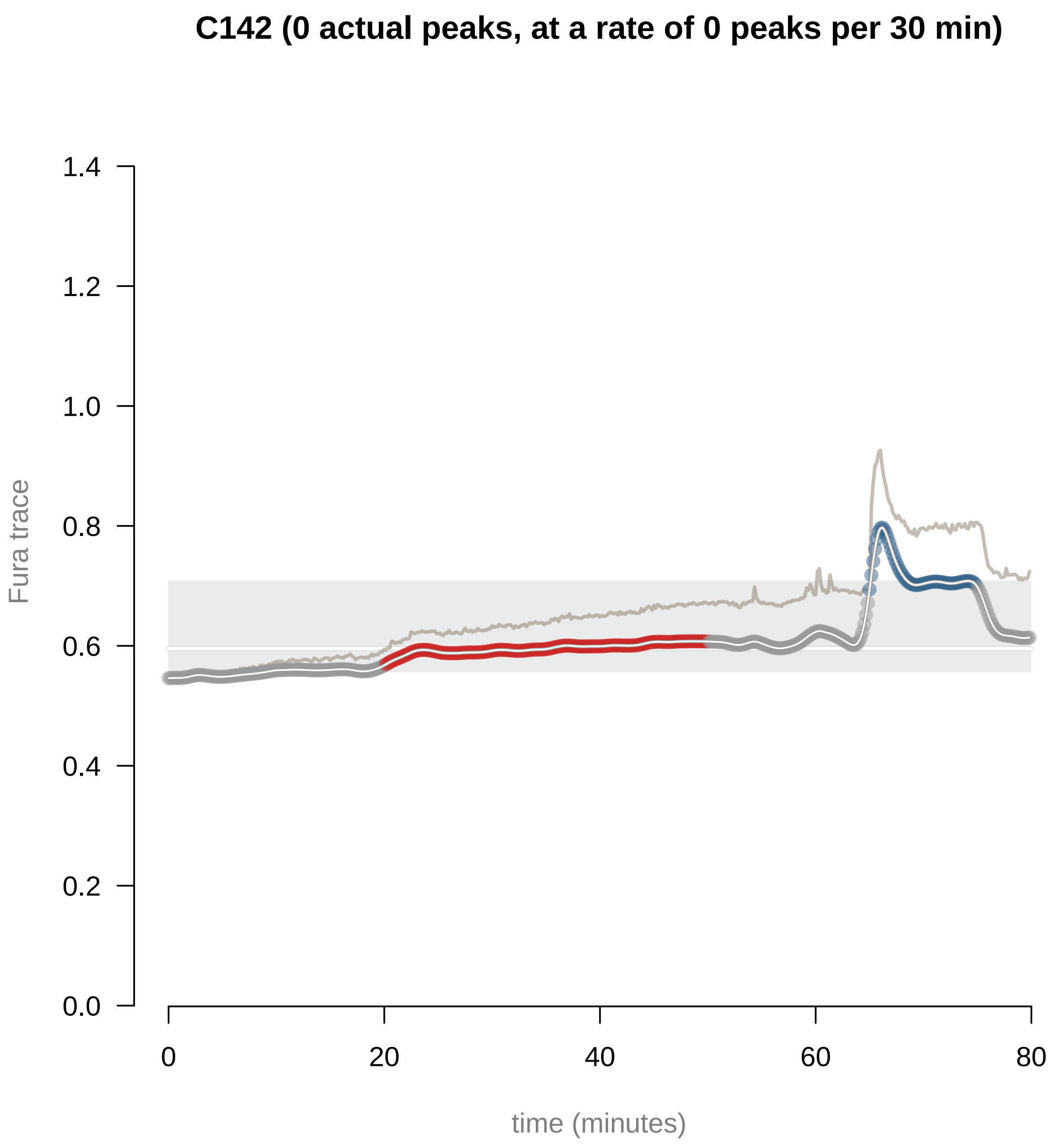
C142 (0 actual peaks, at a rate of 0 peaks per 30 min)
Fura trace
1.4
1.2
1.0
0.8
0.6
0.4
0.2
0.0
0
20
40
60
80
time (minutes)

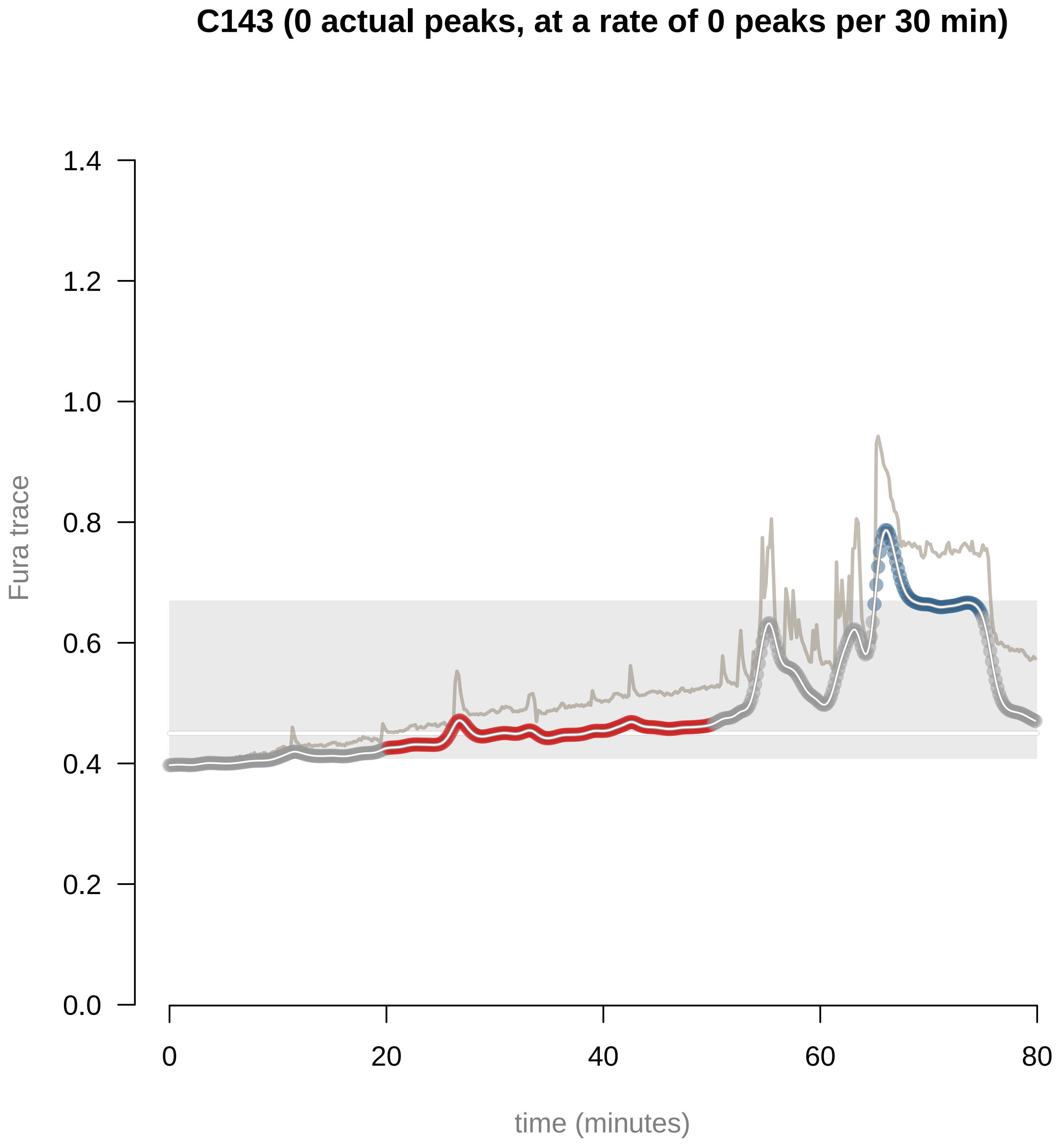
C143 (0 actual peaks, at a rate of 0 peaks per 30 min)
Fura trace
0.0
0.2
0.4
0.6
0.8
1.0
1.2
1.4
0
20
40
60
80
time (minutes)

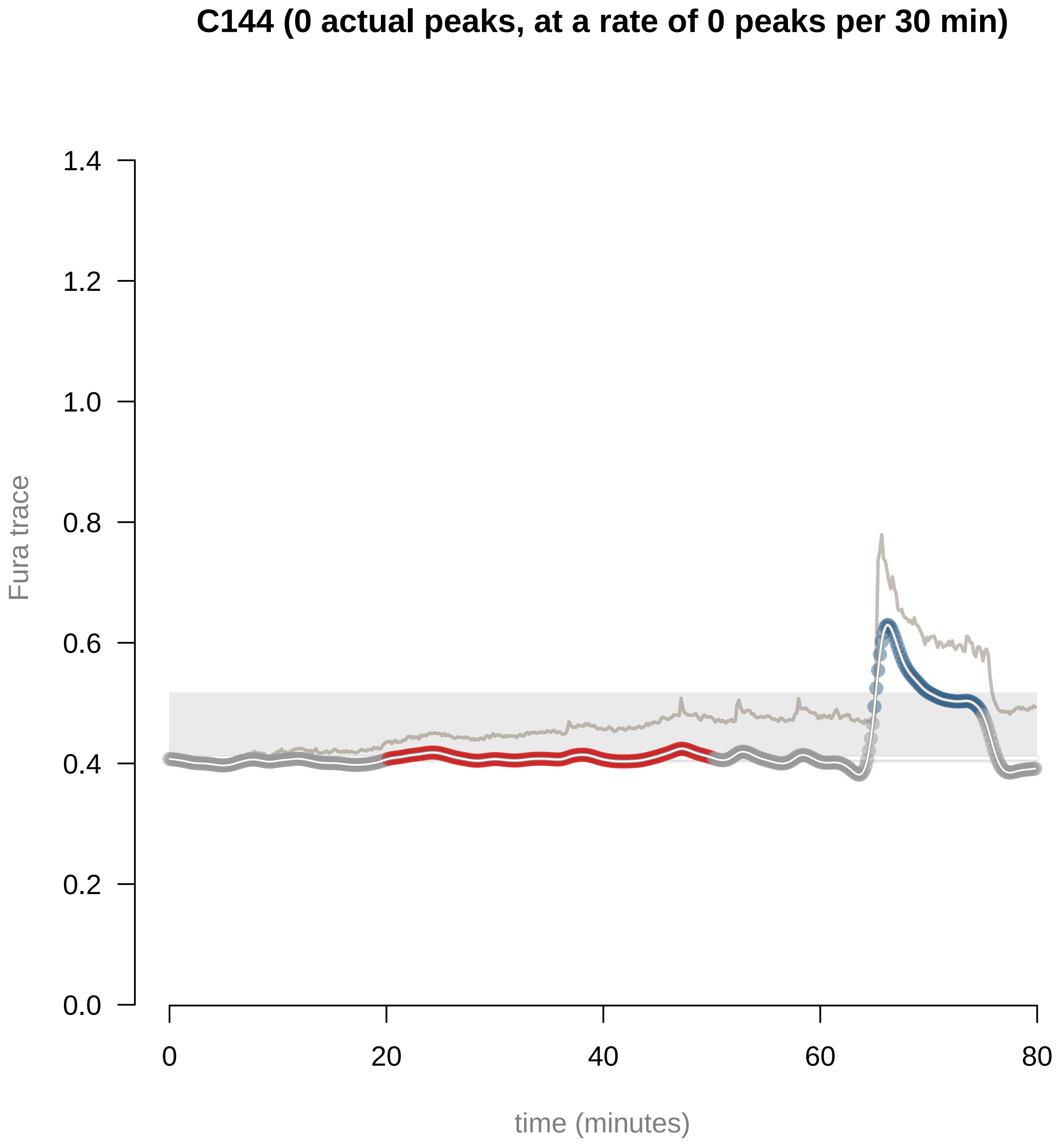
C144 (0 actual peaks, at a rate of 0 peaks per 30 min)
Fura trace
1.4
1.2
1.0
0.8
0.6
0.4
0.2
0.0
0
20
40
60
80
time (minutes)

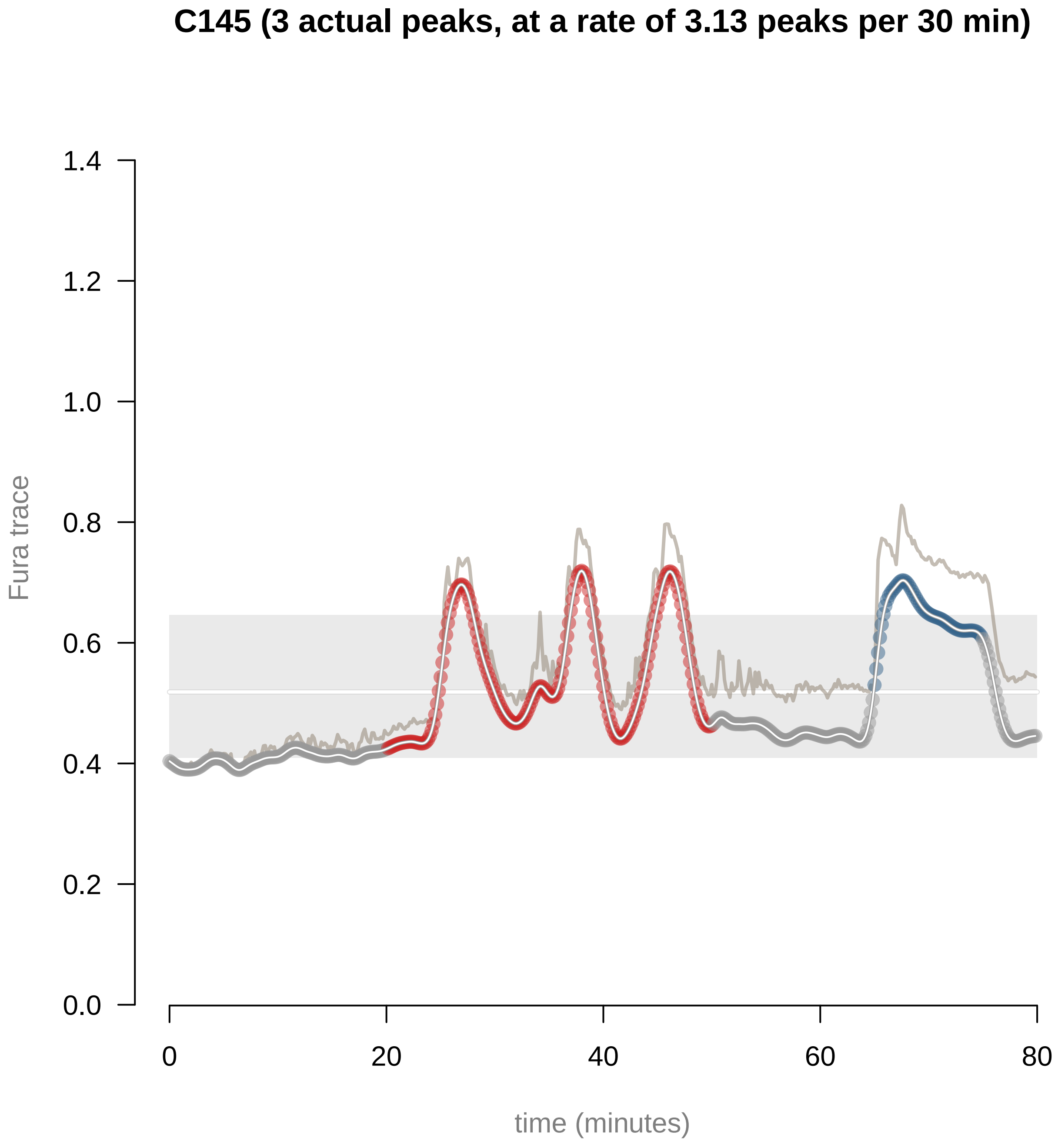

C145 (3 actual peaks, at a rate of 3.13 peaks per 30 min)
Fura trace
1.4
1.2
1.0
0.8
0.6
0.4
0.2
0.0
0
20
40
60
80
time (minutes)

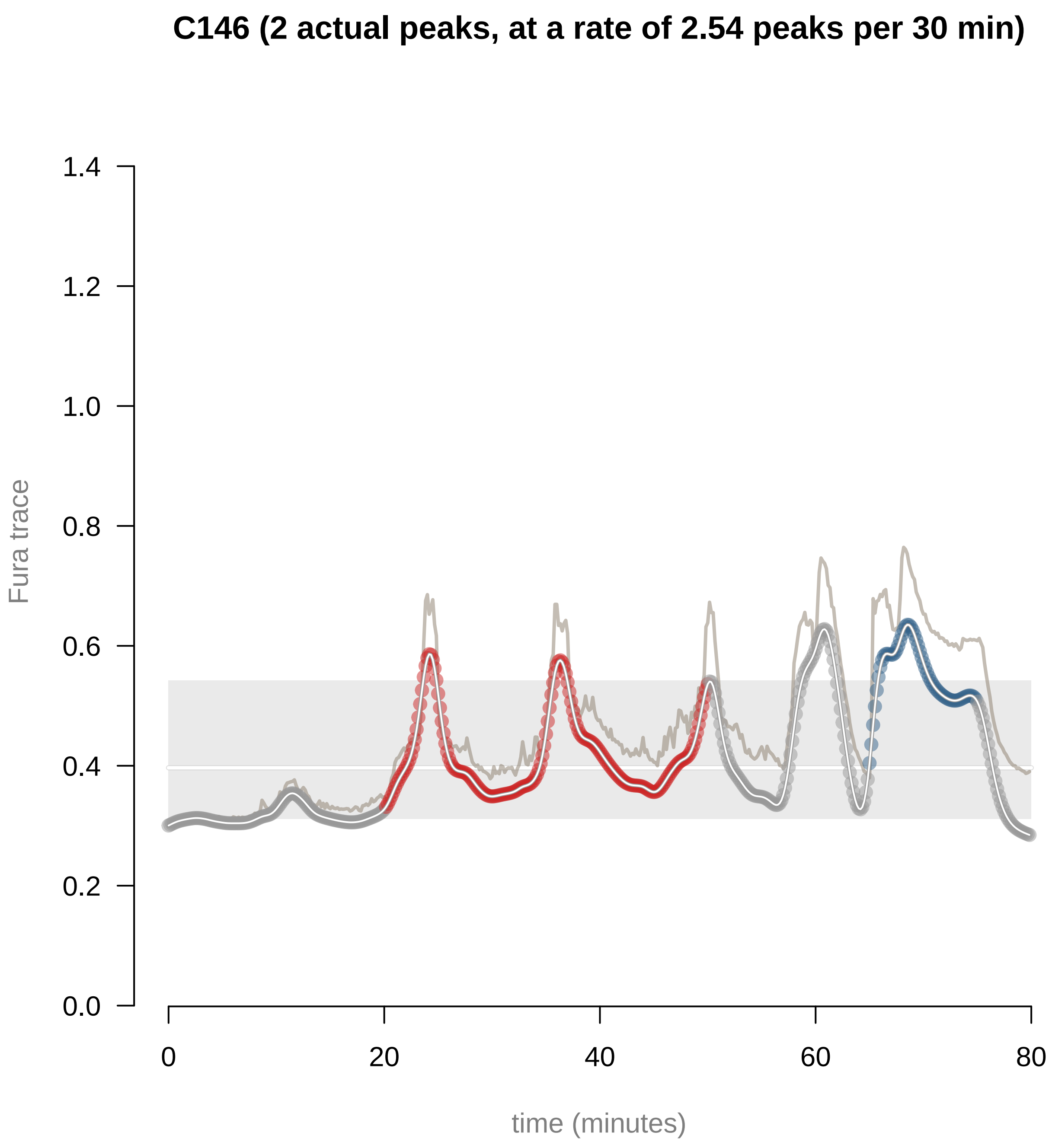
C146 (2 actual peaks, at a rate of 2.54 peaks per 30 min)
Fura trace
time (minutes)
0.0
0.2
0.4
0.6
0.8
1.0
1.2
1.4
0
20
40
60
80

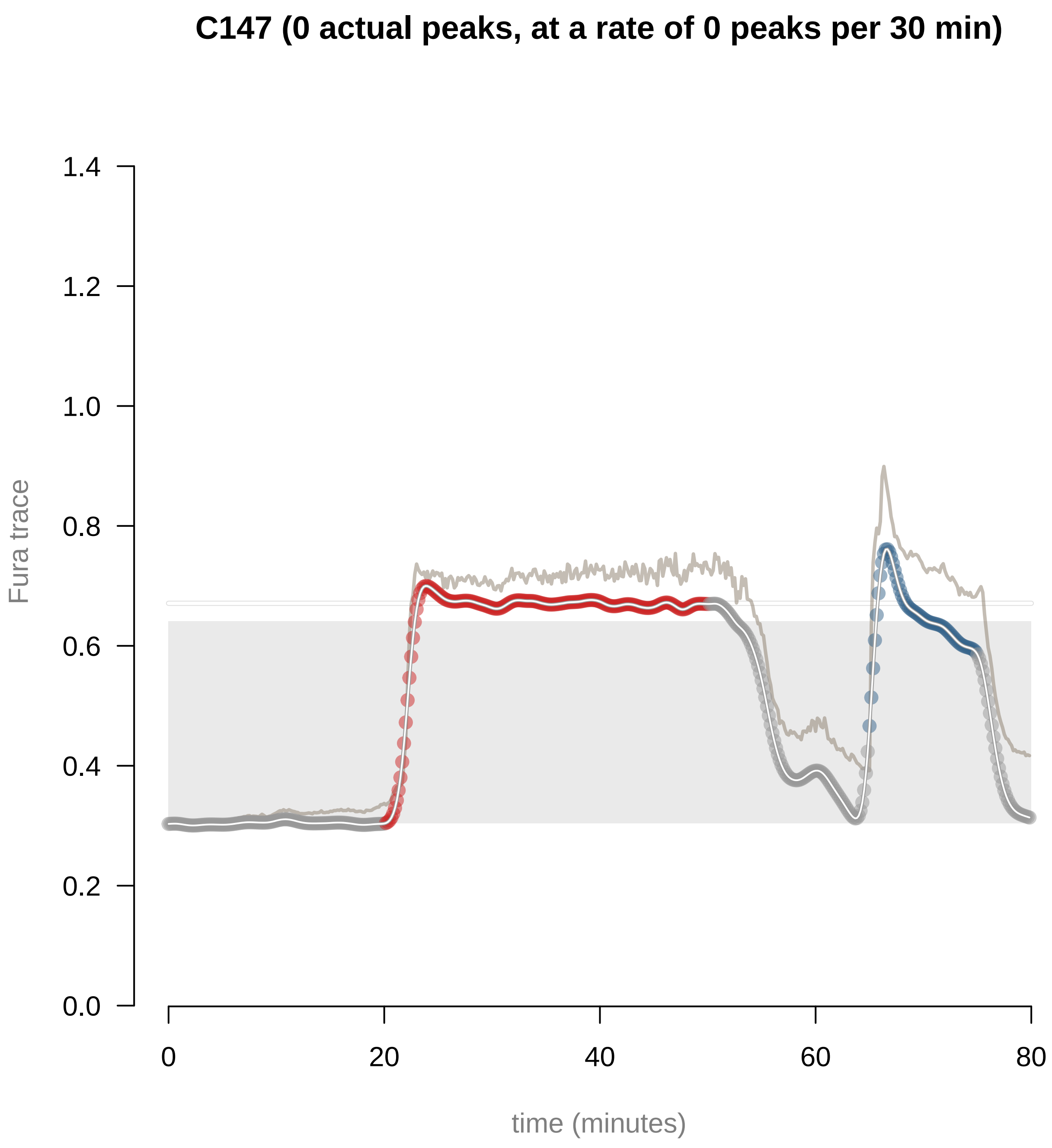
C147 (0 actual peaks, at a rate of 0 peaks per 30 min)
Fura trace
0.0
0.2
0.4
0.6
0.8
1.0
1.2
1.4
0
20
40
60
80
time (minutes)

C148 (0 actual peaks, at a rate of 0 peaks per 30 min)

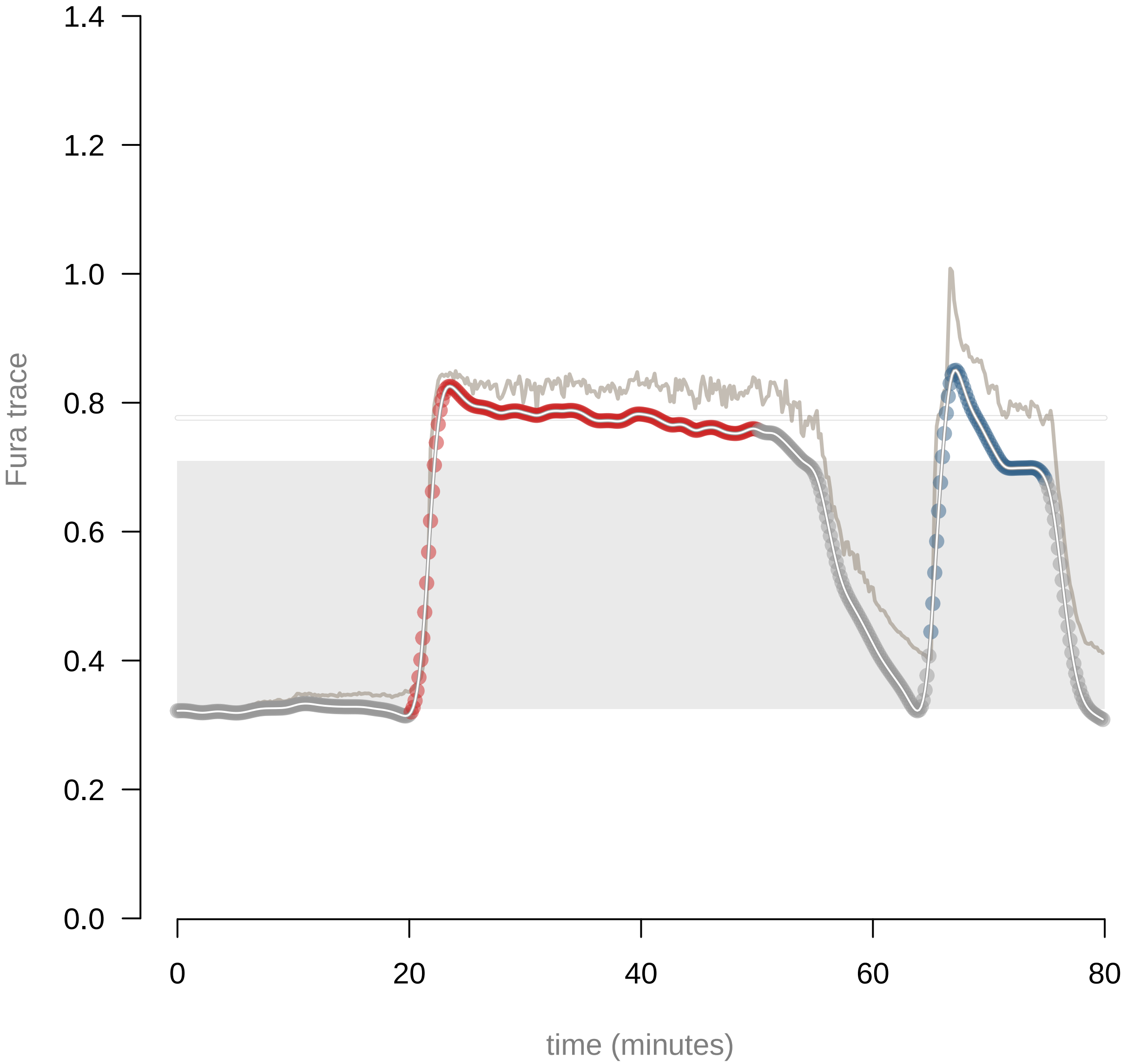

C149 (3 actual peaks, at a rate of 3.5 peaks per 30 min)

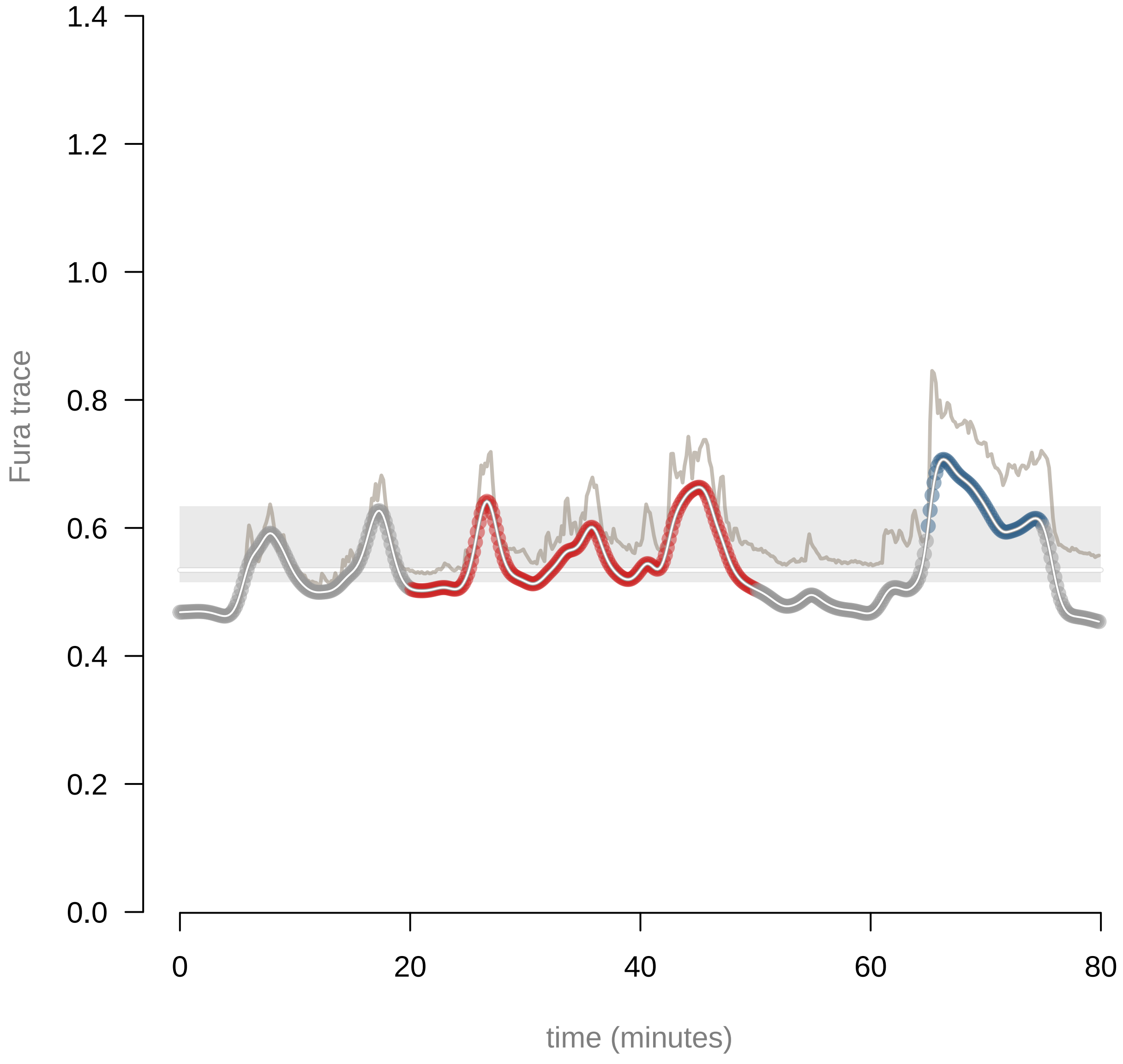

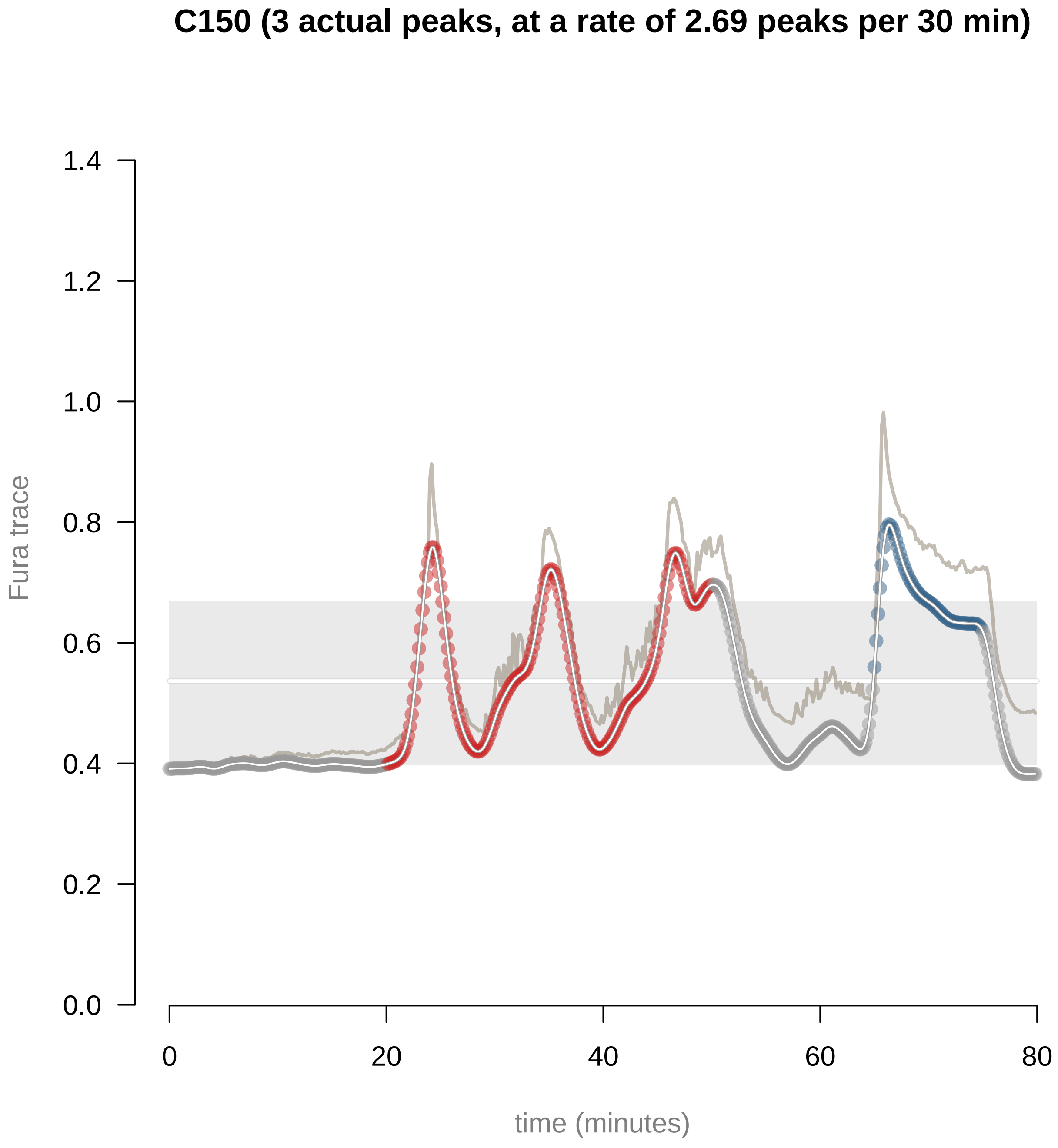
C150 (3 actual peaks, at a rate of 2.69 peaks per 30 min)
Fura trace
1.4
1.2
1.0
0.8
0.6
0.4
0.2
0.0
0
20
40
60
80
time (minutes)

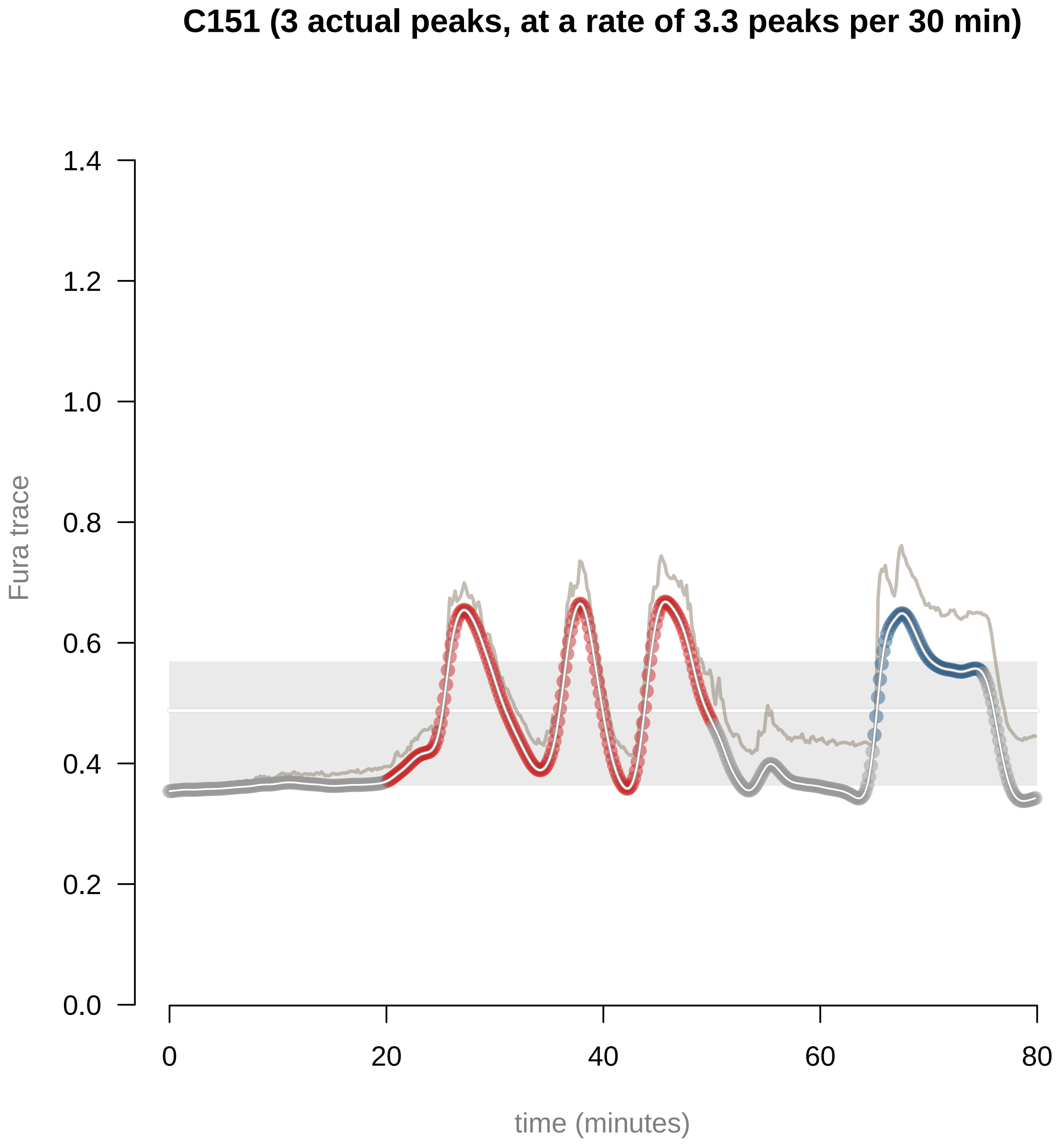

C151 (3 actual peaks, at a rate of 3.3 peaks per 30 min)
Fura trace
time (minutes)
0.0
0.2
0.4
0.6
0.8
1.0
1.2
1.4
0
20
40
60
80

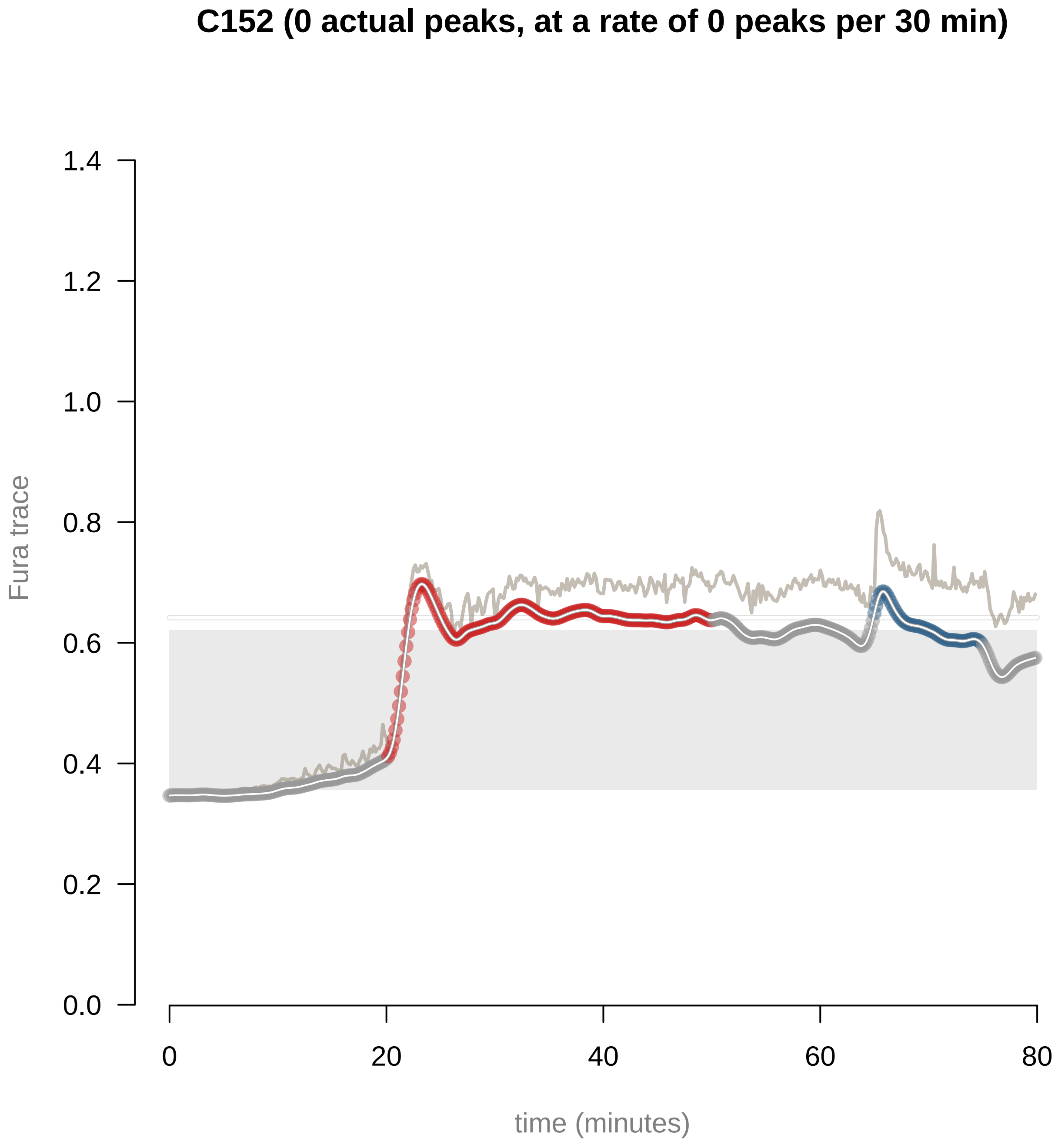
C152 (0 actual peaks, at a rate of 0 peaks per 30 min)
Fura trace
0.0
0.2
0.4
0.6
0.8
1.0
1.2
1.4
0
20
40
60
80
time (minutes)

C153 (1 actual peaks, at a rate of 1 peaks per 30 min)

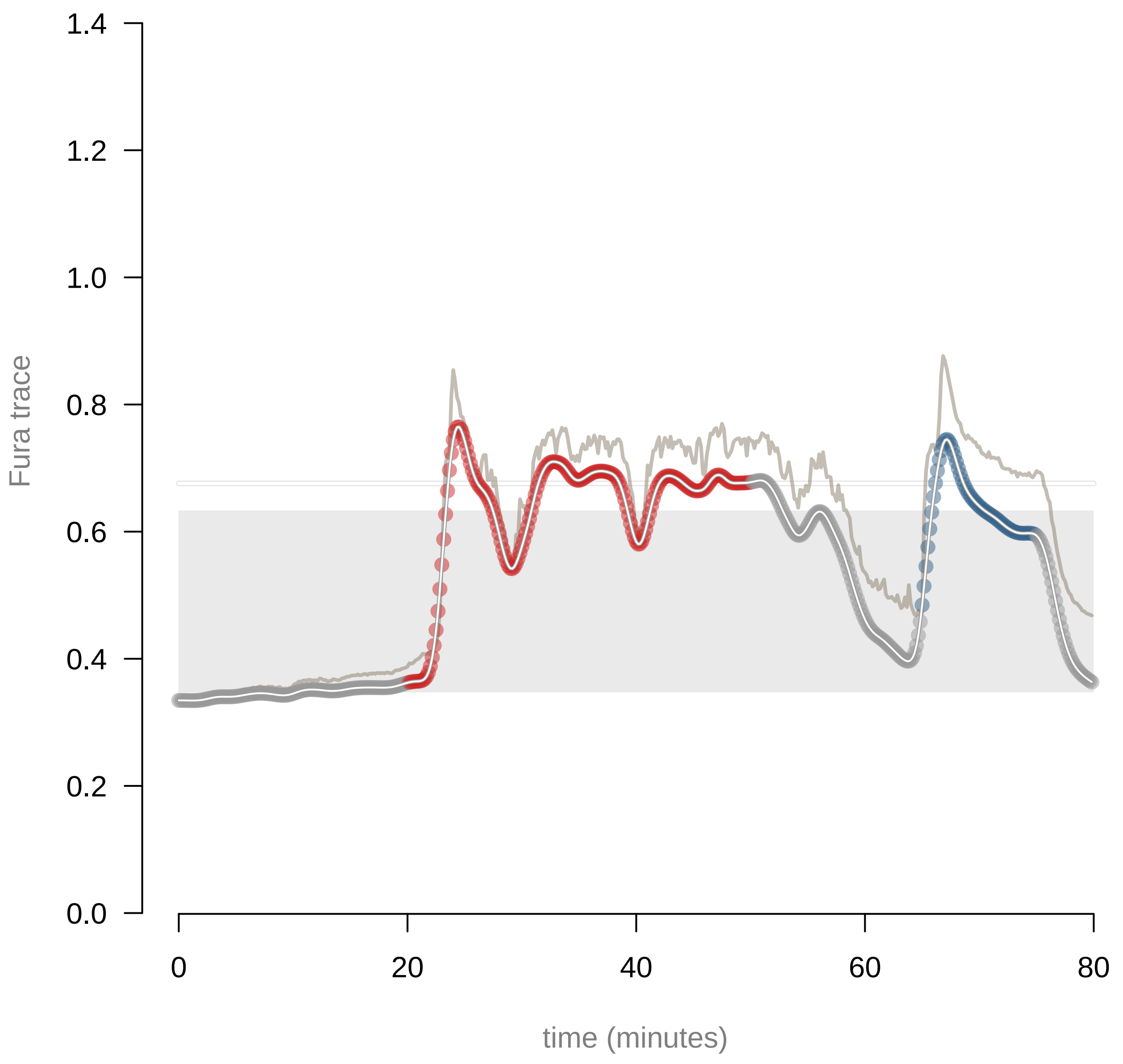

C154 (3 actual peaks, at a rate of 2.93 peaks per 30 min)

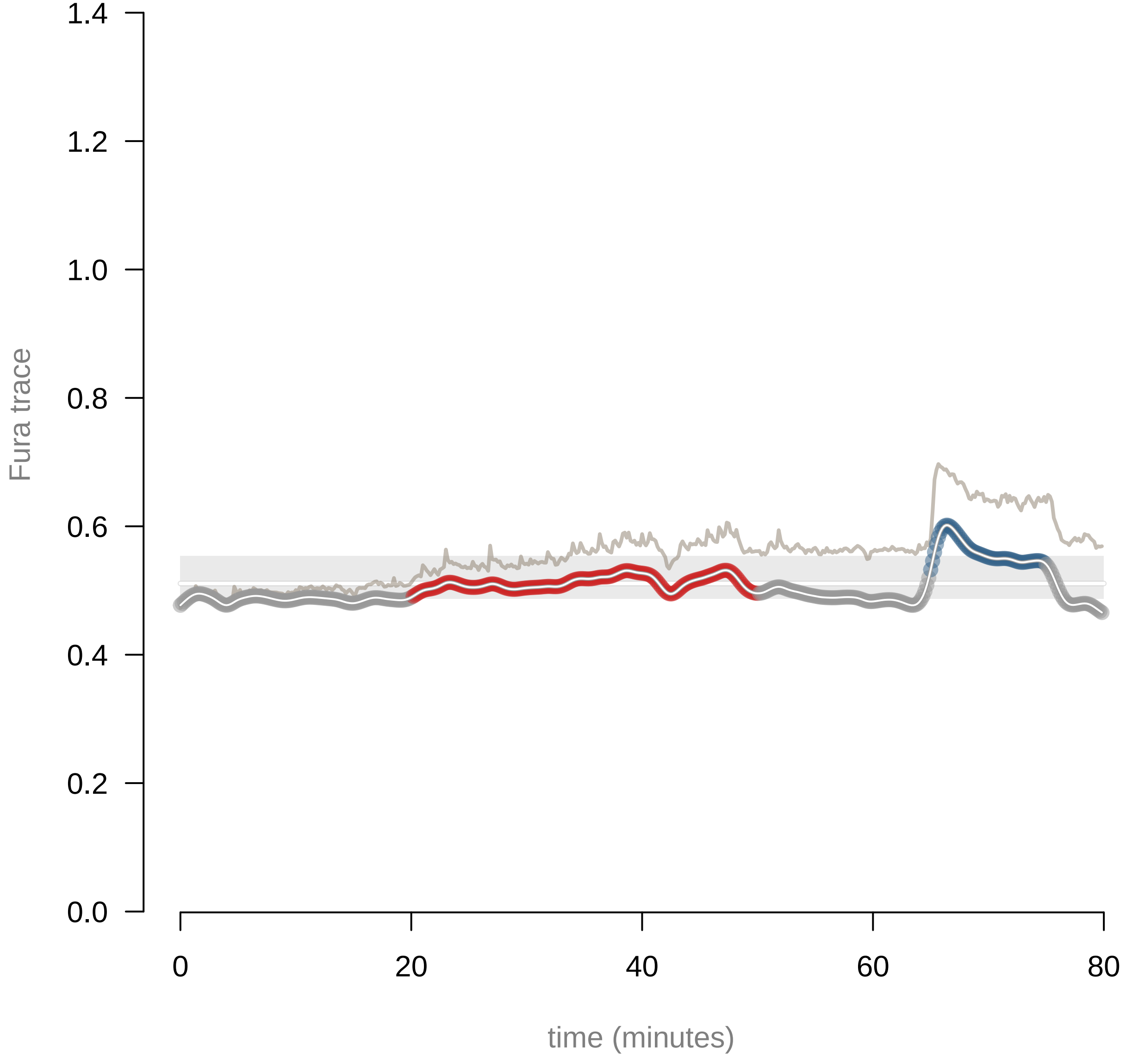

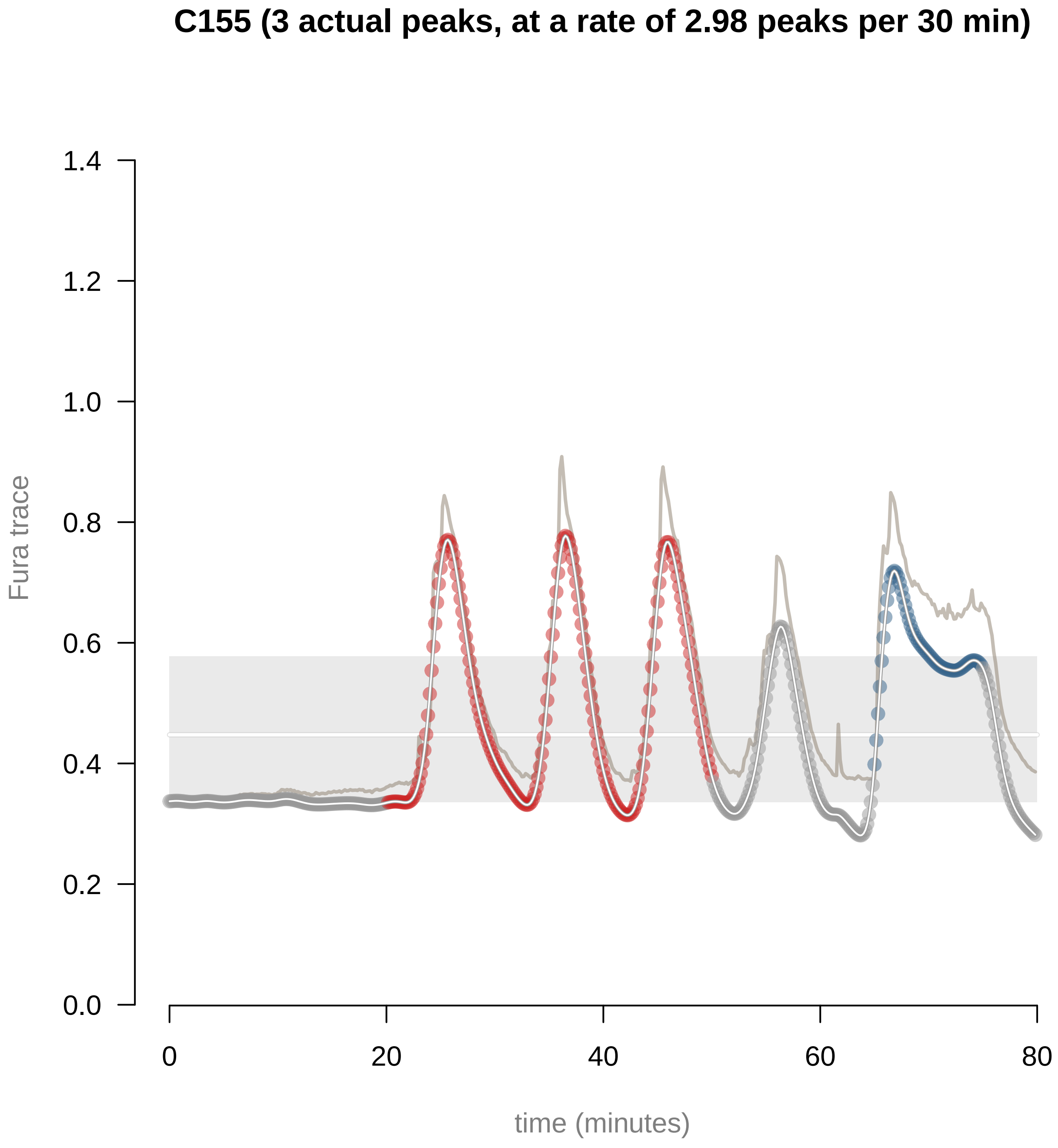

C155 (3 actual peaks, at a rate of 2.98 peaks per 30 min)
Fura trace
time (minutes)
0.0
0.2
0.4
0.6
0.8
1.0
1.2
1.4
0
20
40
60
80

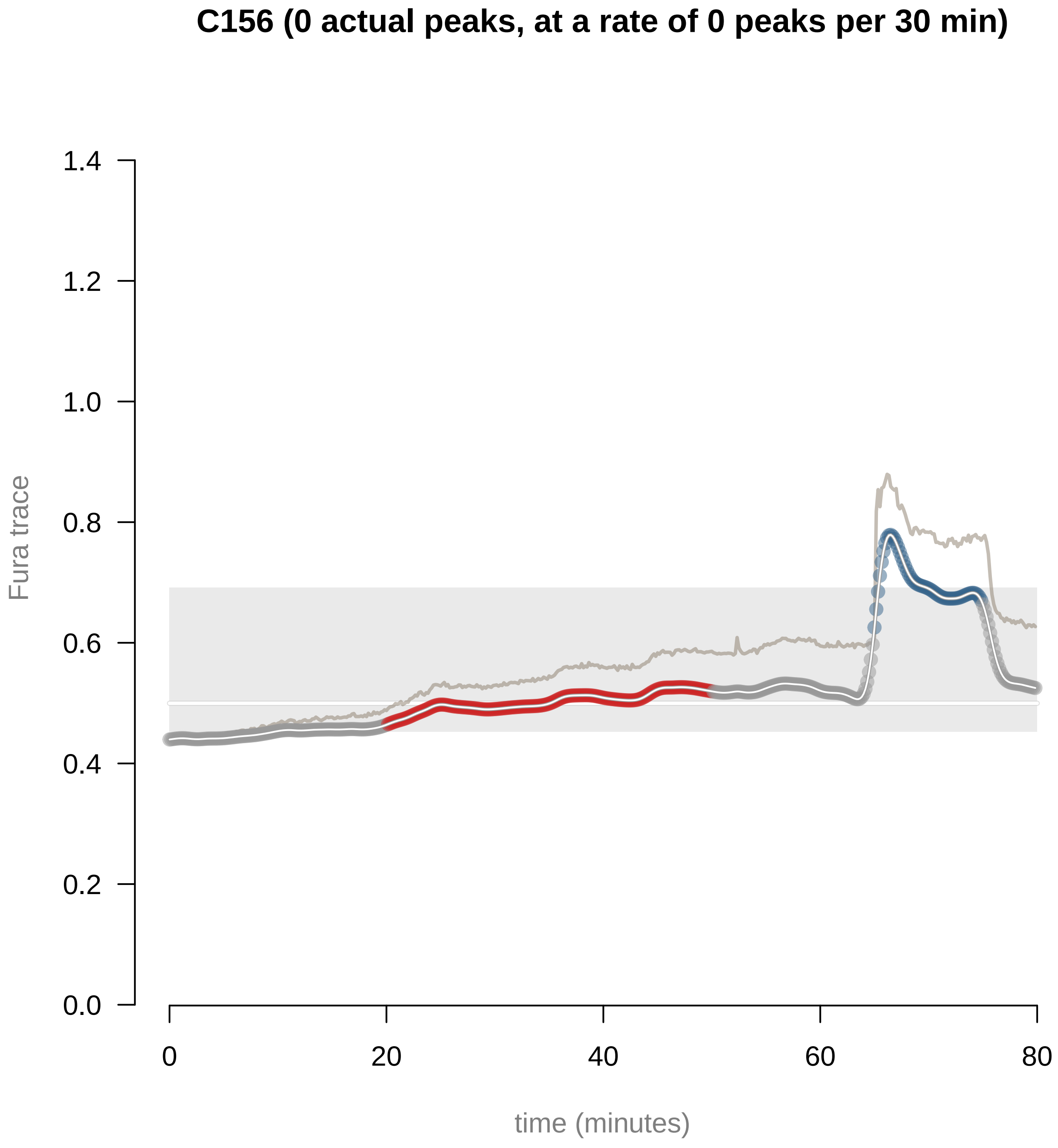

C156 (0 actual peaks, at a rate of 0 peaks per 30 min)
Fura trace
1.4
1.2
1.0
0.8
0.6
0.4
0.2
0.0
0
20
40
60
80
time (minutes)

C157 (3 actual peaks, at a rate of 4.04 peaks per 30 min)

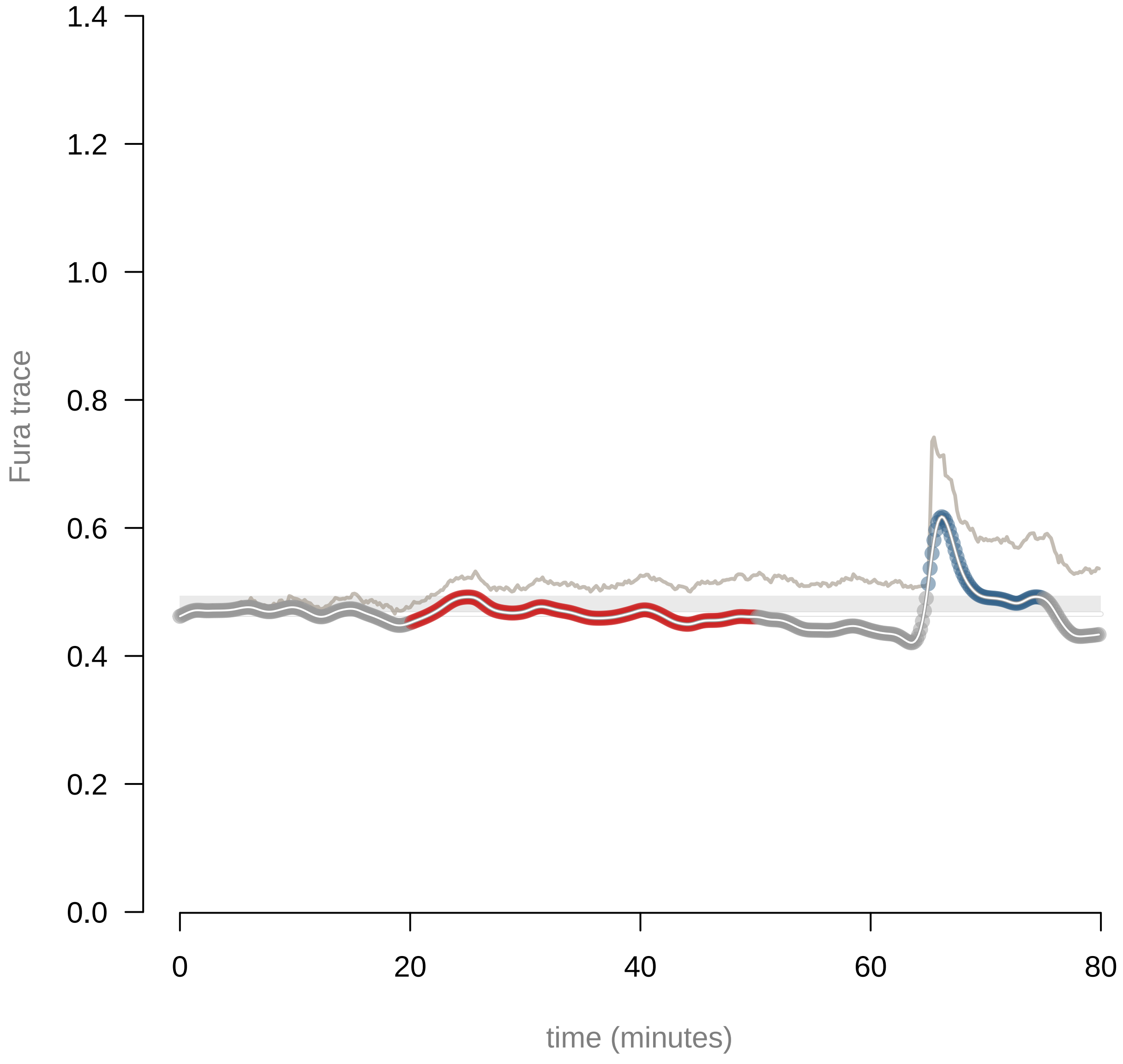

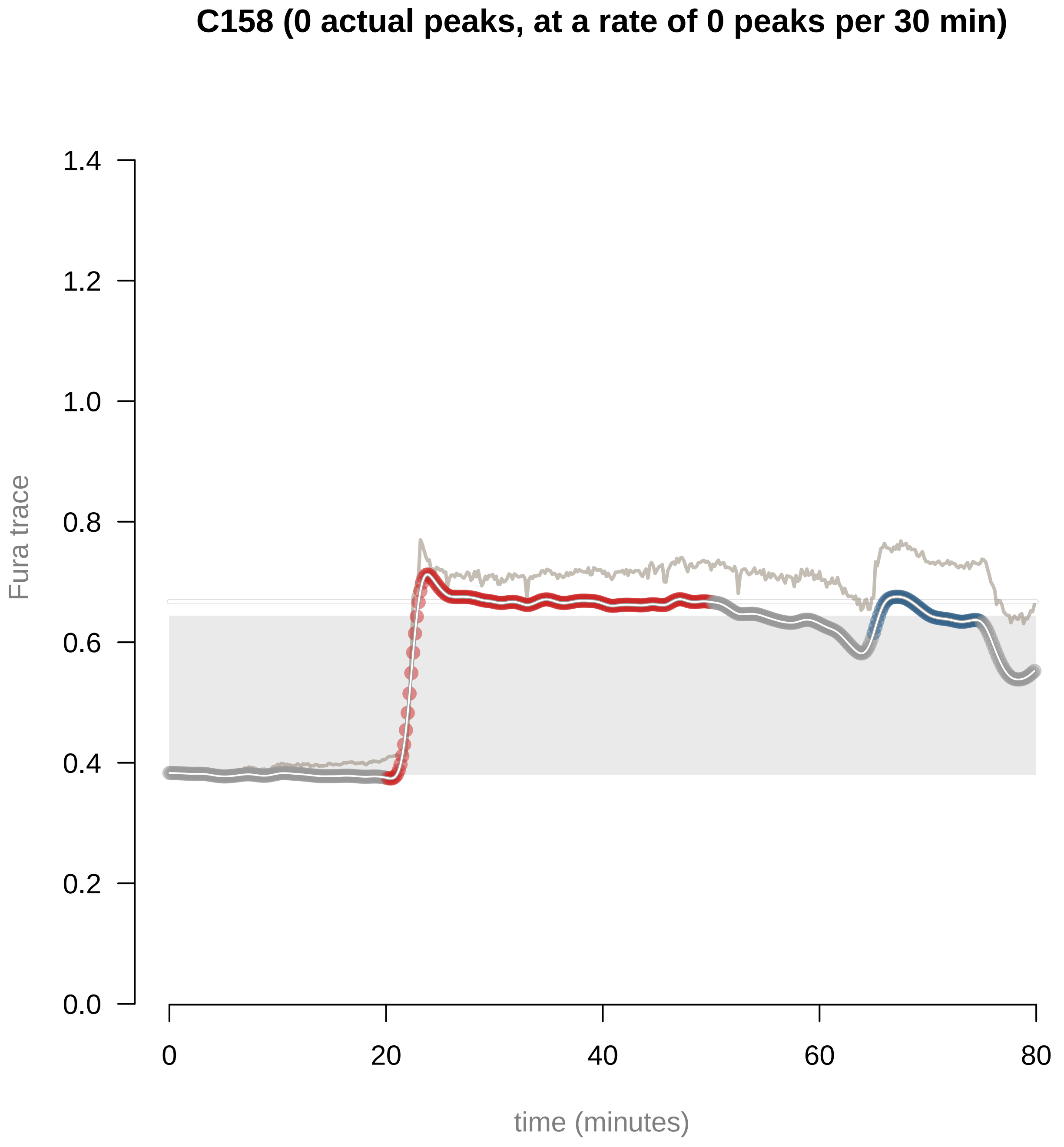

C158 (0 actual peaks, at a rate of 0 peaks per 30 min)
Fura trace
time (minutes)
0.0
0.2
0.4
0.6
0.8
1.0
1.2
1.4
0
20
40
60
80

C159 (3 actual peaks, at a rate of 3.4 peaks per 30 min)

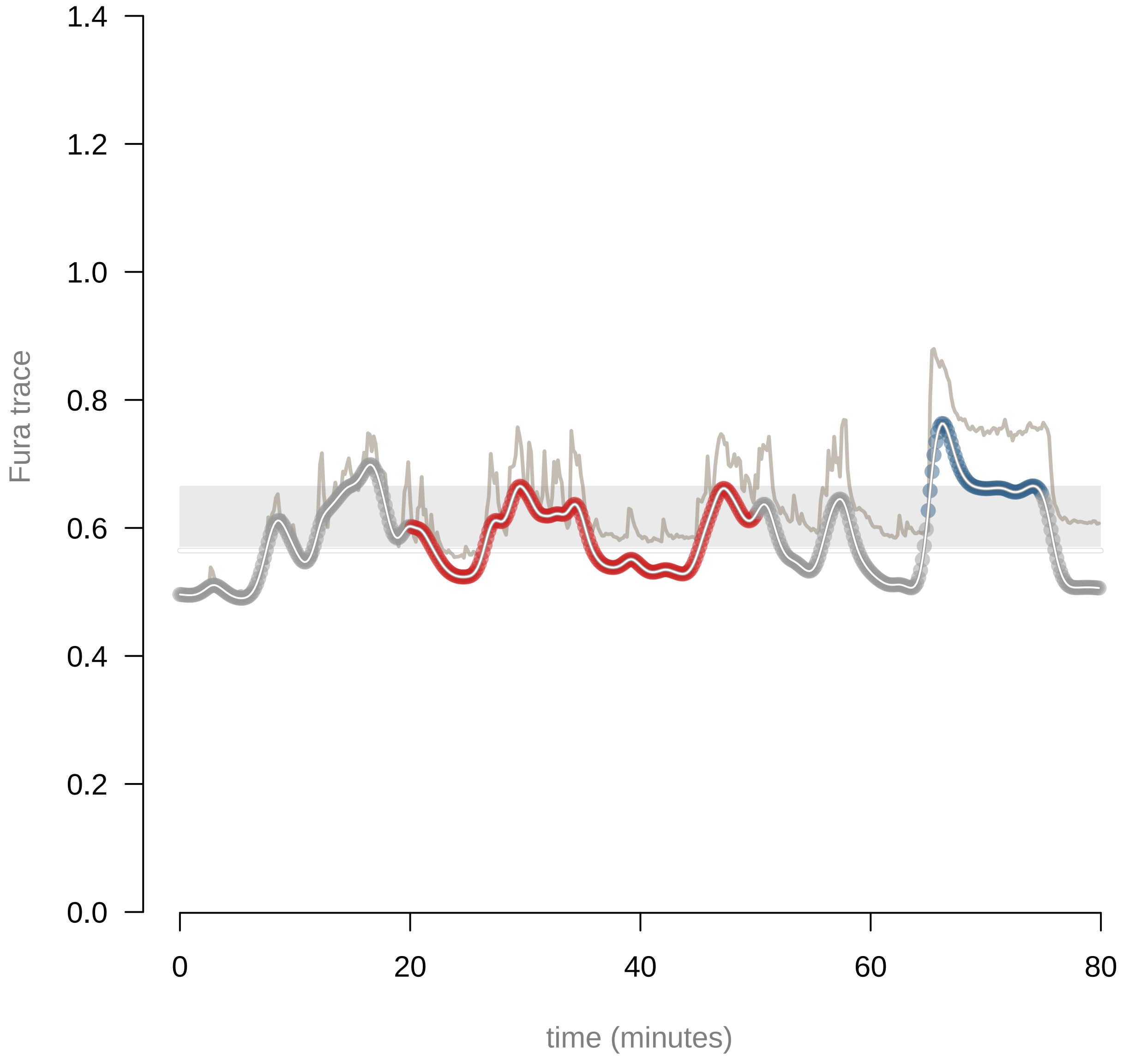

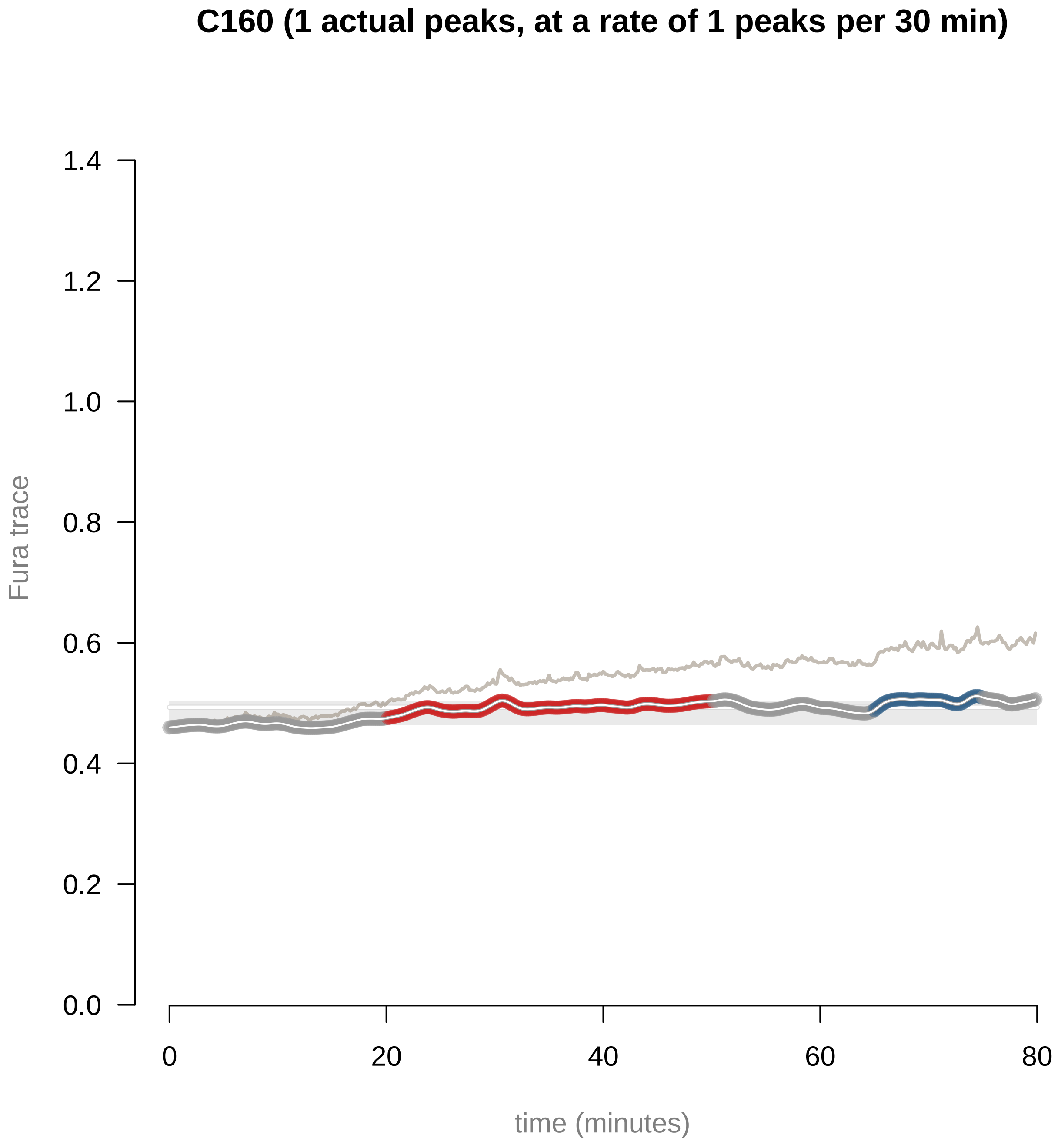
C160 (1 actual peaks, at a rate of 1 peaks per 30 min)
Fura trace
1.4
1.2
1.0
0.8
0.6
0.4
0.2
0.0
0
20
40
60
80
time (minutes)

C161 (3 actual peaks, at a rate of 4.86 peaks per 30 min)

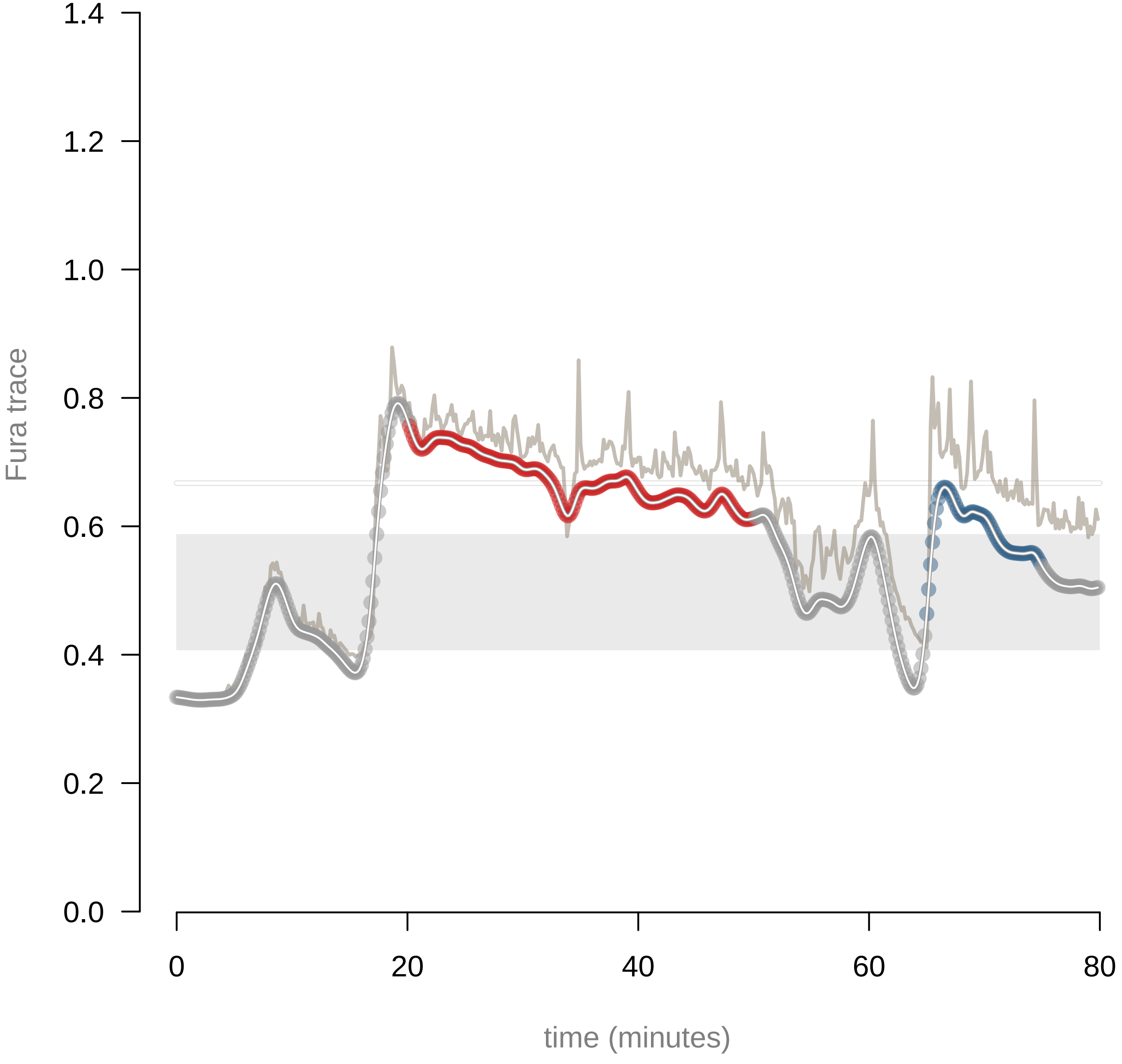

C162 (1 actual peaks, at a rate of 1 peaks per 30 min)

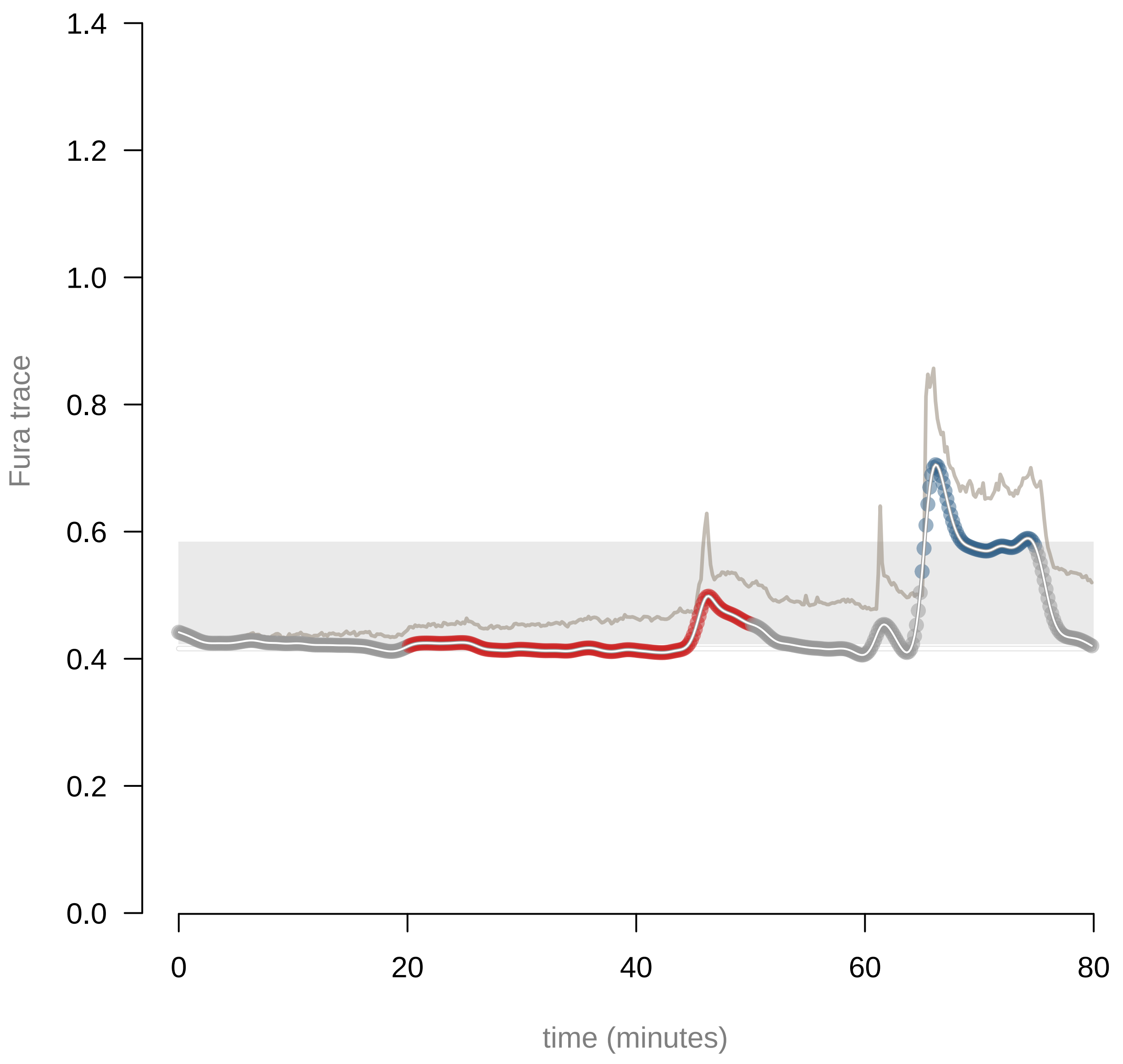

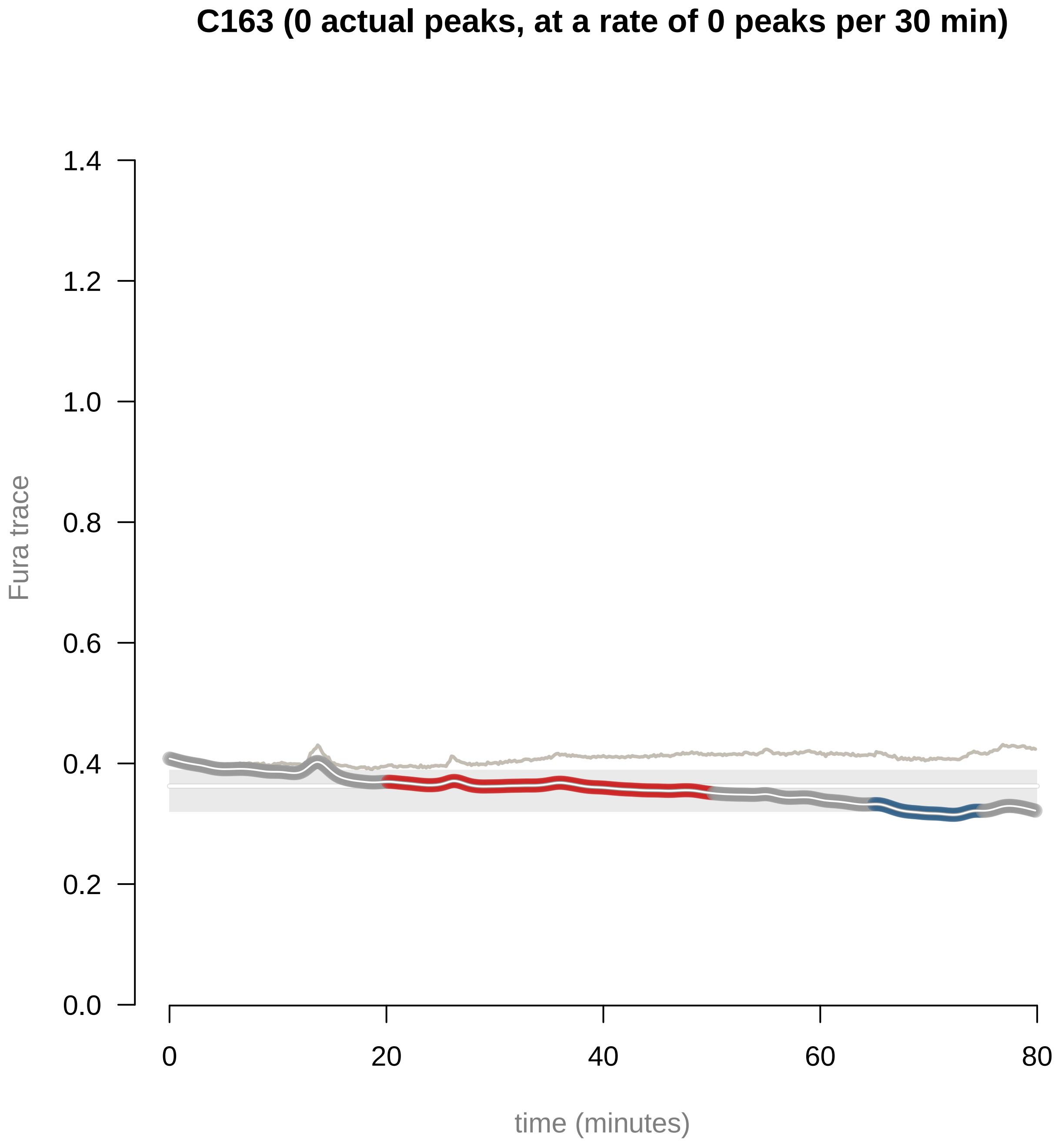

C163 (0 actual peaks, at a rate of 0 peaks per 30 min)
Fura trace
1.4
1.2
1.0
0.8
0.6
0.4
0.2
0.0
0
20
40
60
80
time (minutes)

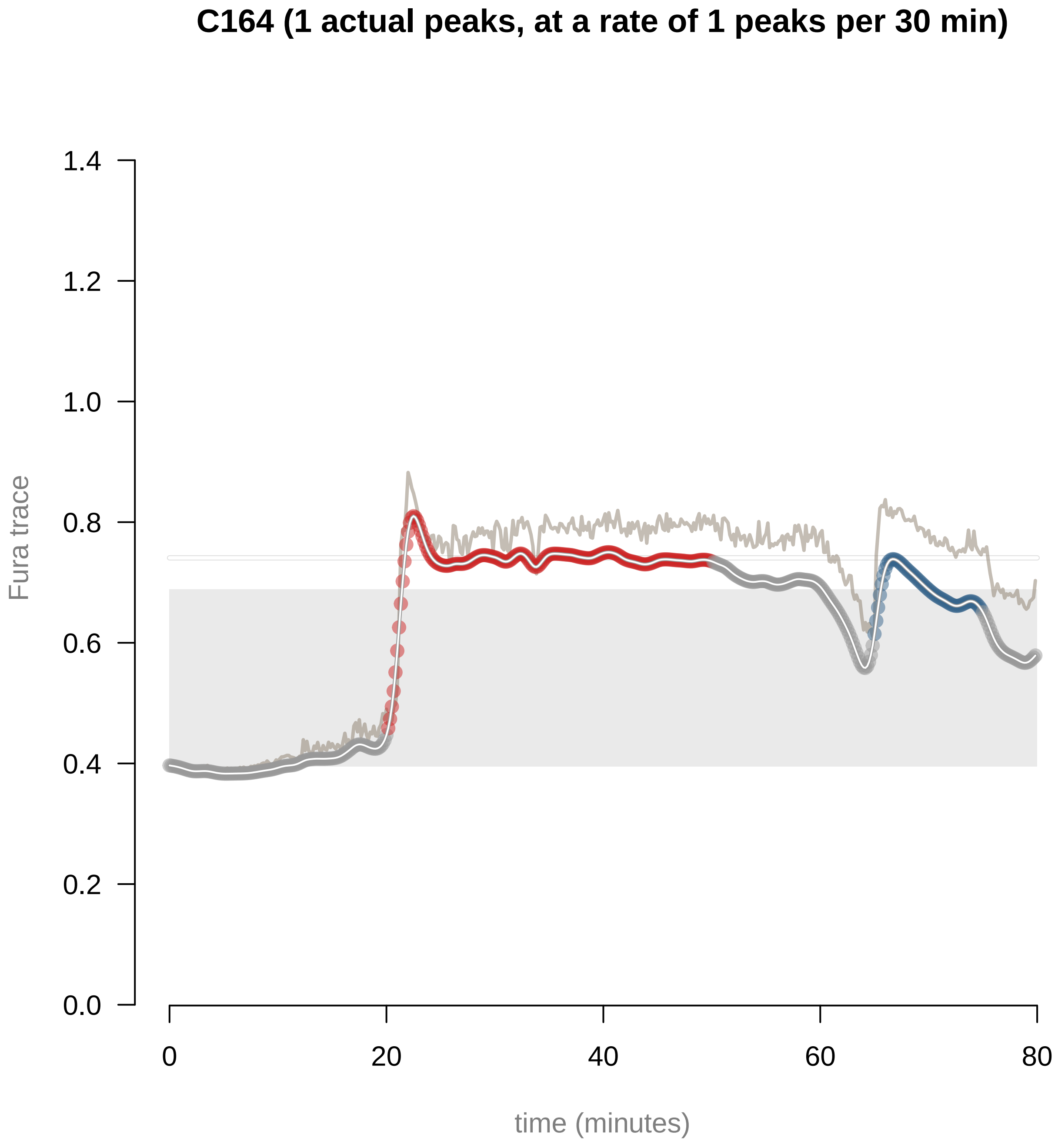
C164 (1 actual peaks, at a rate of 1 peaks per 30 min)
Fura trace
1.4
1.2
1.0
0.8
0.6
0.4
0.2
0.0
0
20
40
60
80
time (minutes)

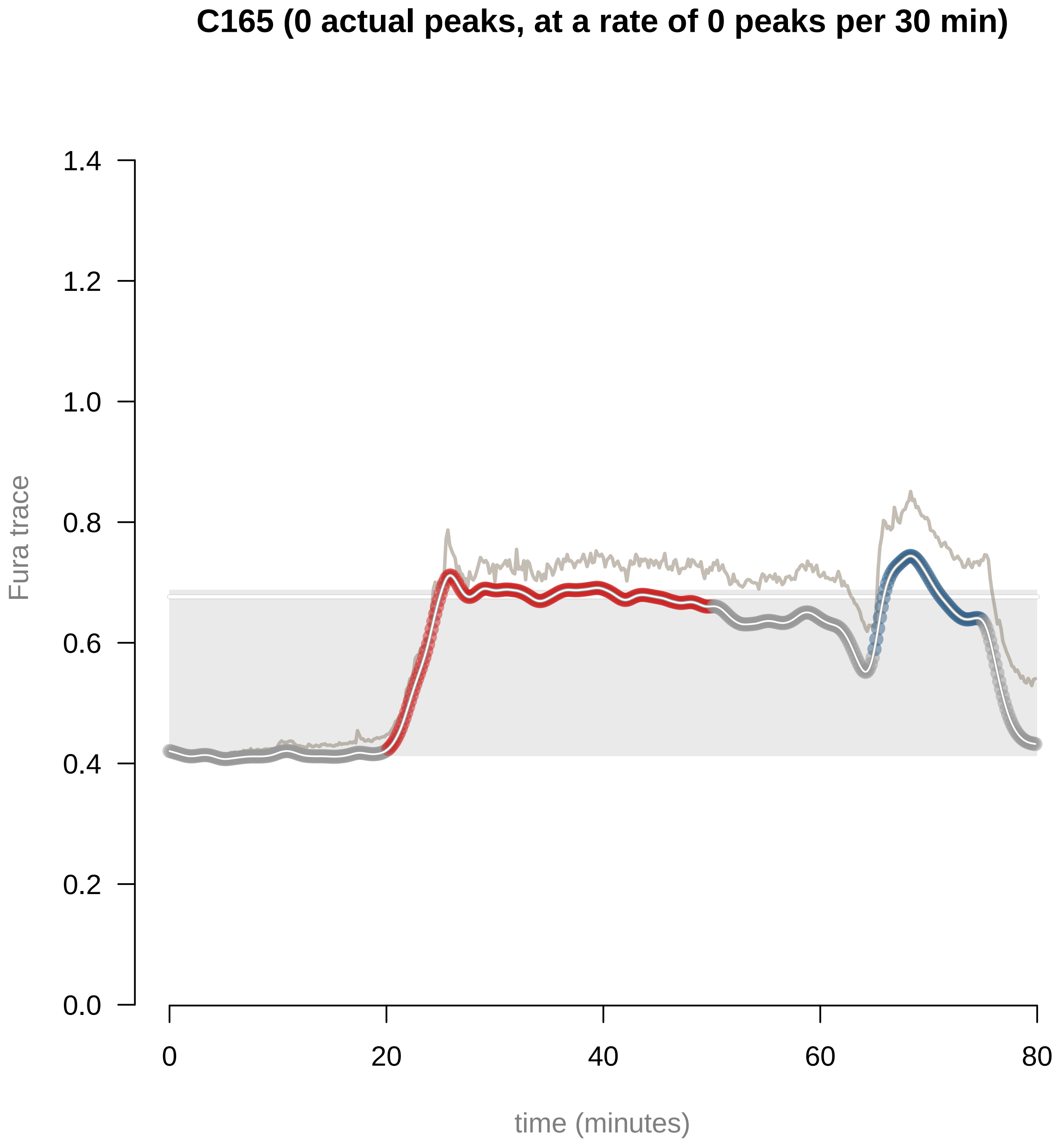
C165 (0 actual peaks, at a rate of 0 peaks per 30 min)
Fura trace
1.4
1.2
1.0
0.8
0.6
0.4
0.2
0.0
0
20
40
60
80
time (minutes)

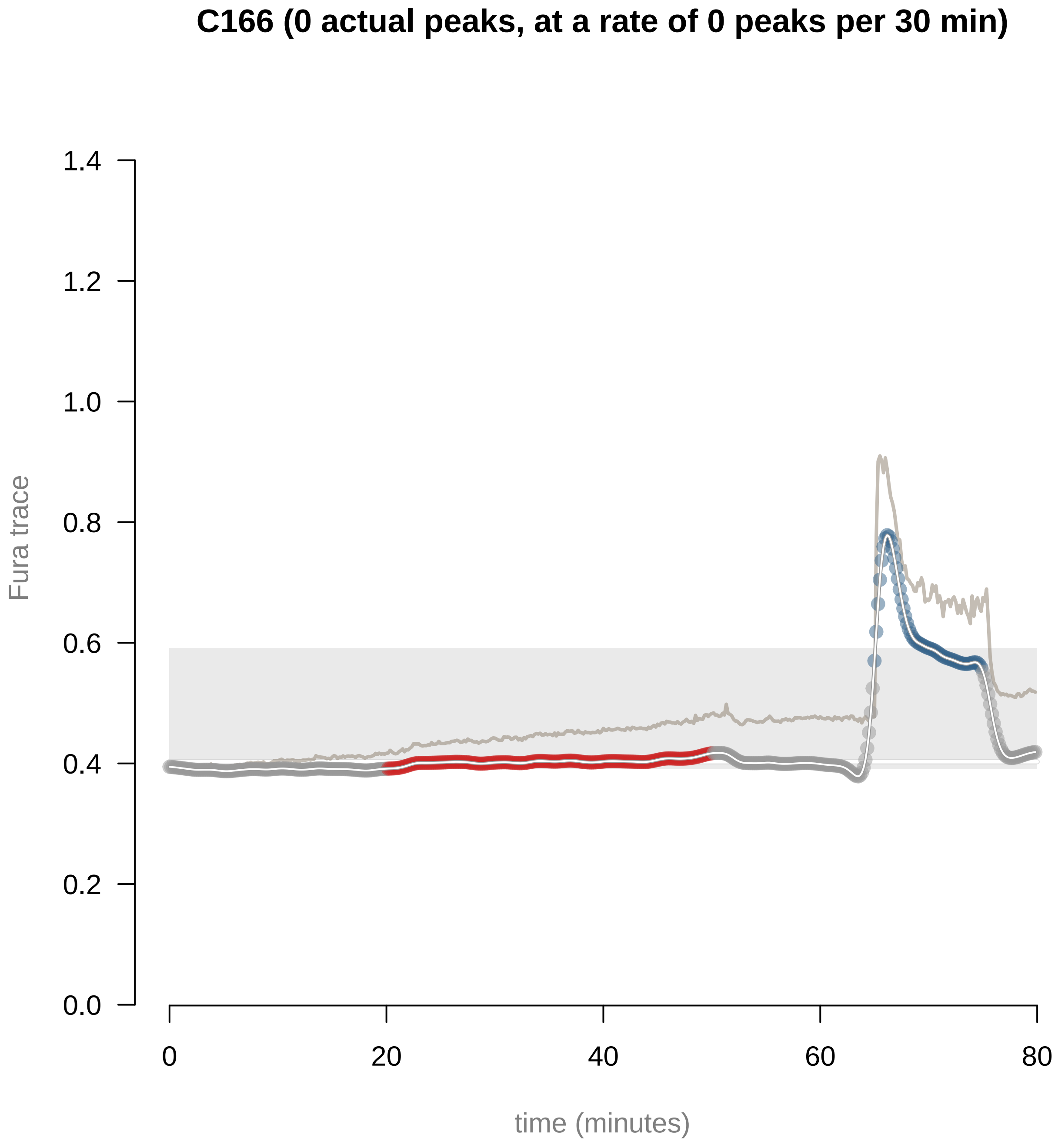

C166 (0 actual peaks, at a rate of 0 peaks per 30 min)
Fura trace
1.4
1.2
1.0
0.8
0.6
0.4
0.2
0.0
0
20
40
60
80
time (minutes)

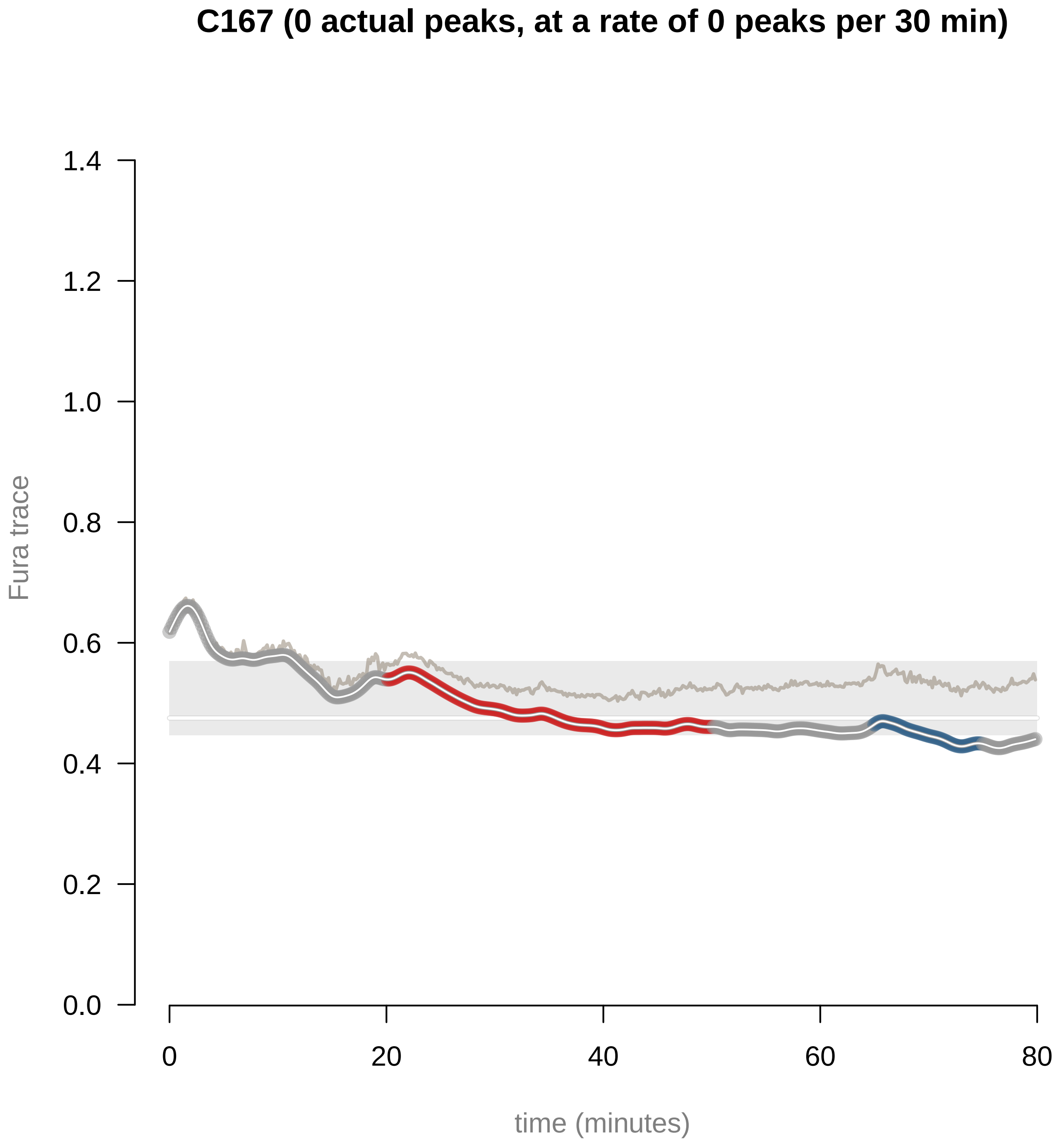
C167 (0 actual peaks, at a rate of 0 peaks per 30 min)
Fura trace
time (minutes)
0.0
0.2
0.4
0.6
0.8
1.0
1.2
1.4
0
20
40
60
80

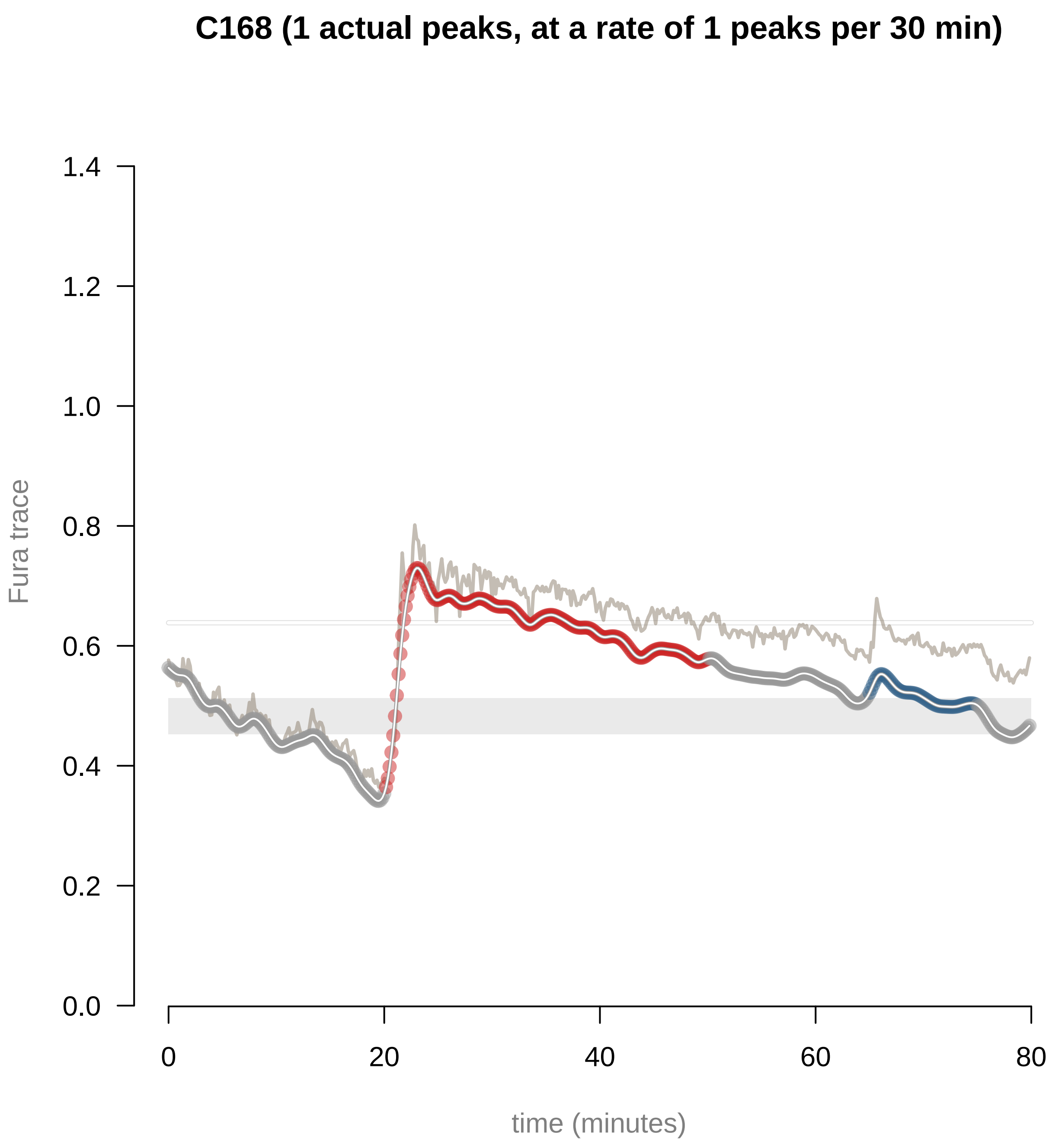
C168 (1 actual peaks, at a rate of 1 peaks per 30 min)
Fura trace
0.0
0.2
0.4
0.6
0.8
1.0
1.2
1.4
0
20
40
60
80
time (minutes)

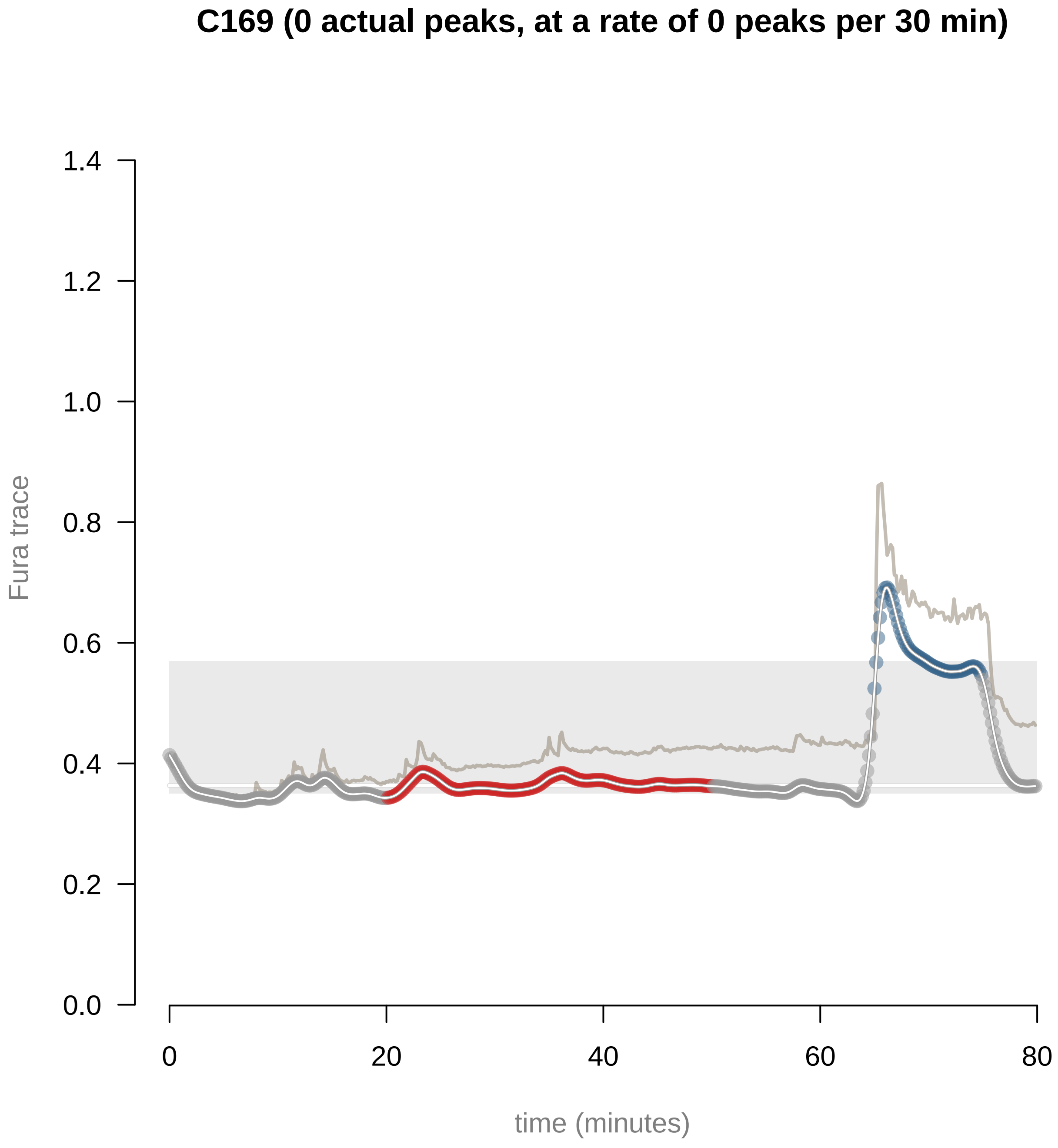
C169 (0 actual peaks, at a rate of 0 peaks per 30 min)
Fura trace
time (minutes)
0.0
0.2
0.4
0.6
0.8
1.0
1.2
1.4
0
20
40
60
80

C170 (3 actual peaks, at a rate of 3.36 peaks per 30 min)

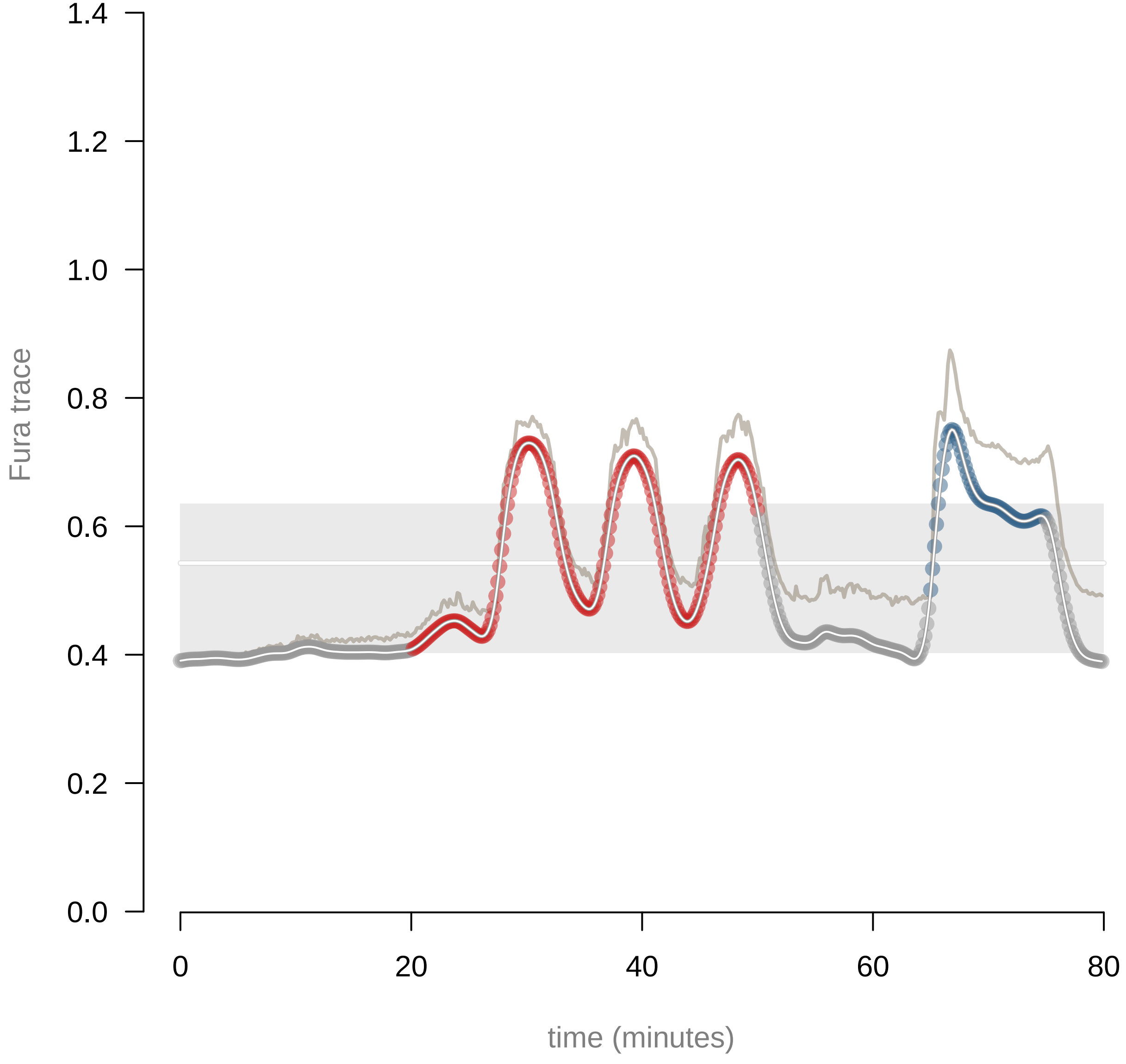

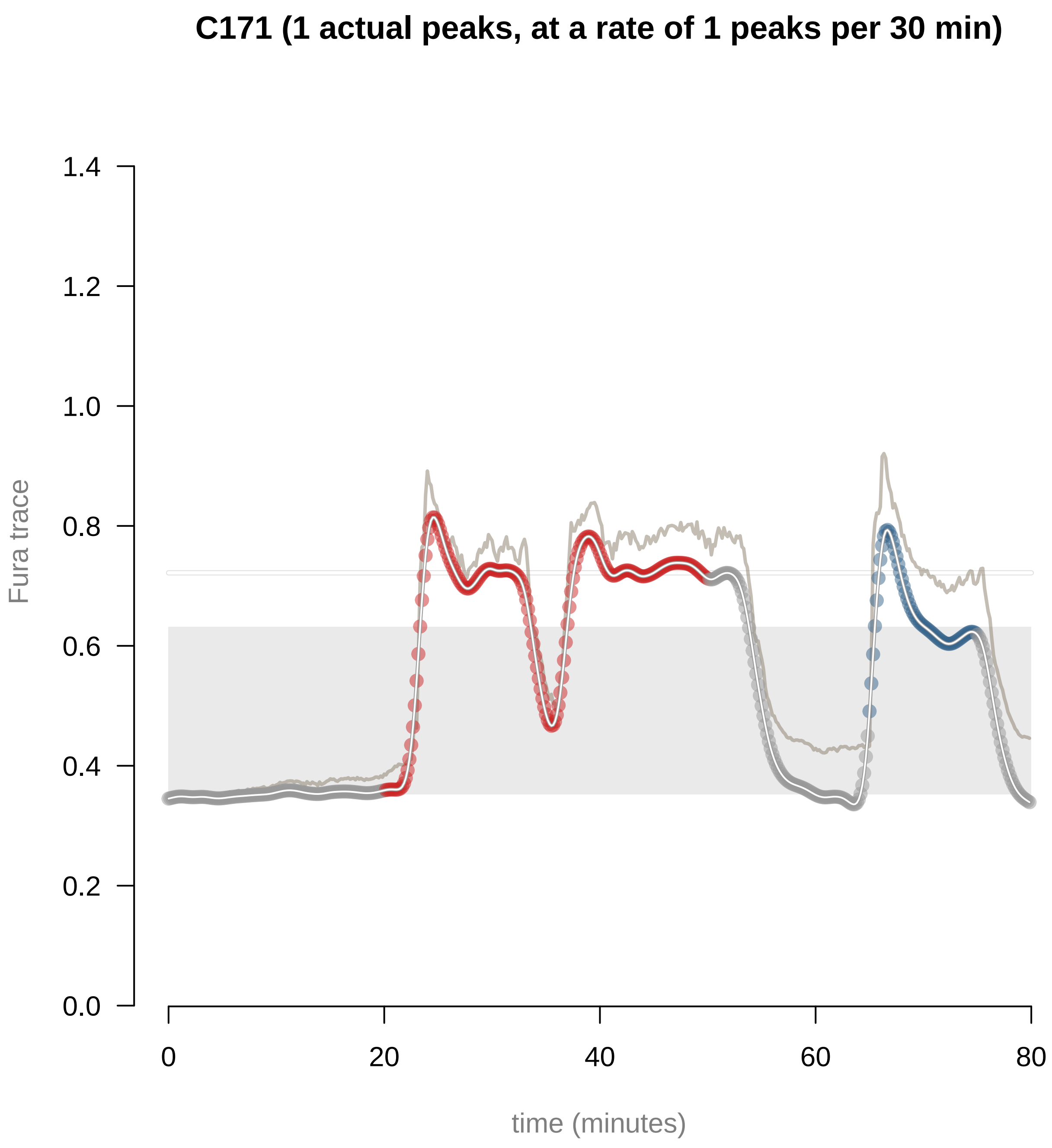
C171 (1 actual peaks, at a rate of 1 peaks per 30 min)
Fura trace
time (minutes)
0.0
0.2
0.4
0.6
0.8
1.0
1.2
1.4
0
20
40
60
80

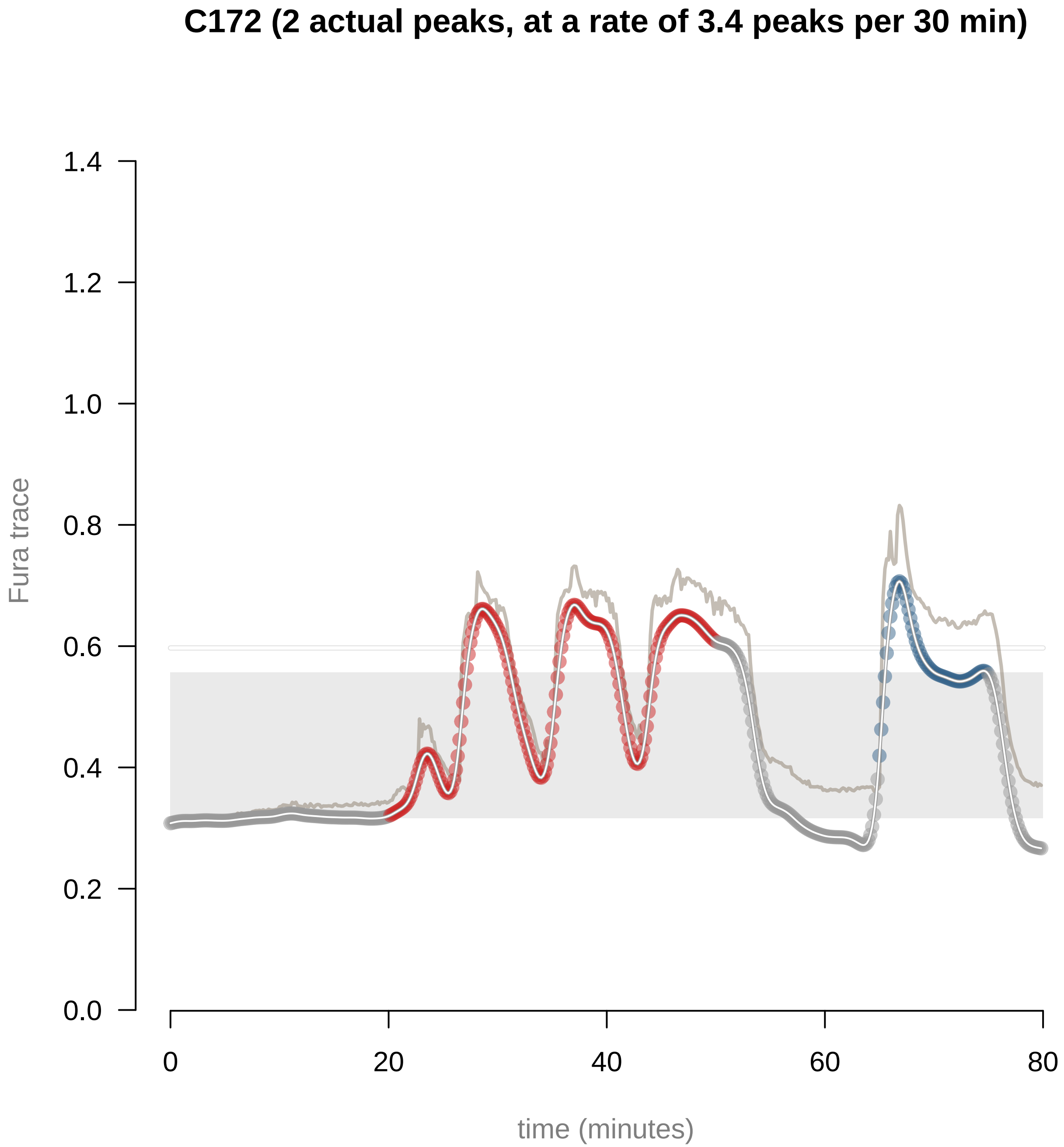
C172 (2 actual peaks, at a rate of 3.4 peaks per 30 min)
Fura trace
1.4
1.2
1.0
0.8
0.6
0.4
0.2
0.0
0
20
40
60
80
time (minutes)

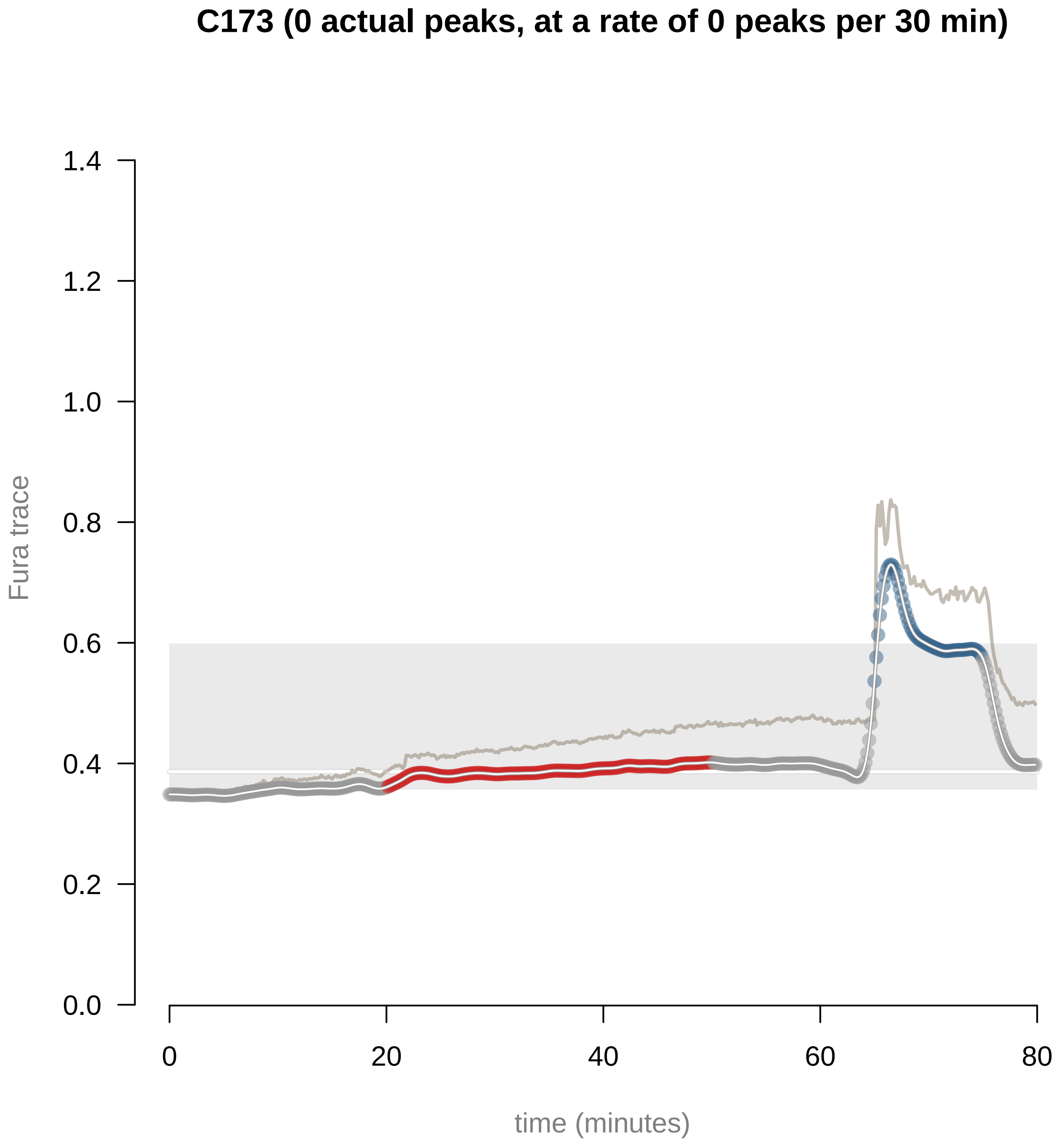
C173 (0 actual peaks, at a rate of 0 peaks per 30 min)
Fura trace
0.0
0.2
0.4
0.6
0.8
1.0
1.2
1.4
0
20
40
60
80
time (minutes)

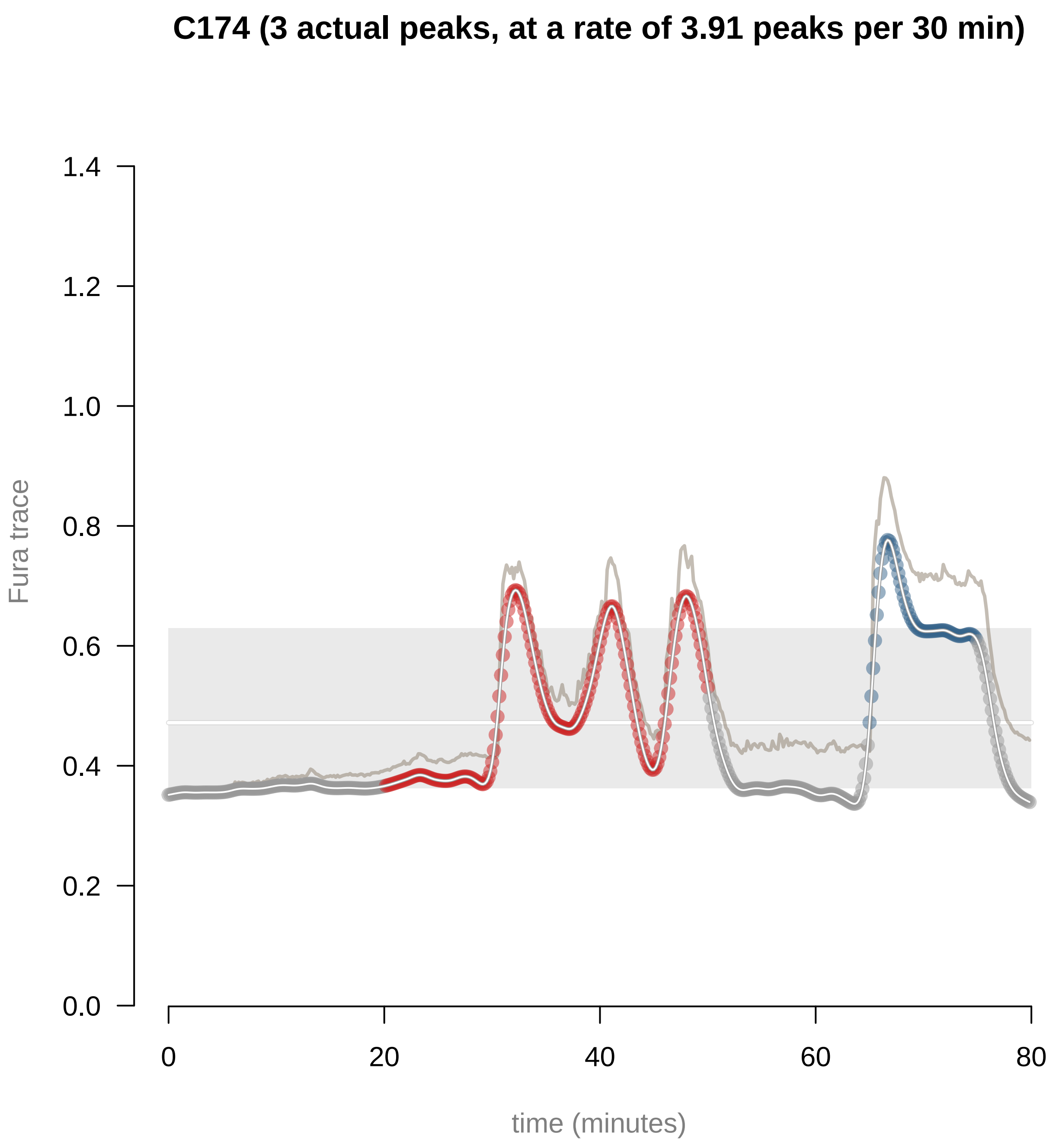

C174 (3 actual peaks, at a rate of 3.91 peaks per 30 min)
Fura trace
time (minutes)
1.4
1.2
1.0
0.8
0.6
0.4
0.2
0.0
0
20
40
60
80

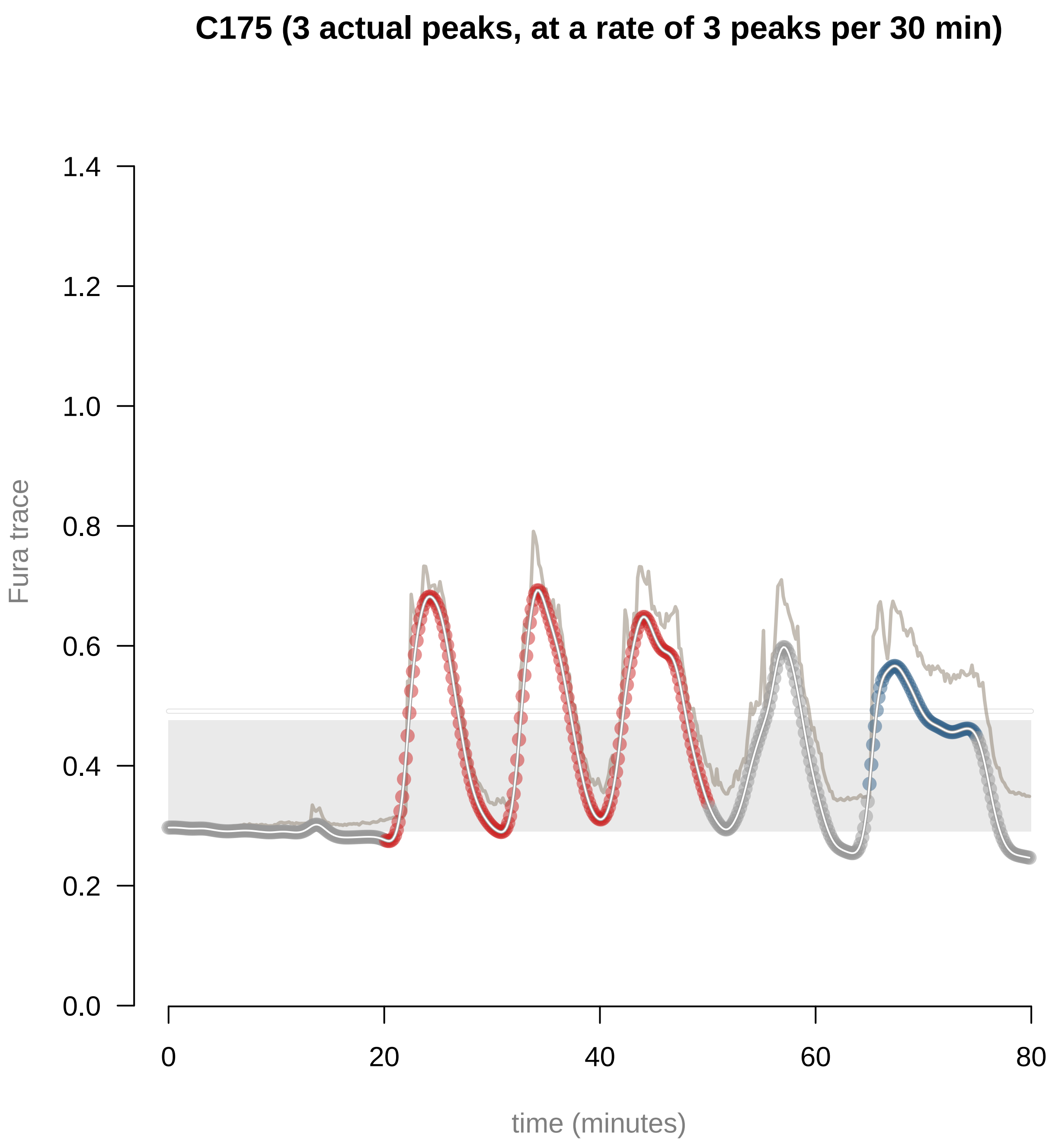
C175 (3 actual peaks, at a rate of 3 peaks per 30 min)
Fura trace
1.4
1.2
1.0
0.8
0.6
0.4
0.2
0.0
0
20
40
60
80
time (minutes)

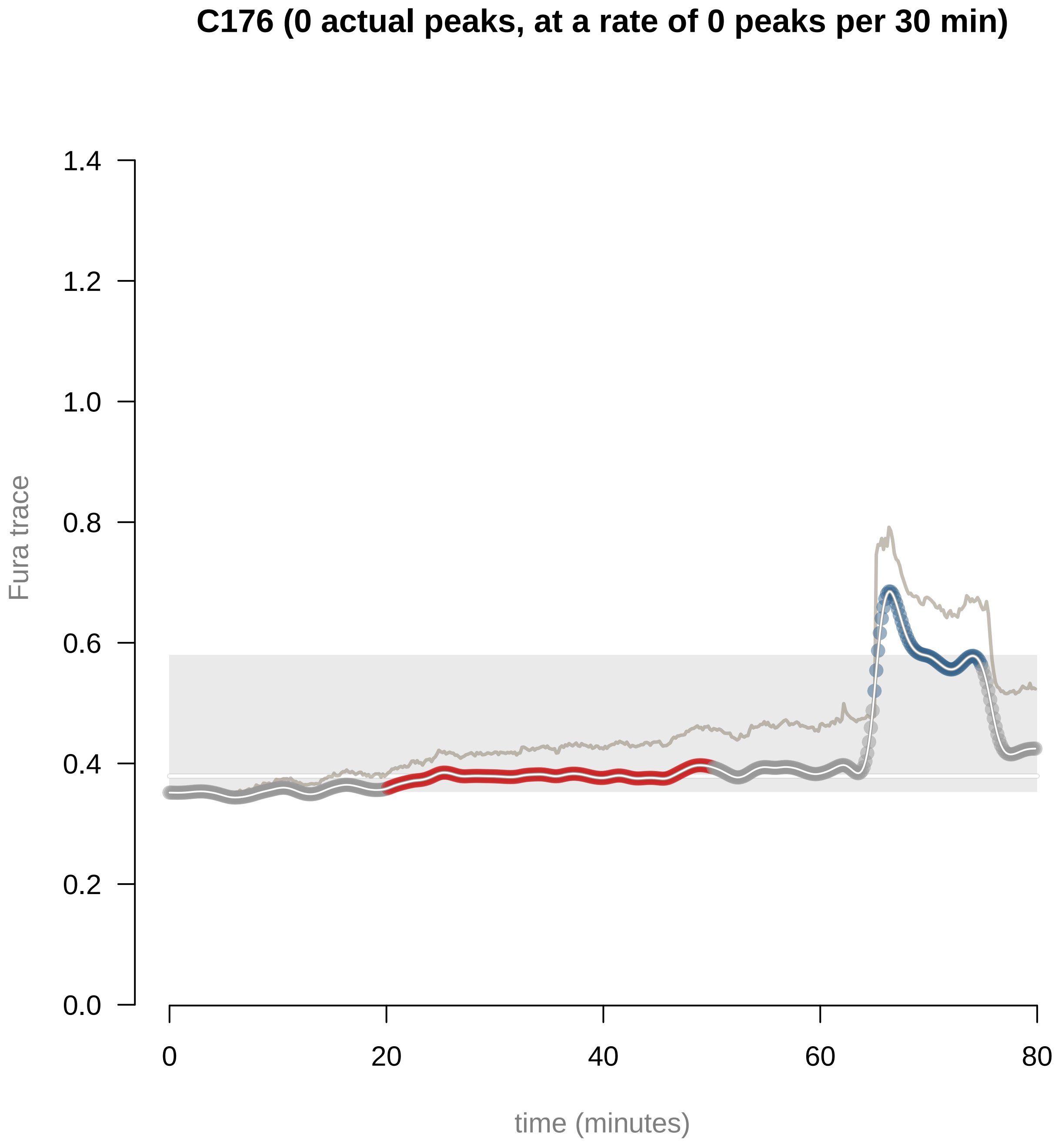
C176 (0 actual peaks, at a rate of 0 peaks per 30 min)
Fura trace
0.0
0.2
0.4
0.6
0.8
1.0
1.2
1.4
0
20
40
60
80
time (minutes)

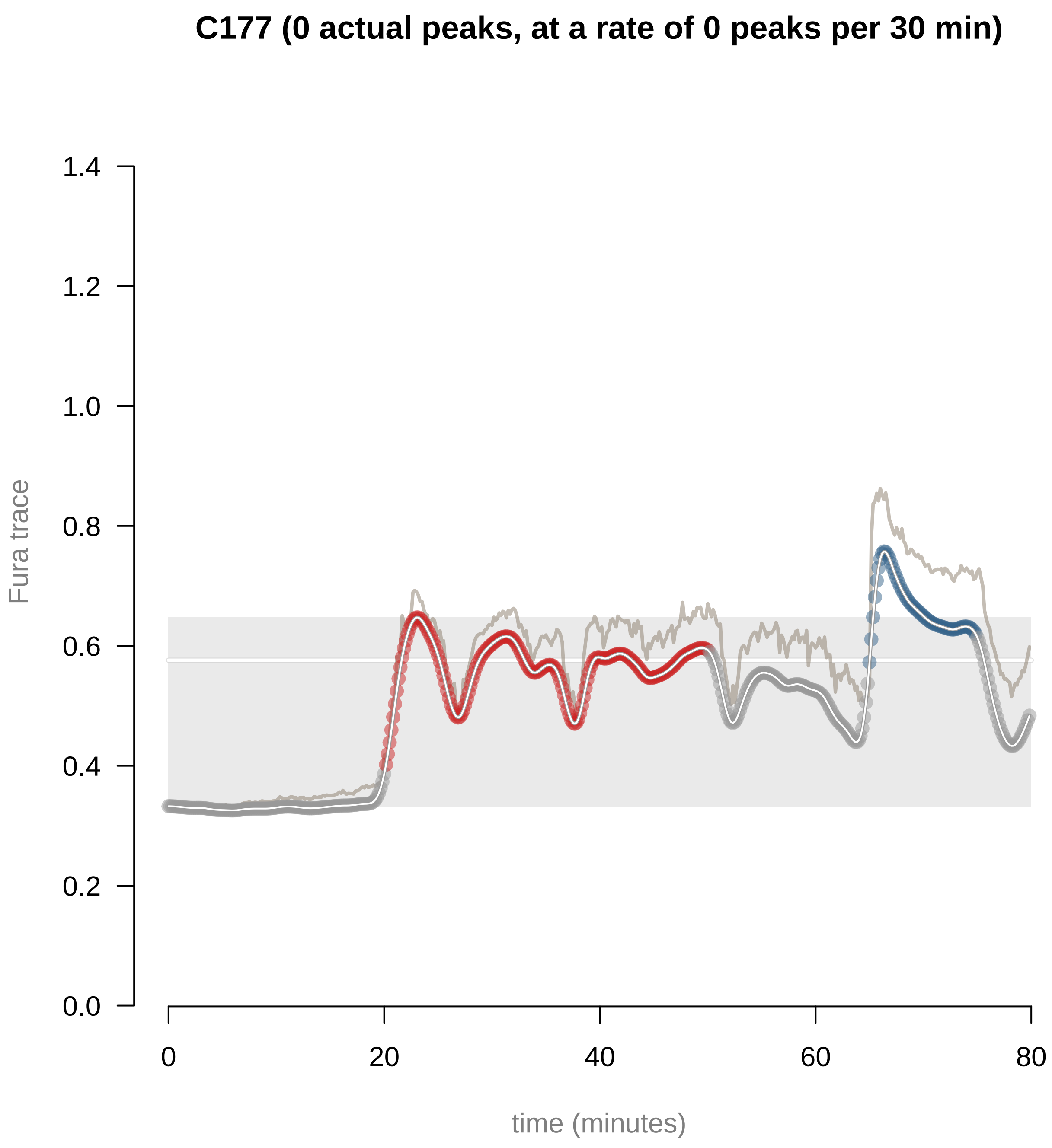

C177 (0 actual peaks, at a rate of 0 peaks per 30 min)
Fura trace
1.4
1.2
1.0
0.8
0.6
0.4
0.2
0.0
0
20
40
60
80
time (minutes)

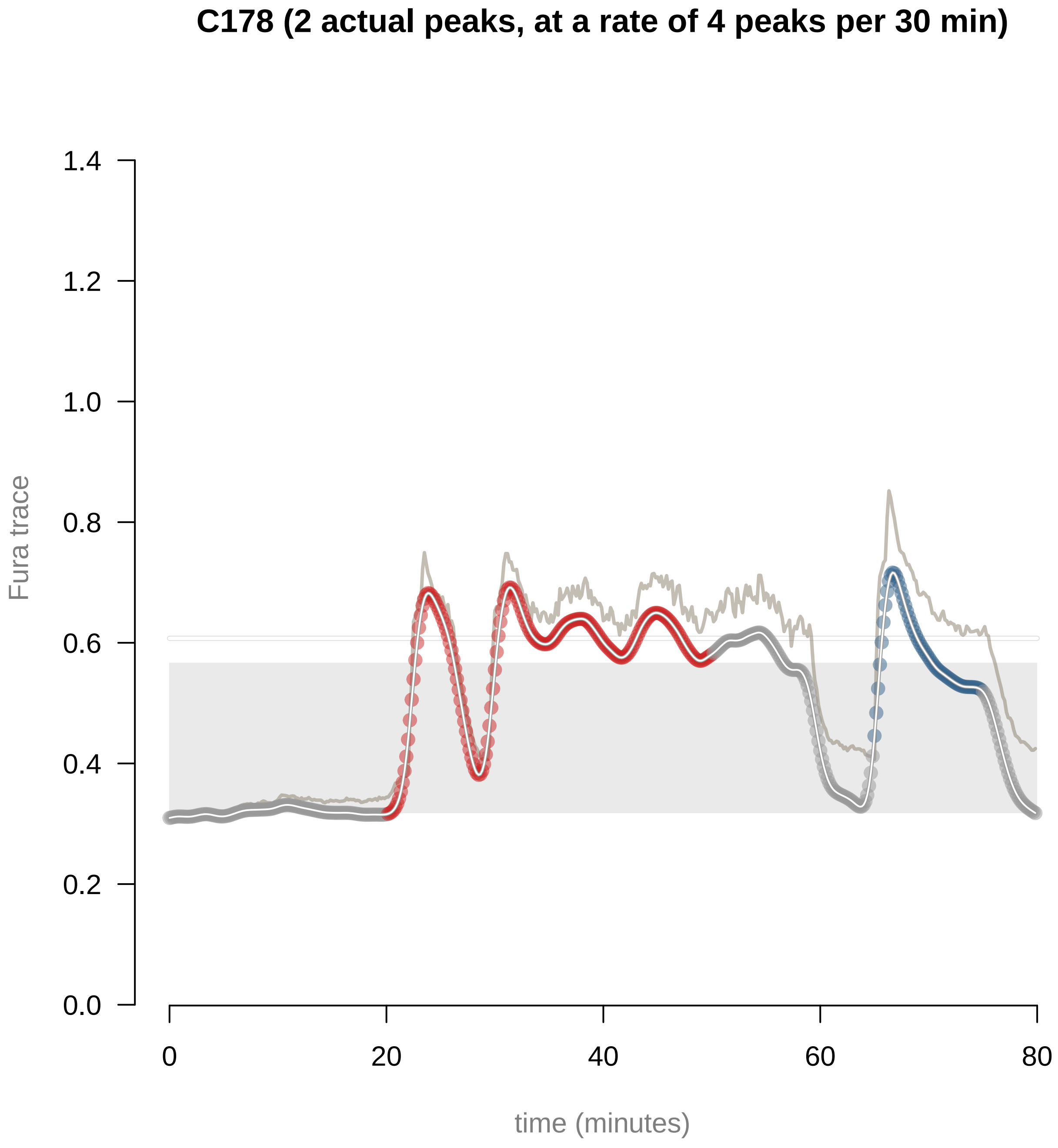
C178 (2 actual peaks, at a rate of 4 peaks per 30 min)
Fura trace
0.0
0.2
0.4
0.6
0.8
1.0
1.2
1.4
0
20
40
60
80
time (minutes)

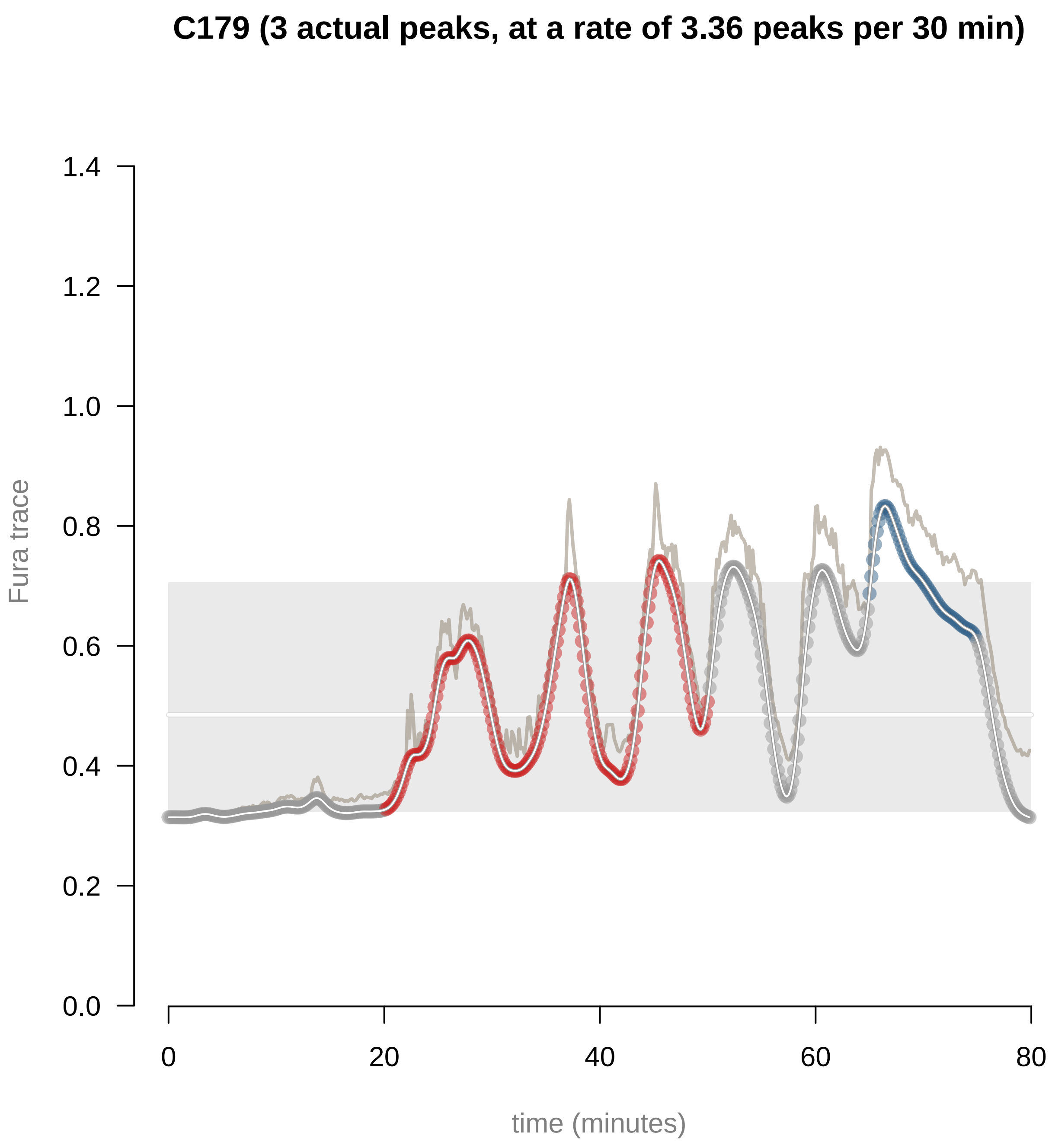

C179 (3 actual peaks, at a rate of 3.36 peaks per 30 min)
1.4
1.2
1.0
0.8
0.6
0.4
0.2
0.0
Fura trace
0
20
40
60
80
time (minutes)

C180 (2 actual peaks, at a rate of 1.15 peaks per 30 min)

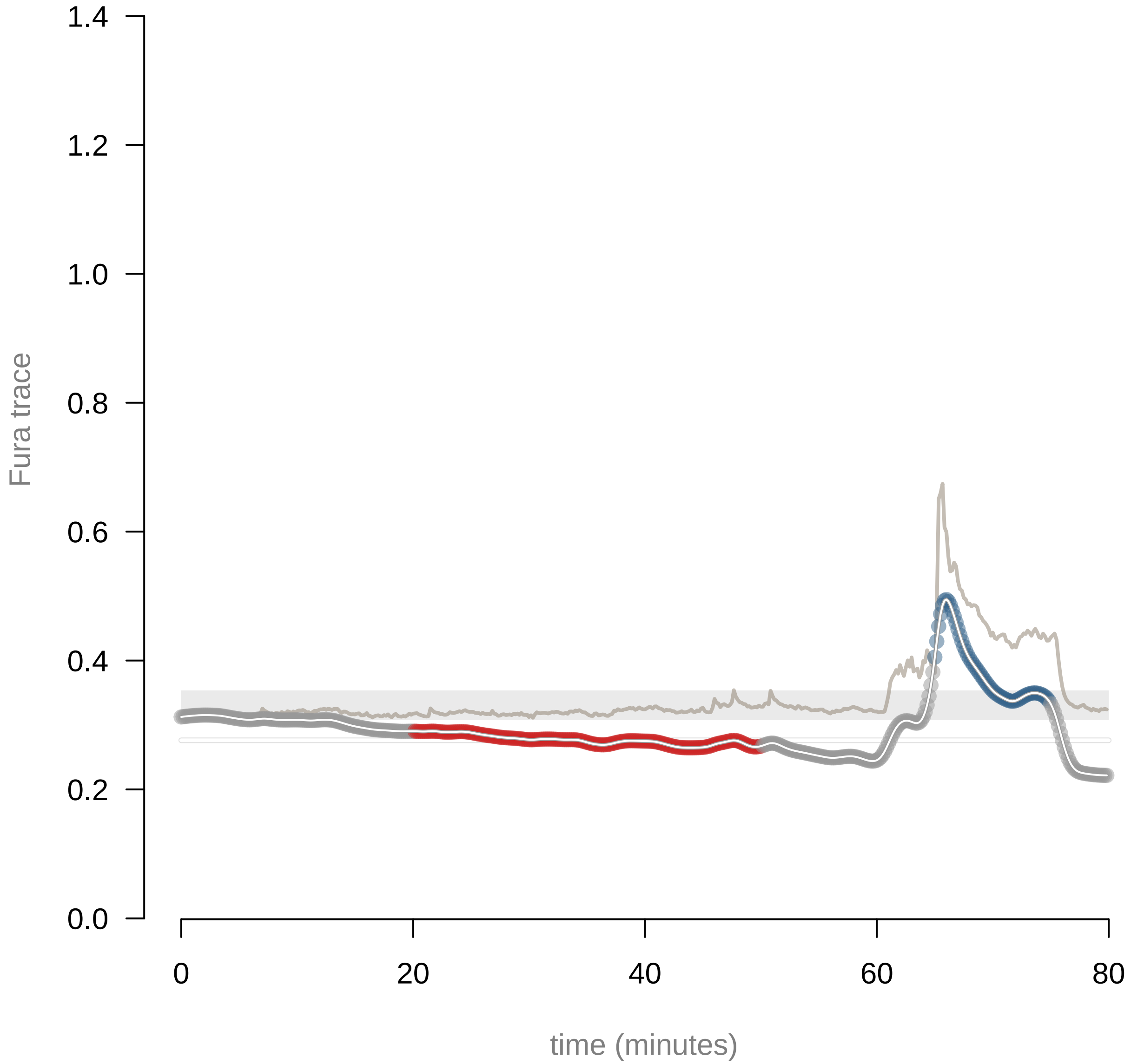

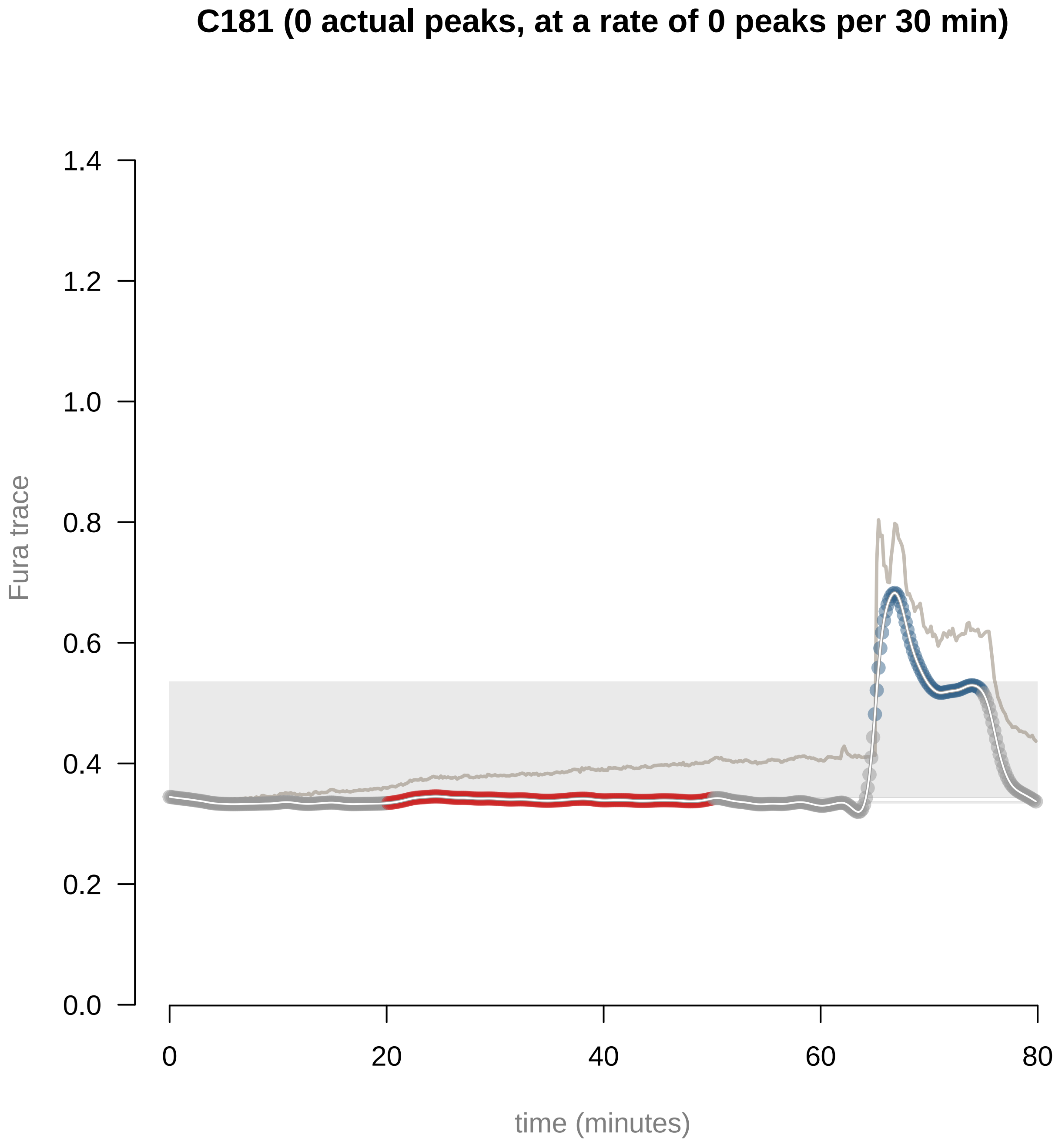

C181 (0 actual peaks, at a rate of 0 peaks per 30 min)
Fura trace
1.4
1.2
1.0
0.8
0.6
0.4
0.2
0.0
0
20
40
60
80
time (minutes)

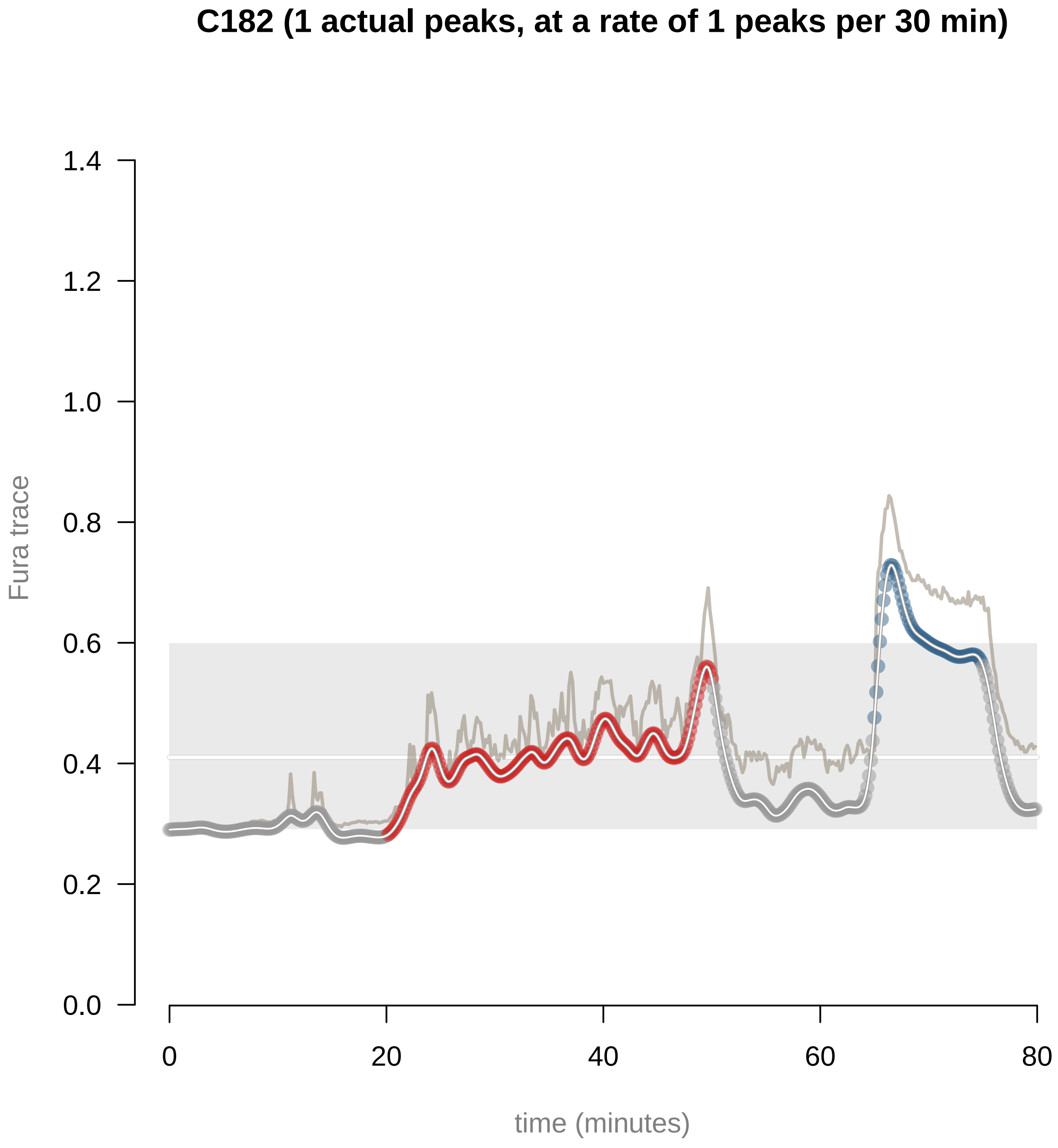

C182 (1 actual peaks, at a rate of 1 peaks per 30 min)
Fura trace
1.4
1.2
1.0
0.8
0.6
0.4
0.2
0.0
0
20
40
60
80
time (minutes)

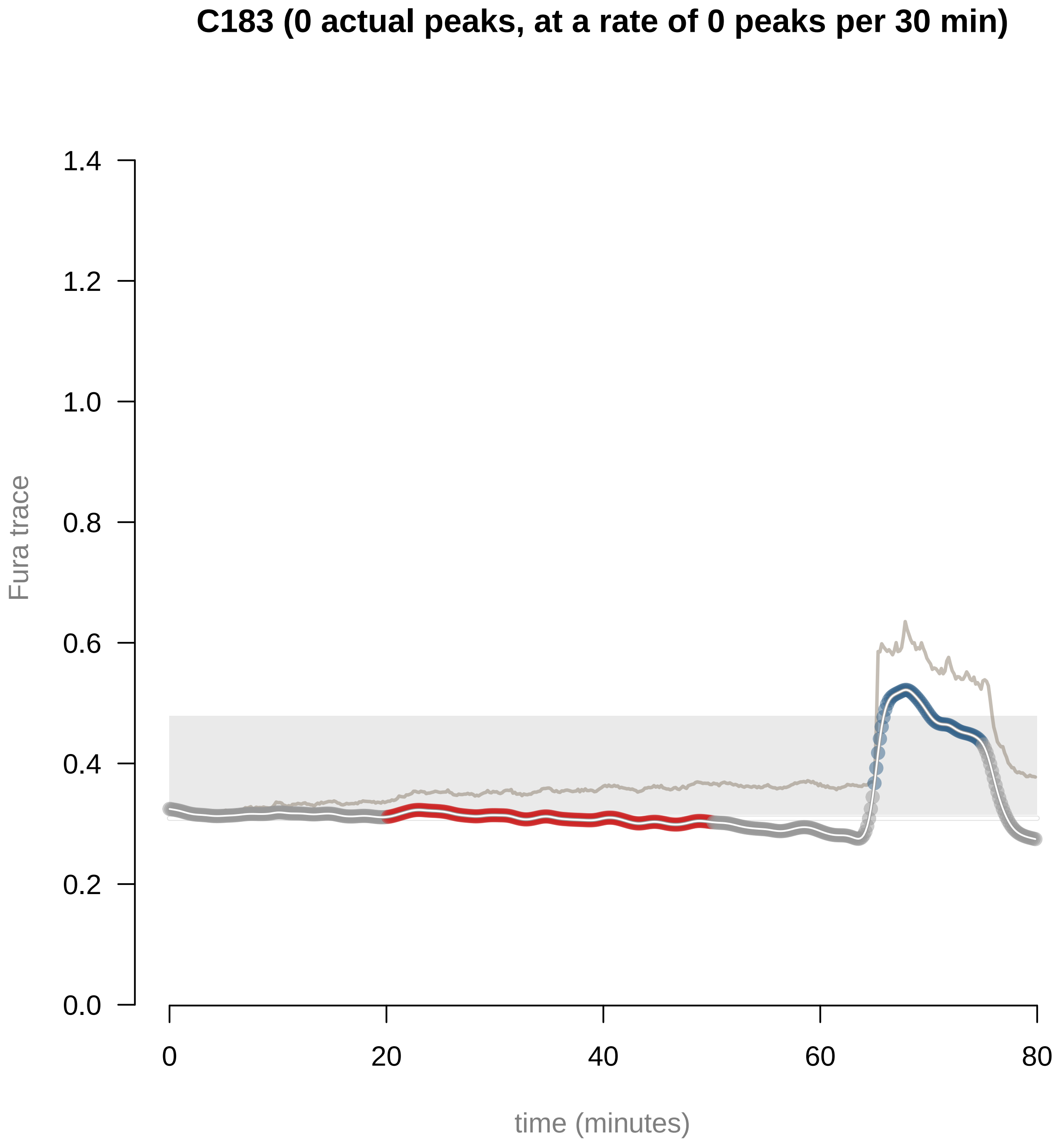

C183 (0 actual peaks, at a rate of 0 peaks per 30 min)
1.4
1.2
1.0
0.8
0.6
0.4
0.2
0.0
Fura trace
0
20
40
60
80
time (minutes)

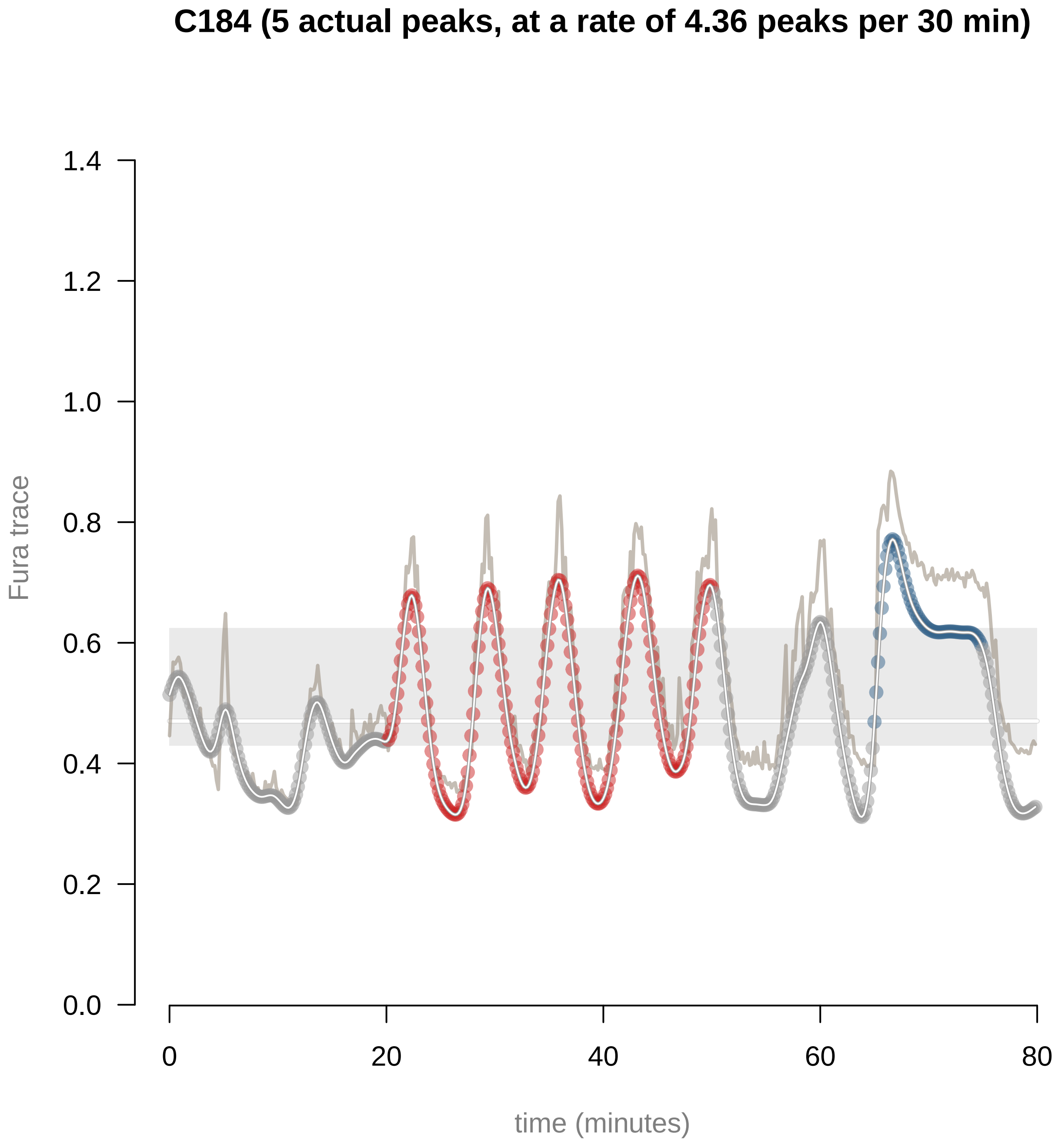
C184 (5 actual peaks, at a rate of 4.36 peaks per 30 min)
Fura trace
1.4
1.2
1.0
0.8
0.6
0.4
0.2
0.0
0
20
40
60
80
time (minutes)

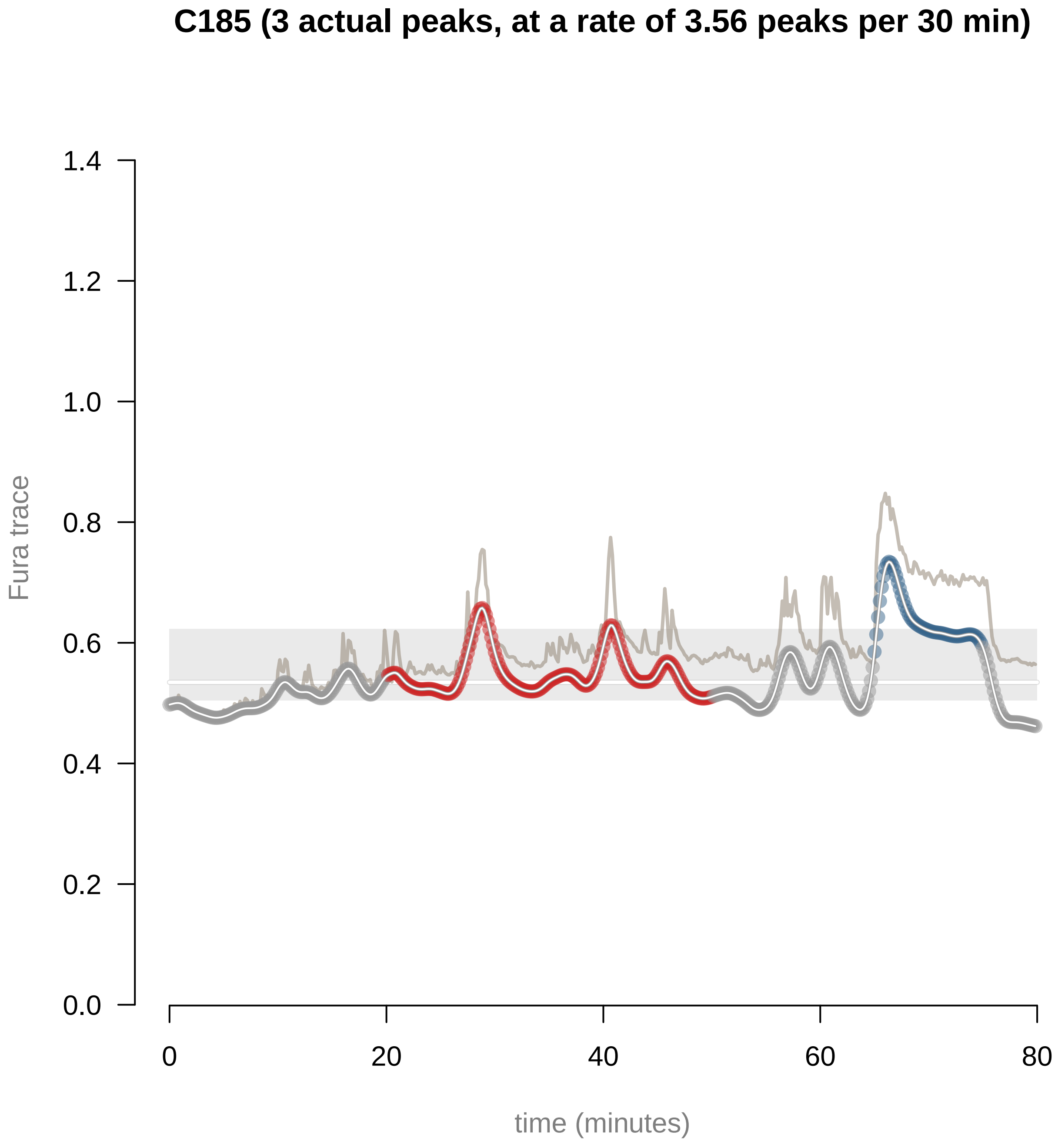
C185 (3 actual peaks, at a rate of 3.56 peaks per 30 min)
Fura trace
1.4
1.2
1.0
0.8
0.6
0.4
0.2
0.0
0
20
40
60
80
time (minutes)

C186 (3 actual peaks, at a rate of 3.33 peaks per 30 min)

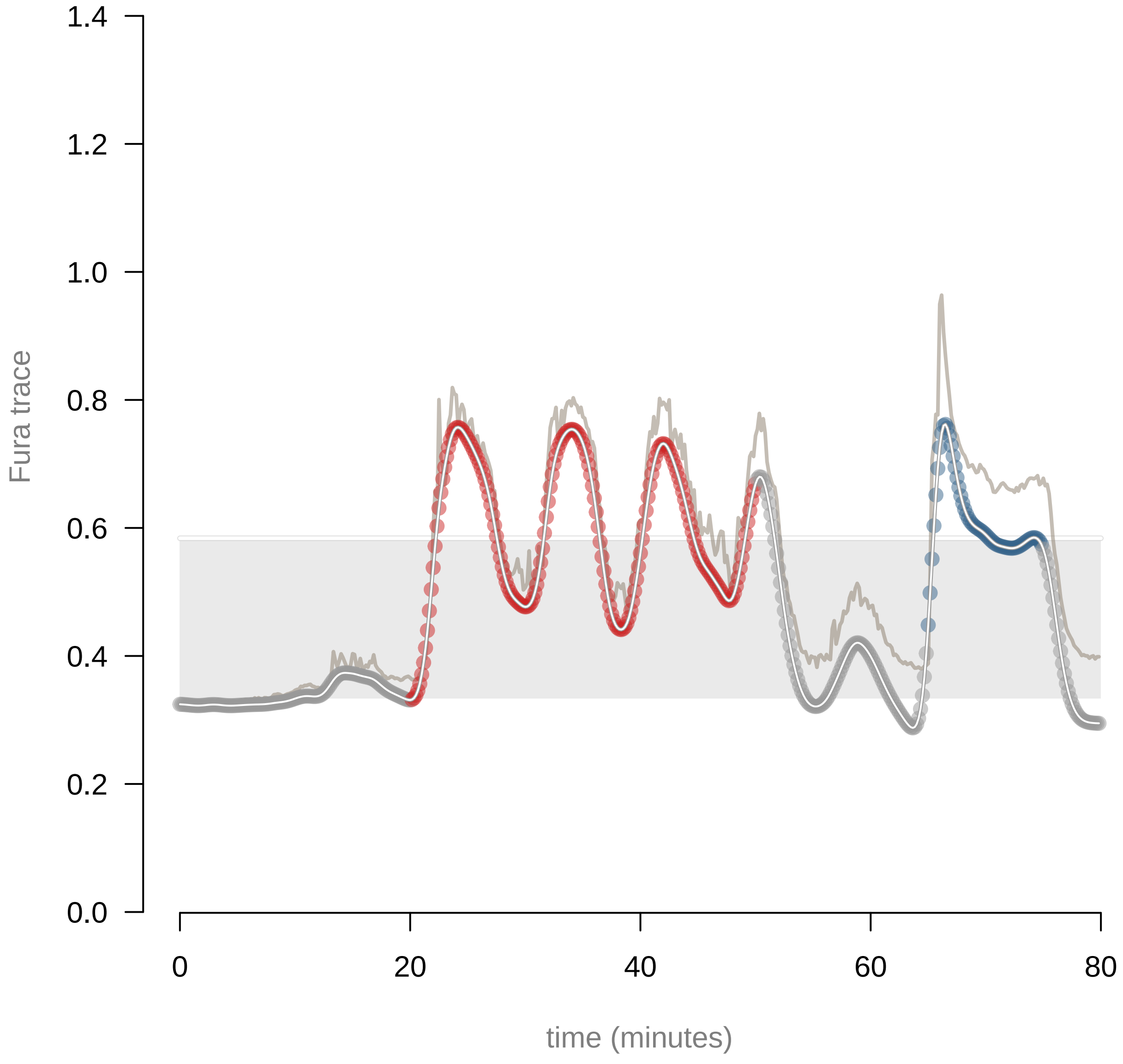

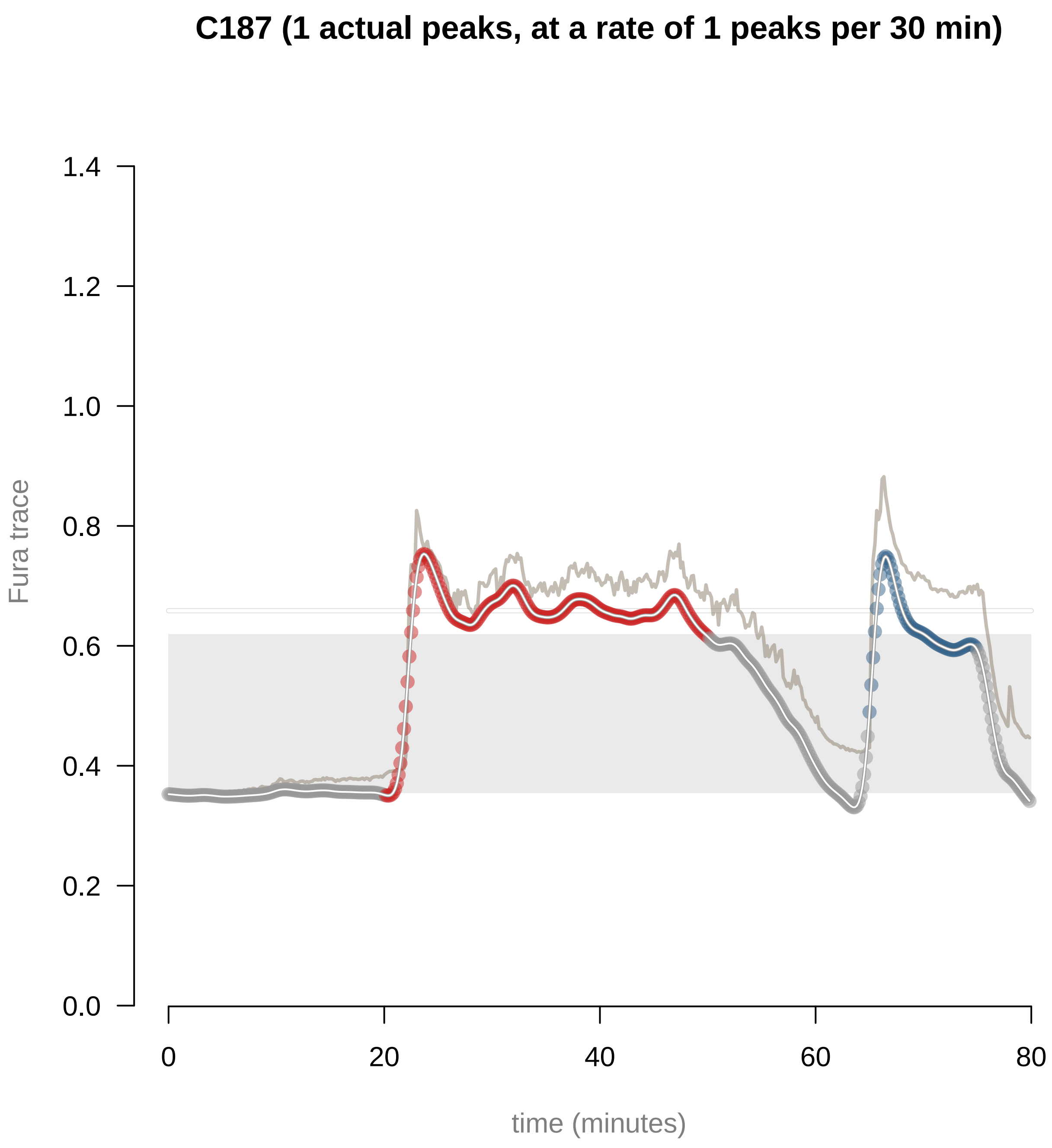

C187 (1 actual peaks, at a rate of 1 peaks per 30 min)
Fura trace
time (minutes)
0.0
0.2
0.4
0.6
0.8
1.0
1.2
1.4
0
20
40
60
80

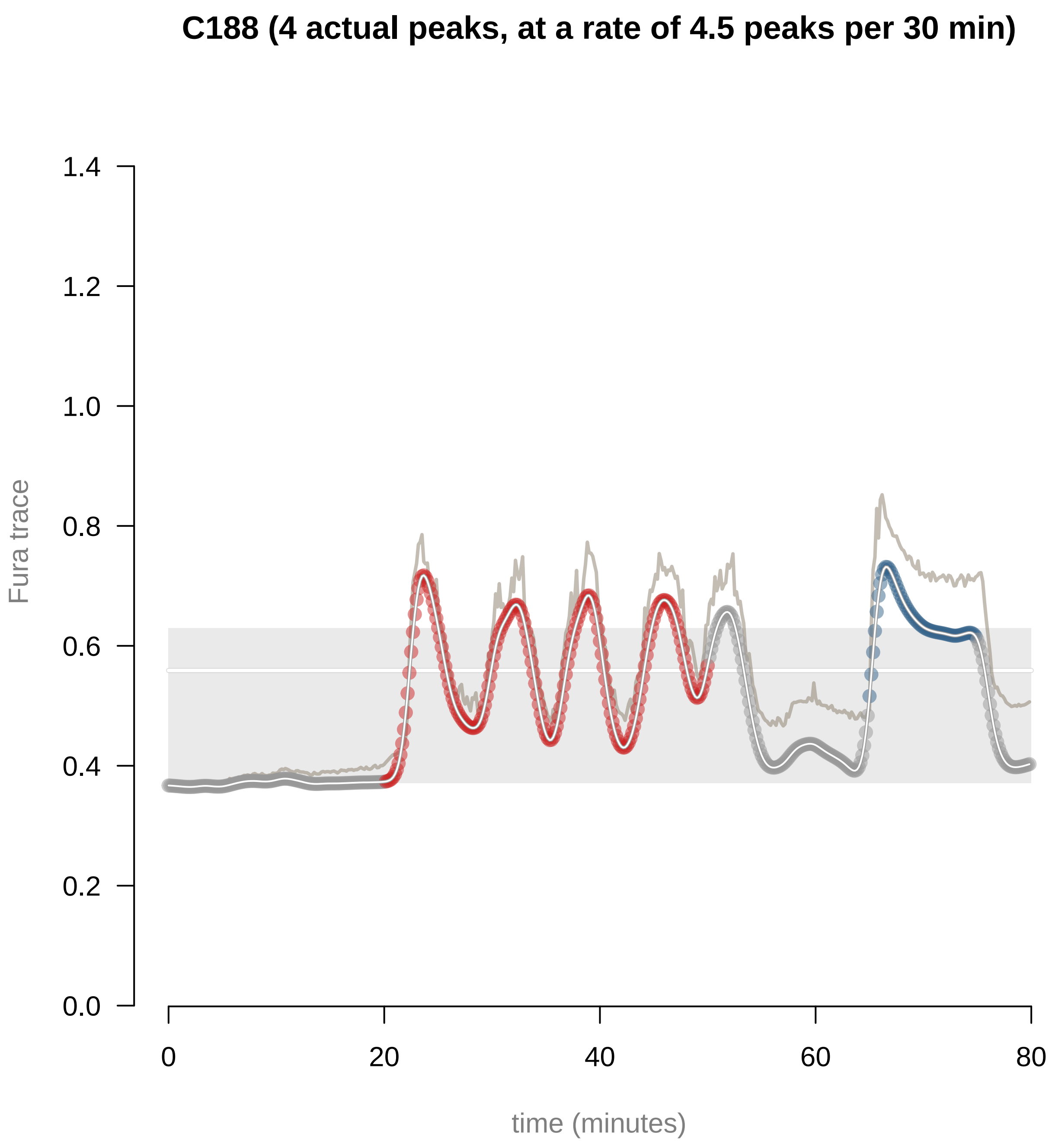

C188 (4 actual peaks, at a rate of 4.5 peaks per 30 min)
Fura trace
1.4
1.2
1.0
0.8
0.6
0.4
0.2
0.0
0
20
40
60
80
time (minutes)

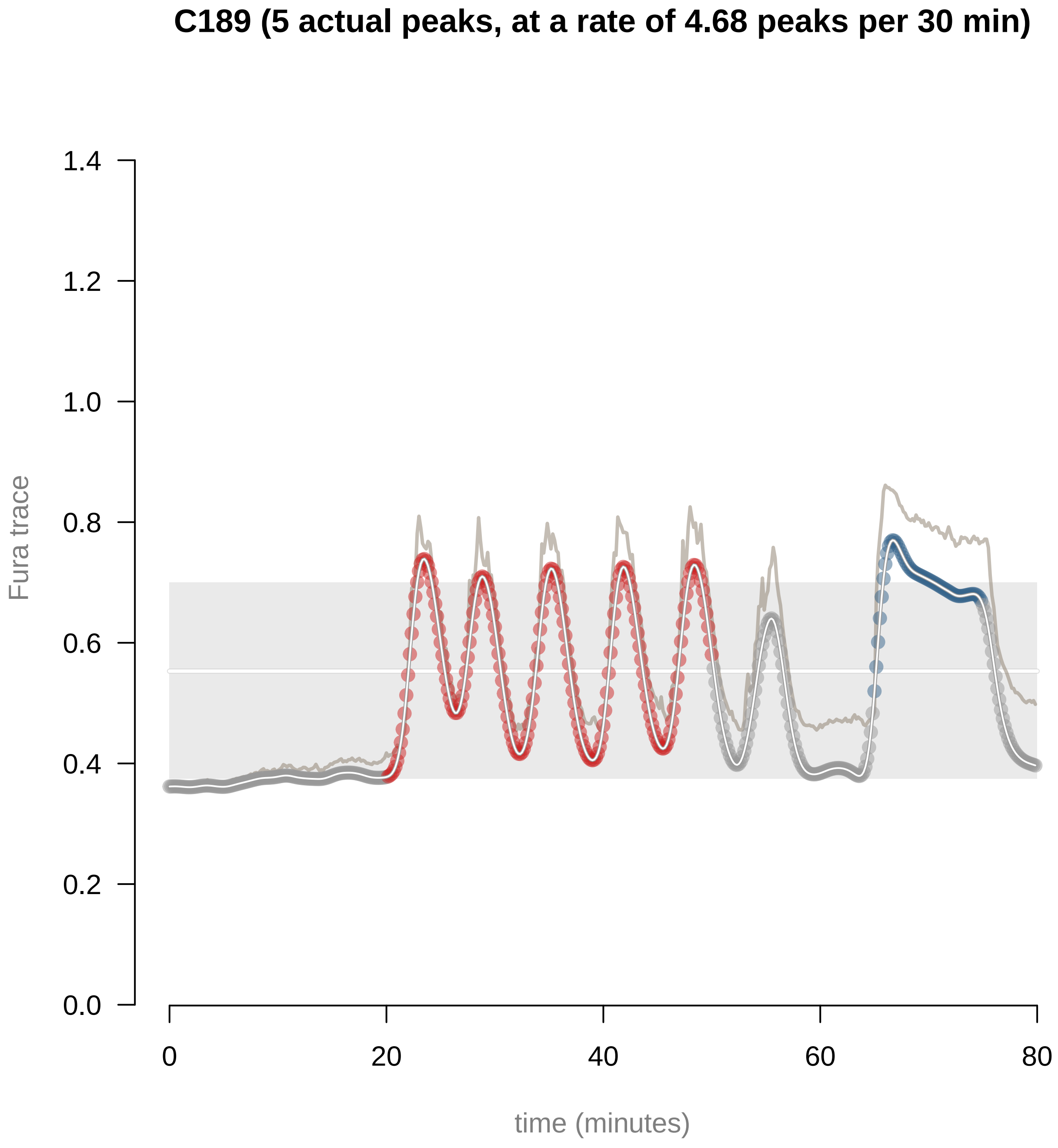
C189 (5 actual peaks, at a rate of 4.68 peaks per 30 min)
Fura trace
time (minutes)
0.0
0.2
0.4
0.6
0.8
1.0
1.2
1.4
0
20
40
60
80

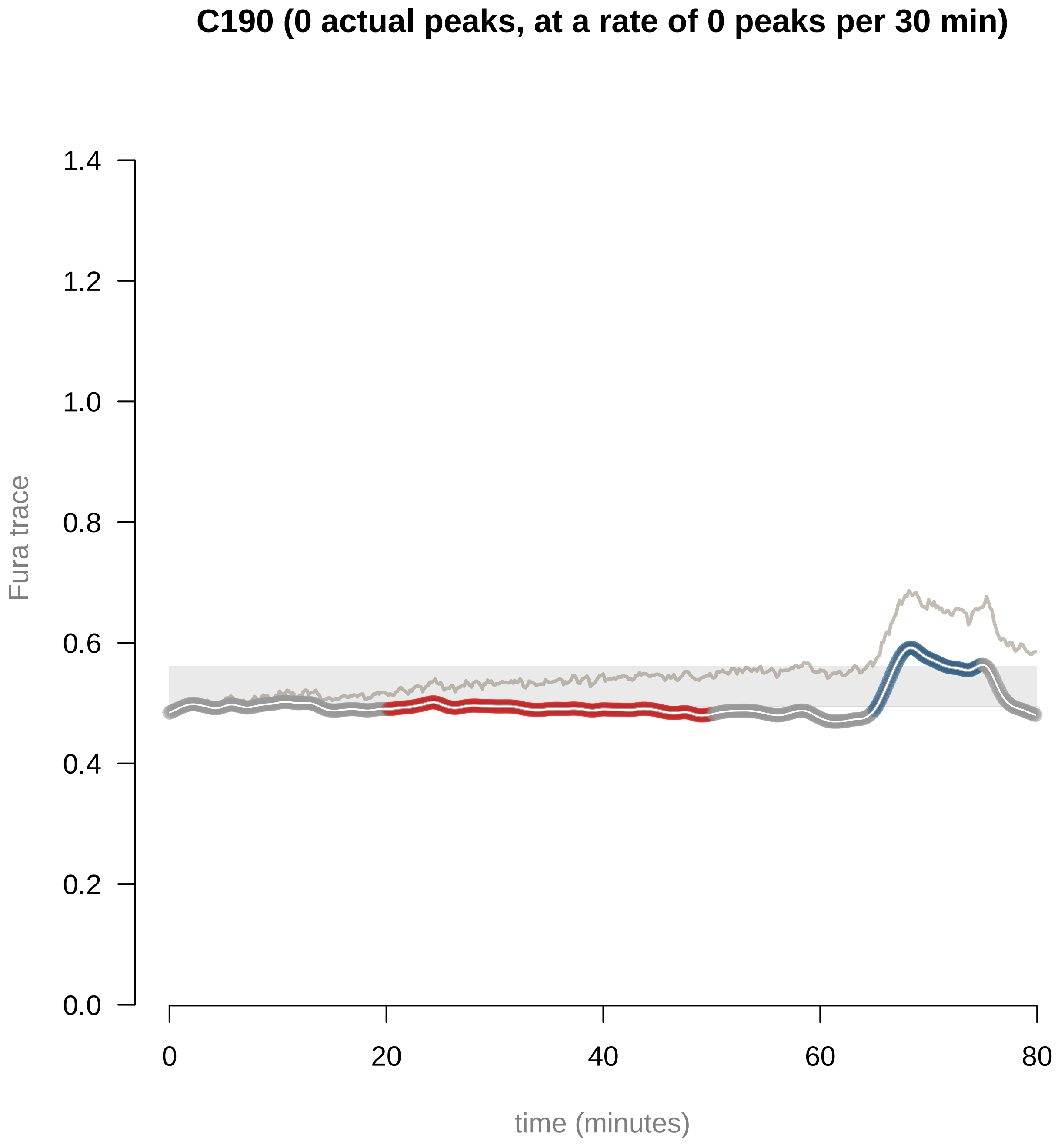
C190 (0 actual peaks, at a rate of 0 peaks per 30 min)
1.4
1.2
1.0
0.8
0.6
0.4
0.2
0.0
Fura trace
0
20
40
60
80
time (minutes)

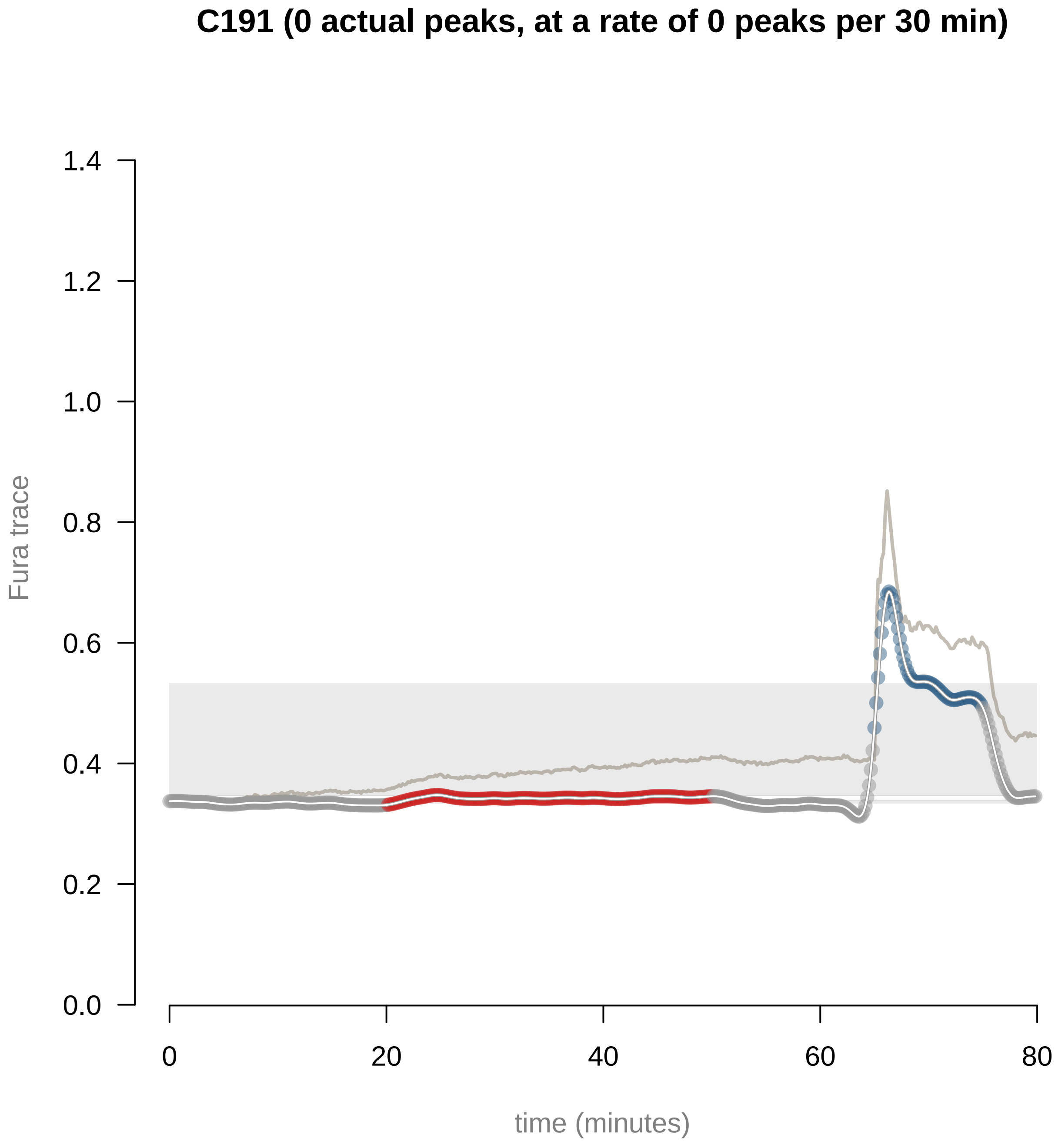
C191 (0 actual peaks, at a rate of 0 peaks per 30 min)
Fura trace
1.4
1.2
1.0
0.8
0.6
0.4
0.2
0.0
0
20
40
60
80
time (minutes)

C192 (3 actual peaks, at a rate of 3.33 peaks per 30 min)

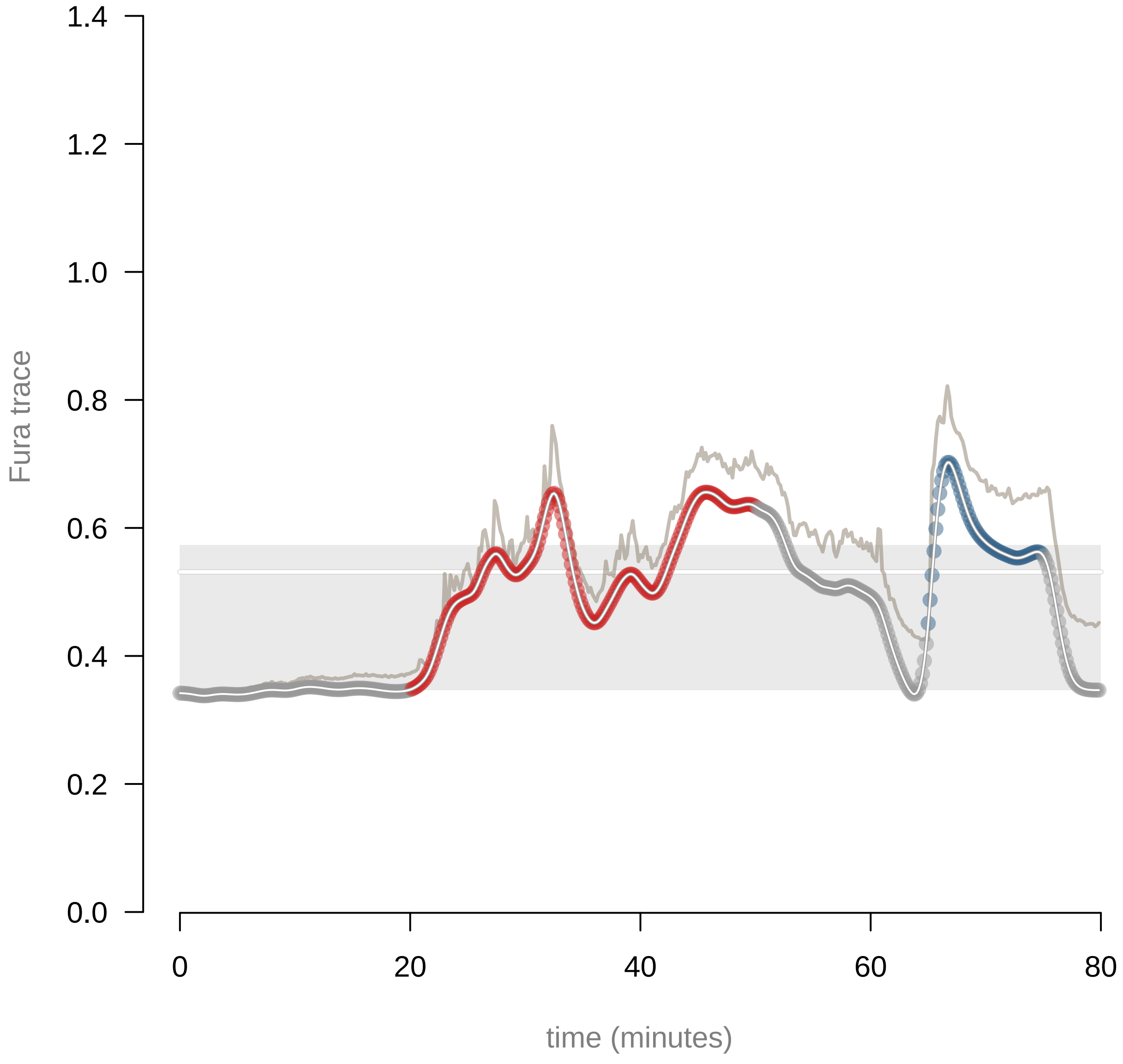

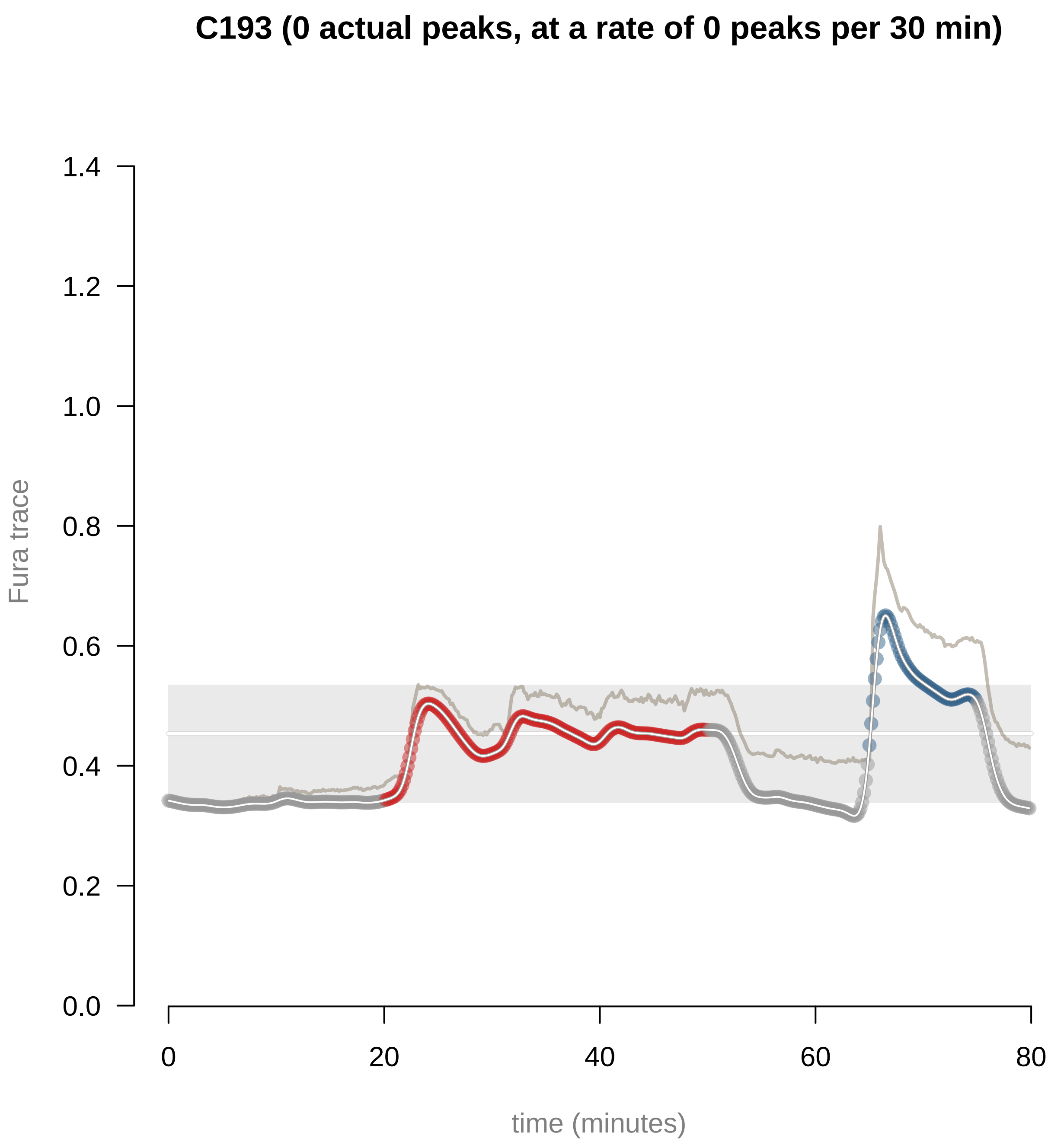
C193 (0 actual peaks, at a rate of 0 peaks per 30 min)
Fura trace
0.0
0.2
0.4
0.6
0.8
1.0
1.2
1.4
0
20
40
60
80
time (minutes)

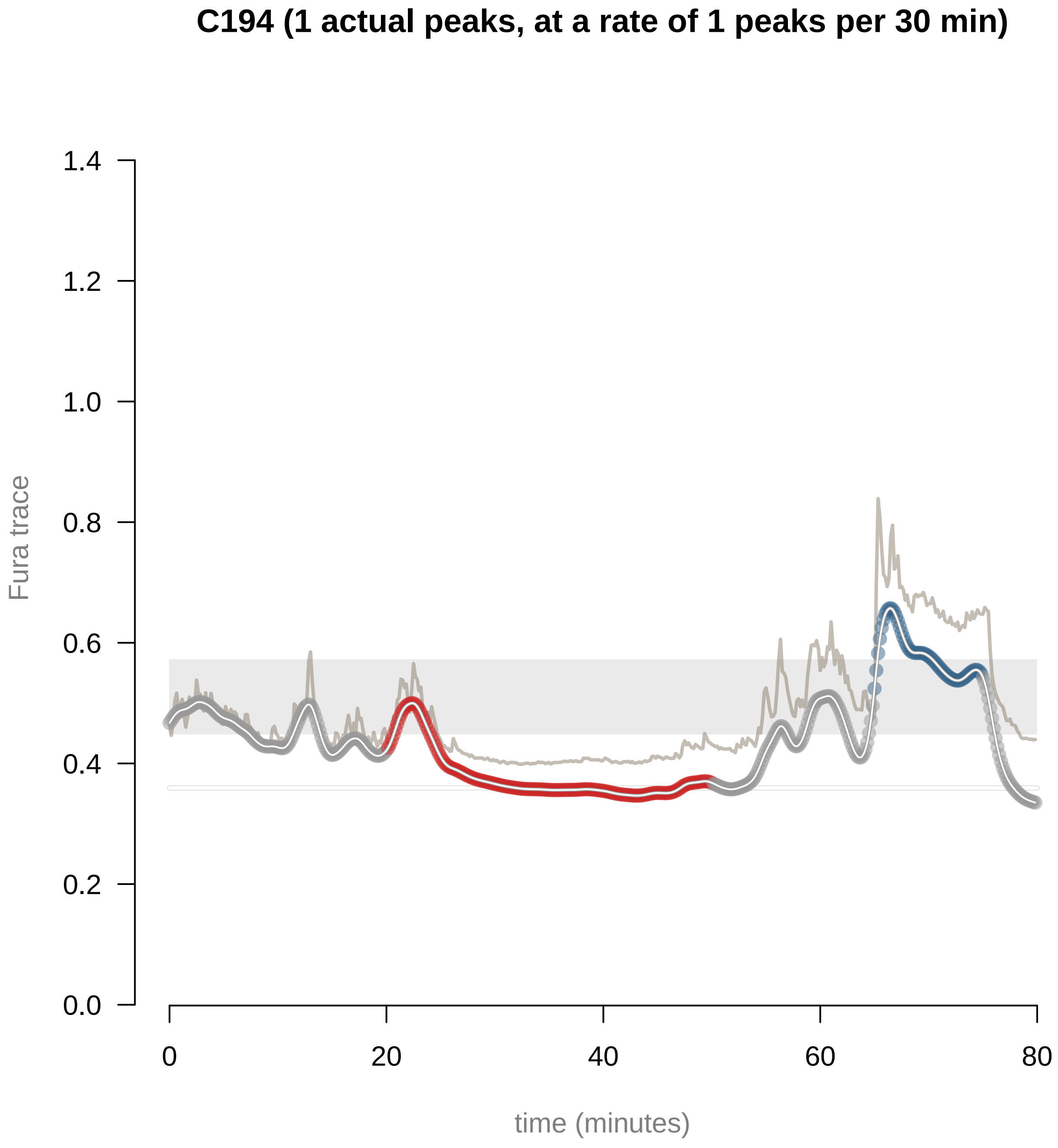

C194 (1 actual peaks, at a rate of 1 peaks per 30 min)
Fura trace
1.4
1.2
1.0
0.8
0.6
0.4
0.2
0.0
0
20
40
60
80
time (minutes)

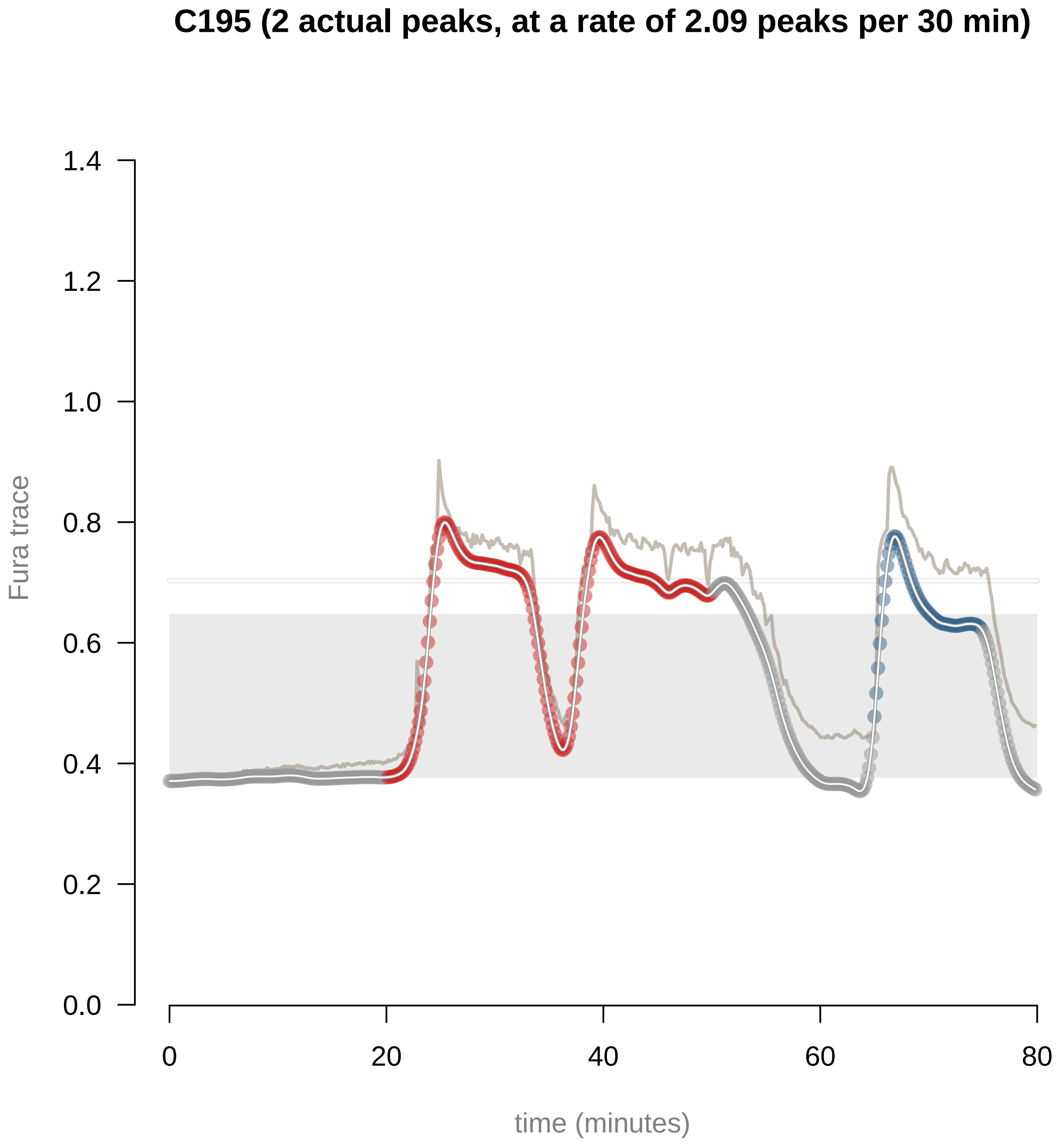

C195 (2 actual peaks, at a rate of 2.09 peaks per 30 min)
Fura trace
0.0
0.2
0.4
0.6
0.8
1.0
1.2
1.4
0
20
40
60
80
time (minutes)

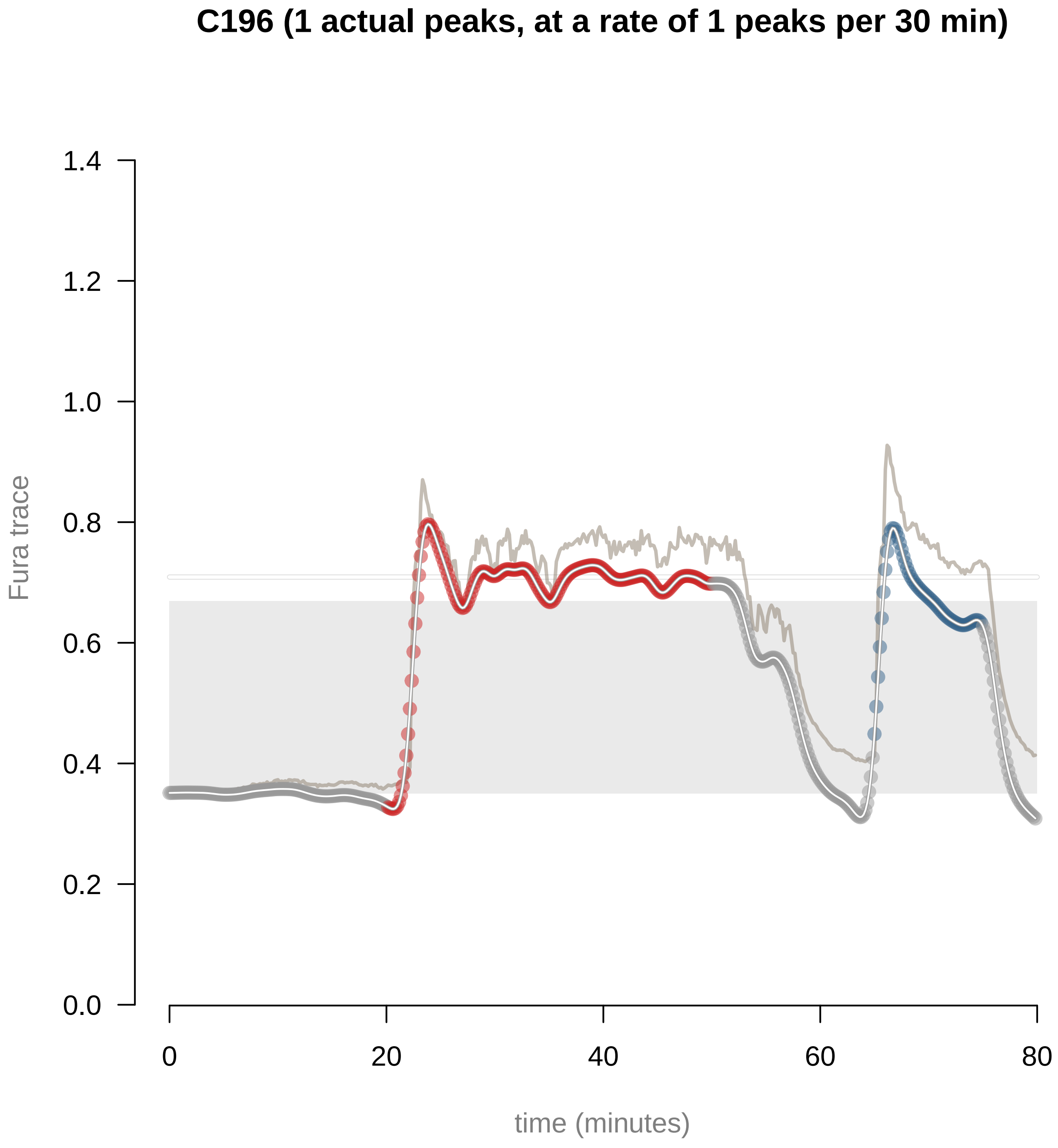
C196 (1 actual peaks, at a rate of 1 peaks per 30 min)
Fura trace
1.4
1.2
1.0
0.8
0.6
0.4
0.2
0.0
0
20
40
60
80
time (minutes)

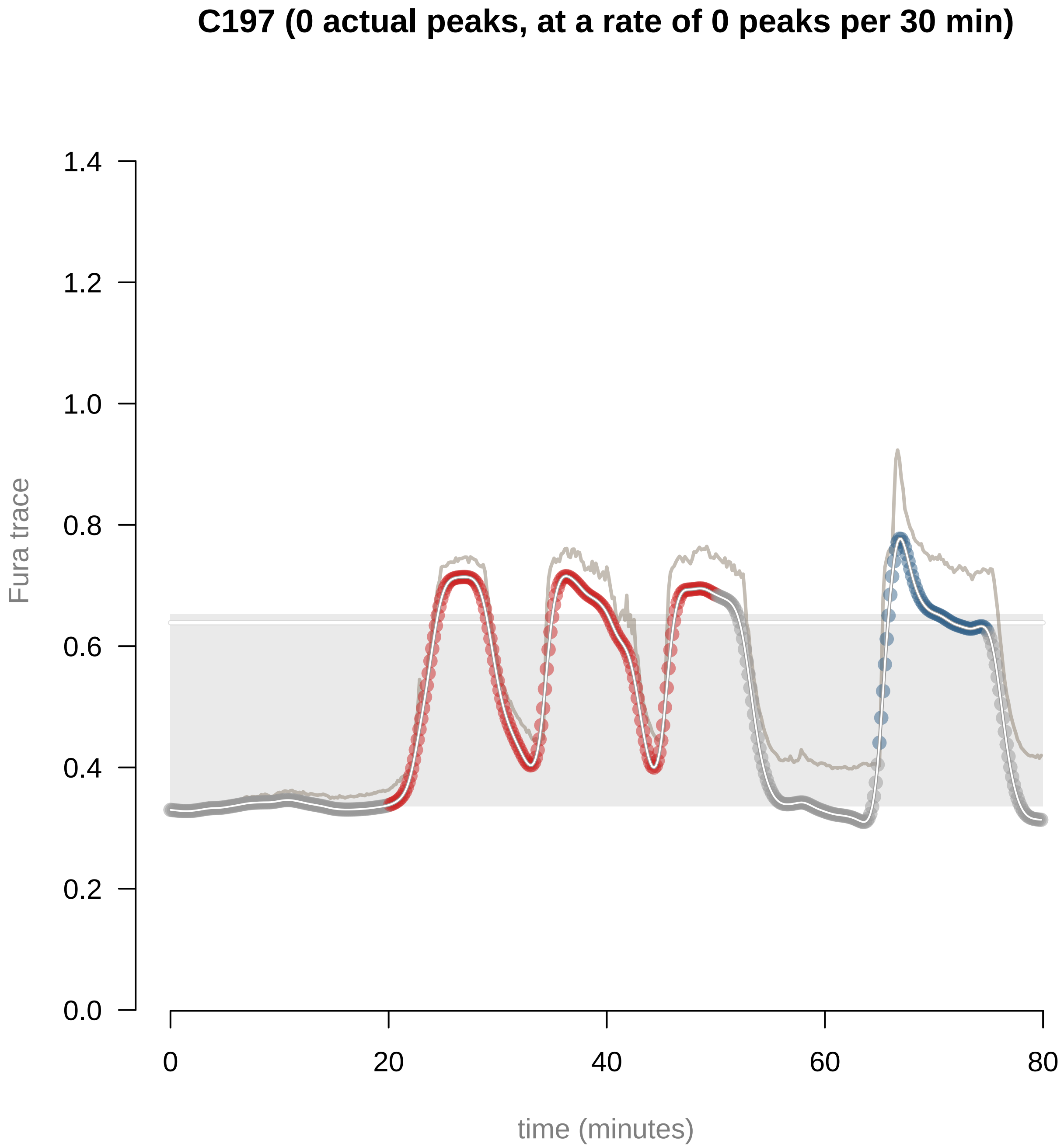

C197 (0 actual peaks, at a rate of 0 peaks per 30 min)
Fura trace
time (minutes)
0.0
0.2
0.4
0.6
0.8
1.0
1.2
1.4
0
20
40
60
80

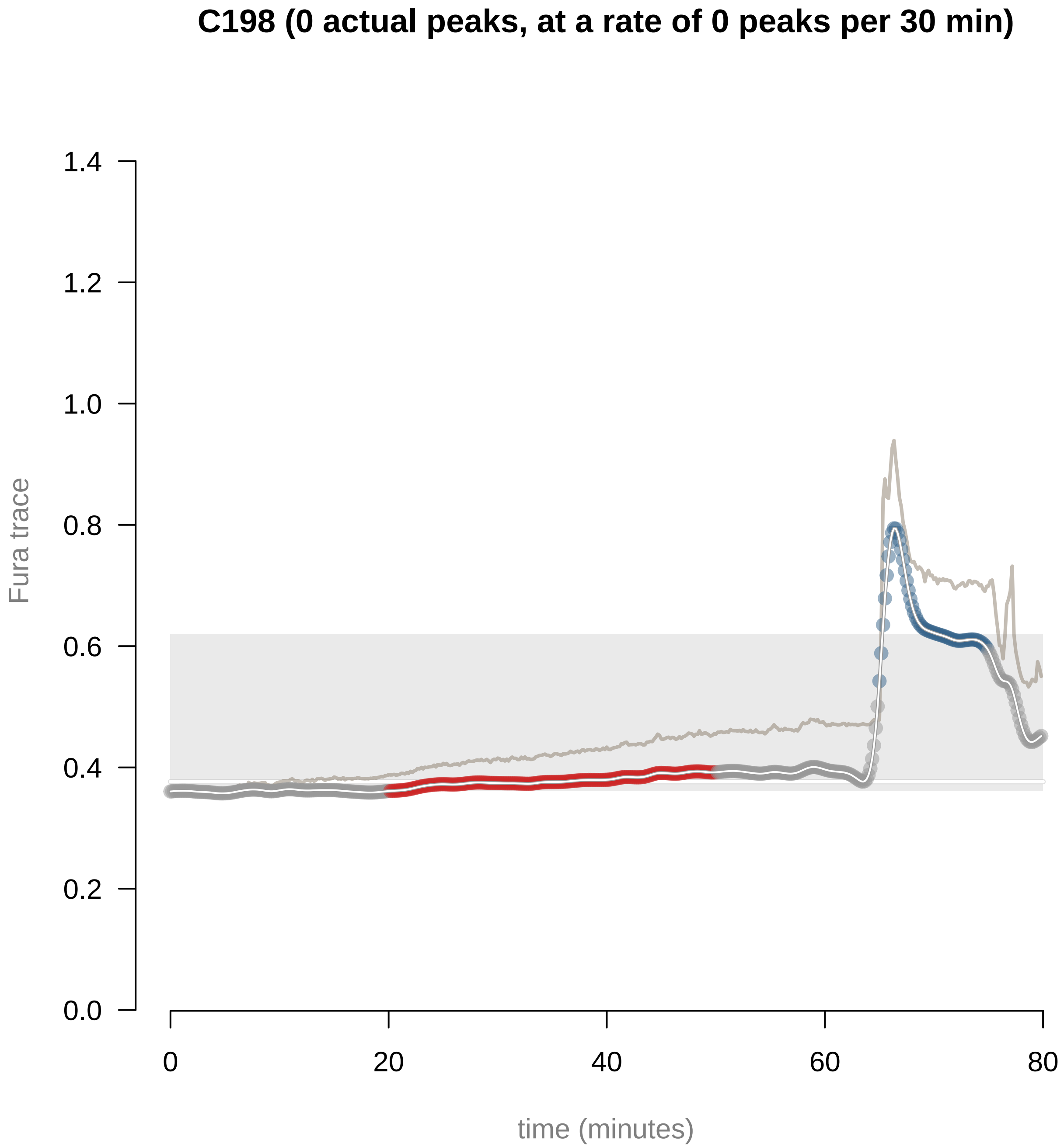

C198 (0 actual peaks, at a rate of 0 peaks per 30 min)
Fura trace
1.4
1.2
1.0
0.8
0.6
0.4
0.2
0.0
0
20
40
60
80
time (minutes)

C199 (0 actual peaks, at a rate of 0 peaks per 30 min)

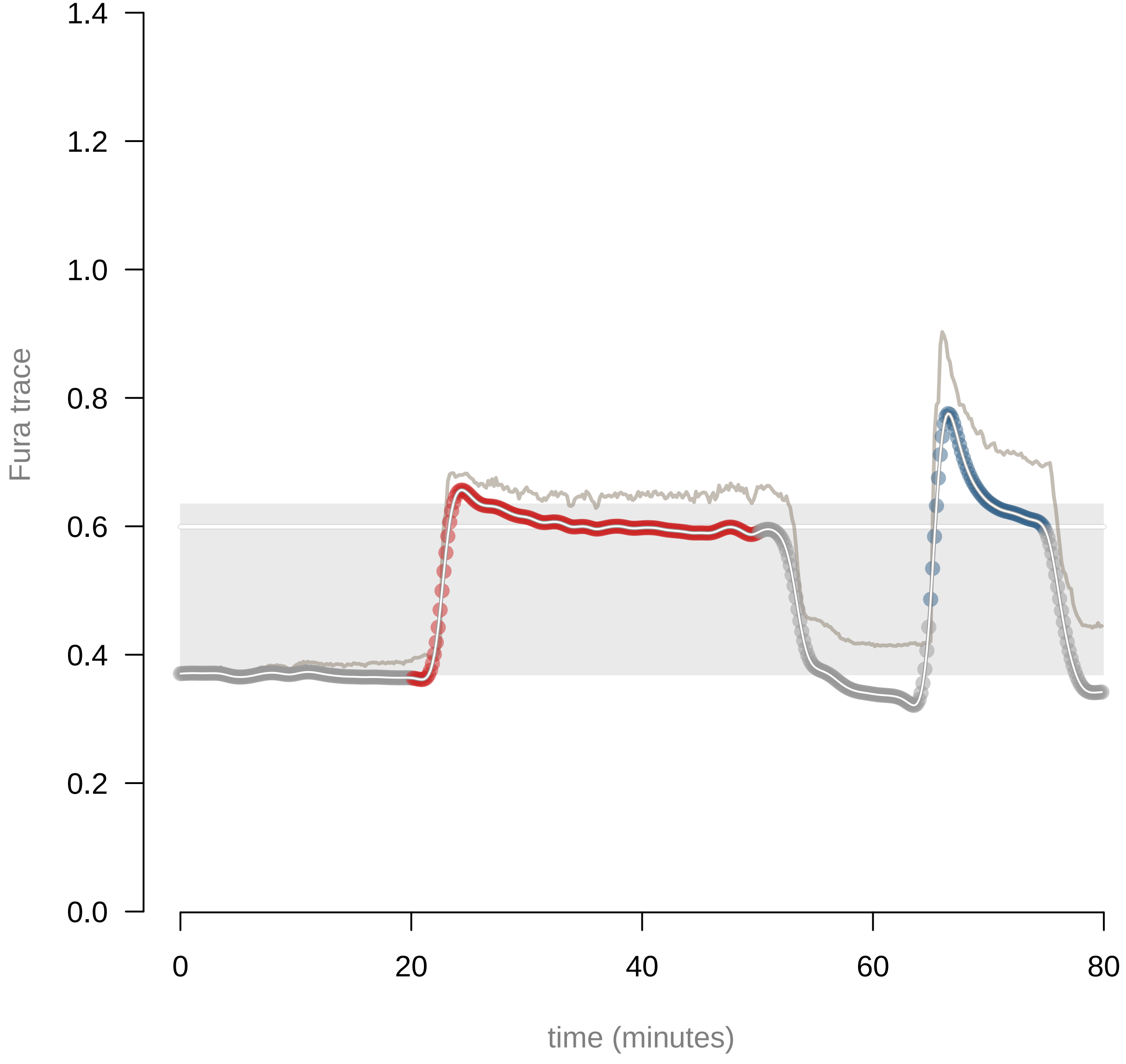

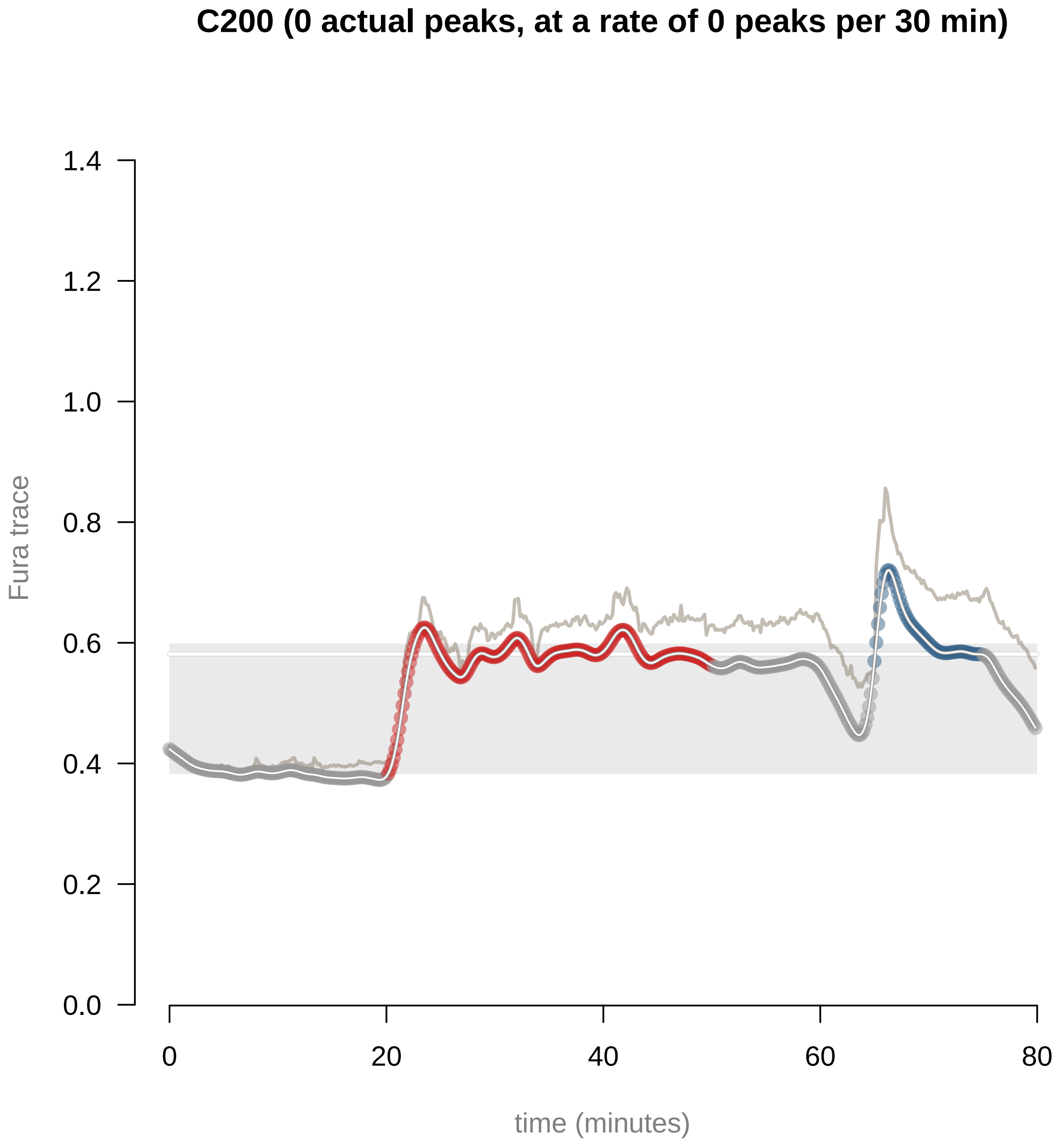

C200 (0 actual peaks, at a rate of 0 peaks per 30 min)
Fura trace
time (minutes)
0.0
0.2
0.4
0.6
0.8
1.0
1.2
1.4
0
20
40
60
80

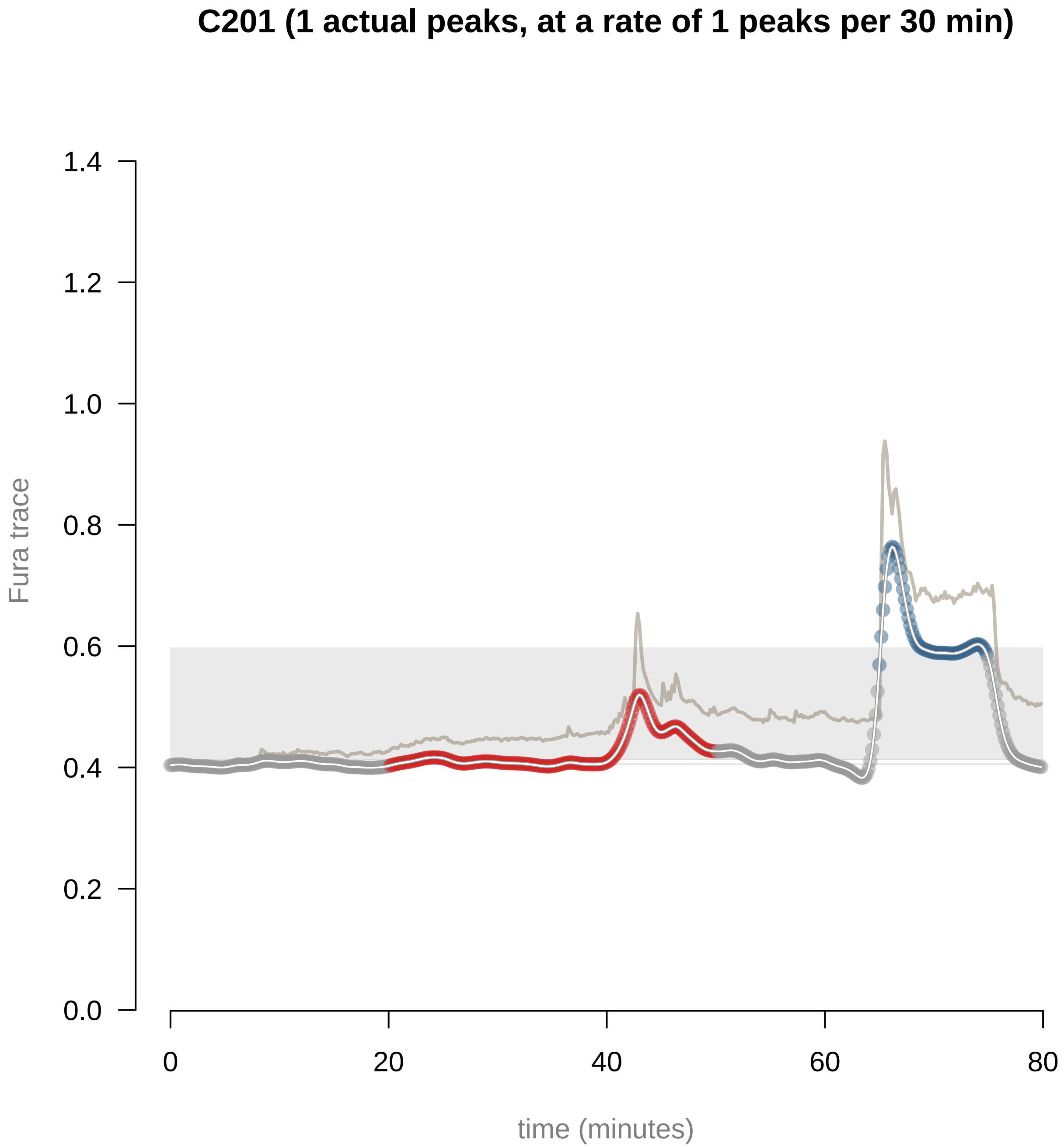
C201 (1 actual peaks, at a rate of 1 peaks per 30 min)
Fura trace
1.4
1.2
1.0
0.8
0.6
0.4
0.2
0.0
0
20
40
60
80
time (minutes)

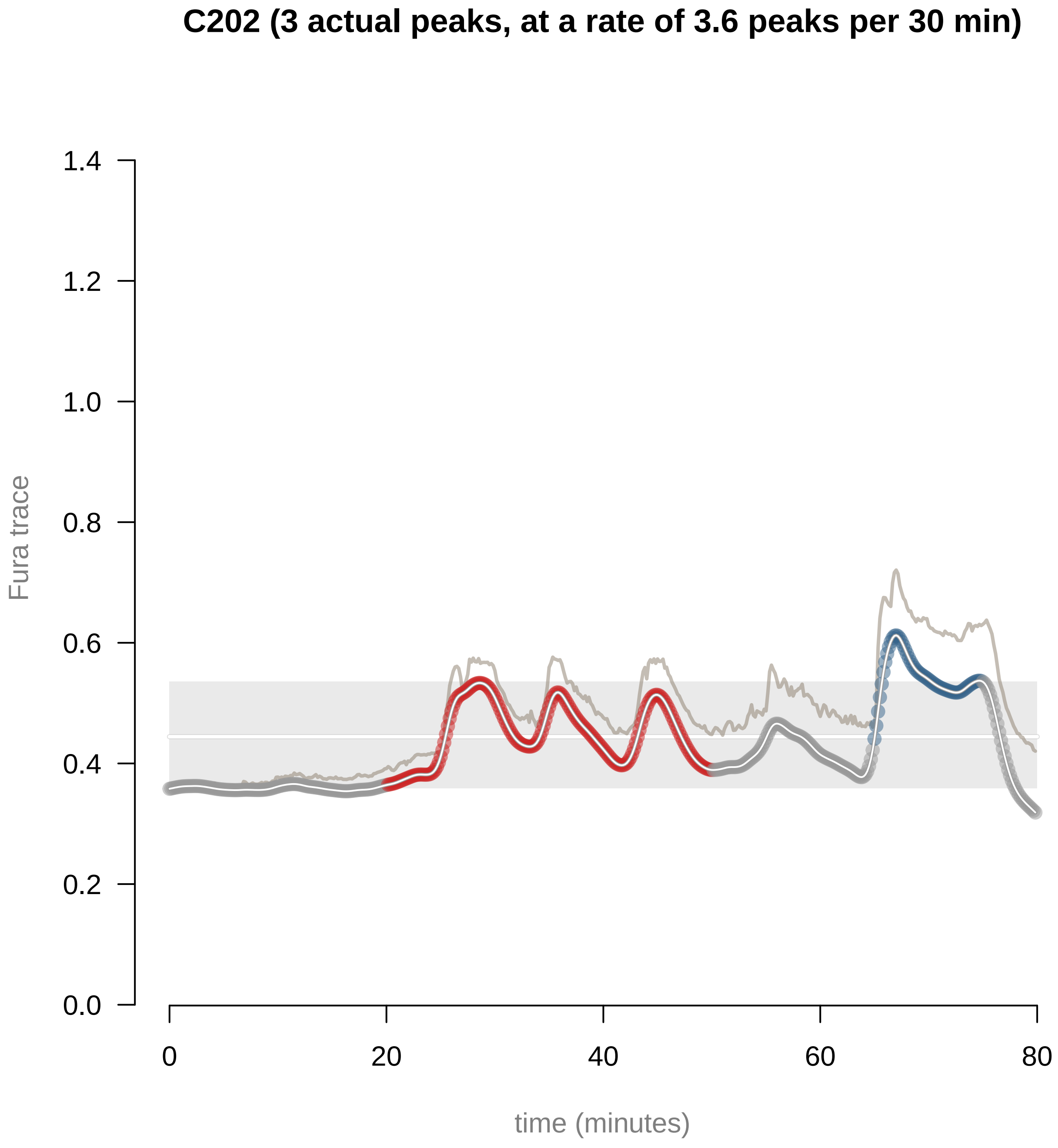
C202 (3 actual peaks, at a rate of 3.6 peaks per 30 min)
Fura trace
time (minutes)
0.0
0.2
0.4
0.6
0.8
1.0
1.2
1.4
0
20
40
60
80

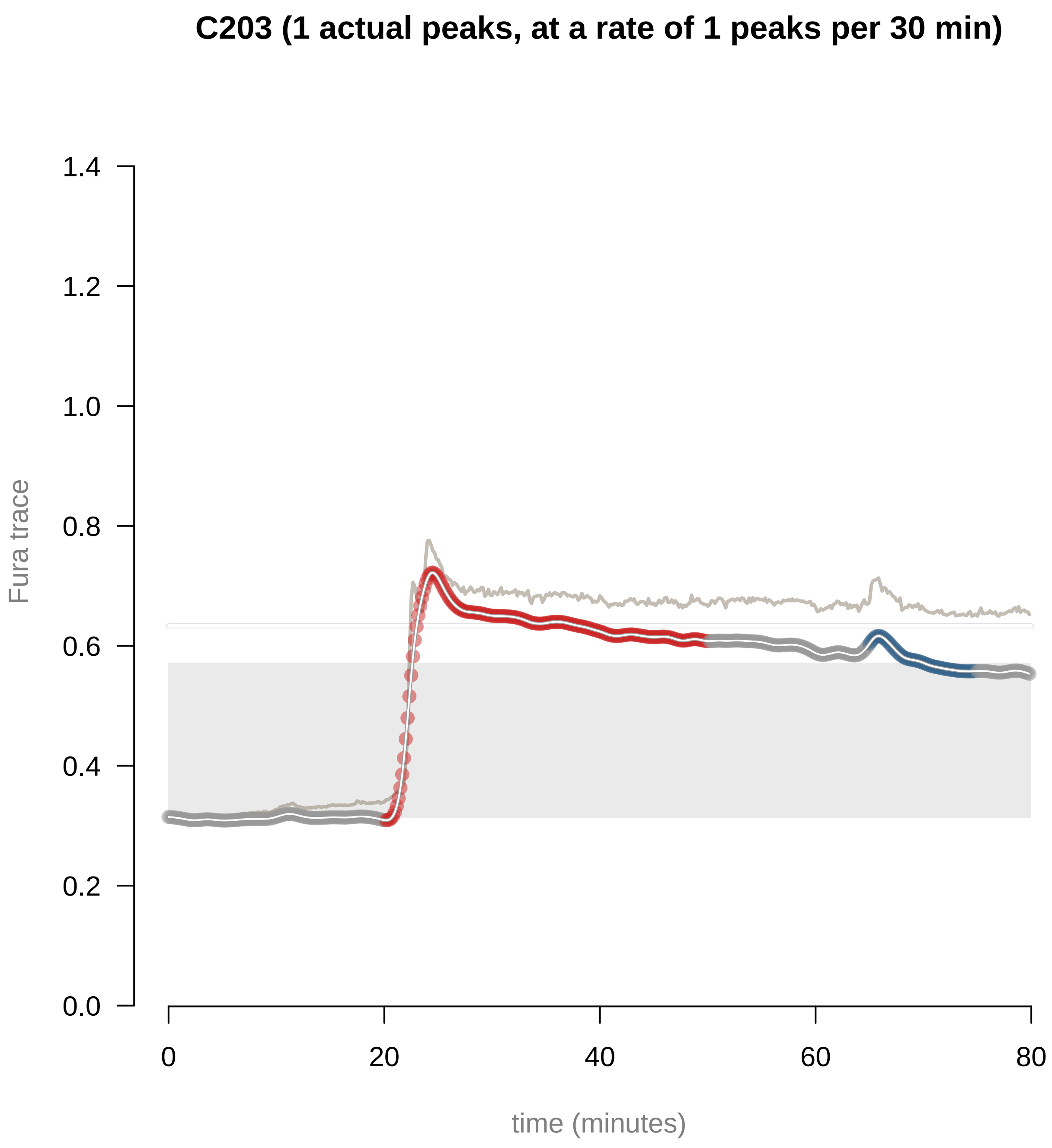
C203 (1 actual peaks, at a rate of 1 peaks per 30 min)
Fura trace
time (minutes)
0.0
0.2
0.4
0.6
0.8
1.0
1.2
1.4
0
20
40
60
80

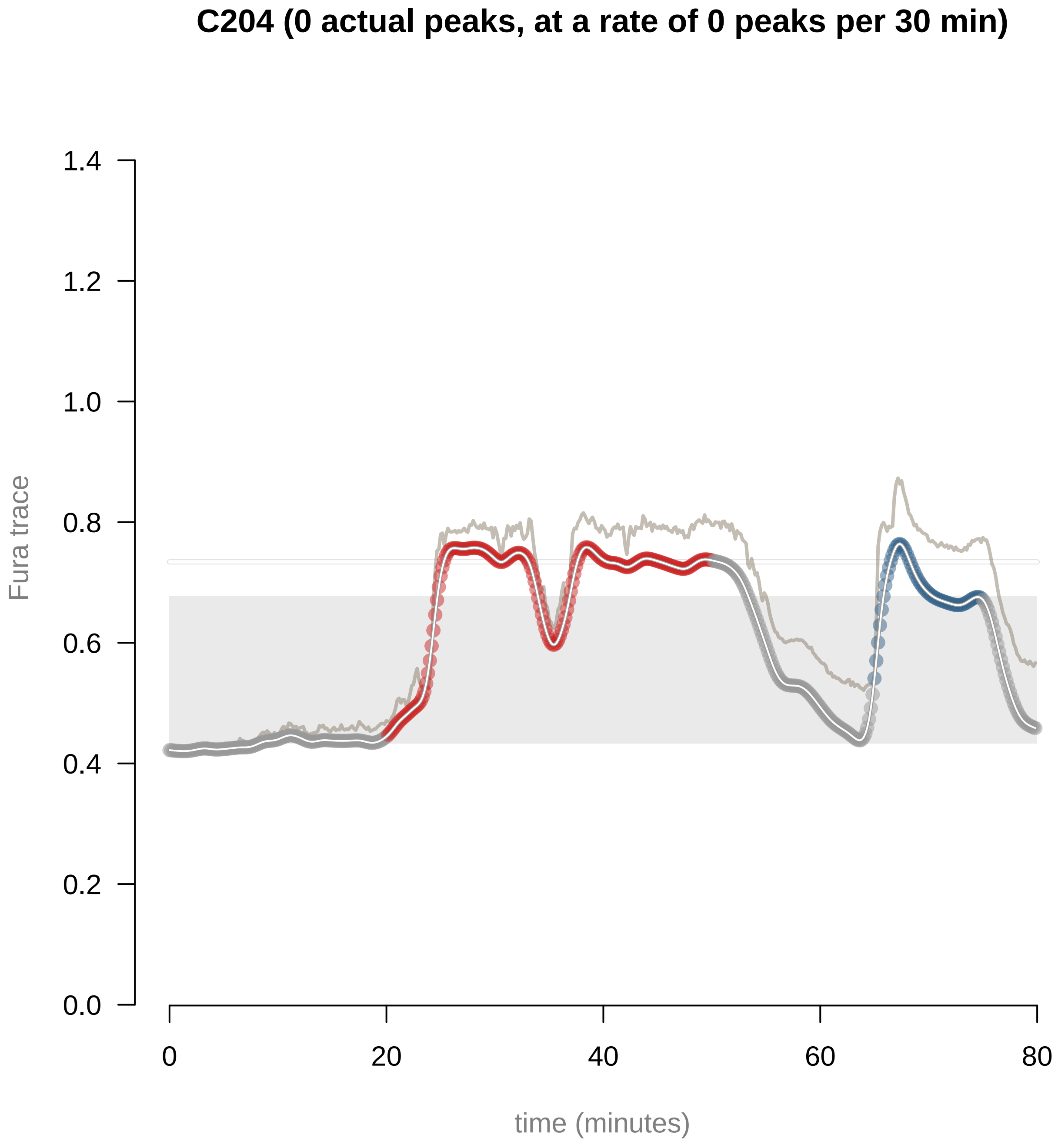

C204 (0 actual peaks, at a rate of 0 peaks per 30 min)
Fura trace
0.0
0.2
0.4
0.6
0.8
1.0
1.2
1.4
0
20
40
60
80
time (minutes)

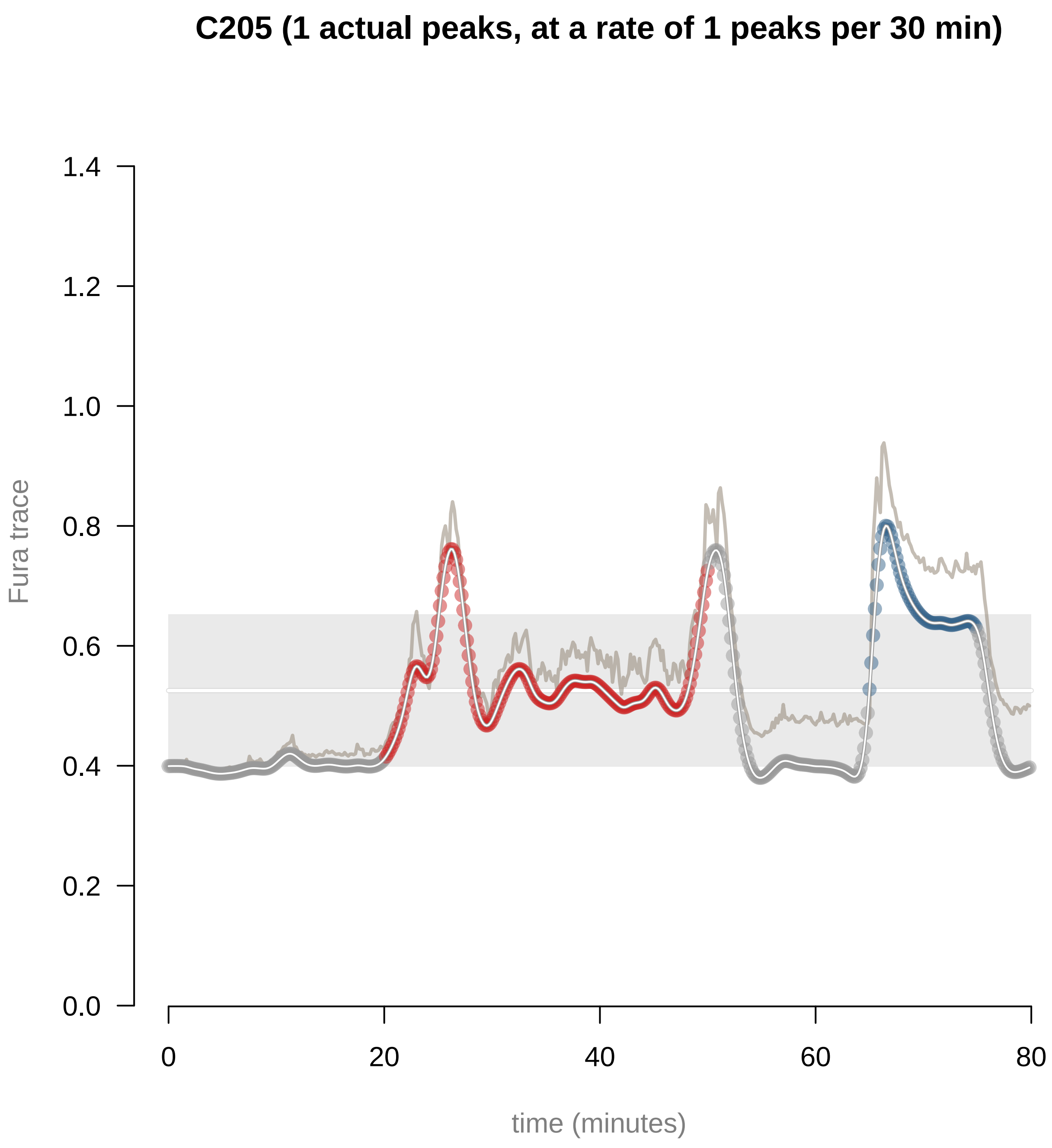
C205 (1 actual peaks, at a rate of 1 peaks per 30 min)
Fura trace
time (minutes)
0.0
0.2
0.4
0.6
0.8
1.0
1.2
1.4
0
20
40
60
80

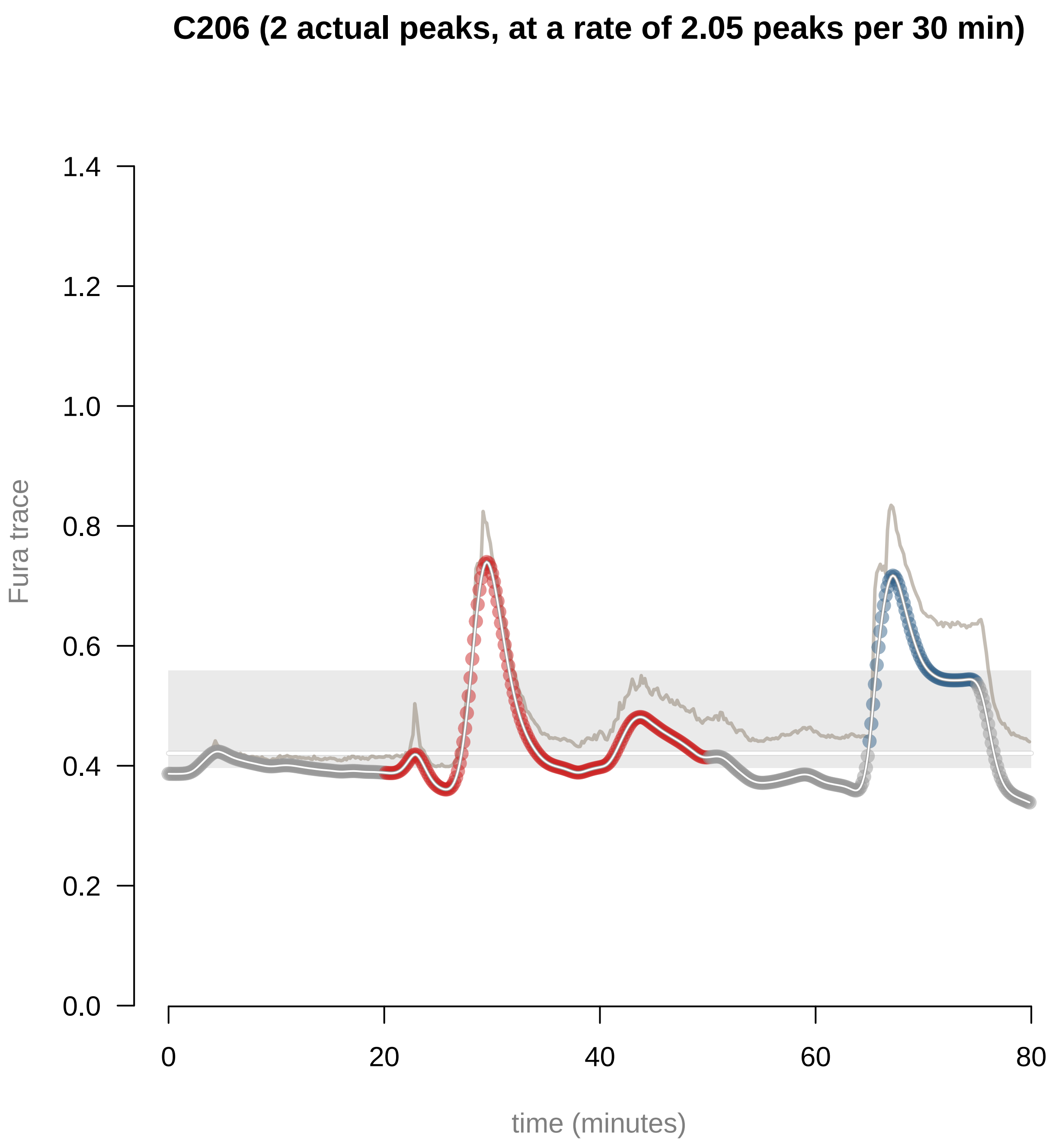

C206 (2 actual peaks, at a rate of 2.05 peaks per 30 min)
Fura trace
0.0
0.2
0.4
0.6
0.8
1.0
1.2
1.4
0
20
40
60
80
time (minutes)

C207 (0 actual peaks, at a rate of 0 peaks per 30 min)

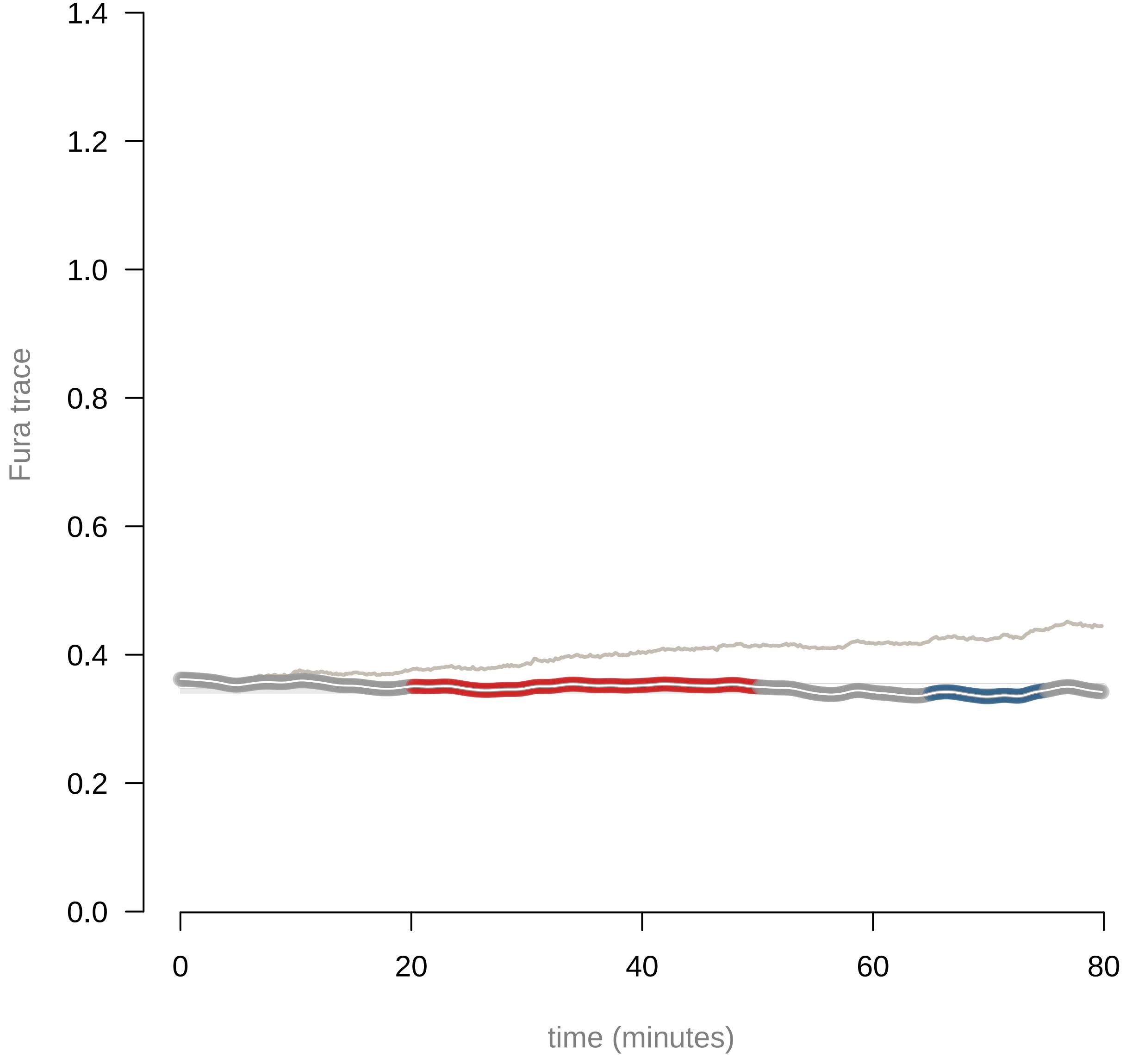

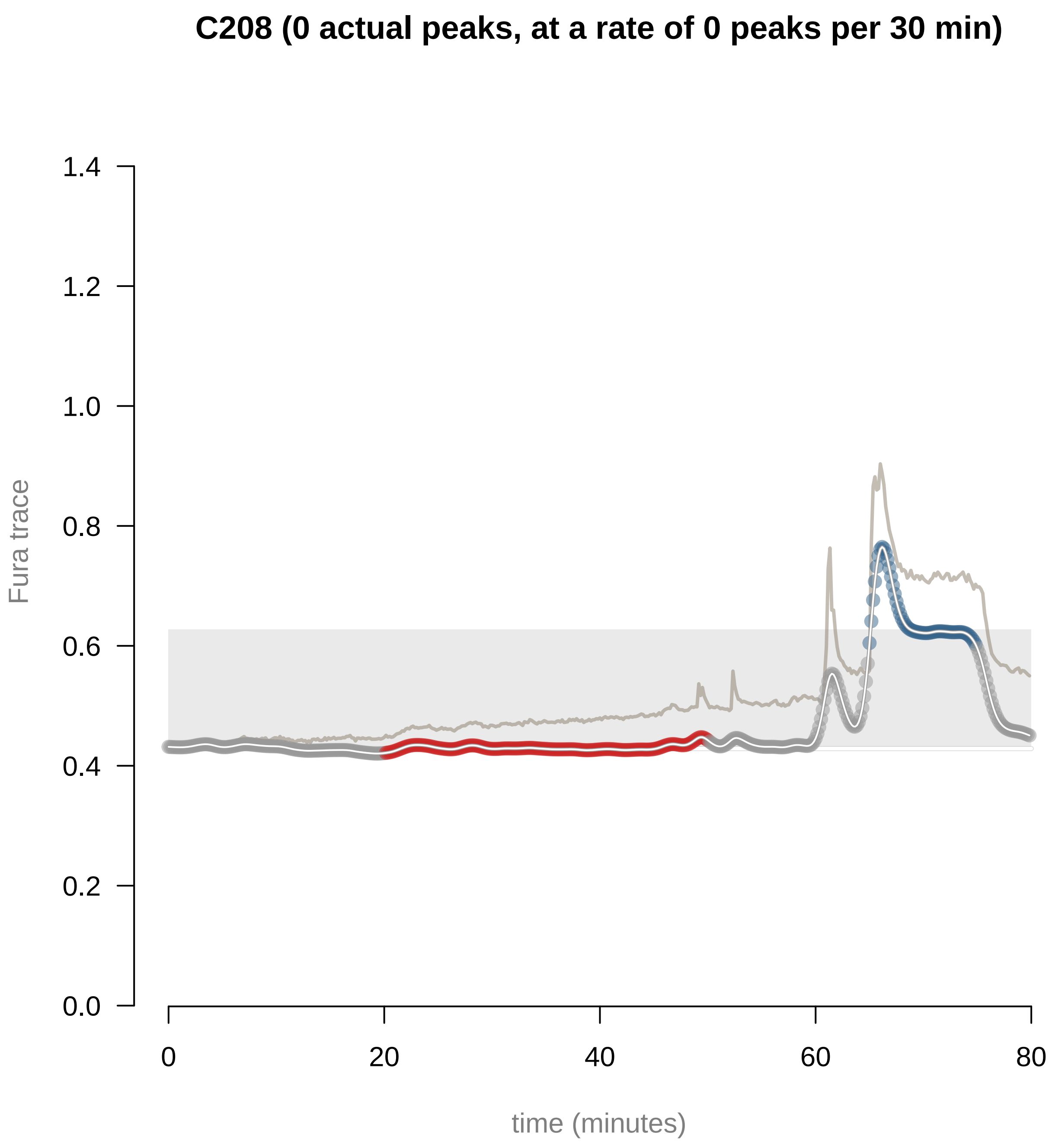
C208 (0 actual peaks, at a rate of 0 peaks per 30 min)
Fura trace
time (minutes)
0.0
0.2
0.4
0.6
0.8
1.0
1.2
1.4
0
20
40
60
80

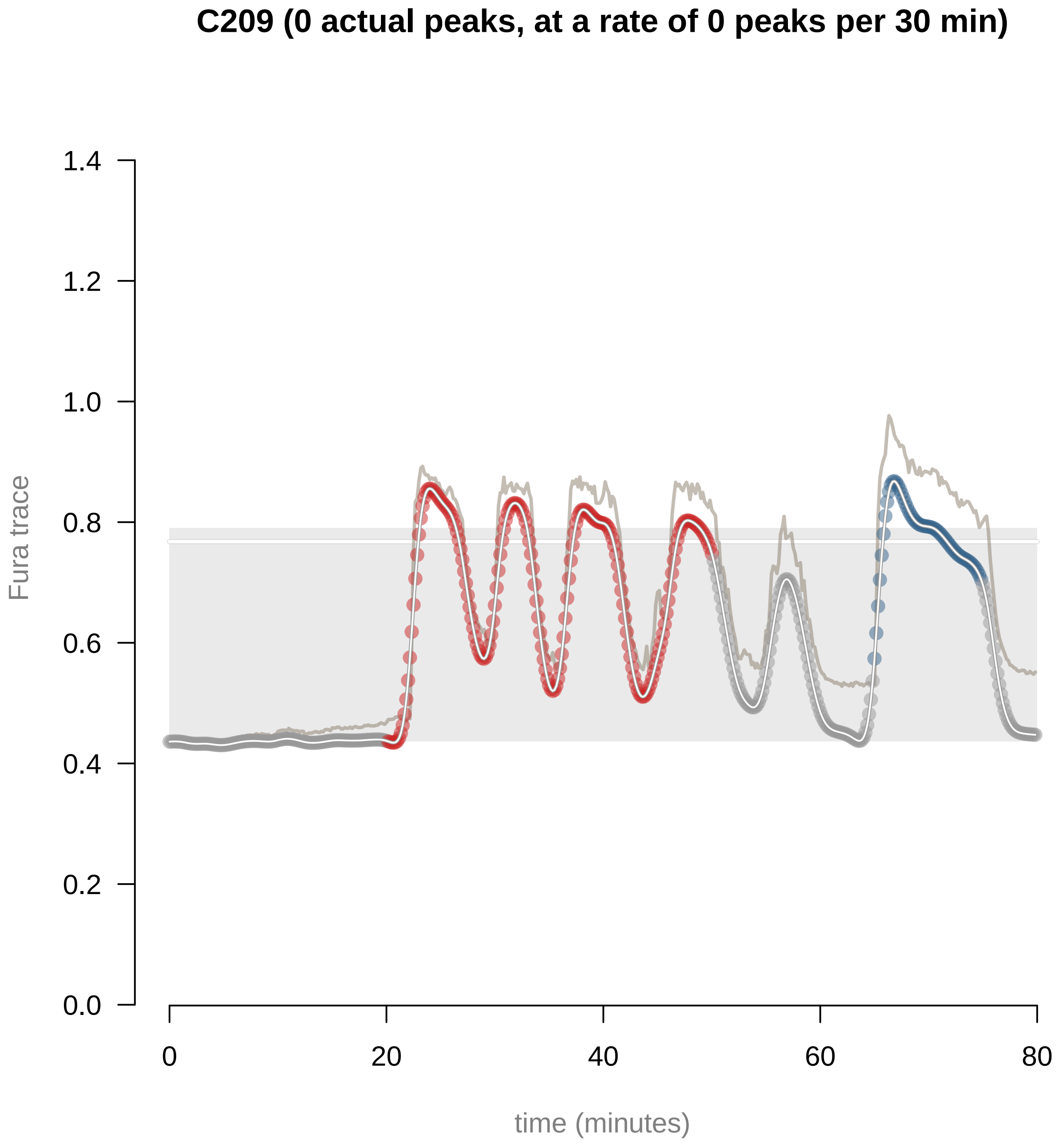

C209 (0 actual peaks, at a rate of 0 peaks per 30 min)
Fura trace
1.4
1.2
1.0
0.8
0.6
0.4
0.2
0.0
0
20
40
60
80
time (minutes)

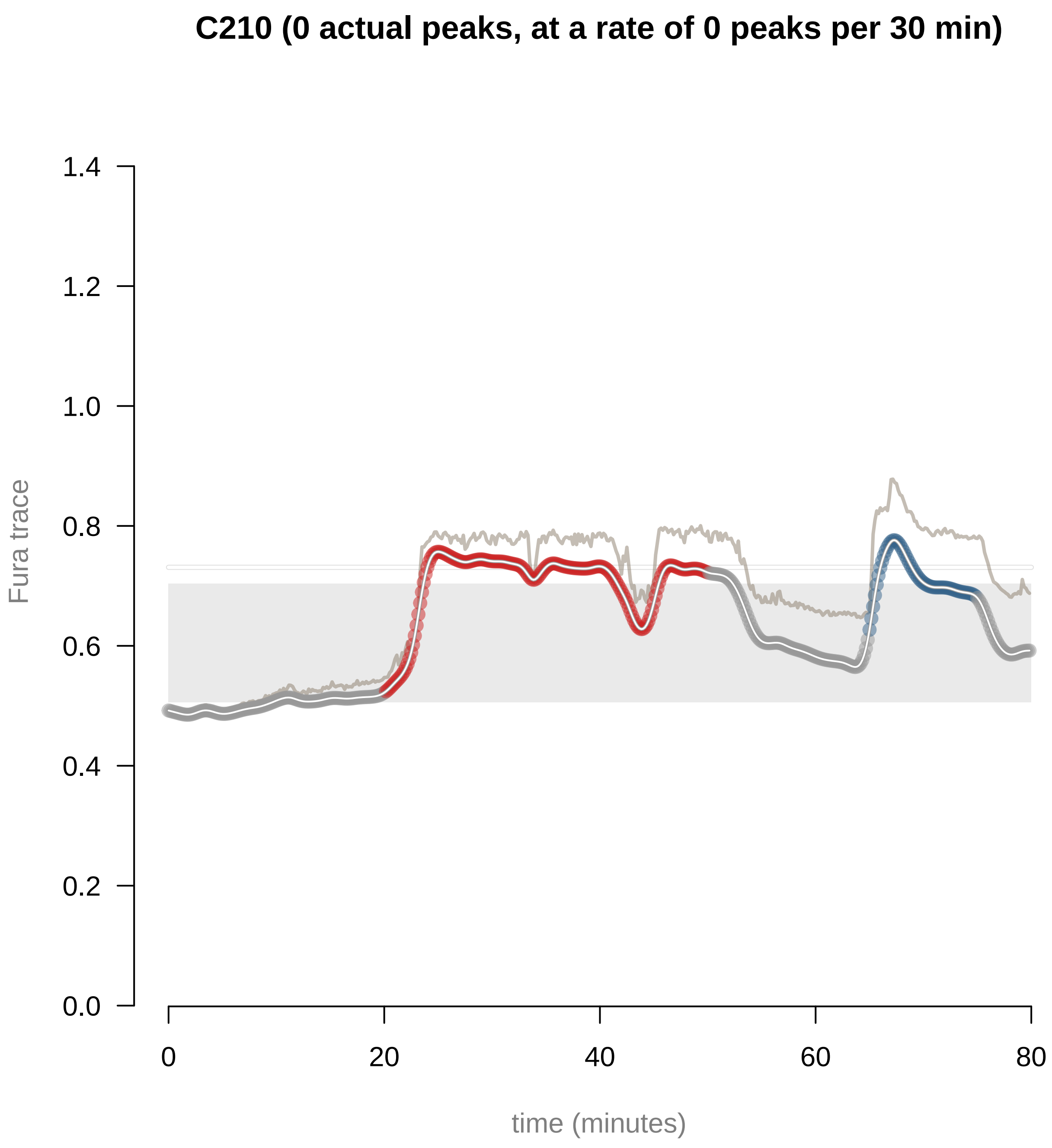

C210 (0 actual peaks, at a rate of 0 peaks per 30 min)
Fura trace
0.0
0.2
0.4
0.6
0.8
1.0
1.2
1.4
0
20
40
60
80
time (minutes)

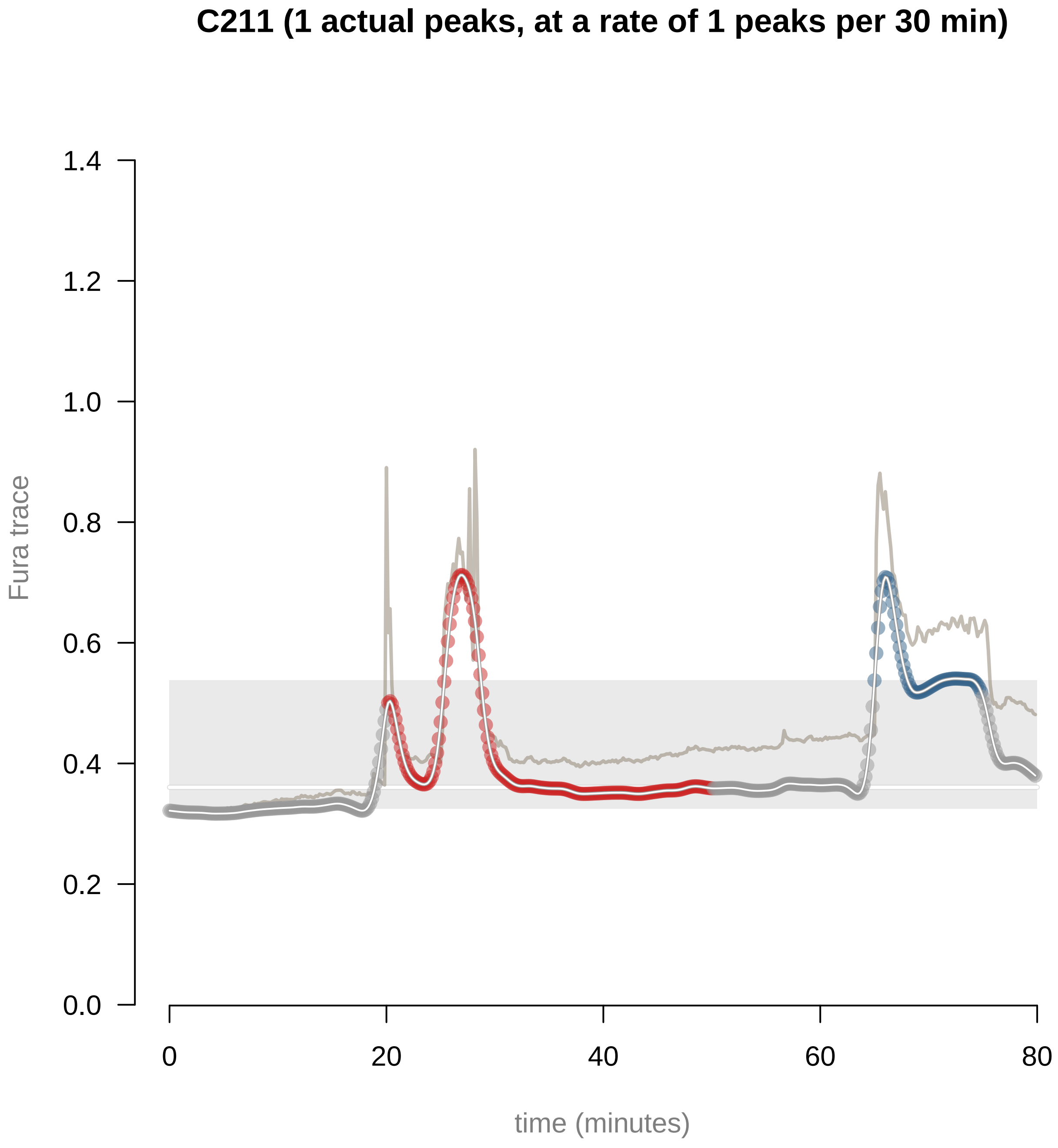

C211 (1 actual peaks, at a rate of 1 peaks per 30 min)
Fura trace
0.0
0.2
0.4
0.6
0.8
1.0
1.2
1.4
0
20
40
60
80
time (minutes)

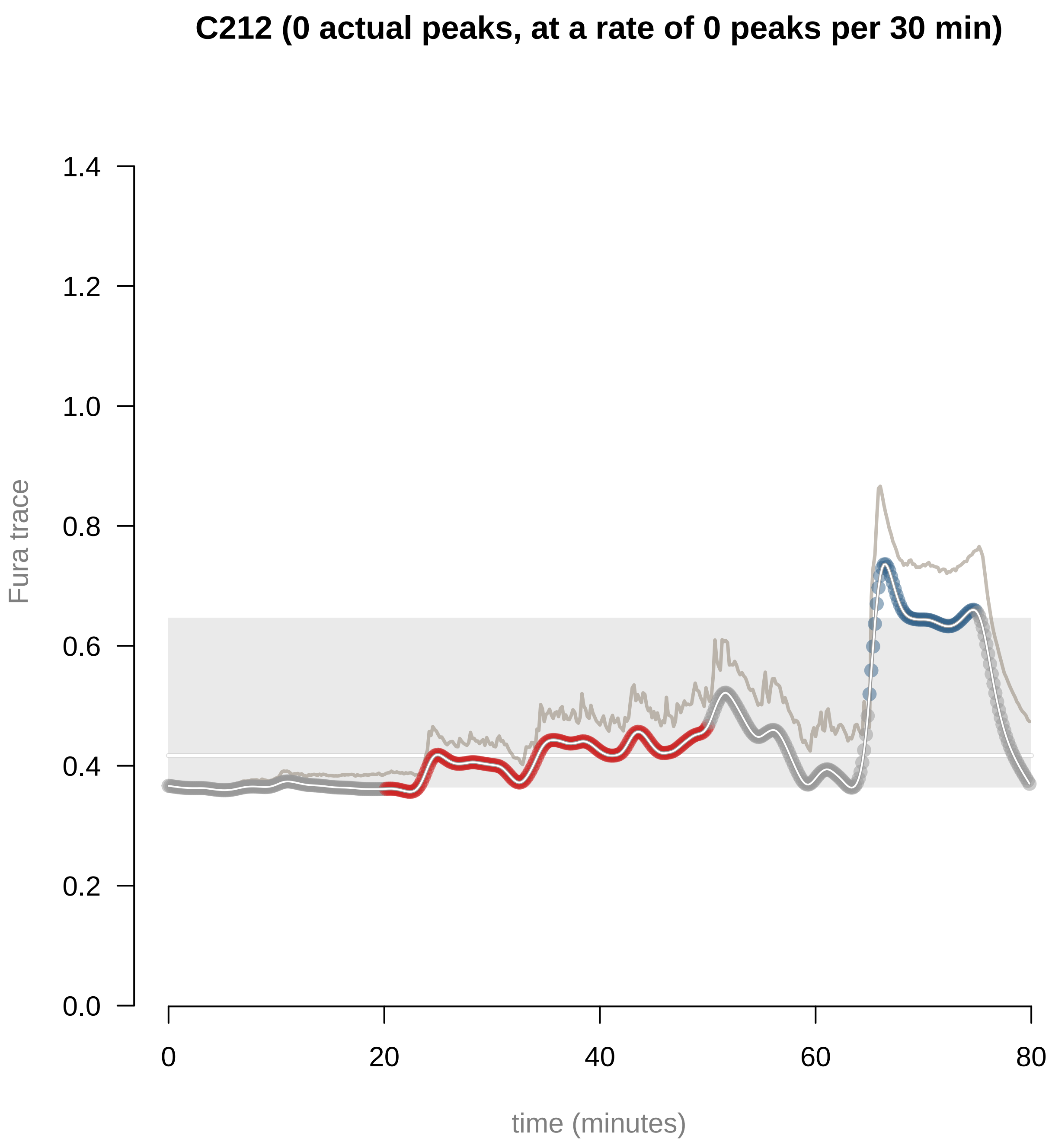

C212 (0 actual peaks, at a rate of 0 peaks per 30 min)
1.4
1.2
1.0
0.8
0.6
0.4
0.2
0.0
Fura trace
0
20
40
60
80
time (minutes)

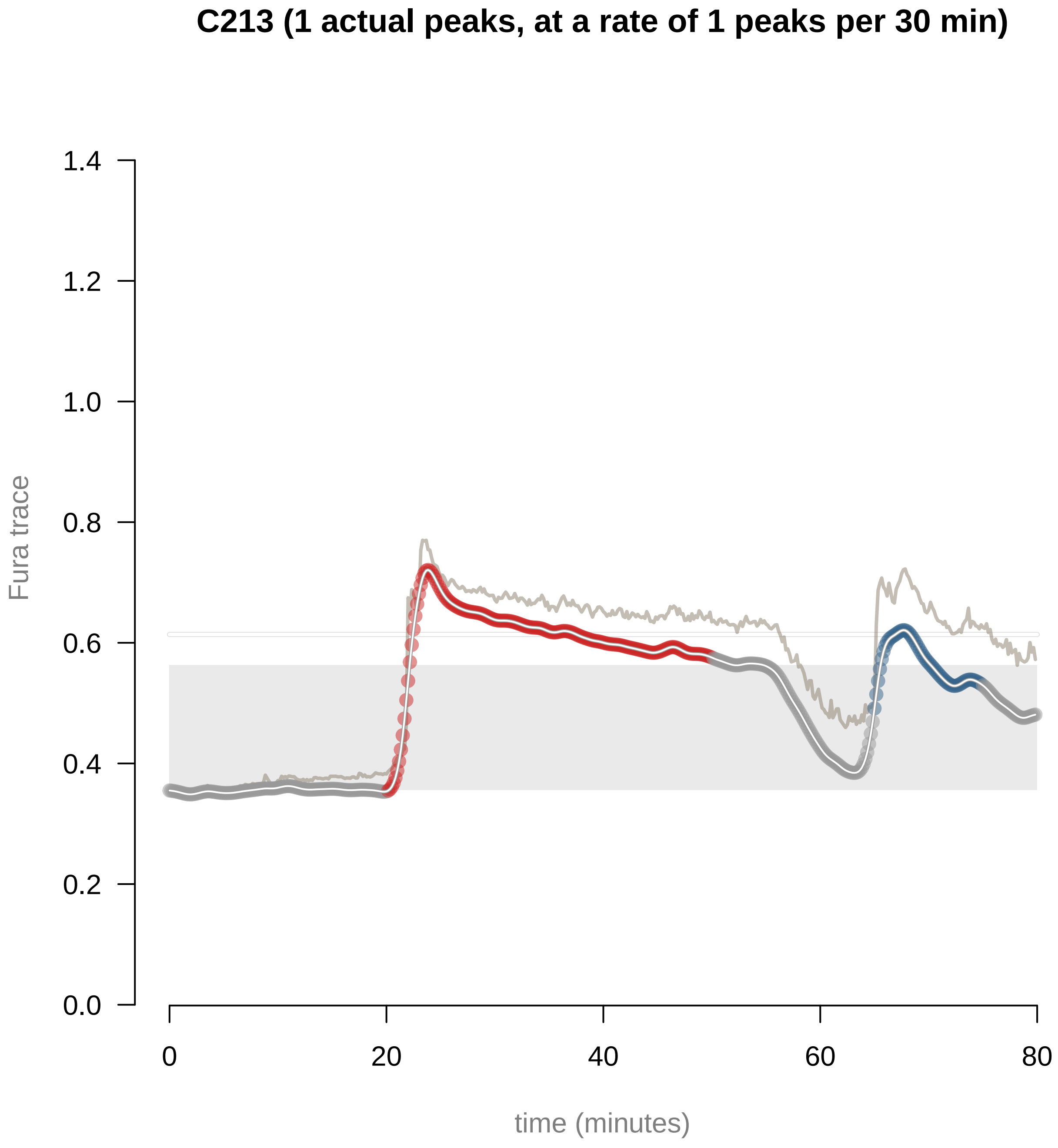

C213 (1 actual peaks, at a rate of 1 peaks per 30 min)
Fura trace
1.4
1.2
1.0
0.8
0.6
0.4
0.2
0.0
0
20
40
60
80
time (minutes)

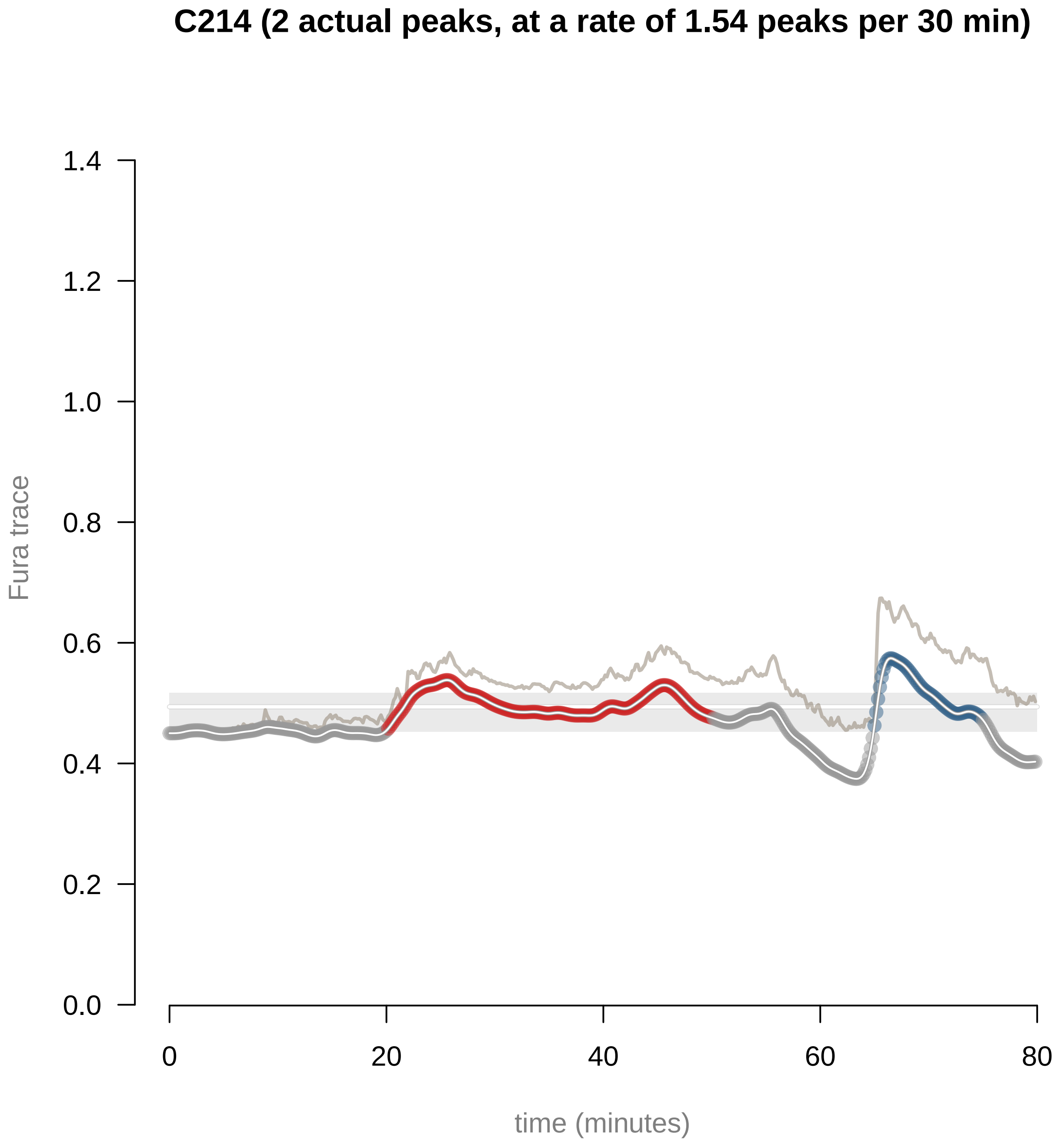

C214 (2 actual peaks, at a rate of 1.54 peaks per 30 min)
Fura trace
time (minutes)
0.0
0.2
0.4
0.6
0.8
1.0
1.2
1.4
0
20
40
60
80

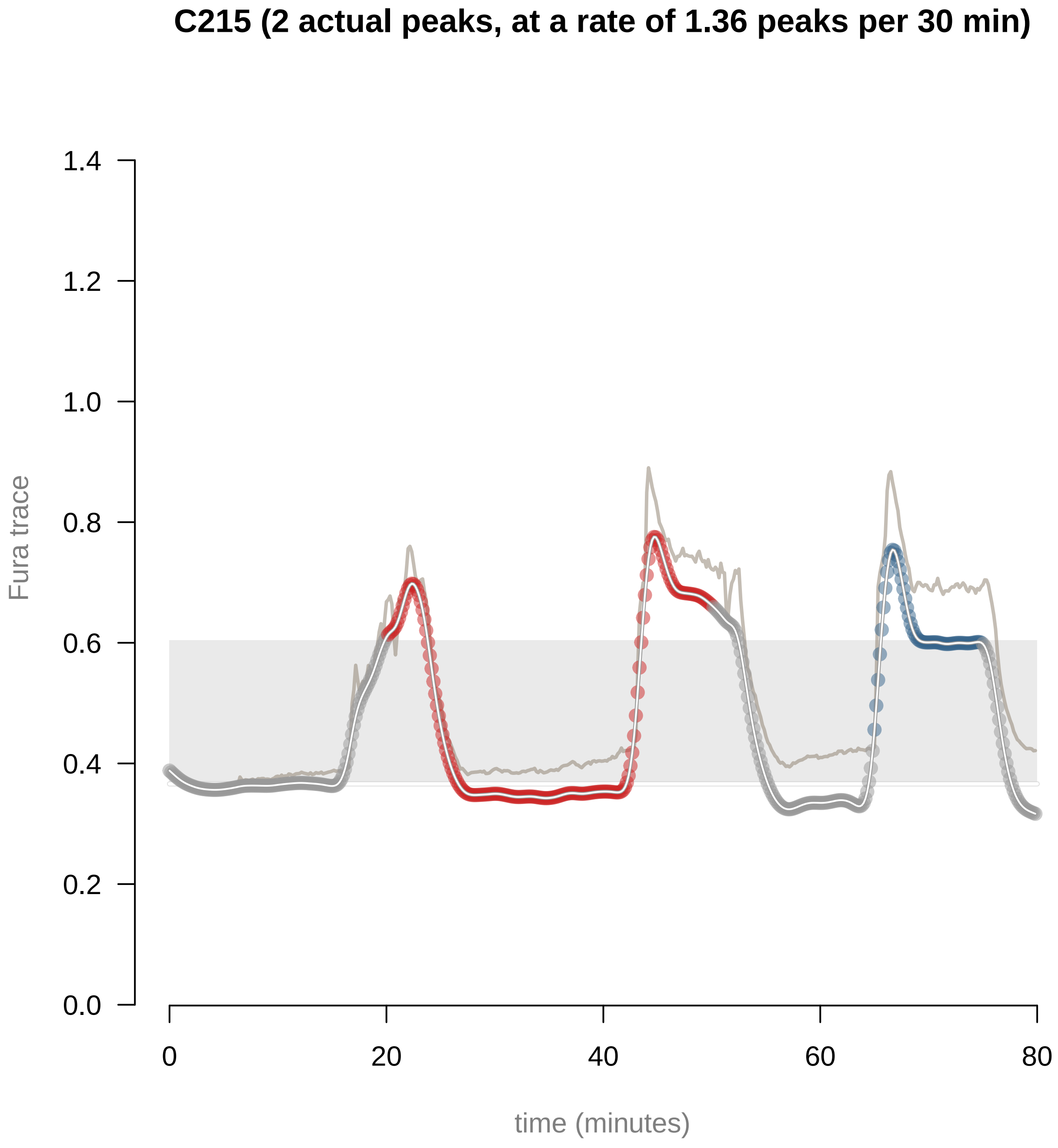
C215 (2 actual peaks, at a rate of 1.36 peaks per 30 min)
Fura trace
1.4
1.2
1.0
0.8
0.6
0.4
0.2
0.0
0
20
40
60
80
time (minutes)

C216 (4 actual peaks, at a rate of 3.83 peaks per 30 min)

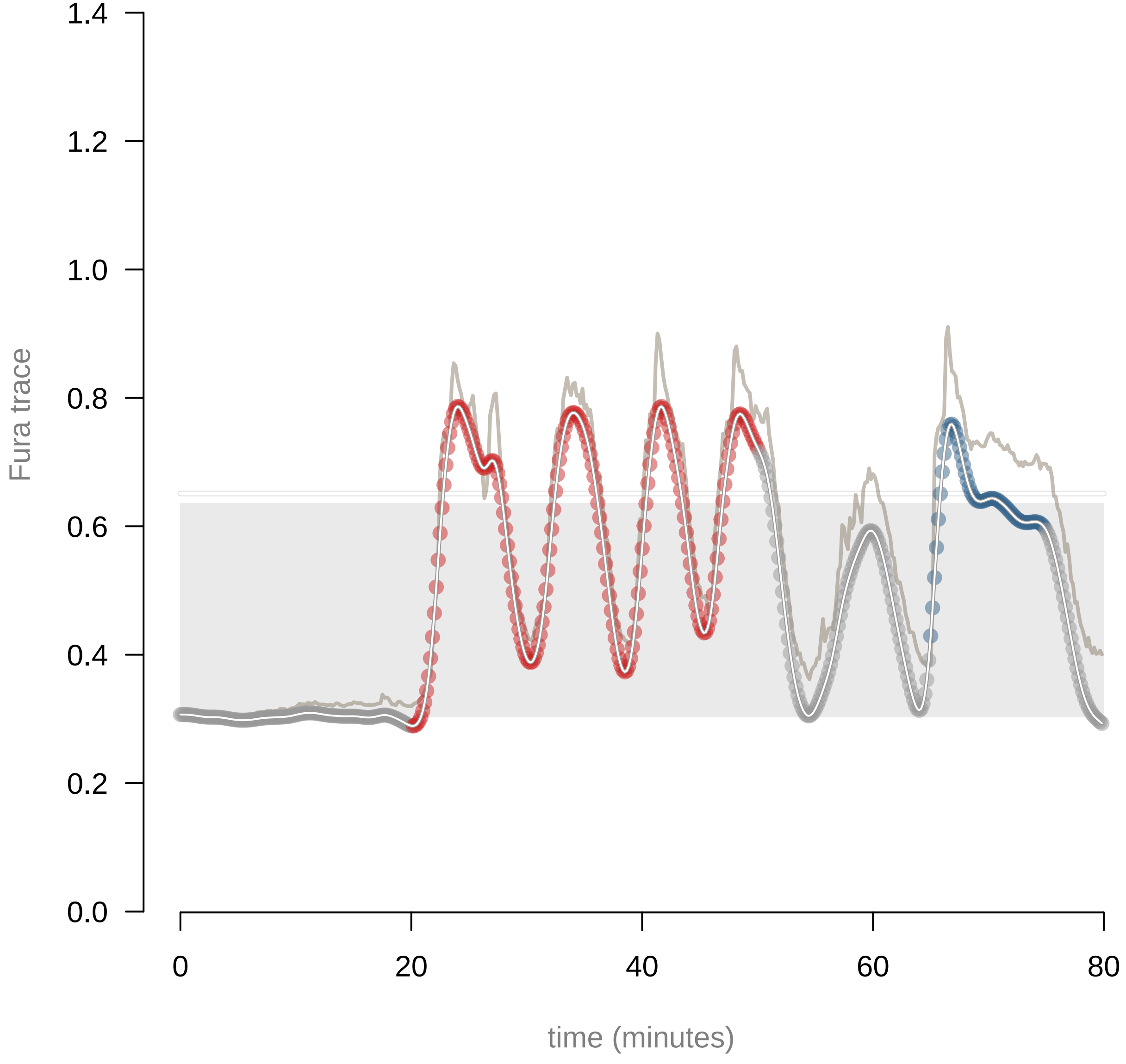

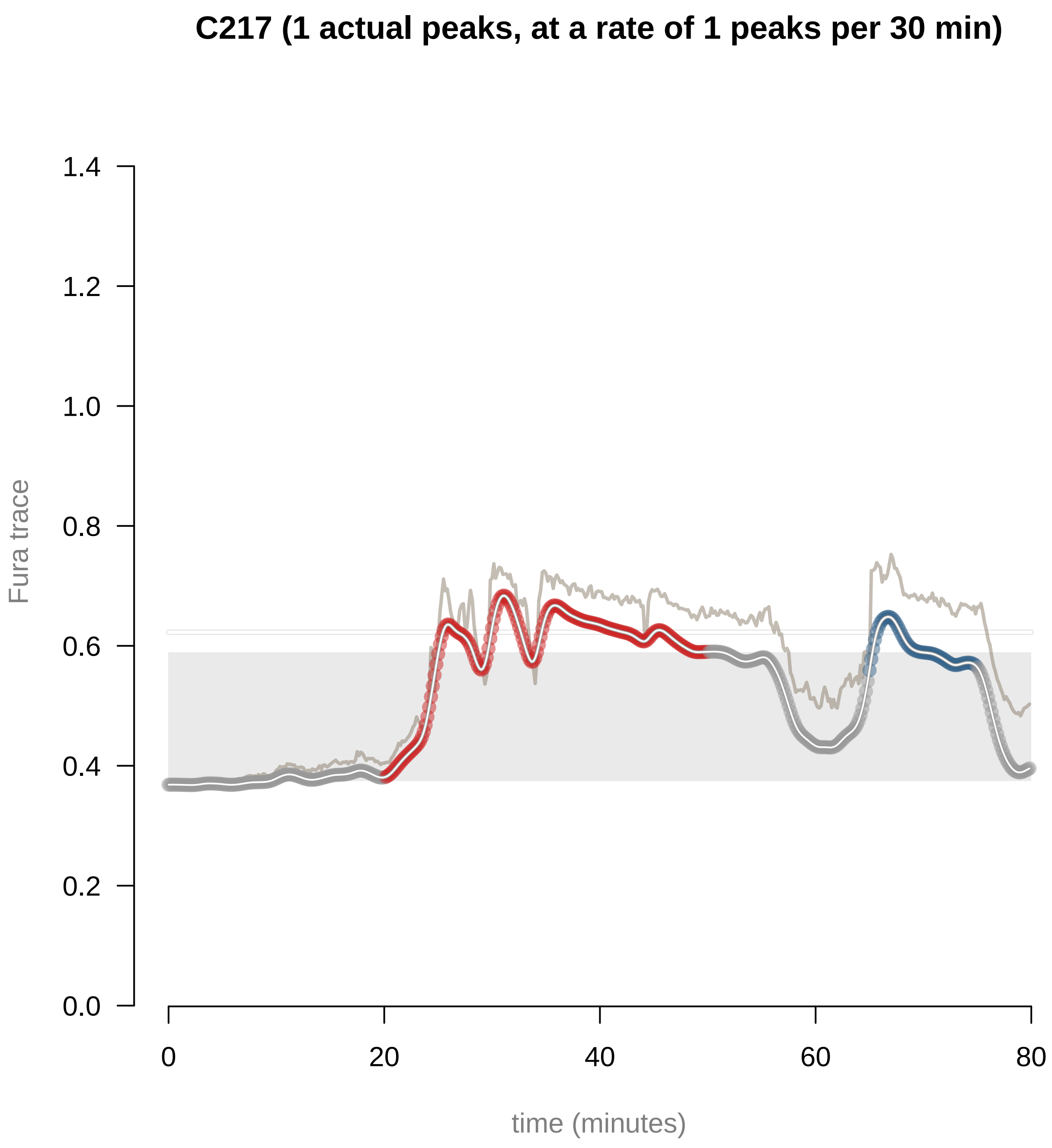

C217 (1 actual peaks, at a rate of 1 peaks per 30 min)
Fura trace
1.4
1.2
1.0
0.8
0.6
0.4
0.2
0.0
0
20
40
60
80
time (minutes)

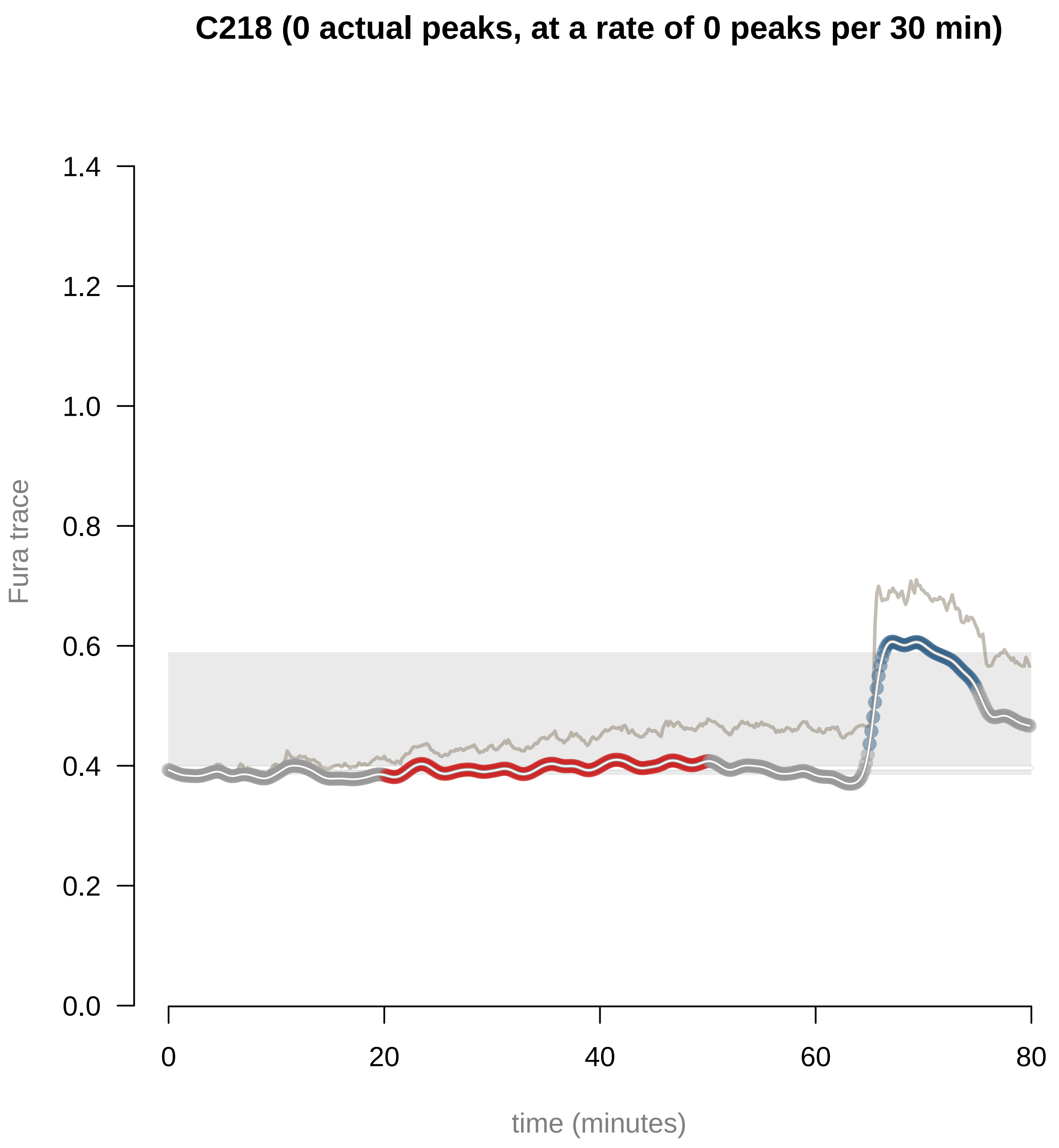

C218 (0 actual peaks, at a rate of 0 peaks per 30 min)
Fura trace
1.4
1.2
1.0
0.8
0.6
0.4
0.2
0.0
0
20
40
60
80
time (minutes)

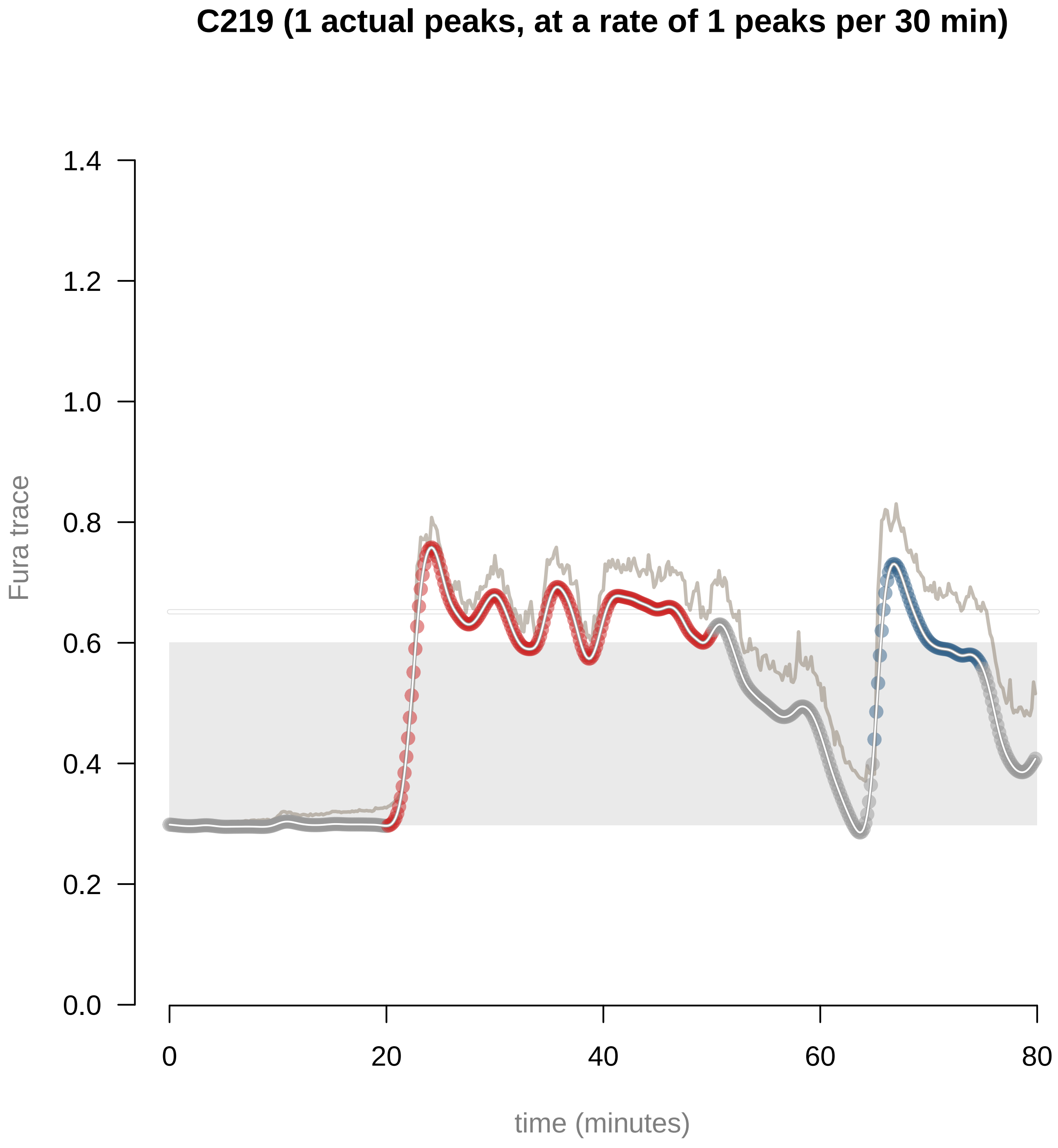
C219 (1 actual peaks, at a rate of 1 peaks per 30 min)
Fura trace
1.4
1.2
1.0
0.8
0.6
0.4
0.2
0.0
0
20
40
60
80
time (minutes)

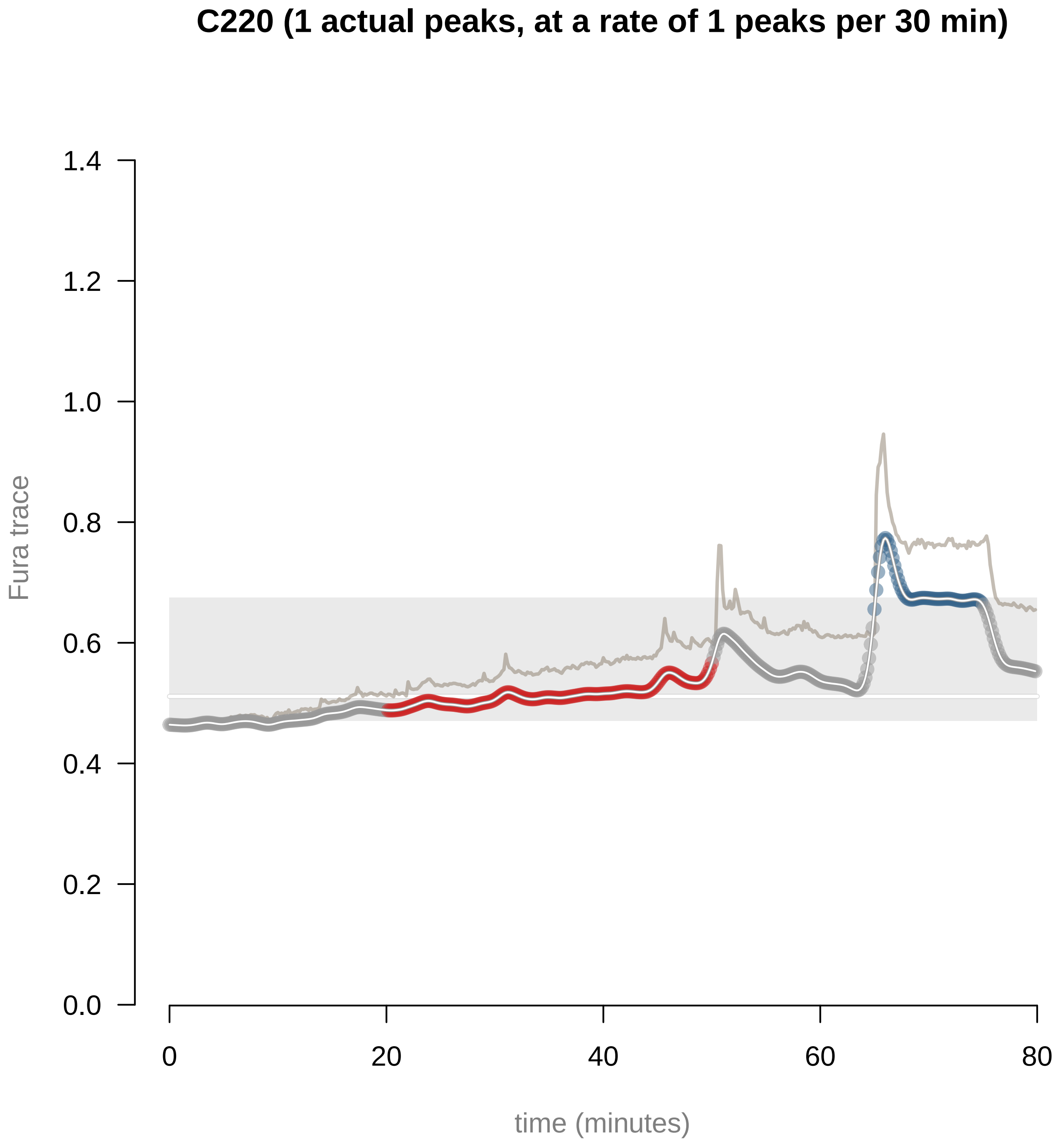

C220 (1 actual peaks, at a rate of 1 peaks per 30 min)
Fura trace
time (minutes)
0.0
0.2
0.4
0.6
0.8
1.0
1.2
1.4
0
20
40
60
80

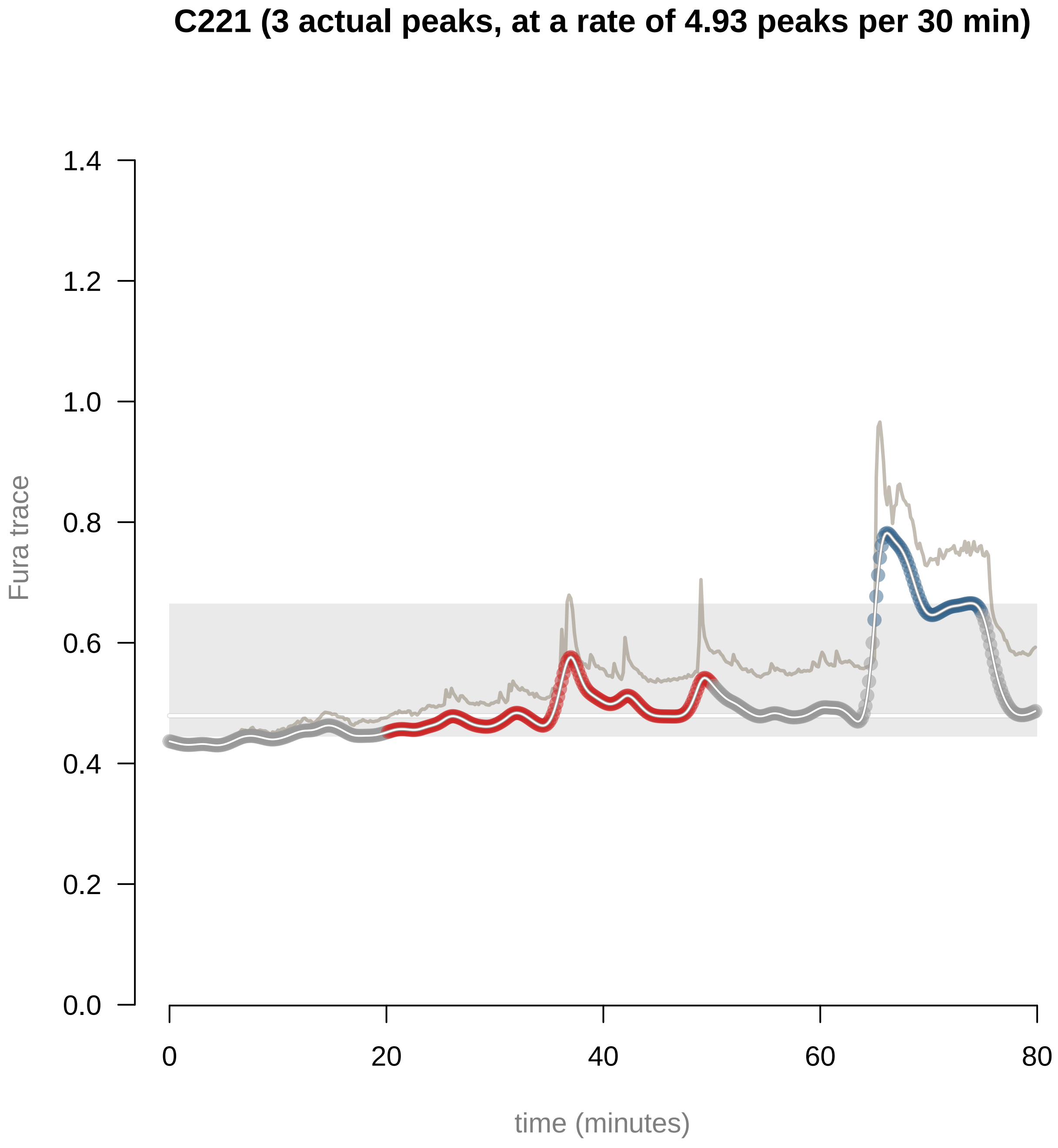

C221 (3 actual peaks, at a rate of 4.93 peaks per 30 min)
Fura trace
0.0
0.2
0.4
0.6
0.8
1.0
1.2
1.4
0
20
40
60
80
time (minutes)

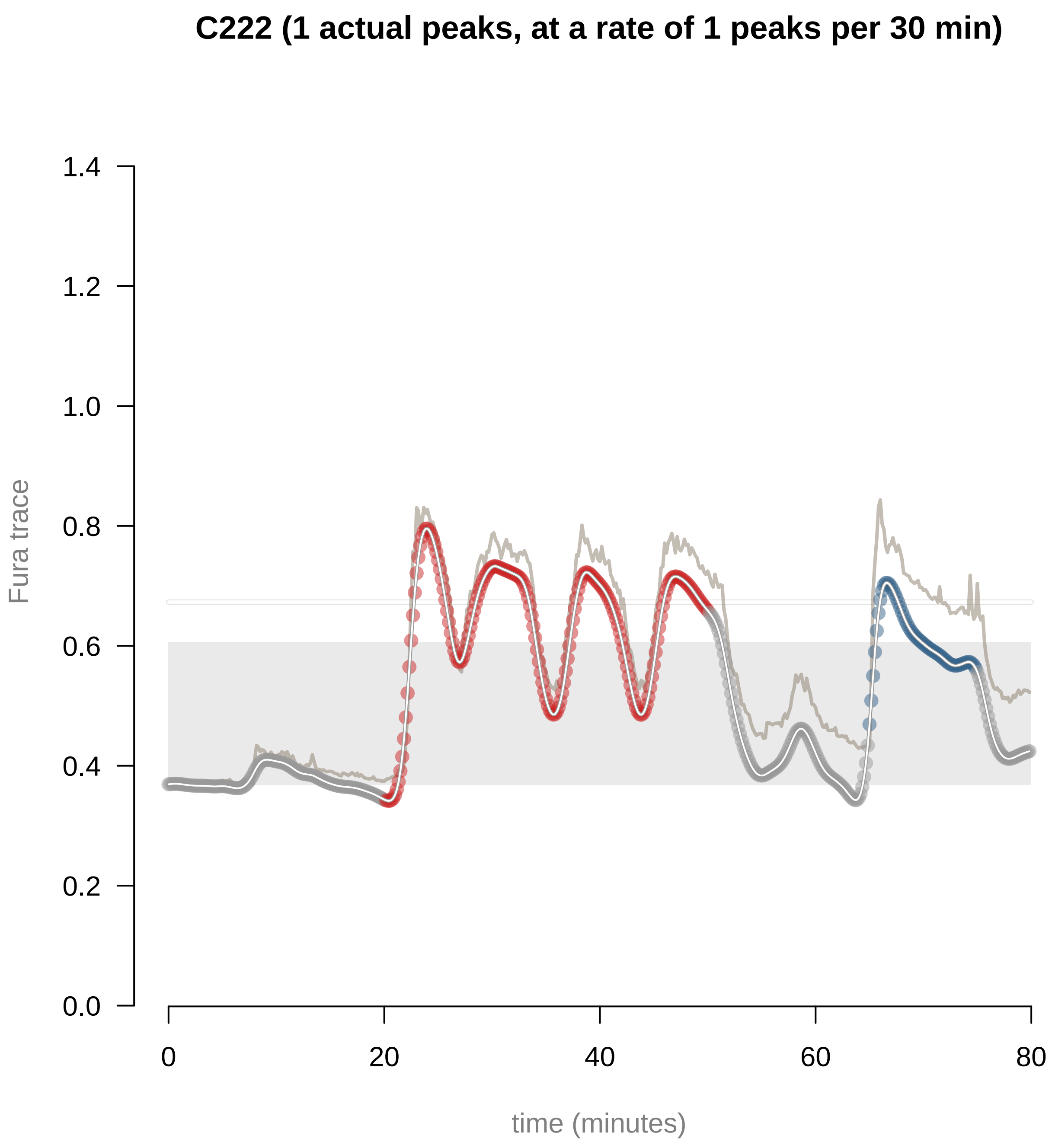
C222 (1 actual peaks, at a rate of 1 peaks per 30 min)
Fura trace
time (minutes)
0.0
0.2
0.4
0.6
0.8
1.0
1.2
1.4
0
20
40
60
80

C223 (0 actual peaks, at a rate of 0 peaks per 30 min)

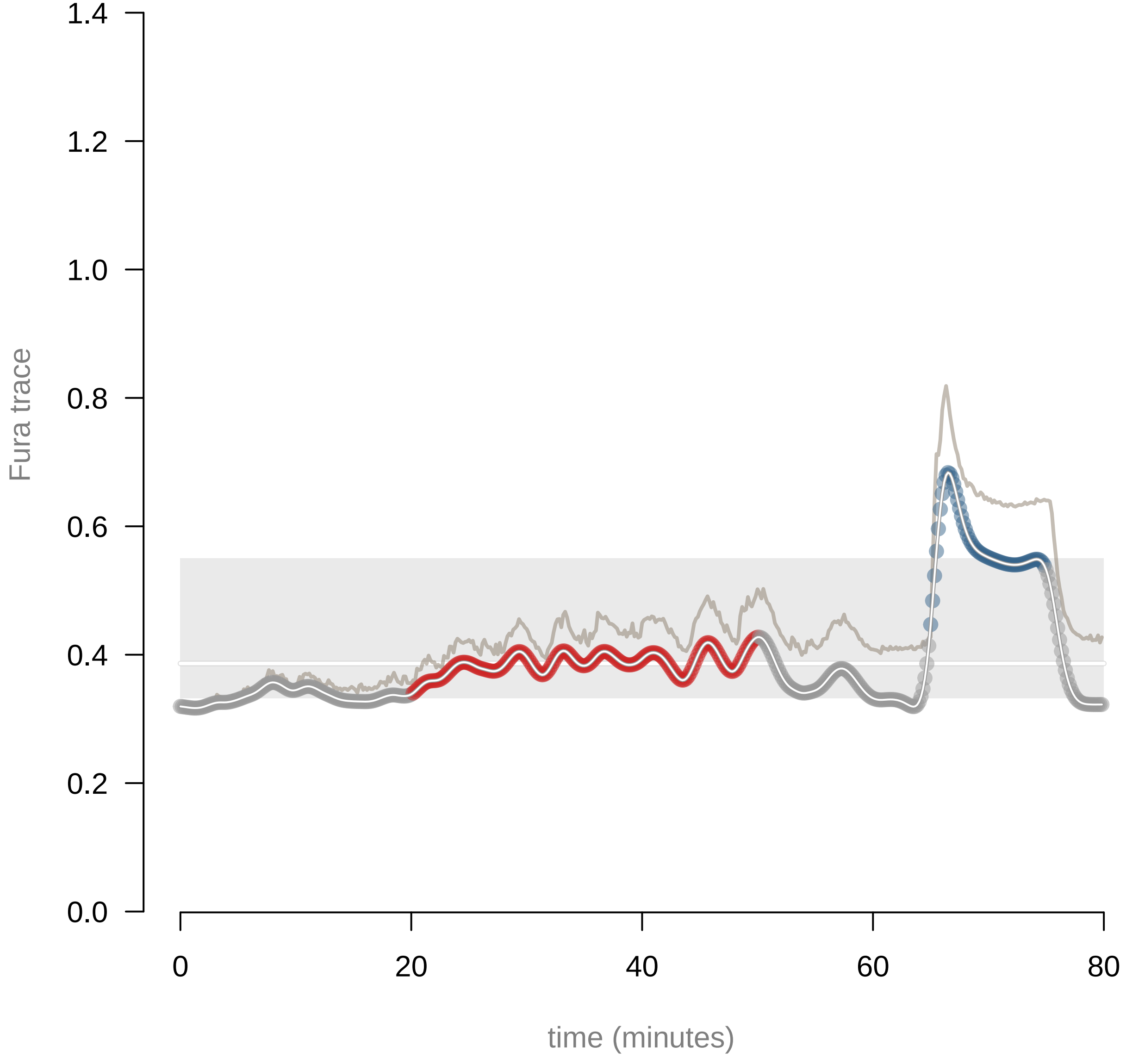

C224 (0 actual peaks, at a rate of 0 peaks per 30 min)

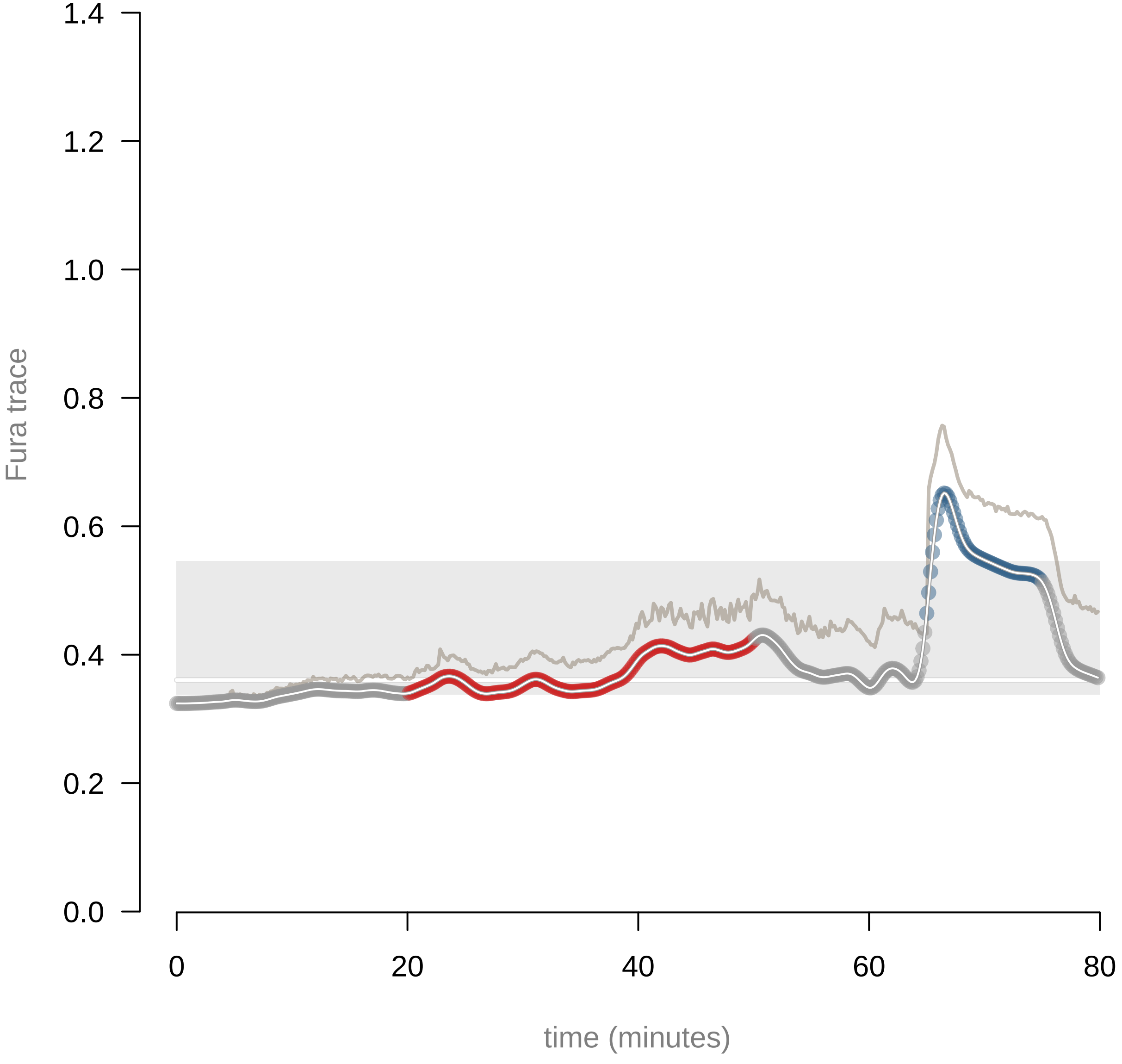

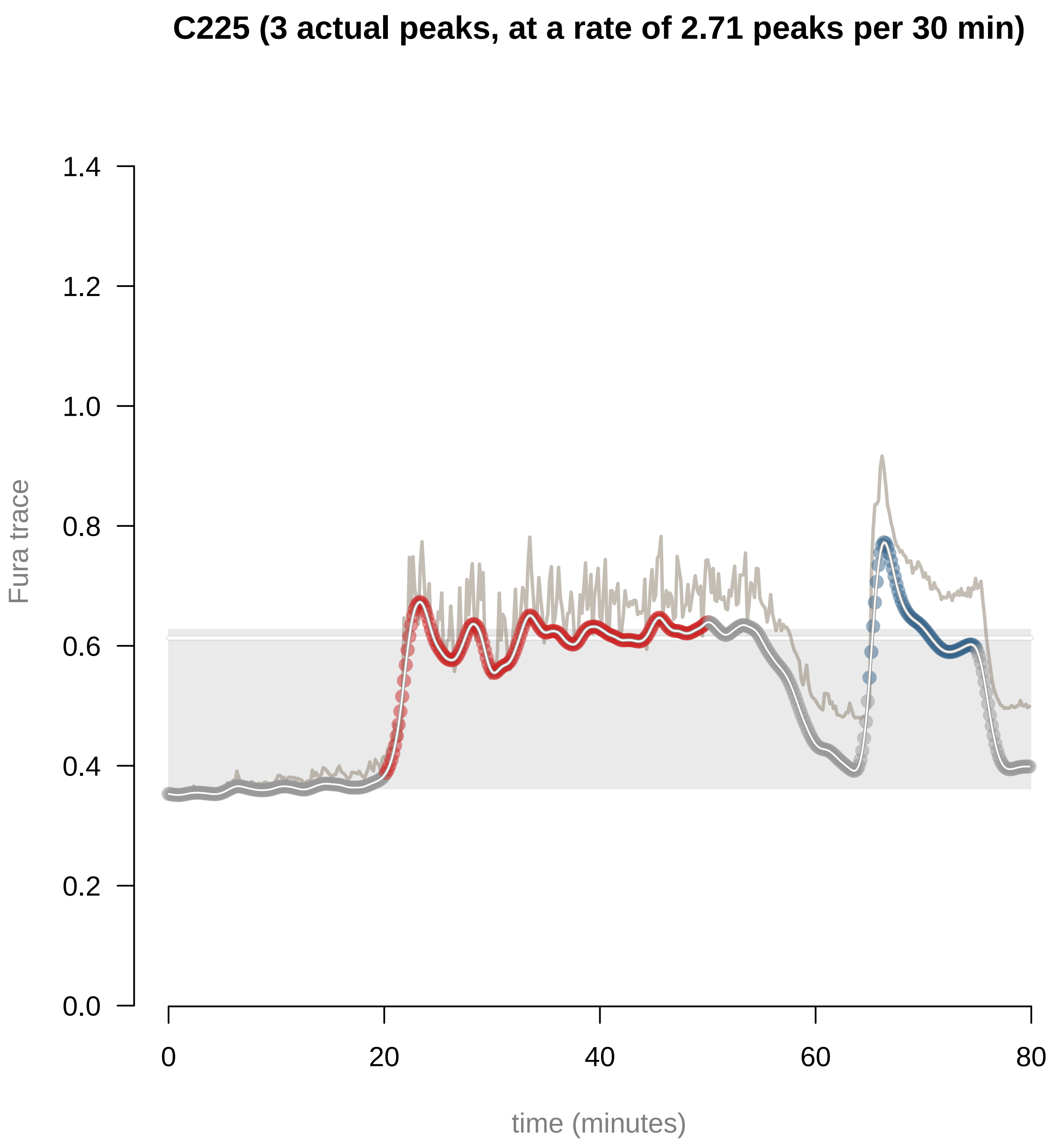

C225 (3 actual peaks, at a rate of 2.71 peaks per 30 min)
Fura trace
time (minutes)
0.0
0.2
0.4
0.6
0.8
1.0
1.2
1.4
0
20
40
60
80

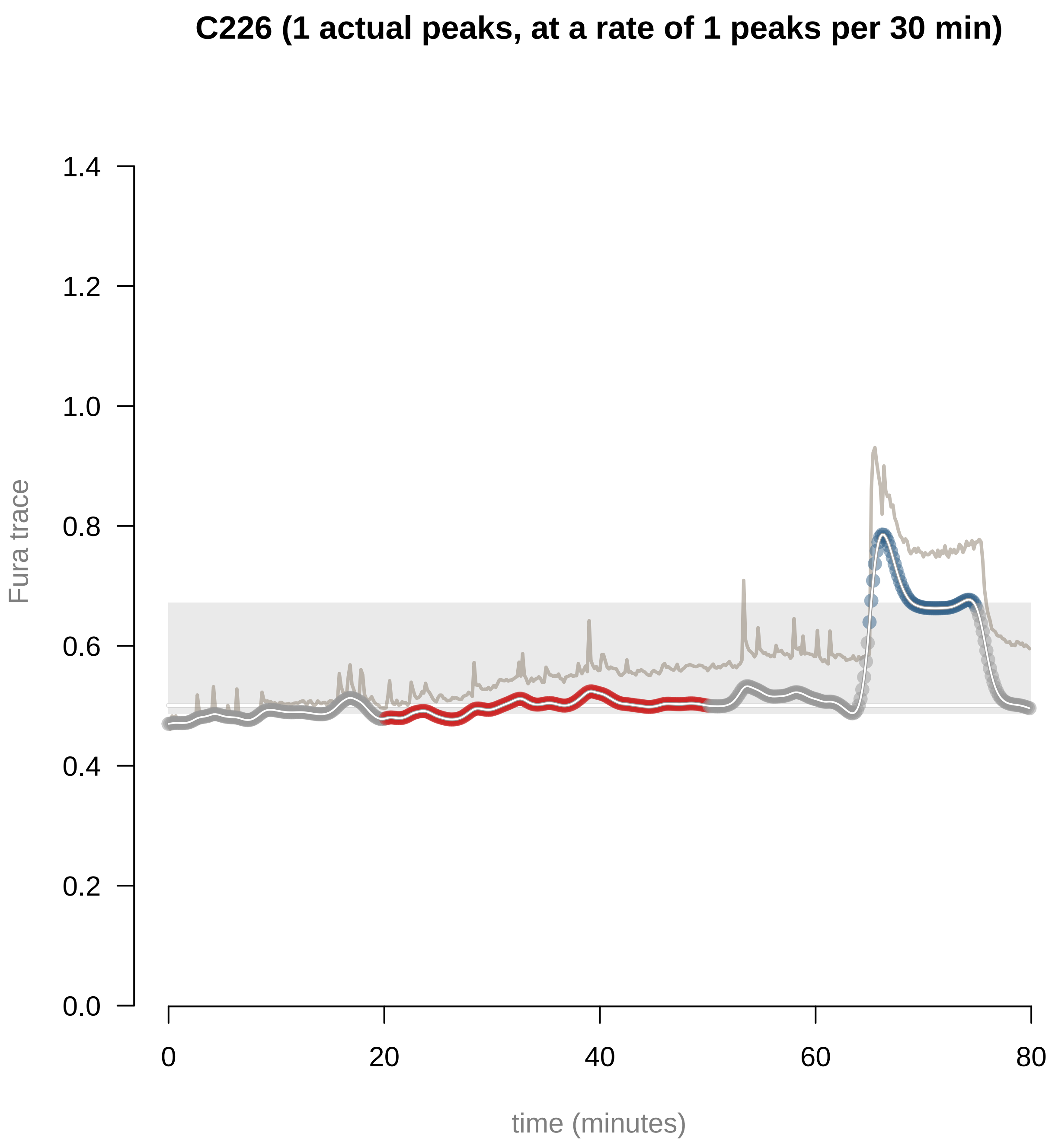
C226 (1 actual peaks, at a rate of 1 peaks per 30 min)
Fura trace
time (minutes)
0.0
0.2
0.4
0.6
0.8
1.0
1.2
1.4
0
20
40
60
80

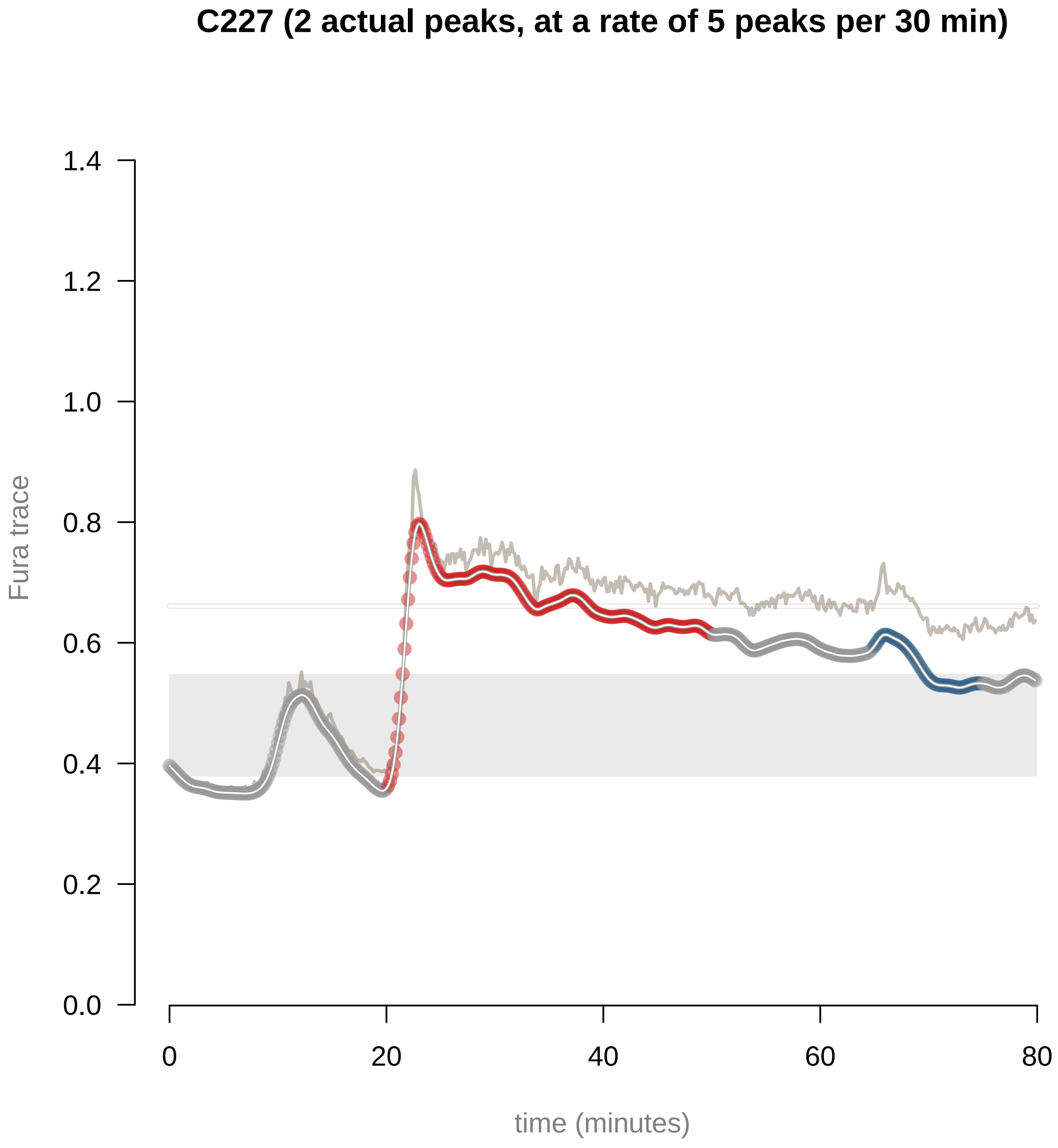

C227 (2 actual peaks, at a rate of 5 peaks per 30 min)
Fura trace
0.0
0.2
0.4
0.6
0.8
1.0
1.2
1.4
0
20
40
60
80
time (minutes)

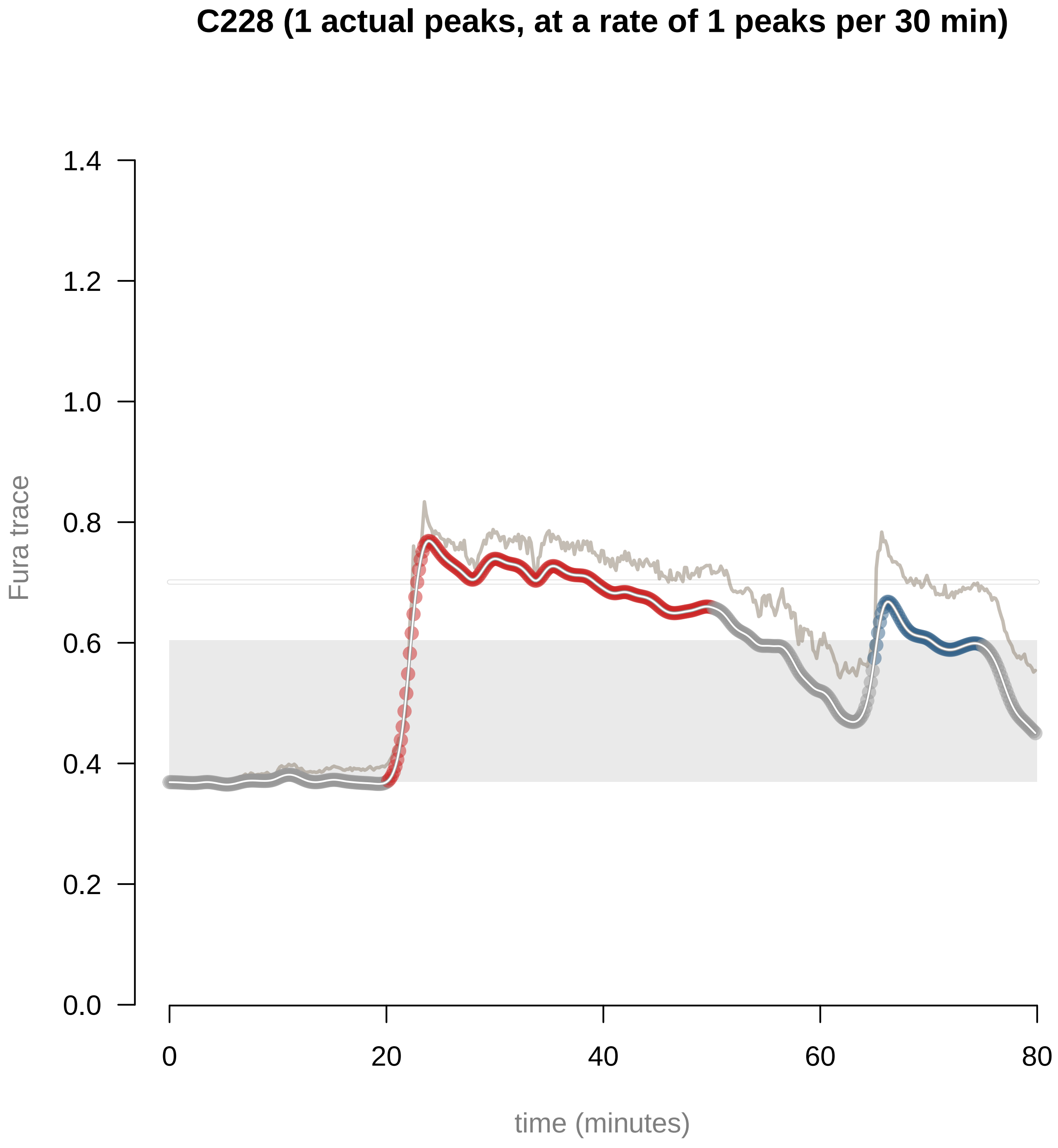

C228 (1 actual peaks, at a rate of 1 peaks per 30 min)
Fura trace
1.4
1.2
1.0
0.8
0.6
0.4
0.2
0.0
0
20
40
60
80
time (minutes)

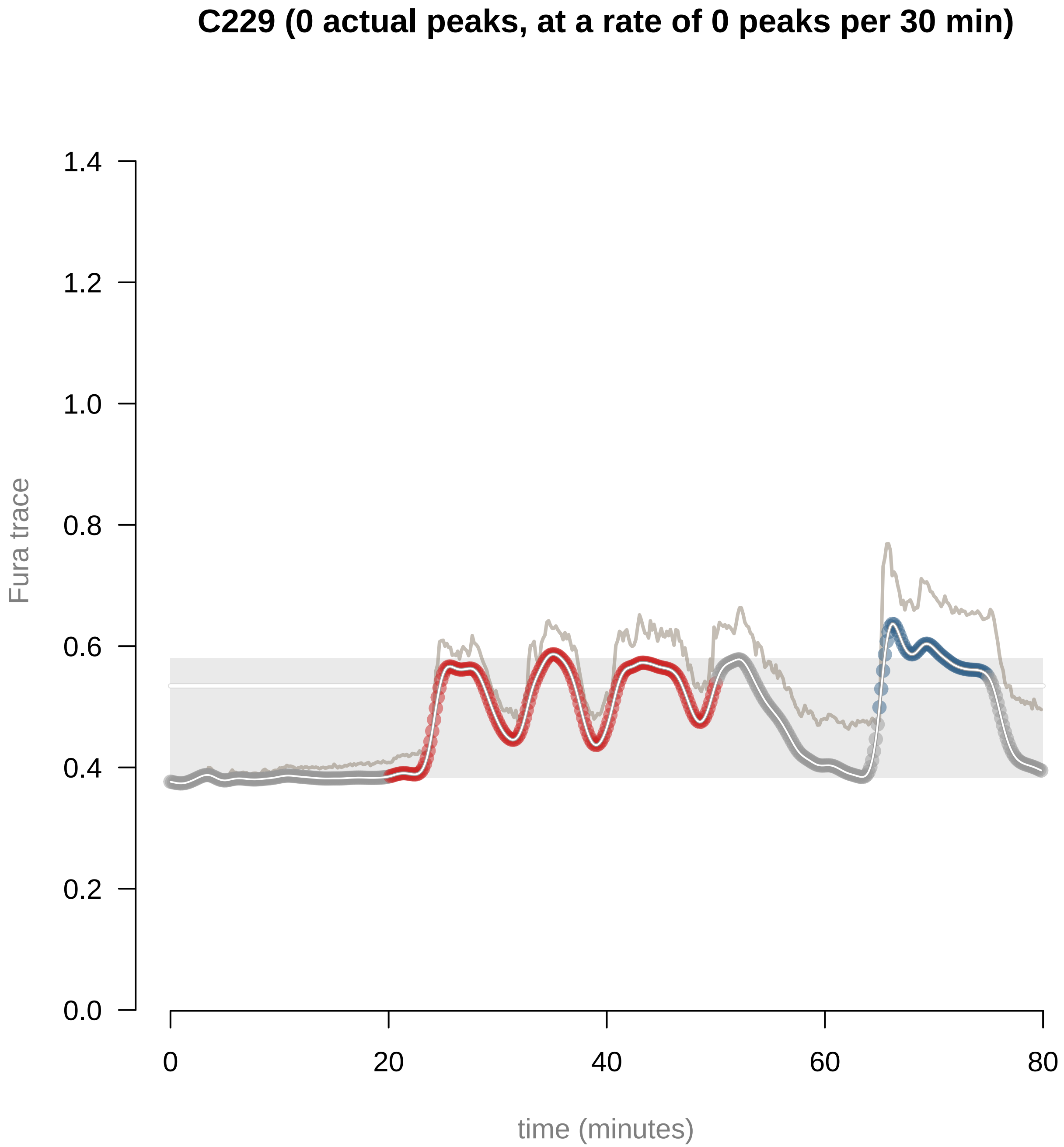
C229 (0 actual peaks, at a rate of 0 peaks per 30 min)
Fura trace
time (minutes)
0.0
0.2
0.4
0.6
0.8
1.0
1.2
1.4
0
20
40
60
80

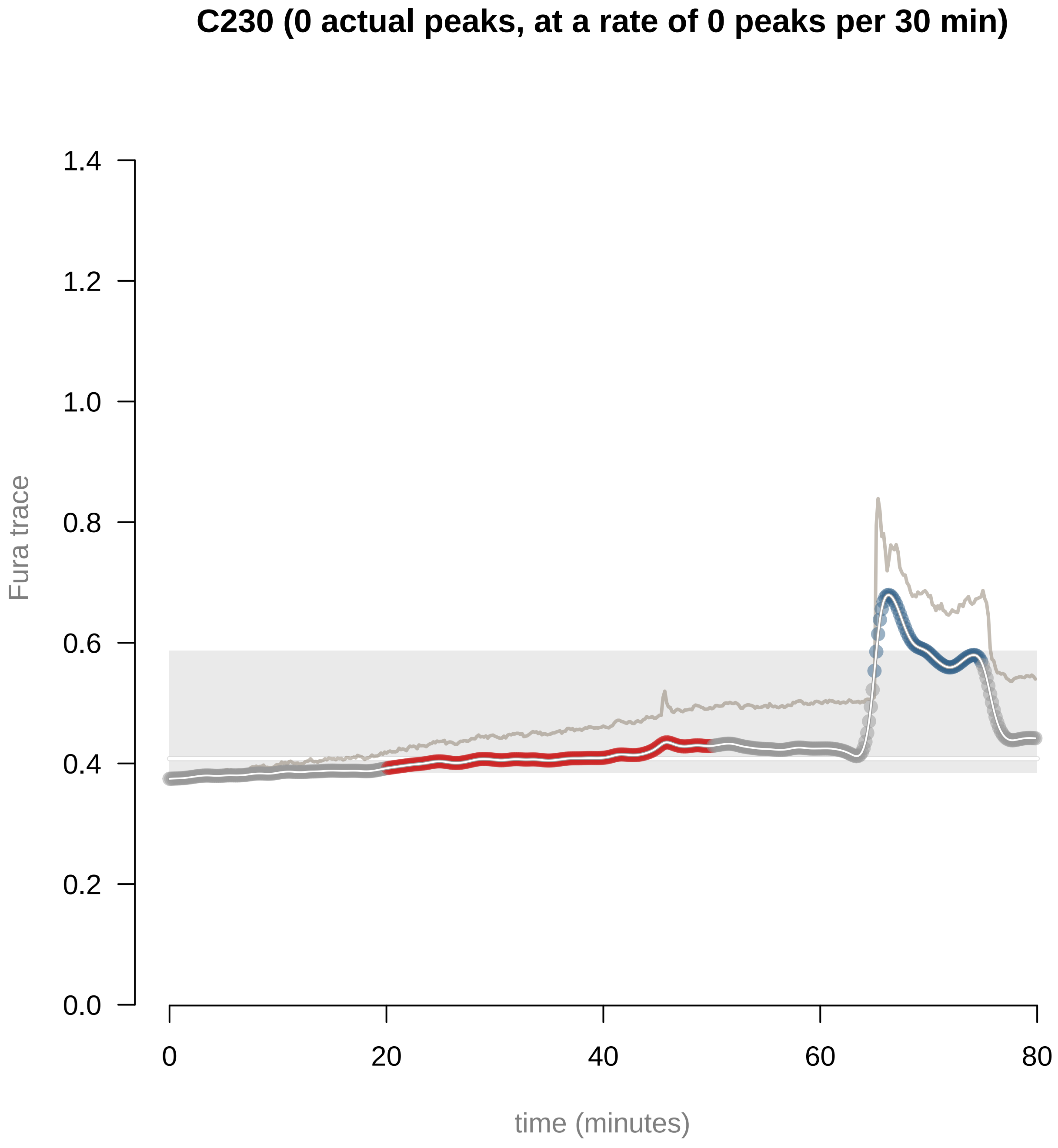

C230 (0 actual peaks, at a rate of 0 peaks per 30 min)
Fura trace
1.4
1.2
1.0
0.8
0.6
0.4
0.2
0.0
0
20
40
60
80
time (minutes)

C231 (1 actual peaks, at a rate of 1 peaks per 30 min)

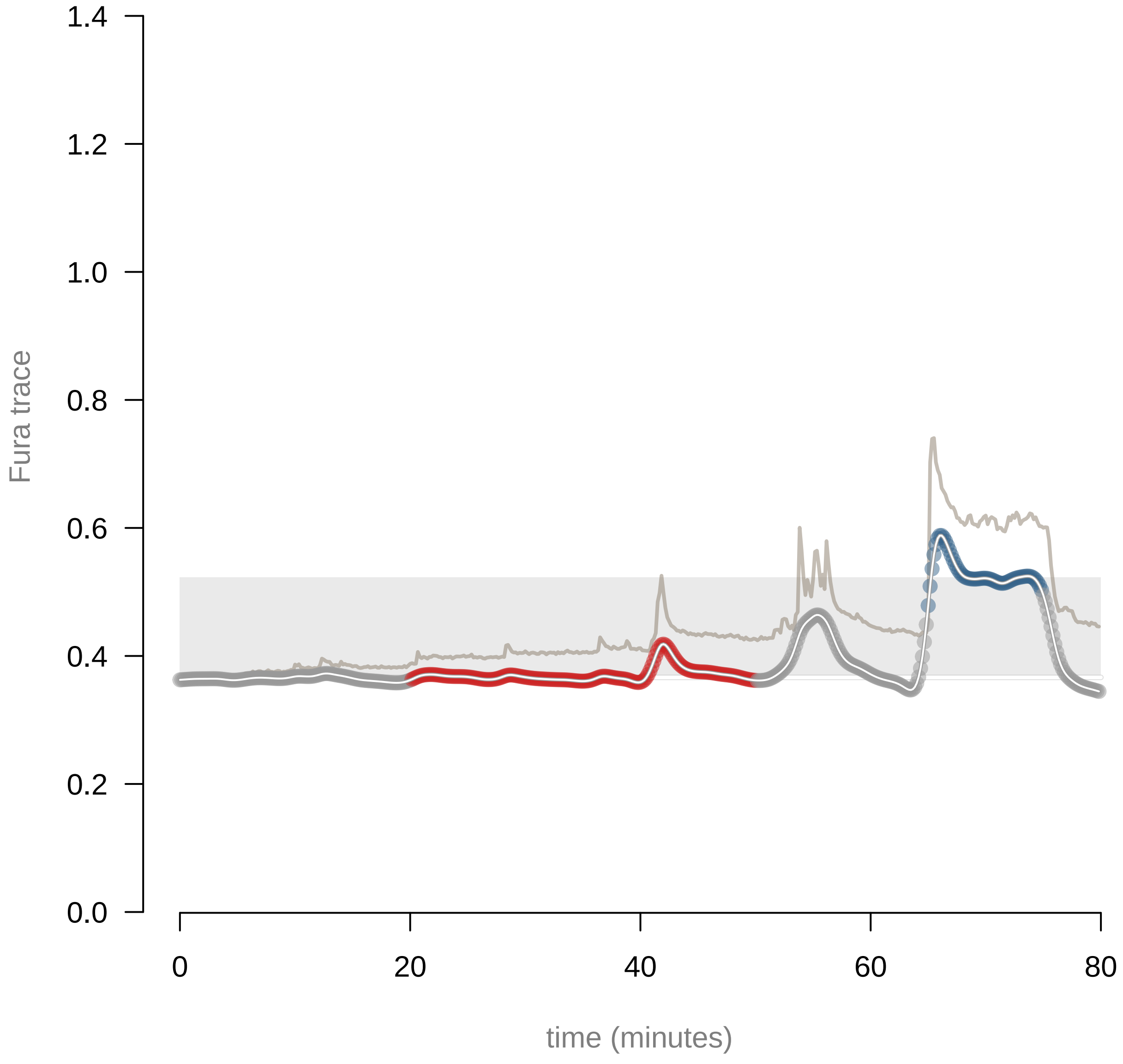

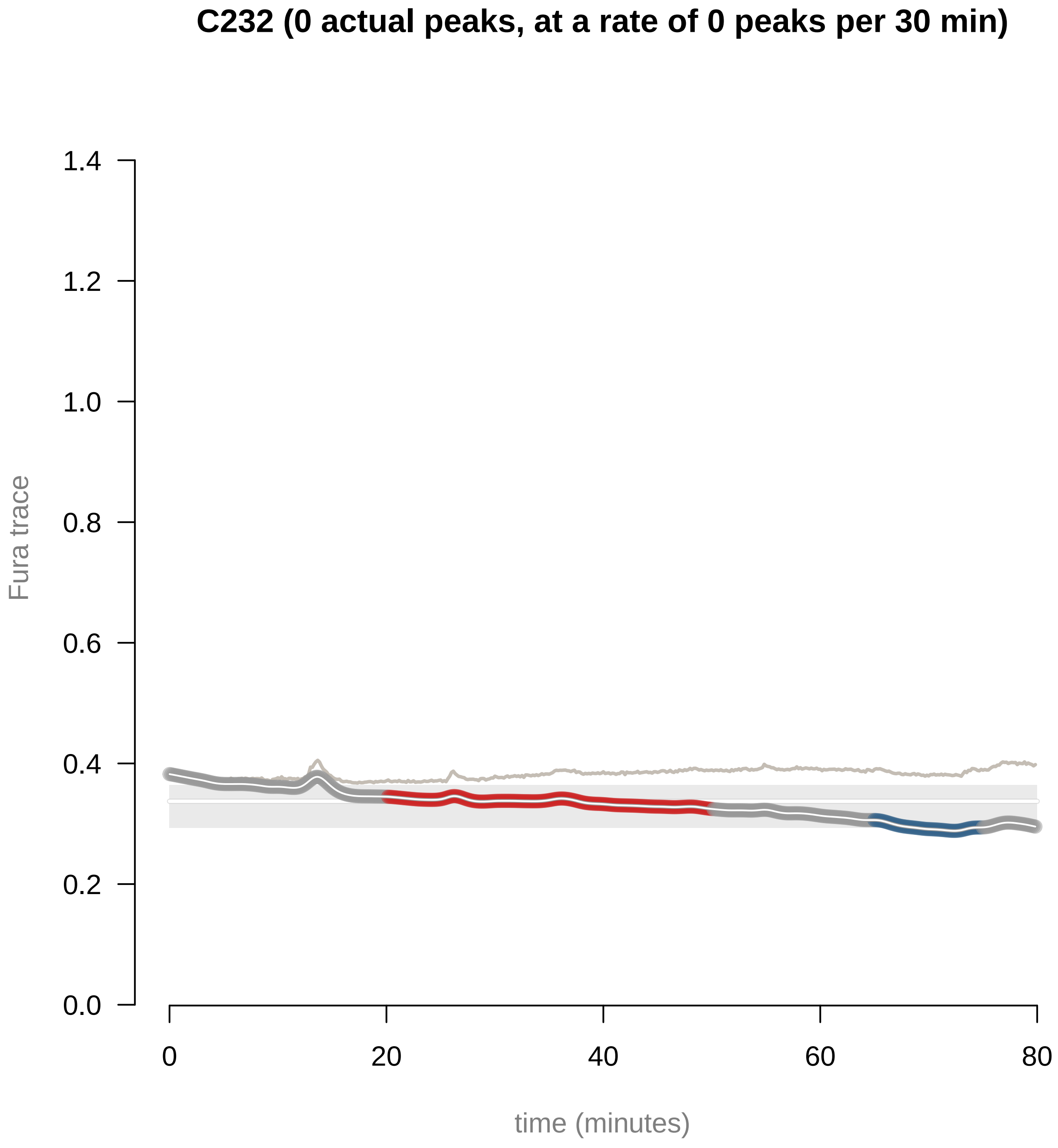
C232 (0 actual peaks, at a rate of 0 peaks per 30 min)
Fura trace
1.4
1.2
1.0
0.8
0.6
0.4
0.2
0.0
0
20
40
60
80
time (minutes)

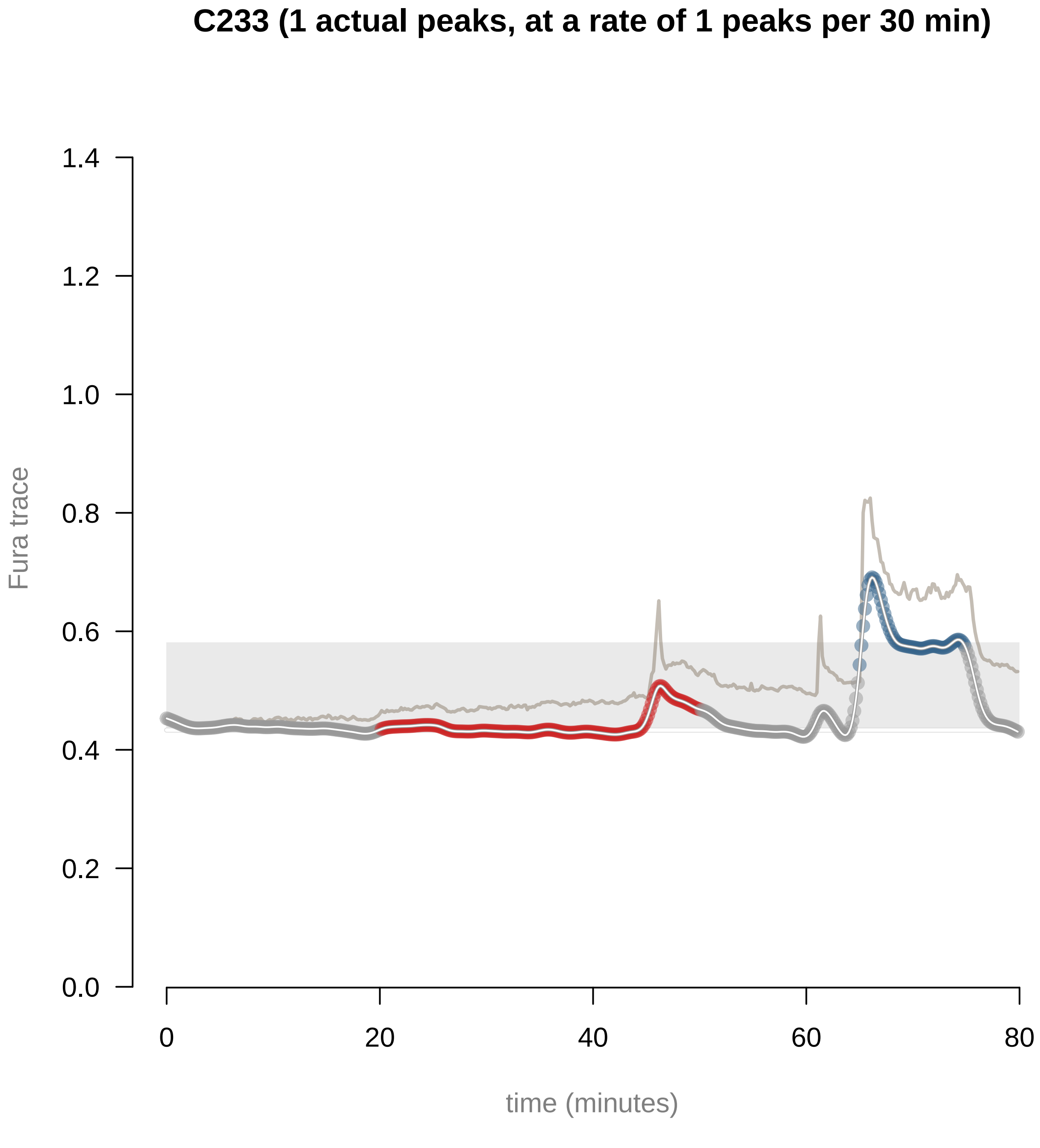
C233 (1 actual peaks, at a rate of 1 peaks per 30 min)
Fura trace
1.4
1.2
1.0
0.8
0.6
0.4
0.2
0.0
0
20
40
60
80
time (minutes)

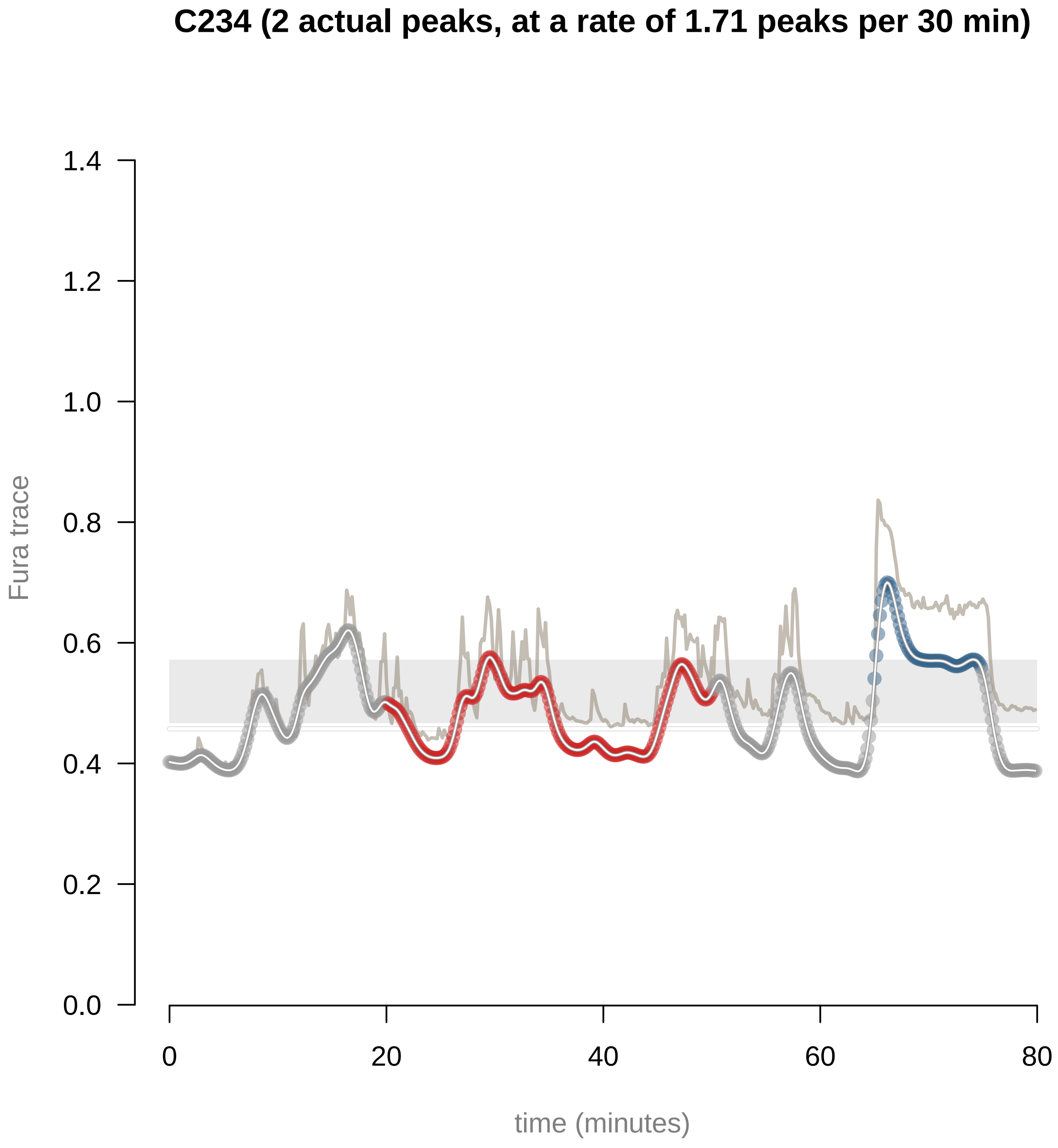

C234 (2 actual peaks, at a rate of 1.71 peaks per 30 min)
Fura trace
1.4
1.2
1.0
0.8
0.6
0.4
0.2
0.0
0
20
40
60
80
time (minutes)

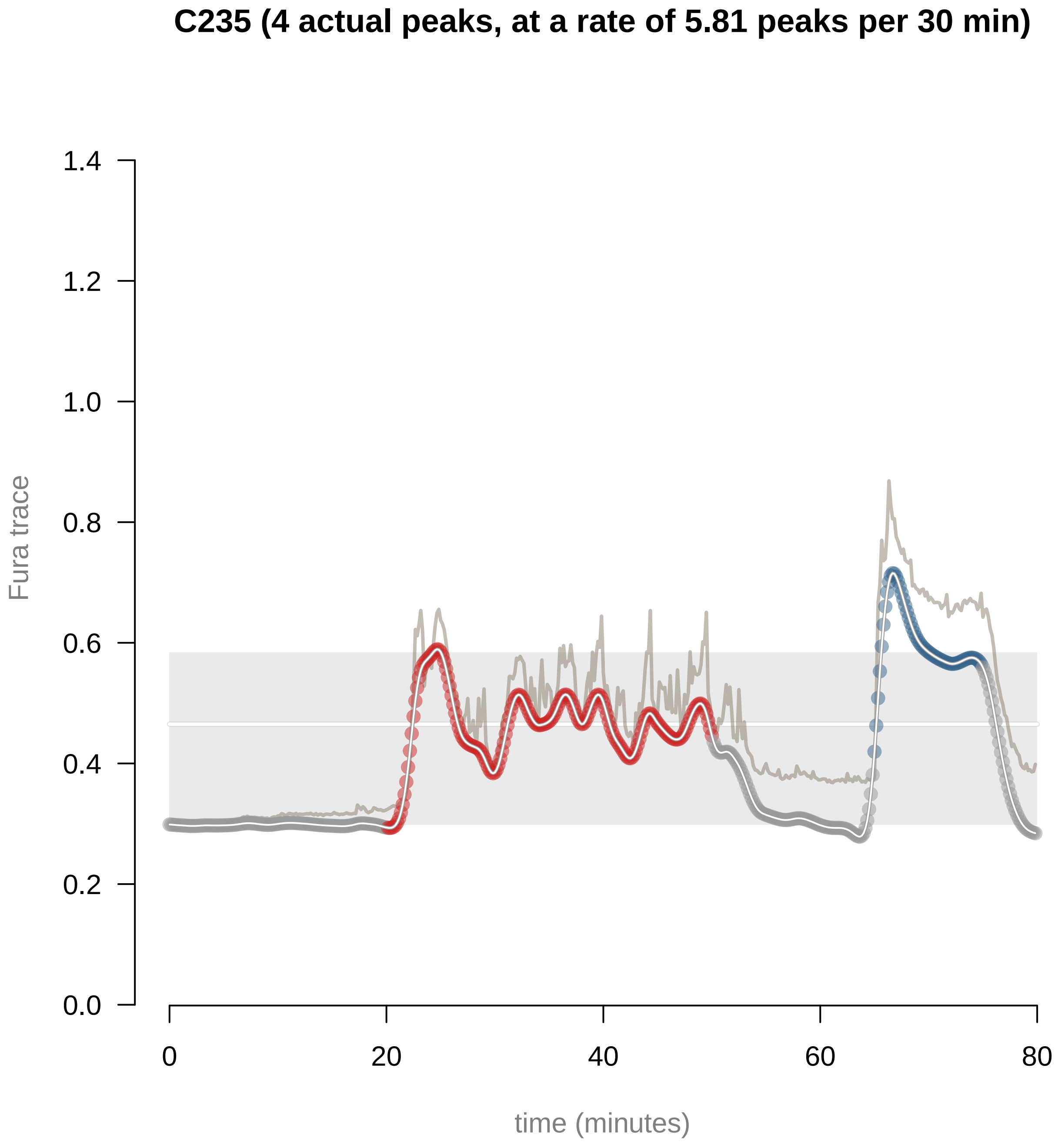

C235 (4 actual peaks, at a rate of 5.81 peaks per 30 min)
Fura trace
0.0
0.2
0.4
0.6
0.8
1.0
1.2
1.4
0
20
40
60
80
time (minutes)

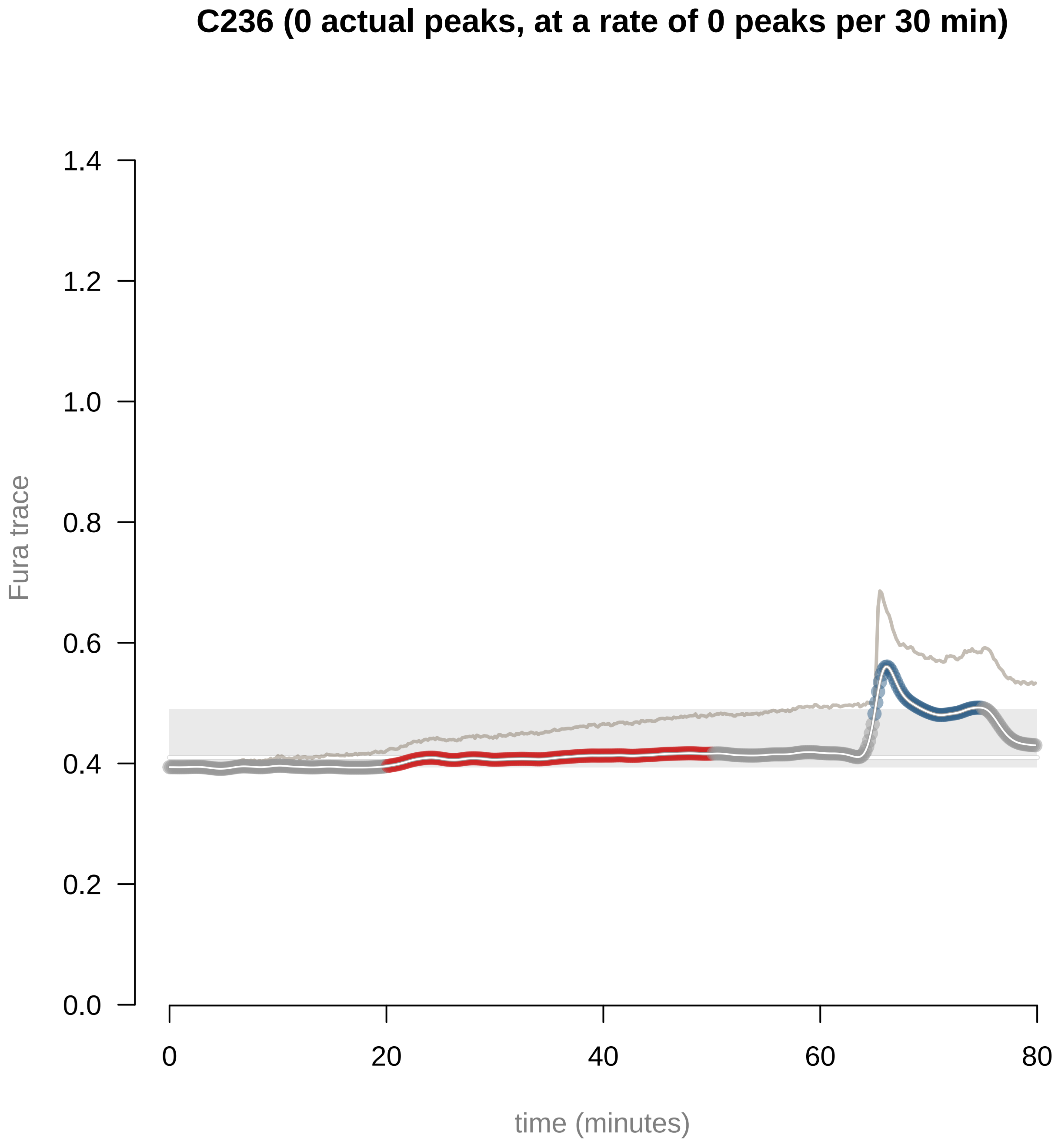

C236 (0 actual peaks, at a rate of 0 peaks per 30 min)
Fura trace
time (minutes)
1.4
1.2
1.0
0.8
0.6
0.4
0.2
0.0
0
20
40
60
80

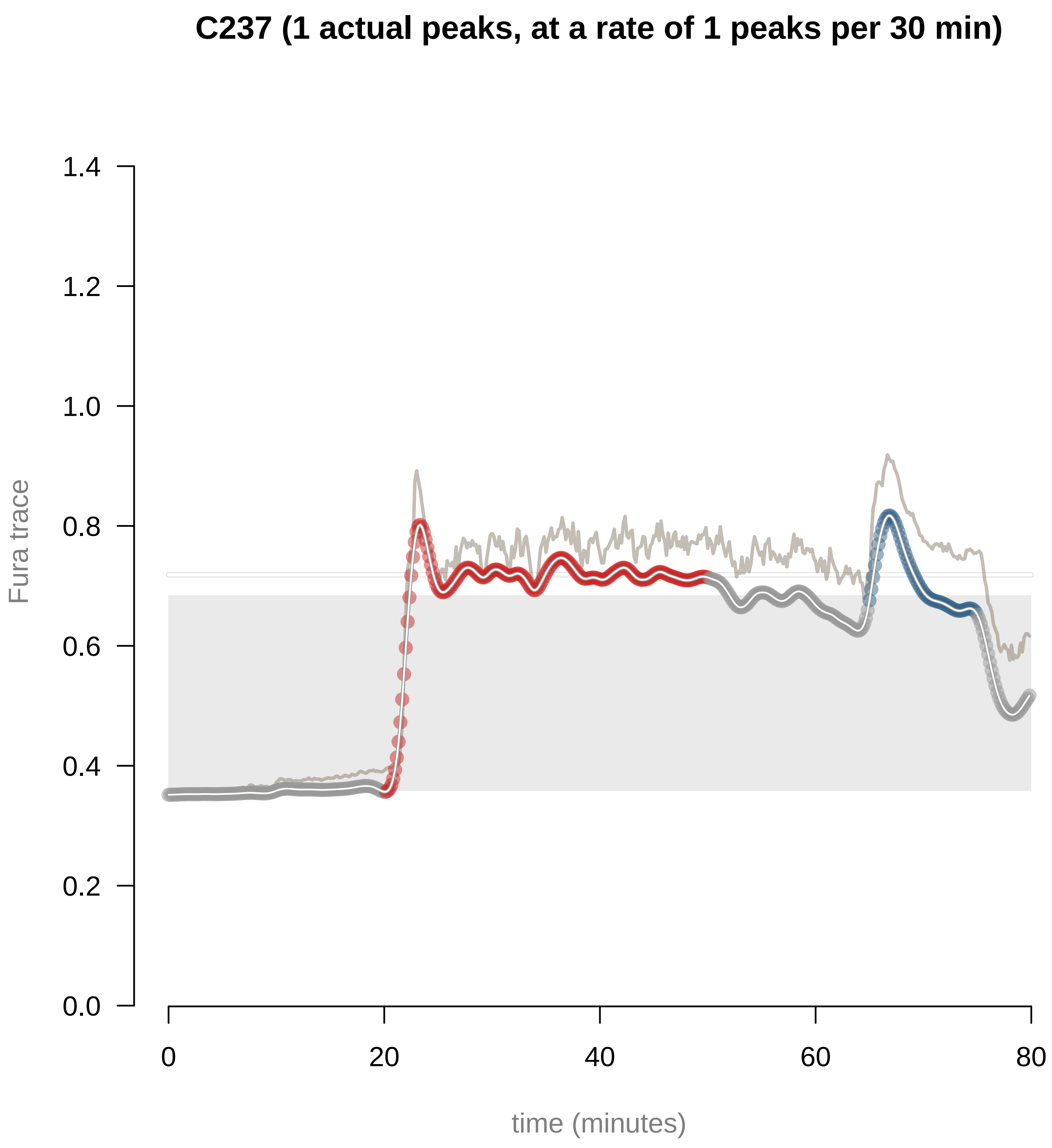

C237 (1 actual peaks, at a rate of 1 peaks per 30 min)
Fura trace
0.0
0.2
0.4
0.6
0.8
1.0
1.2
1.4
0
20
40
60
80
time (minutes)

C238 (1 actual peaks, at a rate of 1 peaks per 30 min)

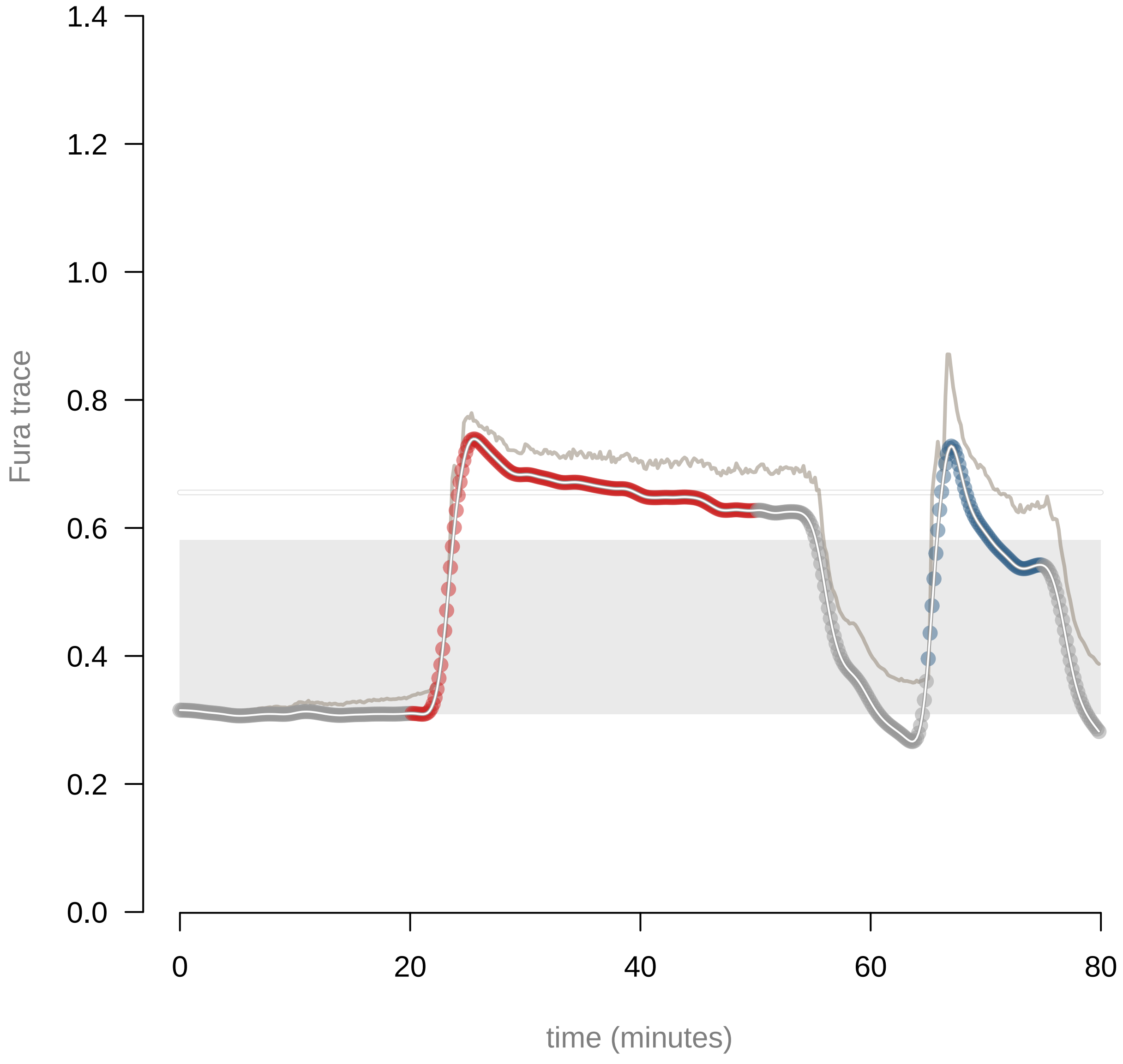

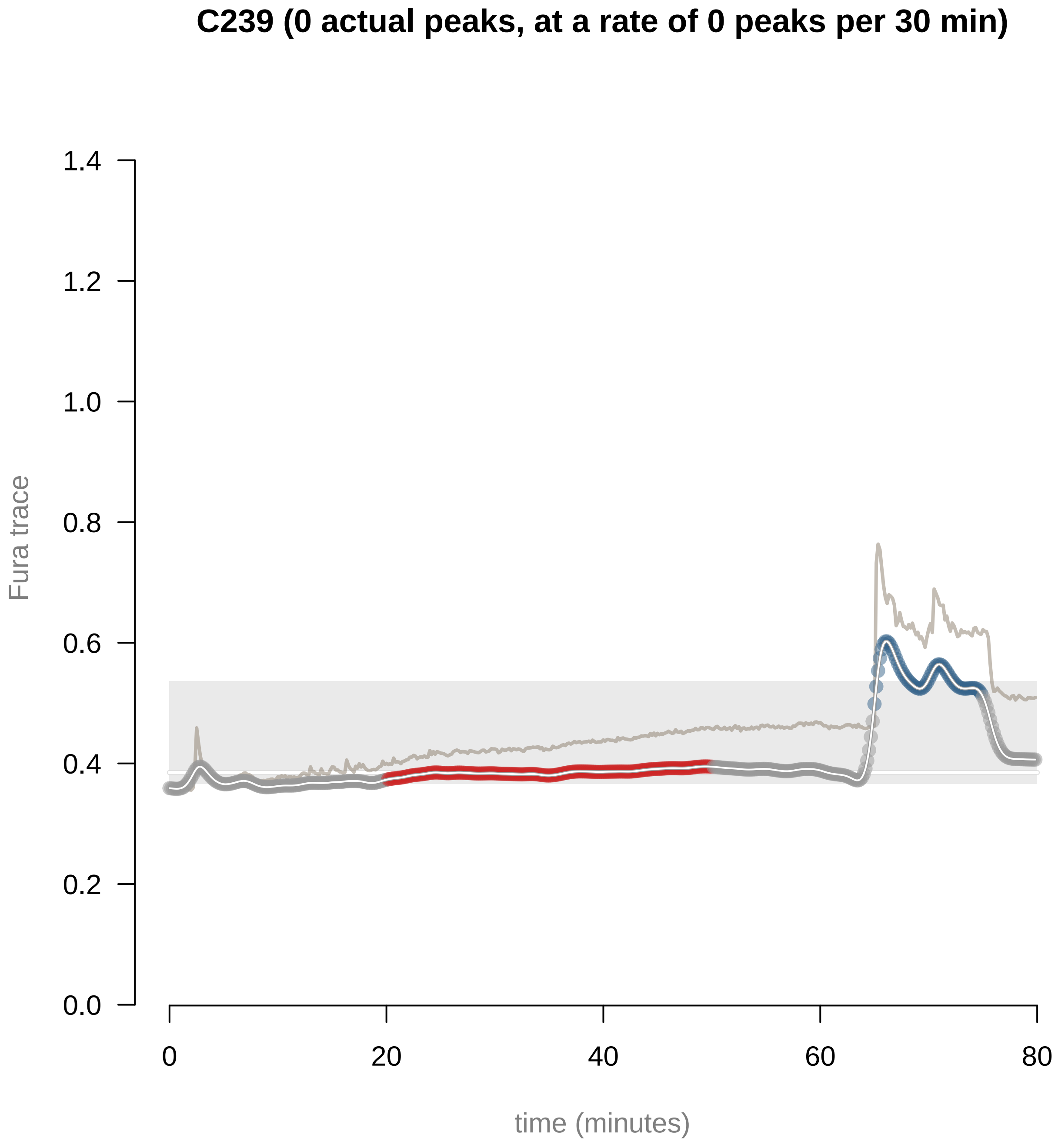

C239 (0 actual peaks, at a rate of 0 peaks per 30 min)
Fura trace
1.4
1.2
1.0
0.8
0.6
0.4
0.2
0.0
0
20
40
60
80
time (minutes)

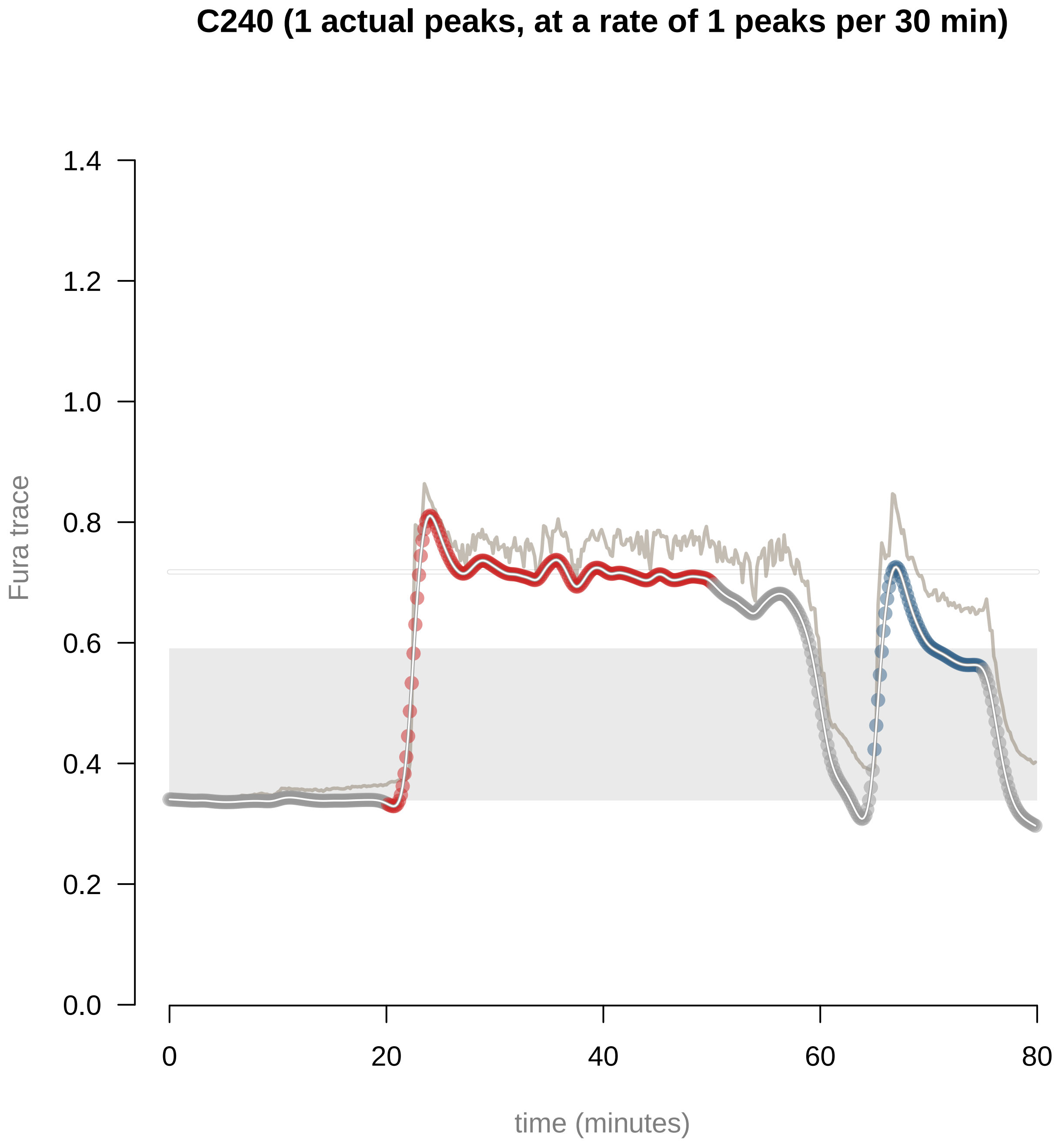
C240 (1 actual peaks, at a rate of 1 peaks per 30 min)
Fura trace
1.4
1.2
1.0
0.8
0.6
0.4
0.2
0.0
0
20
40
60
80
time (minutes)

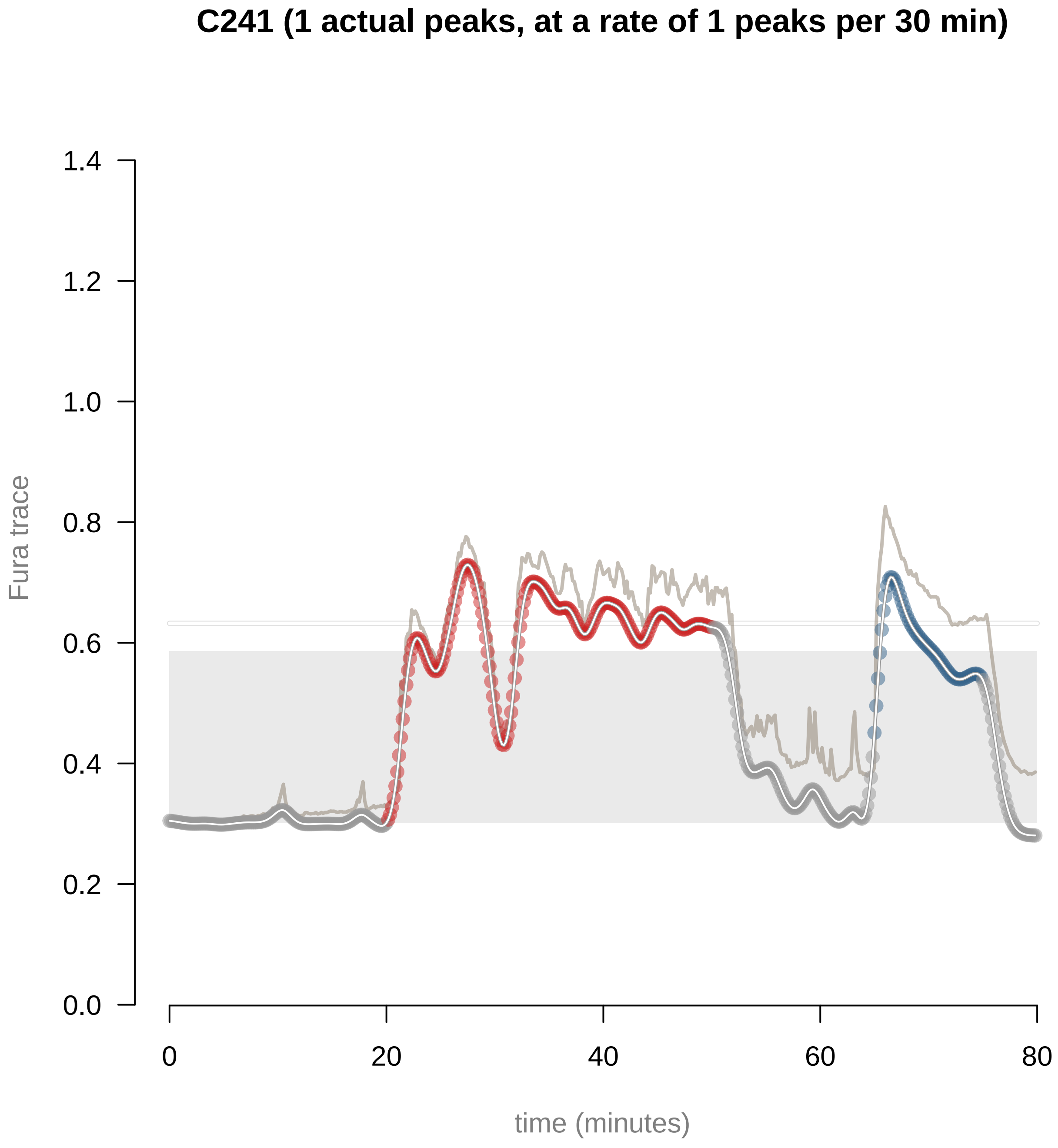

C241 (1 actual peaks, at a rate of 1 peaks per 30 min)
Fura trace
time (minutes)
0.0
0.2
0.4
0.6
0.8
1.0
1.2
1.4
0
20
40
60
80

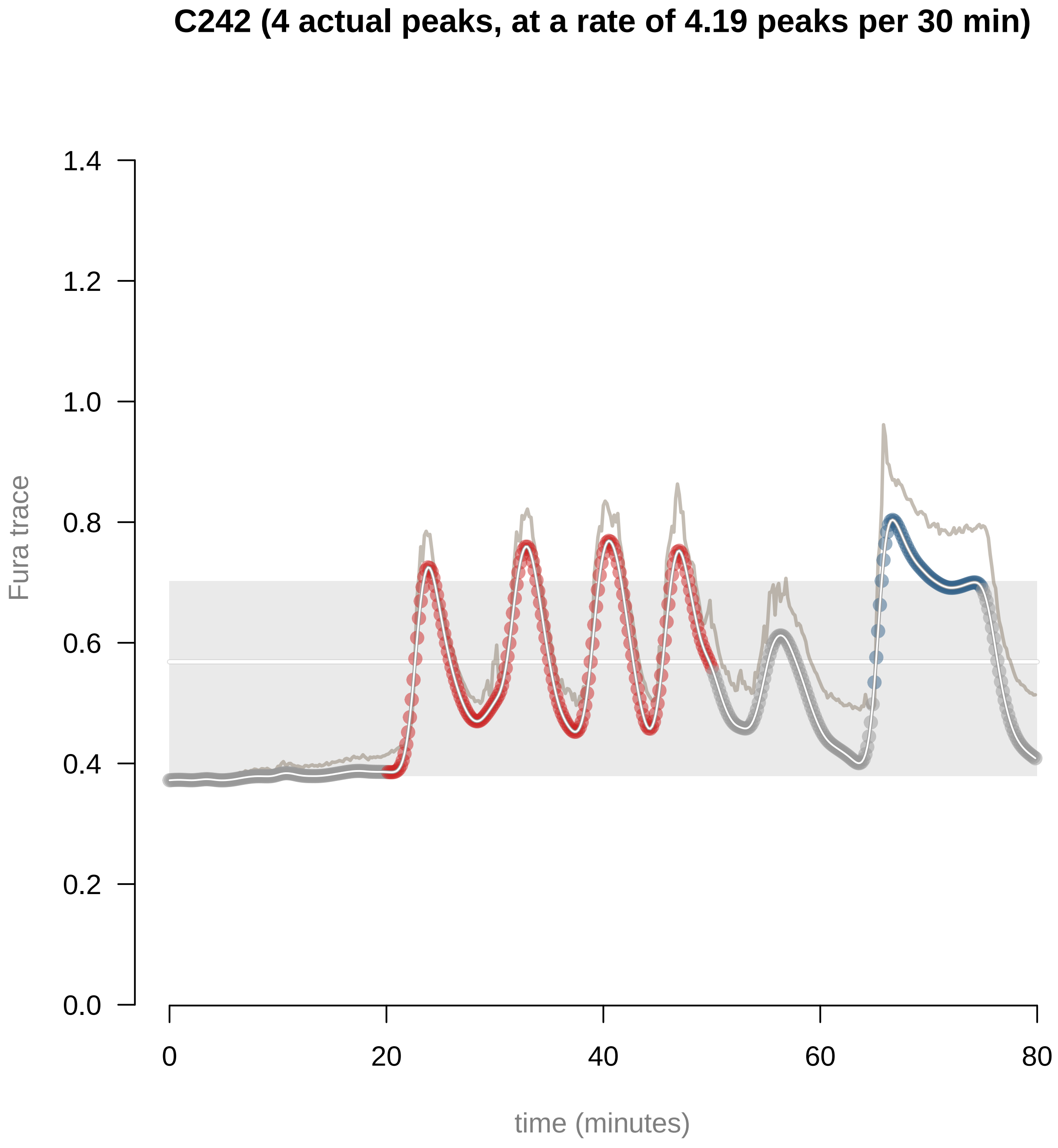

C242 (4 actual peaks, at a rate of 4.19 peaks per 30 min)
Fura trace
1.4
1.2
1.0
0.8
0.6
0.4
0.2
0.0
0
20
40
60
80
time (minutes)

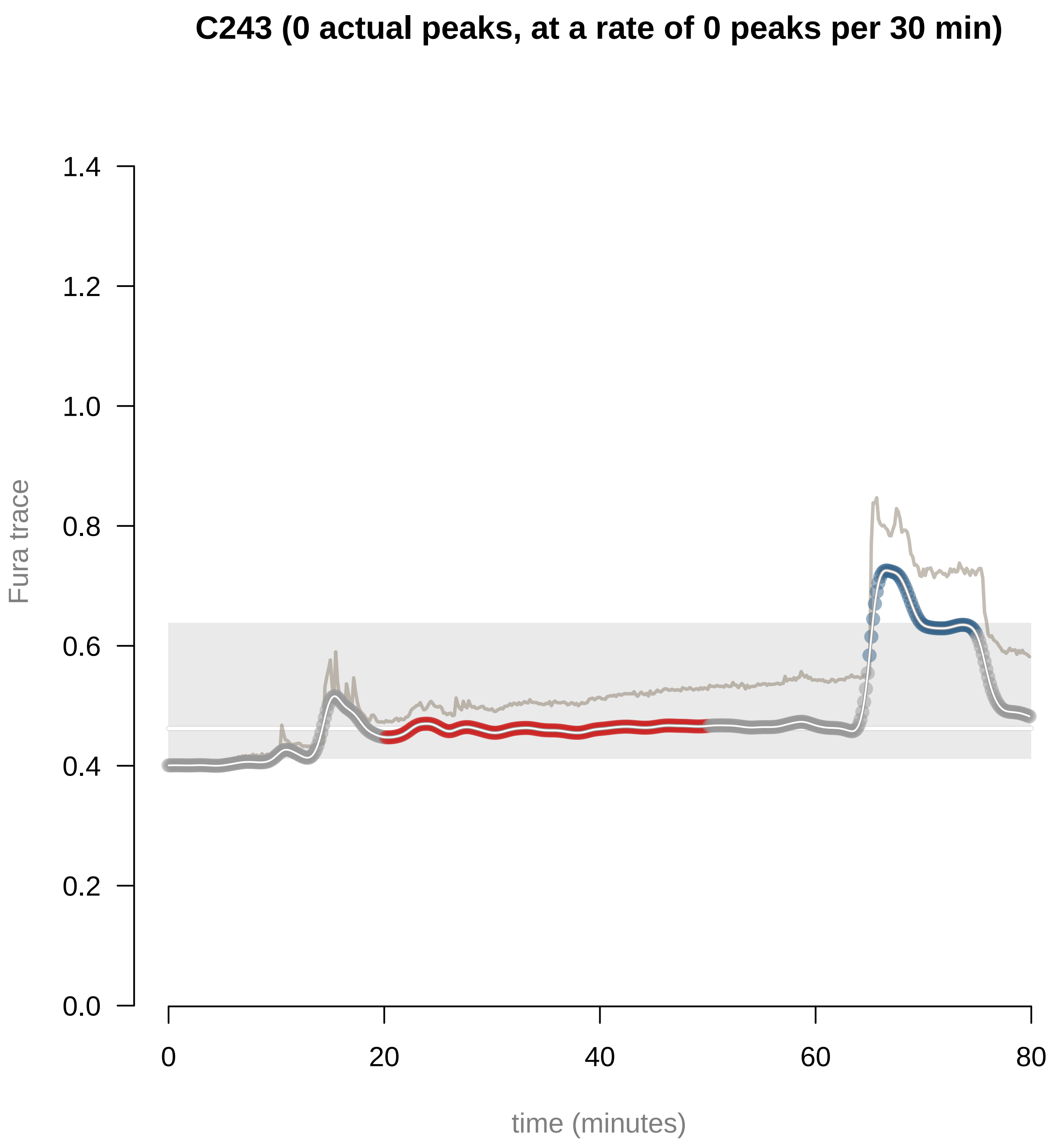
C243 (0 actual peaks, at a rate of 0 peaks per 30 min)
Fura trace
1.4
1.2
1.0
0.8
0.6
0.4
0.2
0.0
0
20
40
60
80
time (minutes)

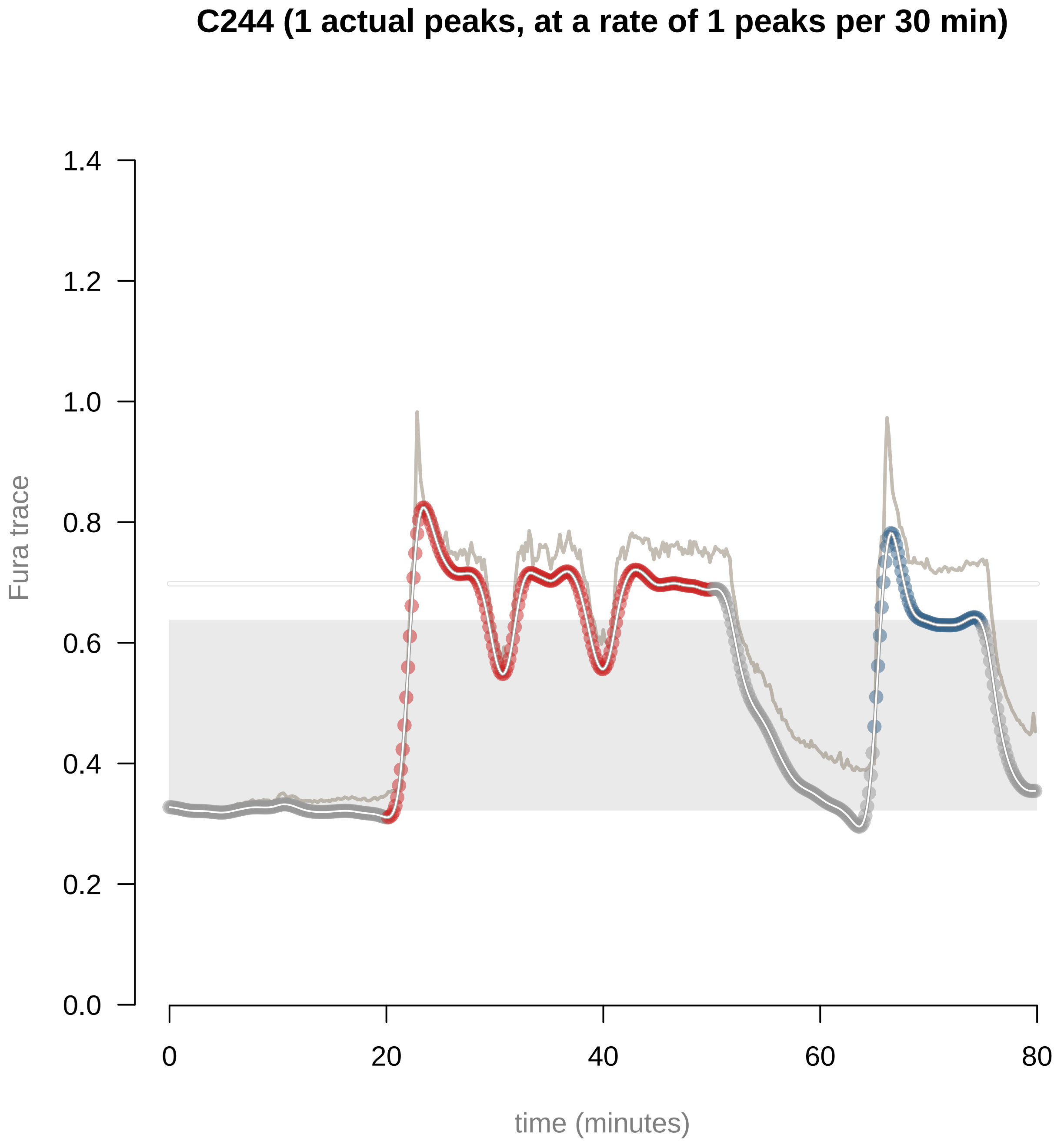

C244 (1 actual peaks, at a rate of 1 peaks per 30 min)
Fura trace
time (minutes)
0.0
0.2
0.4
0.6
0.8
1.0
1.2
1.4
0
20
40
60
80

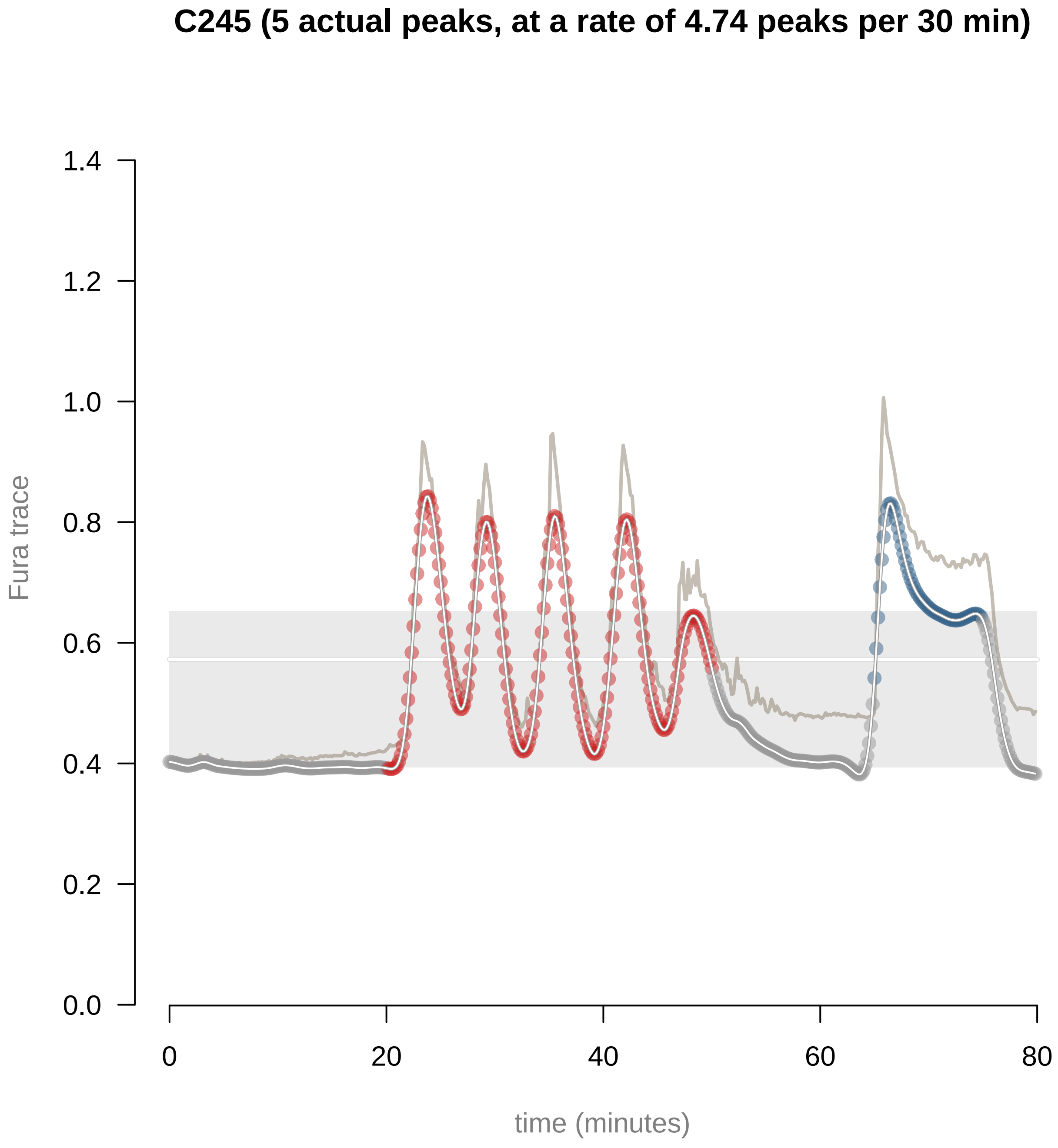
C245 (5 actual peaks, at a rate of 4.74 peaks per 30 min)
Fura trace
1.4
1.2
1.0
0.8
0.6
0.4
0.2
0.0
0
20
40
60
80
time (minutes)

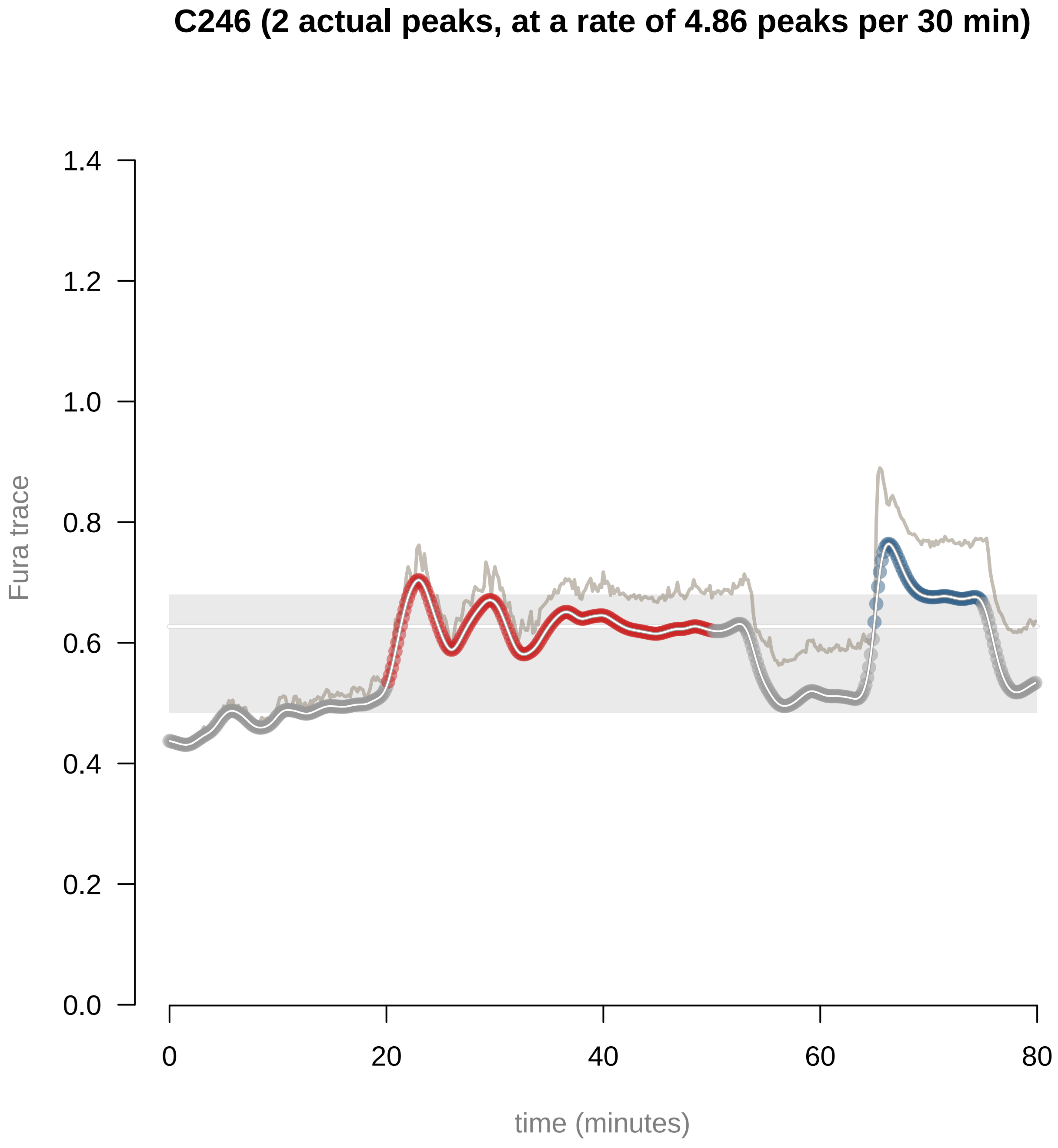
C246 (2 actual peaks, at a rate of 4.86 peaks per 30 min)
Fura trace
1.4
1.2
1.0
0.8
0.6
0.4
0.2
0.0
0
20
40
60
80
time (minutes)

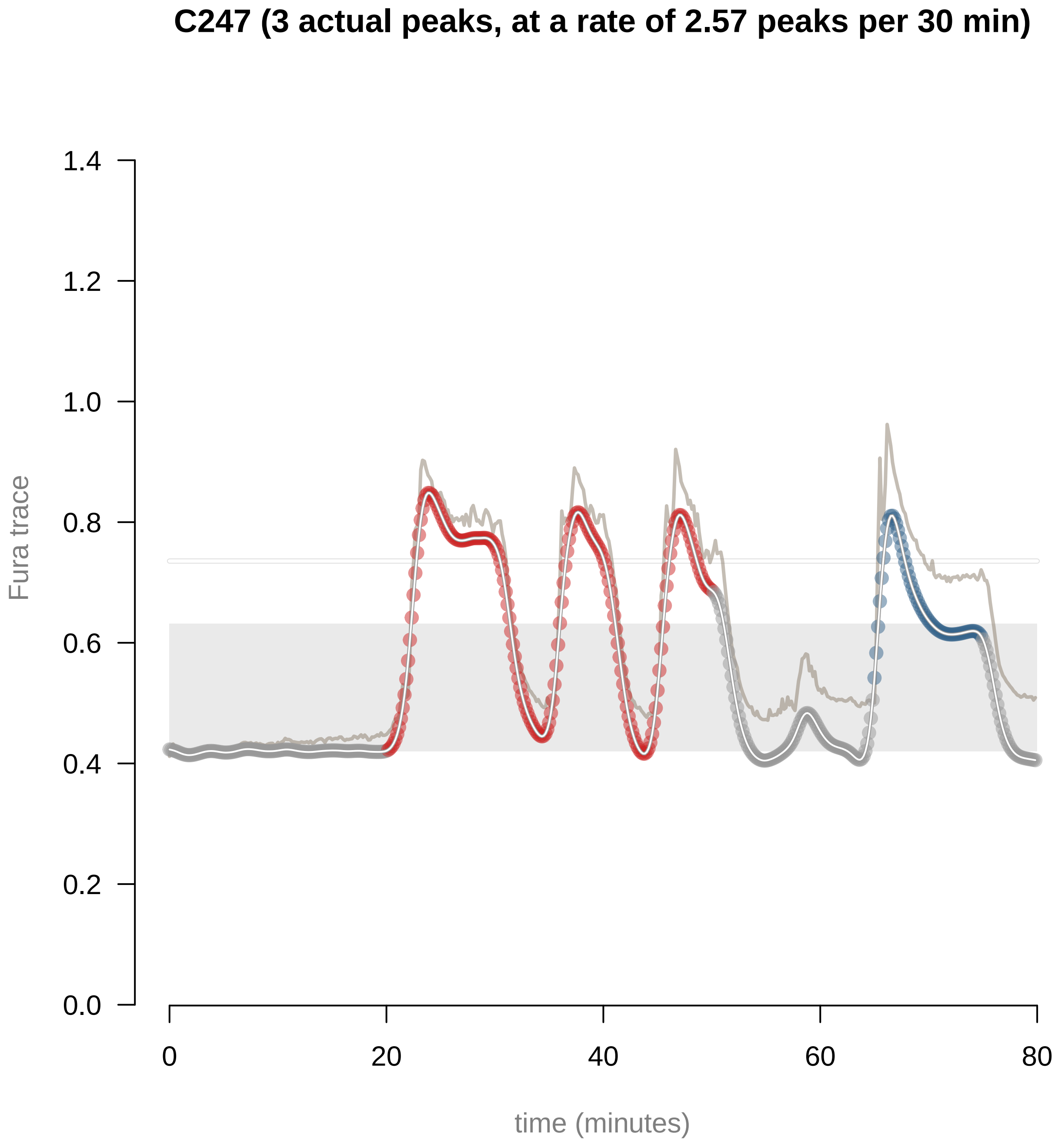
C247 (3 actual peaks, at a rate of 2.57 peaks per 30 min)
Fura trace
1.4
1.2
1.0
0.8
0.6
0.4
0.2
0.0
0
20
40
60
80
time (minutes)

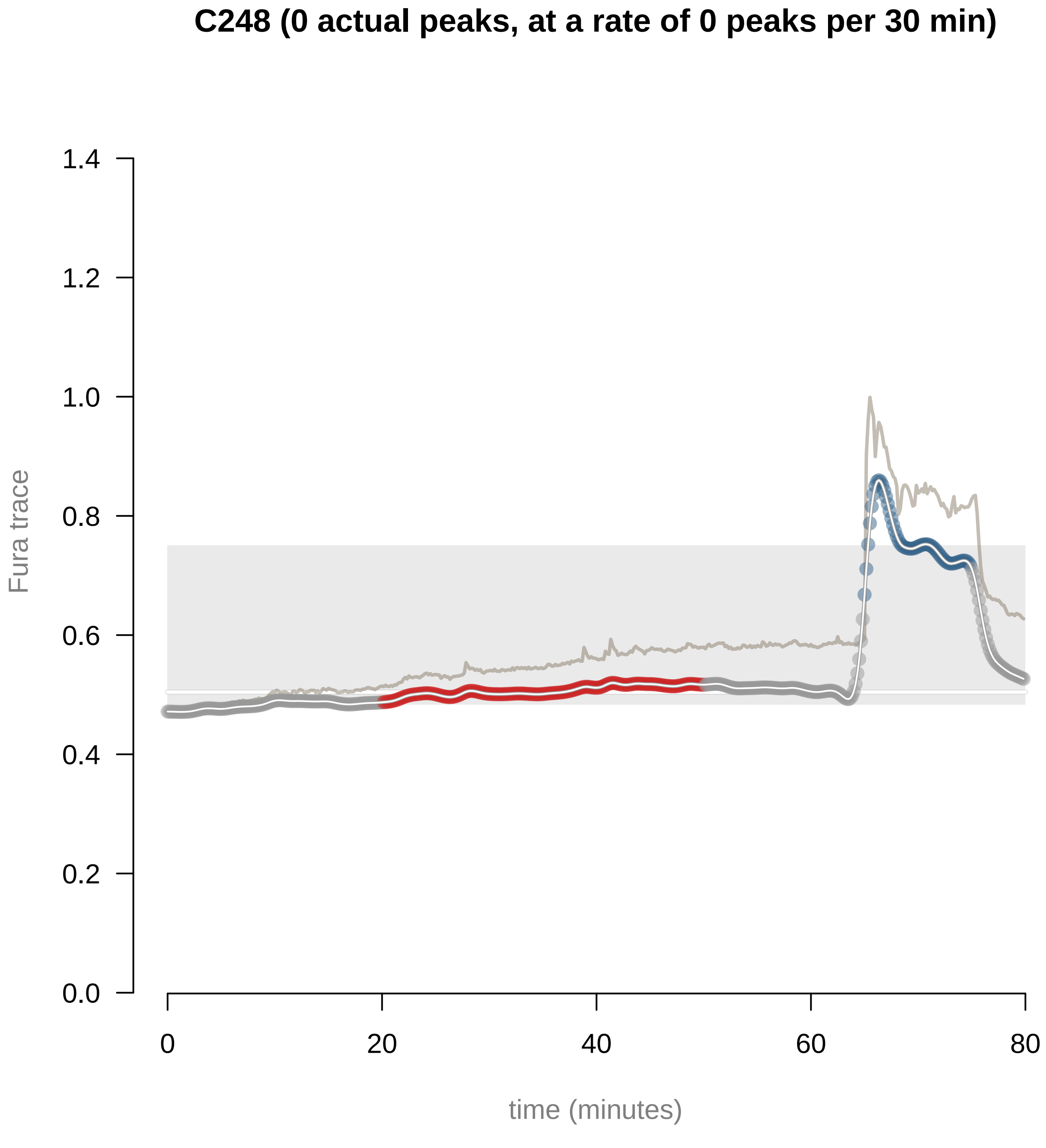
C248 (0 actual peaks, at a rate of 0 peaks per 30 min)
1.4
1.2
1.0
0.8
0.6
0.4
0.2
0.0
Fura trace
0
20
40
60
80
time (minutes)

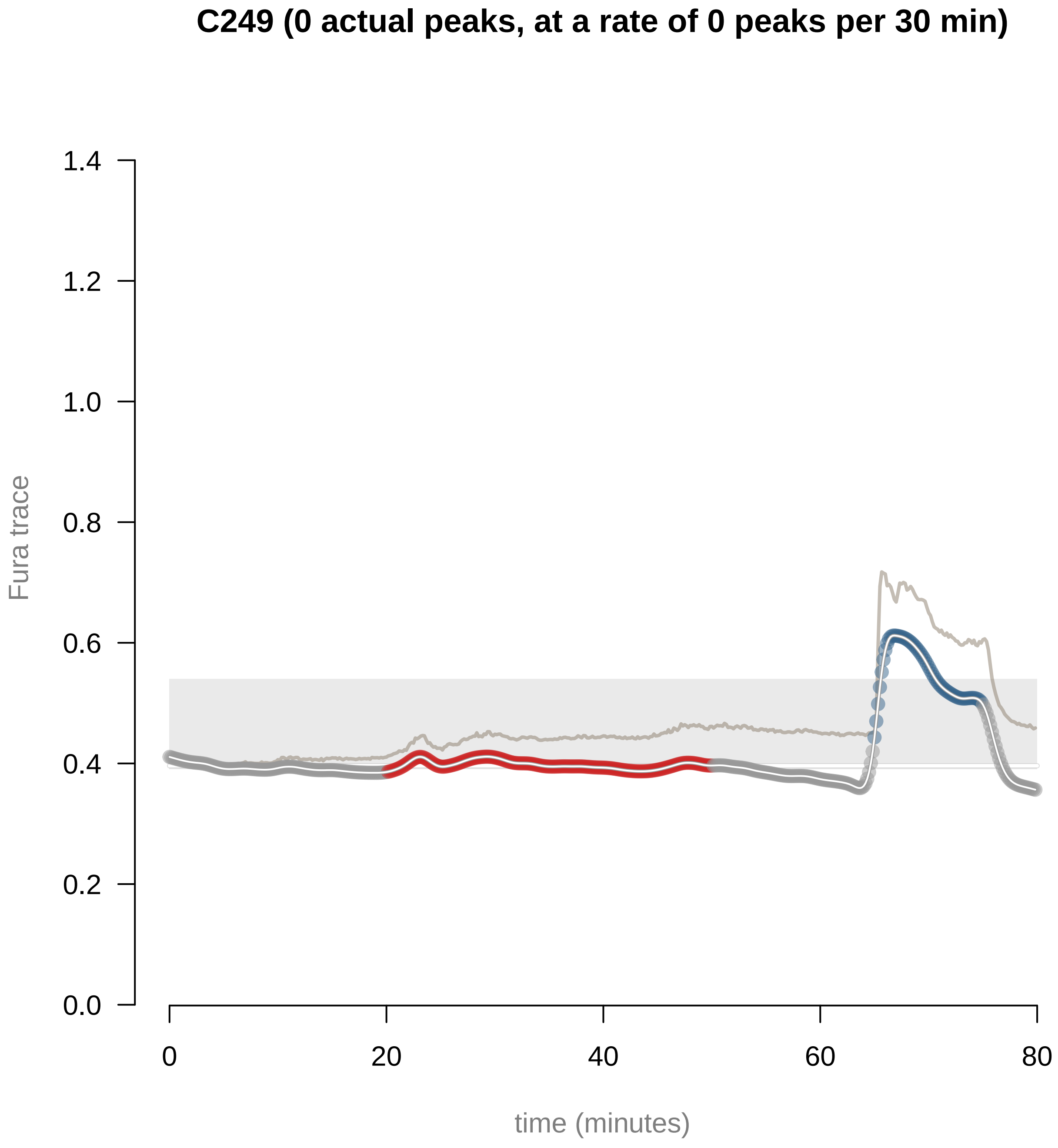

C249 (0 actual peaks, at a rate of 0 peaks per 30 min)
Fura trace
time (minutes)
0.0
0.2
0.4
0.6
0.8
1.0
1.2
1.4
0
20
40
60
80

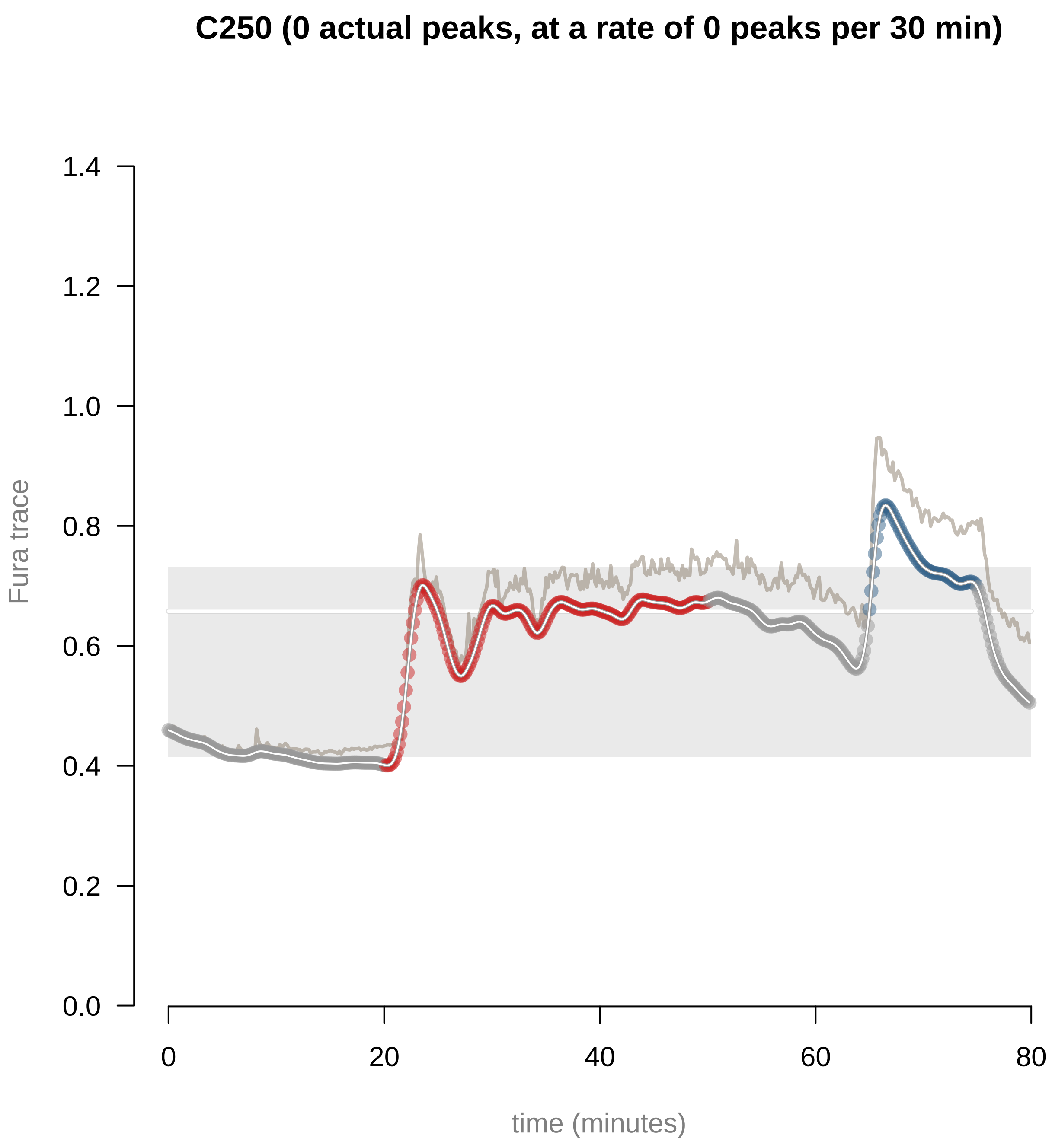
C250 (0 actual peaks, at a rate of 0 peaks per 30 min)
Fura trace
0.0
0.2
0.4
0.6
0.8
1.0
1.2
1.4
0
20
40
60
80
time (minutes)

C251 (3 actual peaks, at a rate of 3.67 peaks per 30 min)

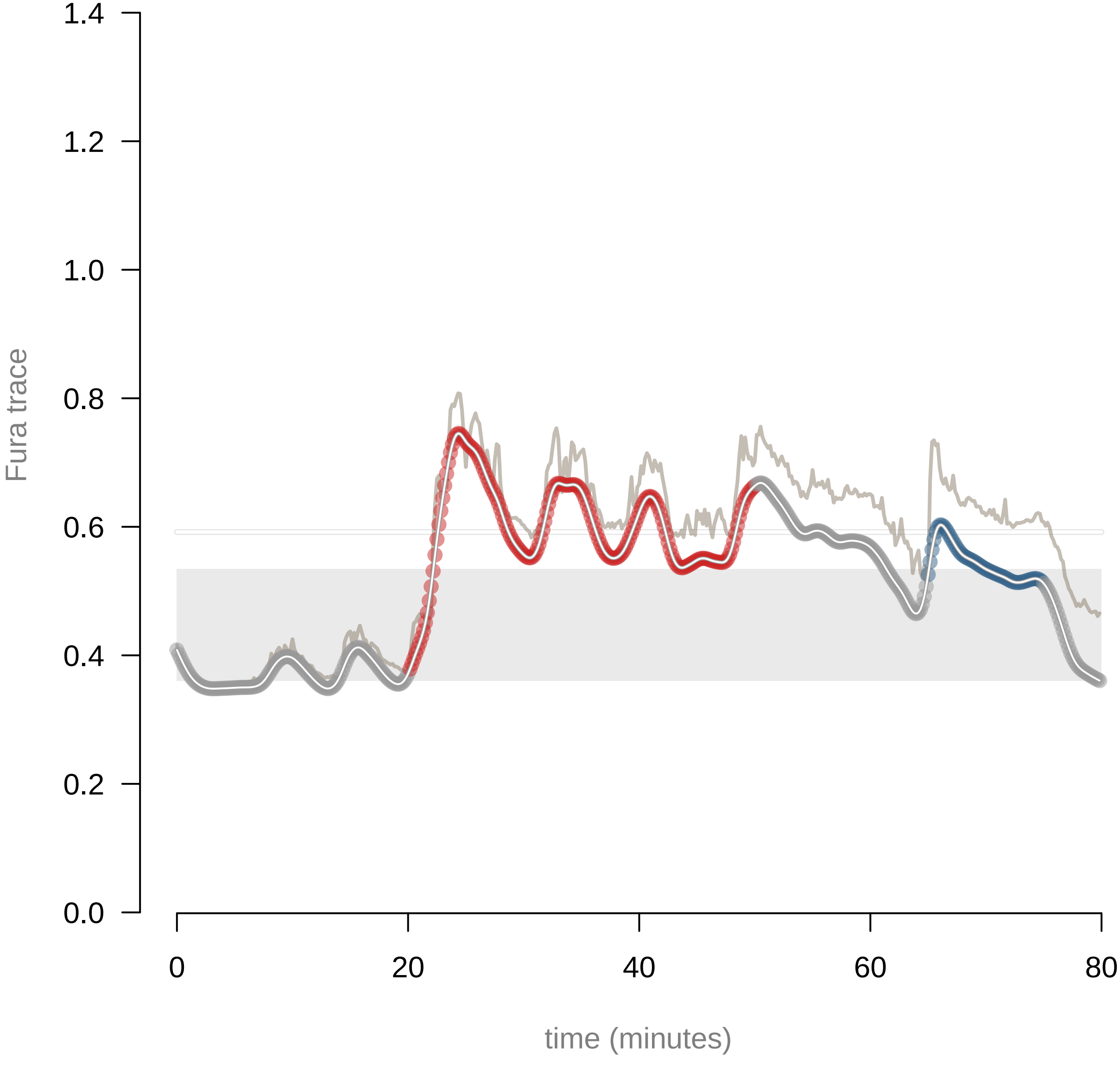

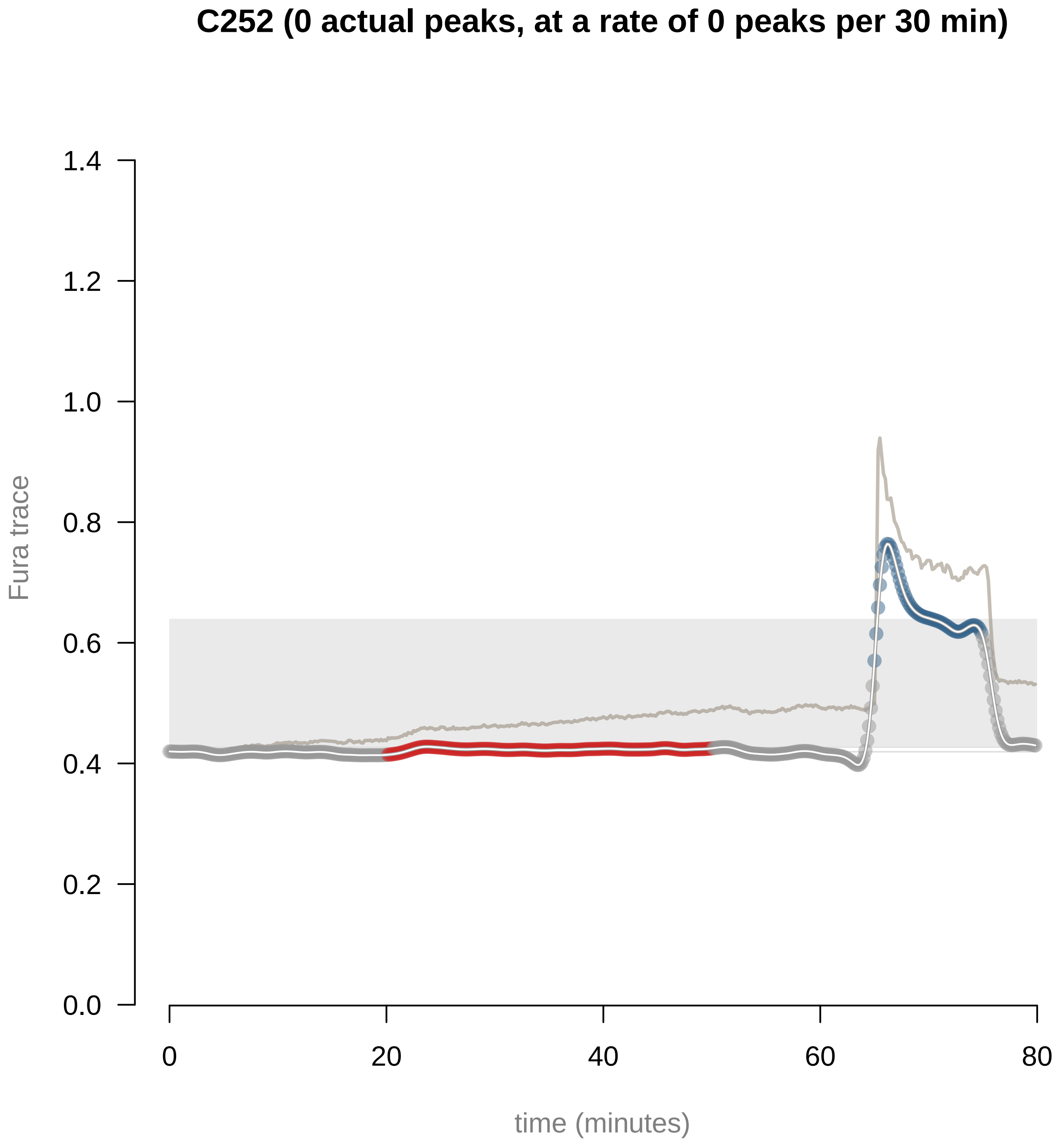
C252 (0 actual peaks, at a rate of 0 peaks per 30 min)
Fura trace
1.4
1.2
1.0
0.8
0.6
0.4
0.2
0.0
0
20
40
60
80
time (minutes)

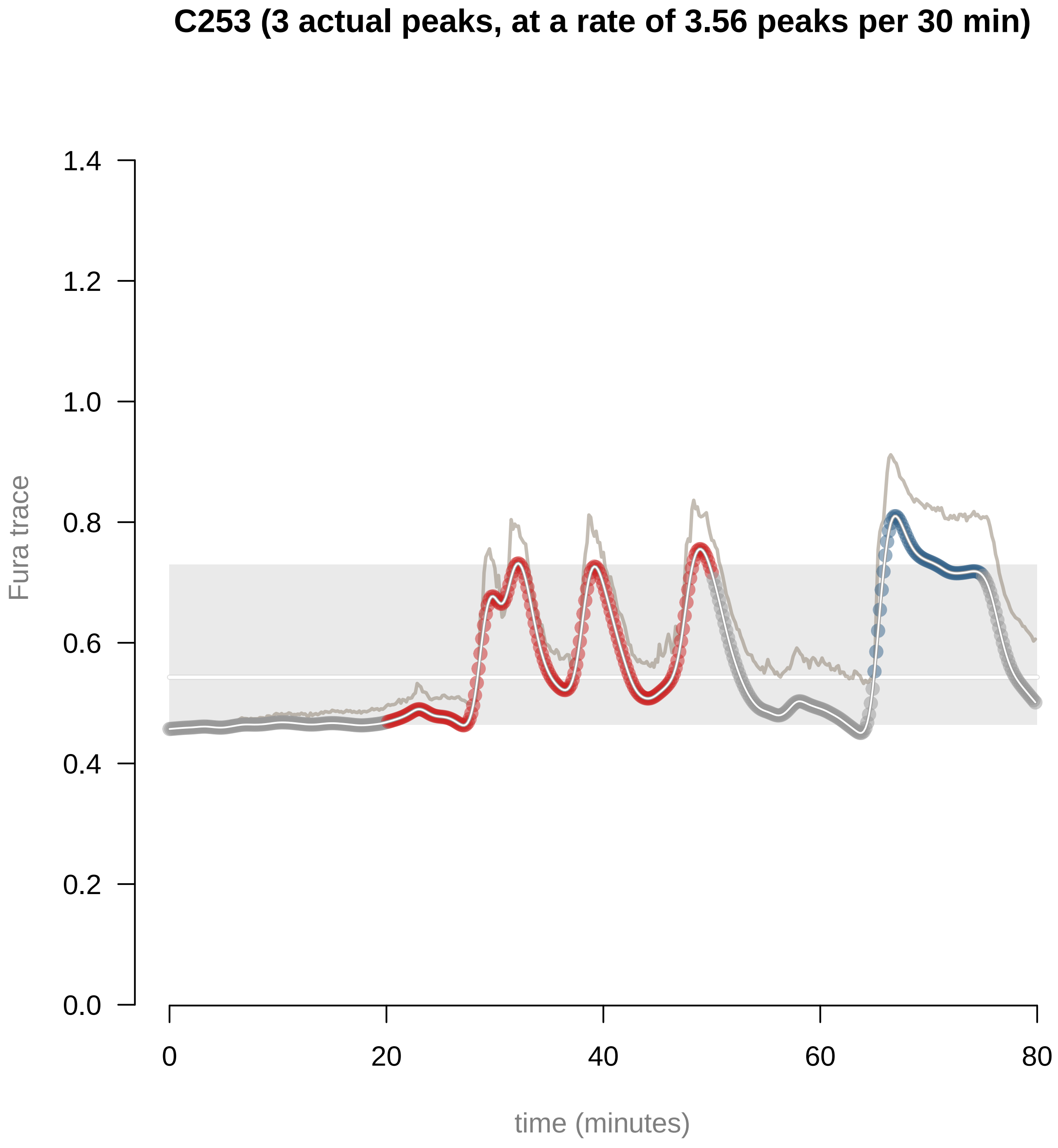
C253 (3 actual peaks, at a rate of 3.56 peaks per 30 min)
Fura trace
0.0
0.2
0.4
0.6
0.8
1.0
1.2
1.4
0
20
40
60
80
time (minutes)

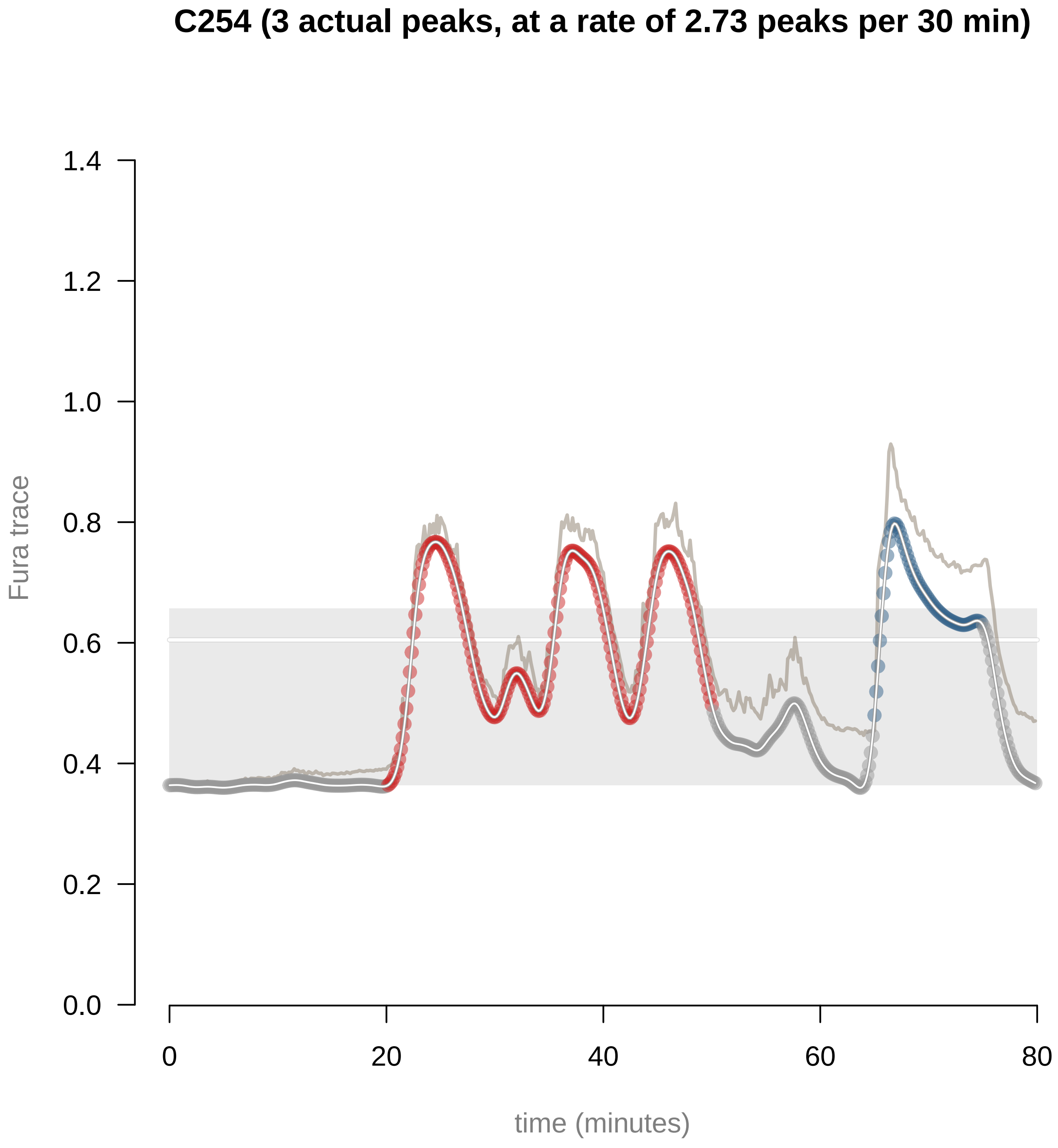

C254 (3 actual peaks, at a rate of 2.73 peaks per 30 min)
Fura trace
1.4
1.2
1.0
0.8
0.6
0.4
0.2
0.0
0
20
40
60
80
time (minutes)

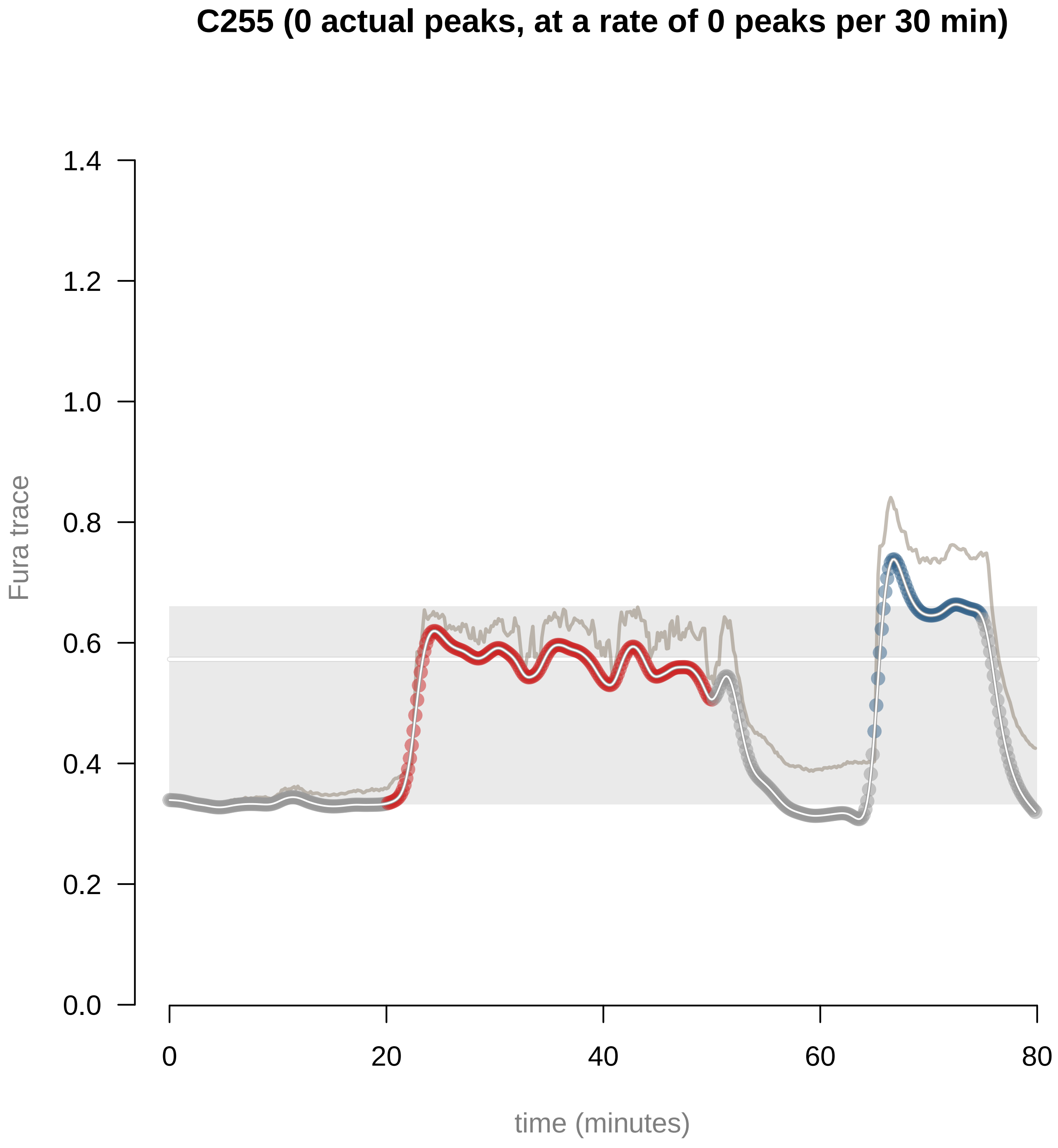

C255 (0 actual peaks, at a rate of 0 peaks per 30 min)
Fura trace
0.0
0.2
0.4
0.6
0.8
1.0
1.2
1.4
0
20
40
60
80
time (minutes)

C256 (4 actual peaks, at a rate of 3.46 peaks per 30 min)

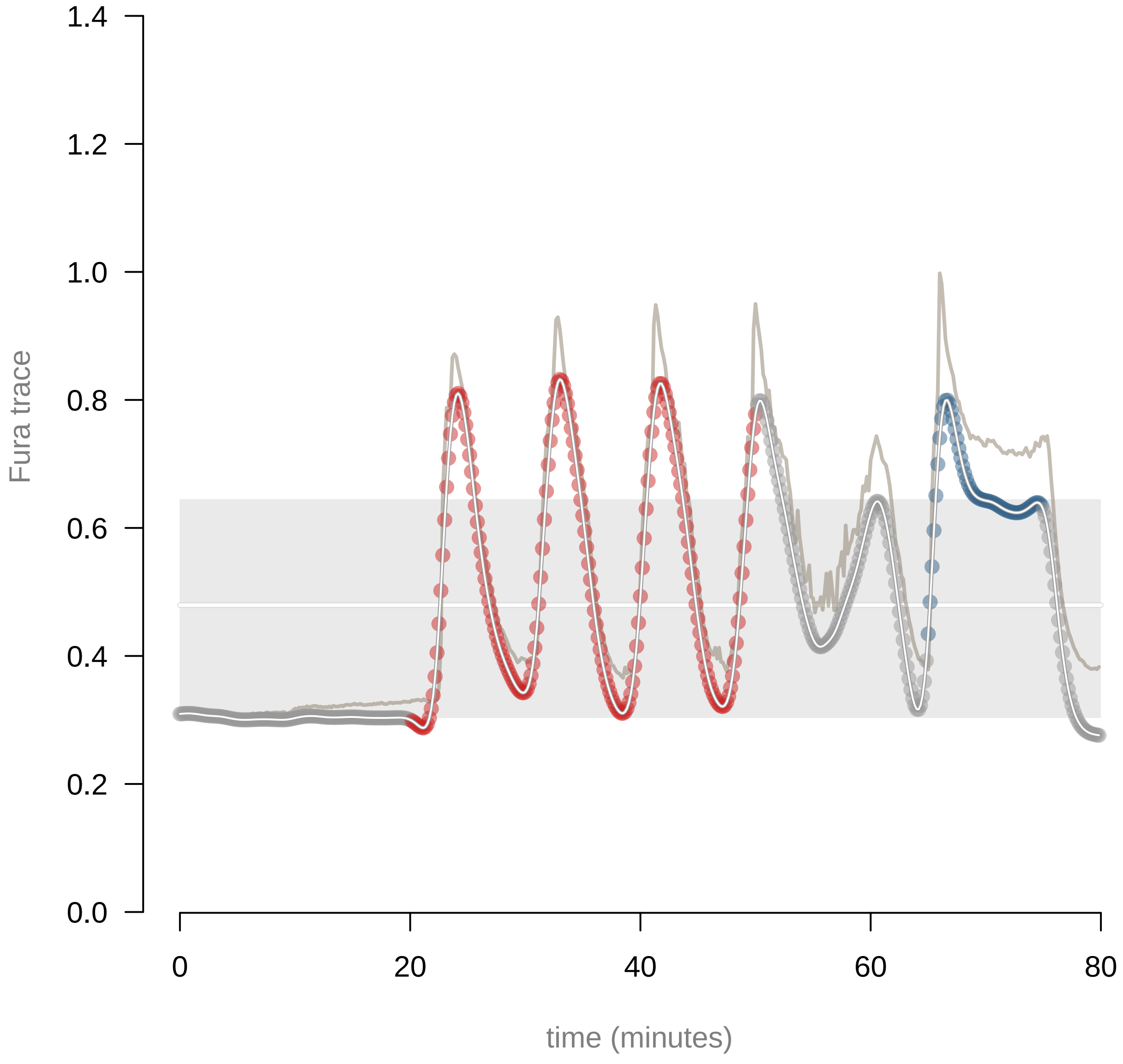

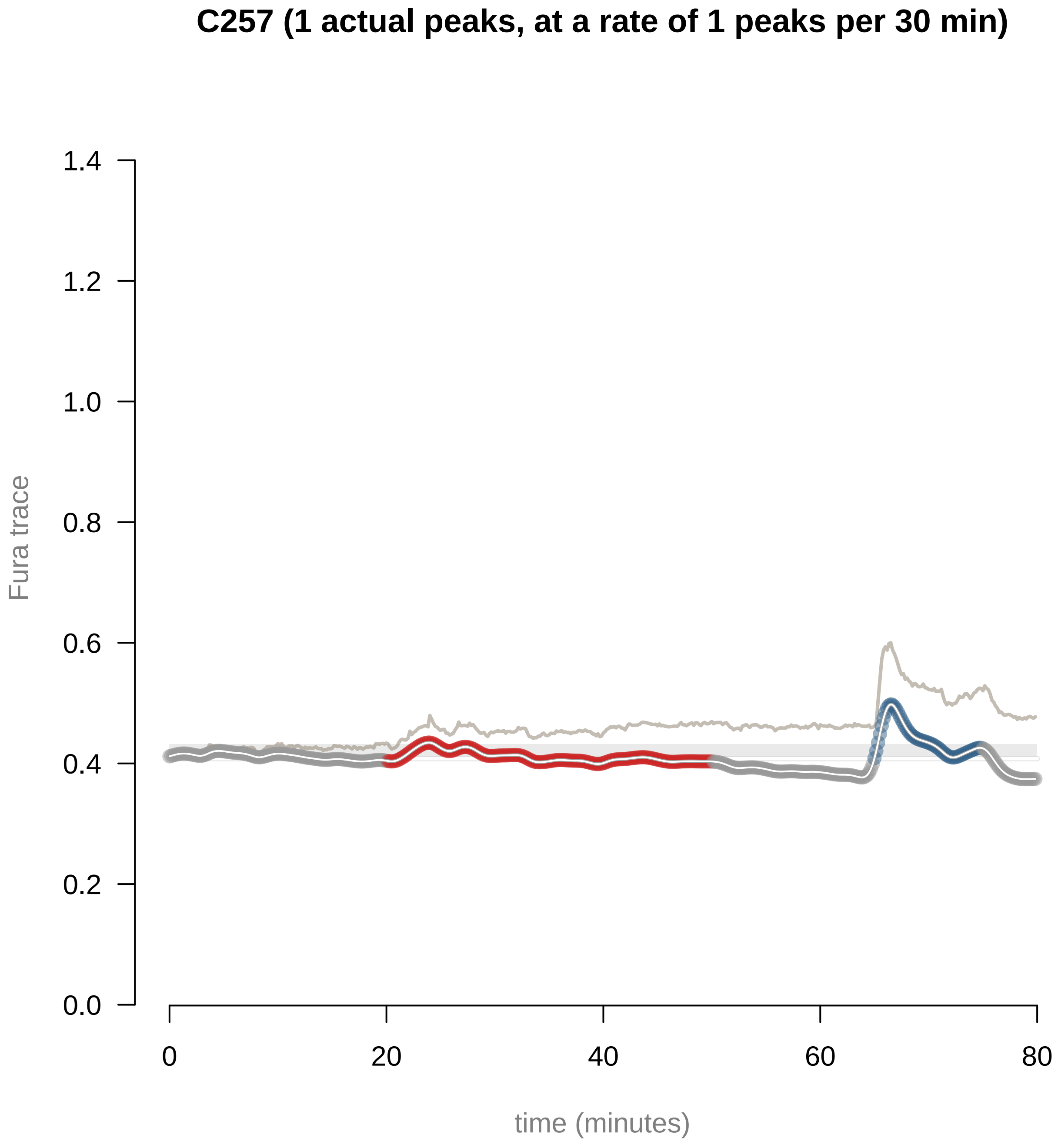
C257 (1 actual peaks, at a rate of 1 peaks per 30 min)
Fura trace
1.4
1.2
1.0
0.8
0.6
0.4
0.2
0.0
0
20
40
60
80
time (minutes)

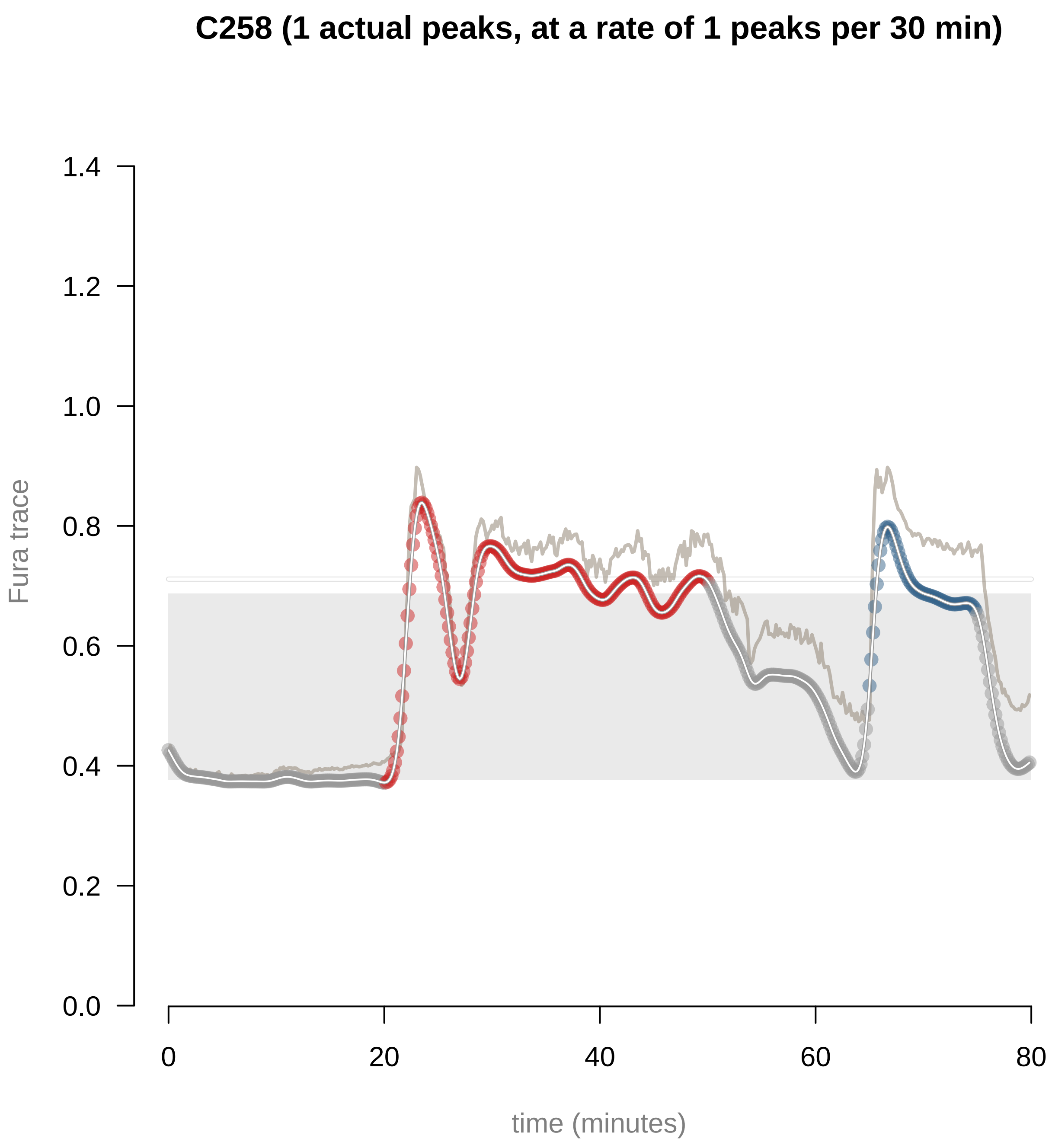

C258 (1 actual peaks, at a rate of 1 peaks per 30 min)
Fura trace
time (minutes)
0.0
0.2
0.4
0.6
0.8
1.0
1.2
1.4
0
20
40
60
80

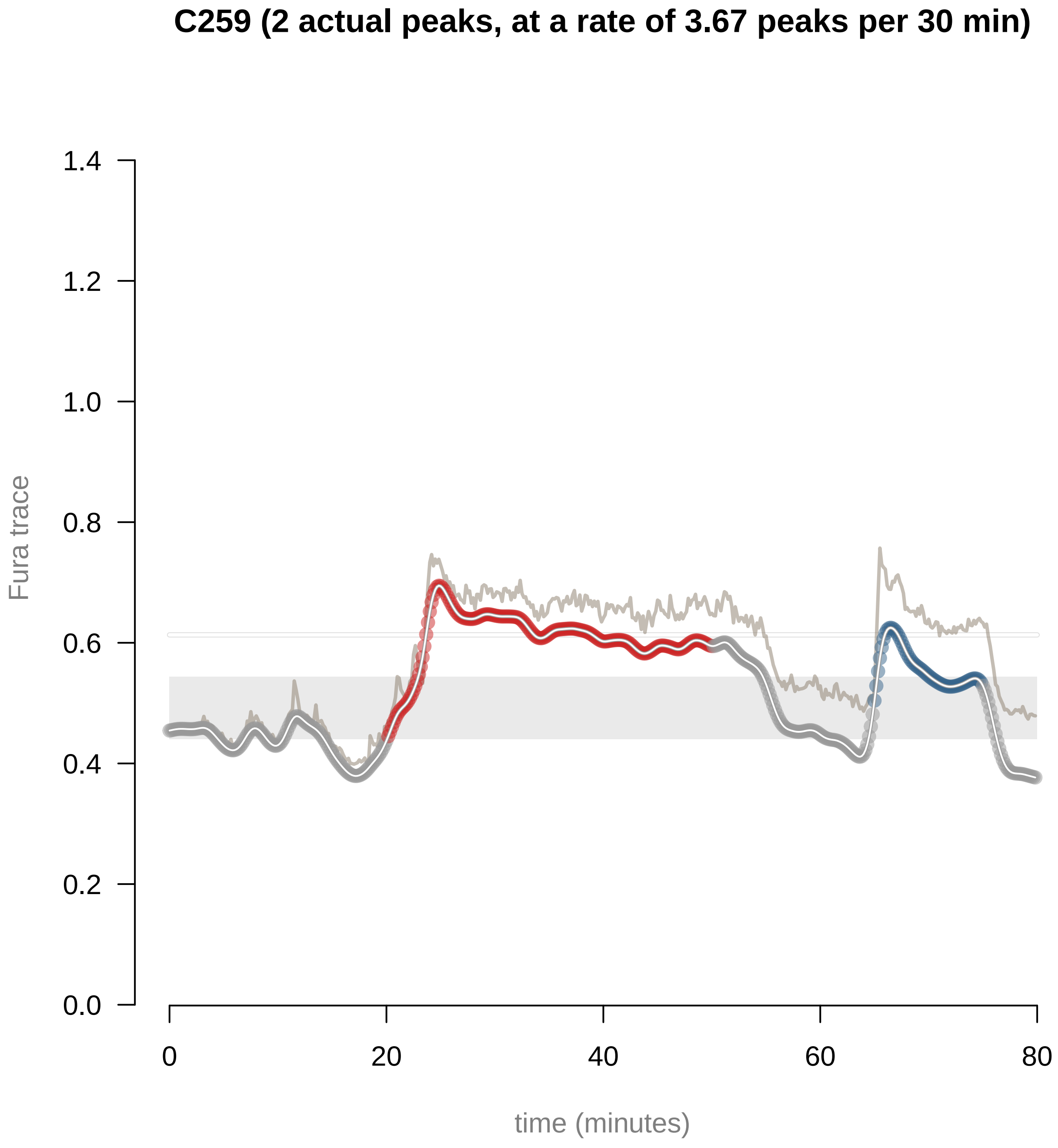
C259 (2 actual peaks, at a rate of 3.67 peaks per 30 min)
Fura trace
0.0
0.2
0.4
0.6
0.8
1.0
1.2
1.4
0
20
40
60
80
time (minutes)

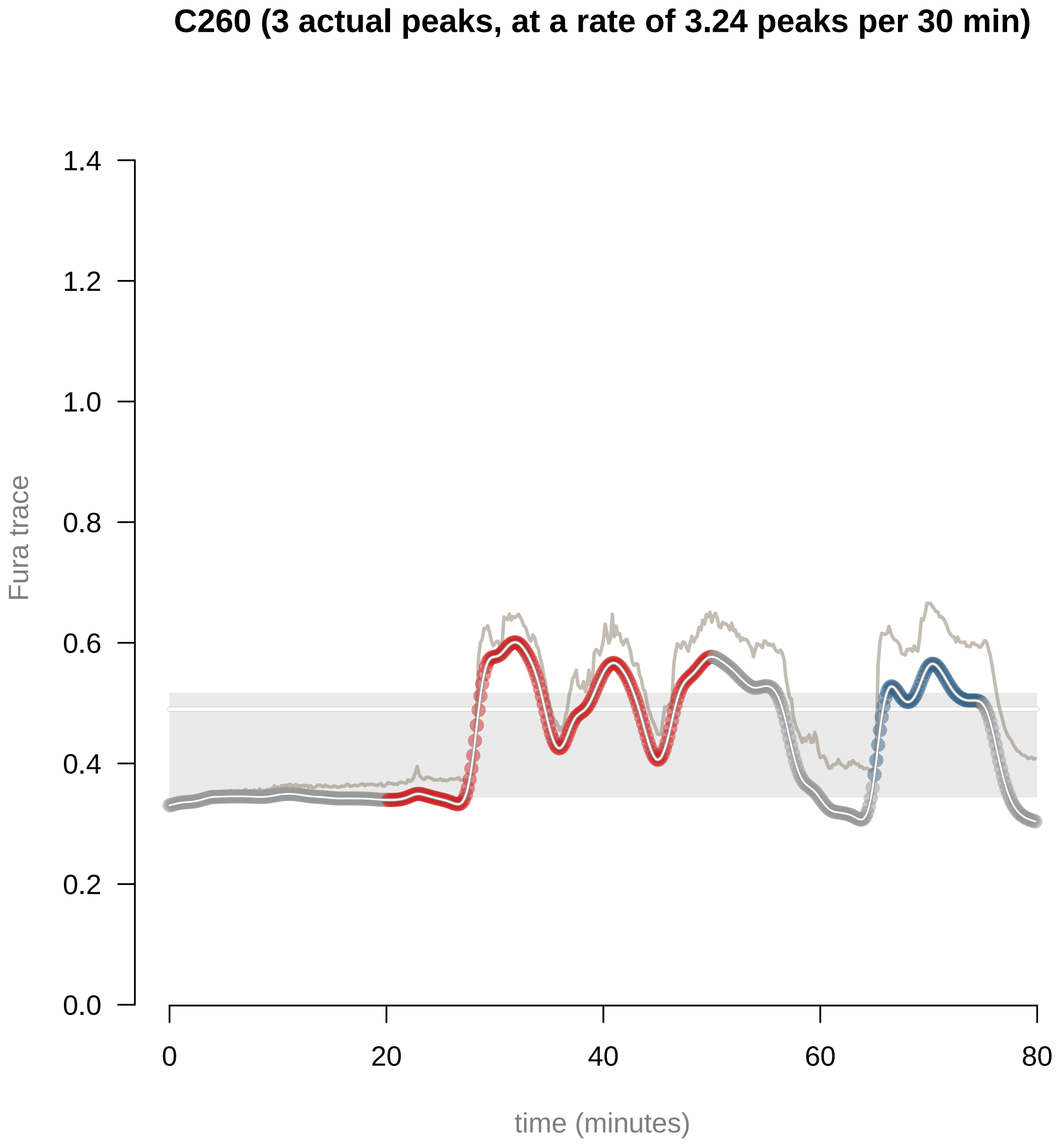

C260 (3 actual peaks, at a rate of 3.24 peaks per 30 min)
Fura trace
0.0
0.2
0.4
0.6
0.8
1.0
1.2
1.4
0
20
40
60
80
time (minutes)

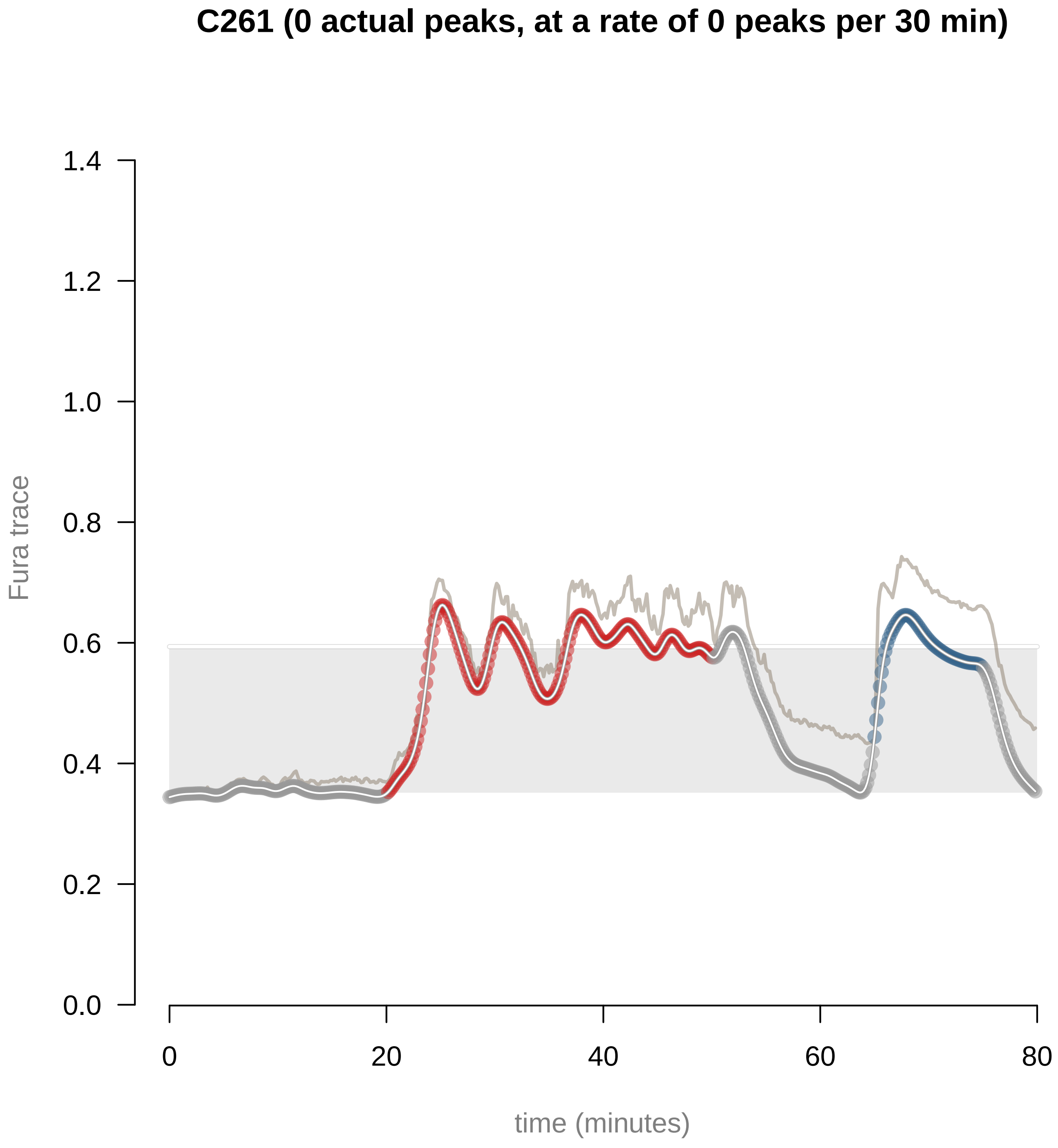
C261 (0 actual peaks, at a rate of 0 peaks per 30 min)
Fura trace
1.4
1.2
1.0
0.8
0.6
0.4
0.2
0.0
0
20
40
60
80
time (minutes)

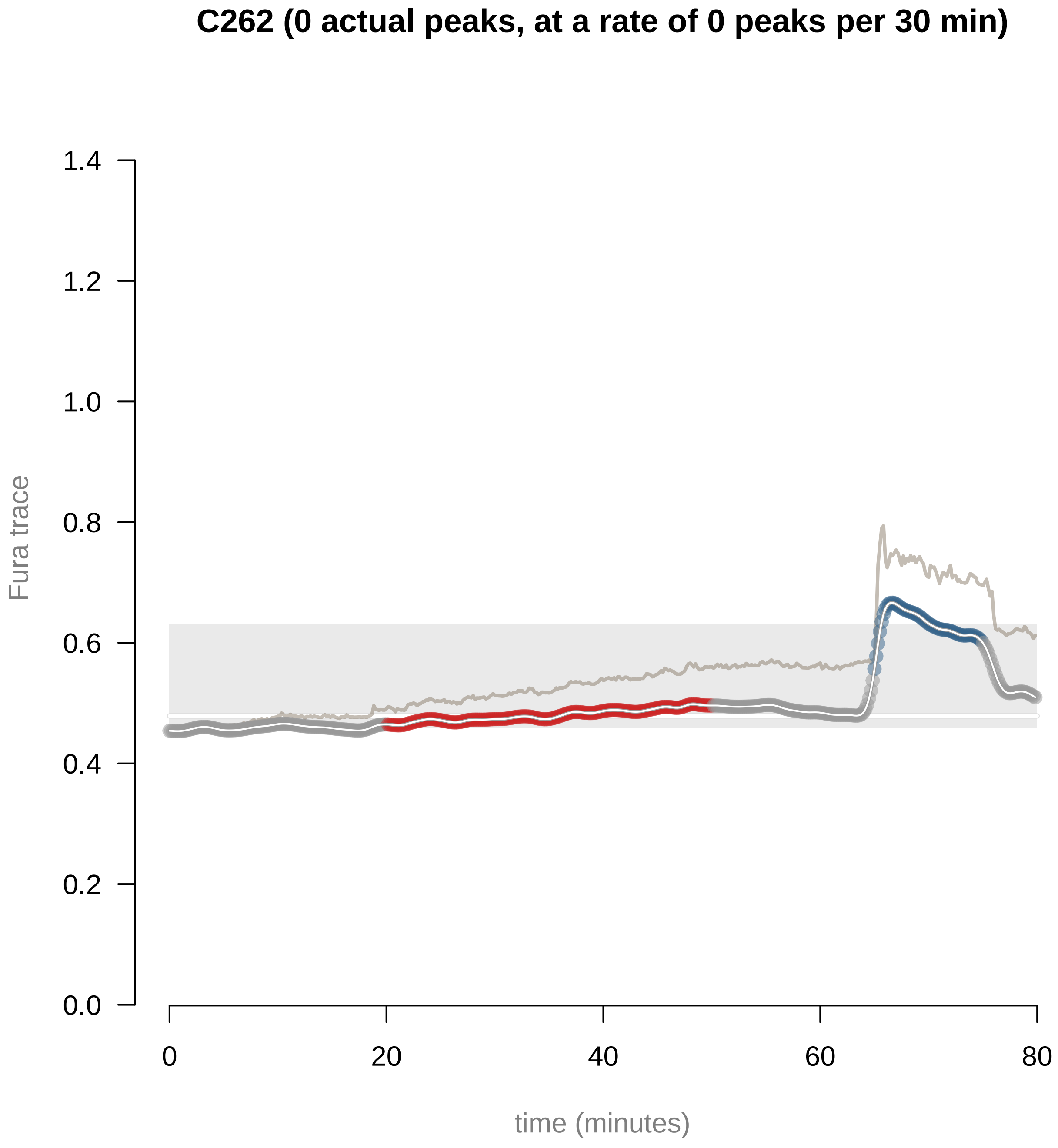
C262 (0 actual peaks, at a rate of 0 peaks per 30 min)
Fura trace
0.0
0.2
0.4
0.6
0.8
1.0
1.2
1.4
0
20
40
60
80
time (minutes)

C263 (1 actual peaks, at a rate of 1 peaks per 30 min)

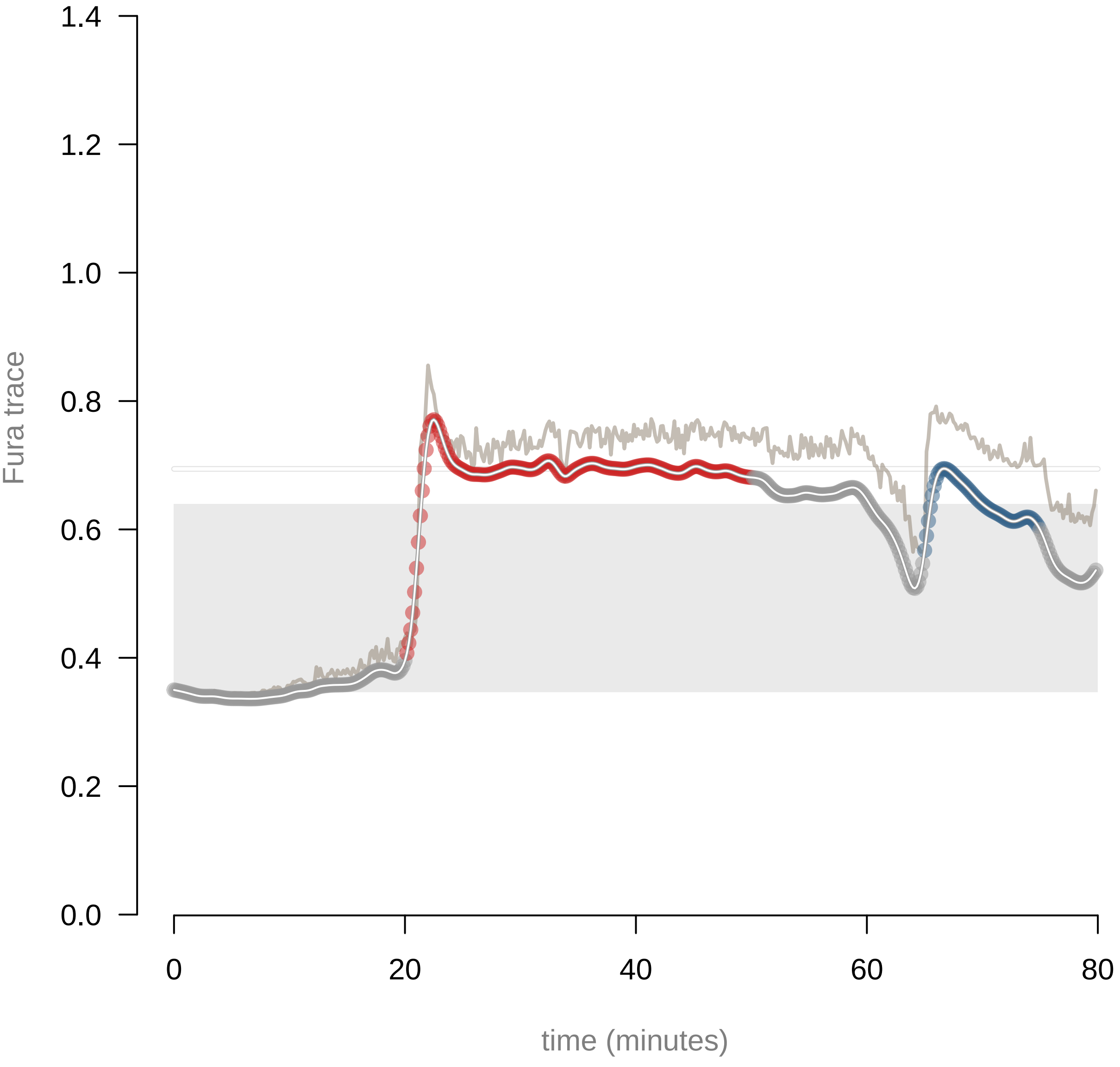

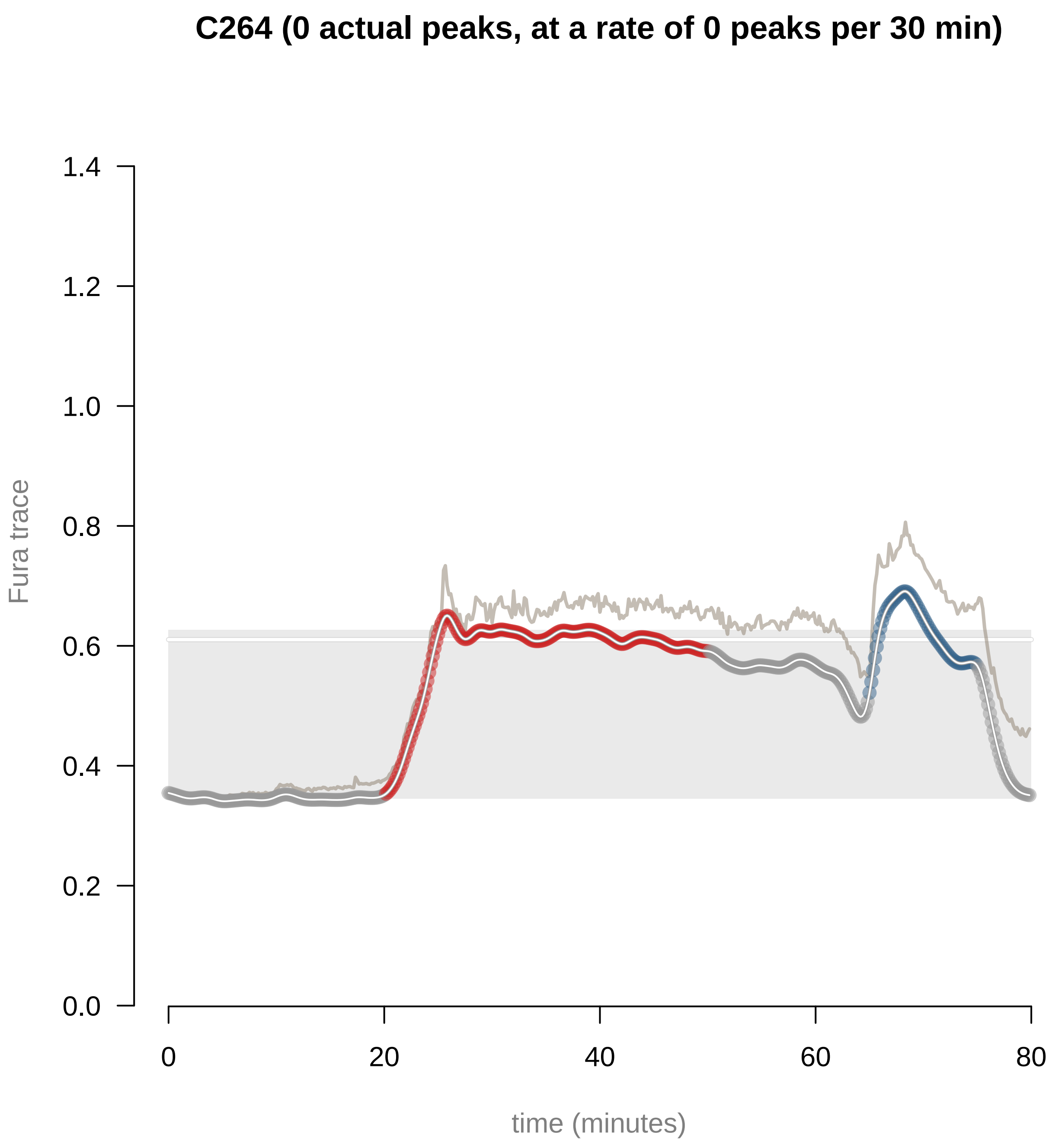
C264 (0 actual peaks, at a rate of 0 peaks per 30 min)
Fura trace
1.4
1.2
1.0
0.8
0.6
0.4
0.2
0.0
0
20
40
60
80
time (minutes)

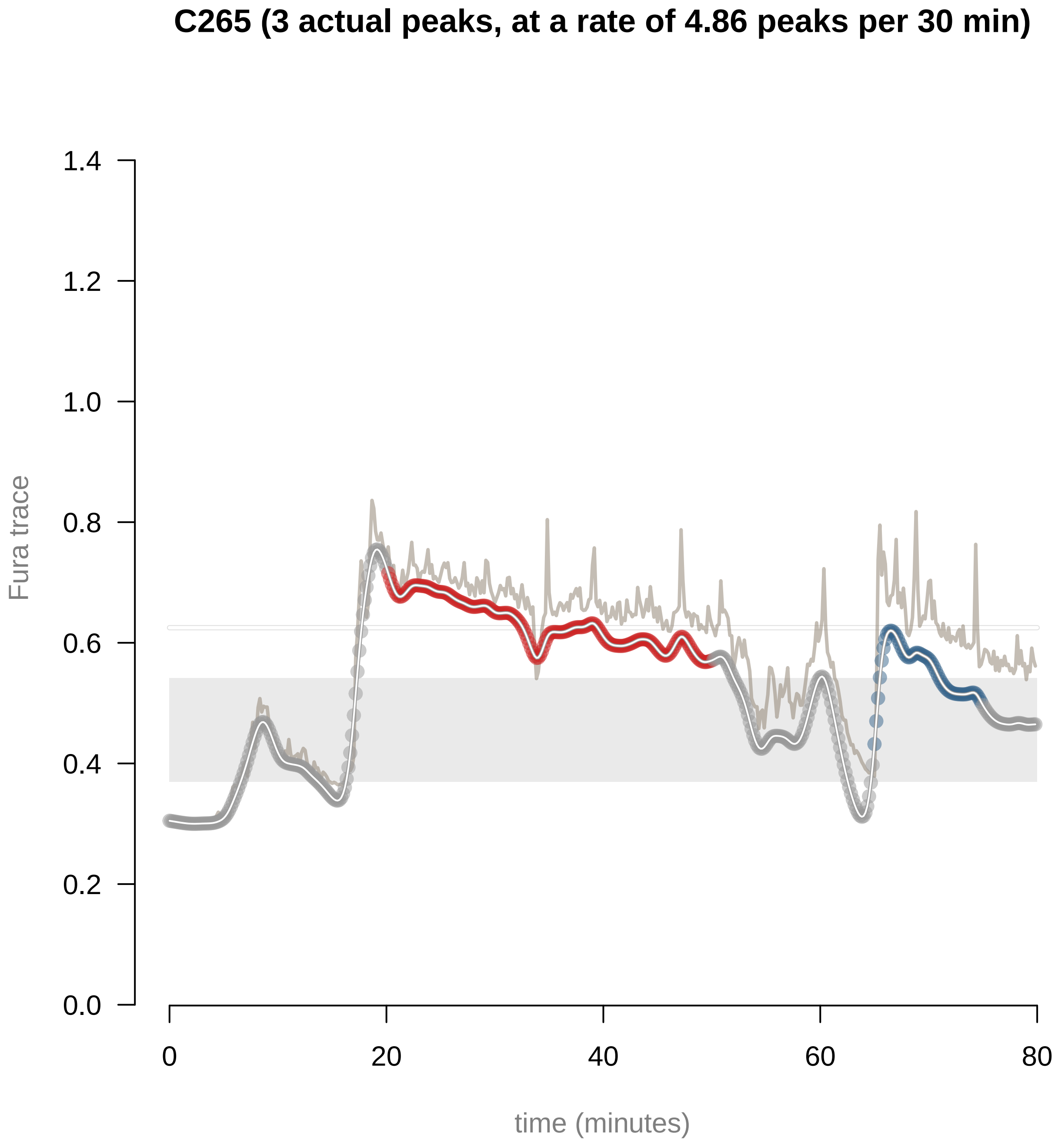

C265 (3 actual peaks, at a rate of 4.86 peaks per 30 min)
Fura trace
time (minutes)
0.0
0.2
0.4
0.6
0.8
1.0
1.2
1.4
0
20
40
60
80

C266 (0 actual peaks, at a rate of 0 peaks per 30 min)

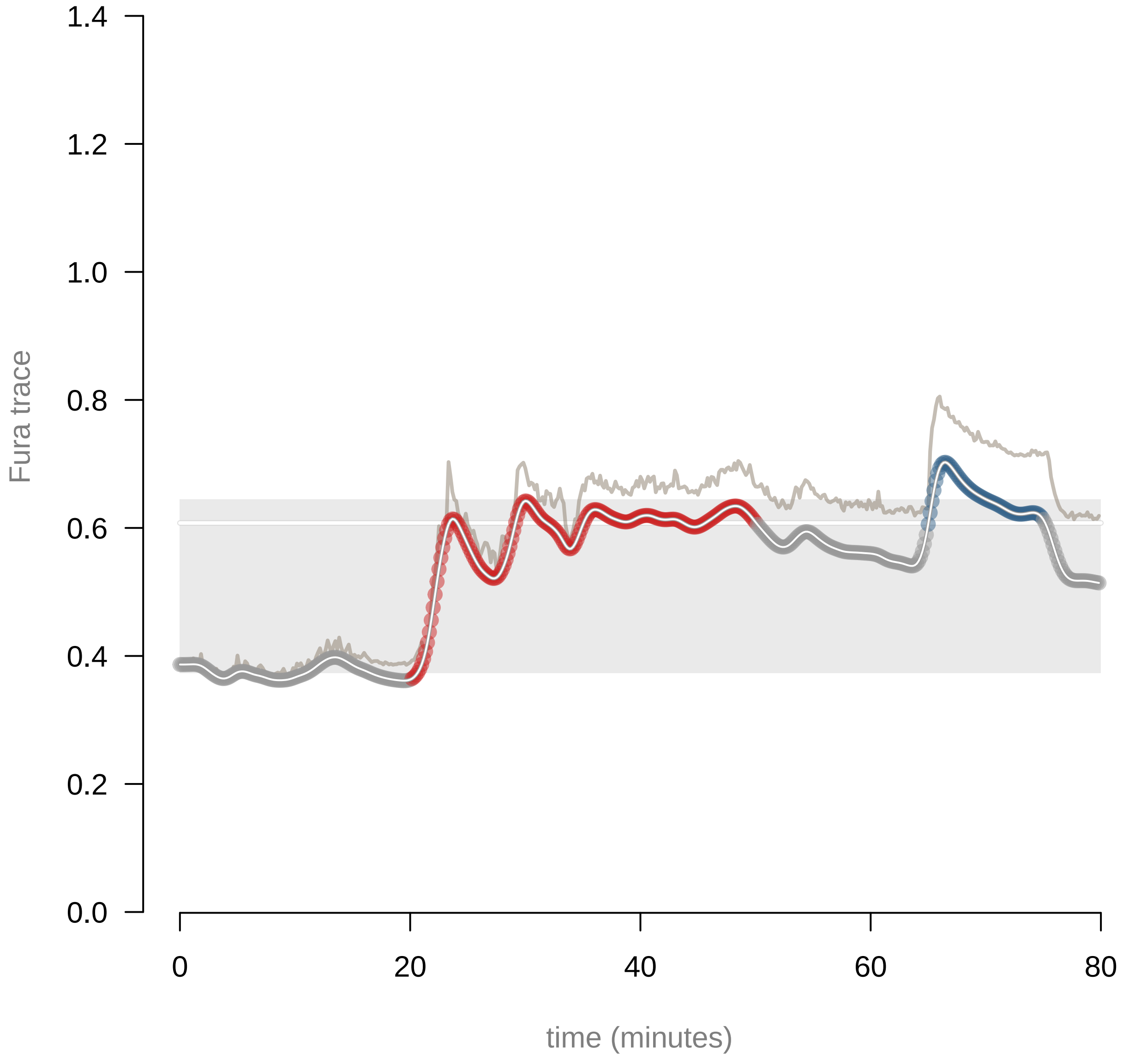

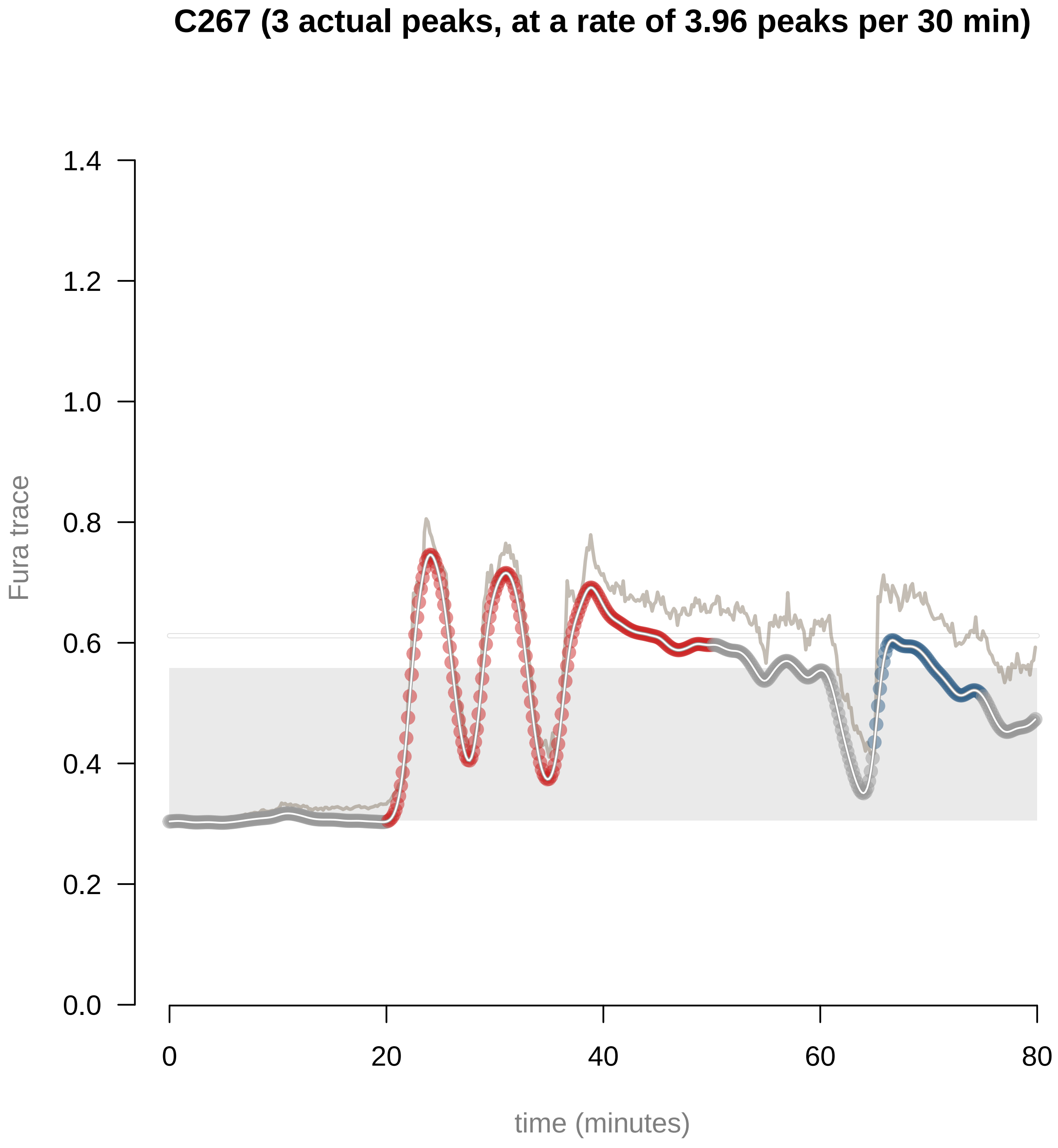
C267 (3 actual peaks, at a rate of 3.96 peaks per 30 min)
Fura trace
1.4
1.2
1.0
0.8
0.6
0.4
0.2
0.0
0
20
40
60
80
time (minutes)

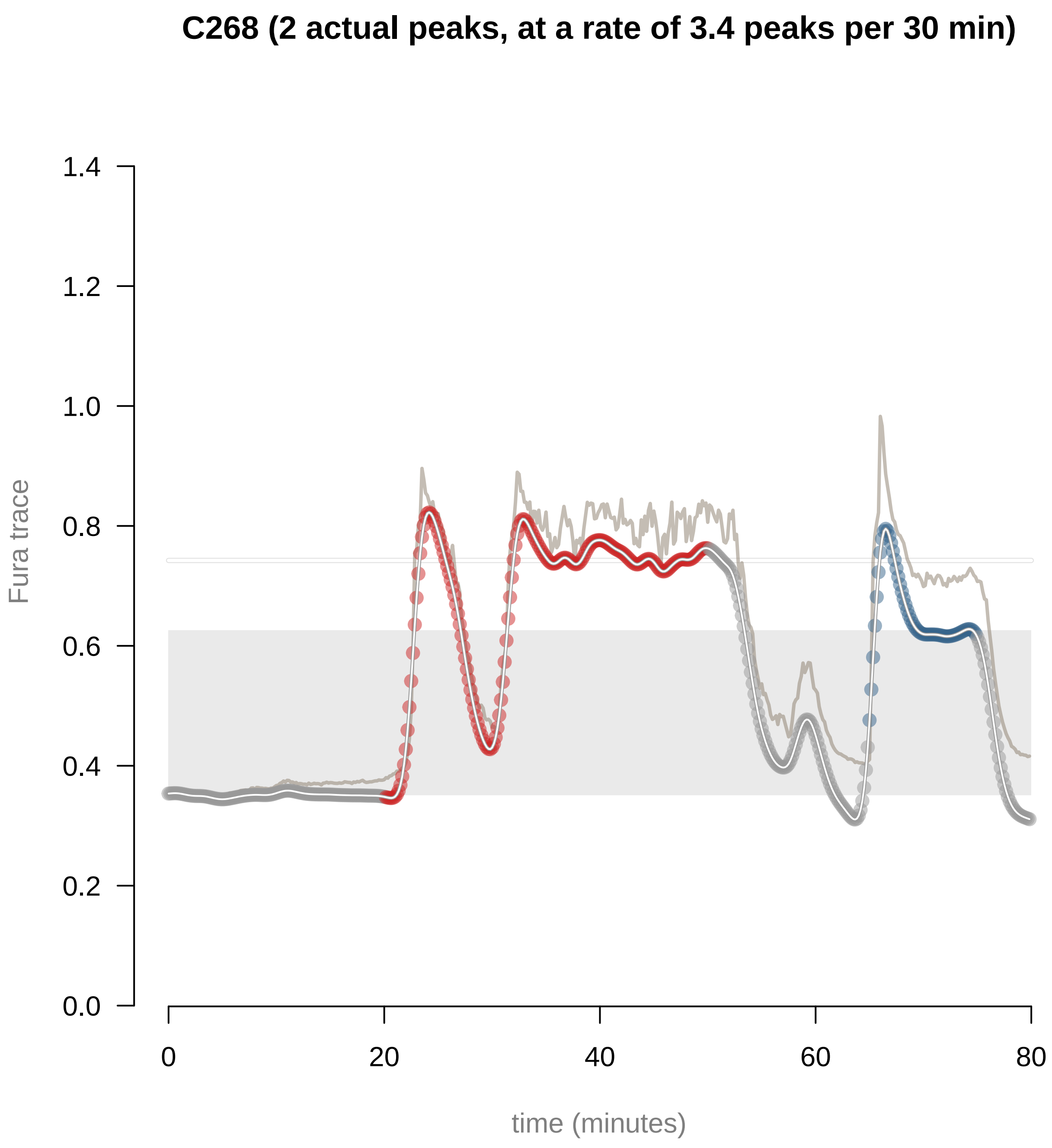

C268 (2 actual peaks, at a rate of 3.4 peaks per 30 min)
Fura trace
1.4
1.2
1.0
0.8
0.6
0.4
0.2
0.0
0
20
40
60
80
time (minutes)

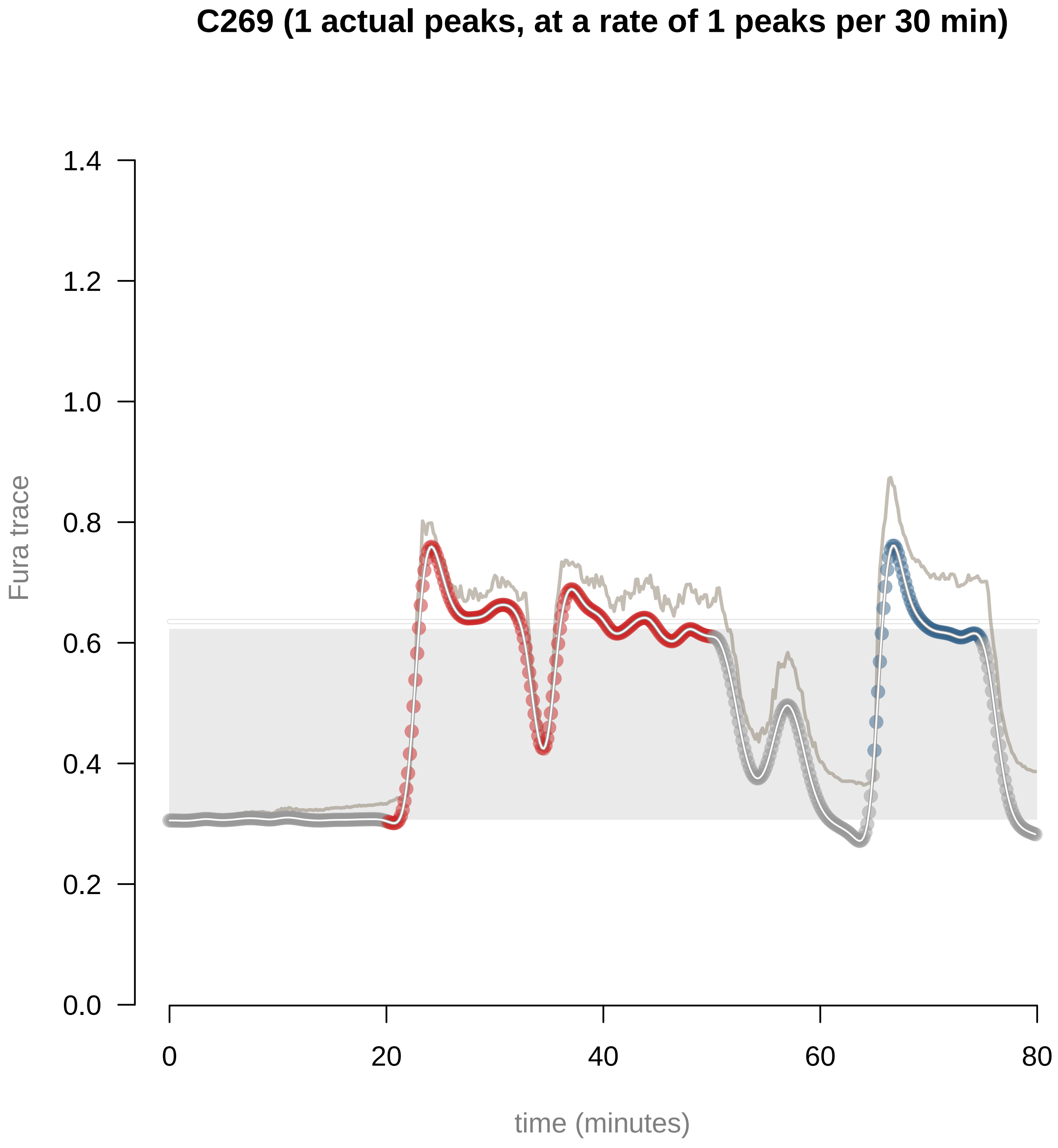
C269 (1 actual peaks, at a rate of 1 peaks per 30 min)
Fura trace
1.4
1.2
1.0
0.8
0.6
0.4
0.2
0.0
0
20
40
60
80
time (minutes)

C270 (3 actual peaks, at a rate of 3.16 peaks per 30 min)

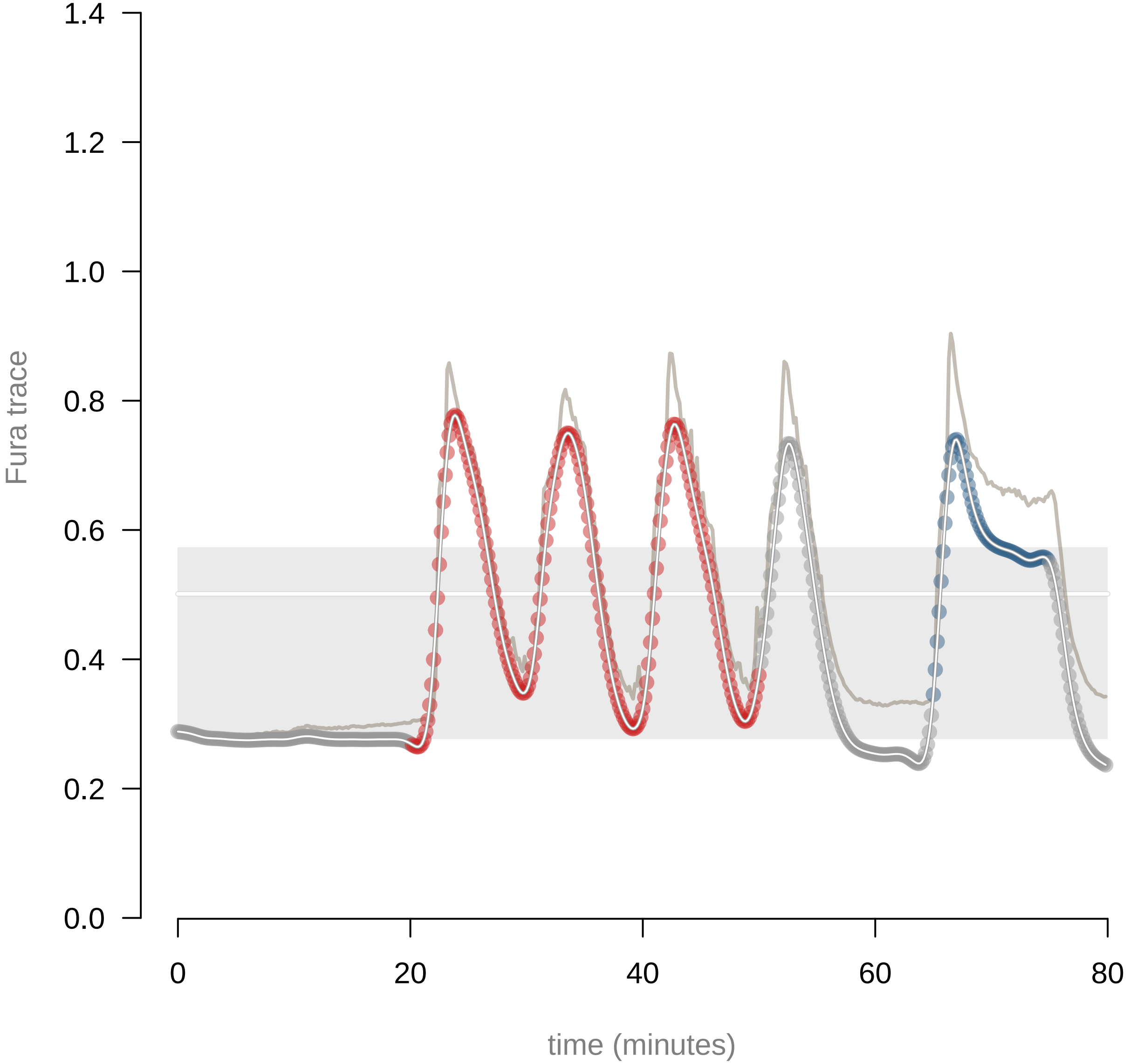

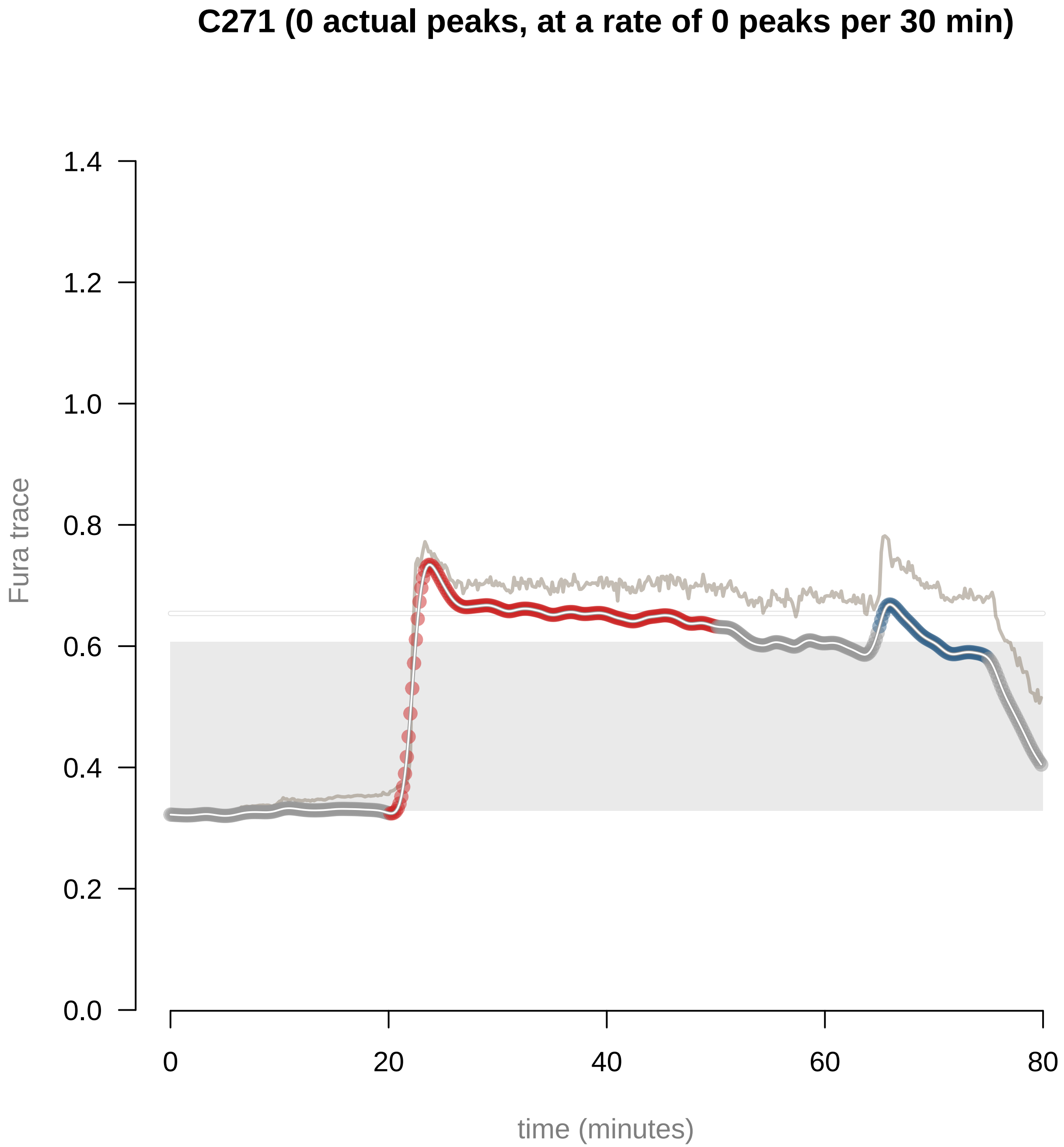
C271 (0 actual peaks, at a rate of 0 peaks per 30 min)
Fura trace
1.4
1.2
1.0
0.8
0.6
0.4
0.2
0.0
0
20
40
60
80
time (minutes)

C272 (0 actual peaks, at a rate of 0 peaks per 30 min)

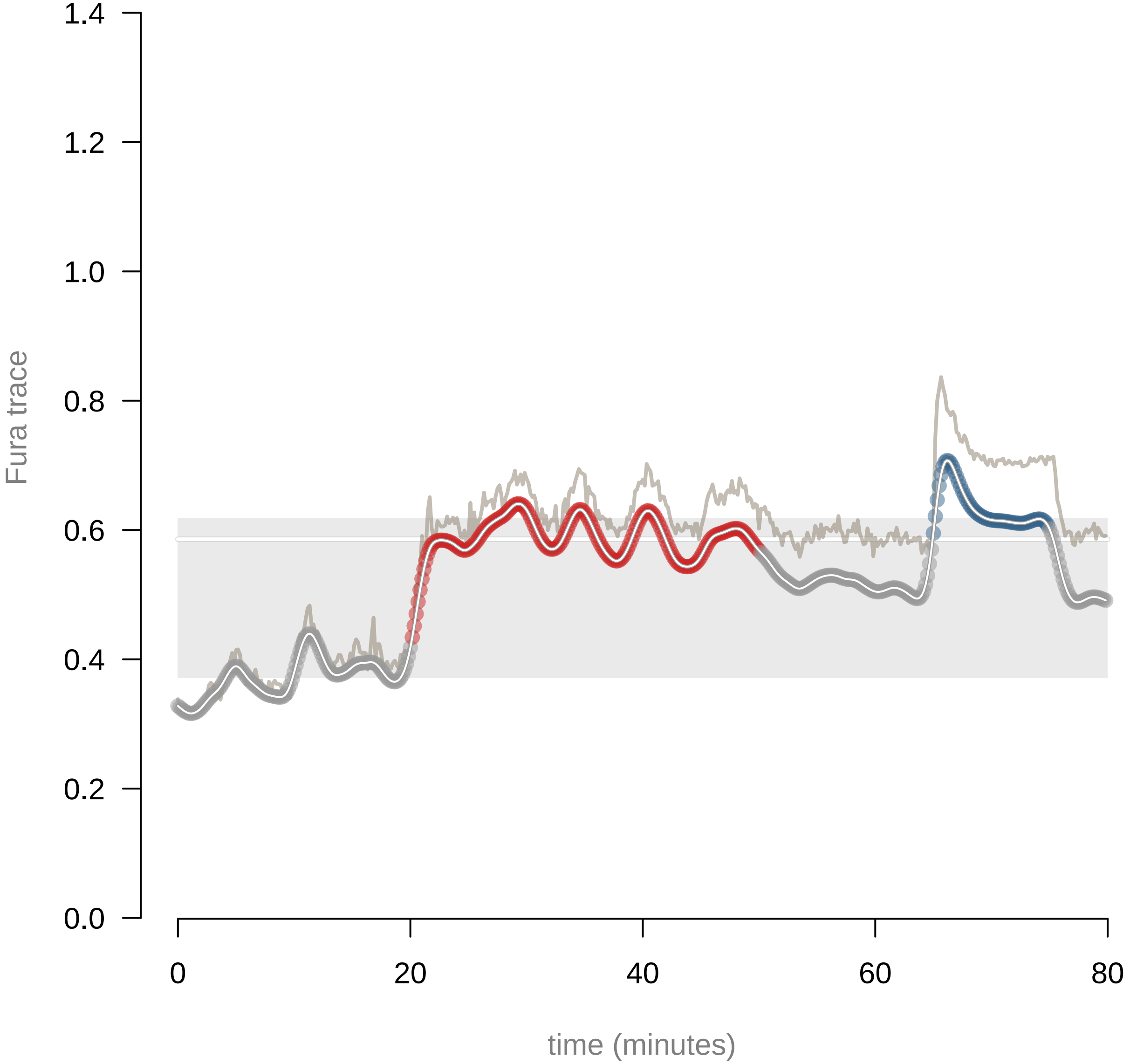

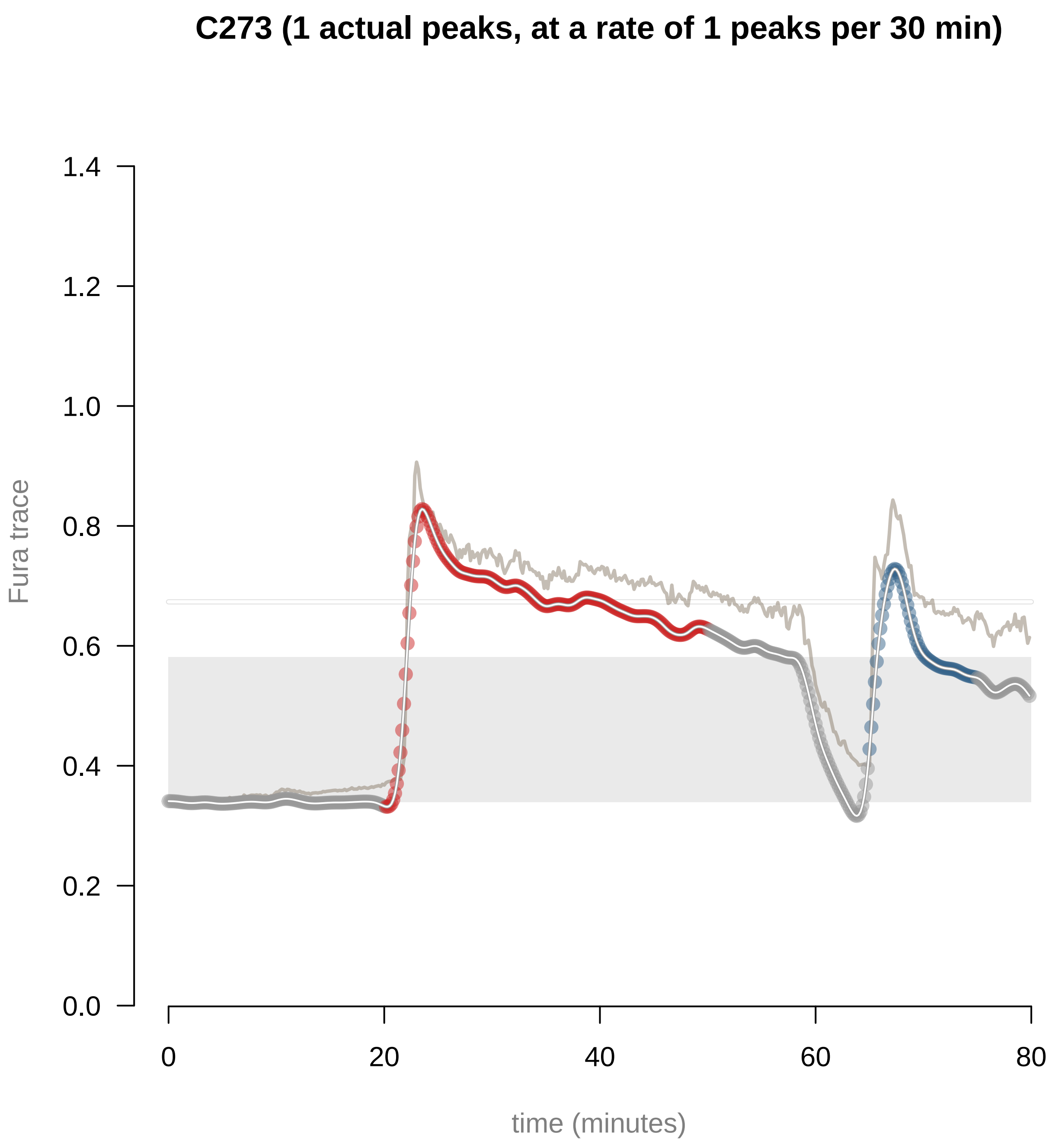

C273 (1 actual peaks, at a rate of 1 peaks per 30 min)
Fura trace
1.4
1.2
1.0
0.8
0.6
0.4
0.2
0.0
0
20
40
60
80
time (minutes)

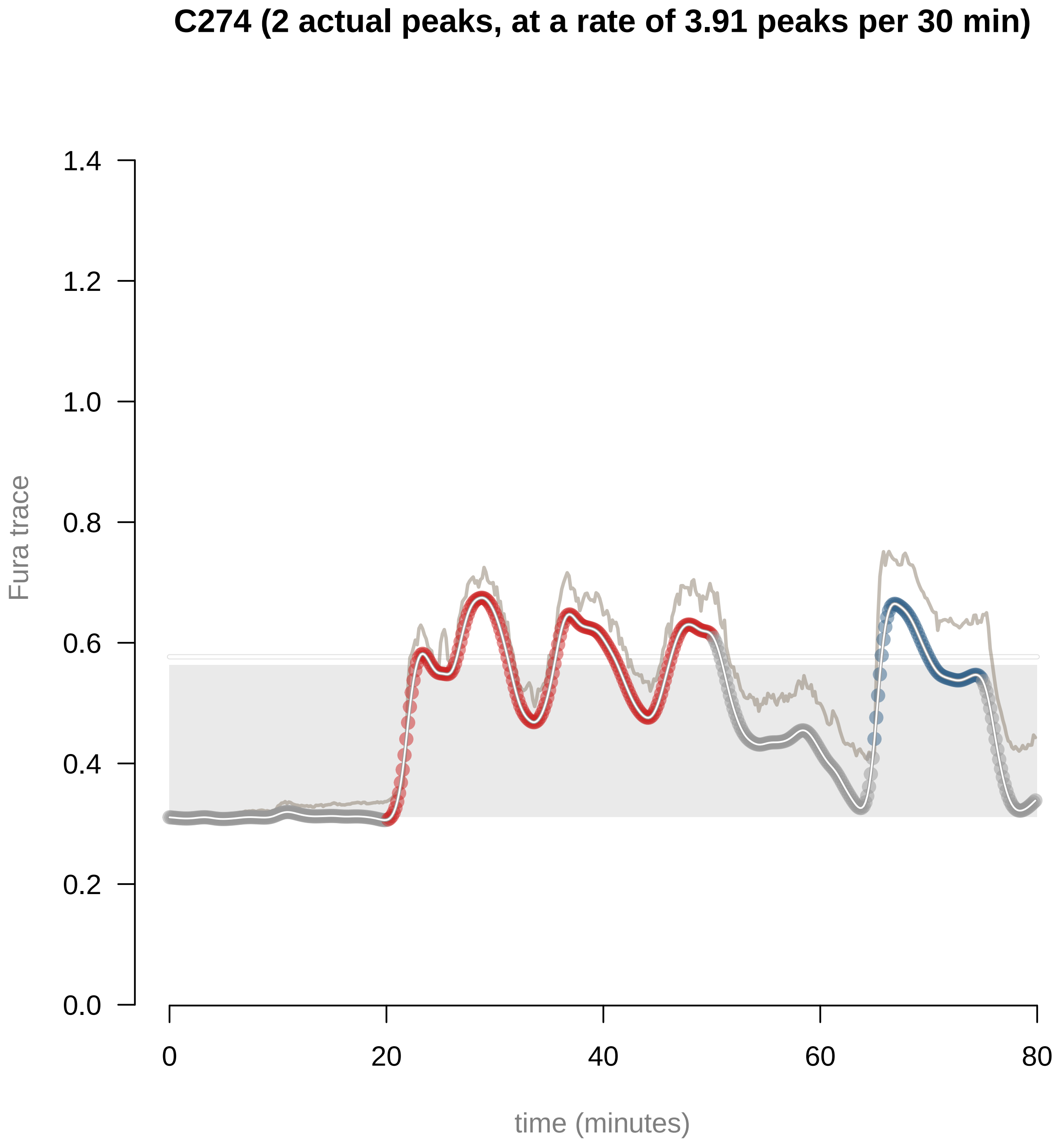
C274 (2 actual peaks, at a rate of 3.91 peaks per 30 min)
Fura trace
1.4
1.2
1.0
0.8
0.6
0.4
0.2
0.0
0
20
40
60
80
time (minutes)

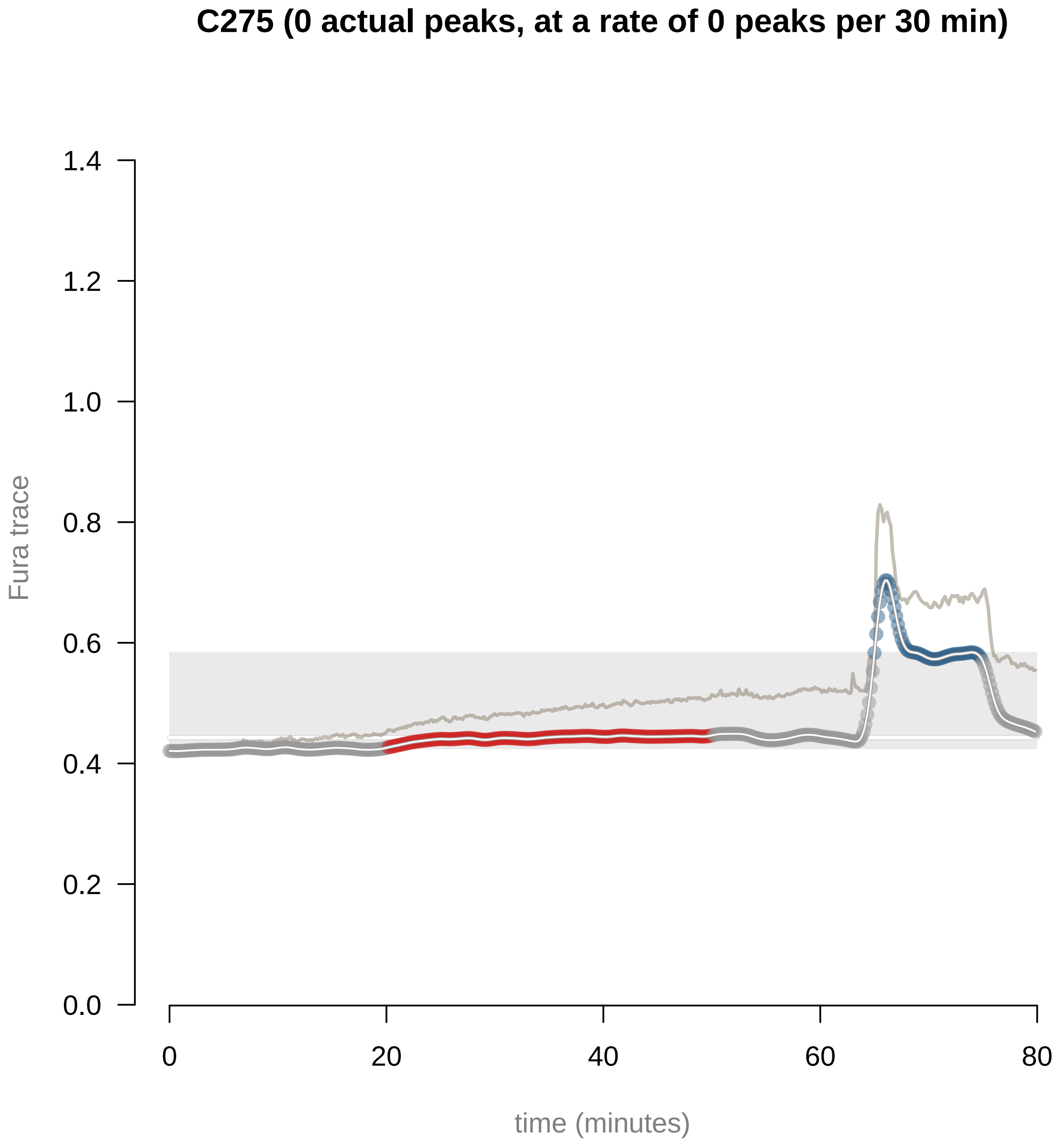
C275 (0 actual peaks, at a rate of 0 peaks per 30 min)
Fura trace
1.4
1.2
1.0
0.8
0.6
0.4
0.2
0.0
0
20
40
60
80
time (minutes)

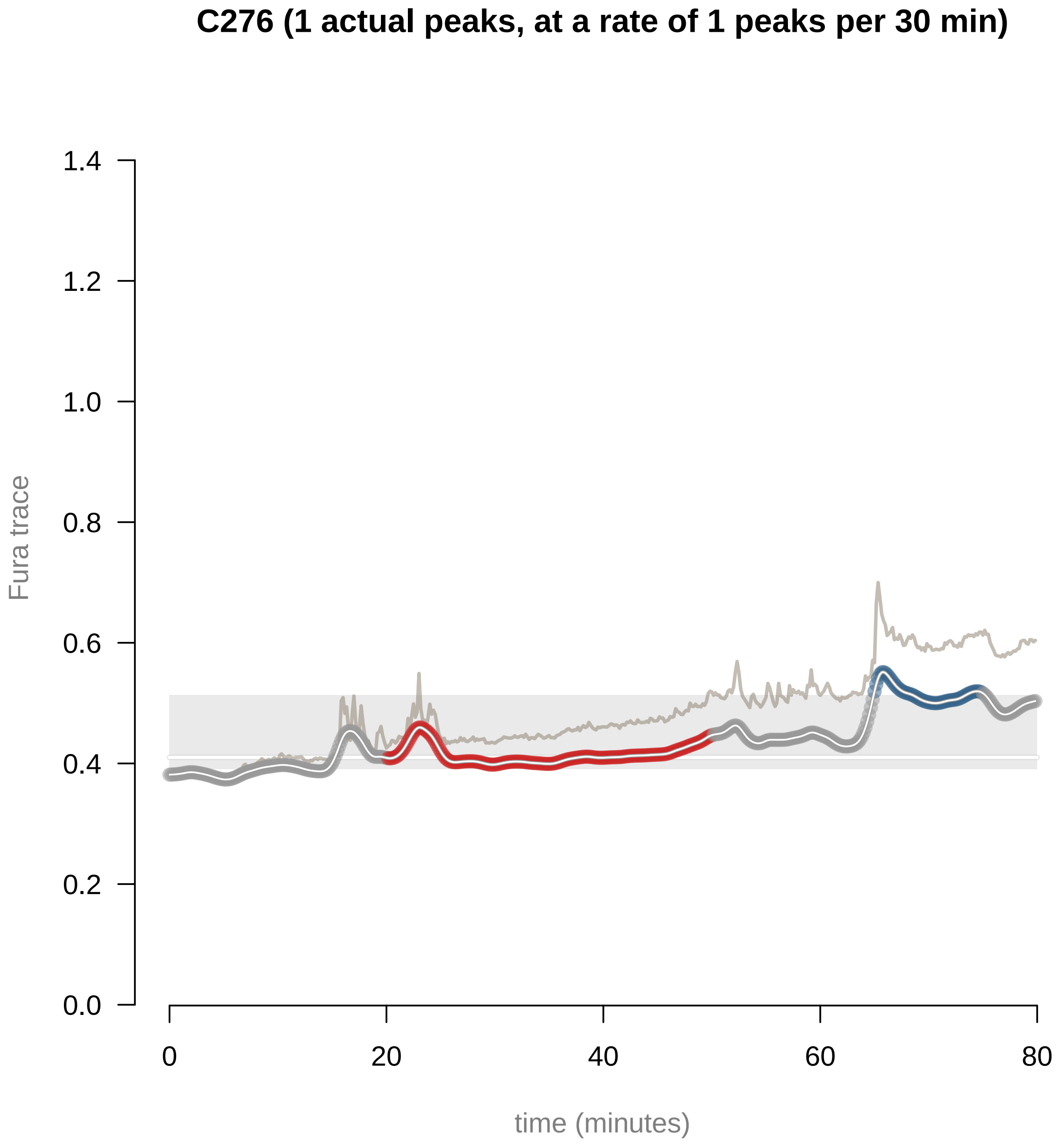
C276 (1 actual peaks, at a rate of 1 peaks per 30 min)
Fura trace
1.4
1.2
1.0
0.8
0.6
0.4
0.2
0.0
0
20
40
60
80
time (minutes)

C277 (3 actual peaks, at a rate of 2.65 peaks per 30 min)

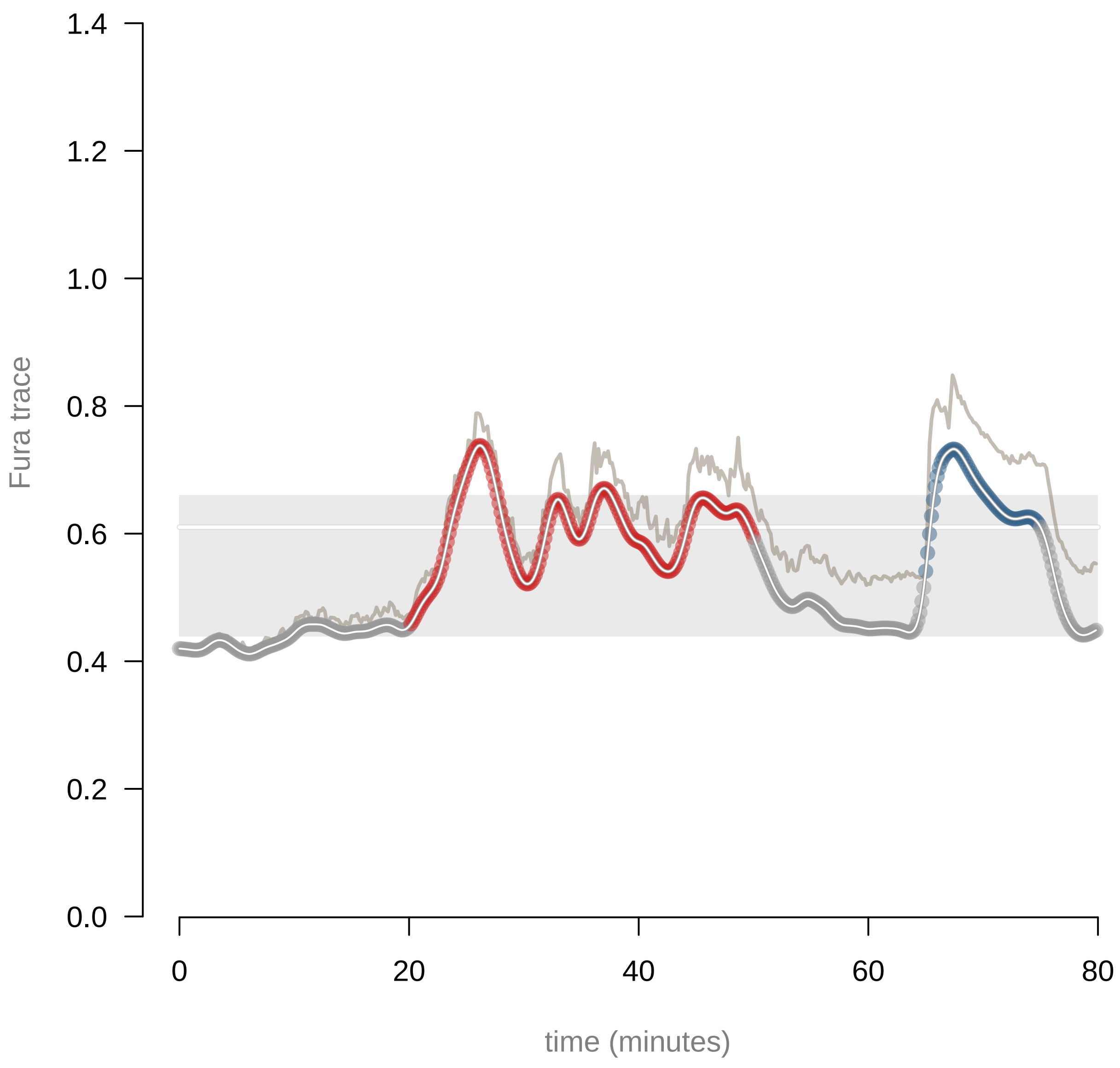

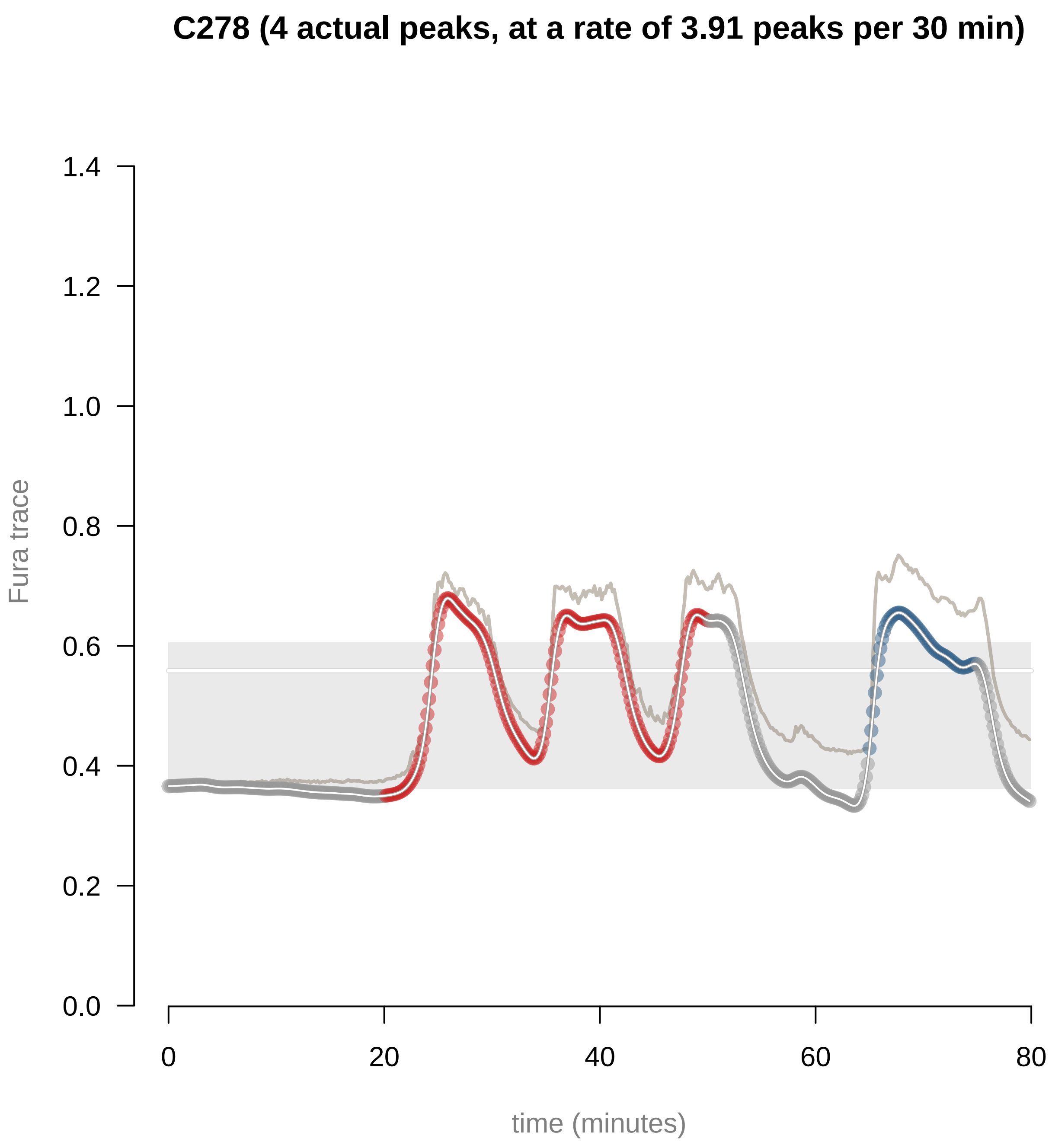

C278 (4 actual peaks, at a rate of 3.91 peaks per 30 min)
Fura trace
1.4
1.2
1.0
0.8
0.6
0.4
0.2
0.0
0
20
40
60
80
time (minutes)

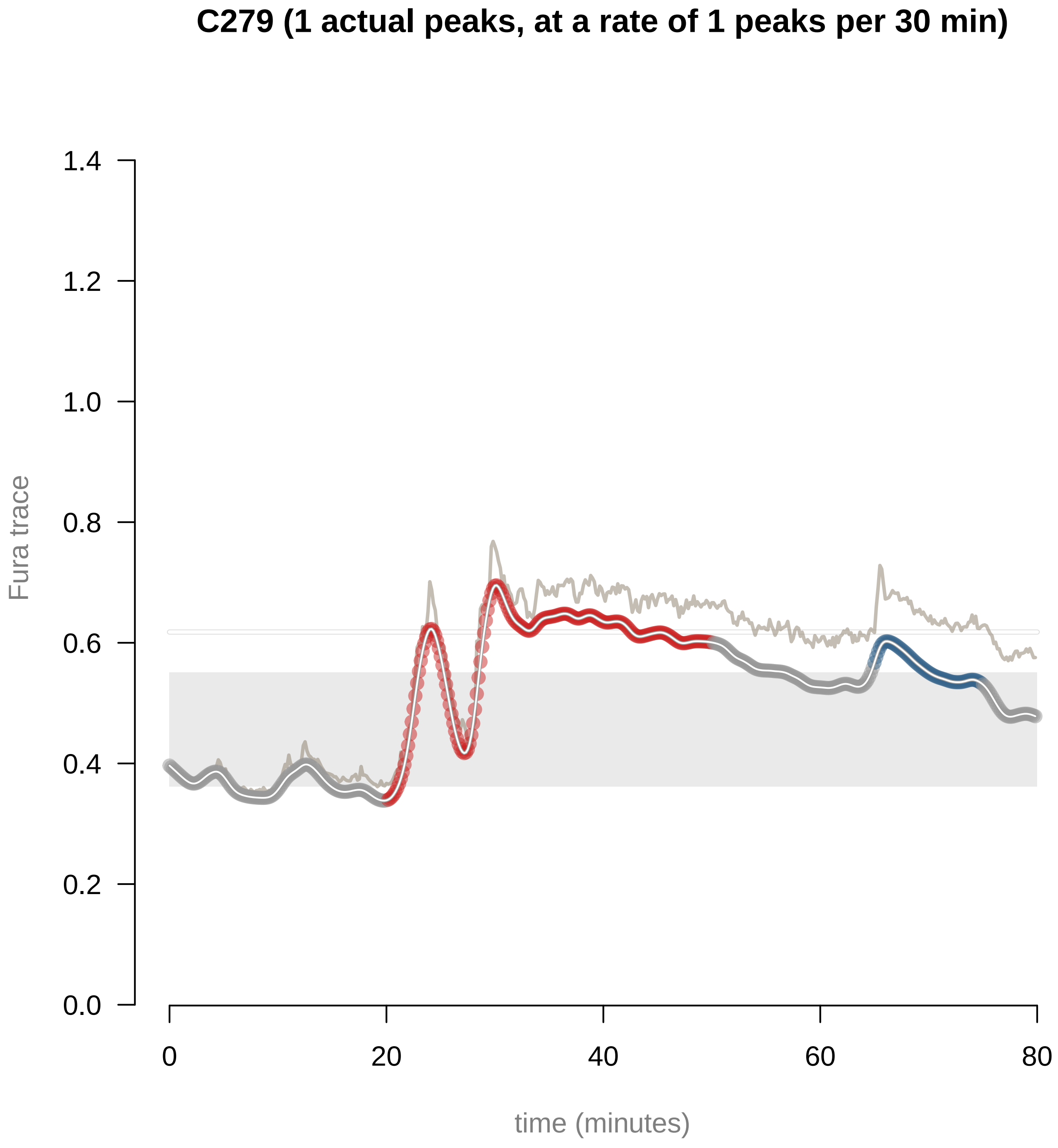

C279 (1 actual peaks, at a rate of 1 peaks per 30 min)
Fura trace
0.0
0.2
0.4
0.6
0.8
1.0
1.2
1.4
0
20
40
60
80
time (minutes)

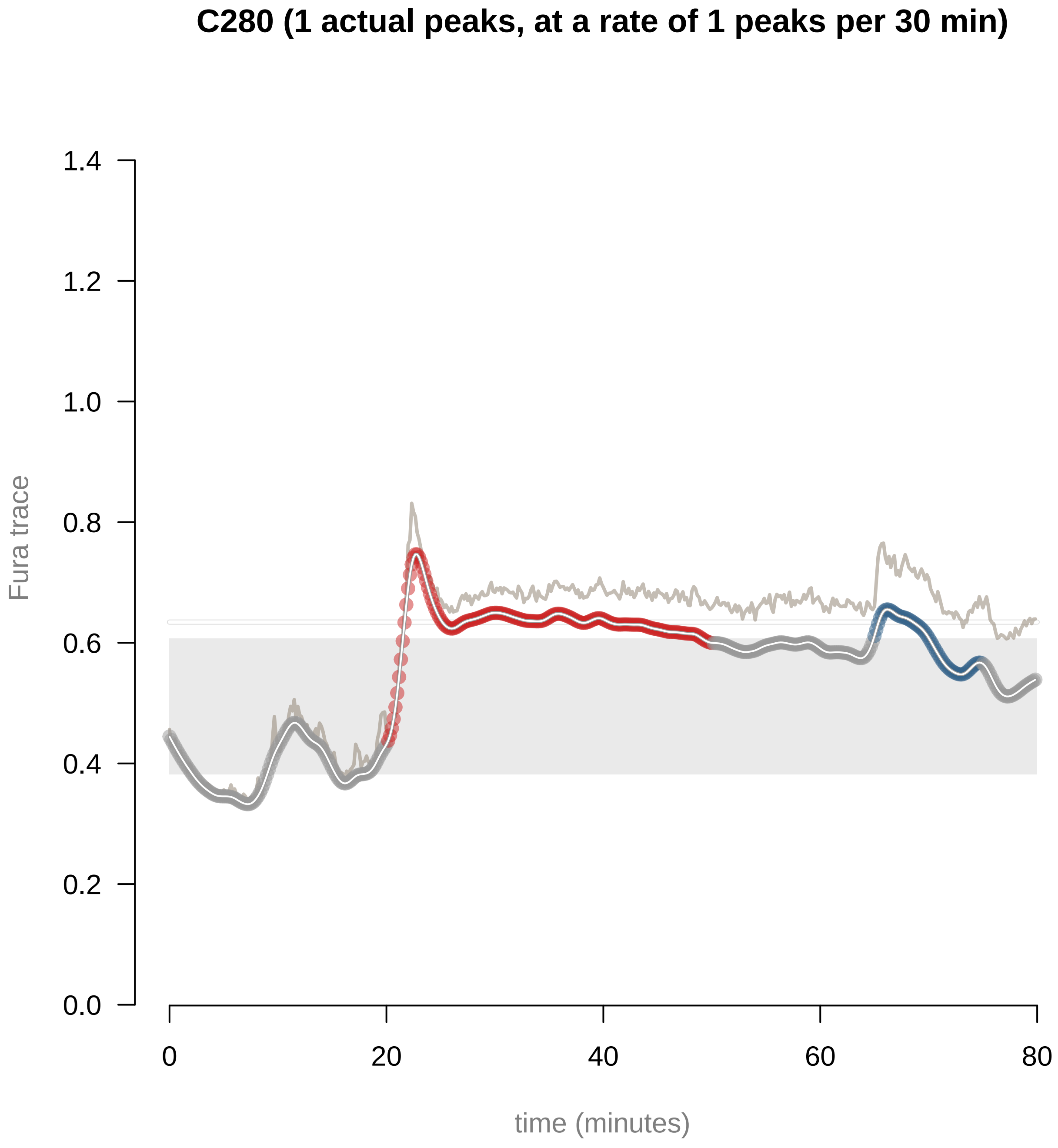

C280 (1 actual peaks, at a rate of 1 peaks per 30 min)
Fura trace
time (minutes)
0.0
0.2
0.4
0.6
0.8
1.0
1.2
1.4
0
20
40
60
80

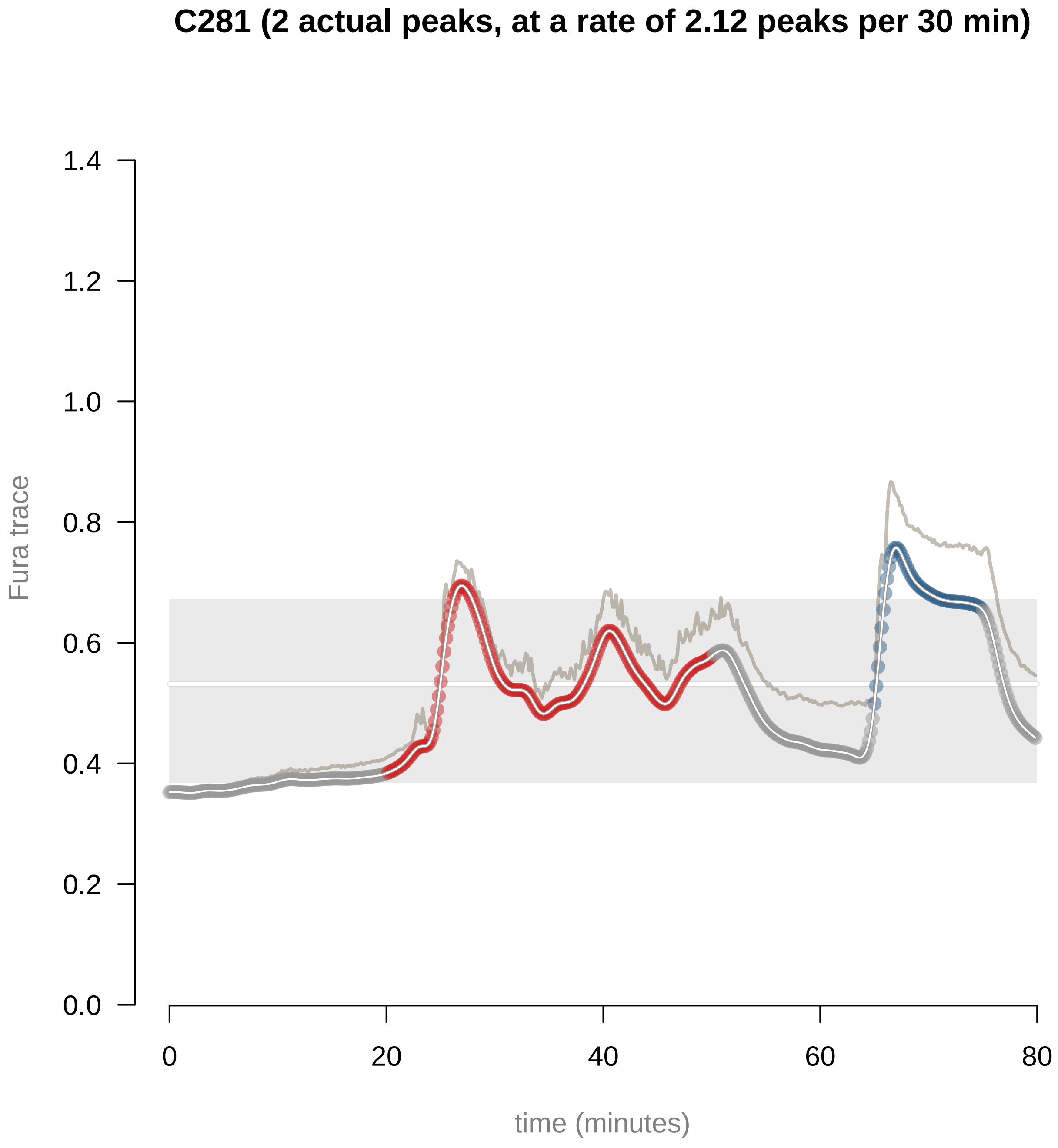
C281 (2 actual peaks, at a rate of 2.12 peaks per 30 min)
Fura trace
0.0
0.2
0.4
0.6
0.8
1.0
1.2
1.4
0
20
40
60
80
time (minutes)

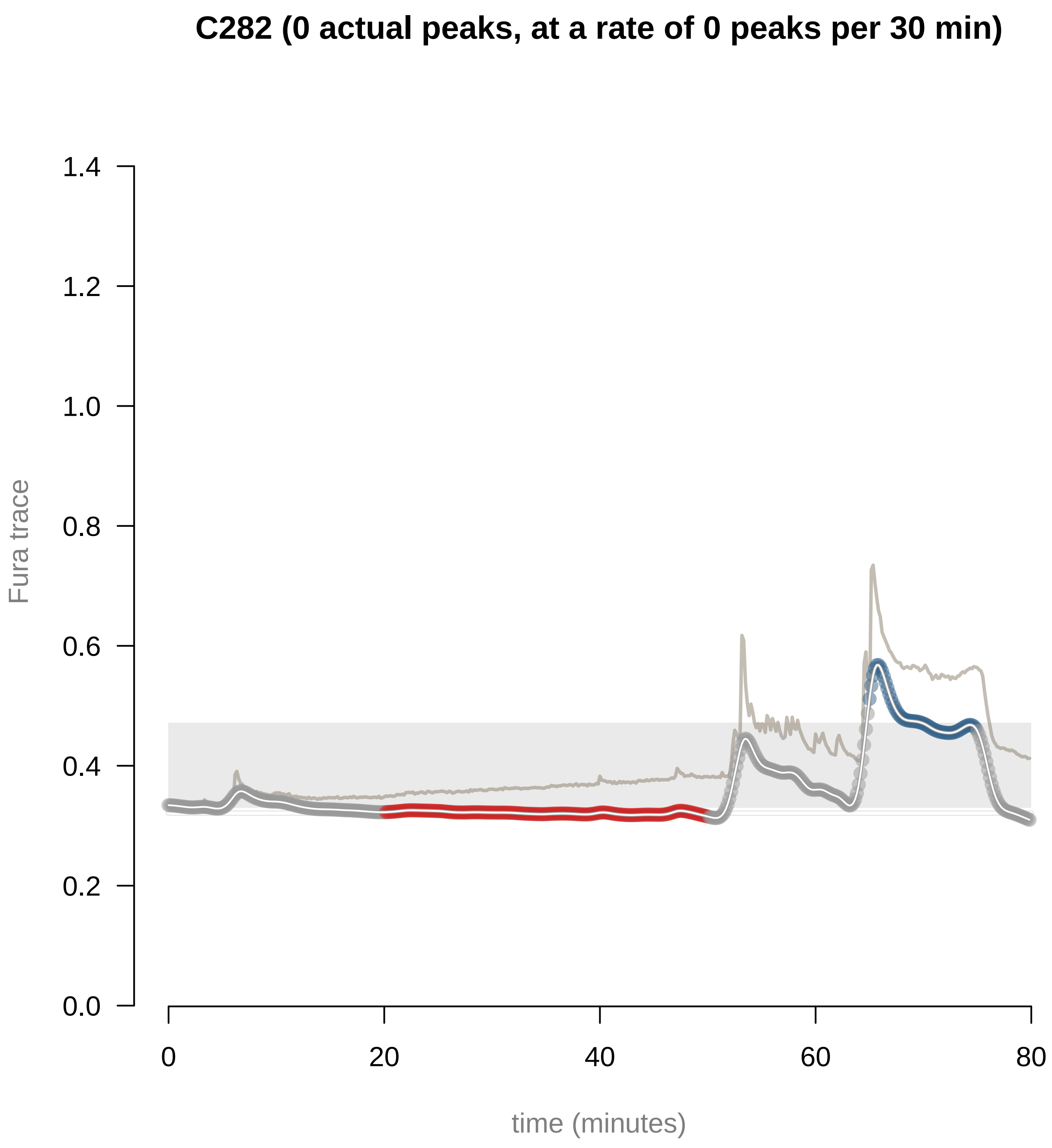
C282 (0 actual peaks, at a rate of 0 peaks per 30 min)
Fura trace
time (minutes)
0.0
0.2
0.4
0.6
0.8
1.0
1.2
1.4
0
20
40
60
80

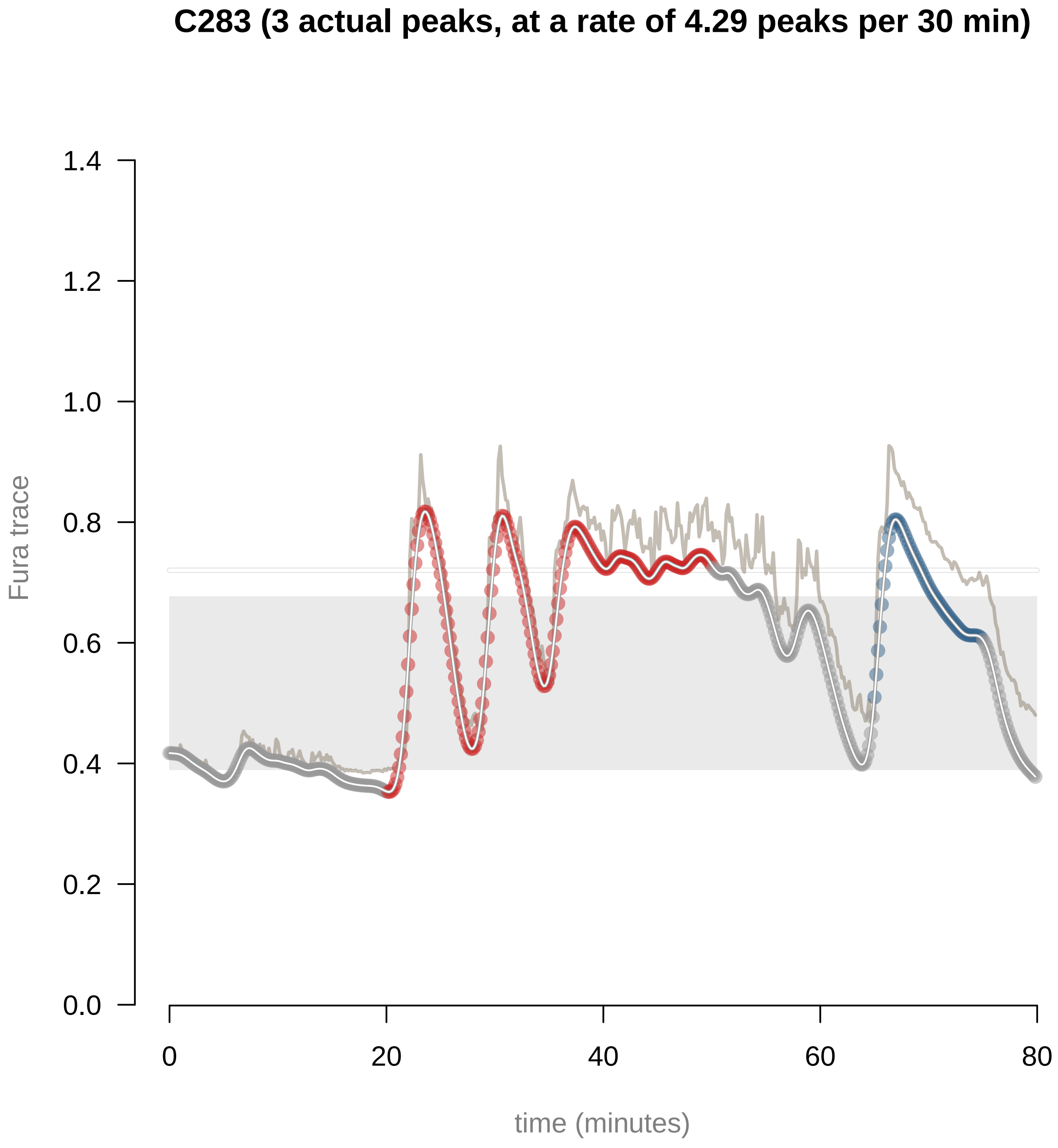
C283 (3 actual peaks, at a rate of 4.29 peaks per 30 min)
1.4
1.2
1.0
0.8
0.6
0.4
0.2
0.0
Fura trace
0
20
40
60
80
time (minutes)

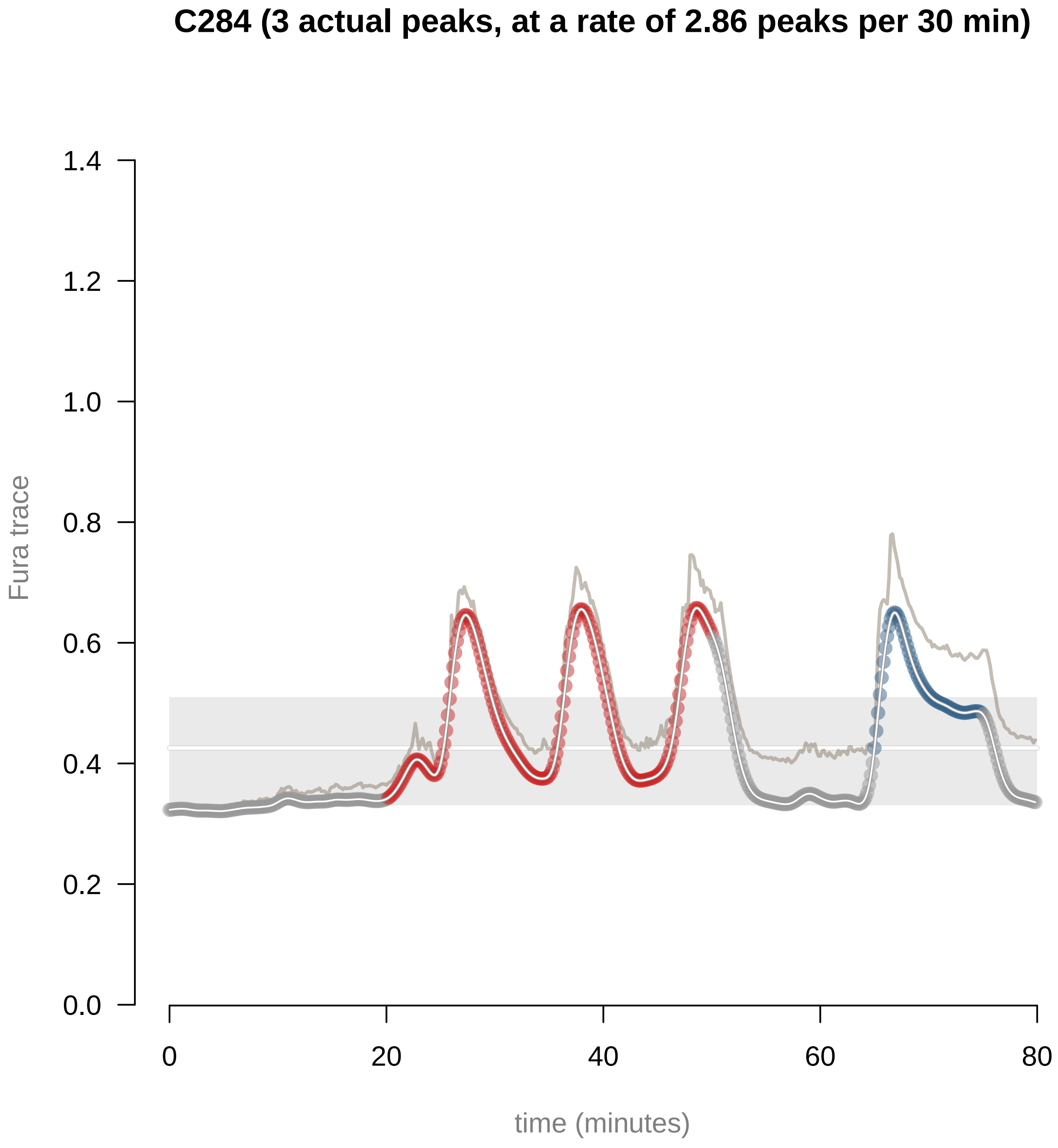

C284 (3 actual peaks, at a rate of 2.86 peaks per 30 min)
Fura trace
1.4
1.2
1.0
0.8
0.6
0.4
0.2
0.0
0
20
40
60
80
time (minutes)

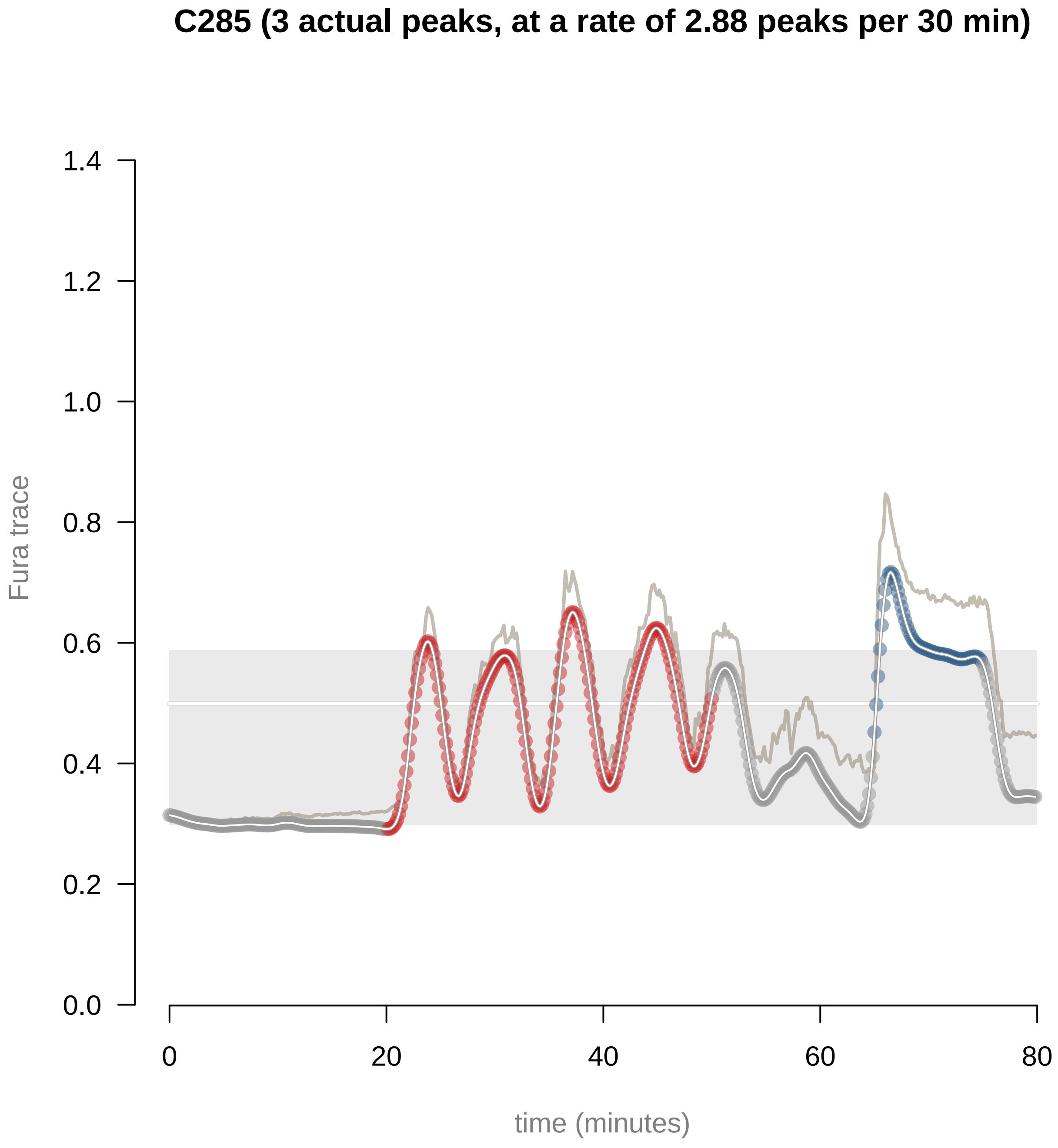

C285 (3 actual peaks, at a rate of 2.88 peaks per 30 min)
Fura trace
1.4
1.2
1.0
0.8
0.6
0.4
0.2
0.0
0
20
40
60
80
time (minutes)

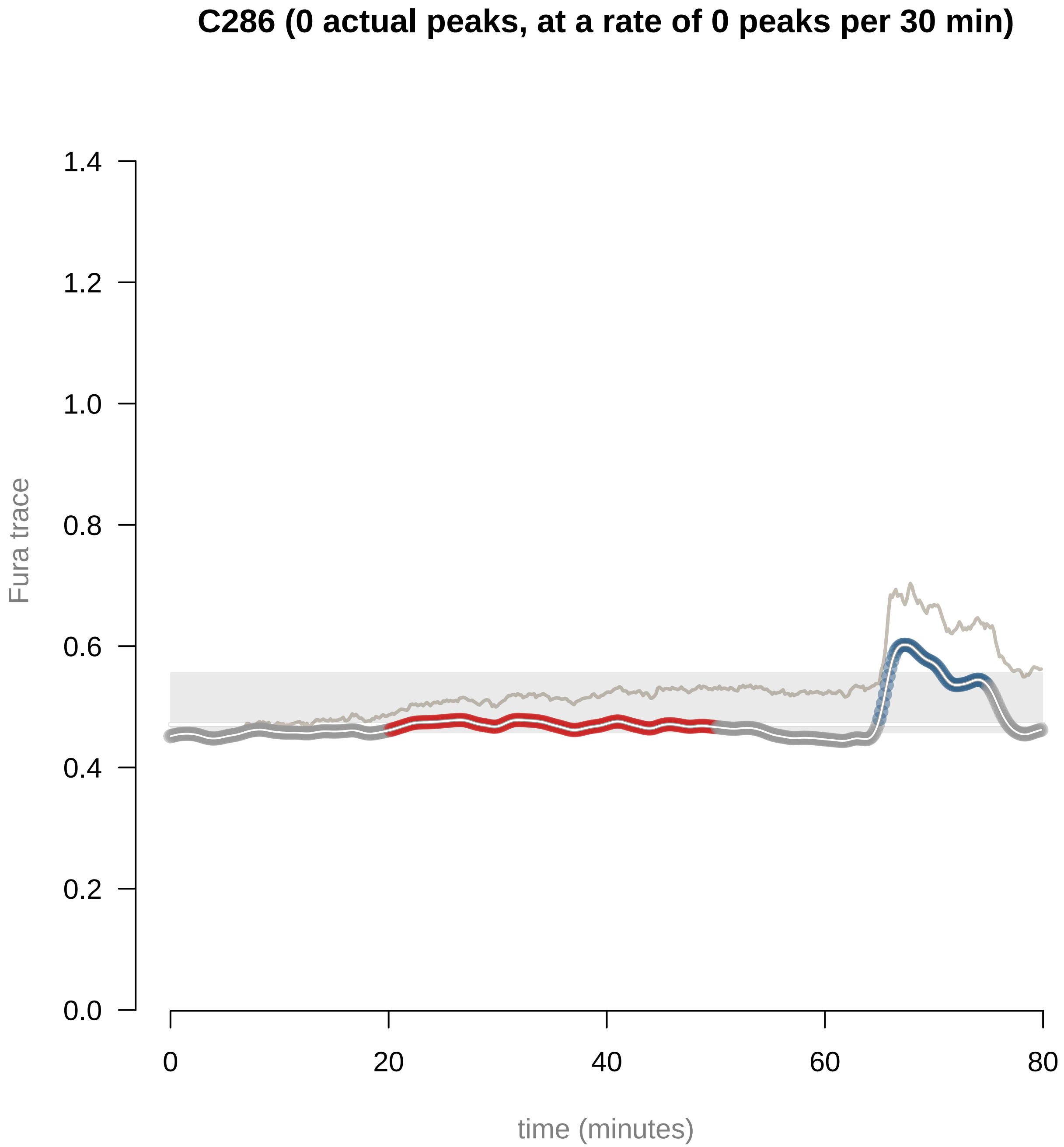
C286 (0 actual peaks, at a rate of 0 peaks per 30 min)
Fura trace
1.4
1.2
1.0
0.8
0.6
0.4
0.2
0.0
0
20
40
60
80
time (minutes)

C287 (2 actual peaks, at a rate of 3.6 peaks per 30 min)

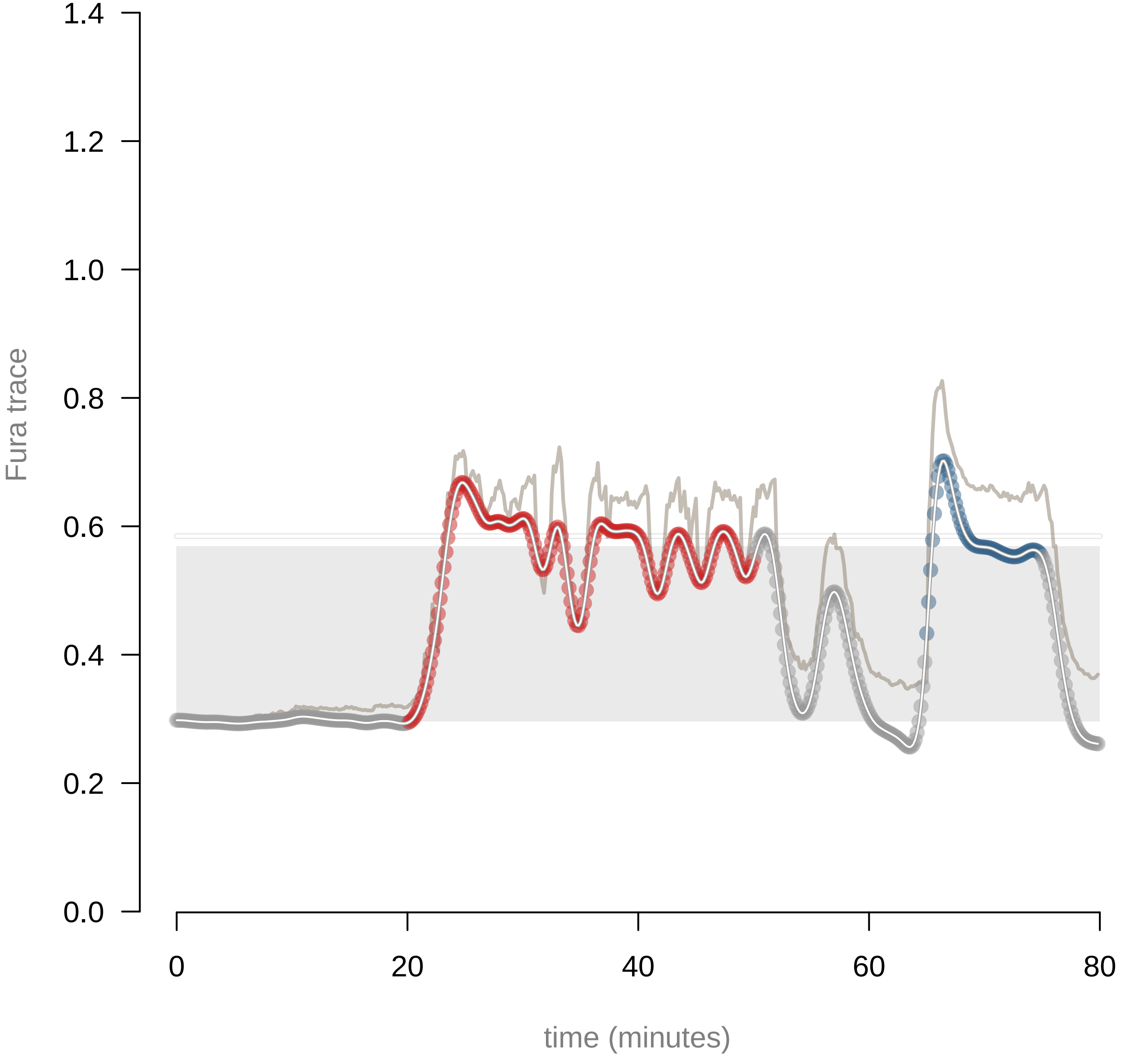

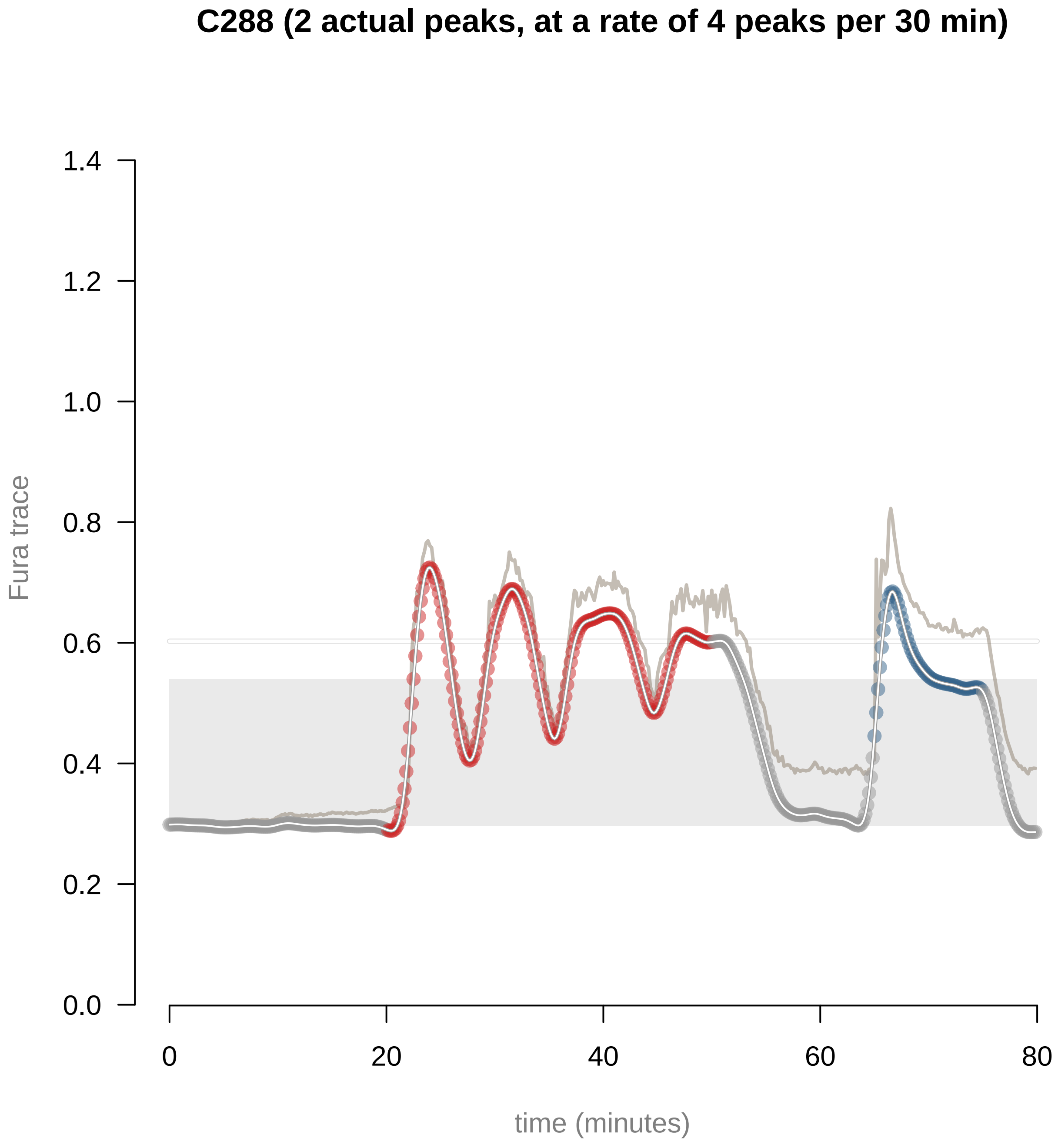
C288 (2 actual peaks, at a rate of 4 peaks per 30 min)
Fura trace
1.4
1.2
1.0
0.8
0.6
0.4
0.2
0.0
0
20
40
60
80
time (minutes)

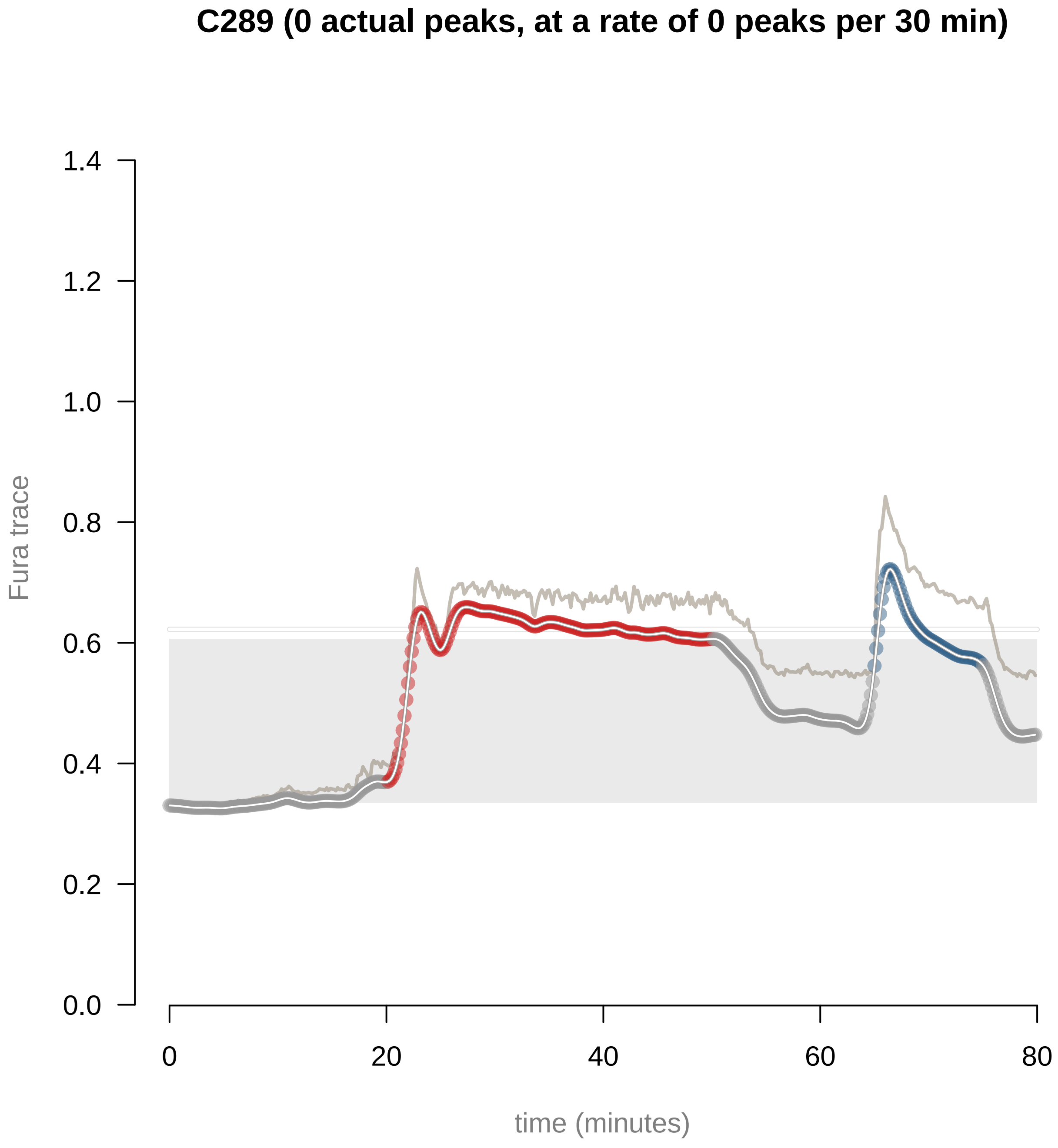

C289 (0 actual peaks, at a rate of 0 peaks per 30 min)
1.4
1.2
1.0
0.8
0.6
0.4
0.2
0.0
Fura trace
0
20
40
60
80
time (minutes)

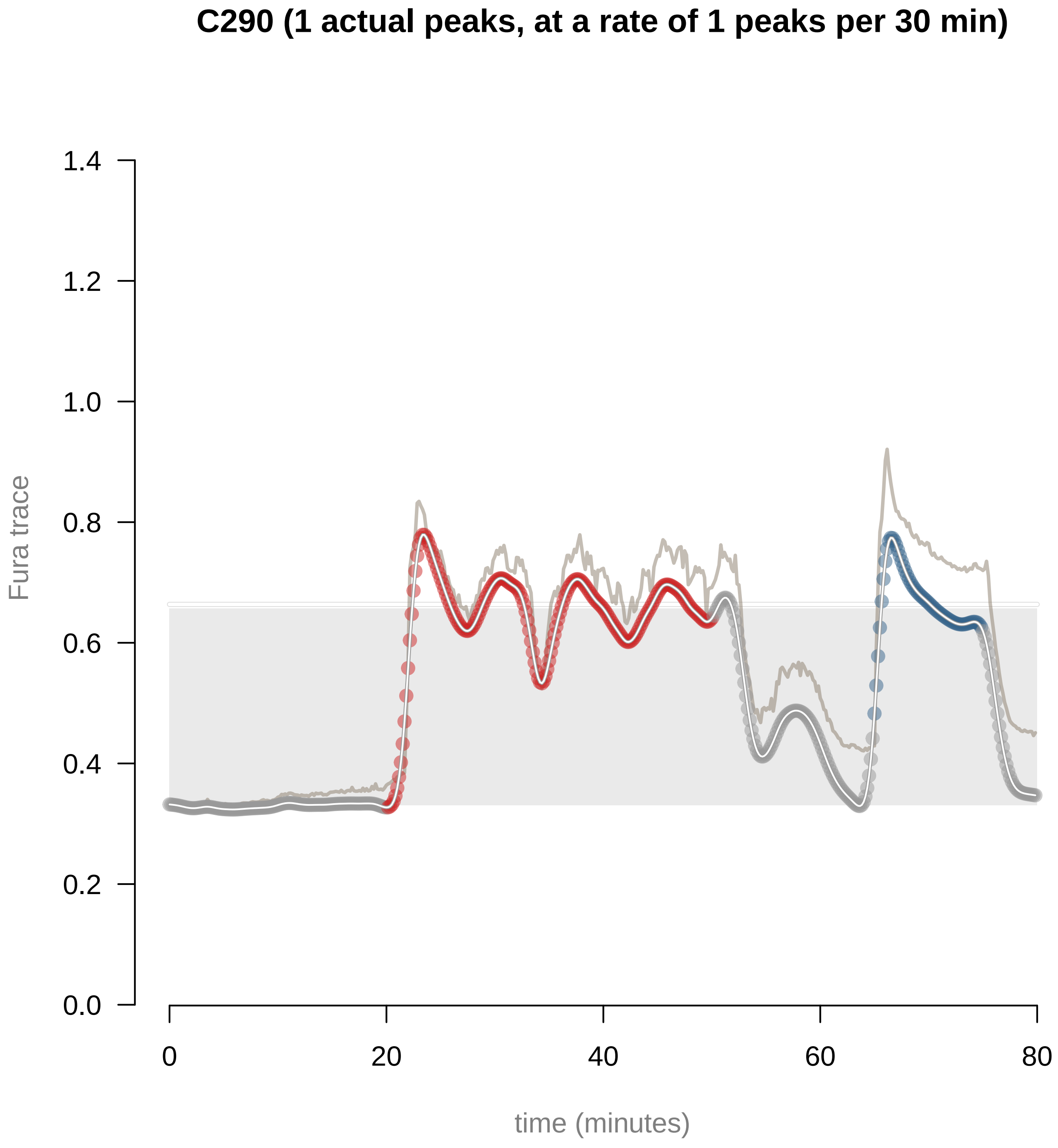
C290 (1 actual peaks, at a rate of 1 peaks per 30 min)
Fura trace
1.4
1.2
1.0
0.8
0.6
0.4
0.2
0.0
0
20
40
60
80
time (minutes)

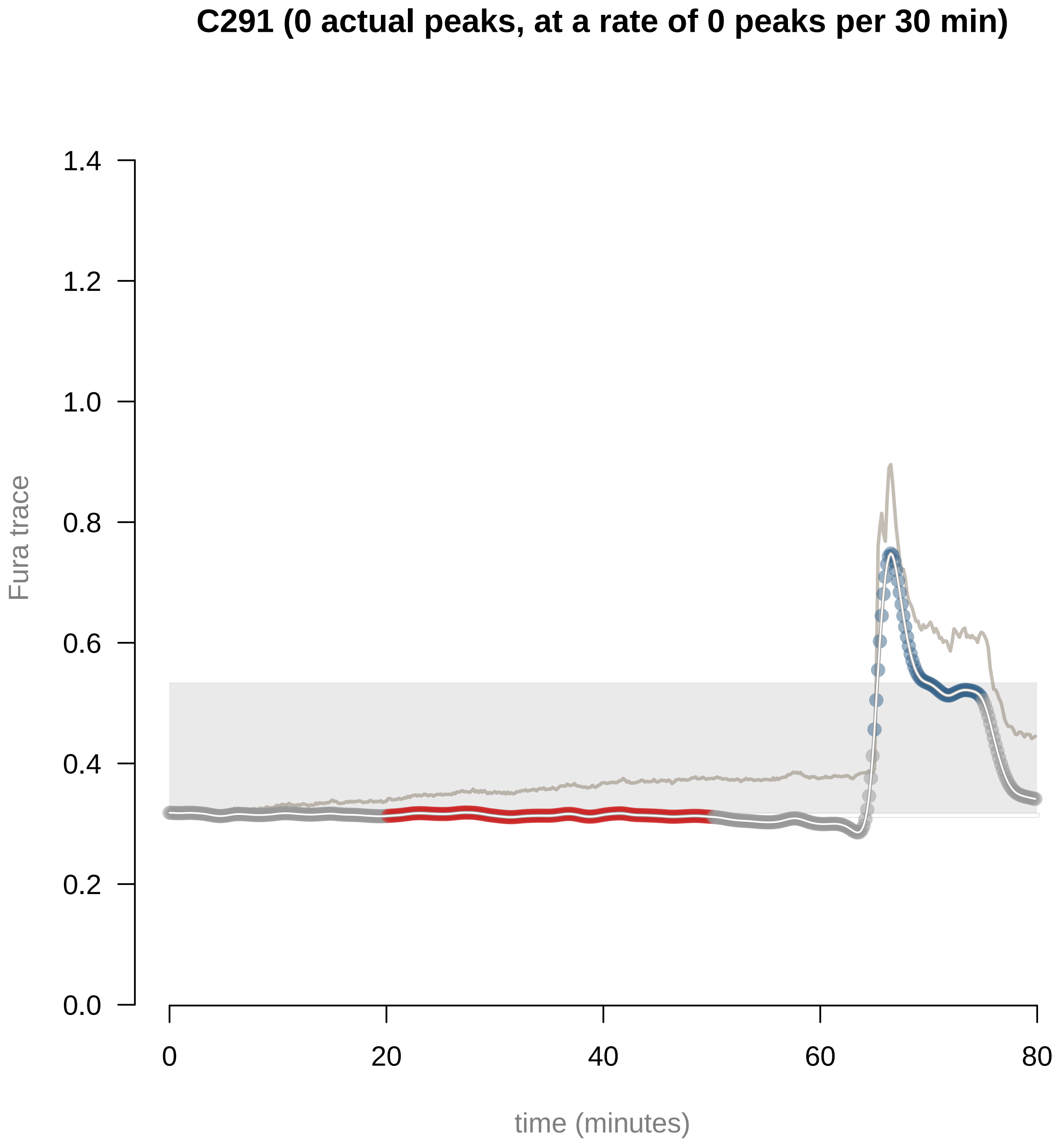
C291 (0 actual peaks, at a rate of 0 peaks per 30 min)
Fura trace
1.4
1.2
1.0
0.8
0.6
0.4
0.2
0.0
0
20
40
60
80
time (minutes)
