# Supplementary material for: Statistical approaches and software for clustering islet cell functional heterogeneity
Source: Islets. 2016 Feb 24;8(2):48–56. doi: 10.1080/19382014.2016.1150664 (PMC4878268; doi:10.1080/19382014.2016.1150664)
Supplement: Supplementary_data.zip [file kisl-08-02-1150664-s001.zip › Supplementary figures.pdf]

**Statistical approaches and software for clustering islet cell functional heterogeneity**

Quin F. Wills[1,2], Tobias Albrecht[3], Ali Asadi[3], Ziliang Ao[4], Garth L. Warnock[4], Timothy J. Kieffer[3,4], James D. Johnson[3,4]

[1] Wellcome Trust Centre for Human Genetics, University of Oxford, Oxford, United Kingdom

[2] Weatherall Institute of Molecular Medicine, University of Oxford, Oxford, United Kingdom

[3] Department of Cellular and Physiological sciences, Life Sciences Centre, University of British Columbia, Vancouver, Canada

[4] Department of Surgery, University of British Columbia, Vancouver, Canada

**SUPPORTING INFORMATION**

## DATA AND ANALYSIS

**Data overview.** The available data comprises Fura-2 ratiometric fluorescence values for 276 human islet cells from a single subject, imaged in three separate microscopy fields (**Fig 1** below). 480 values were generated, QC'ed and normalized for each cell over 80 minutes as described the accompanying manuscript. The data is available online as **S1 Data** and **S1 Software**. All post-imaging data was processed and analysed within the 64 bit R environment (version 3.0.2). The mclust library (version 4.4) was used for Gaussian mixture model clustering of the Fura-2 trace features.

**Data normalization and filtering**. Minor signal drift was noticed over 80 minutes of imaging and was corrected per cell by estimating median increase in fluorescence signal per time step under baseline conditions (first 20 minutes at 3mM glucose). 15 cells were filtered from further analysis due to poor overall signal response, defined as a median KCl response less than 5% over median baseline signal. Plots of the individual traces are provided as **S1 File**, where the raw signal is show together with the smoothed drift-corrected signal. In each plot of **S1 File** the grey rectangle shows the range of median baseline signal to median KCl signal, while the white horizontal line shows median signal during high glucose (20 mM) exposure.

**Exploratory data analysis.** Classical multi-dimensional scaling (MDS) was used to explore projections of the Fura-2 traces. The distance matrix for the projections was calculated as $(1-\rho_{ij})/2$, with $\rho_{ij}$ being the Spearman correlation between cell *i* and *j.* As described in the manuscript, mean silhouette width of the cell imaging fields for each dimension suggested non-homogeneous dispersion of the cells. The silhouette width of each cell per dimension was calculated as $(d-f)/\max\{d,f\}$, where *d* represents its mean distance with cells in other imaging fields and *f* represents its mean distance with cells in the same imaging field. Despite this observation, no consistent spatial effect could be found within each field (**Fig 2** below).

**Cell feature clustering.** As described in the manuscript, Fura-2 trace features were extracted and both non-parametrically and parametrically clustered. The correlations between these features are shown in **Fig 3** below. The non-parametric clustering approach was complete agglomerative hierarchical clustering, while the two-dimensional Gaussian mixtures were allowed to be ellipsoidal and of varying volume, shape, and orientation.

**SOFTWARE**

A software app called TraceCluster is available both online (**https://jimjohnsonsci.shinyapps.io/TraceCluster**) and for download as **S1 Software** to explore the data and allow users to upload their own data for comparison. The app is fully graphical, requiring no coding expertise. However to run the downloaded version locally requires the installation of R (**https://www.r-project.org**) and the Shiny package (**https://cran.r-project.org/web/packages/shiny/index.html**). If running locally, unzip **S4 Software**, and enter the following in an R session, modifying the file path to be the file path to your unzipped data:

```
shiny::runApp("file_path",launch.browser=T)
```

An HTML graphical interface will open in your default web browser, allowing you to explore the cells by features such as magnitude and frequency of oscillations. If you have generated calcium flux data under similar conditions, the software is also able to analyse your data and directly compare against the data in this study, as shown in **Fig 4** below. Detailed instructions are provided in the sofware.

# FIGURES

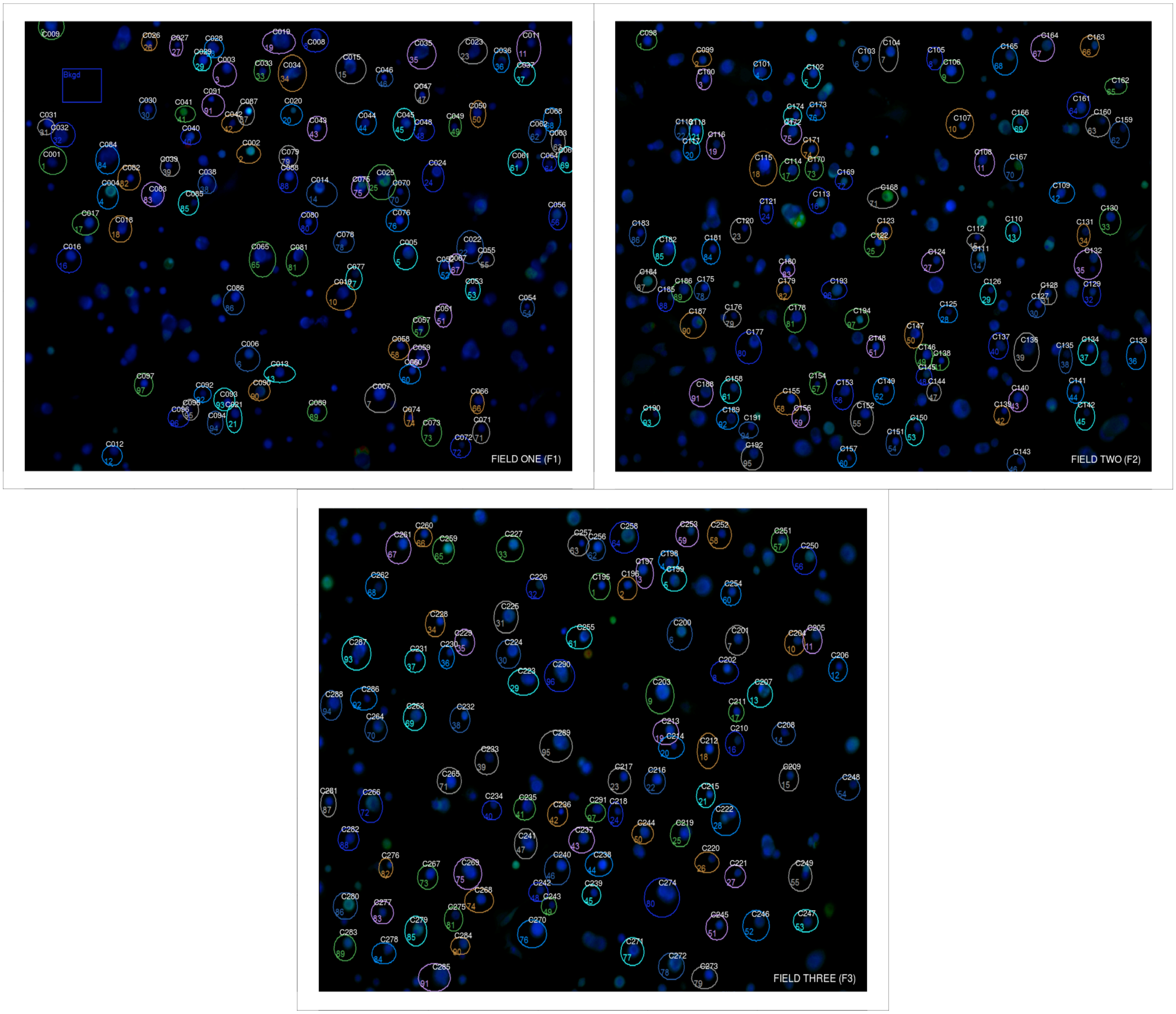


**Figure 1. Imaging fields for the ~300 dispersed islet cells**

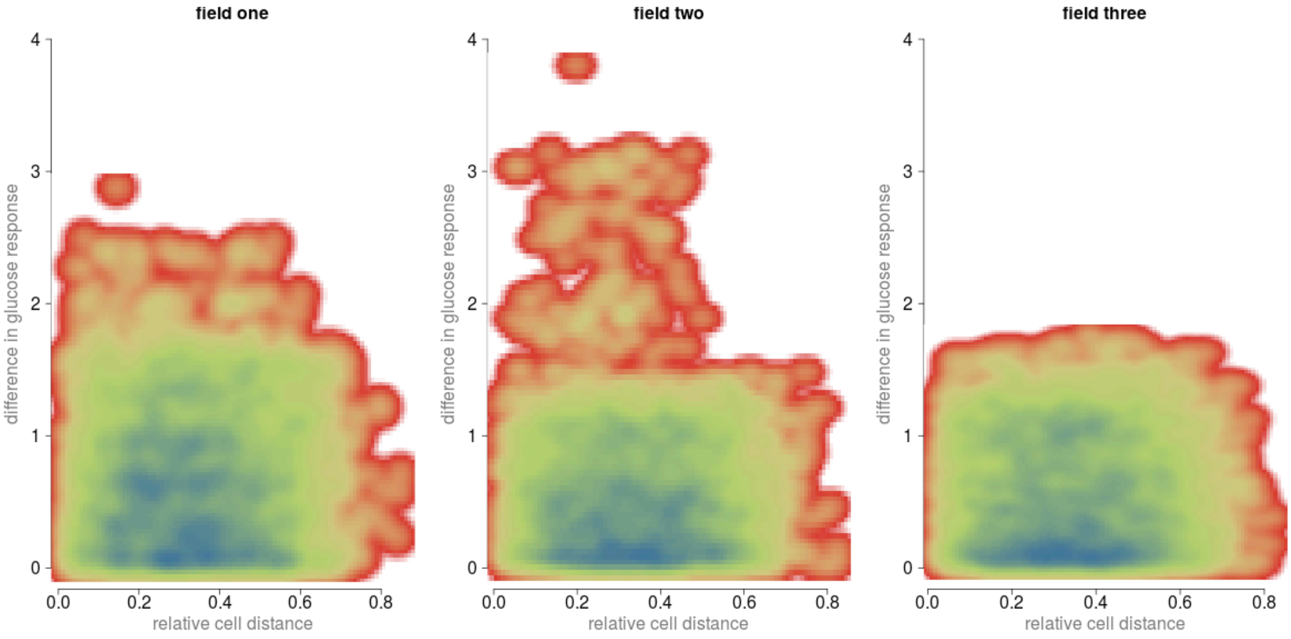


**Figure 2. Spatial effects in the data.** Non-homogeneous cell dispersion was suggested with exploratory data analysis. We considered if cells with very similar (or different) behaviours were more likely to be closer together within fields, but could not observed reproducible trends. The above 2D density plots for pairwise difference in glucose response and spatial distance indicate greater differences in imaging field one because of the greater proportion of non-responders. The cluster in field two was largely due to a single strong glucose responder within the centre of the field.

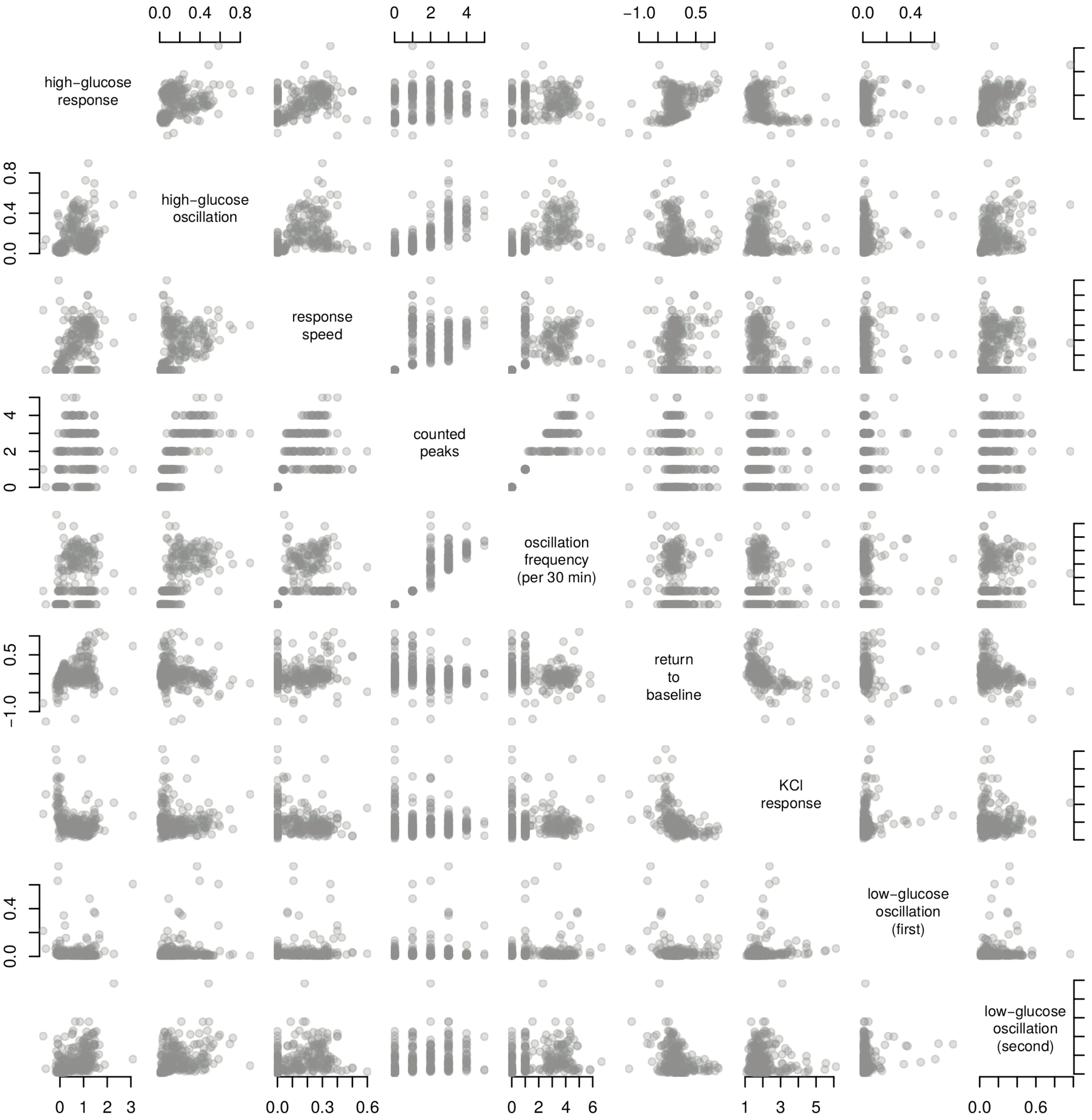


**Figure 3. Correlations between the nine extracted calcium trace features.**

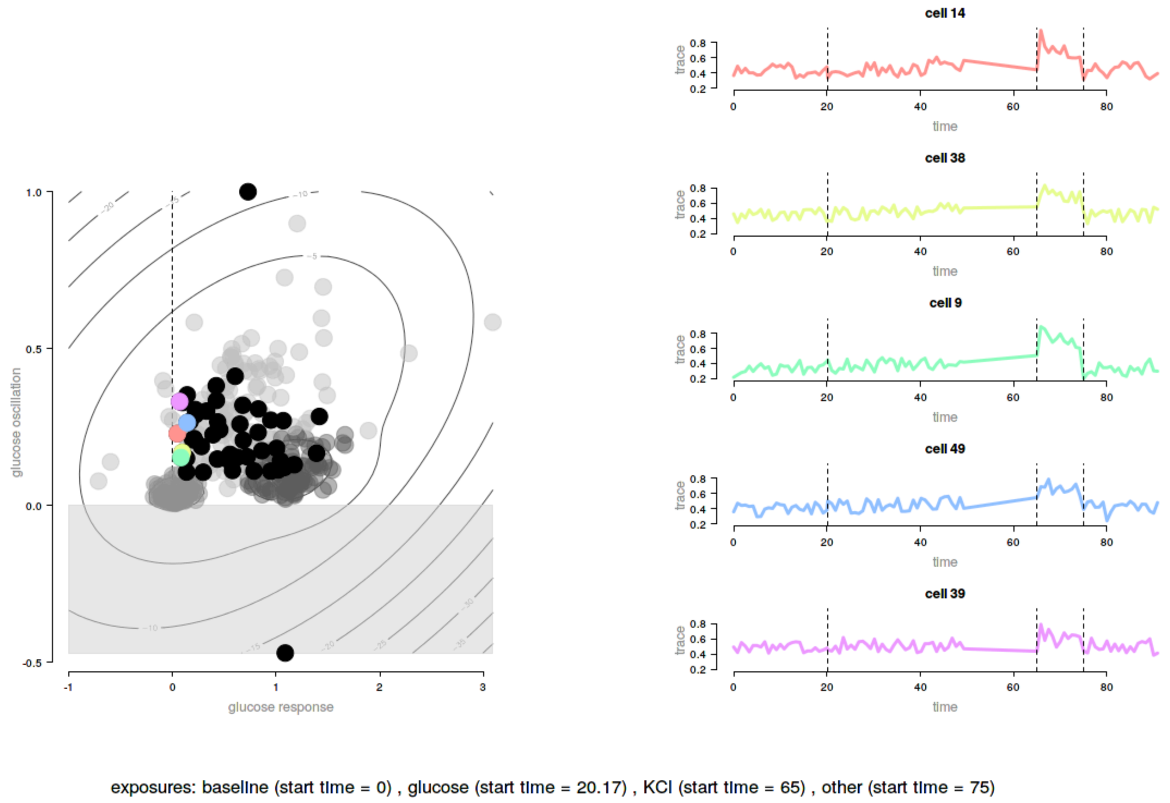


**Figure 4. Software comparative analysis.** The provided software app allows of the upload of similar data for comparison against the reference data and results presented in this study. An example is provided above where the left plot shows the reference data in grey (see **Fig 4** in the manuscript) with results for new data plotted over. The user is able to select cells, wich are shown in colour, with their traces plotted to the right. Instructions for the upload of data and interpretation of results are provided in the software.

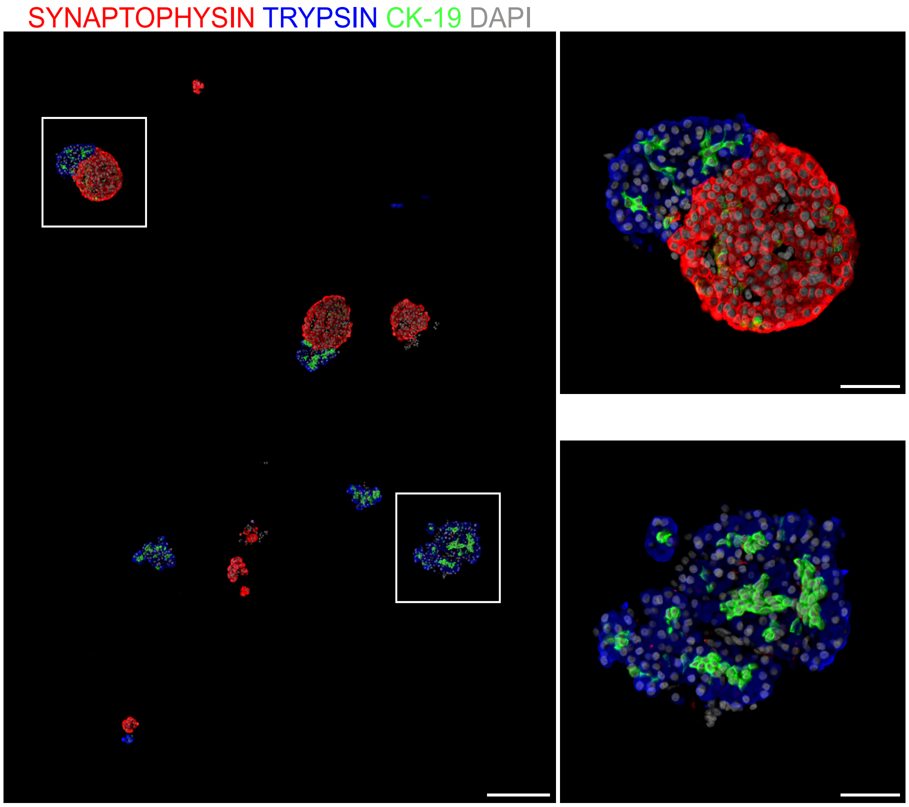


**Figure 5. Endocrine versus exocrine cell types from the HR196 donor.** [top] Analysis of endocrine and exocrine cell types. [bottom] Example of typical human islet cell composition. Insulin, glucagon and PDX1 staining in a typical batch of human islets (50 year old donor, HR 117).
